# Integration of full-length transcriptomics and targeted metabolomics to identify benzylisoquinoline alkaloid biosynthetic genes in *Corydalis yanhusuo*

**DOI:** 10.1038/s41438-020-00450-6

**Published:** 2021-01-10

**Authors:** Dingqiao Xu, Hanfeng Lin, Yuping Tang, Lu Huang, Jian Xu, Sihui Nian, Yucheng Zhao

**Affiliations:** 1 Key Laboratory of Shaanxi Administration of Traditional Chinese Medicine for TCM Compatibility, State Key Laboratory of Research & Development of Characteristic Qin Medicine Resources (Cultivation), Shaanxi Key Laboratory of Chinese Medicine Fundamentals and New Drugs Research, and Shaanxi Collaborative Innovation Center of Chinese Medicinal Resources Industrialization, Shaanxi University of Chinese Medicine, 712046 Xi’an, Shaanxi China; 2 Department of Resources Science of Traditional Chinese Medicines and State Key Laboratory of Natural Medicines, School of Traditional Chinese Pharmacy, China Pharmaceutical University, 210009 Nanjing, Jiangsu China; 3 Institute of Modern Chinese Medicine, School of Pharmacy, Wannan Medical College, 241002 Wuhu, Anhui China

**Keywords:** Sequencing, Metabolomics

## Abstract

*Corydalis yanhusuo* W.T. Wang is a classic herb that is frequently used in traditional Chinese medicine and is efficacious in promoting blood circulation, enhancing energy, and relieving pain. Benzylisoquinoline alkaloids (BIAs) are the main bioactive ingredients in *Corydalis yanhusuo*. However, few studies have investigated the BIA biosynthetic pathway in *C. yanhusuo*, and the biosynthetic pathway of species-specific chemicals such as tetrahydropalmatine remains unclear. We performed full-length transcriptomic and metabolomic analyses to identify candidate genes that might be involved in BIA biosynthesis and identified a total of 101 full-length transcripts and 19 metabolites involved in the BIA biosynthetic pathway. Moreover, the contents of 19 representative BIAs in *C. yanhusuo* were quantified by classical targeted metabolomic approaches. Their accumulation in the tuber was consistent with the expression patterns of identified BIA biosynthetic genes in tubers and leaves, which reinforces the validity and reliability of the analyses. Full-length genes with similar expression or enrichment patterns were identified, and a complete BIA biosynthesis pathway in *C. yanhusuo* was constructed according to these findings. Phylogenetic analysis revealed a total of ten enzymes that may possess columbamine-O-methyltransferase activity, which is the final step for tetrahydropalmatine synthesis. Our results span the whole BIA biosynthetic pathway in *C. yanhusuo*. Our full-length transcriptomic data will enable further molecular cloning of enzymes and activity validation studies.

## Introduction

*Corydalis yanhusuo* W.T. Wang is a medicinal herb in the *Corydalis* genus of the Papaveraceae family. In traditional Chinese medicine, its dried tubers (called “Yan-Hu-Suo” or “Yuan-Hu”) are frequently used as an important therapeutic agent, with well-recorded efficacy in promoting blood circulation, enhancing energy, and providing analgesia^1^. Recent pharmacological research has found that active compounds in *C. yanhusuo* can inhibit tumor cell proliferation^2^; attenuate acute, inflammatory and neuropathic pain^1^; reduce drug addiction^3,4^; and treat cardiovascular diseases^5–7^. Owing to the low productivity of tubers and the short life cycle of *C. yanhusuo*, plant material sources are limited, greatly impeding its further application. Synthetic biology, which harnesses bacteria as chemical synthesis factories, has been working to provide an alternative to circumvent this problem^8,9^. However, little is known about the biosynthetic pathways of active compounds in *C. yanhusuo*, especially its protoberberine- and aporphine-type benzylisoquinoline alkaloids (BIAs).

Characterization of the chemical composition of *C. yanhusuo* has been ongoing for nearly a century^10^, and a series of compounds have been identified, including sugars, amino acid derivatives, triterpenes, anthraquinones, phenolic acids, steroids and organic acids^11^. Additionally, over 60 kinds of alkaloids have been identified in *C. yanhusuo* tubers^10–14^, most of which are BIAs and produce the main pharmacological effects^15,16^. These compounds are all synthesized from tyrosine and undergo a common precursor pathway through the synthesis of (S)-reticuline before diverging into different BIA classes^17^. Among these BIAs, most can be classified as protoberberines and aporphines according to their chemical structures^14^, but protopine-type and benzo[c]phenanthridine-type BIAs are also present in *C. yanhusuo* bulb extracts. Currently, thanks to the persistent efforts of biochemists and phytochemists, the labyrinthine BIA biosynthetic pathways have been well elucidated in the model species *Papaver somniferum* (opium poppy)^17,18^, but the clarification of the aporphine-type BIA biosynthetic pathway will require substantial additional efforts^19^. Additionally, a few amino acid sequences of key enzymes in protoberberine BIA pathways are still missing, which greatly impedes the molecular cloning and metabolic engineering of protoberberines. Furthermore, little attention has been given to the BIA biosynthetic process in *Corydalis*, which has resulted in a lack of knowledge about the biosynthesis of several pharmacologically active alkaloids in *C. yanhusuo*, such as glaucine, corybulbine^20^, and dehydrocorydaline^21^.

To date, research has mainly focused on the pharmacological effects of *C. yanhusuo* extracts and the chemicals isolated from these extracts. Despite its medicinal importance, researchers have paid little attention to the genetics of this plant. Although there are reports regarding the transcriptomes of BIA-producing plants^22–26^, short-read-length NGS without reference genomes generally depends on de novo assembly, which has relatively poor performance in complex regions with repetitive or heterozygous sequences and thus prevents the retrieval of full-length transcript information^27^. Moreover, although one previous study investigated the time-course of BIA production during bulb development^26^, the spatial distribution of transcripts in *C. yanhusuo* remains unknown.

Here, we used single-molecule real-time (SMRT) sequencing on the PacBio Sequel platform, which produces long reads, to fully mine full-length transcriptome information in *C. yanhusuo*. The low raw accuracy of SMRT sequencing can be overcome by the use of NGS short reads to proofread SMRT^28^. SMRT sequencing can offer highly complete transcriptome data for further analysis^29,30^, paving the way for reconstructing transcripts, detecting splicing events, and circumventing computational challenges in NGS data assembly^31^. Metabolic profiling or metabolomics can monitor and quantify metabolite variations in response to both endogenous and exogenous factors in biological systems^29,30^. Broad-scope metabolomics encompassing both primary and secondary metabolism has been largely restricted to model plant species. In this study, the spatial variation of *C. yanhusuo* transcripts in leaves and tubers provides evidence for BIA biosynthesis in tubers rather than in leaves, and high-resolution mass spectrometry applied for alkaloid analysis identified 19 metabolites involved in BIA biosynthesis in the leaves and tubers of *C. yanhusuo*. A targeted metabolomics study of 19 alkaloids was conducted for the BIA accumulation pathway, with special focus on the alkaloids whose content in tubers was four times higher than that in leaves. In tandem with our key full-length transcriptome analysis, this work elucidates the major BIA biosynthesis pathways and their transcriptional regulation among different organs in *C. yanhusuo*.

## Results

### Sequencing and functional annotation

**Fig. 1:**
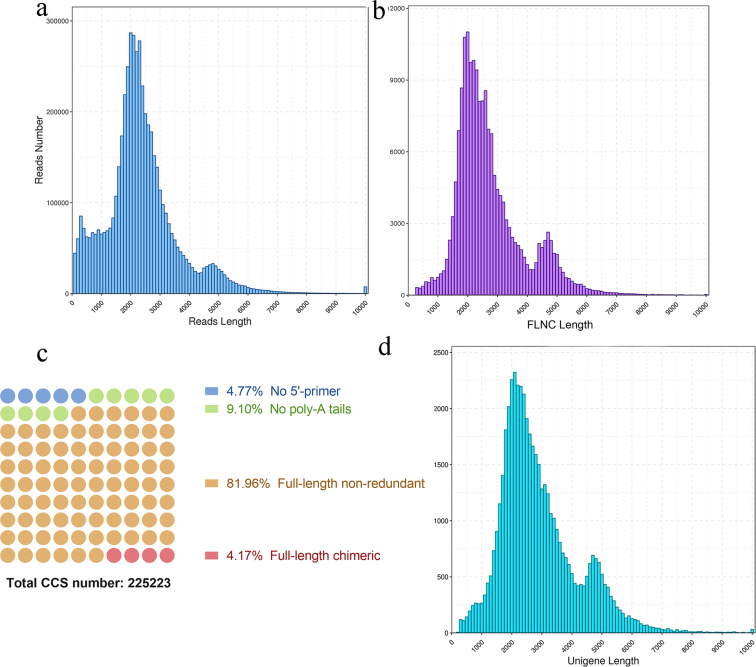
The quality control data for the full-length transcriptome of *C. yanhusuo* obtained by SMRT sequencing on the PacBio sequel platform. **a** The distribution of raw data subread length for SMRT sequencing. **b** The length distribution of full-length nonchimeric (FLNC) reads containing 5ʹ/3ʹ-primers and poly-A tails. **c** The quality of circular consensus sequences (CCS) exhibited as the proportion of FLNC reads. **d** The length distribution of high-quality, full-length, and polished unigene sequences that were proofread by SGS reads

To identify the genes involved in BIA synthesis in *C. yanhusuo*, we obtained the full-length transcriptome by performing SMRT sequencing on the PacBio Sequel platform. The statistics of the SMRT sequencing and comparison with previously reported Illumina sequencing^26^ are listed in Table 1. It produced a total of 5,228,902 subreads (12.24 GB), with an average length of 2341 bp, and the N50 length of all subreads was 2619 bp, much longer than that of Illumina sequencing (Fig. 1a). To improve the quality of the subreads, a total of 225,223 circular consensus sequences (CCSs) were found. The FLNC (full-length nonchimeric) reads were later screened by identifying the coexistence of 5ʹ-primers, 3ʹ-primers and poly-A tails. We detected 184,584 FLNC reads (81.96% of CCS) with an average length of 2693 bp (Fig. 1b, c). In addition, six total RNA samples from tubers and leaves were sequenced using second-generation sequencing (SGS) on the Illumina platform, yielding 86,394 proofread consensus sequences ranging from 188 to 13,219 bp with a mean length of 2748 bp. After clustering the redundant and 95% similar transcript sequences, 49,585 unigenes were identified, among which 47,969 unigenes (96.74%) were over 1000 bp, 36,397 unigenes (73.40%) were over 2000 bp and 17,815 unigenes (35.93%) were over 3000 bp in length (Fig. 1d). To analyze the functions of the unigenes obtained from the full-length transcripts, we annotated the unigenes using the data from seven nucleotide and protein databases (NR, NT, Pfam, Swiss-Prot, KOG, KEGG, and GO) (Fig. S1). Out of 49,585 unigenes, 47,352 (95.50%) were annotated in at least one database, a much higher percentage than was obtained for the previous Illumina sequencing annotation result (65.56%). In addition, using full-length transcript data, the gene structures and transcription factor binding sites were also predicted (Fig. S2). KEGG analysis found 530 unigenes related to the biosynthesis of other secondary metabolites (Table S1), but there remains a question that until now, benzylisoquinoline alkaloids were the only alkaloid class reported in *C. yanhusuo*^13,26,32,33^. This seemingly conflicts with the KEGG annotation result that 69 unigenes were annotated to the tropane/piperidine/pyridine alkaloid metabolism pathway. However, upon further inspection, we found that nearly half of these unigenes were primary amine oxidases, and one-third were aspartate aminotransferases, such as GOTs and ASP5, which have a broad range of substrate specificities and participate in various metabolic pathways. The remaining ten unigenes were annotated to tropinone reductase I in the tropane alkaloid pathway, and seven had a high BLAST identity to this enzyme. Considering that no other enzymes in this pathway were annotated, nor were any tropinone alkaloids reported in *C. yanhusuo*, the function of these predicted proteins needs to be carefully evaluated.

**Table 1:** Statistics of *C. yanhusuo* SMRT sequencing and previously reported Illumina results

	SGS^26^	SMRT
Total clean raw reads	25,013,630	5,228,902
Total nucleotides (bp)	2,251,226,700	236,495,561
GC percentage	44.65%	41.50%
Average read-length (bp)	90	2341
Total contigs	158,101	N.A.^a^
Length range (bp)	60–5,752	N.A.^a^
Mean contig length (bp)	198	N.A.^a^
Total scaffolds	65,839	86,394
Length range (bp)	100–7,977	188–13,219
Mean scaffold length (bp)	340	12,991
Total Unigenes	47,081	49,585
Total nucleotides (bp)	23,016,560	141,504,321
Length range (bp)	150–7,973	188–13,219
Mean unigene length (bp)	489	2853
N50 (bp)	681	3124
No. (%) of annotated unigenes (*e* < 10^−5^)	30,868 (65.56%)	47,352 (95.50%)
NR	30,660 (65.12%)	46,586 (93.95%)
Swiss-Prot	20,664 (43.89%)	40,861 (82.41%)
KEGG	13,111 (27.85%)	45,821 (92.41%)
COG	9,412 (19.99%)	30,808 (62.13%)
GO	12,455 (26.45%)	31,242 (63.01%)

^a^SMRT sequencing produces full-length transcript reads, therefore contig assembly process is not applicable for SMRT sequencing

### Multivariate analysis of Q-TOF-MS data—global metabolomics

**Fig. 2:**
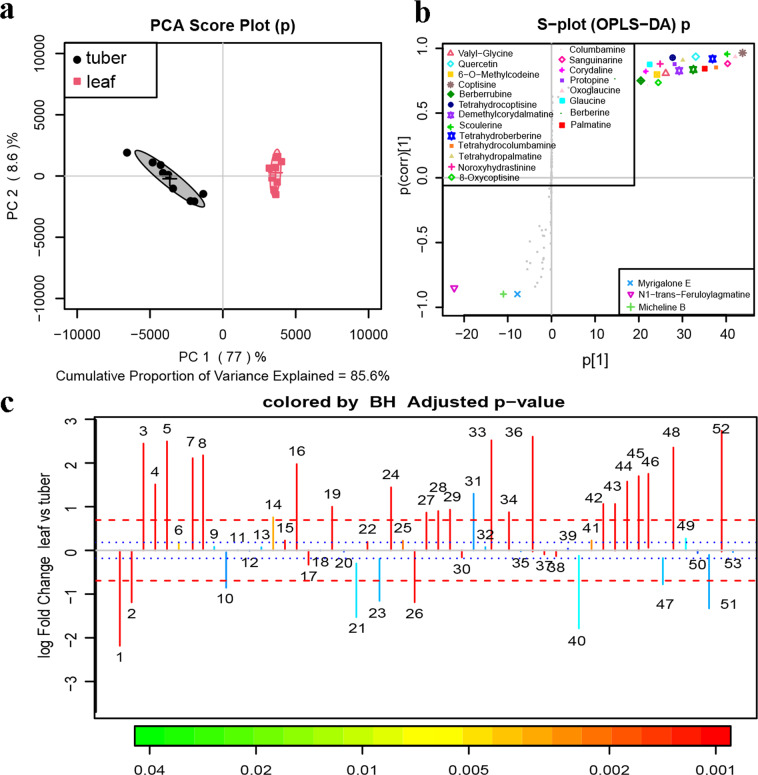
Metabolomic multivariate analysis of QTOF-MS data of *C. yanhusuo* extracts for the tuber and leaf groups. **a** PCA score plots, **b** color-coded S-plots, and **c** fold-change plots in positive ion mode (*n* = 9). In the PCA score plots, the tuber and leaf groups were satisfactorily separated and clearly clustered into two groups. Fold-change plots color-coded according to the corresponding *p*-values adjusted by the Benjamini–Hochberg method indicate the significance of the altered metabolites in *C. yanhusuo* extracts. The blue dotted lines and red dashed lines represent an increase or decrease of 20% and 100%, respectively

To further evaluate the candidate genes, untargeted ultra-performance liquid chromatography quadrupole-time-of-flight mass spectrometry (UPLC-Q-TOF-MS) profiling was conducted to generate mass lists corresponding to a wide variety of metabolites, including alkaloids. Data processing was conducted with Progenesis QI software (Waters, Milford, MA, USA). Compound identification was based not only on accurate mass but also on matching of the MS/MS fragmentation patterns (Fig. S3). Nineteen of these compound annotations were further validated by comparison with authentic standards (Fig. S4). Tuber and leaf groups were analyzed by principal component analysis (PCA) and the orthogonal partial least-squares-discriminant analysis (OPLS-DA) model. In the score plots, the tuber and leaf groups were separated and clustered into two groups (Fig. 2a). The S-plots (Fig. 2b) and color-coded loading plots (Fig. 2c) for OPLS-DA revealed the variation in metabolites in the tuber and leaf compared with the leaf group in the *C. yanhusuo* extracts. The tuber and leaf groups showed clear separation in the OPLS-DA score plots of *C. yanhusuo* extracts with satisfactory goodness of fit and statistical significance. The S-plots and fold-change plots revealed the variation in metabolites in the tuber and leaf groups, including increased levels of scoulerine, tetrahydroberberine, tetrahydrocolumbamine, columbamine, tetrahydropalmatine, sanguinarine, corydaline, protopine, tetrahydrocoptisine, noroxyhydrastinine, dehydrocorydaline, oxoglaucine, 8-oxycoptisine, palmatine, coptisine, berberine, jatrorrhizine, epiberberine, and glaucine. The details of these metabolites are presented in the supporting information (Fig. S4, Fig. S5, and Fig. S6). Thirty-eight of the 53 annotated metabolites were found to be significantly different between leaves and tubers (Table 2). According to the spatial difference in BIA compound accumulation between the leaf and tuber, the total transcripts in each organ are useful sources to perform differential gene expression analysis, which helps in further identification of unigenes involved in BIA biosynthesis.

**Table 2:**
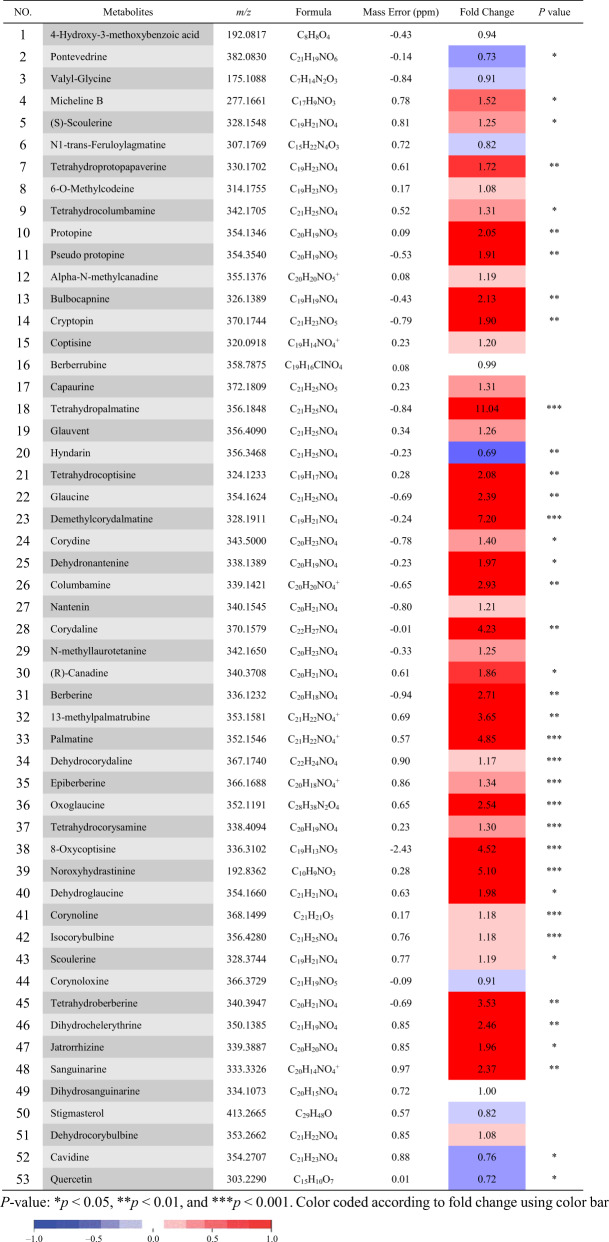
Identified metabolites with fold changes between tuber and leaf groups and their *p*-values in *C. yanhusuo*

### Differential expression of genes between *C. yanhusuo* tubers and leaves

**Fig. 3:**
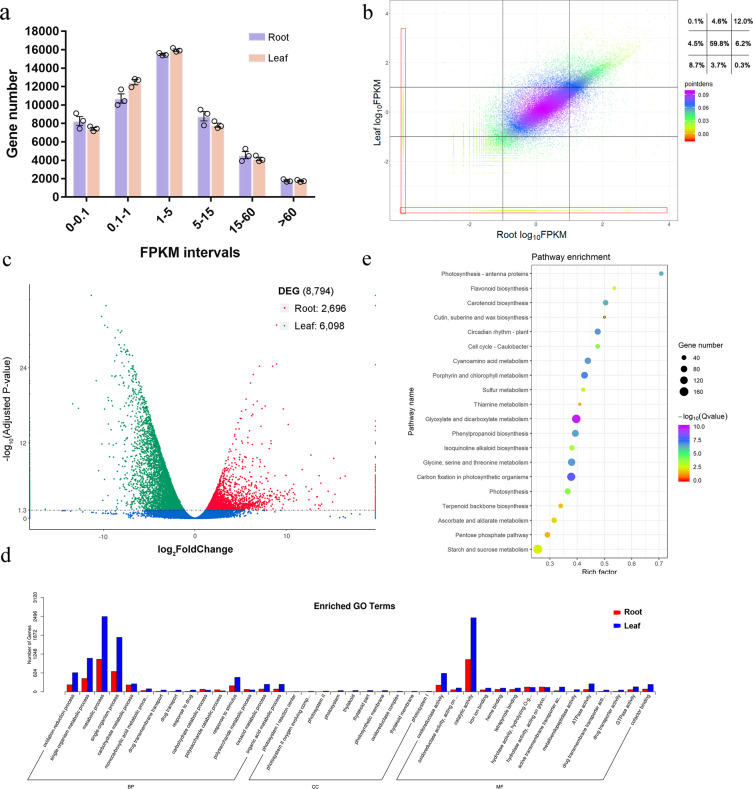
Differential expression of genes between *C. yanhusuo* tubers and leaves. Leaves and tubers were sampled for RNA-seq on the Illumina platform, and their abundance was evaluated by converting read count to FPKM. The gene expression level distribution of six samples is elaborated. **a** Distribution of the number of genes within certain FPKM intervals. **b** Density scatter plot provides an overview of different expression patterns for each gene in different organs. The ratio number in each well indicates the proportion of unigenes in the corresponding frame. Points within the red boxes have FPKM values of 0 in either leaf or tuber. **c** Volcano plot showing the distribution of differentially expressed genes (DEGs) with a *p*-value threshold of 0.05. **d** GO term enrichment results for genes from the tuber and leaf. **e** Pathway enrichment analysis presented in the form of a bubble plot

To investigate the abundance of organ-specific transcripts, leaves and tubers were sampled for RNA-seq on the Illumina platform, and their abundance was evaluated by converting read counts to fragments per kilobase per million (FPKM). The distribution of gene expression levels for six samples is plotted in Fig. 3a. Figure 3b shows the overall expression pattern of each unigene and their relative ratio for each FPKM interval in roots and leaves. The differentially expressed genes (DEGs) were filtered with a threshold of adjusted *p*-values < 0.05 and |log_2_^FoldChange^ | > 0, in which fold-change was defined as the read count in tubers divided by that in leaves. A total of 8794 unigenes (17.74%) were identified as DEGs (Table S2). A volcano plot was used to show the relationship between the adjusted *p*-value and log_2_^FoldChange^ (Fig. 3c). GO term enrichment and pathway enrichment analysis presented in the form of a bubble plot provided certain information (Fig. 3d, e). The most enriched pathway was that of the photosynthesis-antenna proteins, followed by flavonoid biosynthesis and carotenoid biosynthesis, both of which are important secondary metabolites as plant pigments. As we predicted, isoquinoline alkaloid biosynthesis had an enrichment factor of 0.38 with a *Q*-value of 6.02 × 10^−4^, revealing that isoquinoline alkaloid biosynthesis genes are mainly enriched in tubers compared with leaves.

### Identifying candidate genes in the benzylisoquinoline biosynthetic pathway

KEGG analysis found that 530 unigenes were associated with secondary metabolite synthesis (Table S1). One-hundred thirty-four unigenes were annotated to participate in isoquinoline alkaloid biosynthesis, which is the most abundant alkaloid synthesis pathway. Additionally, Pfam annotation found 328 unigenes containing the cytochrome P450 conserved domain, which are known to play indispensable roles in BIA biosynthesis. As most of the reported secondary metabolites in *C. yanhusuo* are BIAs, here, to identify the candidate genes involved in the BIA biosynthetic pathway, we BLAST searched the nonredundant full-length sequence we retrieved from *C. yanhusuo* against the reported BIA biosynthetic enzyme homologs. The protein homologs mainly belong to species in the Papaveraceae family, including *Papaver somniferum*, *Macleaya cordata*, and *Eschscholzia californica*. Sequences from *Coptis japonica* and *Berberis stolonifera* were also used because certain enzymes (for example, columbamine O-methyltransferase and berbamunine synthase) are found exclusively in these plants^34^.

**Fig. 4:**
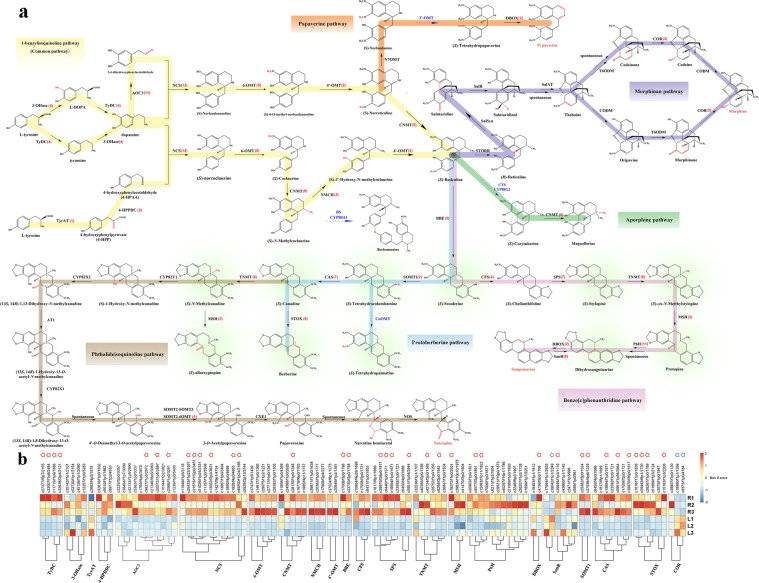
A proposed benzylisoquinoline biosynthetic pathway. **a** A total of 101 nonredundant unigenes are involved in the BIA biosynthetic pathway according to the tblastn results. Highlighted compounds are those previously reported in *C. yanhusuo*. Red numbers after each enzyme are the number of unigenes blasted to their homologs with an identity over 60%. Enzymes catalyzing reactions with blue arrows have not been identified in the Papaveraceae family. **b** Expression profile of all BIA biosynthesis candidate genes. Three biological replicates from the leaf (L) and tuber (R) were plotted individually. Unigenes labeled by red asterisks are DEGs predominant in tubers, while those marked by blue asterisks are predominant in leaves. TyDC tyrosine/dopa decarboxylase, 3-OHase tyrosine 3-hydroxylase, TyrAT tyrosine aminotransferase, 4-HPPDC 4-hydroxyphenylpyruvate decarboxylase, NCS norcoclaurine synthase, 6OMT (S)-norcoclaurine 6-O-methyltransferase, CNMT (S)-coclaurine N-methyltransferase, NMCH (S)-N-methylcoclaurine 3ʹ-hydroxylase; 4ʹOMT (S)-3ʹ-hydroxy-N-methylcoclaurine 4ʹ-O-methyltransferase, N7OMT norreticuline 7-O-methyltransferase, BBE berberine bridge enzyme; SOMT scoulerine 9-O-methyltransferase, CAS canadine synthase, STOX (S)-tetrahydroxyprotoberberine oxidase, CoOMT Columbamine O-methyltransferase, CFS cheilanthifoline synthase, SPS stylopine synthase, TNMT tetrahydroprotoberberine N-methyltransferase, MSH methylstylopine hydroxylase, P6H protopine 6-hydroxylase, DBOX dihydrobenzophenanthridine oxidase, STORR 1,2-dehydroreticuline synthase/reductase, SalSyn salutaridine synthase, SalR salutaridine reductase, SalAT salutaridinol 7-O-acetyltransferase, T6ODM thebaine 6-O-demethylase, COR codeinone reductase, CODM codeine O-demethylase, AT1 (13S,14R)-1,13-dihydroxy-N-methylcandine 13-O-acetyltransferase, CXE1 3-O-acetylpapaveroxine carboxylesterase, NOS noscapine synthase

We found a total of 101 nonredundant unigenes related to the enzymes in the BIA biosynthetic pathway according to the tblastn results (Fig. 4 and Table S3). All the enzymes in the common pathway (pathway in yellow in Fig. 4a or Fig. S7) succeeded in finding their corresponding unigenes, which proves that *C. yanhusuo*, like other species, uses a common pathway to provide the fundamental precursor (S)-reticuline for synthesizing downstream BIAs. Every enzyme in this pathway is indispensable. However, we did not find many hits in the morphinan pathway (purple) or the phthalideisoquinoline pathway (brown) (Fig. 4a), and none of the corresponding compounds has been reported in *C. yanhusuo*. (S)-Allocryptopine is synthesized at the beginning of the phthalideisoquinoline pathway, but it is a structural analog of protopine^35^. All the enzymes in the protoberberine pathway (blue) and the benzo[c]phenanthridine pathway (pink) can find their hits in the transcriptome, verifying that protoberberines and protopines are the representative chemical components in *C. yanhusuo*. We successfully found adequate unigenes required for synthesizing all the BIA compounds in *C. yanhusuo* according to current phytochemistry reports^11,14,36^.

To investigate the expression pattern of the selected candidate genes, a heatmap for all the candidate genes was plotted for each leaf (L1-3) and tuber (R1-3) sample (Fig. 4b or Fig. S8). The heatmap reveals that the expression of BIA biosynthetic genes is well regulated in an organ-specific manner. Among them, 36 unigenes were previously identified as DEGs (Fig. 4b, asterisks after unigene IDs, three of which reoccurred in SPS and CAS), indicating that most of them showed a higher level of expression in tubers (red asterisks). Most of the DEGs are involved in the benzo[c]phenanthridine pathway in BIA biosynthesis, which indicates that the upregulation of protopine- and protoberberine-type alkaloid biosynthetic genes in the tuber is a synchronized process. Meanwhile, typical enzymes such as 3-OHase, TyrAT, DBOX, and COR showed a vague regulation pattern between tubers and leaves; therefore, the reactions catalyzed by these enzymes may not be strictly regulated. Some variances among tuber samples (Fig. 4b, especially R2) may indicate that certain enzymes have their own sub-organ distribution.

Collectively, the expression profiling vindicates the hypothesis that the different expression levels of BIA biosynthetic genes between the two organs may the reason that tubers, not leaves, are used as the therapeutic agent in *C. yanhusuo*. These findings also suggest the possibility of a transcriptional regulation mechanism of BIA biosynthesis in *C. yanhusuo*.

### Analyses of 19 marker compounds in synthesized BIAs-targeted metabolomics

A global metabolomics study revealed that a few alkaloids dominate among the synthesized BIAs. To further justify the reliability of the differentially expressed transcriptome information in Fig. 4b, we chose nineteen representative BIAs synthesized in *C. yanhusuo*, namely, scoulerine, canadine (also known as tetrahydroberberine), tetrahydrocolumbamine, columbamine, tetrahydropalmatine, sanguinarine, corydaline, protopine, tetrahydrocoptisine, noroxyhydrastinine, dehydrocorydaline oxoglaucine, 8-oxycoptisine, palmatine, coptisine, berberine, jatrorrhizine, epiberberine, and glaucine, as marker compounds to investigate their abundance in tubers and leaves. The total ion chromatograms (TICs) of the standard samples were obtained and are displayed in Fig. S4. The metabolite abundance of these compounds between the two groups was quantified through a subsequent targeted metabolomics analysis that was performed to identify candidate metabolites involved in BIA biosynthesis in *C. yanhusuo*. Differences could be observed in the contents of nineteen compounds in tubers and leaves (Fig. S9), which corresponded with the previous transcriptome analysis to some extent (Fig. 4b). This observation also corroborates the hypothesis that the accumulation of BIAs in tubers is related to the BIA biosynthetic gene expression pattern according to our previous analysis.

### Sequence and phylogenetic analysis of O-methyltransferase candidates

**Fig. 5:**
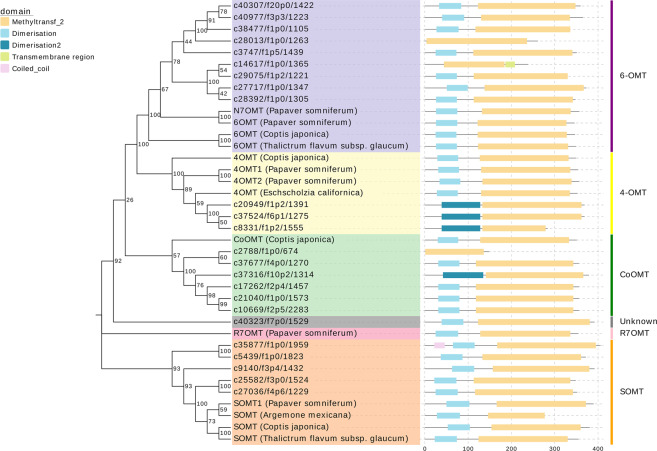
Phylogenetic analysis was performed to investigate the OMT protein family classification in *C. yanhusuo*. The phylogenetic tree shows that all OMT candidates formed four major clades: 6-OMT, 4ʹ-OMT, CoOMT, and SOMT

Phylogenetic analysis was conducted to identify the corresponding O-methyltransferases (OMTs) because most OMT tblastn results shared high similarity, and some of the unigenes matched to several OMTs simultaneously. Another reason for our interest in OMTs is that one O-methylated BIA end-product, (S)-tetrahydropalmatine (THP), has great medicinal value as an analgesic or sedative without addictive side effects and can ameliorate opiate addiction^3,37^; therefore, efforts to identify OMTs that are able to catalyze such a reaction from (S)-tetrahydrocolumbamine to THP (CoOMT) have been ongoing for years. Here, we performed phylogenetic analysis to provide some insights into OMT protein family classification in *C. yanhusuo* (Fig. 5). The dendrogram from the phylogenetic analysis showed that all the OMT candidates clustered into 4 major clades: 6-OMT, 4-OMT, CoOMT, and SOMT. R7OMT is separated from all the presenting clades, which reveals the lack of homologous enzymes in *C. yanhusuo*. The analysis of the domain architectures conducted on SMART^38^ reveals that most of the predicted polypeptide chains and all the OMTs from other plant species contain two domains: the C-terminal methyltransferase domain (in orange) is typical of SAM-dependent O-methyltransferases, and the N-terminal domain (in light or dark blue) is a dimerization domain that is found in a variety of plant OMTs. The dimerization domain not only stabilizes the homodimer through hydrophobic interactions but also provides an extra water-mediated hydrogen bond with the substrate to improve specificity^39^. However, some candidates do not possess a dimerization domain; thus, their ability to catalyze corresponding reactions is questionable.

Phylogenetic analysis found nine unigenes mapped closely to the 6-OMT clade, and most had a high tblastn score to Ps6-OMT. Three unigenes were classified as 4-OMTs. Six and five unigenes were mapped to the CoOMT and SOMT clades, respectively, which might help explain the accumulation of THP in *C. yanhusuo*, as they were both reported to catalyze the reaction from tetrahydrocolumbamine to THP^40,41^. It is possible that enzymes with CoOMT activity could appear from the translational products of these 10 unigenes in the CoOMT and SOMT clades (one with insufficient length was excluded). All the sequence information used in the phylogenetic analysis is provided in Table S4.

## Discussion

The identification of BIA biosynthetic candidate genes from *C. yanhusuo* is a pivotal step in the investigation of the pharmaceutical value of *C. yanhusuo* and may provide additional information to guide BIA-related pathway elucidation (e.g., corydaline, dehydrocorybulbine). In this study, we generated the first full-length transcriptome for *C. yanhusuo* and investigated the transcripts related to BIA biosynthesis in both leaf and tuber tissue of *C. yanhusuo* by combining SMRT and SGS technologies. A previous study focused on elucidating whether BIA biosynthetic candidate genes were transcriptionally regulated over the time-course of bulb development based on SGS short-read data^26^. Although the SGS results provided a general view of the BIA biosynthetic pathway in the *C. yanhusuo* bulb, full-length transcripts and transcriptome data from other tissues had not yet been obtained, which greatly impeded the molecular cloning and gene verification process.

In line with traditional Chinese medicine usage of *C. yanhusuo* tubers, the bioinformatic analysis revealed that isoquinoline alkaloid biosynthesis genes were mainly enriched in tubers rather than leaves. We then crudely found 101 unigenes related to BIA biosynthesis according to similarity with enzyme orthologs from other species. The presence of such genes in *C. yanhusuo*, as well as their relevance to alkaloid biosynthesis, was validated by conducting quantitative reverse transcription pCR (qRT-PCR) analysis between the control and methyl jasmonate (MeJA) treatment groups (Fig. S10). External application of MeJA is known to dramatically promote the accumulation of alkaloids and the expression of key genes^42–46^. In particular, MeJA was also reported to trigger in vitro accumulation of dihydrosanguinarine, a product in the BIA pathway, and the expression of key biosynthetic genes in *Eschscholzia californica* cell culture^42,47^. Thus, we expected that the external application of MeJA would increase the expression of BIA biosynthetic genes, and indeed, the fold changes shown in Fig. S10 indicate the relevance of these genes to alkaloid bioproduction. To clarify the distribution of BIAs in different tissues, 19 BIAs were evaluated in tubers and leaves and observed to be enriched in tubers. Thus, we could infer that the spatial distribution of such genes is likely responsible for the difference in BIA contents in tubers and leaves. However, such inference is based on the following presumptions: (i) no other genes are responsible for BIA biosynthesis, (ii) BIAs are locally synthesized in the tubers instead of being transported there, and (iii) BIAs do not undergo automatic degradation in the leaf tissue. To address points (i) and (iii), we used tblastn to crudely filter the BIA biosynthetic genes, which circumvented potential hit escape. Using the multidrug-resistant protein CjABCB1-3, which is involved in berberine translocation^48,49^, as a query, a total of 21 full-length hits whose products may function in unloading BIAs from tubers and promote their transport to aerial parts of the plant were found. In addition, as considerable amounts of compounds can still be detected in the leaves (Table 2 and Fig. S9), given that the biosynthesis of BIAs in leaves is not as extensive as that in tubers, we believe that major BIAs in *C. yanhusuo* do not undergo significant degradation. This interpretation is in line with the biological role of alkaloids, that is, to protect plants against insects or herbivores. There is no way for organisms to evolve a feature (here, alkaloid degradation) that is potentially detrimental to them. Related to presumption (ii), a report has already monitored BIA accumulation in tubers during development and concluded that the biosynthesis of THP showed a latent accumulation pattern, which might be the result of biosynthesis using nutrients transported from the aerial to the terrestrial parts^50^. This study ruled out the possibility that BIAs are synthesized elsewhere and then transported to the tuber. Considering that the leaf is the primary place where organic building blocks are generated through photosynthesis, a higher content of BIAs detected in tubers supports the assignment of the biosynthetic role to tubers but not leaves. Hence, it is quite convincing that the spatial difference in BIA contents is caused by BIA-related DEGs.

With respect to the identified unigene candidates (Fig. 5), 6 out of 12 NCS genes and all 4 TyDC genes were denoted as DEGs, indicating that the entrance of the 1-benzylisoquinoline pathway is a pivotal point for the regulation of BIA biosynthesis. Although enzymes involved in pathways other than protopine and protoberberine pathways were found (e.g., COR in the morphinan pathway), they showed a variable expression pattern between tuber and leaf samples. Two mapped to nonfunctional NADPH-dependent COR genes from lotus and poppy. Another mapped to a zinc finger protein that widely exists in the Papaveraceae family, and the reason for its annotation as a BIA pathway in KEGG is unclear. This could be due to faulty annotation or functional interpretation of the blast result, or this gene could be a genetic remnant of evolution when diverging from other morphine-producing plants. All the OMT candidate sequence sources were used in the phylogenetic analysis, but one unigene could not be identified (in gray, labeled unigene 1529), as it did not belong to any clade. According to the annotation information, unigene 1529 resembles the gene coding trans-resveratrol di-O-methyltransferase (ROMT) from *Vitis vinifera*, an enzyme that converts resveratrol to pterostilbene in stilbenoid biosynthesis^51^. We queried VvROMT via tblastn and found that it has 45% identity to unigene 1529 and less than 40% to other unigenes; therefore, we inferred that unigene 1529 might be an ortholog of the VvROMT gene since early basal groups of eudicots diverged in Rosidae^52^. Back to the discussion of papaverine biosynthesis, no 3ʹ-O-methyltransferase candidates were found in any OMT gene, according to our phylogenetic analysis, which could be why no papaverine has ever been detected in *C. yanhusuo*.

A previous study identified a series of genes that mapped to the morphinan pathway^26^. However, our method identified very few enzymes in this pathway except for COR. The query sequences we used were all from *P. somniferum*, in the same family (Table S3), from which high identity hits (60%) should have been found if they existed. Such results indicate the lack of morphinan pathway-related enzymes, which corresponds with the chemical profile in *C. yanhusuo*^11,14,36^.

An interesting phenomenon is that although many aporphine compounds have been reported in *C. yanhusuo*^14^, we could not find the key enzyme (corytuberine synthase/CYP80G2)^53^ at the beginning of the aporphine pathway (green) using the threshold we set. A similar phenomenon can also be observed on CoOMT^41^, the product of which, (S)-tetrahydropalmatine (THP), abundantly exists in the tubers. Considering that the query plant we used (*Coptis japonica*) is not in the Papaveraceae family, there might be great variance in protein sequences. Therefore, when we adjusted the tblastn threshold to 40%, we found two unigene hits for CjCYP80G2 (51 and 48%) and two unigene hits for CjCoOMT (both 40%) (not shown in Fig. 4). Additionally, it was reported that SOMT1 in *P. somniferum* could catalyze the conversion from tetrahydrocolumbamine to THP^40^, for which we obtained two hits after filtering unigenes successfully blasted to other OMTs with higher scores. We found five unigenes in the same clade with CjCoOMT and PsSOMT1, excluding a truncated unigene (c2788/f1p0/674). Further study could focus on cloning these unigenes to validate their activity at the protein level.

In summary, we reported the first full-length transcriptomes of the herbal medicinal plant *Corydalis yanhusuo* W.T. Wang. We also used the retrieved data to localize the metabolism of benzylisoquinoline alkaloids to the tuber and carried out organ-differentiated transcriptome analysis using a combined SMRT and SGS approach. This enabled us to identify the unigenes related to the BIA biosynthetic pathway. The expression patterns of the genes were validated through qRT-PCR, and UPLC-MS/MS was used to detect the metabolite products of the reactions catalyzed by the putative enzymes we found. Moreover, phylogenetic analysis identified ten candidate genes for THP synthesis; further studies of these genes are needed to verify their functions. Finally, our study provides an approach with which to analyze full-length transcriptome data and the biosynthesis process in other medicinal plants.

## Material and methods

### Plant materials and sample preparation

One-year-old *Corydalis yanhusuo* material was collected from the agriculture base in Pan’an City, Zhejiang Province, China (120.450 °E, 29.054 °N), which is the genuine production area of *C. yanhusuo*. The *C. yanhusuo* plants were then transplanted to pots in the laboratory containing a mixture of perlite, vermiculite, and peat moss at a ratio of 1:1:1 and cultured for 7 days at a temperature of 20 °C with a day length of 12 h. The relative humidity was between 40 and 70%. The plants were harvested in mid-April, and fresh tissues were sampled. After removing the soil and dead wood, the tissues were immediately frozen in liquid nitrogen to prevent RNA degradation. All experimental groups contained three biological replicates from tubers and leaves for sequencing.

### RNA extraction, quality assessment, and quantification

Total RNA was extracted using the RNAsimple Total RNA Kit (Tiangen Biotech, Beijing, China) according to the manufacturer’s protocol. The quality of the total RNA was determined by 1% agarose gel electrophoresis, and the OD_260/280_ values were checked with a NanoDrop ND-1000 spectrophotometer (NanoDrop Technologies, Wilmington, DE, USA). The total RNA samples with good quality were preserved for later Iso-Seq library construction.

### Iso-Seq library construction and single-molecular real-time (SMRT) sequencing

After enriching mRNA from total RNA by oligo-dT magnetic beads, the cDNA library was reverse transcribed using the Clontech SMARTer PCR cDNA Synthesis Kit (Takara Bio, Mountain View, CA, USA) and Phusion High-fidelity DNA polymerase (NEB, Ipswich, MA, USA) using PCR amplification to yield a large quantity of cDNA. Size selection (>4 kb) to obtain full-length transcripts was conducted following the BluePippin Size Selection System protocol. Then, the repair (including end repair and internal repair) and exonuclease treatment of the full-length cDNA was performed. The Pacific Biosciences DNA Template Prep Kit 2.0 was used to construct the SMRT bell iso-seq libraries. The overall process was performed according to the isoform sequencing protocol (Iso-Seq) and by Pacific Biosciences (PN 100-092-800-03). After construction of the Iso-seq library, SMRT sequencing was performed on a Pacific Bioscience PacBio Sequel platform (Novogene, Beijing, China).

### Illumina cDNA library construction and second-generation sequencing (SGS)

The cDNA library for SGS was constructed using the NEBNext® Ultra™ RNA Library Prep Kit for Illumina® (NEB, Beverly, MA, USA) according to the protocol provided by the manufacturer. The cDNA library sample was sent for sequencing on the high-throughput Illumina HiSeq platform (Novogene, Beijing, China).

The quality control of the SGS raw results was implemented through in-house Perl scripts: the adapter and primer sequences and those reads with poly-N were completely removed from the sequenced raw reads, and low-quality data (>50% bases with *Q*-value ≤ 20) were also filtered by calculating the Q20 and Q30 scores and sequence duplication level. The clean data yielded by Illumina were further used for correction of the PacBio sequencing data.

### Gene functional annotation

Gene function was annotated based on the following databases: NR (NCBI nonredundant protein sequences)^54^; NT (NCBI nonredundant nucleotide sequences); Pfam (Protein family)^55^; KOG/COG (Clusters of Orthologous Groups of proteins)^56^; Swiss-Prot (a manually annotated and reviewed protein sequence database)^57^; KO (KEGG Ortholog database)^58^; and GO (Gene Ontology)^59^. BLAST software was used for NT database analysis with the *E*-value set at 1e^−10^. The software Diamond BLASTX V0.8.36 (https://github.com/bbuchfink/diamond) was used for NR, KOG, Swiss-Prot, and KEGG database analysis with the *E*-value set at 1e^−10^. The software Hmmscan V3.1b2 (http://hmmer.org/) was used for Pfam database analysis.

### Gene expression levels and differential expression analysis

The input data for differential expression analysis is the read count of each gene retrieved by bowtie2 in RSEM software (V1.3.0)^60^. The differential expression analysis steps were as follows: The read count was first normalized using the DESeq *R* package (1.10.1), and then a hypothesis test on the negative binominal distribution model was conducted to calculate the *p*-values. Finally, the *p*-values were adjusted using Benjamini and Hochberg’s multiple testing approach to control the false discovery rate^61^. Genes with adjusted *p*-values < 0.05 were assigned as differentially expressed genes (DEGs).

### GO and KEGG enrichment analysis

GO (Gene Ontology) term enrichment analysis of DEGs was performed via the GOseq *R* package^62^, which is based on the Wallenius noncentral hypergeometric distribution in which the gene selection bias was corrected by gene length. GO terms with corrected *p*-values < 0.05 were considered significantly enriched among DEGs. KEGG (Kyoto Encyclopedia of Genes and Genomes) pathway enrichment analysis was then performed using KOBAS (2.0) software, which carries out hypergeometric testing to identify the DEG-enriched KEGG pathways with a false discovery rate < 0.05.

### Identification of candidates in the BIAs pathway

Local tblastn 2.2.10 was used to identify BIA pathway-related genes. Query sequences were retrieved from the GenBank and SwissProt databases. Tblastn was performed against *C. yanhusuo* FLNC fasta sequences. The results were filtered by identity >60% to allow for sequencing error. The heatmap was generated using the pheatmap *R* package with row scaling.

### UPLC-Q-TOF-MS analysis for the nontargeted metabolomics study

For the metabolomics experiments, nine samples from each group were used for separate analyses. Analyses were performed using a Waters ACQUITY UPLC system coupled with SYNAPT G2-Si HDMS Q-TOF-MS. Samples were analyzed using an ACQUITY UPLC BEH column (2.1 × 100 mm, 1.7 μm, Waters Corporation, Milford, MA, USA) in positive mode. The column temperature was maintained at 40 °C, and the flow rate of the mobile phase was 0.30 mL/min, with an injection volume of 1.0 µL. Mobile phase A was 0.1% (v/v) formic acid/water, while mobile phase B was 0.1% (v/v) formic acid/acetonitrile. The automatic sampler was set at 4 °C during the analysis of all samples.

To acquire mass spectrometry data, a SYNAPT G2-Si HDMS with an electrospray ionization source was used. All data were collected in MS^E^ mode, and the parameters were as follows: MS^E^ range 50–1000 *m/z*; MS^E^ high energy 20 to 50 eV. Leucine enkephalin was continuously acquired and used to correct data (*m/z* 556.2771 in positive mode).

All data were acquired in MassLynx V4.2 and imported into Progenesis QI V2.0 (Waters Corporation, Milford, MA, USA) for background noise elimination, normalization to a reference sample, retention time correction, peak selection, and identification of compounds with reference to databases such as METLIN, HMDB, and Lipid Maps. Structural confirmation was conducted by comparison with the reference standards (*t*_R_ and MS data) or matching with theoretical data or commercial libraries. After Pareto scaling, principal component analysis (PCA) and orthogonal partial least-squares-discriminant analysis (OPLS-DA) were performed. The significantly different metabolites were identified based on the combination of statistically significant values for variable importance in projection (VIP) obtained from the OPLS-DA model and two-tailed Student’s *t*-test (*p*-value) applied to the raw data, and metabolites with VIP values larger than 1.0 and *p*-values < 0.05 were considered significantly different. Global metabolomics was performed as described in the Supporting Information.

### Analysis of alkaloid by targeted metabolomics

Considering that isoquinoline alkaloids are the main component of *C. yanhusuo*, 19 alkaloids (including scoulerine, canadine, tetrahydrocolumbamine, columbamine, tetrahydropalmatine, sanguinarine, corydaline, protopine, tetrahydrocoptisine, noroxyhydrastinine, dehydrocorydaline, oxoglaucine, 8-oxycoptisine, palmatine, coptisine, berberine, jatrorrhizine, epiberberine, and glaucine) in tubers and leaves were detected by targeted metabolomics profiling by electrospray ionization (ESI) tandem mass spectrometry (MS/MS). The profiling was performed with a Waters ACQUITY UPLC H-Class system consisting of a quaternary pump with degasser and autosampler in combination with a Waters Xevo TQ-XS mass spectrometer. Targeted metabolomics was performed as described in the Supporting Information.

### qRT-PCR analysis

RNase-free DNase I (Takara Biotechnology, Dalian, China) was used to pretreat RNA samples before their use in reverse transcription to minimize DNA contamination. Following the instructions accompanying the HiScript Q RT SuperMix for qPCR kit (Vazyme, China), 1 μg of template RNA for cDNA analysis was added to a 20 mL admixture and diluted ten times for qRT-PCR analysis. The primer pairs used are shown in Table S5. The relative expression levels of the target genes were calculated using the 2^−ΔΔCT^ approach with *GAPDH* as the reference gene^63^. Each gene was analyzed in three biological replicates and three technical replicates.

### Phylogenetic analysis

The OMT candidates were selected from all genes with tblast hits to 4ʹ-OMT, 6-OMT, CoOMT, SOMT1, N7OMT, and R7OMT, regardless of their scores. The protein sequences of these candidates were retrieved from the CDS prediction fasta files. Sequences from other species were acquired from the UniProt Knowledgebase. The alignment before phylogenetic analysis was achieved by MUSCLE with default parameters. MEGA-X was used to construct a neighbor-joining tree using the bootstrap method (1000 replications) with the Poisson model and pairwise deletion^64^. Tree visualization was achieved using the EvolView webpage^65^.

## Supplementary information

Primer pairs used in qRT-PCR verification

Integration of full-length transcriptome and targeted metabolomics for identifying Benzylisoquinoline Alkaloid Biosynthetic genes in Corydalis yanhusuo

Figure S1

Figure S2

Figure S3

Figure S4

Figure S5

Figure S6

Figure S7

Figure S8

Figure S9

Figure S10

Identifying candidate genes in Benzylisoquinoline biosynthetic pathway

Filter result of 8,794 differential expressed genes

BIA synthetic pathway-related unigenes and putative enzymes

Sequence information of all OMT candidates and reference sequences

## Data Availability

The PacBio SMRT reads and the Illumina SGS reads generated in this study have been submitted to the BioProject database of the National Center for Biotechnology Information (accession number PRJNA539894). Annotation results and sequences are available at 10.6084/m9.figshare.12800222.v1.

## References

[1] Wang L (2016). The antinociceptive properties of the Corydalis yanhusuo extract. PLoS ONE.

[2] Xu Z (2012). Dehydrocorydaline inhibits breast cancer cells proliferation by inducing apoptosis in MCF-7 cells. Am. J. Chin. Med.

[3] Wang JB, Mantsch JR (2012). l-tetrahydropalamatine: a potential new medication for the treatment of cocaine addiction. Future Med. Chem..

[4] Xu W (2013). L-isocorypalmine reduces behavioral sensitization and rewarding effects of cocaine in mice by acting on dopamine receptors. Drug Alcohol Depend..

[5] Wen C, Wu L, Ling H, Li L (2007). Salutary effects of Corydalis yanhusuo extract on cardiac hypertrophy due to pressure overload in rats. J. Pharm. Pharm..

[6] Wu L, Ling H, Li L, Jiang L, He M (2007). Beneficial effects of the extract from Corydalis yanhusuo in rats with heart failure following myocardial infarction. J. Pharm. Pharm..

[7] Han Y (2012). l-Tetrahydropalmatine, an active component of Corydalis yanhusuo W.T. Wang, protects against myocardial ischaemia-reperfusion injury in rats. PLoS ONE.

[8] Luo X (2019). Complete biosynthesis of cannabinoids and their unnatural analogues in yeast. Nature.

[9] Lin GM, Warden-Rothman R, Voigt CA (2019). Retrosynthetic design of metabolic pathways to chemicals not found in nature. . Curr. Opin. Syst. Biol..

[10] Chou T (1936). The alkaloids of the Chinese Corydalis ambigua Cham, et Sch.(yen-hu-so). VI. identification of corydalis D and corydalis M. Chin. J. Physiol..

[11] Yang X, Yang X, Liu J (2014). Study on material base of Corydalis Rhizoma. Zhongguo Zhongyao Zazhi.

[12] Sun M, Liu J, Lin C, Miao L, Lin L (2014). Alkaloid profiling of the traditional Chinese medicine Rhizoma corydalis using high performance liquid chromatography-tandem quadrupole time-of-flight mass spectrometry. Acta Pharm. Sin. B.

[13] Li Q (2017). Fingerprint-efficacy study of the quaternary alkaloids in Corydalis yanhusuo. J. Ethnopharmacol..

[14] Wu XS, Xu J, Zhang XM, Zhang TJ, Chen CQ (2015). Research progress on chemical constituents and pharmacological activities of Yuanhu Zhitong Prescription. Zhong Cao Yao.

[15] Cabedo N, Berenguer I, Figadère B, Cortes D (2009). An overview on benzylisoquinoline derivatives with dopaminergic and serotonergic activities. Curr. Med. Chem..

[16] Kumar A (2015). Current knowledge and pharmacological profile of berberine: an update. Eur. J. Pharm..

[17] Beaudoin GA, Facchini PJ (2014). Benzylisoquinoline alkaloid biosynthesis in opium poppy. Planta.

[18] Hagel JM, Facchini PJ (2013). Benzylisoquinoline alkaloid metabolism: a century of discovery and a brave new world. Plant Cell Physiol..

[19] Yang M (2017). Digital gene expression analysis provides insight into the transcript profile of the genes involved in aporphine alkaloid biosynthesis in Lotus (Nelumbo nucifera). Front. Plant Sci..

[20] Nishiyama Y (2010). Antinociceptive effects of the extracts of Xylopia parviflora bark and its alkaloidal components in experimental animals. J. Nat. Med..

[21] Zhao X (2003). The influence of dehydrocorydaline on intracellular free calcium concentration during hypoxia in myocardial cell of guinea-pigs. China Appl. Physiol..

[22] Hagel JM (2015). Transcriptome analysis of 20 taxonomically related benzylisoquinoline alkaloid-producing plants. BMC Plant Biol..

[23] Zhao Y (2019). Transcriptomic profiles of 33 opium poppy samples in different tissues, growth phases, and cultivars. Sci. Data.

[24] He SM (2018). Identification and characterization of genes involved in benzylisoquinoline alkaloid biosynthesis in species. Front. Plant Sci..

[25] Pourmazaheri H (2019). Comparative analysis of the root and leaf transcriptomes in Chelidonium majus L. PLoS ONE.

[26] Liao D (2016). Identification and developmental expression profiling of putative alkaloid biosynthetic genes in Corydalis yanhusuo bulbs. Sci. Rep..

[27] Morganti S (2019). Complexity of genome sequencing and reporting: next generation sequencing (NGS) technologies and implementation of precision medicine in real life. Crit. Rev. Oncol. Hemat..

[28] Au KF, Underwood JG, Lee L, Wong WH (2012). Improving PacBio long read accuracy by short read alignment. PLoS ONE.

[29] Au KF (2013). Characterization of the human ESC transcriptome by hybrid sequencing. Proc. Natl Acad. Sci. USA.

[30] Xu Z (2015). Full-length transcriptome sequences and splice variants obtained by a combination of sequencing platforms applied to different root tissues of Salvia miltiorrhiza and tanshinone biosynthesis. Plant J..

[31] Chao Q (2019). The developmental dynamics of the Populus stem transcriptome. Plant Biotechnol. J..

[32] Du W (2018). Development and validation of a HPLC-ESI-MS/MS method for simultaneous quantification of fourteen alkaloids in mouse plasma after oral administration of the extract of Corydalis yanhusuo tuber: application to pharmacokinetic study. Molecules.

[33] Zhang J (2009). Systematic screening and characterization of tertiary and quaternary alkaloids from Corydalis yanhusuo W.T. Wang using ultra-performance liquid chromatography-quadrupole-time-of-flight mass spectrometry. Talanta.

[34] Desgagné-PenixIsabel I (2010). Integration of deep transcriptome and proteome analyses reveals the components of alkaloid metabolism in opium poppy cell cultures. BMC Plant Biol..

[35] Fu XY, Liang WZ, Tu GS (1986). Chemical studies on the alkaloids isolated from the tuber of Yuanhu (Corydalis turtschaninovii Bees. f. yanhusuo Y. H. Chou et C. C. Hsu). Acta Pharm. Sin..

[36] He K, Gao JL, Zhao GS (2007). Advances in studies on chemistry, pharmacology, and quality control of Corydalis yanhusuo. Zhong Cao Yao.

[37] Chu H, Jin G, Friedman E, Zhen X (2008). Recent development in studies of tetrahydroprotoberberines: mechanism in antinociception and drug addiction. Cell Mol. Neurobiol..

[38] Letunic I, Bork P (2018). 20 years of the SMART protein domain annotation resource. Nucleic Acids Res..

[39] Botros HG (2013). Crystal structure and functional mapping of human ASMT, the last enzyme of the melatonin synthesis pathway. J. Pineal Res..

[40] Dang TT, Facchini PJ (2012). Characterization of three O-methyltransferases involved in noscapine biosynthesis in opium poppy. Plant Physiol..

[41] Morishige T, Dubouzet E, Choi KB, Yazaki K, Sato F (2002). Molecular cloning of columbamine O-methyltransferase from cultured Coptis japonica cells. Eur. J. Biochem..

[42] Cho HY, Rhee HS, Yoon SY, Park JM (2008). Differential induction of protein expression and benzophenanthridine alkaloid accumulation in Eschscholtzia californica suspension cultures by methyl jasmonate and yeast extract. J. Microbiol. Biotechnol..

[43] Glazebrook J (2001). Genes controlling expression of defense responses in Arabidopsis–2001 status. Curr. Opin. Plant Biol..

[44] Colque R, Viladomat F, Bastida J, Codina C (2004). Improved production of galanthamine and related alkaloids by methyl jasmonate in Narcissus confusus shoot-clumps. Planta Med..

[45] Wei X, Vrieling K, Mulder PPJ, Klinkhamer PGL (2019). Methyl jasmonate changes the composition and distribution rather than the concentration of defence compounds: a study on pyrrolizidine alkaloids. J. Chem. Ecol..

[46] Ketchum RE (2003). Taxus metabolomics: methyl jasmonate preferentially induces production of taxoids oxygenated at C-13 in Taxus x media cell cultures. Phytochemistry.

[47] Cho HY (2008). Synergistic effects of sequential treatment with methyl jasmonate, salicylic acid and yeast extract on benzophenanthridine alkaloid accumulation and protein expression in Eschscholtzia californica suspension cultures. J. Biotechnol..

[48] Shitan N (2003). Involvement of CjMDR1, a plant multidrug-resistance-type ATP-binding cassette protein, in alkaloid transport in Coptis japonica. Proc. Natl Acad. Sci. USA.

[49] Shitan N (2013). Characterization of Coptis japonica CjABCB2, an ATP-binding cassette protein involved in alkaloid transport. Phytochemistry.

[50] Feng Z (2010). Research on the accumulation dynamic of dry matter and two alkaloids in the tubers of Corydalis yanhusuo. J. Anhui Agric. Sci..

[51] Schmidlin L (2008). A stress-inducible resveratrol O-methyltransferase involved in the biosynthesis of pterostilbene in grapevine. Plant Physiol..

[52] Chase MW (2016). An update of the Angiosperm Phylogeny Group classification for the orders and families of flowering plants: APG IV. Bot. J. Linn. Soc..

[53] Ikezawa N, Iwasa K, Sato F (2008). Molecular cloning and characterization of CYP80G2, a cytochrome P450 that catalyzes an intramolecular C-C phenol coupling of (S)-reticuline in magnoflorine biosynthesis, from cultured Coptis japonica cells. J. Biol. Chem..

[54] Li WZ, Jaroszewski L, Godzik A (2002). Tolerating some redundancy significantly speeds up clustering of large protein databases. Bioinformatics.

[55] Finn RD (2016). The Pfam protein families database: towards a more sustainable future. Nucleic Acids Res..

[56] Tatusov RL (2003). The COG database: an updated version includes eukaryotes. BMC Bioinforma..

[57] Bairoch A, Apweiler R (2000). The SWISS-PROT protein sequence database and its supplement TrEMBL in 2000. Nucleic Acids Res..

[58] Kanehisa M, Goto S, Kawashima S, Okuno Y, Hattori M (2004). The KEGG resource for deciphering the genome. Nucleic Acids Res..

[59] Ashburner M (2000). Gene ontology: tool for the unification of biology. The Gene Ontology Consortium. Nat. Genet.

[60] Li B, Dewey CN (2011). RSEM: accurate transcript quantification from RNA-Seq data with or without a reference genome. BMC Bioinforma..

[61] Benjamini Y, Hochberg Y (1995). Controlling the false discovery rate: a practical and powerful approach to multiple testing. J. R. Stat. Soc. B.

[62] Young MD, Wakefield MJ, Smyth GK, Oshlack A (2010). Gene ontology analysis for RNA-seq: accounting for selection bias. Genome Biol..

[63] Bao Z (2020). Identification and selection of reference genes for quantitative transcript analysis in Corydalis yanhusuo. Genes.

[64] Kumar S, Stecher G, Li M, Knyaz C, Tamura K (2018). MEGA X: molecular evolutionary genetics analysis across computing platforms. Mol. Biol. Evol..

[65] He Z (2016). Evolview v2: an online visualization and management tool for customized and annotated phylogenetic trees. Nucleic Acids Res..

