# Supplementary material for: Integration of full-length transcriptomics and targeted metabolomics to identify benzylisoquinoline alkaloid biosynthetic genes in Corydalis yanhusuo
Source: Hortic Res. 2021 Jan 10;8:16. doi: 10.1038/s41438-020-00450-6 (PMC7797006; doi:10.1038/s41438-020-00450-6)
Supplement: Supplementary file 1 — Primer pairs used in qRT-PCR verification [file 41438_2020_450_MOESM1_ESM.pdf]

Table S5. Primer pairs used in qRT-PCR verification

Unigene ID	Enzyme abbreviation	Forward primers	Reverse primers
i1_HQ_YHS_c37524/f6p1/1275	4’-OMT	5' GCTCATCCTCATCCCAACA 3'	5' CGTCAGTCCAGTCGTGCA 3'
i1_LQ_YHS_c14617/f1p0/1365	6-OMT	5' TGAGAAGGCTAACGGAAAG 3'	5' CAACGCCCAAGTAACGAG 3'
i1_HQ_YHS_c17058/f2p0/1721	CFS	5' AGTCGCAGACGGTAAGTT 3'	5' ACTAGCAAGAGCAAACCC 3'
i1_LQ_YHS_c4736/f5p0/1766	DBOX	5' ACTCCTCCAACTTACCCA 3'	5' TACATGATCCCTGTCCAA 3'
i1_LQ_YHS_c26267/f1p0/1789	STOX	5' GAAGACCTATTCTGGGCTAT 3'	5' CTCCTGACCGTAAACACC 3'
i1_HQ_YHS_c39834/f10p10/1975	MSH	5' CCATTTGGAACGGGTAGA 3'	5' CCTGCGGTTTCAGTCATA 3'
i2_LQ_YHS_c51026/f1p24/2331	NCS	5' TTGCCCAGTGGACCTAAT 3'	5' CTGCCTGAGCACATAACC 3'
i1_HQ_YHS_c39871/f17p0/1638	NMCH	5' TGCCTTGCTTATGGAACT 3'	5' GCTCTGCTCGTATCTTGG 3'
i1_LQ_YHS_c21069/f1p0/1820	P6H	5' TTTGGTCTAACCCGACAG 3'	5' ACCCTTCTACCTGCTCCA 3'
i1_LQ_YHS_c42734/f2p0/1369	TNMT	5' GGGAATCACTTGGCTCGTT 3'	5' TTGGTCCACTGCTTCTTTGTT 3'
i2_LQ_YHS_c53720/f1p1/2154	3-OHase	5' GTTCTCATAGGTTTAGGAGG[illegible]	5' TTGGACAAGTCTGGTGGC 3'
i1_LQ_YHS_c2693/f4p2/1575	TyrAT	5' GATAACCCTTTCGTGCCG 3'	5' CCACCCAAGTCTCCATCC 3'
i4_LQ_YHS_c8977/f1p2/4550	4-HPPDC	5' CTGGGTTGGGTGACTTCT 3'	5' ATCACTCCACCACCTTGC 3'
i2_LQ_YHS_c5951/f1p54/2944	SPS/CAS	5' TCACAACCCGAAAGTATGG	5' CAGCACTGAATGGCAACA 3'
i2_LQ_YHS_c33007/f1p6/2484	TyDC	5' GCACAAATCGCAGGAATC 3'	5' CACGGTTGGACAAAGGAA 3'
i1_HQ_YHS_c23730/f2p0/1758	BBE	5' GCCGATACACCGTTCGTAAT	5' GCACCTGTTTCAACCCAAGC 3'
i1_HQ_YHS_c1985/f4p11/1737	CNMT	5' CGGTGACAGTGTTCTTGATC	5' TAAGGTTCCTGGATTGTTCT 3'
