# Supplementary material for: Integration of full-length transcriptomics and targeted metabolomics to identify benzylisoquinoline alkaloid biosynthetic genes in Corydalis yanhusuo
Source: Hortic Res. 2021 Jan 10;8:16. doi: 10.1038/s41438-020-00450-6 (PMC7797006; doi:10.1038/s41438-020-00450-6)
Supplement: Supplementary file 2 — Integration of full-length transcriptome and targeted metabolomics for identifying Benzylisoquinoline Alkaloid Biosynthetic genes in Corydalis yanhusuo [file 41438_2020_450_MOESM2_ESM.docx]

**Supporting Information**

**Integration of full-length transcriptome and targeted metabolomics for identifying Benzylisoquinoline Alkaloid Biosynthetic genes in *Corydalis yanhusuo***

Dingqiao Xu^#1^, Hanfeng Lin^#2^, Yuping Tang^1^, Lu Huang^1^, Jian Xu^2^, and Sihui Nian*^3^, Yucheng Zhao*^2^

^1^ Key Laboratory of Shaanxi Administration of Traditional Chinese Medicine for TCM Compatibility, and State Key Laboratory of Research & Development of Characteristic Qin Medicine Resources (Cultivation), and Shaanxi Key Laboratory of Chinese Medicine Fundamentals and New Drugs Research, and Shaanxi Collaborative Innovation Center of Chinese Medicinal Resources Industrialization, Shaanxi University of Chinese Medicine, Xi’an 712046, Shaanxi, China

^2^ Department of Resources Science of Traditional Chinese Medicines and State Key Laboratory of Natural Medicines, School of Traditional Chinese Pharmacy, China Pharmaceutical University, Nanjing 210009, Jiangsu, China.

^3^ Institute of Modern Chinese Medicine, School of Pharmacy, Wannan Medical College, Wuhu 241002, Anhui, China

^#^These authors contributed equally to this work.

^*^To whom correspondence should be address to Yucheng Zhao and Sihui Nian.

Tel/Fax: +86-25-83271248 or +86-553-3932622

E-mail: [zhaoyucheng1986@126.com](mailto:zhaoyucheng1986@126.com) (Y.C. Z), [niansihui@126.com](mailto:niansihui@126.com) (S.H. N)

Running title: Benzylisoquinoline Alkaloid Biosynthetic in *C. yanhusuo*

**Supplementary method**

1. **Chemical and reagents**

A total of 19 reference standards (purity ≥ 98 %), including scoulerine, tetrahydroberberine, tetrahydrocolumbamine, columbamine, tetrahydropalmatine, sanguinarine, corydaline, protopine, tetrahydrocoptisine, noroxyhydrastinine, dehydrocorydaline, oxoglaucine, 8-oxycoptisine, palmatine, coptisine, berberine, jatrorrhizine, epiberberine, and glaucine were purchased from Qingdao Jieshikang Technology Co., Ltd. Acetonitrile and methanol were obtained from ROE Scientific Inc. (Newark USA). HPLC-grade formic acid from Sigma (St. Louis, MO, USA) was used as additives in the mobile phase. Deionized water used for all experiments were produced using a Milli-Q purification system (Millipore Corp., Bedford, MA, USA).

1. **Sample preparation**

The tuber and leaf samples were pulverized into homoge- neous powder (through a 50-mesh sieve) after being dried at 60 °C under reduced pressure. An aliquot of 200 mg of each sample powder was accurately weighed and placed in a 50 mL centrifuge tube. Ultrasonic extraction (30 kHz) was performed in 25 mL of methanol at room temperature for 30 min. To an adequate volume of the methanol extract, an equal volume of methanol was added, and later centrifuged at 12,000 rpm for 10 min. The supernatant was used as the sample solution. Two replicates were prepared for each sample. For validation of the analytical method, a pooled sample of all the samples was prepared as the quality control (QC) sample.

For the reference standards, 1 mg was dissolved in 0.5 mL of acetonitrile and then diluted to 1 mL with acetonitrile. Nineteen mixed reference standard solutions were prepared according to their chemical classification and molecular weight. All the solutions were stored at 4 °C prior to analysis.

1. **QTOF-MS Analysis for global metabolomics**

For metabolomics experiments, every nine samples from each group were all used to analyze separately. Analyses were performed using a Waters ACQUITY UPLC system coupled with a SYNAPT G2-Si HDMS quadrupole-time-of-flight mass spectrometry, Q-TOF-MS. Samples were analyzed using an ACQUITY UPLC BEH column (2.1 mm × 100 mm, 1.7 μm, Waters Corporation, Milford, MA, USA) in positive mode. The column temperature was maintained at 40 °C and the flow rate of mobile phase was 0.30 mL/min, accompanied with an injection volume of 1.0 µL. Mobile phase A was 0.1% (*v/v*) formic acid/acetonitrile, while mobile phase B was 0.1% (*v/v*) formic acid/water. The automatic sampler was set at 4 °C during all samples analysis. The column was eluted with a linear gradient of 10 % A over 0 -1.6 min, 10 - 23 % A over 1.6 - 3 min, 23 – 28 % A over 3- 6 min, 28 – 60 % A over 6 - 9 min, 60 – 90 % A over 9 - 11 min, 90 – 90 % A over 11 - 12 min, 90 – 10 % A over 12 - 13 min, 10 – 10 % A over 13 - 14 min. The flow rate was set at 0.3 mL•min^-1^.

High-accuracy MS data were recorded in positive ionization modes controlled by MassLynx 4.2 (Waters, Manchester, UK). Capillary voltage was 3.0 kV for positive mode. Source temperature was set at 150 °C with a cone gas flow of 50 L/h, and desolvation temperature was set at 300 °C with desolvation gas flow of 850 L/h. Leucine-enkephalin (Waters Co., Manchester, UK) was used as the lock mass generating a reference ion at *m/z* 556.2771 in positive mode, which was introduced by a lockspray at 5 μL/min for data calibration. The MS^E^ data were acquired in centroid mode using ramp collision energy in two scan functions. For Function 1 (low energy), scan range 100-1000 Da, scan time 0.25 s, and collision energy 10 V were set (Fig. S3). In the case of Function 2 (high energy), scan range 100-1000 Da, scan time 0.25 s, and a collision energy ramp 20- 50 V were employed.

All data were acquired in MassLynx V4.2 and were imported into Progenesis QI V2.0 (Waters Corporation, Milford, MA, USA) to clean background noise, be normalized by a reference sample, correct retention time, pick peak and identify compounds with databases such as METLIN, HMDB, and Lipid Maps ^1-3^. Structural confirmation was conducted by comparison with the reference standards (t_R_ and MS, MS/MS data) or matching with theoretical data or commercial library (Fig. S3, Fig. S6).

1. **Data processing by Progenesis QI**

Efficient metabolomics data processing was performed by Progenesis QI, while pattern-recognition chemometrics was applied for species classification and potential markers discovery. The detailed workflow for data processing facilitated by Progenesis QI is shown in Fig. S5, which involved “create a new experiment”, “import data”, “review alignment”, “experiment design setup”, “peak picking”, “review deconvolution”, and “identify compounds” in sequence. In general, the whole process ran automatically using default parameter settings ^4-5^. Here, processing of the positive mode MS^E^ data was taken as an example to illustrate the entire process by use of Progenesis QI.

1. In the stage of create a new experiment, adduct ion was carefully selected as it would influence the number of characterized compounds and also the identification accuracy.
2. The MS^E^ data acquired by UPLC-QTOF-MS for the tuber and leaf samples were imported into the Progenesis QI software, generating a 2D ion intensity map with the retention time and m/z information as the ordinate and abscissa, respectively. Both the low energy and high energy data are displayed in the window (Fig. S5a).
3. Peak alignment was carried out in automatic manner taking a QC run as the reference.
4. In the Experiment designs stage, only one group were set up for the purpose of holistically viewing the general clustering and discrepancy of the tested samples in PCA.
5. For peak picking, the thresholds of sensitivity, chromatographic peak width, and retention time limits can be set to achieve the maximum real ion signals with noise excluded.
6. The adduct ions corresponding to the same compound were well grouped in the deconvolution stage.
7. Further compound identification was performed by searching the HMDB database. The identification results combined with the intensity data were exported as .*xls* files for subsequent compound confirmation and multivariate statistical analysis.

**5. Multivariate statistical analysis**

The multivariate data matrix was analyzed by EZinfo software 2.8 (Waters Corp., Milford, USA). All the variables were mean-centered and Pareto-scaled prior to PCA and PLS-DA. Here, “Unsupervised” PCA and “Supervised” OPLS-DA were used to process the acquired UPLC-MS data. In the PCA scores, the PCA results are displayed as score plots indicating the scatter of the samples, which indicates similar metabolomics compositions when clustered together, and compositionally different metabolomes when dispersed. Additionally, in order to maximize the variations between tuber and leaf samples, sophisticated supervised methods including OPLS-DA was further employed. R^2^X and R^2^Y represent the raction of the variance of X matrix and Y matrix, respectively, while Q^2^Y suggests the predictive accuracy of the model. The cumulative values of R^2^X, R^2^Y and Q^2^Y close to 1 indicate an excellent model. Variable influence on projection (VIP) values greater than 1.0 is considered statistically significant for group discrimination.

**6. TQ-MS Analysis for targeted metabolomics**

Chromatographic separation was performed on a Waters ACQUITY UPLC I-Class system coupled to a Waters Xevo TQ-XS mass spectrometer (Waters Co., Manchester, UK) via an electrospray ionization (ESI) interface ^6^. An aliquotof 1 μL of each sample solution was injected onto an ACQUITY UPLC BEH column (2.1 mm× 100 mm, 1.7 μm; Waters Corporation, Milford, MA, USA) equipped with an ACQUITY UPLC BEH 1.7 μm Van Guard Pre-column. The column temperature was held at 40 °C, and the flow rate was set at 0.3 mL/min. The mobile phase consisted of A (acetonitrile) and B (0.1 % formic acid). A linear gradient elution program was used: 0-1.6 min, 10-10 % (A); 1.6-3min, 10-23 % (A); 3-6 min, 23-28 % (A); 6-9 min, 28-60 % (A); 9-11 min, 60-90 % (A); 12-13 min, 90-10 % (A); and 13-14 min, 10 % (A).

High-accuracy MS data was recorded in positive modes controlled by MassLynx 4.2 (Waters, Manchester, UK). Capillary voltage was 3.0 kV for positive mode, whereas cone voltage was 25 V Source temperature was set at 150 °C with a cone gas flow of 50 L/h, and desolvation temperature was set at 450 °C with desolvation gas flow of 850 L/h.

**7. Method validation**

Linearity was evaluated by analysing the standard solutions of at seven different concentration levels. Based on the obtained results, the linearity of the analytical response across the studied range was excellent.

Sensitivity of the method was evaluated by determining limits of detection (LOD) and the pre-determined values of quantitation limits (LOQ). LOD and LOQ were defined at the concentration with a signal-to-noise ratio (determined by peak height) of at least 3 and 10, respectively. The parameter was determined empirically by triplicate analysis of a series of concentrations of standard solution. Calibration curves constructed from the peak area ratios of each analyst using a 1/*x*^2^ weighted linear least – squares regression model. The LLOQ is defined as the lowest concentration on the calibration curve at which at acceptable accuracy (RE) within ± 20% and a precision below 20% can be obtained.

Method precision was checked by intra-day and inter-day variability. The intra-day variability study was conducted by the injection of the same standard solution at six consecutive times in the same day. The inter-day variability study was conducted for three successive days using the same solution. The precision were expressed in terms of relative standard deviation (RSD). Based on our results, the developed method was precise.

The recovery rate was performed by adding a known amount of standards into a certain amount of *C. yanhusuo* extract. The mixture was extracted and analysed using the method mentioned above. Three replicates were performed for the test.

Samples were alternated in random order in the analysis batch. Before analysing the sample sequence, quality control (QC) samples were run. To visually evaluate chromatographic reproducibility, during analysis of the sample sequence, the QC sample was analysed repeatedly with the analytical run after every nine samples. The current UPLC-TQ-MS/MS method was validate for its linearity, intra - day and inter - day precision, accuracy, stability, extraction recovery and matrix effect.

The nineteen marker compounds in leaf and tuber group were detected in this manuscript (Fig. S9).

References

[1] Qiu S., *et al.* Nontargeted metabolomic analysis and “commercial-homophyletic” comparison-induced biomarkers verification for the systematic chemical differentiation of five different parts of Panax ginseng. *J Chromatogr A* **1453**, 78-87 (2016).

[2] Xu X.F., *et al.* Chemical comparison of two drying methods of mountain cultivated ginseng by UPLC-QOTF-MS/MS and multivariate statistical analysis. *Molecules*. **27**, 717 (2017).

[3] Li Y., *et al*. A novel approach to transforming a non-targeted metabolic profiling method to a pseudo-targeted method using the retention time locking gas chromatography/mass spectrometry-selected ions monitoring. *J Chromatogr A* **1255**, 228-236 (2012).

[4] Zhao Y.N., *et al.* Metabolic response of rice leaves and seeds under transgenic backcross breeding and pesticide stress by pseudotargeted metabolomics. *Metabolomics.* **11**, 1802-1814 (2015).

[5] Naz S., *et al*. Development of a liquid chromatography-high resolution mass spectrometry metabolomics method with high specificity for metabolite identification using all ion fragmentation acquisition. *Anal Chem* **89**, 7933-7942 (2017).

[6] Wang J, *et al.* Quantitative comparison and metabolite profiling of saponins in different parts of the root of Panax notoginseng. *J Agricultural Food Chem* **62**, 9024-9034 (2014).

**Supplemental Figures**


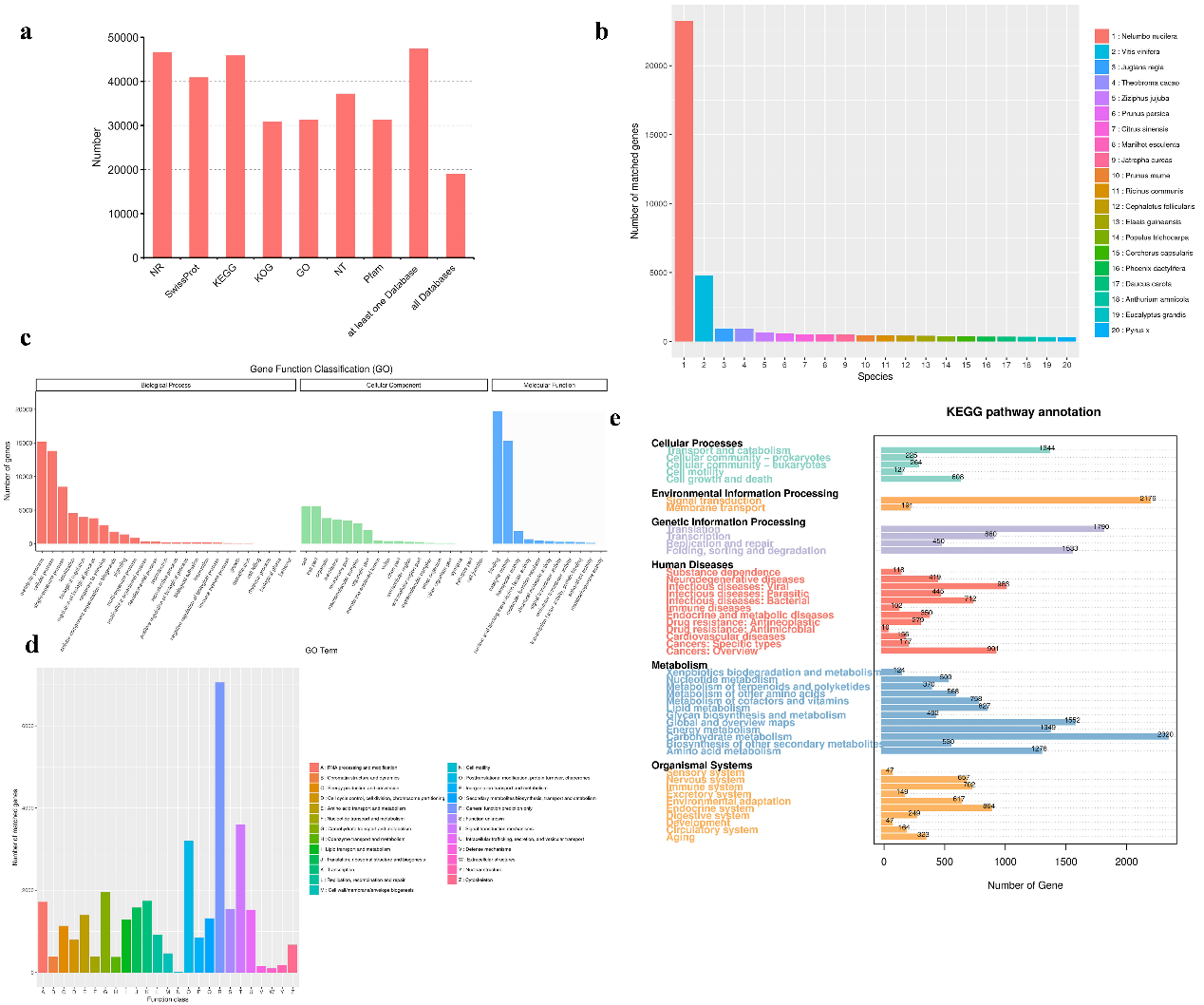


Fig. S1 The unigenes using the data from seven nucleotide and protein databases (NR, NT, Pfam, Swiss-Prot, KOG, KEGG, GO) were functionally annotated.


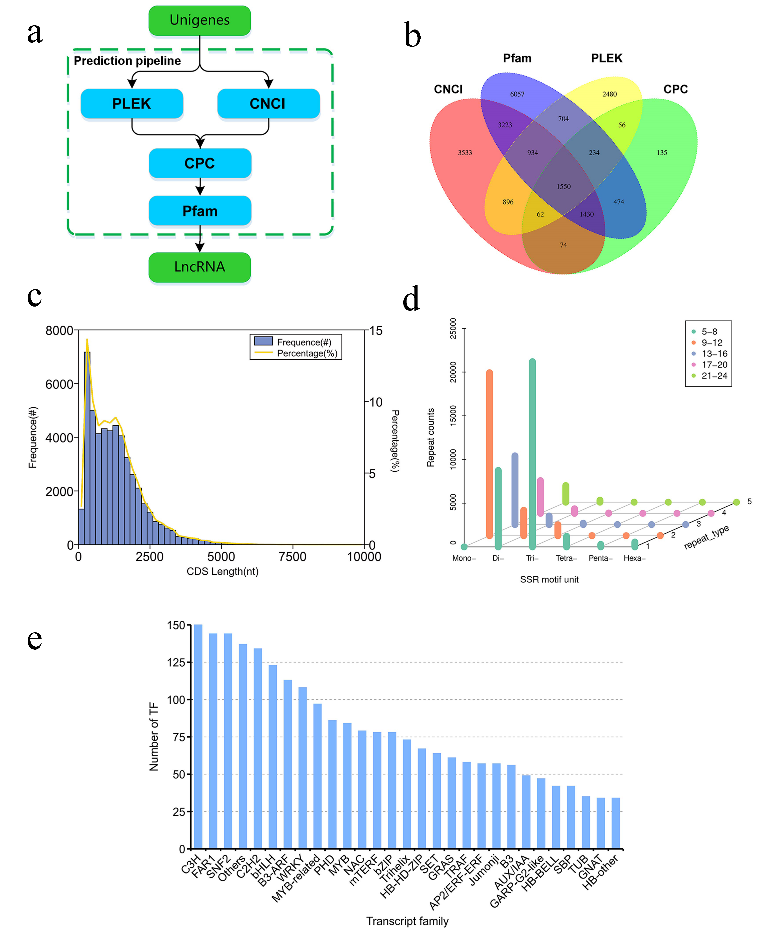


Fig. S2 The gene structures and transcription factors are predicted by the full-length transcripts data.


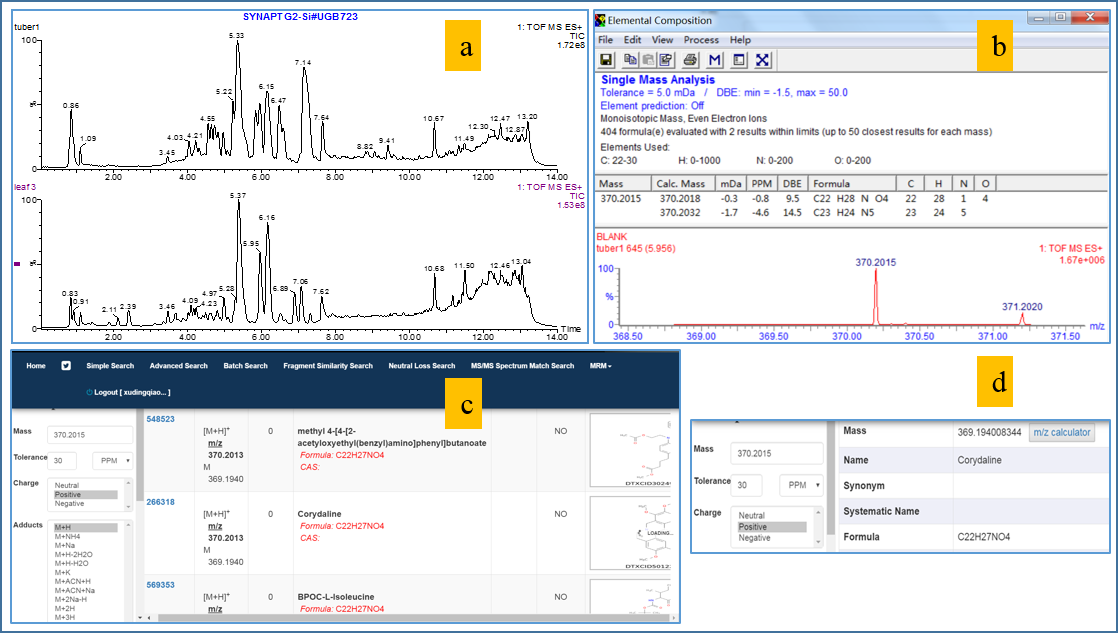


Fig. S3 Analysis of compounds extracted from tuber using UPLC-QTOF/MS. (a) Total ion chromatogram (TIC) of the tuber and leaf of *C. yanhusuo* extract analyzed by UPLC-QTOF-MS in a positive ion mode. Analysis performed in positive ion mode detected an average of 53 compounds, each assigned their own chromatographic retention time. (b) The accurate mass and elemental composition for the precursor ions and fragment ions were analyzed with the MassLynxV4.2 software (Waters Co., Mil-ford, USA). (c, d) The identity of compound was confirmed by searching the MS/MS spectrum from METLIN (<https://metlin.scripps.edu/index.php>) (Corydaline, compound 5.956_370.2015 as an example).


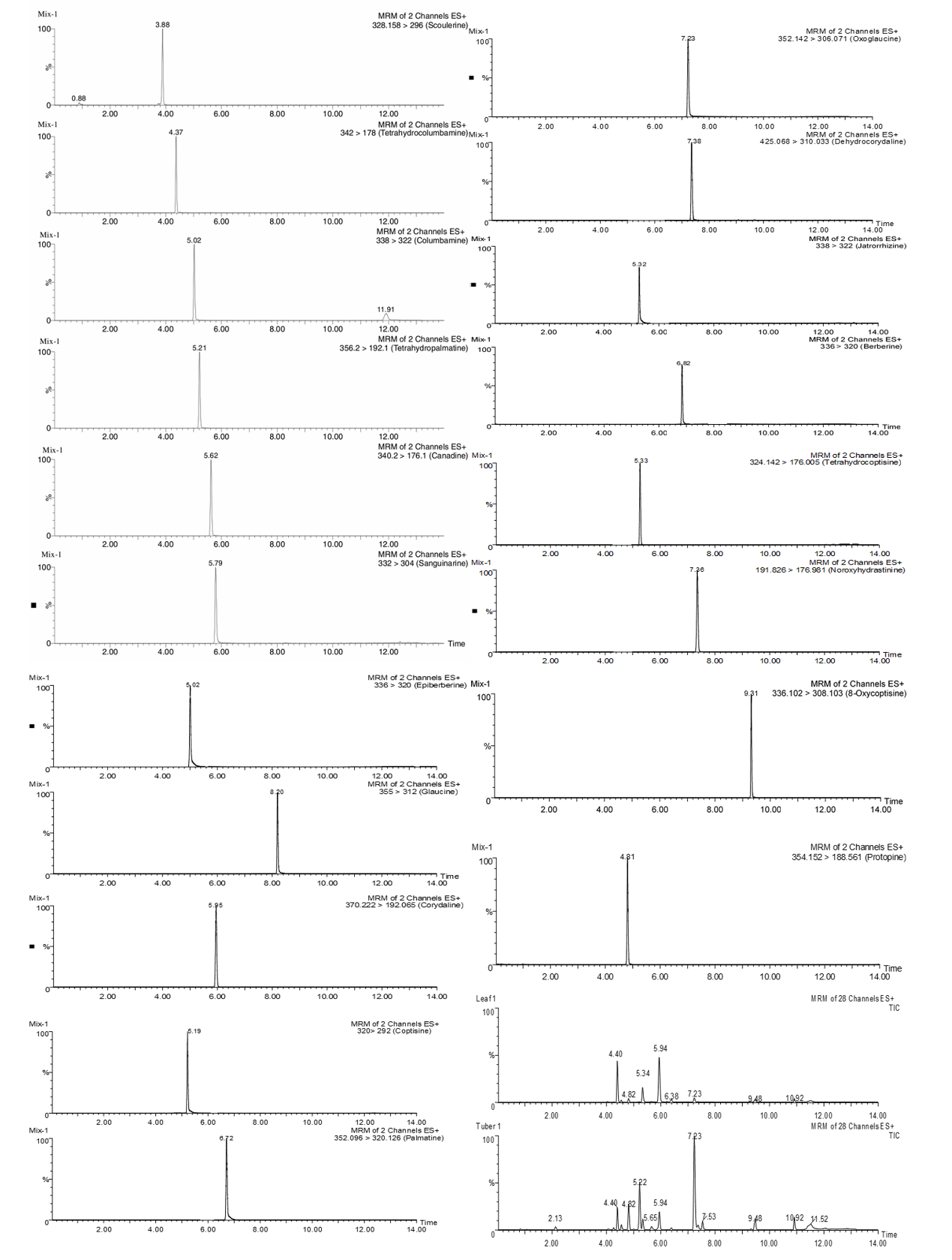


Fig. S4 The metabolic changes of marker compounds between tuber and leaf groups were accurately quantified through a subsequent targeted metabolomics analysis. The total ion chromatograms (TIC) of the standard samples were obtained and displayed.


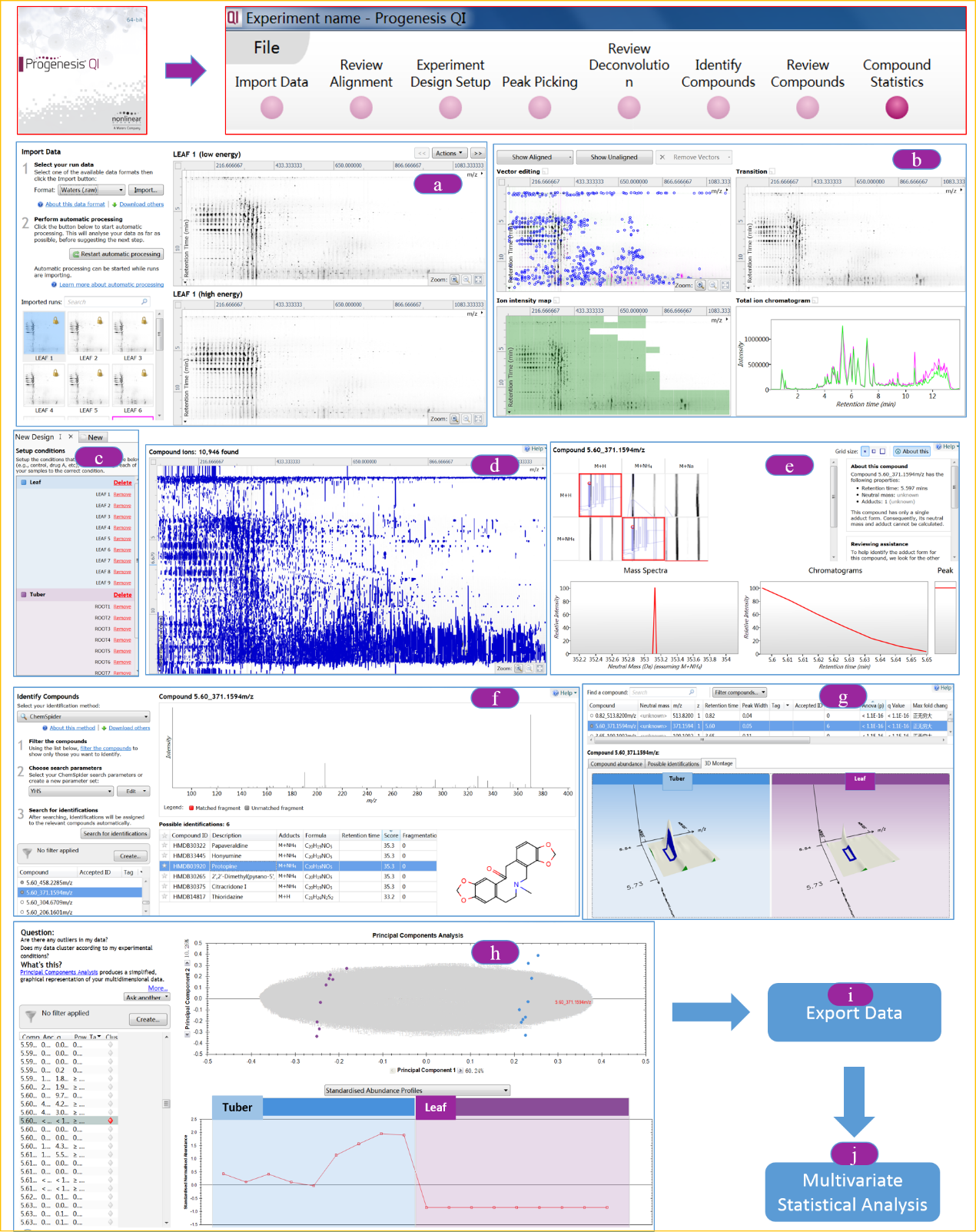


Fig. S5 Visualized menu-guided workflow for computational processing of high-accuracy MS data by Progenesis QI (a) import data; (b) review alignment; (c) experiment design setup; (d) peak picking; (e) review deconvolution; (f) identify compounds; (g) review compounds; (h) compound identification result, taking compound 5.60_371.1594 as an example.


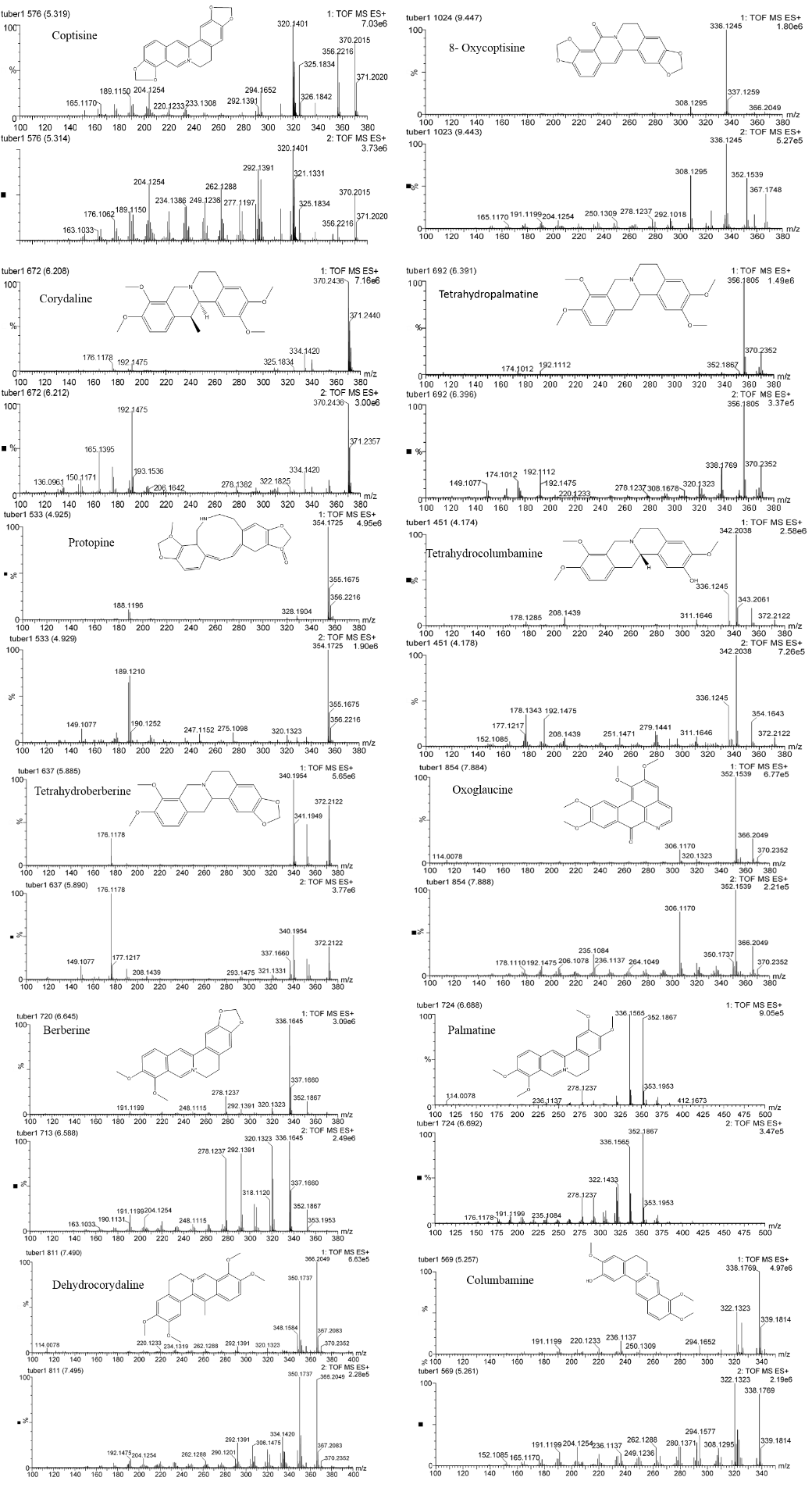


Fig. S6 Structural confirmation was conducted by comparison with the reference standards (t_R_ and MS, MS/MS data) or matching with theoretical data or commercial library.


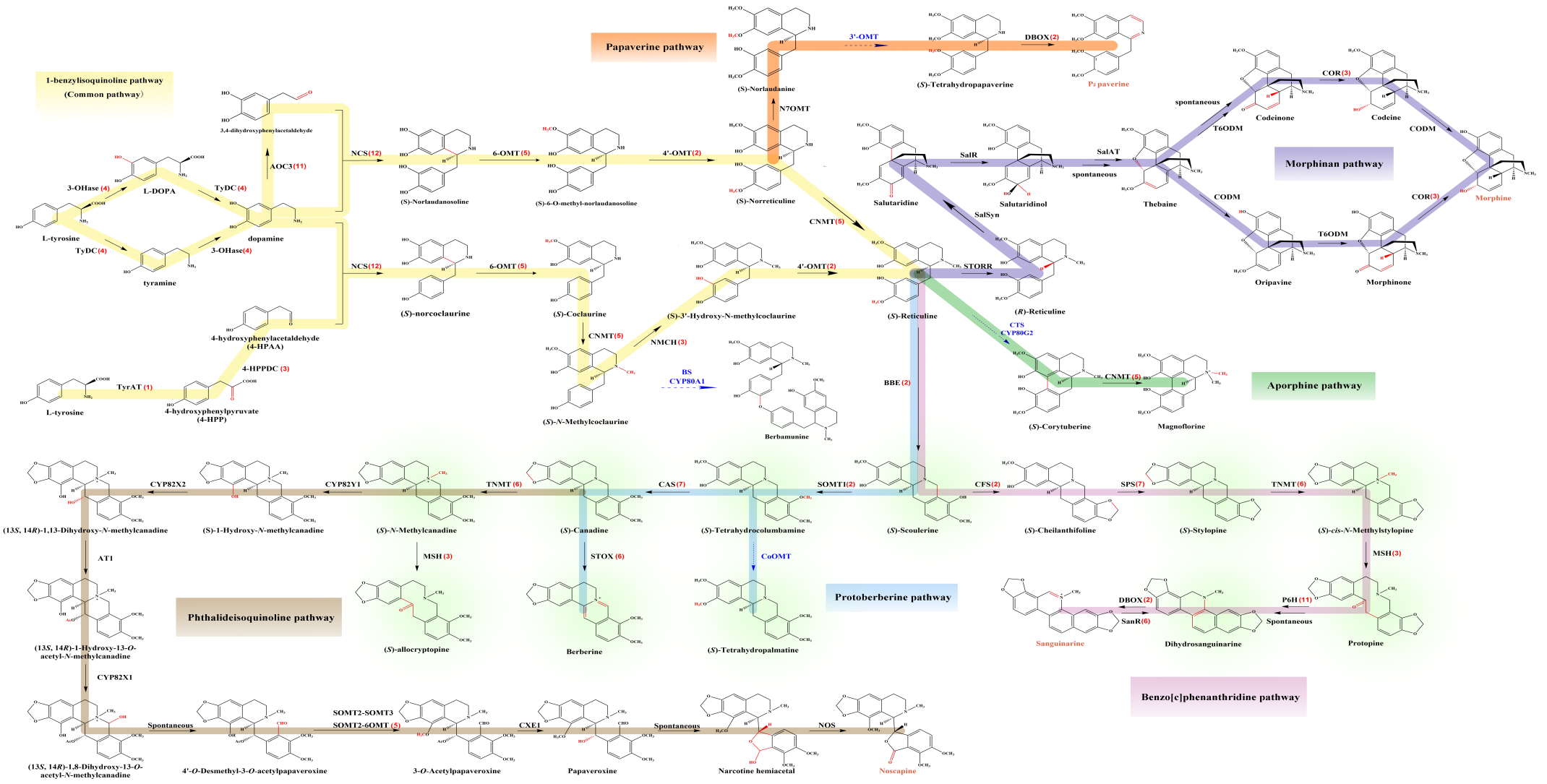


Fig. S7 101 non-redundant unigenes are involved in BIA biosynthetic pathway according to the tblastn results. Highlighted compounds are those previously reported in *C. yanhusuo*. Red numbers after each enzyme are the number of unigenes blasted to their homologs with an identity over 60%. Enzymes catalyzing reactions with blue arrows have not been identified in Papaveraceae Family.

**
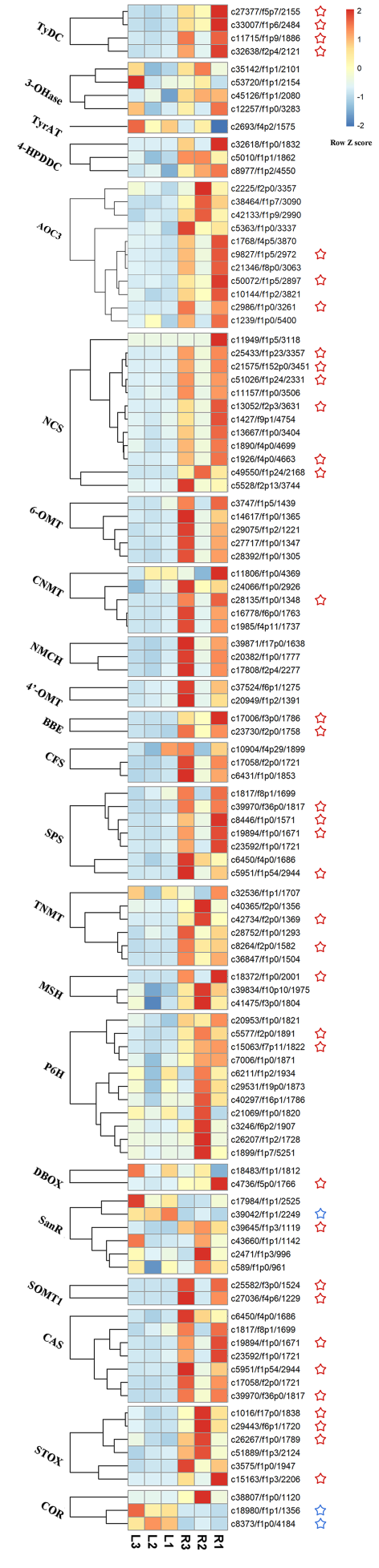
**

Fig. S8 Expression profile of all the BIA-biosynthesis candidate genes. Three biological replicas from leaf (L) and tuber (R) were plotted individually. Unigenes labeled by red asterisks are DEGs predominant in tubers, while blue asterisks are predominant in leaves.


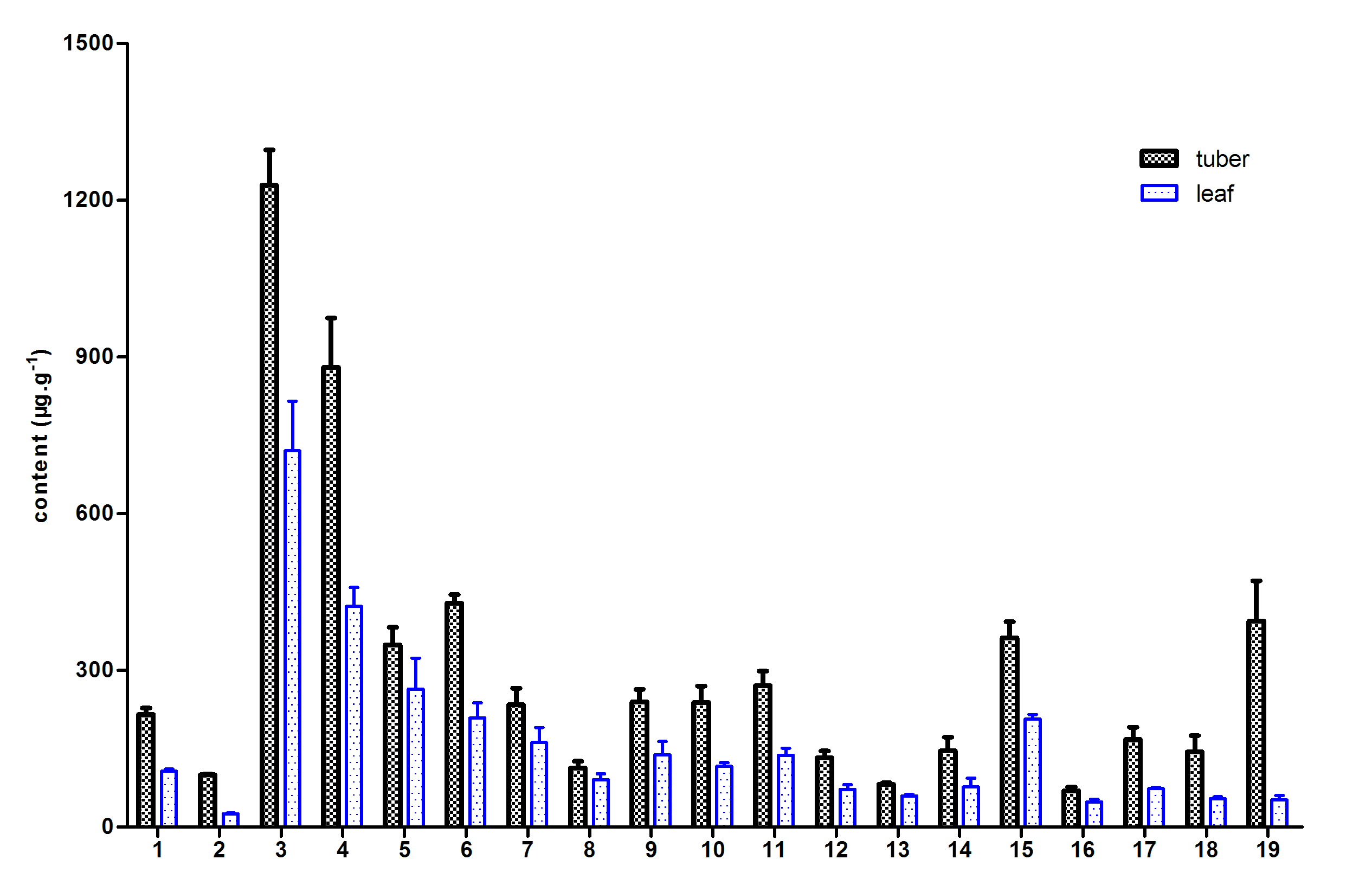


Fig. S9 The content of 19 marker compounds were successfully detected by Waters Xevo TQ-XS mass spectrometer via an electrospray ionization (ESI) interface. 1. Sanguinarine; 2. Tetrahydroberberine; 3. Tetrahydropalmatine; 4. Coptisine; 5. Palmatine; 6. Scoulerine; 7. Corydaline; 8. Columbamine; 9. Oxoglaucine; 10. Tetrahydrocoptisine; 11. Noroxyhydrastinine; 12. Dehydrocorydaline; 13. Berberine; 14. 8-Oxycoptisine; 15. Glaucine; 16. Epiberberine; 17. Tetrahydrocolumbamine; 18. Jatrorrhizine; 19. Protopine.


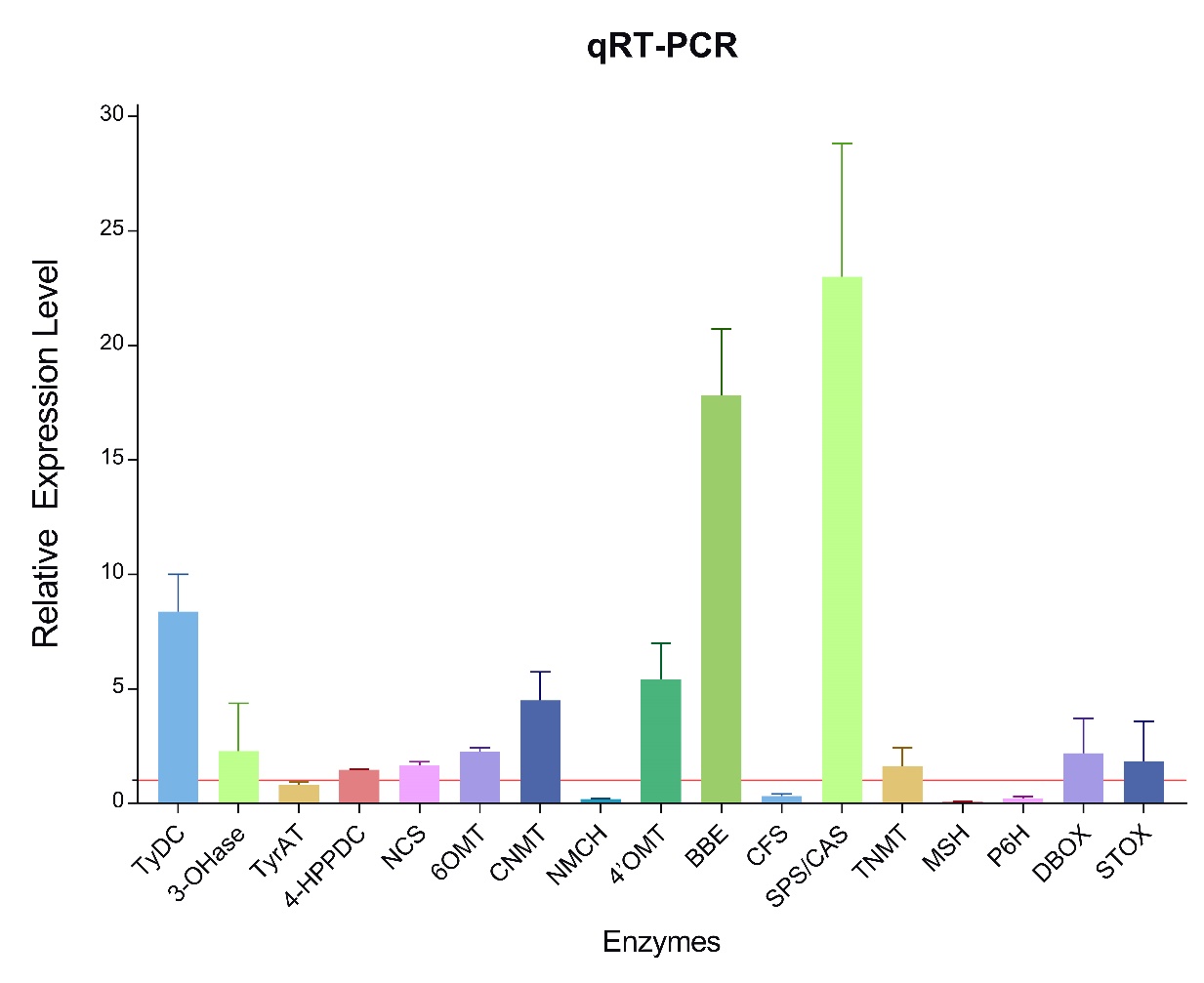


Fig. S10 Such genes were validated for their presence in *C. yanhusuo* and relevance with alkaloid biosynthesis by conducting qRT-PCR analysis between leaves and tubers.
