# Supplementary figures and images for: Integration of full-length transcriptomics and targeted metabolomics to identify benzylisoquinoline alkaloid biosynthetic genes in Corydalis yanhusuo
Source: Hortic Res. 2021 Jan 10;8:16. doi: 10.1038/s41438-020-00450-6 (PMC7797006; doi:10.1038/s41438-020-00450-6)

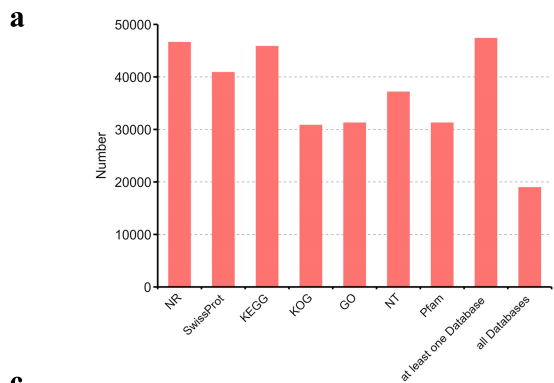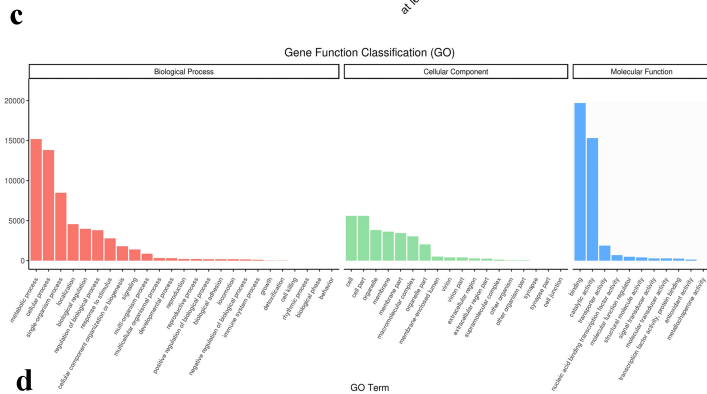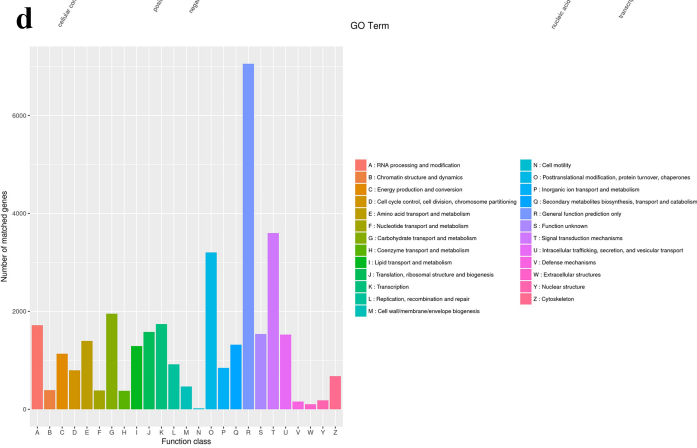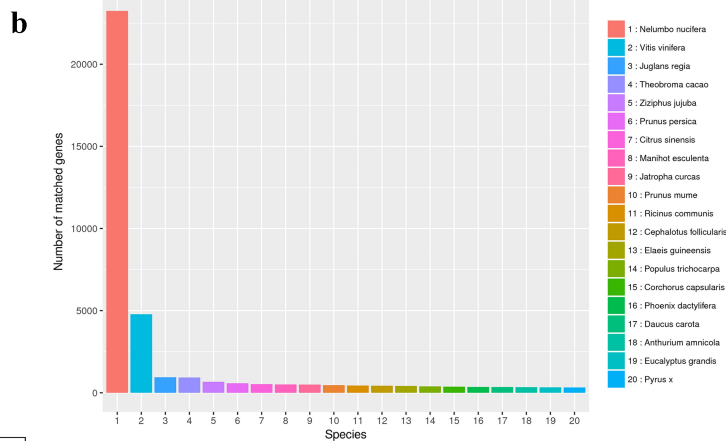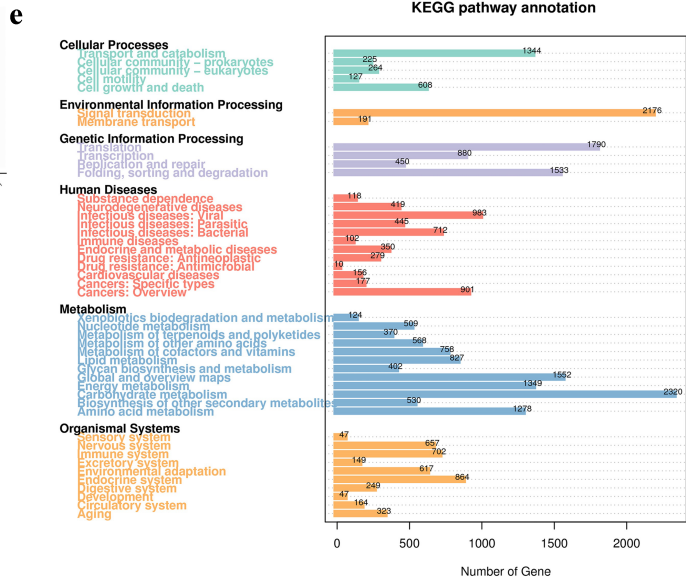

Supplement: Supplementary file 3 — Figure S1 [file 41438_2020_450_MOESM3_ESM.pdf]

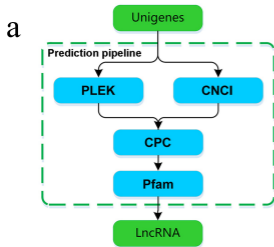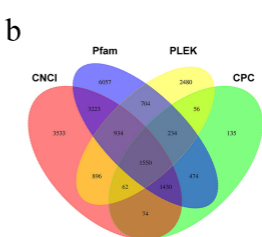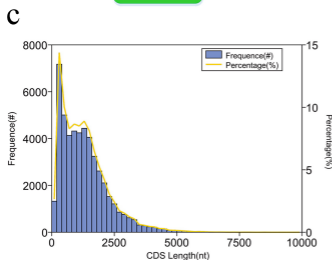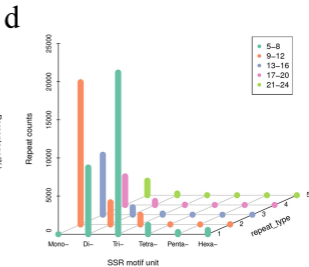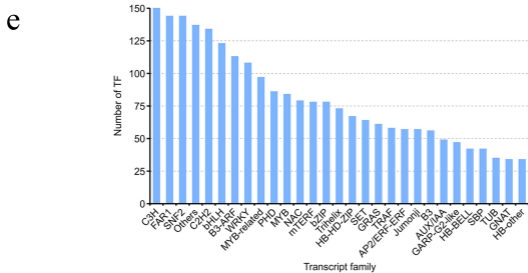

Supplement: Supplementary file 4 — Figure S2 [file 41438_2020_450_MOESM4_ESM.pdf]

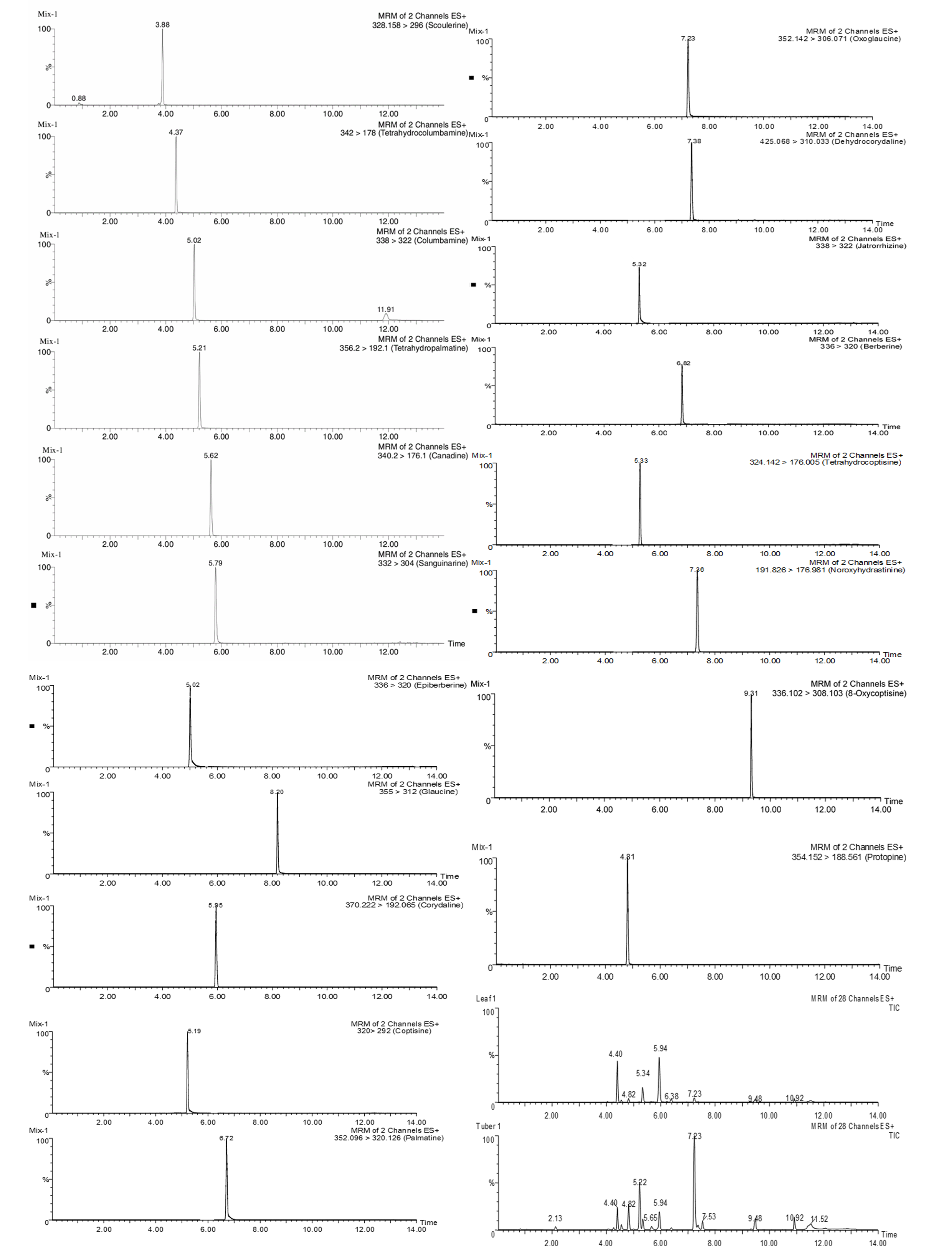

Supplement: Supplementary file 6 — Figure S4 [file 41438_2020_450_MOESM6_ESM.tif]

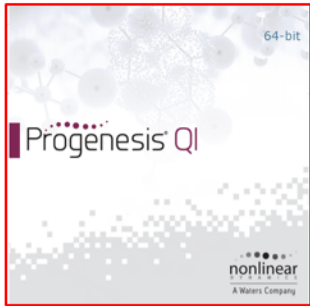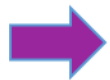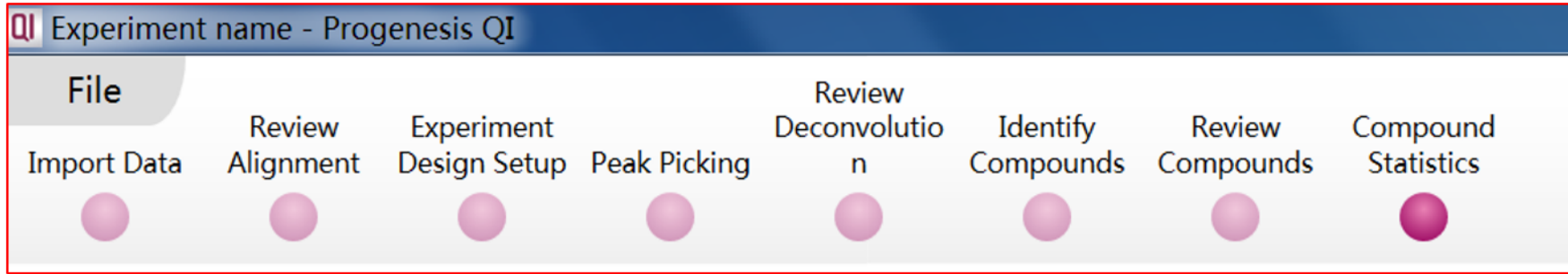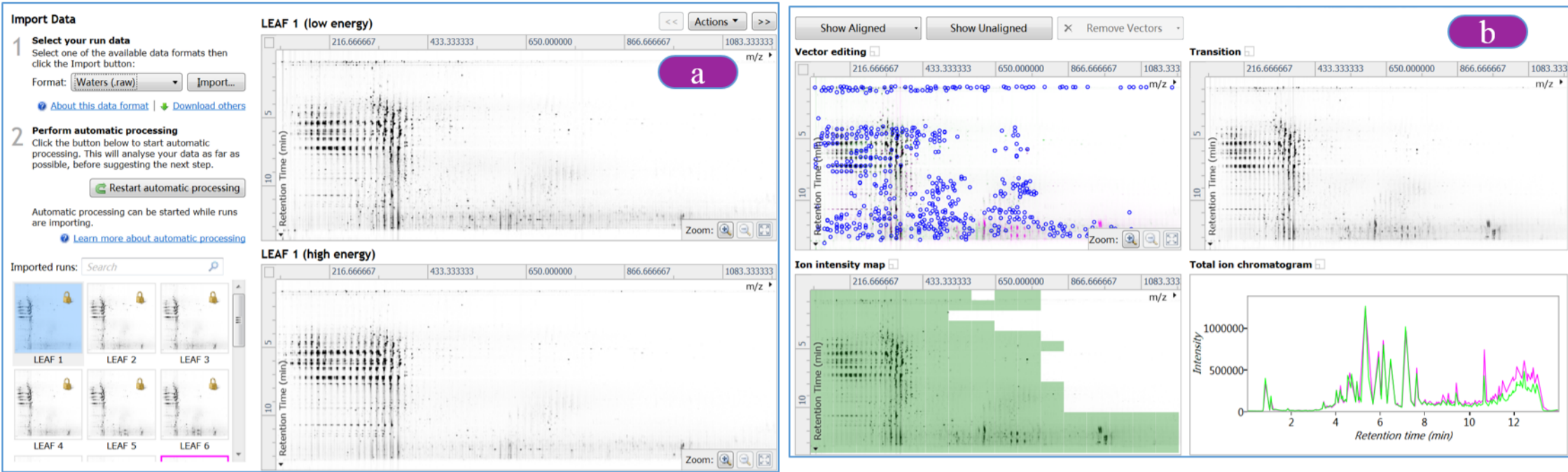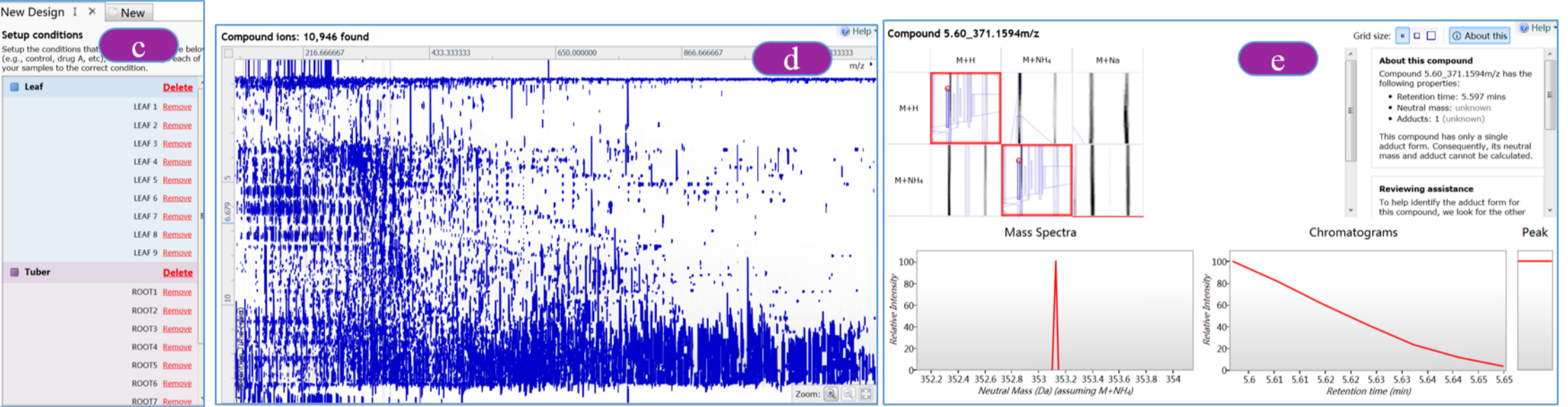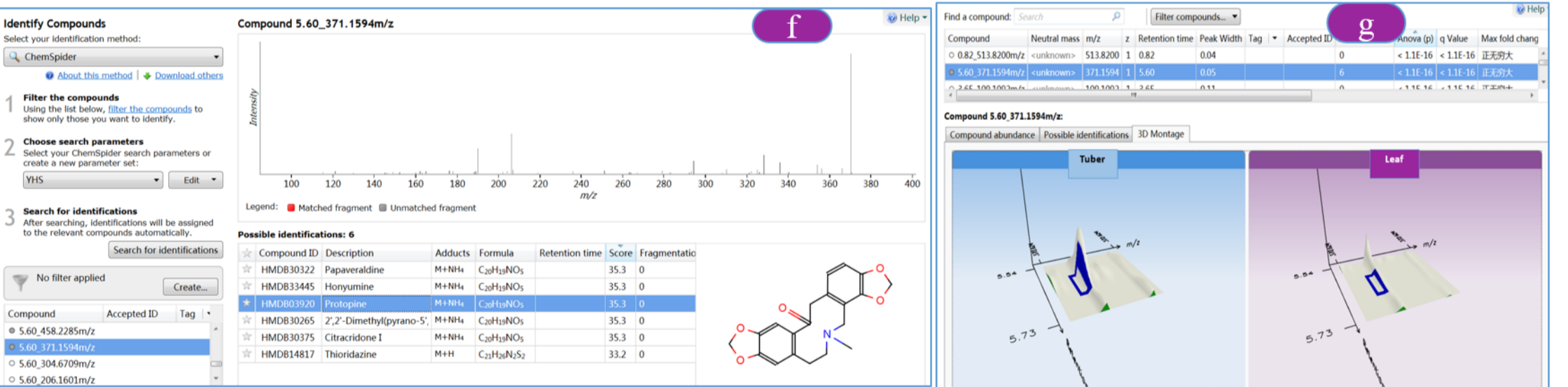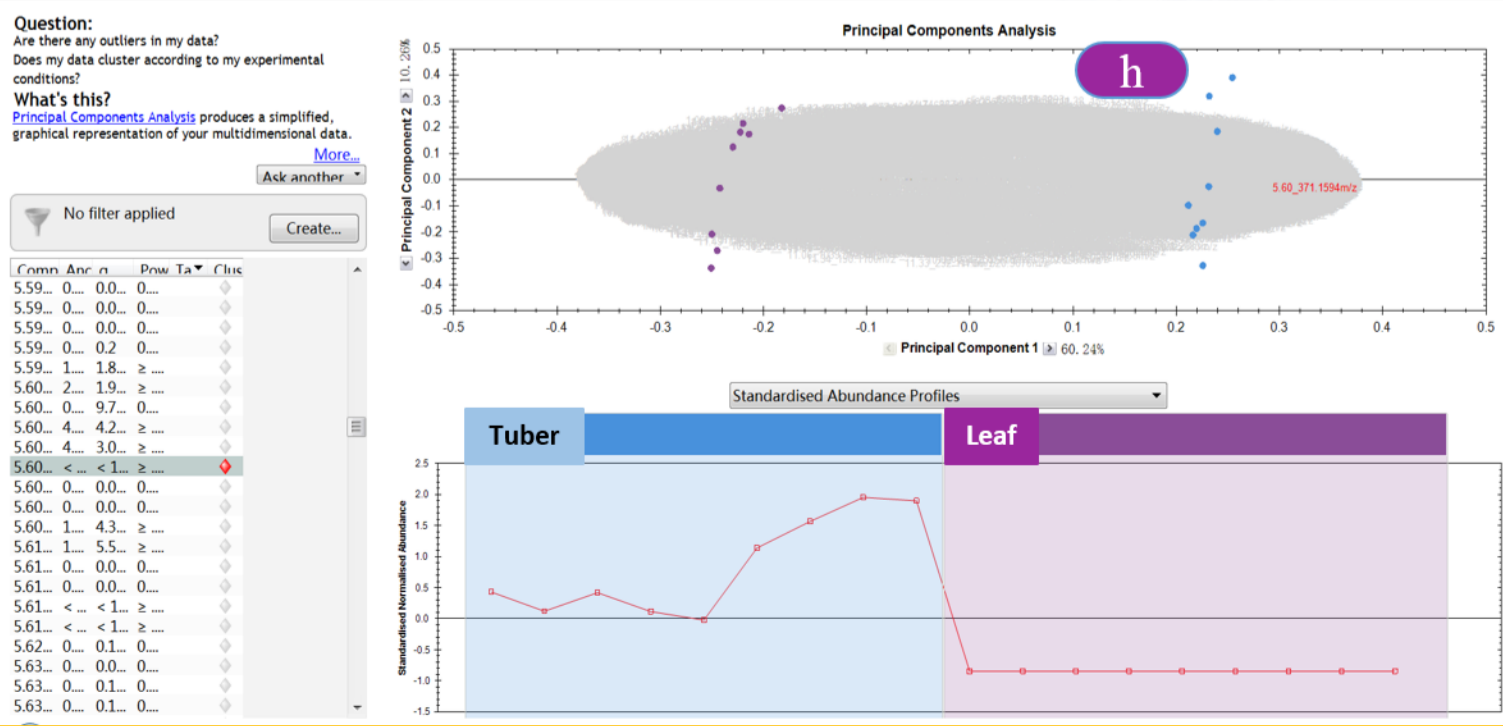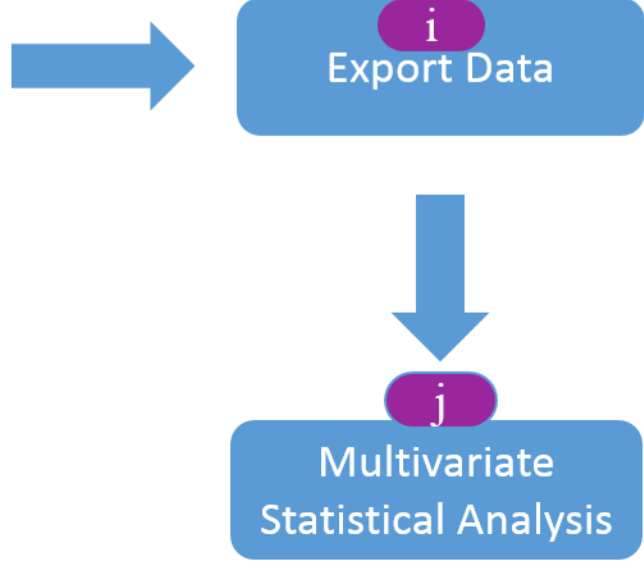

Supplement: Supplementary file 7 — Figure S5 [file 41438_2020_450_MOESM7_ESM.pdf]

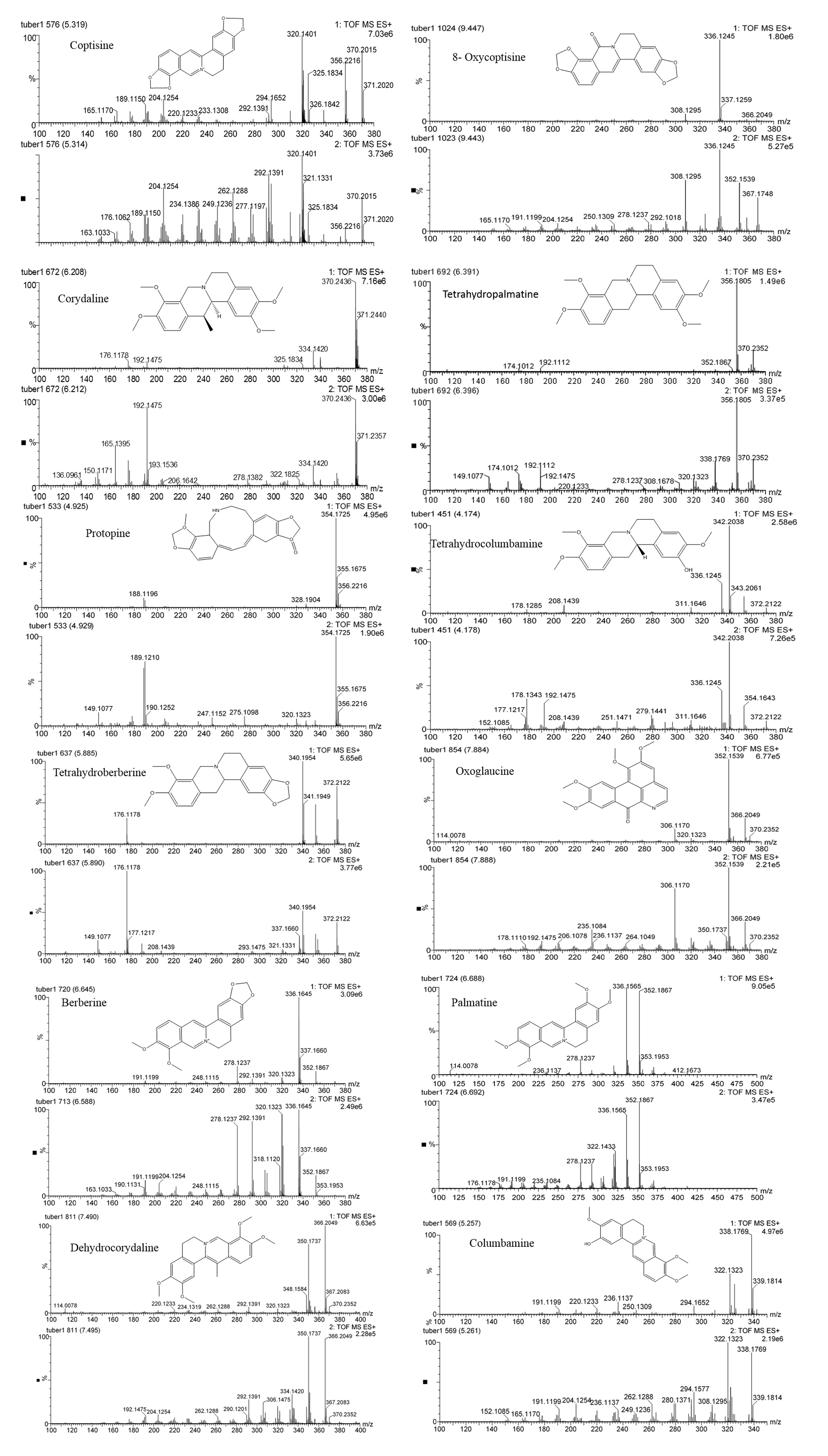

Supplement: Supplementary file 8 — Figure S6 [file 41438_2020_450_MOESM8_ESM.tif]

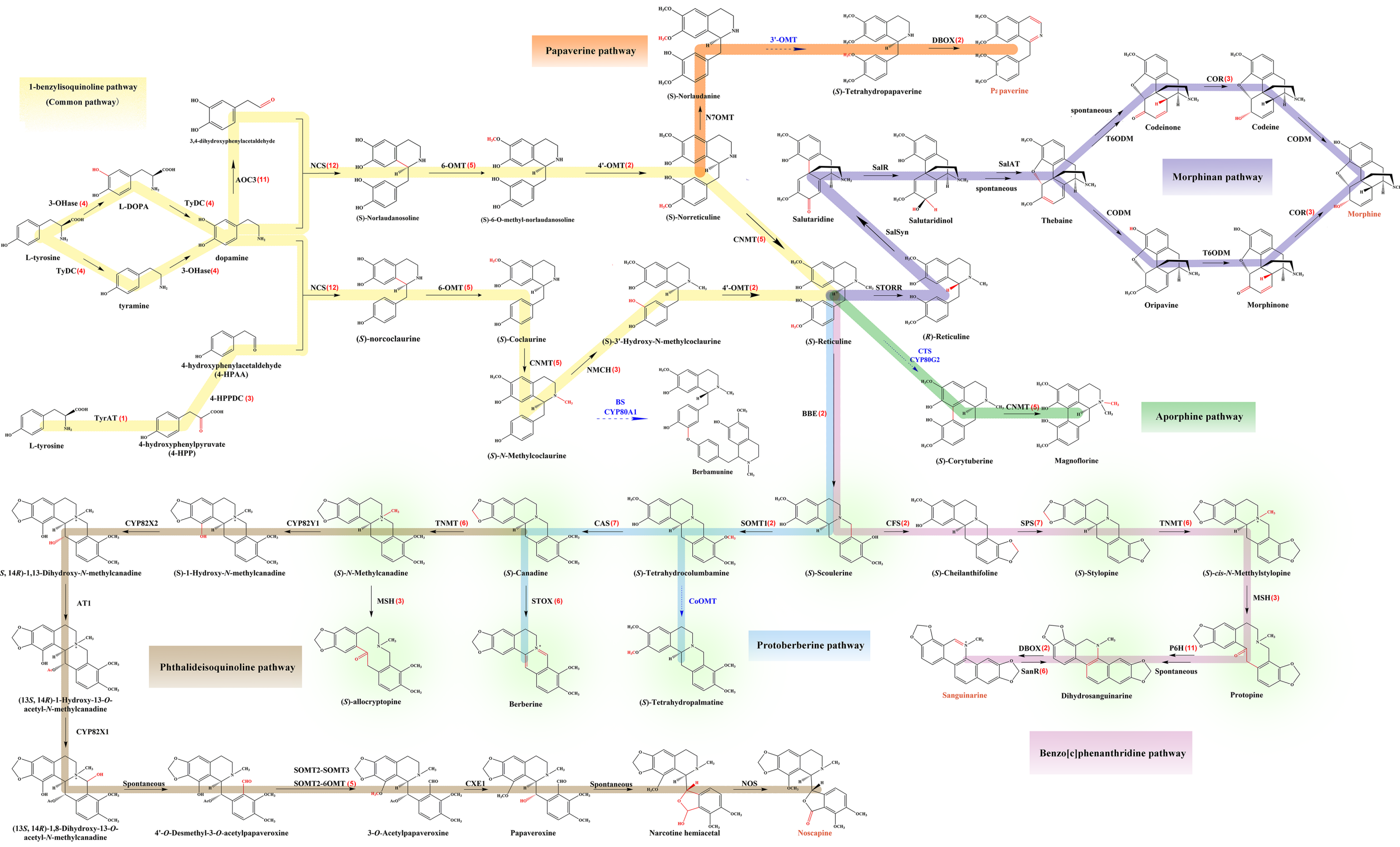

Supplement: Supplementary file 9 — Figure S7 [file 41438_2020_450_MOESM9_ESM.pdf]

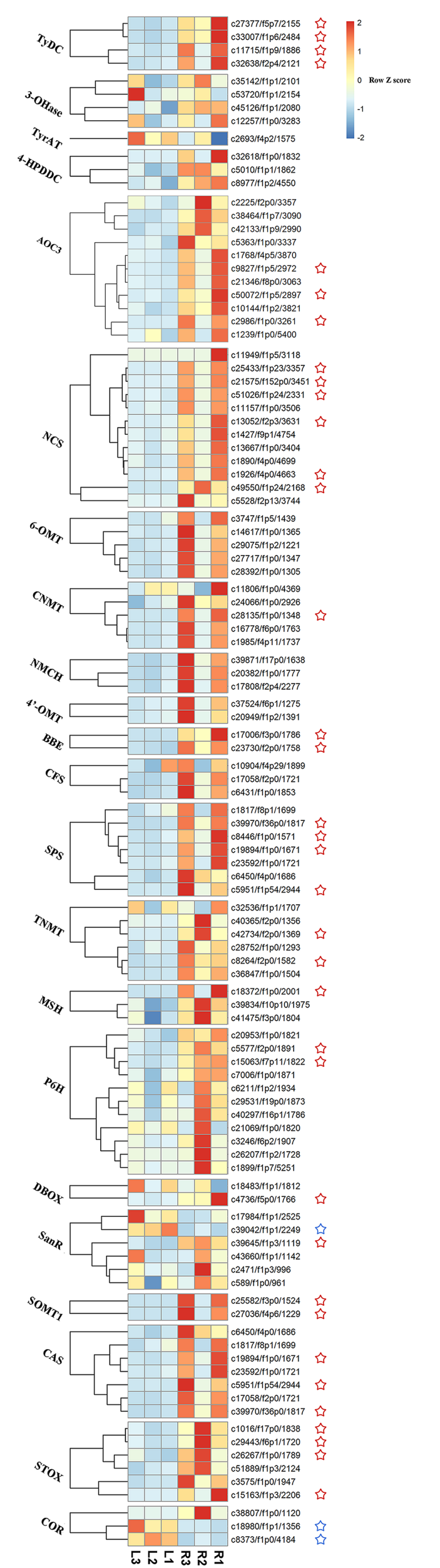

Supplement: Supplementary file 10 — Figure S8 [file 41438_2020_450_MOESM10_ESM.pdf]

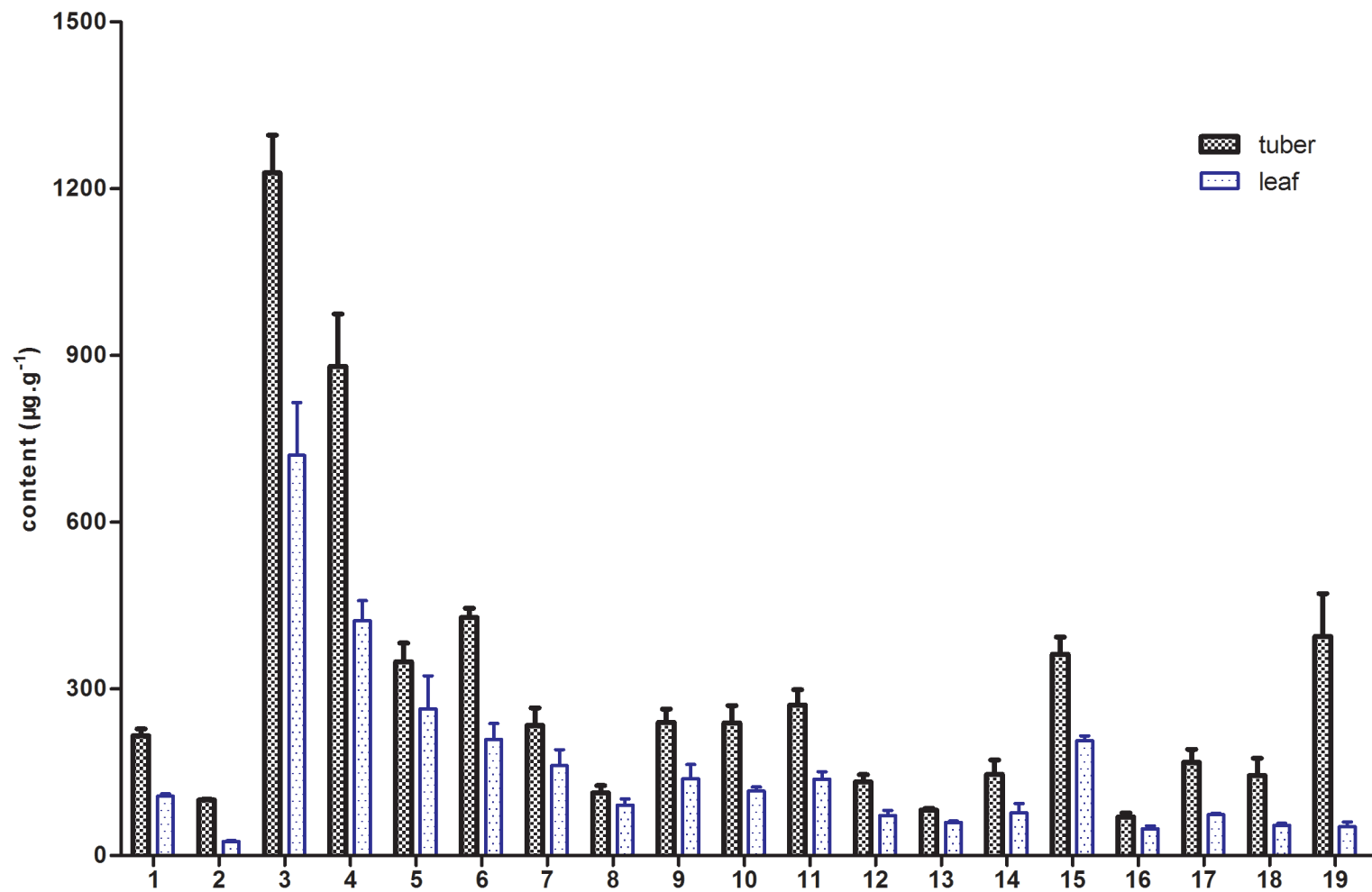

Supplement: Supplementary file 11 — Figure S9 [file 41438_2020_450_MOESM11_ESM.pdf]

## qRT-PCR

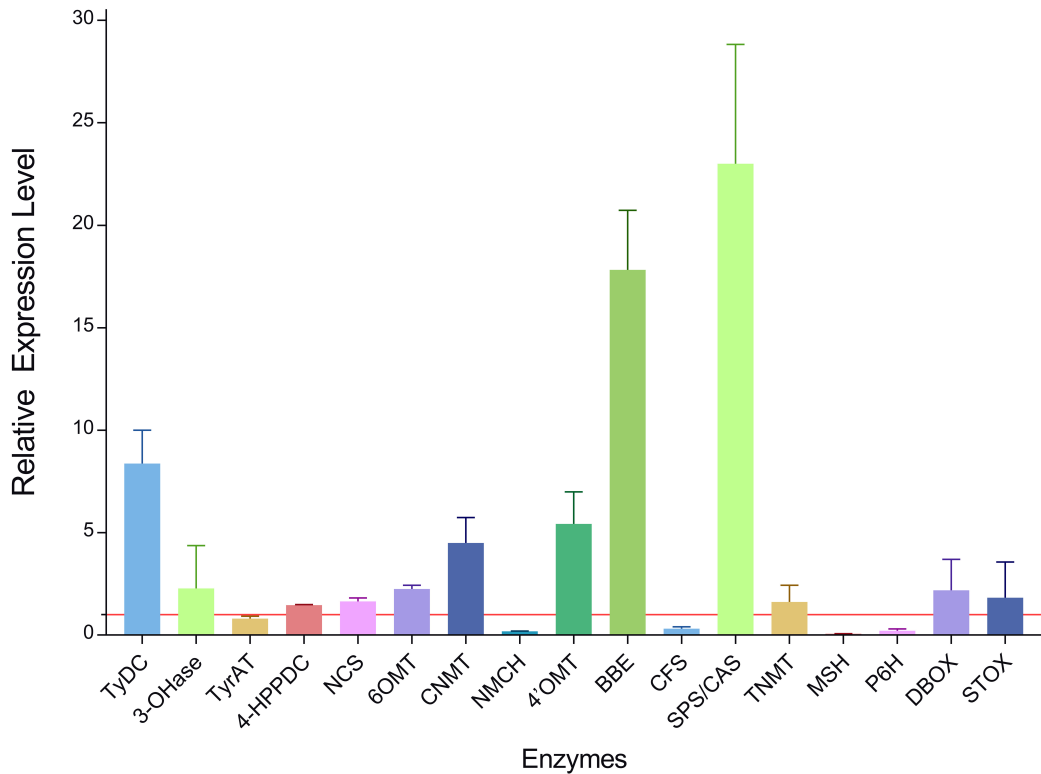

Supplement: Supplementary file 12 — Figure S10 [file 41438_2020_450_MOESM12_ESM.pdf]
