# Supplementary material for: Integration of full-length transcriptomics and targeted metabolomics to identify benzylisoquinoline alkaloid biosynthetic genes in Corydalis yanhusuo
Source: Hortic Res. 2021 Jan 10;8:16. doi: 10.1038/s41438-020-00450-6 (PMC7797006; doi:10.1038/s41438-020-00450-6)
Supplement: Supplementary file 5 — Figure S3 [file 41438_2020_450_MOESM5_ESM.pdf]

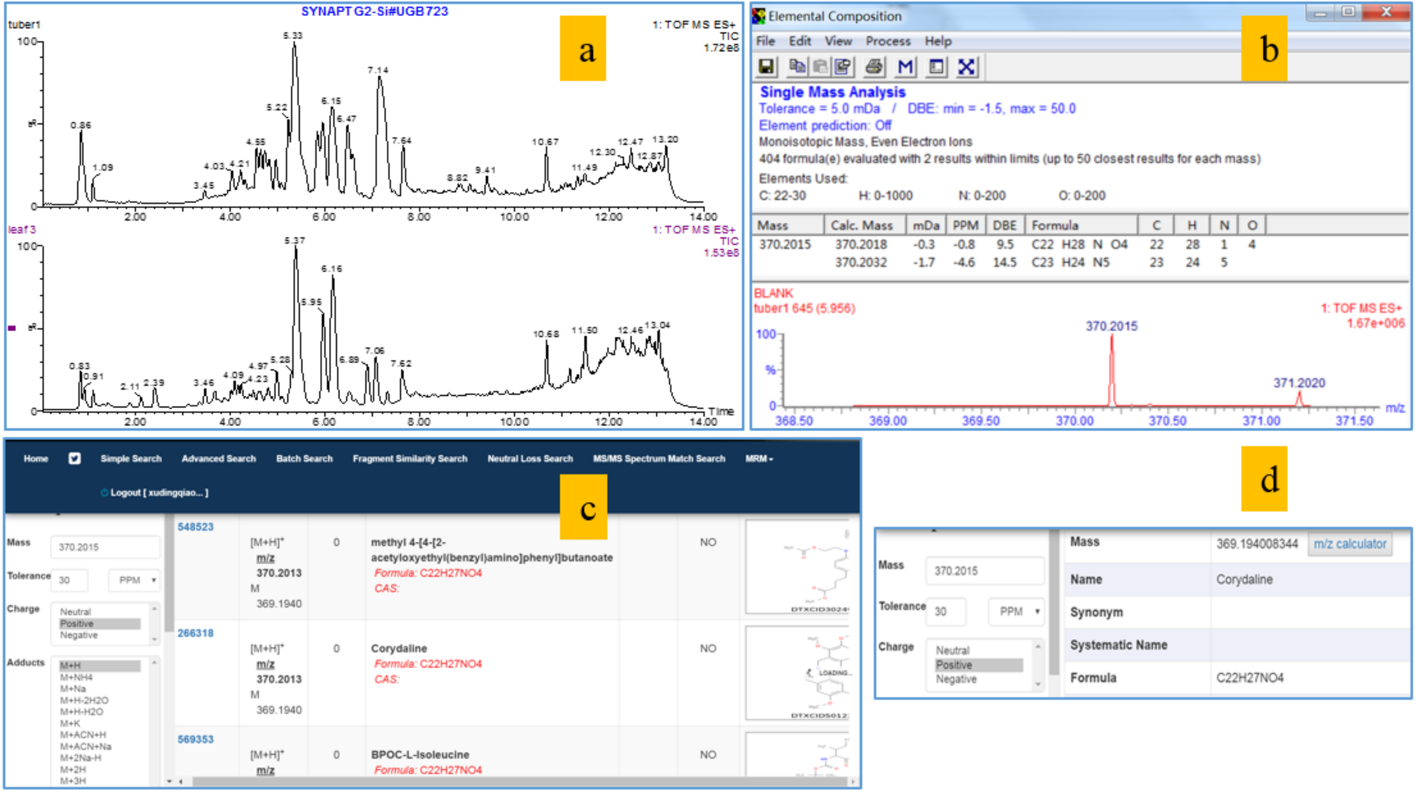

a
SYNAPT G2-Si#UGB723
tuber1
1: TOF MS ES+
TIC
1.72e8
0.86
1.09
3.45
4.03
4.21
4.55
5.22
5.33
6.15
6.47
7.14
7.64
8.82
9.41
10.67
11.49
12.30
12.47
12.87
13.20
leaf 3
1: TOF MS ES+
TIC
1.53e8
0.83
0.91
2.11
2.39
3.46
4.09
4.23
4.97
5.28
5.37
5.95
6.16
6.89
7.06
7.62
10.68
11.50
12.46
13.04
Time
2.00
4.00
6.00
8.00
10.00
12.00
14.00
b
Elemental Composition
File Edit View Process Help
Single Mass Analysis
Tolerance = 5.0 mDa / DBE: min = -1.5, max = 50.0
Element prediction: Off
Monoisotopic Mass, Even Electron Ions
404 formula(e) evaluated with 2 results within limits (up to 50 closest results for each mass)
Elements Used:
C: 22-30 H: 0-1000 N: 0-200 O: 0-200
Mass Calc. Mass mDa PPM DBE Formula C H N O
370.2015 370.2018 -0.3 -0.8 9.5 C22 H28 N O4 22 28 1 4
370.2032 -1.7 -4.6 14.5 C23 H24 N5 23 24 5
BLANK
tuber1 645 (5.956)
1: TOF MS ES+
1.67e+006
370.2015
371.2020
100
%
0
m/z
368.50
369.00
369.50
370.00
370.50
371.00
371.50
c
Home
Simple Search
Advanced Search
Batch Search
Fragment Similarity Search
Neutral Loss Search
MS/MS Spectrum Match Search
MRM
Logout [xudingqiao...]
Mass
370.2015
Tolerance
30
PPM
Charge
Neutral
Positive
Negative
Adducts
M+H
M+NH4
M+Na
M+H-2H2O
M+H-H2O
M+K
M+ACN+H
M+ACN+Na
M+2Na-H
M+2H
M+3H
548523
[M+H]+
m/z
370.2013
M
369.1940
0
methyl 4-[4-[2-acetyloxyethyl(benzyl)amino]phenyl]butanoate
Formula: C22H27NO4
CAS:
NO
266318
[M+H]+
m/z
370.2013
M
369.1940
0
Corydaline
Formula: C22H27NO4
CAS:
NO
LOADING...
569353
[M+H]+
m/z
0
BPOC-L-Isoleucine
Formula: C22H27NO4
NO
d
Mass
370.2015
Tolerance
30
PPM
Charge
Neutral
Positive
Negative
Mass 369.194008344
m/z calculator
Name Corydaline
Synonym
Systematic Name
Formula C22H27NO4
