# Supplementary material for: Integration of full-length transcriptomics and targeted metabolomics to identify benzylisoquinoline alkaloid biosynthetic genes in Corydalis yanhusuo
Source: Hortic Res. 2021 Jan 10;8:16. doi: 10.1038/s41438-020-00450-6 (PMC7797006; doi:10.1038/s41438-020-00450-6)
Supplement: Supplementary file 13 — Identifying candidate genes in Benzylisoquinoline biosynthetic pathway [file 41438_2020_450_MOESM13_ESM.pdf]

KEGG Pathway Number	Pathway Name	Reads Number
ko00232	Metabolism; Biosynthesis of other secondary metabolites; Caffeine metabolism	9
ko00254	Metabolism; Biosynthesis of other secondary metabolites; Aflatoxin biosynthesis	5
ko00261	Metabolism; Biosynthesis of other secondary metabolites; Monobactam biosynthesis	26
ko00311	Metabolism; Biosynthesis of other secondary metabolites; Penicillin and cephalosporin biosynthes	1
ko00401	Metabolism; Biosynthesis of other secondary metabolites; Novobiocin biosynthesis	5
ko00521	Metabolism; Biosynthesis of other secondary metabolites; Streptomycin biosynthesis	44
ko00524	Metabolism; Biosynthesis of other secondary metabolites; Neomycin, kanamycin and gentamicin bios	14
ko00901	Metabolism; Biosynthesis of other secondary metabolites; Indole alkaloid biosynthesis	23
ko00940	Metabolism; Biosynthesis of other secondary metabolites; Phenylpropanoid biosynthesis	242
ko00941	Metabolism; Biosynthesis of other secondary metabolites; Flavonoid biosynthesis	41
ko00943	Metabolism; Biosynthesis of other secondary metabolites; Isoflavonoid biosynthesis	2
ko00944	Metabolism; Biosynthesis of other secondary metabolites; Flavone and flavonol biosynthesis	6
ko00945	Metabolism; Biosynthesis of other secondary metabolites; Stilbenoid, diarylheptanoid and ginger	25
ko00950	Metabolism; Biosynthesis of other secondary metabolites; Isoquinoline alkaloid biosynthesis	134
ko00960	Metabolism; Biosynthesis of other secondary metabolites; Tropane, piperidine and pyridine alkal	69
ko00966	Metabolism; Biosynthesis of other secondary metabolites; Glucosinolate biosynthesis	6
