# Supplementary material for: Integration of full-length transcriptomics and targeted metabolomics to identify benzylisoquinoline alkaloid biosynthetic genes in Corydalis yanhusuo
Source: Hortic Res. 2021 Jan 10;8:16. doi: 10.1038/s41438-020-00450-6 (PMC7797006; doi:10.1038/s41438-020-00450-6)
Supplement: Supplementary file 14 — Filter result of 8,794 differential expressed genes [file 41438_2020_450_MOESM14_ESM.pdf]

Table S2. Filter result of 8,794 differential expressed genes.

Unigene ID	Root readcount	Leaf readcoun	log2FoldChange	P value	adjusted P value
i1_HQ_YHS_c39893/f20p5/1810	986.1691095	0	Inf	1.21E-40	2.93E-36
i2_LQ_YHS_c3628/f1p4/2394	0.359624837	921.1397855	-11.323	1.16E-40	2.93E-36
i1_LQ_YHS_c38850/f1p0/1333	0	699.8617724	-Inf	7.63E-40	1.23E-35
i2_LQ_YHS_c24739/f1p5/2646	1.263932271	1054.369449	-9.7042	1.31E-38	1.27E-34
i2_LQ_YHS_c27356/f1p33/2501	3120.838834	0	Inf	1.22E-38	1.27E-34
i1_LQ_YHS_c8155/f1p3/1942	1.983181944	2324.710154	-10.195	8.14E-37	6.56E-33
i1_HQ_YHS_c11101/f3p1/1912	1040.448534	2.123233398	8.9367	2.21E-35	1.53E-31
i1_LQ_YHS_c43833/f1p9/1042	2.538355225	2345.276661	-9.8516	2.86E-35	1.73E-31
i1_LQ_YHS_c28148/f1p0/1177	0.719249673	541.2208431	-9.5555	7.00E-35	3.76E-31
i0_LQ_YHS_c1800/f1p0/461	2.723412986	925.0306781	-8.4079	2.05E-34	9.91E-31
i4_HQ_YHS_c14833/f3p1/4448	3.23662353	826.9855543	-7.9972	3.09E-34	1.36E-30
i1_LQ_YHS_c38836/f1p0/1293	3.227396614	918.9474674	-8.1535	5.78E-34	2.33E-30
i1_LQ_YHS_c36873/f1p0/1944	0	400.6367691	-Inf	1.50E-33	5.59E-30
i2_LQ_YHS_c21385/f1p0/2283	1.34660235	565.542573	-8.7142	4.84E-33	1.67E-29
i2_LQ_YHS_c52078/f1p5/2524	0	355.5677044	-Inf	5.57E-32	1.79E-28
i1_LQ_YHS_c12183/f1p0/1400	2.168239704	678.0729286	-8.2888	6.53E-32	1.97E-28
i1_LQ_YHS_c33152/f1p2/2008	5.991508569	1873.975848	-8.289	8.46E-32	2.27E-28
i2_LQ_YHS_c40716/f1p1/2146	6.068301423	1262.554296	-7.7008	8.12E-32	2.27E-28
i1_LQ_YHS_c43676/f1p1/1049	1.978568486	537.2407735	-8.085	2.76E-31	7.03E-28
i1_LQ_YHS_c32742/f1p0/1635	2.569835652	554.2579964	-7.7527	5.07E-31	1.23E-27
i1_LQ_YHS_c3110/f1p3/2007	5.349051749	902.9014663	-7.3991	5.94E-31	1.37E-27
i2_HQ_YHS_c29685/f9p4/3004	6.393104516	1072.864116	-7.3907	1.11E-30	2.33E-27
i2_HQ_YHS_c32508/f2p4/2325	4.521537169	722.688786	-7.3204	1.09E-30	2.33E-27
i1_LQ_YHS_c19846/f1p0/1750	2.876998694	538.3418794	-7.5478	1.19E-30	2.40E-27
i2_LQ_YHS_c6807/f1p2/2819	1.876180805	509.4060761	-8.0849	1.26E-30	2.44E-27
i0_HQ_YHS_c402/f2p16/879	4.850953721	1248.402596	-8.0076	1.48E-30	2.75E-27
i1_HQ_YHS_c23969/f2p0/1544	3.268095583	622.4802183	-7.5734	2.00E-30	3.58E-27
i1_LQ_YHS_c19620/f1p24/1841	6.325538579	904.0427733	-7.1591	4.19E-30	7.23E-27
i1_HQ_YHS_c17036/f2p0/1651	3.540436881	624.4337828	-7.4625	4.62E-30	7.69E-27
i0_LQ_YHS_c3010/f1p2/955	7.471979026	1067.189802	-7.1581	5.99E-30	9.65E-27
i1_LQ_YHS_c6231/f1p1/1510	1.089365194	408.4481025	-8.5505	6.75E-30	1.05E-26
i1_LQ_YHS_c10092/f1p0/1482	7.192496734	812.8716524	-6.8204	7.48E-30	1.13E-26
i2_HQ_YHS_c56929/f7p6/2172	0	739.9950393	-Inf	1.27E-29	1.87E-26
i1_LQ_YHS_c8580/f2p8/1839	1.993672628	868.552833	-8.767	5.79E-29	8.23E-26
i1_LQ_YHS_c18921/f1p0/1448	3.529946197	535.185704	-7.2442	7.97E-29	1.10E-25
i2_LQ_YHS_c2939/f1p4/2198	1.634047791	407.9432066	-7.9638	1.21E-28	1.62E-25
i1_LQ_YHS_c10167/f1p5/1814	368.4143865	0.75225497	8.9359	1.88E-28	2.46E-25
i1_HQ_YHS_c588/f19p0/1875	3530.829994	0	Inf	2.48E-28	3.15E-25
i2_LQ_YHS_c34989/f1p16/2336	7.059892395	747.7911951	-6.7268	4.14E-28	5.13E-25
i1_LQ_YHS_c28299/f1p2/1065	5.776242523	665.5373134	-6.8482	4.95E-28	5.60E-25
i1_LQ_YHS_c36899/f1p2/1471	1562.020652	11.6488744	7.0671	4.76E-28	5.60E-25
i1_LQ_YHS_c5635/f1p0/1504	680.1740099	1.864503878	8.511	4.99E-28	5.60E-25
i2_LQ_YHS_c14983/f1p3/2076	1.90638909	409.4729099	-7.7468	4.95E-28	5.60E-25
i2_LQ_YHS_c39892/f1p0/3016	6.536191166	717.3048254	-6.778	9.01E-28	9.89E-25
i1_HQ_YHS_c2840/f4p0/1805	7.702349214	963.1405905	-6.9663	1.29E-27	1.39E-24
i0_LQ_YHS_c3127/f1p4/1021	5.446825971	614.0310899	-6.8168	1.50E-27	1.58E-24
i1_LQ_YHS_c7778/f1p11/1857	7.701543807	790.4980921	-6.6815	1.86E-27	1.91E-24
i2_LQ_YHS_c11673/f1p1/2710	2.965545999	447.180728	-7.2364	2.96E-27	2.98E-24
i1_HQ_YHS_c2729/f15p4/1929	6.242868499	605.4042727	-6.5995	3.94E-27	3.88E-24
i1_HQ_YHS_c25688/f2p0/1421	10.72162915	987.3582382	-6.525	4.64E-27	4.49E-24
i1_LQ_YHS_c38851/f1p0/1251	2.80020584	409.9074696	-7.1936	4.85E-27	4.60E-24
i1_LQ_YHS_c5254/f1p0/1487	10.13243953	830.8883745	-6.3576	1.06E-26	9.85E-24
i1_HQ_YHS_c2516/f4p0/1939	796.6088632	9.47327001	6.3939	1.31E-26	1.19E-23
i2_LQ_YHS_c27286/f1p8/2458	6.027602454	563.7341116	-6.5473	1.39E-26	1.25E-23
i1_LQ_YHS_c4696/f1p0/1428	6.962118172	604.7680032	-6.4407	1.88E-26	1.62E-23
i2_LQ_YHS_c19499/f1p0/2716	2.697818159	390.8458039	-7.1787	1.85E-26	1.62E-23
i1_LQ_YHS_c11638/f1p8/1968	4.532027853	488.2578319	-6.7513	2.01E-26	1.70E-23
i2_LQ_YHS_c49587/f1p0/2114	1.089365194	311.0338698	-8.1574	2.14E-26	1.78E-23
i2_LQ_YHS_c3006/f1p6/2363	9.855484776	3951.32332	-8.6472	2.33E-26	1.91E-23
i3_LQ_YHS_c4519/f1p5/3401	0.514474312	276.5757173	-9.0704	2.50E-26	2.01E-23
i1_HQ_YHS_c41526/f5p0/1879	15.86002831	1768.370183	-6.8009	2.91E-26	2.31E-23
i1_HQ_YHS_c39815/f13p3/1756	9.331783547	4027.382669	-8.7535	3.50E-26	2.73E-23
i1_HQ_YHS_c6798/f5p0/1952	14.94729937	1307.145629	-6.4504	3.82E-26	2.93E-23
i0_LQ_YHS_c1902/f1p0/779	13.44711968	3304.521708	-7.941	5.13E-26	3.87E-23
i1_LQ_YHS_c35660/f1p0/1776	16.05764593	1728.641039	-6.7502	6.73E-26	5.00E-23
i1_LQ_YHS_c31530/f2p0/1686	16.1239481	1418.598172	-6.4591	8.04E-26	5.89E-23
i0_LQ_YHS_c2345/f1p2/891	16.72236464	1481.057635	-6.4687	1.09E-25	7.85E-23
i3_LQ_YHS_c7380/f1p2/3270	3.257604899	389.3666933	-6.9012	1.32E-25	9.37E-23
i3_LQ_YHS_c3598/f1p3/3893	0	221.7068819	-Inf	1.61E-25	1.13E-22
i1_LQ_YHS_c14078/f1p0/1074	3.699899815	6545.433474	-10.789	1.65E-25	1.14E-22
i1_LQ_YHS_c21318/f1p105/1993	3.149339992	361.5026866	-6.8428	2.09E-25	1.42E-22
i2_LQ_YHS_c24226/f1p4/2134	11.2427861	792.0633867	-6.1385	2.60E-25	1.74E-22
i1_LQ_YHS_c11968/f1p2/1798	3.957136971	420.6888029	-6.7322	2.80E-25	1.86E-22
i5_LQ_YHS_c1819/f1p0/5809	1.361706493	300.0428225	-7.7836	2.97E-25	1.94E-22
i2_LQ_YHS_c36491/f1p9/2154	16.66321183	1397.09991	-6.3896	3.50E-25	2.25E-22
i3_LQ_YHS_c12353/f1p0/4022	11.31957895	760.1072205	-6.0693	4.10E-25	2.60E-22
i1_HQ_YHS_c2331/f4p4/1818	1038.487258	15.2025645	6.094	4.32E-25	2.71E-22
i1_LQ_YHS_c28721/f1p0/1070	0	661.7084519	-Inf	5.02E-25	3.11E-22
i2_LQ_YHS_c51411/f1p7/2583	1.316394066	297.1352203	-7.8184	5.08E-25	3.11E-22
i0_LQ_YHS_c2288/f1p1/810	3.442662659	1073.934741	-8.2852	8.41E-25	5.08E-22
i2_LQ_YHS_c52816/f1p6/2438	18.62621782	1331.391439	-6.1595	9.75E-25	5.81E-22
i1_LQ_YHS_c21672/f1p0/1931	1.361706493	287.1367494	-7.7202	1.23E-24	7.27E-22
i1_LQ_YHS_c9095/f1p1/1906	2.080956166	311.7464714	-7.227	1.43E-24	8.31E-22
i4_LQ_YHS_c4521/f1p2/4564	15.49198197	982.4110947	-5.9867	1.51E-24	8.67E-22
i1_LQ_YHS_c13005/f1p2/1829	17.29011452	980.9589546	-5.8262	2.04E-24	1.16E-21
i1_HQ_YHS_c10801/f2p1/1771	19.24354654	3019.853205	-7.294	2.98E-24	1.67E-21
i1_LQ_YHS_c5937/f1p0/1857	3.170321361	349.53896	-6.7847	3.61E-24	2.00E-21

i1_LQ_YHS_c4615/f1p0/1534	12.34391412	753.8663816	-5.9324	3.96E-24	2.18E-21
i1_HQ_YHS_c39890/f5p0/1627	23.15951781	2053.094497	-6.4701	5.46E-24	2.96E-21
i1_LQ_YHS_c13640/f1p3/1142	10.66916736	630.9658914	-5.886	6.09E-24	3.27E-21
i1_LQ_YHS_c10651/f2p0/1935	21.41718836	2209.161855	-6.6886	6.47E-24	3.44E-21
i1_LQ_YHS_c38523/f1p3/1250	22.19224952	2475.023186	-6.8012	7.46E-24	3.92E-21
i2_LQ_YHS_c41921/f1p21/2061	7.951631596	534.1408741	-6.0698	8.31E-24	4.32E-21
i1_LQ_YHS_c23053/f1p5/1810	3.083037822	337.8462085	-6.7759	9.62E-24	4.94E-21
i2_LQ_YHS_c21407/f1p2/2736	3.81277818	363.744048	-6.5759	1.40E-23	7.14E-21
i2_LQ_YHS_c14953/f1p18/2073	6.808532464	476.7035794	-6.1296	1.55E-23	7.81E-21
i1_LQ_YHS_c13698/f1p8/1322	22.82086597	2321.770526	-6.6687	1.57E-23	7.82E-21
i2_LQ_YHS_c17628/f8p9/2284	21.73323965	1492.759318	-6.1019	1.63E-23	8.03E-21
i2_LQ_YHS_c15302/f1p8/2444	21.73658934	1327.976377	-5.933	1.74E-23	8.48E-21
i2_LQ_YHS_c27348/f1p6/2157	14.48782276	827.3096823	-5.8355	1.80E-23	8.70E-21
i1_LQ_YHS_c10273/f1p0/1967	24.63283053	2226.434005	-6.498	2.66E-23	1.27E-20
i6_LQ_YHS_c627/f1p0/6591	0	188.486296	-Inf	2.90E-23	1.37E-20
i1_LQ_YHS_c27473/f1p0/1111	24.23504263	2552.197558	-6.7185	3.28E-23	1.54E-20
i2_LQ_YHS_c55229/f1p68/2176	24.78768	1819.125531	-6.1975	3.31E-23	1.54E-20
i2_LQ_YHS_c59591/f1p1/2038	4.131704048	348.613998	-6.3987	4.16E-23	1.91E-20
i0_LQ_YHS_c1964/f1p1/922	24.28369637	2947.723233	-6.9235	6.51E-23	2.97E-20
i1_LQ_YHS_c5628/f1p0/1883	15.75383258	2213.528488	-7.1345	6.74E-23	3.04E-20
i1_LQ_YHS_c20609/f1p0/1899	23.36555694	1260.138247	-5.7531	7.27E-23	3.21E-20
i1_LQ_YHS_c21668/f1p1/1914	13.04805965	743.1628955	-5.8318	7.32E-23	3.21E-20
i2_LQ_YHS_c33733/f1p1/2696	23.01308147	1285.005027	-5.8032	7.19E-23	3.21E-20
i1_LQ_YHS_c26277/f1p3/1562	1.876180805	271.1229247	-7.175	9.04E-23	3.92E-20
i2_LQ_YHS_c48836/f1p5/2405	9.111912418	496.1089275	-5.7668	9.09E-23	3.92E-20
i1_HQ_YHS_c2896/f7p2/1948	25.50230784	2516.067462	-6.6244	1.09E-22	4.67E-20
i2_LQ_YHS_c39571/f1p8/2856	26.5417639	1601.033671	-5.9146	1.15E-22	4.87E-20
i1_HQ_YHS_c27205/f2p0/1126	15.30692421	1272.452999	-6.3773	1.25E-22	5.26E-20
i1_LQ_YHS_c34704/f1p0/1496	0.272341299	196.662993	-9.4961	1.30E-22	5.43E-20
i1_LQ_YHS_c5366/f1p13/1478	17.3215782	847.9966373	-5.6134	1.36E-22	5.60E-20
i1_LQ_YHS_c24347/f1p0/1961	18.59600953	857.8180902	-5.5276	1.52E-22	6.19E-20
i2_HQ_YHS_c4242/f2p0/2410	11.60241093	590.9196782	-5.6705	1.53E-22	6.19E-20
i1_HQ_YHS_c21222/f4p0/1721	22.37776565	3502.660348	-7.2902	2.08E-22	8.37E-20
i1_LQ_YHS_c6855/f1p0/1793	2.625638763	283.451227	-6.7543	2.10E-22	8.39E-20
i3_LQ_YHS_c12873/f1p2/3188	26.41710596	1809.223581	-6.0978	2.21E-22	8.74E-20
i1_LQ_YHS_c27262/f2p0/1294	28.52955093	1977.706032	-6.1152	2.25E-22	8.85E-20
i1_LQ_YHS_c33450/f1p29/1928	6.808532464	427.2897891	-5.9717	2.47E-22	9.64E-20
i1_LQ_YHS_c11233/f1p1/1698	1245.68685	25.50154994	5.6102	2.62E-22	1.01E-19
i1_LQ_YHS_c32206/f1p0/1627	22.37269383	1072.140616	-5.5826	2.86E-22	1.10E-19
i0_LQ_YHS_c915/f1p0/838	24.96351085	1316.092542	-5.7203	3.02E-22	1.15E-19
i2_LQ_YHS_c33883/f1p6/2086	7.419517231	648.8835537	-6.4505	3.57E-22	1.35E-19
i1_LQ_YHS_c3887/f1p1/1873	19.42017442	1277.770709	-6.0399	3.64E-22	1.36E-19
i1_LQ_YHS_c13959/f1p0/1302	0.272341299	189.6901706	-9.444	4.28E-22	1.58E-19
i2_LQ_YHS_c38133/f1p4/2850	13.49830934	622.3893889	-5.527	4.26E-22	1.58E-19
i4_LQ_YHS_c4829/f1p0/4960	25.15572635	1180.803102	-5.5527	4.31E-22	1.58E-19
i1_LQ_YHS_c27096/f3p0/1119	13.38208966	1649.574481	-6.9456	4.60E-22	1.67E-19
i3_LQ_YHS_c7150/f1p2/3879	0.514474312	283.6897747	-9.107	5.16E-22	1.86E-19
i2_HQ_YHS_c60302/f3p0/2061	284.9659779	2.501592453	6.8318	5.99E-22	2.14E-19
i4_HQ_YHS_c2058/f3p1/4647	20.65597595	891.8986441	-5.4322	6.56E-22	2.33E-19
i1_LQ_YHS_c3267/f1p2/1717	20.28714095	803.3254009	-5.3073	7.08E-22	2.50E-19
i1_LQ_YHS_c10830/f1p3/1552	19.7542128	823.8707674	-5.3822	7.27E-22	2.54E-19
i1_LQ_YHS_c6496/f1p0/1752	2.338193322	261.63915	-6.806	7.52E-22	2.61E-19
i2_LQ_YHS_c25427/f1p6/2710	20.66646664	892.8626656	-5.4331	8.28E-22	2.86E-19
i1_LQ_YHS_c24383/f1p2/1983	27.15147653	3546.802948	-7.0293	8.64E-22	2.96E-19
i1_LQ_YHS_c43865/f1p0/1008	29.14975424	1481.732087	-5.6677	9.08E-22	3.09E-19
i2_LQ_YHS_c59863/f1p0/2062	31.03642573	2188.394917	-6.1398	9.53E-22	3.22E-19
i1_LQ_YHS_c11259/f1p0/1467	25.32948802	1145.709666	-5.4993	1.05E-21	3.52E-19
i1_LQ_YHS_c38039/f1p9/1351	31.35407945	2246.871074	-6.1631	1.17E-21	3.90E-19
i2_LQ_YHS_c37210/f1p3/2378	23.36889826	4302.265762	-7.5244	1.23E-21	4.07E-19
i2_LQ_YHS_c24778/f1p3/2162	30.44515856	1731.948288	-5.83	1.31E-21	4.31E-19
i1_LQ_YHS_c28807/f1p2/1334	17.42985148	716.2713335	-5.3609	1.54E-21	5.02E-19
i1_LQ_YHS_c31894/f1p0/1430	30.31383474	1587.885659	-5.711	1.58E-21	5.14E-19
i1_LQ_YHS_c28158/f1p0/1098	1.07887451	11366.99238	-13.363	1.70E-21	5.46E-19
i1_LQ_YHS_c35715/f1p1/1751	31.74517634	2375.291579	-6.2254	1.87E-21	5.98E-19
i1_LQ_YHS_c32675/f1p2/1908	20.07566621	921.2316363	-5.52	2.26E-21	7.17E-19
i2_LQ_YHS_c4479/f1p4/2229	913.6299452	10.7831116	6.4048	2.47E-21	7.82E-19
i3_LQ_YHS_c10503/f1p0/3365	33.73933245	2015.749019	-5.9007	2.59E-21	8.13E-19
i1_LQ_YHS_c14432/f1p0/1109	26.97690108	3916.145358	-7.1816	2.88E-21	8.98E-19
i1_LQ_YHS_c13773/f1p5/1176	2.178730389	248.1817401	-6.8318	3.05E-21	9.46E-19
i1_LQ_YHS_c4877/f1p0/1710	1.448990031	220.9889861	-7.2528	3.53E-21	1.09E-18
i0_HQ_YHS_c376/f2p0/1003	5.925214773	3052.857492	-9.0091	3.84E-21	1.17E-18
i2_LQ_YHS_c41508/f1p1/2895	219.7881403	1.061616699	7.6937	4.05E-21	1.23E-18
i1_LQ_YHS_c35573/f1p0/1549	22.93248057	4770.74945	-7.7007	4.40E-21	1.33E-18
i0_LQ_YHS_c910/f1p0/678	17.02364208	673.0839499	-5.3052	4.74E-21	1.42E-18
i4_LQ_YHS_c13565/f1p0/4120	8.341464717	419.7225684	-5.653	5.07E-21	1.51E-18
i2_LQ_YHS_c60586/f82p6/2549	21.7768132	872.3806501	-5.3241	5.21E-21	1.54E-18
i3_LQ_YHS_c7011/f1p51/3498	21.31066234	833.0437251	-5.2887	5.56E-21	1.64E-18
i2_HQ_YHS_c48467/f2p0/2451	3.81277818	292.4691269	-6.2613	5.79E-21	1.69E-18
i1_LQ_YHS_c24252/f1p0/1817	16.64764933	630.3304565	-5.2427	6.13E-21	1.78E-18
i2_HQ_YHS_c17757/f3p5/2229	19.33290763	684.8213784	-5.1466	6.26E-21	1.81E-18
i2_LQ_YHS_c61017/f14p2/2109	29.86532392	1274.971966	-5.4159	6.75E-21	1.94E-18
i3_HQ_YHS_c1787/f3p0/4038	9.078354442	473.5476386	-5.7049	7.52E-21	2.15E-18
i2_LQ_YHS_c36042/f1p3/2167	11.72705212	504.3907653	-5.4266	7.86E-21	2.23E-18
i1_HQ_YHS_c2594/f2p0/1437	31.49381641	1501.963862	-5.5756	8.43E-21	2.38E-18
i2_HQ_YHS_c1782/f12p5/2575	28.42221949	1100.589229	-5.2751	8.62E-21	2.42E-18
i0_LQ_YHS_c1025/f1p6/817	36.40980854	2339.997134	-6.006	9.26E-21	2.59E-18
i1_LQ_YHS_c4456/f1p0/1650	1.634047791	11106.30664	-12.731	1.04E-20	2.89E-18
i1_LQ_YHS_c20522/f1p1/1911	480.9414314	2.964509375	7.3419	1.12E-20	3.08E-18
i2_LQ_YHS_c40194/f1p4/2248	30.35787502	1256.780126	-5.3715	1.12E-20	3.08E-18

i1_LQ_YHS_c29061/f1p0/1225	30.52910078	1262.885923	-5.3704	1.14E-20	3.09E-18
i2_LQ_YHS_c8671/f3p10/2489	8.693940186	404.9848596	-5.5417	1.14E-20	3.09E-18
i1_LQ_YHS_c7420/f1p0/1605	33.7434708	1404.355202	-5.3792	1.32E-20	3.57E-18
i1_LQ_YHS_c27510/f1p0/1257	0.631966135	179.95446	-8.1536	1.35E-20	3.61E-18
i1_LQ_YHS_c34818/f1p1/1992	7.195024269	562.1610405	-6.2878	1.37E-20	3.65E-18
i3_LQ_YHS_c19082/f1p0/3562	7919.187728	9.652497578	9.6802	1.38E-20	3.65E-18
i1_HQ_YHS_c40079/f3p1/1689	22.68112064	1360.068906	-5.906	1.40E-20	3.70E-18
i2_LQ_YHS_c21146/f1p0/2809	0.257237156	241.2215923	-9.873	1.46E-20	3.82E-18
i2_HQ_YHS_c19345/f4p1/2187	17.1075843	633.1922813	-5.2099	1.52E-20	3.98E-18
i1_LQ_YHS_c24466/f1p5/1959	413.9704457	8.709364406	5.5708	1.88E-20	4.88E-18
i1_LQ_YHS_c14632/f1p1/1132	38.89744922	1889.835045	-5.6024	1.90E-20	4.90E-18
i4_HQ_YHS_c2523/f2p1/4935	19.28042909	718.6695634	-5.2201	2.16E-20	5.55E-18
i3_LQ_YHS_c8580/f1p8/3554	13.70021012	525.2620802	-5.2608	2.36E-20	6.03E-18
i1_LQ_YHS_c9807/f1p1/1480	8.987262853	418.2650934	-5.5404	2.38E-20	6.05E-18
i1_LQ_YHS_c14709/f1p0/1221	16.12649239	564.8672538	-5.1304	3.01E-20	7.62E-18
i4_LQ_YHS_c4350/f1p5/4536	34.18575735	1422.257613	-5.3786	3.19E-20	8.03E-18
i0_LQ_YHS_c1747/f1p0/962	900.2035688	26.03133261	5.1119	3.38E-20	8.47E-18
i1_LQ_YHS_c43768/f1p0/1009	33.19416637	3804.490829	-6.8406	3.56E-20	8.86E-18
i2_HQ_YHS_c48369/f2p8/2164	39.06405314	1791.035982	-5.5188	4.02E-20	9.97E-18
i1_LQ_YHS_c28333/f1p0/1147	22.81164743	1582.784493	-6.1166	4.17E-20	1.03E-17
i2_LQ_YHS_c37733/f1p6/2684	21.54644301	750.6570161	-5.1226	4.31E-20	1.06E-17
i2_LQ_YHS_c44689/f1p3/2065	19.47725805	649.4006606	-5.0592	5.03E-20	1.23E-17
i1_LQ_YHS_c13825/f1p0/1387	29.25214192	1000.642583	-5.0962	5.18E-20	1.26E-17
i0_LQ_YHS_c2790/f1p4/978	38.13828086	1808.628597	-5.5675	5.29E-20	1.28E-17
i2_LQ_YHS_c59479/f1p2/2036	36.67673097	1514.897972	-5.3682	5.34E-20	1.28E-17
i2_LQ_YHS_c40677/f1p1/2187	4.641564902	294.1968947	-5.986	5.46E-20	1.31E-17
i6_LQ_YHS_c495/f1p0/6730	22.13609937	1886.840065	-6.4134	7.96E-20	1.90E-17
i1_LQ_YHS_c20607/f1p0/1702	37.80298708	1505.749628	-5.3158	9.80E-20	2.31E-17
i1_LQ_YHS_c8736/f1p0/1439	33.6701557	1158.624664	-5.1048	9.75E-20	2.31E-17
i2_HQ_YHS_c17397/f3p7/2697	3354.234221	41.27339124	6.3446	1.11E-19	2.60E-17
i1_LQ_YHS_c33302/f1p3/1879	24.87623568	772.6391141	-4.957	1.22E-19	2.84E-17
i2_LQ_YHS_c9406/f1p0/2101	38.36117138	1494.948665	-5.2843	1.35E-19	3.13E-17
i1_LQ_YHS_c20464/f1p0/1824	36.94652798	1428.448924	-5.2729	1.41E-19	3.26E-17
i2_HQ_YHS_c17503/f3p4/2559	8.714921554	1428.587642	-7.3569	1.83E-19	4.20E-17
i1_LQ_YHS_c11320/f1p0/1602	576.5515458	7.728988443	6.221	1.96E-19	4.50E-17
i0_LQ_YHS_c2827/f1p2/536	24.11421787	716.7100404	-4.8934	2.07E-19	4.73E-17
i2_LQ_YHS_c49755/f1p8/2972	2.375550975	222.4685757	-6.5492	2.26E-19	5.13E-17
i4_LQ_YHS_c6493/f1p19/4305	44.1239122	2305.900684	-5.7076	2.29E-19	5.18E-17
i0_LQ_YHS_c3357/f1p0/326	16.55113888	1005.336202	-5.9246	2.58E-19	5.79E-17
i1_LQ_YHS_c38462/f1p0/1123	35.92935893	4598.078267	-6.9997	2.65E-19	5.93E-17
i4_LQ_YHS_c14092/f1p0/4191	37.91840973	1294.194341	-5.093	2.66E-19	5.93E-17
i2_LQ_YHS_c52570/f1p1/2363	44.290558	1487.516561	-5.0698	2.82E-19	6.26E-17
i0_LQ_YHS_c331/f2p0/579	25.2601832	1520.505178	-5.9115	3.06E-19	6.75E-17
i1_HQ_YHS_c9791/f4p0/1576	29.19854442	876.1786134	-4.9073	3.33E-19	7.31E-17
i0_LQ_YHS_c839/f1p4/657	11.1508891	892.2370751	-6.3222	3.66E-19	7.99E-17
i1_HQ_YHS_c31382/f4p0/1608	43.62455041	1681.521516	-5.2685	3.79E-19	8.25E-17
i1_LQ_YHS_c34261/f1p6/1835	24.10326045	832.5201921	-5.1102	3.99E-19	8.62E-17
i2_HQ_YHS_c17429/f2p2/2177	19.14068375	605.7000054	-4.9839	3.99E-19	8.62E-17
i0_LQ_YHS_c2881/f1p0/928	36.76783093	1153.236597	-4.9711	4.42E-19	9.50E-17
i0_LQ_YHS_c3713/f1p2/836	0	136.748474	-Inf	4.48E-19	9.58E-17
i1_LQ_YHS_c5725/f1p1/1998	3.976854572	243.2201007	-5.9345	5.19E-19	1.10E-16
i1_LQ_YHS_c13811/f1p24/1122	1246.847546	39.60355571	4.9765	5.49E-19	1.16E-16
i3_HQ_YHS_c1112/f8p0/3350	10.34896935	398.5403541	-5.2672	5.81E-19	1.23E-16
i0_LQ_YHS_c803/f1p0/549	41.79749845	1350.68664	-5.0141	5.90E-19	1.24E-16
i2_LQ_YHS_c25414/f1p2/2503	1391.369659	41.55775359	5.0652	6.07E-19	1.27E-16
i2_LQ_YHS_c12641/f1p2/2156	24.3811403	711.2345333	-4.8665	6.27E-19	1.31E-16
i1_LQ_YHS_c24373/f1p0/1903	47.0953438	1986.480183	-5.3985	6.52E-19	1.35E-16
i2_LQ_YHS_c49244/f1p3/2315	34.89117502	1054.586817	-4.9177	7.17E-19	1.48E-16
i2_LQ_YHS_c21251/f1p3/2174	33.80099604	1067.585893	-4.9811	8.15E-19	1.68E-16
i2_HQ_YHS_c17875/f2p4/2355	44.9015009	1753.775793	-5.2876	8.82E-19	1.81E-16
i3_LQ_YHS_c19506/f1p6/3787	17.99090199	556.0503451	-4.9499	9.77E-19	1.99E-16
i1_LQ_YHS_c8320/f1p0/1875	9.053565023	347.6918345	-5.2632	1.01E-18	2.05E-16
i1_LQ_YHS_c21731/f1p0/1595	29.33828975	823.7650003	-4.8114	1.02E-18	2.07E-16
i2_HQ_YHS_c37438/f2p0/2337	2.88287592	218.060613	-6.2411	1.07E-18	2.15E-16
i1_LQ_YHS_c6470/f1p2/1945	50.08776514	2784.9245	-5.797	1.09E-18	2.19E-16
i1_LQ_YHS_c35312/f1p2/1664	26.80615042	751.1544427	-4.8085	1.12E-18	2.24E-16
i1_HQ_YHS_c2630/f7p0/1936	42.03246535	1373.658609	-5.0304	1.17E-18	2.33E-16
i1_LQ_YHS_c38216/f1p2/1275	45.36098588	1516.816837	-5.0635	1.21E-18	2.39E-16
i1_LQ_YHS_c6436/f1p1/1933	6.952891256	311.2812636	-5.4845	1.27E-18	2.51E-16
i1_LQ_YHS_c9690/f1p0/1583	7.897897659	334.4158974	-5.404	1.31E-18	2.57E-16
i4_LQ_YHS_c11837/f1p0/4878	20.48776121	586.9533429	-4.8404	1.40E-18	2.73E-16
i1_LQ_YHS_c14012/f1p1/1217	10.44213848	371.1833068	-5.1516	1.52E-18	2.95E-16
i3_LQ_YHS_c14711/f1p1/3022	14.29435187	569.4229588	-5.316	1.52E-18	2.96E-16
i2_LQ_YHS_c36497/f1p0/2335	43.65855837	4032.934006	-6.5294	1.53E-18	2.96E-16
i2_LQ_YHS_c45249/f1p2/2282	849.3368687	0	Inf	1.55E-18	2.99E-16
i1_LQ_YHS_c10165/f1p0/1863	2.080956166	658.0430703	-8.3048	1.87E-18	3.58E-16
i1_HQ_YHS_c8279/f4p0/1500	40.21716785	2320.523944	-5.8505	1.88E-18	3.58E-16
i2_LQ_YHS_c60804/f7p0/2168	23.74155806	865.4297784	-5.1879	1.95E-18	3.71E-16
i2_LQ_YHS_c13743/f1p0/2751	23.42135168	675.1836425	-4.8494	2.02E-18	3.82E-16
i2_LQ_YHS_c19599/f1p12/2526	10.76440567	369.3979049	-5.1008	2.09E-18	3.93E-16
i4_LQ_YHS_c9551/f1p0/4832	50.80446216	2445.122235	-5.5888	2.09E-18	3.93E-16
i5_LQ_YHS_c3169/f1p1/5147	0	128.4359535	-Inf	2.20E-18	4.13E-16
i1_LQ_YHS_c3388/f1p1/1756	10.6981035	783.51234	-6.1945	2.42E-18	4.52E-16
i2_LQ_YHS_c18537/f1p4/2139	51.32229454	3320.807652	-6.0158	2.45E-18	4.56E-16
i2_LQ_YHS_c25388/f1p0/2250	8.442580256	336.1396823	-5.3152	2.70E-18	5.01E-16
i2_LQ_YHS_c59170/f1p3/2055	672.3200281	25.40972436	4.7257	3.05E-18	5.62E-16
i1_LQ_YHS_c38760/f1p1/1106	52.86951715	2303.964696	-5.4455	3.13E-18	5.76E-16
i2_HQ_YHS_c17706/f4p2/2362	37.92049565	1412.50019	-5.2191	3.16E-18	5.78E-16
i1_LQ_YHS_c18066/f1p0/1479	12.5940019	2805.280798	-7.7993	3.20E-18	5.83E-16

i2_HQ_YHS_c60650/f2p6/2042	26.12966052	6606.758005	-7.9821	3.27E-18	5.95E-16
i2_LQ_YHS_c52204/f1p0/2497	5.240786843	253.340292	-5.5951	3.34E-18	6.03E-16
i6_LQ_YHS_c250/f1p0/6306	0	208.867557	-Inf	3.35E-18	6.03E-16
i2_LQ_YHS_c43816/f1p4/2024	4.423762947	233.5020438	-5.722	3.47E-18	6.24E-16
i3_LQ_YHS_c18971/f1p0/3256	13.11770313	405.0784575	-4.9486	3.57E-18	6.39E-16
i1_LQ_YHS_c4326/f1p0/1515	8.568493588	330.2772574	-5.2685	3.77E-18	6.72E-16
i2_LQ_YHS_c26723/f1p0/2902	14.13153924	446.3373122	-4.9811	3.99E-18	7.10E-16
i1_LQ_YHS_c33616/f1p3/1954	15.53395308	457.0248092	-4.8788	4.10E-18	7.23E-16
i4_HQ_YHS_c1694/f5p0/4608	52.89718952	1956.376468	-5.2088	4.10E-18	7.23E-16
i2_HQ_YHS_c45281/f20p6/2660	29.2773897	810.7945639	-4.7915	4.13E-18	7.25E-16
i1_LQ_YHS_c32267/f1p37/1488	21.0101903	558.5770951	-4.7326	4.20E-18	7.35E-16
i2_LQ_YHS_c51003/f1p12/2691	15.24062204	464.4020185	-4.9294	4.27E-18	7.44E-16
i3_LQ_YHS_c14196/f1p0/3106	49.06260782	1758.550768	-5.1636	4.73E-18	8.22E-16
i1_LQ_YHS_c36824/f1p1/1812	3.715003958	279.5015995	-6.2333	4.79E-18	8.29E-16
i1_LQ_YHS_c13662/f1p0/1314	54.0432913	2360.46352	-5.4488	4.93E-18	8.51E-16
i1_LQ_YHS_c38276/f1p0/1083	53.65299144	2127.538245	-5.3094	4.96E-18	8.53E-16
i2_LQ_YHS_c4001/f1p6/2215	50.81955793	1856.201044	-5.1908	5.14E-18	8.81E-16
i4_LQ_YHS_c3836/f1p6/4407	0.544682597	384.6462506	-9.4639	5.42E-18	9.25E-16
i1_LQ_YHS_c12679/f1p0/1831	13.08875862	411.8156182	-4.9756	5.48E-18	9.29E-16
i5_LQ_YHS_c3551/f1p9/5645	17.61283169	509.7091952	-4.855	5.47E-18	9.29E-16
i1_LQ_YHS_c9825/f1p0/1907	45.38243398	3178.298487	-6.13	5.74E-18	9.67E-16
i2_LQ_YHS_c18686/f1p8/2424	53.29004204	1718.028005	-5.0107	5.73E-18	9.67E-16
i1_HQ_YHS_c6582/f2p0/1850	55.3864159	2798.973044	-5.6592	6.10E-18	1.02E-15
i1_LQ_YHS_c13054/f1p3/1753	44.60102886	1293.121264	-4.8576	6.13E-18	1.03E-15
i1_LQ_YHS_c43935/f1p0/1020	10516.37472	8.835115645	10.217	6.53E-18	1.09E-15
i1_LQ_YHS_c13568/f1p7/1335	9.696021842	1161.465556	-6.9043	6.66E-18	1.11E-15
i4_LQ_YHS_c5589/f1p9/4544	33.66332826	892.9219412	-4.7293	6.85E-18	1.13E-15
i3_LQ_YHS_c9102/f1p0/4000	17.07276256	486.8339232	-4.8337	6.92E-18	1.14E-15
i1_HQ_YHS_c41502/f4p1/1543	55.53538815	3111.936315	-5.8083	7.66E-18	1.25E-15
i1_LQ_YHS_c14050/f1p0/1206	0	127.1037974	-Inf	7.65E-18	1.25E-15
i2_LQ_YHS_c18362/f1p3/2186	17.85877276	508.6041003	-4.8318	7.83E-18	1.28E-15
i3_LQ_YHS_c3232/f1p29/3474	15.56796941	447.3468776	-4.8447	8.00E-18	1.30E-15
i1_LQ_YHS_c10526/f1p0/1882	0	172.3247588	-Inf	8.21E-18	1.33E-15
i2_LQ_YHS_c20824/f1p9/2248	55.93664542	3021.668955	-5.7554	8.45E-18	1.37E-15
i2_HQ_YHS_c18918/f3p3/2420	10.22814458	2107.155477	-7.6866	9.04E-18	1.46E-15
i2_LQ_YHS_c51323/f1p3/2758	11.57347479	370.3580979	-5	9.69E-18	1.56E-15
i2_HQ_YHS_c19637/f3p5/2513	45.46095734	1277.67044	-4.8127	1.07E-17	1.72E-15
i4_LQ_YHS_c9187/f1p0/4527	41.58221566	1122.857926	-4.7551	1.08E-17	1.72E-15
i1_LQ_YHS_c43982/f1p0/1042	0.817023896	176.034101	-7.7513	1.10E-17	1.74E-15
i5_LQ_YHS_c2136/f1p1/5639	47.85863376	1364.686476	-4.8336	1.12E-17	1.77E-15
i0_HQ_YHS_c244/f4p0/641	58.53036215	3485.858577	-5.8962	1.19E-17	1.87E-15
i2_HQ_YHS_c27913/f2p2/2072	42.73214224	1081.621078	-4.6617	1.19E-17	1.87E-15
i1_LQ_YHS_c33588/f1p1/1860	39.57677183	1055.017115	-4.7365	1.21E-17	1.90E-15
i2_LQ_YHS_c18960/f1p4/1967	29.57821715	714.9887509	-4.5953	1.22E-17	1.90E-15
i1_LQ_YHS_c10870/f1p3/1832	21.90941754	547.1247883	-4.6422	1.26E-17	1.96E-15
i1_LQ_YHS_c3584/f1p0/1559	37.36116727	904.0198324	-4.5967	1.28E-17	1.98E-15
i2_LQ_YHS_c4430/f1p2/2445	0.514474312	141.7017392	-8.1055	1.33E-17	2.06E-15
i1_LQ_YHS_c8245/f1p3/1976	39.94369922	992.6015156	-4.6352	1.36E-17	2.10E-15
i2_LQ_YHS_c7838/f1p1/2140	167.8312575	1.112248908	7.2374	1.38E-17	2.12E-15
i3_LQ_YHS_c10187/f1p3/3519	9.476142339	329.6678519	-5.1206	1.38E-17	2.12E-15
i4_LQ_YHS_c5272/f1p7/4717	133.6932815	0	Inf	1.40E-17	2.13E-15
i1_LQ_YHS_c12549/f1p3/1820	51.01942303	1317.634782	-4.6908	1.48E-17	2.26E-15
i2_LQ_YHS_c64564/f1p3/2013	6.263849867	465.7679449	-6.2164	1.50E-17	2.27E-15
i1_HQ_YHS_c11215/f2p0/1995	41.5696558	1046.133187	-4.6534	1.56E-17	2.37E-15
i0_LQ_YHS_c1459/f1p4/591	30.37297917	747.305529	-4.6208	1.62E-17	2.44E-15
i2_LQ_YHS_c42174/f1p6/2258	49.46835887	1353.653847	-4.7742	1.62E-17	2.44E-15
i3_LQ_YHS_c4379/f1p2/3375	54.64282679	1866.64964	-5.0943	1.66E-17	2.48E-15
i2_HQ_YHS_c60205/f11p6/2168	34.24156883	6466.277078	-7.561	1.73E-17	2.58E-15
i2_LQ_YHS_c14630/f1p67/2071	18.5192083	1576.442068	-6.4115	1.80E-17	2.68E-15
i2_LQ_YHS_c40698/f1p3/2572	9.304924953	304.7482952	-5.0335	1.81E-17	2.69E-15
i2_LQ_YHS_c50250/f1p4/2693	47.05131189	1197.77774	-4.67	1.81E-17	2.69E-15
i2_LQ_YHS_c20395/f1p8/2980	30.87822656	764.1787613	-4.6292	1.87E-17	2.76E-15
i2_LQ_YHS_c26262/f1p0/2551	0	144.569761	-Inf	2.00E-17	2.94E-15
i1_LQ_YHS_c20573/f1p3/1455	38.6842356	960.033853	-4.6333	2.07E-17	3.04E-15
i3_LQ_YHS_c3073/f1p3/3270	52.76077713	1539.845335	-4.8672	2.14E-17	3.13E-15
i0_LQ_YHS_c1650/f1p8/386	2.897980062	197.2616137	-6.0889	2.18E-17	3.19E-15
i1_LQ_YHS_c36901/f1p13/1902	0	117.6189163	-Inf	2.29E-17	3.33E-15
i2_LQ_YHS_c50372/f1p6/2333	60.25039621	2952.800203	-5.615	2.35E-17	3.41E-15
i2_LQ_YHS_c38056/f1p3/2096	36.09214644	872.9578665	-4.5962	2.39E-17	3.46E-15
i1_LQ_YHS_c6137/f1p1/1916	10.40016737	332.1596846	-4.9972	2.71E-17	3.91E-15
i3_LQ_YHS_c10324/f1p0/3790	8.714921554	310.1904634	-5.1535	2.80E-17	4.03E-15
i1_LQ_YHS_c6066/f1p2/1506	12.75139566	455.2155588	-5.1578	2.91E-17	4.17E-15
i2_LQ_YHS_c49463/f1p1/2508	11.11480359	348.2023814	-4.9694	2.95E-17	4.22E-15
i0_HQ_YHS_c379/f2p0/905	51.19684794	1302.816946	-4.6694	2.98E-17	4.24E-15
i0_LQ_YHS_c1836/f1p1/728	18.10251659	463.4222331	-4.6781	3.33E-17	4.73E-15
i2_LQ_YHS_c20610/f1p2/2129	17.58437066	443.7577637	-4.6574	3.53E-17	5.00E-15
i1_HQ_YHS_c4310/f4p0/1871	60.06581356	1919.841746	-4.9983	3.56E-17	5.03E-15
i2_LQ_YHS_c11852/f1p6/2257	23.52248396	548.380284	-4.5431	3.58E-17	5.04E-15
i3_LQ_YHS_c9209/f1p5/3262	5.705335269	520.8959911	-6.5125	3.67E-17	5.15E-15
i2_HQ_YHS_c32659/f3p21/2104	44.90484221	1126.634419	-4.649	3.78E-17	5.26E-15
i2_LQ_YHS_c38512/f1p4/2467	1.263932271	152.4548758	-6.9143	3.76E-17	5.26E-15
i2_LQ_YHS_c61000/f22p9/2189	56.26942841	2937.640539	-5.7062	3.77E-17	5.26E-15
i2_HQ_YHS_c1913/f5p2/2491	919.289511	32.06825004	4.8413	3.99E-17	5.54E-15
i1_LQ_YHS_c11472/f1p4/1475	50.38823718	1296.687397	-4.6856	4.08E-17	5.65E-15
i2_LQ_YHS_c33812/f1p15/2535	55.72553447	1537.774856	-4.7864	4.16E-17	5.74E-15
i1_HQ_YHS_c2837/f3p4/1463	61.09730647	1941.896613	-4.9902	4.21E-17	5.79E-15
i3_LQ_YHS_c16694/f1p0/3665	954.5771552	42.48516578	4.4898	4.26E-17	5.84E-15
i2_LQ_YHS_c40278/f1p28/2786	59.20726854	2046.572707	-5.1113	4.42E-17	6.05E-15
i6_LQ_YHS_c896/f1p0/6635	0	113.8838264	-Inf	4.77E-17	6.51E-15

i2_HQ_YHS_c32496/f2p0/2381	5.261768211	239.490421	-5.5083	5.07E-17	6.90E-15
i3_LQ_YHS_c4390/f1p9/3756	14.93345899	448.4807643	-4.9084	5.51E-17	7.48E-15
i1_LQ_YHS_c19716/f1p56/1800	1.490961142	853.7972622	-9.1615	5.58E-17	7.55E-15
i3_LQ_YHS_c11081/f1p1/3941	31.1438936	695.2891191	-4.4806	5.82E-17	7.86E-15
i2_LQ_YHS_c52941/f1p5/2132	24.06810841	533.1571008	-4.4694	6.02E-17	8.10E-15
i2_LQ_YHS_c64742/f1p4/2056	0.544682597	133.3752366	-7.9359	6.23E-17	8.36E-15
i2_LQ_YHS_c44998/f1p12/2073	53.8711237	4678.979231	-6.4405	6.25E-17	8.37E-15
i3_HQ_YHS_c996/f7p0/3387	26.0692607	575.1806945	-4.4636	6.55E-17	8.75E-15
i3_LQ_YHS_c4031/f1p0/3609	3.268095583	199.5897866	-5.9324	6.60E-17	8.78E-15
i2_LQ_YHS_c50640/f1p19/2438	3.464916169	205.7630005	-5.892	6.64E-17	8.81E-15
i2_LQ_YHS_c33413/f1p0/2572	18.03668953	447.3430419	-4.6324	6.67E-17	8.83E-15
i2_HQ_YHS_c45696/f2p2/2113	2.390655117	181.6192396	-6.2474	6.71E-17	8.85E-15
i1_HQ_YHS_c1547/f8p0/1601	64.10133745	3344.664303	-5.7054	7.09E-17	9.33E-15
i1_LQ_YHS_c6795/f1p3/1585	21.73785311	518.7298579	-4.5767	7.57E-17	9.94E-15
i3_LQ_YHS_c10729/f1p2/3320	0	111.5058536	-Inf	7.64E-17	1.00E-14
i1_HQ_YHS_c1367/f5p0/1927	32.70636517	735.0818093	-4.4903	7.76E-17	1.01E-14
i2_HQ_YHS_c11636/f2p21/3003	18.77056823	464.7111468	-4.6298	8.04E-17	1.05E-14
i0_LQ_YHS_c1388/f1p0/928	49.92415549	1178.666674	-4.5613	8.11E-17	1.05E-14
i2_LQ_YHS_c20084/f1p2/2396	18.06563404	424.4142194	-4.5542	8.12E-17	1.05E-14
i1_LQ_YHS_c34473/f1p0/1521	645.8486772	0	Inf	8.25E-17	1.07E-14
i1_LQ_YHS_c6911/f1p1/1487	204.383236	3.239945446	5.9792	8.61E-17	1.11E-14
i2_LQ_YHS_c36783/f1p0/2074	44.83522385	971.5778717	-4.4376	8.83E-17	1.13E-14
i3_LQ_YHS_c10220/f1p3/3069	4.841726805	229.677734	-5.5679	8.85E-17	1.13E-14
i0_HQ_YHS_c348/f2p0/626	65.8101173	3516.958981	-5.7399	8.93E-17	1.14E-14
i0_HQ_YHS_c312/f2p0/815	61.30113161	3898.58224	-5.9909	9.09E-17	1.16E-14
i4_LQ_YHS_c5772/f1p6/4434	29.95754284	688.9406617	-4.5234	9.10E-17	1.16E-14
i0_LQ_YHS_c3054/f1p0/711	64.7030953	2206.93517	-5.0921	9.18E-17	1.16E-14
i2_HQ_YHS_c58283/f2p1/2040	34.63312408	793.7420299	-4.5184	9.23E-17	1.17E-14
i4_HQ_YHS_c2376/f2p0/4437	56.33776626	1510.705897	-4.745	9.28E-17	1.17E-14
i2_LQ_YHS_c60400/f2p5/2225	8.97215871	333.2442889	-5.215	1.04E-16	1.31E-14
i1_HQ_YHS_c37892/f2p0/1118	10.33847866	319.1388869	-4.9481	1.06E-16	1.34E-14
i1_LQ_YHS_c43936/f1p0/1045	69.66914975	2847.424639	-5.353	1.07E-16	1.34E-14
i1_HQ_YHS_c1731/f11p1/1637	39.95738641	885.9846326	-4.4707	1.19E-16	1.49E-14
i4_LQ_YHS_c4607/f1p0/4728	2.647892274	185.5093037	-6.1305	1.21E-16	1.51E-14
i2_LQ_YHS_c51843/f1p6/2490	68.42987046	2427.337602	-5.1486	1.31E-16	1.63E-14
i1_LQ_YHS_c7992/f1p0/1722	44.89563204	981.7128715	-4.4507	1.32E-16	1.64E-14
i2_LQ_YHS_c41824/f1p0/2245	21.66774289	511.211139	-4.5603	1.33E-16	1.64E-14
i1_LQ_YHS_c38584/f1p0/1302	40.60700097	883.1753705	-4.4429	1.41E-16	1.74E-14
i4_HQ_YHS_c2291/f2p4/4613	4.89165269	220.0142588	-5.4911	1.42E-16	1.74E-14
i2_LQ_YHS_c40183/f1p8/2891	50.80112922	1138.569551	-4.4862	1.43E-16	1.75E-14
i1_LQ_YHS_c8223/f1p2/1405	12.58604713	352.4311549	-4.8074	1.47E-16	1.79E-14
i1_LQ_YHS_c7505/f1p0/1944	1.634047791	159.2505231	-6.6067	1.62E-16	1.98E-14
i2_LQ_YHS_c18409/f1p6/1927	54.72008638	1395.051871	-4.6721	1.62E-16	1.98E-14
i2_LQ_YHS_c23153/f1p5/2744	18.87469479	454.571279	-4.59	1.66E-16	2.02E-14
i3_LQ_YHS_c10728/f1p7/3515	59.43590823	1535.62013	-4.6913	1.77E-16	2.14E-14
i2_LQ_YHS_c33598/f1p1/2320	70.44706874	2432.351908	-5.1097	1.92E-16	2.33E-14
i1_LQ_YHS_c3818/f1p0/1961	52.13898813	1135.805945	-4.4452	2.01E-16	2.42E-14
i1_LQ_YHS_c18594/f1p0/1759	53.38225258	1925.20302	-5.1725	2.06E-16	2.47E-14
i0_LQ_YHS_c3002/f1p1/755	27.89758479	1124.369065	-5.3328	2.16E-16	2.59E-14
i0_LQ_YHS_c1222/f1p0/561	23.45538476	500.2538964	-4.4147	2.21E-16	2.65E-14
i2_HQ_YHS_c35332/f2p4/2217	26.31933174	1205.745345	-5.5177	2.28E-16	2.72E-14
i1_HQ_YHS_c2220/f7p1/1963	72.33074596	2268.278187	-4.9708	2.30E-16	2.73E-14
i3_LQ_YHS_c3300/f1p0/3246	11.18110576	309.437024	-4.7905	2.30E-16	2.73E-14
i2_LQ_YHS_c44521/f1p6/2009	9.256262835	295.4511691	-4.9963	2.39E-16	2.83E-14
i1_LQ_YHS_c35905/f1p1/1843	60.26423659	1481.620436	-4.6197	2.50E-16	2.95E-14
i2_LQ_YHS_c4017/f1p1/2923	16.30900586	399.0052433	-4.6127	2.64E-16	3.11E-14
i1_LQ_YHS_c21269/f1p2/1607	11.96123036	337.0051314	-4.8163	2.71E-16	3.18E-14
i2_LQ_YHS_c56502/f1p3/2485	65.2221747	1817.86645	-4.8007	2.72E-16	3.19E-14
i2_LQ_YHS_c5843/f1p4/2560	60.52400128	4783.912306	-6.3045	2.85E-16	3.33E-14
i2_LQ_YHS_c54018/f1p2/2226	61.42912249	1517.426895	-4.6266	2.93E-16	3.42E-14
i2_LQ_YHS_c1598/f4p0/2790	9.500473397	281.7571837	-4.8903	2.99E-16	3.48E-14
i2_LQ_YHS_c7491/f1p2/2238	22.09240613	475.01917	-4.4264	3.28E-16	3.81E-14
i1_LQ_YHS_c20071/f1p0/1846	3.707049183	197.2602342	-5.7337	3.55E-16	4.12E-14
i2_HQ_YHS_c57778/f2p2/2047	0	308.7267853	-Inf	3.58E-16	4.13E-14
i1_HQ_YHS_c40213/f4p0/1882	935.1913644	45.43151002	4.3635	3.64E-16	4.19E-14
i3_HQ_YHS_c1635/f4p0/3613	69.61112428	2036.665046	-4.8707	3.67E-16	4.22E-14
i1_LQ_YHS_c34476/f1p0/1875	12.54534816	334.6156612	-4.7373	3.72E-16	4.27E-14
i2_LQ_YHS_c5538/f1p4/2633	4.902143374	239.0868291	-5.608	3.74E-16	4.28E-14
i3_HQ_YHS_c21338/f7p0/3092	51.01846444	1078.291393	-4.4016	3.83E-16	4.37E-14
i2_LQ_YHS_c42129/f1p2/2213	15.64811196	358.7820565	-4.519	3.86E-16	4.40E-14
i2_LQ_YHS_c55254/f1p2/2303	17.46259568	714.1294514	-5.3538	3.97E-16	4.51E-14
i3_LQ_YHS_c2488/f2p5/3138	69.22732994	1770.2827	-4.6765	4.06E-16	4.61E-14
i1_LQ_YHS_c34256/f1p16/1370	21.02735524	649.0967871	-4.9481	4.12E-16	4.66E-14
i2_LQ_YHS_c18257/f1p0/2743	8.418257571	382.7113182	-5.5066	4.45E-16	5.03E-14
i3_LQ_YHS_c5275/f1p2/3134	8.941950426	283.6737389	-4.9875	4.47E-16	5.04E-14
i1_LQ_YHS_c13454/f1p3/1180	64.6204336	1533.952609	-4.5691	4.52E-16	5.07E-14
i1_LQ_YHS_c36841/f1p4/1751	45.07987602	936.6764704	-4.377	4.64E-16	5.20E-14
i1_LQ_YHS_c33582/f1p0/1451	14.50419905	365.7035353	-4.6561	4.86E-16	5.43E-14
i1_LQ_YHS_c5636/f1p1/1512	43.57429422	856.8838487	-4.2976	4.86E-16	5.43E-14
i2_LQ_YHS_c36511/f1p0/2131	11.71194798	320.8598672	-4.7759	5.12E-16	5.70E-14
i1_LQ_YHS_c13680/f1p0/1367	8.988534995	588.245489	-6.0322	5.14E-16	5.71E-14
i2_HQ_YHS_c3386/f2p4/2226	71.93294131	2102.43082	-4.8693	5.49E-16	6.07E-14
i2_LQ_YHS_c29379/f1p0/2071	15.39213857	384.2772441	-4.6419	5.49E-16	6.07E-14
i1_LQ_YHS_c8985/f1p4/1521	35.67051096	695.4665288	-4.2852	5.54E-16	6.11E-14
i2_LQ_YHS_c38624/f1p0/2414	0	170.620212	-Inf	5.93E-16	6.53E-14
i4_LQ_YHS_c9728/f1p2/4324	21.49110664	476.0058699	-4.4692	6.02E-16	6.61E-14
i2_LQ_YHS_c59920/f1p1/2134	22.40877934	489.9534464	-4.4505	6.04E-16	6.61E-14
i3_LQ_YHS_c15040/f1p4/3034	11.06821902	410.6571151	-5.2134	6.05E-16	6.61E-14
i1_LQ_YHS_c23123/f1p0/1663	14.15252898	355.0354704	-4.6488	6.09E-16	6.64E-14

i1_LQ_YHS_c7775/f1p4/1948	2.88287592	173.1873626	-5.9087	6.30E-16	6.85E-14
i0_LQ_YHS_c603/f1p0/863	18.04671348	403.1131611	-4.4814	6.71E-16	7.27E-14
i1_LQ_YHS_c24899/f1p1/1366	72.22500021	3525.576243	-5.6092	6.69E-16	7.27E-14
i2_LQ_YHS_c22586/f1p0/2112	12.95951234	321.613188	-4.6332	6.80E-16	7.35E-14
i3_LQ_YHS_c4346/f1p1/3878	0	102.7257135	-Inf	6.94E-16	7.49E-14
i2_LQ_YHS_c33516/f1p1/2115	61.34102517	1449.969283	-4.563	7.24E-16	7.79E-14
i1_LQ_YHS_c18163/f1p0/1886	527.0127165	25.69302092	4.3584	7.32E-16	7.86E-14
i2_LQ_YHS_c15248/f1p1/2054	63.88908243	1475.391224	-4.5294	7.52E-16	8.05E-14
i2_LQ_YHS_c12718/f2p12/2448	71.30237538	1912.167438	-4.7451	7.65E-16	8.16E-14
i2_LQ_YHS_c52579/f1p4/2674	56.81822424	1238.118576	-4.4457	7.64E-16	8.16E-14
i3_LQ_YHS_c4883/f1p2/3143	5.607561047	217.3971802	-5.2768	7.81E-16	8.31E-14
i0_LQ_YHS_c1844/f1p3/534	2.702431617	160.8435404	-5.8953	8.11E-16	8.61E-14
i1_LQ_YHS_c24566/f2p0/1839	32.3750733	634.3646012	-4.2924	8.44E-16	8.95E-14
i2_LQ_YHS_c54083/f1p5/2593	76.78228422	2621.736979	-5.0936	8.66E-16	9.15E-14
i2_LQ_YHS_c22771/f1p1/2444	5.174484673	224.5655033	-5.4396	9.23E-16	9.74E-14
i1_LQ_YHS_c3860/f1p1/1845	21.19236511	463.9081398	-4.4522	9.75E-16	1.03E-13
i1_LQ_YHS_c14740/f1p0/1378	68.85880011	1628.475198	-4.5637	9.95E-16	1.05E-13
i2_LQ_YHS_c33003/f1p6/2735	2.980650142	175.8853226	-5.8829	1.02E-15	1.07E-13
i2_LQ_YHS_c51561/f1p68/2835	2.163626246	159.1205087	-6.2005	1.04E-15	1.09E-13
i1_LQ_YHS_c35243/f1p2/1970	14.67495807	349.1822425	-4.5726	1.08E-15	1.12E-13
i2_LQ_YHS_c27246/f1p6/2650	53.8787398	1113.027911	-4.3686	1.14E-15	1.19E-13
i2_LQ_YHS_c10555/f1p2/2680	29.56899024	575.7465773	-4.2833	1.16E-15	1.20E-13
i1_LQ_YHS_c21887/f1p5/1487	24.1758982	486.3581701	-4.3304	1.19E-15	1.24E-13
i1_LQ_YHS_c13963/f1p0/1371	183.7211554	3.300016492	5.7989	1.20E-15	1.25E-13
i2_HQ_YHS_c14784/f3p3/2728	53.41894964	1036.471719	-4.2782	1.21E-15	1.25E-13
i3_HQ_YHS_c2169/f4p0/3324	5.976404426	223.8368705	-5.227	1.34E-15	1.38E-13
i1_LQ_YHS_c37813/f2p0/1073	67.30187547	5116.066723	-6.2482	1.35E-15	1.39E-13
i1_LQ_YHS_c25943/f1p1/1816	1.361706493	225.7821816	-7.3734	1.37E-15	1.40E-13
i3_LQ_YHS_c3847/f1p1/3566	1.543422937	145.2722557	-6.5565	1.40E-15	1.43E-13
i2_LQ_YHS_c22134/f1p2/2853	8.670881269	264.6096229	-4.9315	1.41E-15	1.44E-13
i1_LQ_YHS_c27680/f1p0/1070	33.42340923	810.152488	-4.5993	1.49E-15	1.52E-13
i3_LQ_YHS_c18508/f1p0/3094	43.65488675	825.0469204	-4.2403	1.54E-15	1.57E-13
i0_LQ_YHS_c2377/f1p2/779	68.68296089	1557.650352	-4.5033	1.62E-15	1.65E-13
i3_HQ_YHS_c21222/f49p0/3620	76.70962134	2309.50446	-4.912	1.64E-15	1.66E-13
i5_LQ_YHS_c4672/f1p1/5046	15.91376225	356.6333101	-4.4861	1.68E-15	1.70E-13
i0_LQ_YHS_c3685/f2p0/817	81.03769599	2708.629313	-5.0628	1.76E-15	1.77E-13
i1_LQ_YHS_c24555/f2p0/1417	37.22141357	6314.578693	-7.4064	1.76E-15	1.77E-13
i2_LQ_YHS_c7741/f1p5/2758	64.92597745	1408.520907	-4.4392	1.81E-15	1.82E-13
i2_HQ_YHS_c19275/f2p1/2570	11.24913843	290.2808061	-4.6896	1.82E-15	1.82E-13
i2_LQ_YHS_c41452/f1p2/2930	36.49342046	653.8543529	-4.1633	1.94E-15	1.93E-13
i3_LQ_YHS_c7443/f1p3/3534	10.88316126	291.687049	-4.7443	1.94E-15	1.93E-13
i1_LQ_YHS_c35603/f1p0/1532	17.81633491	398.2172425	-4.4823	1.97E-15	1.96E-13
i1_LQ_YHS_c4928/f2p0/1465	83.81023783	3037.478655	-5.1796	2.10E-15	2.09E-13
i2_LQ_YHS_c20160/f1p12/2318	69.37359635	1555.474652	-4.4868	2.18E-15	2.16E-13
i2_LQ_YHS_c33783/f1p2/2282	24.45952722	488.7730336	-4.3207	2.25E-15	2.23E-13
i1_HQ_YHS_c40253/f4p0/1402	41.12689415	7537.113993	-7.5178	2.32E-15	2.29E-13
i1_LQ_YHS_c3793/f1p0/1924	12.95950397	587.7463902	-5.5031	2.34E-15	2.31E-13
i2_LQ_YHS_c39740/f1p0/2144	5.509786825	316.628094	-5.8446	2.36E-15	2.32E-13
i2_LQ_YHS_c60428/f52p6/2443	56.81778262	1019.381354	-4.1652	2.38E-15	2.34E-13
i3_LQ_YHS_c19384/f1p0/3723	19.7610235	491.7085177	-4.6371	2.46E-15	2.41E-13
i1_LQ_YHS_c13701/f1p0/1309	350.4496453	15.35777826	4.5122	2.52E-15	2.47E-13
i4_LQ_YHS_c9784/f1p1/4201	14.16174753	339.3859088	-4.5829	2.53E-15	2.47E-13
i3_LQ_YHS_c6616/f1p1/3748	78.89727347	1912.968772	-4.5997	2.58E-15	2.52E-13
i2_LQ_YHS_c21615/f1p0/2396	24.04537141	473.329391	-4.299	2.63E-15	2.55E-13
i1_LQ_YHS_c11287/f2p0/1897	5.71916727	214.0618992	-5.2261	2.77E-15	2.69E-13
i2_LQ_YHS_c3001/f1p3/2802	15.54238296	329.6671382	-4.4067	2.94E-15	2.84E-13
i5_LQ_YHS_c2851/f1p0/5650	23.02945776	448.2460151	-4.2827	2.96E-15	2.86E-13
i2_LQ_YHS_c50770/f1p0/2280	39.59570077	708.9124832	-4.1622	3.03E-15	2.93E-13
i1_LQ_YHS_c39697/f1p0/1265	85.2072328	3446.266057	-5.3379	3.21E-15	3.09E-13
i3_LQ_YHS_c18107/f1p0/3560	48.64925742	883.0577204	-4.182	3.28E-15	3.15E-13
i4_LQ_YHS_c4706/f1p0/4487	13.00608854	648.1464578	-5.6391	3.32E-15	3.19E-13
i1_LQ_YHS_c24923/f1p0/1499	73.17557029	1522.709745	-4.3791	3.55E-15	3.39E-13
i1_LQ_YHS_c21183/f1p1/1554	86.65876711	2545.00126	-4.8762	3.61E-15	3.45E-13
i1_LQ_YHS_c11354/f1p1/1877	25.90390379	500.7297309	-4.2728	3.67E-15	3.50E-13
i3_LQ_YHS_c15266/f14p0/3203	44.00053478	792.4358553	-4.1707	3.96E-15	3.77E-13
i2_LQ_YHS_c1381/f5p4/2197	30.74309469	2032.732779	-6.047	3.97E-15	3.77E-13
i2_HQ_YHS_c8565/f2p0/2532	37.99614443	650.744229	-4.0982	3.99E-15	3.78E-13
i1_LQ_YHS_c28365/f1p0/1295	29.44100773	1023.713986	-5.1198	4.01E-15	3.79E-13
i3_HQ_YHS_c1935/f4p2/3145	20.76805729	551.2176577	-4.7302	4.04E-15	3.81E-13
i2_LQ_YHS_c54612/f1p5/2548	3.268095583	170.579057	-5.7058	4.07E-15	3.83E-13
i1_LQ_YHS_c18254/f1p0/1503	23.10831141	576.5820408	-4.641	4.10E-15	3.86E-13
i0_LQ_YHS_c1788/f1p0/974	9.996502251	264.6813435	-4.7267	4.21E-15	3.95E-13
i0_LQ_YHS_c3661/f3p0/965	0	384.0239226	-Inf	4.39E-15	4.11E-13
i1_LQ_YHS_c5807/f1p0/1798	3.23662353	654.8535011	-7.6605	4.49E-15	4.19E-13
i1_LQ_YHS_c38952/f1p0/1205	44.36190687	775.3087192	-4.1274	4.50E-15	4.20E-13
i2_LQ_YHS_c3769/f1p0/2726	11.76187387	290.93748	-4.6285	4.65E-15	4.33E-13
i2_LQ_YHS_c13424/f1p0/2823	8.893305056	242.4097865	-4.7686	4.70E-15	4.37E-13
i2_LQ_YHS_c55611/f1p2/2408	42.20241059	758.7407425	-4.1682	4.73E-15	4.39E-13
i1_LQ_YHS_c13417/f1p0/1381	89.61796078	3032.960899	-5.0808	4.76E-15	4.40E-13
i4_LQ_YHS_c13551/f1p0/4084	67.70103845	1427.445057	-4.3981	4.78E-15	4.41E-13
i2_LQ_YHS_c53978/f1p1/2499	115.4534693	0.359993938	8.3251	5.00E-15	4.61E-13
i1_LQ_YHS_c26473/f1p8/1914	222.0837928	6.369700195	5.1237	5.21E-15	4.80E-13
i2_LQ_YHS_c20906/f1p19/2717	21.55152321	661.2124417	-4.9393	5.29E-15	4.86E-13
i1_LQ_YHS_c26036/f1p0/1840	2.527864541	149.8935248	-5.8899	5.37E-15	4.92E-13
i5_LQ_YHS_c4290/f1p0/5761	5.673854843	208.8822507	-5.2022	5.37E-15	4.92E-13
i2_LQ_YHS_c5451/f1p3/2210	6.808532464	226.1656301	-5.0539	5.42E-15	4.95E-13
i1_HQ_YHS_c20700/f2p0/1439	47.3735707	824.0960416	-4.1207	5.44E-15	4.96E-13
i3_LQ_YHS_c12169/f1p0/3605	1.90638909	144.7400623	-6.2465	5.71E-15	5.20E-13
i2_LQ_YHS_c10461/f1p1/2571	36.73682562	626.5398328	-4.0921	5.73E-15	5.21E-13

i1_LQ_YHS_c14695/f1p0/1331	22.88302979	1094.57497	-5.5799	6.00E-15	5.43E-13
i2_HQ_YHS_c17716/f3p3/2432	13.9221588	395.7545119	-4.8292	6.00E-15	5.43E-13
i2_LQ_YHS_c51426/f1p9/2334	37.06369788	778.2894516	-4.3922	6.10E-15	5.51E-13
i0_LQ_YHS_c762/f1p0/854	49.43114603	830.3505324	-4.0702	6.13E-15	5.51E-13
i1_LQ_YHS_c19021/f1p0/1846	13.91801207	324.6899789	-4.544	6.12E-15	5.51E-13
i1_HQ_YHS_c39987/f18p3/1962	91.37285008	3447.73084	-5.2377	6.23E-15	5.60E-13
i1_LQ_YHS_c10577/f1p3/1958	22.92707845	457.6183717	-4.319	6.34E-15	5.67E-13
i2_LQ_YHS_c28870/f1p1/2044	22.42562236	435.9656602	-4.281	6.33E-15	5.67E-13
i3_LQ_YHS_c17754/f1p4/3838	61.13211146	1134.78497	-4.2143	6.83E-15	6.10E-13
i1_LQ_YHS_c17443/f1p0/1572	17.26577509	363.1033248	-4.3944	7.07E-15	6.30E-13
i4_LQ_YHS_c6208/f1p1/4335	17.85705063	349.082718	-4.289	7.17E-15	6.38E-13
i1_LQ_YHS_c39413/f1p0/1170	9.766132063	917.9747436	-6.5545	7.59E-15	6.74E-13
i2_HQ_YHS_c32615/f2p8/2510	51.04038765	946.4055517	-4.2127	7.61E-15	6.75E-13
i3_LQ_YHS_c16831/f1p0/3655	45.14699197	742.2841059	-4.0393	7.98E-15	7.07E-13
i2_LQ_YHS_c52185/f1p0/2102	113.5373949	0	Inf	8.17E-15	7.21E-13
i2_LQ_YHS_c35288/f1p9/2926	18.46214142	367.648535	-4.3157	8.20E-15	7.23E-13
i2_LQ_YHS_c12363/f1p3/2105	5.71916727	309.16822	-5.7564	8.39E-15	7.38E-13
i1_LQ_YHS_c31818/f1p0/1803	83.79052023	1964.771559	-4.5514	8.46E-15	7.44E-13
i1_LQ_YHS_c6651/f1p2/1705	48.52001115	814.1736824	-4.0687	8.66E-15	7.60E-13
i3_LQ_YHS_c6187/f1p0/3783	19.52257048	379.8350047	-4.2822	8.94E-15	7.83E-13
i2_LQ_YHS_c25697/f1p3/2350	92.63897959	3568.12708	-5.2674	9.71E-15	8.48E-13
i1_LQ_YHS_c33254/f1p0/1671	46.90026209	789.3057446	-4.0729	9.78E-15	8.53E-13
i2_LQ_YHS_c26924/f1p2/2250	4.054911194	179.5897634	-5.4689	9.81E-15	8.53E-13
i2_LQ_YHS_c52130/f1p1/2764	8.743060665	320.6032681	-5.1965	9.81E-15	8.53E-13
i1_LQ_YHS_c11986/f1p4/1767	7.825726637	219.7725879	-4.8116	1.01E-14	8.78E-13
i4_LQ_YHS_c13613/f1p1/4114	0	91.31681071	-Inf	1.01E-14	8.78E-13
i1_HQ_YHS_c1431/f5p4/1791	27.83429364	492.3874666	-4.1449	1.02E-14	8.78E-13
i1_LQ_YHS_c4350/f1p5/2034	18.29934555	350.0792868	-4.2578	1.02E-14	8.81E-13
i1_HQ_YHS_c4133/f3p2/1861	161.2164927	2.879951507	5.8068	1.03E-14	8.89E-13
i2_LQ_YHS_c49795/f1p2/2384	1.361706493	128.7898653	-6.5635	1.05E-14	9.04E-13
i2_LQ_YHS_c51149/f1p1/2290	8.639400842	247.1608772	-4.8384	1.06E-14	9.10E-13
i1_HQ_YHS_c15170/f2p0/1674	73.96479375	1382.494177	-4.2243	1.07E-14	9.15E-13
i1_LQ_YHS_c18710/f1p3/1965	85.57097924	4426.800319	-5.693	1.07E-14	9.17E-13
i1_LQ_YHS_c19425/f1p1/1931	23.41086937	426.5523019	-4.1875	1.11E-14	9.47E-13
i1_LQ_YHS_c11370/f1p0/1939	23.83598259	442.481728	-4.2144	1.14E-14	9.75E-13
i1_LQ_YHS_c18886/f1p0/1831	21.03451299	414.974402	-4.3022	1.16E-14	9.90E-13
i0_LQ_YHS_c781/f1p0/584	62.41483624	2496.139645	-5.3217	1.17E-14	9.91E-13
i1_HQ_YHS_c15142/f6p0/1991	3580.566069	97.13082463	5.2041	1.19E-14	1.01E-12
i1_LQ_YHS_c35329/f1p1/1610	16.34047791	1057.57418	-6.0162	1.20E-14	1.01E-12
i1_LQ_YHS_c27555/f1p0/1346	24.11961998	446.9063924	-4.2117	1.21E-14	1.02E-12
i3_HQ_YHS_c1458/f7p0/3138	46.03665363	764.8379645	-4.0543	1.22E-14	1.03E-12
i1_HQ_YHS_c13339/f2p1/1324	92.77523042	3368.878968	-5.1824	1.23E-14	1.04E-12
i2_LQ_YHS_c23036/f1p2/2276	61.81449534	1026.152305	-4.0532	1.24E-14	1.05E-12
i2_LQ_YHS_c19651/f1p4/2601	6.253359183	403.5478477	-6.012	1.26E-14	1.06E-12
i3_LQ_YHS_c10934/f1p0/3912	92.73087659	3980.653139	-5.4238	1.28E-14	1.07E-12
i1_HQ_YHS_c27213/f2p0/1238	51.21703227	835.1411987	-4.0273	1.29E-14	1.08E-12
i0_LQ_YHS_c684/f1p0/551	93.52643563	2669.842443	-4.8352	1.29E-14	1.08E-12
i1_LQ_YHS_c26663/f1p0/1920	6.689776874	206.1524977	-4.9456	1.31E-14	1.09E-12
i2_LQ_YHS_c20358/f1p3/2318	21.51496259	418.7139897	-4.2826	1.31E-14	1.09E-12
i2_HQ_YHS_c60318/f3p1/2087	27.19057306	463.6430864	-4.0918	1.32E-14	1.10E-12
i3_LQ_YHS_c14421/f1p3/3045	24.22581571	871.1271157	-5.1683	1.35E-14	1.12E-12
i2_HQ_YHS_c57842/f2p1/2073	15.14631719	338.0501636	-4.4802	1.37E-14	1.13E-12
i3_HQ_YHS_c1545/f5p2/3310	58.58788739	981.0588418	-4.0657	1.38E-14	1.14E-12
i3_LQ_YHS_c14480/f1p1/3045	0.257237156	100.2196579	-8.6059	1.44E-14	1.19E-12
i2_HQ_YHS_c29654/f26p2/2297	37.36706125	1217.684514	-5.0262	1.45E-14	1.19E-12
i2_LQ_YHS_c19701/f1p5/2698	53.38400821	853.2135238	-3.9984	1.45E-14	1.19E-12
i3_LQ_YHS_c16720/f1p0/3291	74.19479177	1465.035574	-4.3035	1.47E-14	1.21E-12
i4_LQ_YHS_c3536/f1p2/4995	6.990248908	219.7872431	-4.9746	1.48E-14	1.22E-12
i2_HQ_YHS_c32007/f14p8/2547	97.17909866	3232.980768	-5.0561	1.52E-14	1.24E-12
i4_LQ_YHS_c3603/f1p0/4696	42.3510693	667.501022	-3.9783	1.53E-14	1.25E-12
i2_LQ_YHS_c43134/f2p0/2013	11.52689022	972.7426464	-6.399	1.54E-14	1.25E-12
i2_LQ_YHS_c50349/f1p2/2929	33.00924505	560.1138744	-4.0848	1.55E-14	1.26E-12
i3_LQ_YHS_c8353/f1p0/3704	215.2344166	0	Inf	1.55E-14	1.26E-12
i4_LQ_YHS_c9851/f1p1/5040	230.7985624	0.707744466	8.3492	1.56E-14	1.27E-12
i1_LQ_YHS_c31912/f1p0/2014	81.96729308	1541.573028	-4.2332	1.59E-14	1.29E-12
i1_LQ_YHS_c9899/f1p1/1393	79.47770291	1580.069126	-4.3133	1.61E-14	1.30E-12
i1_LQ_YHS_c19430/f1p0/1953	0.272341299	182.0122071	-9.3844	1.62E-14	1.31E-12
i2_LQ_YHS_c23756/f1p1/2024	6.330152037	199.2659696	-4.9763	1.63E-14	1.31E-12
i2_LQ_YHS_c3138/f1p15/2645	80.05004113	1646.993623	-4.3628	1.63E-14	1.31E-12
i2_LQ_YHS_c34082/f1p12/2491	53.09146583	880.784438	-4.0522	1.68E-14	1.35E-12
i1_HQ_YHS_c16638/f5p0/1794	44.70929377	739.1022555	-4.0471	1.69E-14	1.36E-12
i1_LQ_YHS_c35616/f1p0/1827	295.4116156	1.82611508	7.3378	1.71E-14	1.37E-12
i1_HQ_YHS_c1664/f3p3/1818	1267.082123	72.0969662	4.1354	1.72E-14	1.38E-12
i4_LQ_YHS_c11917/f1p0/4353	4.136317506	165.2514624	-5.3202	1.81E-14	1.44E-12
i1_HQ_YHS_c27356/f2p33/1212	1.634047791	1881.21641	-10.169	1.82E-14	1.45E-12
i1_HQ_YHS_c2859/f2p5/1980	88.39552451	1964.839026	-4.4743	1.83E-14	1.45E-12
i4_LQ_YHS_c6847/f1p5/4216	7.646546103	218.3282293	-4.8355	1.83E-14	1.45E-12
i1_LQ_YHS_c8221/f1p6/2038	13.10386276	294.4291674	-4.4899	1.91E-14	1.52E-12
i0_HQ_YHS_c184/f5p0/998	61.71036878	7451.111405	-6.9158	1.93E-14	1.53E-12
i4_LQ_YHS_c6077/f1p0/5050	11.1043129	445.6369107	-5.3267	1.97E-14	1.55E-12
i2_HQ_YHS_c37010/f2p5/2365	93.40847707	4321.884698	-5.532	1.99E-14	1.57E-12
i1_LQ_YHS_c32290/f1p0/1411	15.06606333	310.9957168	-4.3675	2.01E-14	1.58E-12
i2_LQ_YHS_c51319/f1p8/2295	24.32693125	1590.5725	-6.0308	2.02E-14	1.59E-12
i1_LQ_YHS_c25652/f1p0/1707	23.71515783	421.9994312	-4.1534	2.04E-14	1.60E-12
i3_HQ_YHS_c2154/f2p0/3386	9.53194545	250.3866081	-4.7152	2.05E-14	1.61E-12
i1_LQ_YHS_c13736/f1p2/1319	95.02201023	2507.425433	-4.7218	2.07E-14	1.62E-12
i1_HQ_YHS_c10835/f4p0/1751	68.03796816	1150.805432	-4.0802	2.16E-14	1.69E-12
i1_LQ_YHS_c32735/f1p12/1999	7.142562474	213.0240557	-4.8984	2.18E-14	1.70E-12
i0_LQ_YHS_c1796/f1p6/1000	82.83470122	1551.314464	-4.2271	2.18E-14	1.70E-12

i2_LQ_YHS_c6041/f1p1/2926	15.86209749	325.6090983	-4.3595	2.21E-14	1.72E-12
i1_LQ_YHS_c25349/f1p0/1780	139.7455956	1.787726281	6.2885	2.22E-14	1.72E-12
i2_LQ_YHS_c39917/f1p1/2869	24.75840519	470.5509819	-4.2484	2.22E-14	1.72E-12
i2_LQ_YHS_c52169/f1p0/2149	0.514474312	106.7679138	-7.6972	2.22E-14	1.72E-12
i2_LQ_YHS_c7044/f1p11/2438	7.701543807	228.5203016	-4.891	2.28E-14	1.76E-12
i1_HQ_YHS_c5227/f2p5/1925	3.519455513	172.3479452	-5.6138	2.33E-14	1.79E-12
i2_LQ_YHS_c25273/f1p2/2725	33.52024161	560.4429412	-4.0635	2.33E-14	1.79E-12
i1_LQ_YHS_c13948/f1p0/1353	80.75278646	1567.568194	-4.2789	2.36E-14	1.81E-12
i2_LQ_YHS_c26886/f1p1/2678	6.337301405	222.6626106	-5.1348	2.46E-14	1.89E-12
i2_LQ_YHS_c36027/f1p7/2695	1.72133133	131.004765	-6.25	2.50E-14	1.91E-12
i1_LQ_YHS_c12315/f1p2/1623	3.499737912	159.6845296	-5.5118	2.55E-14	1.95E-12
i2_LQ_YHS_c7655/f1p26/2314	84.39018378	1742.308537	-4.3678	2.57E-14	1.96E-12
i4_LQ_YHS_c12418/f1p4/4814	12.49749145	340.6000495	-4.7684	2.58E-14	1.97E-12
i1_HQ_YHS_c17001/f2p0/1822	1.089365194	529.3041646	-8.9245	2.76E-14	2.10E-12
i1_LQ_YHS_c9190/f1p0/1783	40.86679079	623.1130833	-3.9305	2.82E-14	2.15E-12
i1_LQ_YHS_c12206/f1p0/1835	87.29359946	1737.827717	-4.3153	2.86E-14	2.17E-12
i4_LQ_YHS_c5508/f1p0/4312	2.897980062	149.2454586	-5.6865	2.89E-14	2.19E-12
i1_HQ_YHS_c2446/f5p0/1942	11.84120263	276.654193	-4.5462	2.93E-14	2.22E-12
i1_HQ_YHS_c15076/f6p0/1496	72.27887896	1658.098583	-4.5198	2.97E-14	2.24E-12
i2_HQ_YHS_c17215/f7p4/2986	86.04568805	1798.865604	-4.3858	3.10E-14	2.34E-12
i1_LQ_YHS_c17901/f1p2/1505	19.24942377	363.2629599	-4.2381	3.20E-14	2.41E-12
i4_LQ_YHS_c11530/f1p0/4378	3.931542145	178.2779495	-5.5029	3.27E-14	2.46E-12
i3_LQ_YHS_c14985/f1p3/3029	14.90787254	297.9383063	-4.3209	3.29E-14	2.47E-12
i2_LQ_YHS_c6297/f1p6/2915	45.82393186	708.575284	-3.9507	3.32E-14	2.49E-12
i1_LQ_YHS_c9373/f1p0/1421	3.540436881	162.6990784	-5.5221	3.34E-14	2.50E-12
i1_LQ_YHS_c26227/f1p1/1593	60.86361171	915.6414094	-3.9111	3.45E-14	2.58E-12
i2_HQ_YHS_c13054/f2p3/2845	60.69030841	1105.725776	-4.1874	3.65E-14	2.72E-12
i1_LQ_YHS_c24803/f1p0/2009	93.08589634	1896.502614	-4.3486	3.78E-14	2.80E-12
i1_LQ_YHS_c39366/f1p20/1333	32.23661686	484.8657492	-3.9108	3.77E-14	2.80E-12
i3_LQ_YHS_c14579/f1p0/3037	62.28062946	1006.181874	-4.014	3.77E-14	2.80E-12
i4_LQ_YHS_c7260/f1p3/4475	0	87.11745901	-Inf	3.78E-14	2.80E-12
i2_LQ_YHS_c22896/f1p1/2549	13.47859174	468.1952039	-5.1184	3.96E-14	2.93E-12
i1_LQ_YHS_c31922/f1p0/1706	1.089365194	117.1603427	-6.7489	4.04E-14	2.99E-12
i1_LQ_YHS_c10960/f1p0/1731	47.31524005	690.0807947	-3.8664	4.34E-14	3.20E-12
i1_LQ_YHS_c41533/f1p1/1291	78.00682316	1243.9724	-3.9952	4.44E-14	3.27E-12
i2_HQ_YHS_c61603/f5p4/2058	75.531065	1264.403152	-4.0652	4.44E-14	3.27E-12
i3_LQ_YHS_c19792/f1p1/3444	90.13797365	5323.902854	-5.8842	4.46E-14	3.27E-12
i1_LQ_YHS_c34274/f1p0/1981	8.159748273	222.0032481	-4.7659	4.52E-14	3.31E-12
i1_LQ_YHS_c3664/f1p3/1493	8.421598887	221.1692794	-4.7149	4.59E-14	3.36E-12
i1_LQ_YHS_c8315/f1p0/1662	45.94015992	679.7499026	-3.8872	4.63E-14	3.38E-12
i2_LQ_YHS_c8104/f1p1/2285	98.40125368	2226.120382	-4.4997	4.64E-14	3.38E-12
i0_LQ_YHS_c1299/f1p0/904	23.65220535	395.0766299	-4.0621	4.88E-14	3.56E-12
i2_LQ_YHS_c4606/f4p1/2412	30.59793886	490.1619018	-4.0018	4.98E-14	3.63E-12
i2_LQ_YHS_c36433/f1p5/2618	19.03956822	1086.771673	-5.8349	5.07E-14	3.68E-12
i1_LQ_YHS_c11612/f1p0/1457	69.29981451	1152.736193	-4.0561	5.11E-14	3.70E-12
i1_LQ_YHS_c34192/f1p8/1824	19.4365507	364.2376883	-4.228	5.11E-14	3.70E-12
i2_LQ_YHS_c5542/f1p0/2640	69.44753137	1087.893499	-3.9695	5.14E-14	3.72E-12
i1_LQ_YHS_c3909/f1p3/1576	73.46126848	6930.486447	-6.5598	5.18E-14	3.74E-12
i2_LQ_YHS_c21019/f1p3/2993	37.48325581	627.3249488	-4.0649	5.19E-14	3.74E-12
i1_HQ_YHS_c29182/f19p3/1927	95.85415502	1904.492609	-4.3124	5.21E-14	3.74E-12
i2_LQ_YHS_c59321/f1p0/2023	2.178730389	136.298513	-5.9671	5.20E-14	3.74E-12
i5_LQ_YHS_c1982/f1p3/5072	14.96460075	318.8486819	-4.4132	5.28E-14	3.79E-12
i2_HQ_YHS_c32298/f3p1/2499	61.82037257	923.6553914	-3.9012	5.51E-14	3.95E-12
i1_LQ_YHS_c24007/f1p1/1721	48.75959988	739.9245317	-3.9236	5.55E-14	3.97E-12
i1_LQ_YHS_c23075/f1p0/1808	8.773268949	225.9700157	-4.6869	5.57E-14	3.98E-12
i2_LQ_YHS_c10383/f1p1/2205	76.05301897	1538.458589	-4.3383	5.66E-14	4.04E-12
i2_LQ_YHS_c34866/f1p5/2225	57.74492999	883.3165603	-3.9352	5.67E-14	4.04E-12
i1_LQ_YHS_c27630/f1p0/1142	80.32480703	1381.950812	-4.1047	5.88E-14	4.18E-12
i2_LQ_YHS_c21934/f1p37/2636	66.06746577	1073.216264	-4.0219	6.06E-14	4.31E-12
i1_HQ_YHS_c31516/f2p0/1767	17.29390583	336.0274391	-4.2802	6.27E-14	4.45E-12
i0_LQ_YHS_c794/f1p0/486	18.28169712	332.0738621	-4.183	6.45E-14	4.57E-12
i1_LQ_YHS_c22960/f1p0/1948	8.745596574	357.5570854	-5.3535	6.49E-14	4.59E-12
i1_HQ_YHS_c6561/f3p0/1960	53.54885651	823.3871549	-3.9426	6.57E-14	4.64E-12
i2_HQ_YHS_c52480/f2p9/2698	102.059624	3948.327771	-5.2738	6.58E-14	4.64E-12
i4_LQ_YHS_c2793/f1p0/5041	36.12870706	2962.838356	-6.3577	6.88E-14	4.84E-12
i2_LQ_YHS_c24220/f1p12/2559	104.3657098	2307.269111	-4.4665	6.94E-14	4.88E-12
i2_LQ_YHS_c7628/f1p20/2234	0	272.7561486	-Inf	7.31E-14	5.14E-12
i1_HQ_YHS_c1852/f10p0/1876	61.12813468	872.6481899	-3.8355	7.43E-14	5.21E-12
i1_LQ_YHS_c27626/f1p0/1237	53.42229096	789.8433449	-3.8861	7.49E-14	5.23E-12
i1_LQ_YHS_c32073/f1p0/1443	14.1479239	763.1625929	-5.7533	7.48E-14	5.23E-12
i3_LQ_YHS_c7950/f1p0/3939	30.93959335	1053.646492	-5.0898	7.76E-14	5.42E-12
i1_LQ_YHS_c13398/f2p0/1191	107.7829547	2948.15933	-4.7736	7.89E-14	5.50E-12
i1_HQ_YHS_c16949/f4p0/1552	110.3990614	2816.606877	-4.6732	8.01E-14	5.58E-12
i4_LQ_YHS_c10497/f1p0/4914	97.11599299	5139.420002	-5.7258	8.21E-14	5.71E-12
i2_HQ_YHS_c9060/f2p1/2739	4.184165843	164.3956521	-5.2961	8.32E-14	5.77E-12
i3_LQ_YHS_c19710/f1p2/3139	45.29944198	645.3229898	-3.8325	8.38E-14	5.81E-12
i1_LQ_YHS_c36573/f1p0/1680	4.978936228	172.0534721	-5.1109	8.43E-14	5.84E-12
i2_LQ_YHS_c21446/f1p0/2092	7.082145905	201.951606	-4.8337	8.47E-14	5.86E-12
i0_LQ_YHS_c1377/f1p0/405	47.05143995	691.3684822	-3.8771	8.57E-14	5.92E-12
i1_HQ_YHS_c17064/f2p0/1908	50.8045986	751.5710795	-3.8869	8.60E-14	5.93E-12
i2_HQ_YHS_c12585/f2p2/2675	51.30048264	789.9869031	-3.9448	8.69E-14	5.98E-12
i3_HQ_YHS_c8832/f2p0/3131	35.38366032	549.5000808	-3.957	8.99E-14	6.18E-12
i2_LQ_YHS_c54015/f1p0/2770	18.57628356	346.7724372	-4.2225	9.21E-14	6.32E-12
i1_LQ_YHS_c14077/f1p0/1146	47.02492003	660.640401	-3.8124	9.35E-14	6.40E-12
i1_LQ_YHS_c24352/f1p6/1939	12.20290501	401.6229493	-5.0405	9.34E-14	6.40E-12
i2_LQ_YHS_c43374/f1p3/2018	20.47091819	356.1249317	-4.1207	9.55E-14	6.53E-12
i2_LQ_YHS_c41284/f1p6/2481	108.6667643	2592.058822	-4.5761	9.80E-14	6.69E-12
i0_LQ_YHS_c2260/f1p0/687	83.64933402	1303.95655	-3.9624	1.01E-13	6.88E-12
i2_LQ_YHS_c4259/f1p1/2340	1.634047791	123.5606514	-6.2406	1.02E-13	6.93E-12

i0_HQ_YHS_c344/f2p0/866	77.19264873	7120.039152	-6.5273	1.07E-13	7.30E-12
i1_LQ_YHS_c11700/f1p4/1624	27.04239784	431.2136173	-3.9951	1.10E-13	7.44E-12
i2_LQ_YHS_c24433/f1p1/2611	1.90638909	128.0903346	-6.0702	1.10E-13	7.47E-12
i2_HQ_YHS_c37767/f2p12/2181	82.98352866	6706.578983	-6.3366	1.11E-13	7.54E-12
i1_LQ_YHS_c3714/f1p0/1972	41.86504764	623.4795138	-3.8965	1.13E-13	7.60E-12
i2_HQ_YHS_c61740/f2p20/2429	110.4281089	3191.048123	-4.8529	1.13E-13	7.61E-12
i2_LQ_YHS_c25971/f1p5/2624	61.50097159	899.1000951	-3.8698	1.13E-13	7.64E-12
i2_LQ_YHS_c5292/f1p1/2613	101.3570821	0.359993938	8.1373	1.15E-13	7.73E-12
i1_LQ_YHS_c18016/f1p0/2000	16.87466983	625.5298757	-5.2121	1.17E-13	7.88E-12
i3_LQ_YHS_c12691/f1p0/3766	57.87626219	832.5607355	-3.8465	1.22E-13	8.22E-12
i3_LQ_YHS_c11926/f1p0/3248	25.39070162	414.4528749	-4.0288	1.25E-13	8.41E-12
i2_LQ_YHS_c38498/f1p0/2146	0	81.80728348	-Inf	1.29E-13	8.61E-12
i1_HQ_YHS_c2272/f9p0/1850	103.8302457	2049.516305	-4.303	1.29E-13	8.65E-12
i1_LQ_YHS_c20051/f1p0/1449	62.25978453	910.6979363	-3.8706	1.33E-13	8.90E-12
i2_LQ_YHS_c55310/f1p0/2452	28.97645093	456.923898	-3.979	1.35E-13	9.02E-12
i2_LQ_YHS_c5922/f1p2/2693	8.515226387	221.22939	-4.6994	1.38E-13	9.17E-12
i2_LQ_YHS_c22720/f1p10/2392	64.8479292	949.1356168	-3.8715	1.39E-13	9.21E-12
i3_HQ_YHS_c10080/f2p14/3685	498.6149023	34.92244896	3.8357	1.39E-13	9.25E-12
i2_HQ_YHS_c6338/f2p2/2338	15.1609546	302.2782166	-4.3174	1.41E-13	9.32E-12
i2_LQ_YHS_c25289/f1p12/2861	25.42090991	413.289874	-4.0231	1.41E-13	9.32E-12
i1_HQ_YHS_c8706/f5p0/2024	71.39048108	1039.239504	-3.8637	1.42E-13	9.41E-12
i4_LQ_YHS_c13122/f1p0/4152	2.723412986	140.7841598	-5.6919	1.43E-13	9.43E-12
i1_LQ_YHS_c17989/f1p2/1764	60.16279912	837.1543661	-3.7986	1.51E-13	9.96E-12
i2_LQ_YHS_c43722/f1p3/2046	4.902143374	173.068127	-5.1418	1.52E-13	9.99E-12
i4_LQ_YHS_c4313/f1p13/4248	2.435967545	140.9114487	-5.8542	1.55E-13	1.02E-11
i1_LQ_YHS_c6931/f1p0/2045	4.074628794	156.0552882	-5.2592	1.56E-13	1.03E-11
i1_LQ_YHS_c26656/f1p0/1611	0	80.84527264	-Inf	1.70E-13	1.11E-11
i2_LQ_YHS_c11341/f1p2/2126	80.36469221	1283.186666	-3.997	1.70E-13	1.11E-11
i2_HQ_YHS_c24391/f2p0/2166	231.3466306	10.27343283	4.4931	1.73E-13	1.13E-11
i3_LQ_YHS_c8379/f1p0/3766	66.17893555	1006.755612	-3.9272	1.73E-13	1.13E-11
i1_LQ_YHS_c12716/f1p0/1771	13.18065561	264.4573719	-4.3265	1.73E-13	1.13E-11
i3_LQ_YHS_c14388/f1p3/3058	2.615148079	130.1652278	-5.6373	1.75E-13	1.14E-11
i1_LQ_YHS_c36406/f1p0/1604	12.06108213	250.4634648	-4.3762	1.77E-13	1.15E-11
i2_LQ_YHS_c18111/f1p4/2331	40.12527923	576.7876111	-3.8455	1.86E-13	1.21E-11
i1_LQ_YHS_c29120/f1p0/1301	80.06771468	1188.749021	-3.8921	1.90E-13	1.23E-11
i1_LQ_YHS_c6703/f1p0/1868	41.70445738	589.0431682	-3.8201	1.90E-13	1.23E-11
i4_LQ_YHS_c13780/f1p1/4079	20.25738265	350.3275908	-4.1122	1.92E-13	1.24E-11
i1_LQ_YHS_c19132/f1p2/1424	75.67574571	1158.487571	-3.9363	1.94E-13	1.26E-11
i3_LQ_YHS_c11183/f1p44/3370	99.40506584	1837.824743	-4.2085	2.08E-13	1.34E-11
i2_HQ_YHS_c1336/f6p11/2142	14.42359814	281.7982585	-4.2882	2.09E-13	1.35E-11
i2_LQ_YHS_c41635/f1p0/2316	96.7338011	1610.149052	-4.057	2.10E-13	1.35E-11
i0_LQ_YHS_c2247/f1p6/870	115.4794267	4215.966947	-5.1902	2.31E-13	1.48E-11
i1_HQ_YHS_c29411/f5p13/1438	110.727511	2401.305859	-4.4387	2.38E-13	1.52E-11
i2_LQ_YHS_c29509/f1p1/2032	29.40076712	453.8877878	-3.9484	2.38E-13	1.52E-11
i0_LQ_YHS_c1232/f1p0/918	118.4862916	2824.197358	-4.575	2.43E-13	1.56E-11
i2_LQ_YHS_c35755/f1p1/2282	53.67811116	761.8492413	-3.8271	2.44E-13	1.56E-11
i3_LQ_YHS_c17077/f1p0/3162	1.089365194	108.4654731	-6.6376	2.44E-13	1.56E-11
i2_LQ_YHS_c41374/f1p1/2208	42.45377052	603.640953	-3.8297	2.49E-13	1.59E-11
i3_HQ_YHS_c15259/f20p0/3903	115.4162636	2703.13208	-4.5497	2.50E-13	1.59E-11
i3_LQ_YHS_c11783/f1p0/3156	33.25631345	505.9523033	-3.9273	2.51E-13	1.60E-11
i2_LQ_YHS_c42005/f1p3/2310	34.72169651	489.4335816	-3.8172	2.52E-13	1.60E-11
i2_LQ_YHS_c54592/f1p0/2659	0	220.5473168	-Inf	2.57E-13	1.63E-11
i4_LQ_YHS_c5607/f1p0/4346	27.34541416	418.4723655	-3.9358	2.58E-13	1.63E-11
i4_LQ_YHS_c3868/f1p5/4427	115.0956156	4516.831339	-5.2944	2.58E-13	1.63E-11
i5_LQ_YHS_c4077/f1p0/5270	36.02044215	517.5131039	-3.8447	2.62E-13	1.66E-11
i1_HQ_YHS_c2667/f4p6/1911	25.28417559	393.0035557	-3.9582	2.67E-13	1.68E-11
i3_LQ_YHS_c5058/f1p3/3960	37.33488673	548.0892923	-3.8758	2.71E-13	1.71E-11
i2_LQ_YHS_c43566/f1p6/2181	5.71916727	254.6423522	-5.4765	2.77E-13	1.75E-11
i1_LQ_YHS_c29816/f8p8/1925	7.527782138	828.213957	-6.7816	2.78E-13	1.75E-11
i2_HQ_YHS_c45416/f2p4/2079	119.3727974	3426.520413	-4.8432	2.93E-13	1.84E-11
i2_HQ_YHS_c921/f23p5/2386	100.8189457	1582.287724	-3.9722	2.94E-13	1.84E-11
i3_LQ_YHS_c19878/f1p5/3954	11.83993049	251.4662702	-4.4086	2.96E-13	1.85E-11
i2_LQ_YHS_c8973/f1p17/2300	86.5638591	1335.392603	-3.9474	2.99E-13	1.87E-11
i0_LQ_YHS_c2512/f1p13/805	408.0310854	26.51206142	3.944	3.04E-13	1.90E-11
i2_LQ_YHS_c40001/f1p7/2174	85.98641556	4983.170168	-5.8568	3.09E-13	1.92E-11
i3_LQ_YHS_c6438/f1p0/3123	38.02079741	530.5337065	-3.8026	3.18E-13	1.98E-11
i1_HQ_YHS_c10569/f2p0/1591	87.62411104	1267.725564	-3.8548	3.22E-13	2.00E-11
i2_LQ_YHS_c23265/f1p3/2839	0.544682597	106.4971804	-7.6112	3.28E-13	2.04E-11
i1_LQ_YHS_c6259/f1p0/1848	92.00843879	1383.258283	-3.9102	3.36E-13	2.08E-11
i3_LQ_YHS_c19474/f1p12/3388	4.358732919	154.1219067	-5.144	3.40E-13	2.11E-11
i2_HQ_YHS_c17285/f4p6/2593	113.9868548	4815.151182	-5.4006	3.48E-13	2.15E-11
i0_LQ_YHS_c1098/f1p0/818	80.8062236	1112.686313	-3.7834	3.59E-13	2.22E-11
i2_LQ_YHS_c39042/f1p1/2249	75.23758914	1496.282314	-4.3138	3.61E-13	2.23E-11
i2_HQ_YHS_c32291/f3p2/2606	13.69893798	266.8275656	-4.2838	3.63E-13	2.24E-11
i2_HQ_YHS_c60989/f7p1/2115	111.0888998	2073.965746	-4.2226	3.63E-13	2.24E-11
i2_LQ_YHS_c37194/f1p3/2493	112.1869917	2307.297557	-4.3622	3.75E-13	2.31E-11
i2_LQ_YHS_c22368/f1p1/2086	93.73534934	1502.489655	-4.0026	3.76E-13	2.31E-11
i2_HQ_YHS_c32409/f2p6/2575	106.6861099	5706.356093	-5.7411	3.83E-13	2.35E-11
i3_LQ_YHS_c4639/f1p0/3468	36.69611827	507.4817396	-3.7897	3.88E-13	2.37E-11
i2_LQ_YHS_c33555/f1p2/2012	11.15550256	238.809768	-4.42	3.89E-13	2.38E-11
i1_LQ_YHS_c40148/f1p6/1927	112.2124345	1935.812537	-4.1086	3.91E-13	2.39E-11
i5_LQ_YHS_c4244/f1p0/5671	86.199324	1189.873865	-3.787	3.96E-13	2.42E-11
i1_HQ_YHS_c7934/f2p9/1521	21.60526552	338.7989794	-3.971	4.03E-13	2.45E-11
i2_LQ_YHS_c38392/f1p33/2216	5.601683821	161.6708748	-4.8511	4.13E-13	2.52E-11
i1_LQ_YHS_c38307/f1p4/1089	108.1262117	5644.007144	-5.7059	4.16E-13	2.52E-11
i2_LQ_YHS_c35921/f1p14/3062	12.14962944	254.1091744	-4.3865	4.16E-13	2.52E-11
i1_LQ_YHS_c23546/f1p0/1426	925.7465971	68.85919301	3.7489	4.26E-13	2.58E-11
i1_LQ_YHS_c38011/f1p0/1271	124.4905049	2831.16571	-4.5073	4.32E-13	2.61E-11
i1_LQ_YHS_c3883/f1p0/1802	10.78838968	237.6290415	-4.4612	4.34E-13	2.62E-11

i0_LQ_YHS_c2132/f1p2/644	5.076710451	243.2536682	-5.5824	4.35E-13	2.63E-11
i2_HQ_YHS_c60893/f9p2/2071	72.6243415	979.9438602	-3.7542	4.39E-13	2.65E-11
i2_LQ_YHS_c54595/f1p3/2394	124.6593969	2862.774468	-4.5214	4.47E-13	2.69E-11
i2_HQ_YHS_c12543/f2p0/2985	34.6600078	900.318693	-4.6991	4.48E-13	2.70E-11
i3_HQ_YHS_c6257/f2p2/3255	120.6960106	4043.634698	-5.0662	4.53E-13	2.72E-11
i1_LQ_YHS_c6113/f1p0/1642	4.841726805	163.8994023	-5.0811	4.59E-13	2.76E-11
i1_LQ_YHS_c9596/f1p0/1700	88.76658188	1331.587379	-3.907	4.76E-13	2.85E-11
i3_LQ_YHS_c5174/f1p0/3485	41.65059538	555.9666146	-3.7386	5.01E-13	3.00E-11
i2_LQ_YHS_c58479/f1p0/2065	2.210210815	119.2864202	-5.7541	5.45E-13	3.26E-11
i1_HQ_YHS_c20107/f4p0/1960	44.51214288	601.9532995	-3.7574	5.56E-13	3.32E-11
i1_LQ_YHS_c24834/f1p7/1610	116.503221	2341.440243	-4.329	5.55E-13	3.32E-11
i0_LQ_YHS_c824/f1p0/988	127.4482816	3299.662377	-4.6943	5.65E-13	3.36E-11
i1_LQ_YHS_c5756/f1p6/1533	81.46963666	1148.672443	-3.8176	5.69E-13	3.39E-11
i1_LQ_YHS_c39762/f1p5/1116	26.81156092	394.1629615	-3.8779	5.71E-13	3.39E-11
i1_LQ_YHS_c24757/f1p0/1930	105.6046911	1817.026777	-4.1048	5.74E-13	3.41E-11
i2_LQ_YHS_c51191/f1p1/2187	4.370495745	212.8913565	-5.6062	6.06E-13	3.59E-11
i3_LQ_YHS_c17970/f1p3/3671	23.88129502	369.8615739	-3.953	6.13E-13	3.63E-11
i1_HQ_YHS_c40301/f17p0/1996	122.5550348	2473.176211	-4.3349	6.17E-13	3.65E-11
i2_HQ_YHS_c32594/f2p1/2679	62.96587117	819.6908093	-3.7024	6.19E-13	3.66E-11
i0_LQ_YHS_c1721/f1p5/377	0	224.2936596	-Inf	6.21E-13	3.66E-11
i2_LQ_YHS_c59897/f1p6/2063	6.057810738	239.6055863	-5.3057	6.59E-13	3.88E-11
i2_LQ_YHS_c33870/f1p3/2728	3.162366586	139.2151146	-5.4602	6.77E-13	3.99E-11
i1_LQ_YHS_c43669/f1p0/1020	52.75028645	690.7279974	-3.7109	6.85E-13	4.03E-11
i1_LQ_YHS_c13633/f1p1/1202	79.05336159	1135.296084	-3.8441	6.89E-13	4.05E-11
i1_LQ_YHS_c27953/f1p0/1178	33.02261869	451.4352086	-3.773	6.99E-13	4.10E-11
i1_LQ_YHS_c17883/f1p0/1623	34.60294092	458.5068848	-3.728	7.20E-13	4.22E-11
i1_LQ_YHS_c10325/f1p0/1572	0	75.26906033	-Inf	7.27E-13	4.25E-11
i1_LQ_YHS_c19625/f1p1/1968	9.423680544	210.4099816	-4.4808	7.40E-13	4.33E-11
i2_LQ_YHS_c56254/f1p0/2077	7.709506956	179.6138167	-4.5421	8.27E-13	4.83E-11
i3_LQ_YHS_c20506/f1p0/3037	5.991508569	173.0277659	-4.8519	8.41E-13	4.90E-11
i2_LQ_YHS_c21542/f1p0/2430	0	201.4316968	-Inf	8.53E-13	4.96E-11
i1_LQ_YHS_c9774/f1p1/1636	48.44750145	616.7259651	-3.6701	8.81E-13	5.13E-11
i2_LQ_YHS_c37080/f1p2/2914	0	75.33619911	-Inf	8.95E-13	5.20E-11
i1_LQ_YHS_c43929/f1p0/1078	132.1749328	3272.608499	-4.6299	9.10E-13	5.28E-11
i5_LQ_YHS_c3155/f1p2/5374	3.268095583	214.320782	-6.0352	9.62E-13	5.58E-11
i2_LQ_YHS_c52675/f1p5/2513	70.31000413	895.1368063	-3.6703	1.01E-12	5.87E-11
i2_LQ_YHS_c41651/f1p0/2350	8.088841019	191.7171954	-4.5669	1.02E-12	5.87E-11
i3_LQ_YHS_c18875/f1p1/3356	77.62028949	1056.389001	-3.7666	1.03E-12	5.92E-11
i2_LQ_YHS_c7040/f1p1/2546	1.808614868	111.0477926	-5.9402	1.04E-12	6.01E-11
i1_LQ_YHS_c31748/f1p0/2019	27.13635563	397.4603316	-3.8725	1.06E-12	6.08E-11
i1_LQ_YHS_c8667/f1p4/1547	95.6371166	1431.768415	-3.9041	1.09E-12	6.27E-11
i1_LQ_YHS_c26308/f1p0/1573	435.2694445	14.20382342	4.9376	1.12E-12	6.45E-11
i1_HQ_YHS_c29913/f2p0/1352	37.55544358	981.3986395	-4.7077	1.13E-12	6.50E-11
i1_LQ_YHS_c18848/f1p123/1499	82.10861573	1124.131117	-3.7751	1.14E-12	6.54E-11
i4_LQ_YHS_c9512/f1p6/4226	18.5309795	296.1871498	-3.9985	1.17E-12	6.71E-11
i1_LQ_YHS_c36805/f1p3/1903	108.9516571	1715.92947	-3.9772	1.17E-12	6.71E-11
i2_LQ_YHS_c9481/f1p1/2788	11.55710688	216.3136071	-4.2263	1.18E-12	6.75E-11
i1_LQ_YHS_c24719/f1p0/1799	0	87.80679882	-Inf	1.19E-12	6.80E-11
i1_HQ_YHS_c6192/f3p0/1466	52.38620134	1385.852498	-4.7254	1.25E-12	7.13E-11
i1_LQ_YHS_c38698/f1p0/1228	82.09939718	1106.379052	-3.7523	1.26E-12	7.15E-11
i1_LQ_YHS_c3045/f1p0/1901	26.81664111	378.7972414	-3.8202	1.28E-12	7.29E-11
i1_HQ_YHS_c3363/f2p5/1899	63.35871531	777.3822247	-3.617	1.29E-12	7.34E-11
i2_LQ_YHS_c40360/f1p1/2355	27.53254947	381.964991	-3.7942	1.30E-12	7.38E-11
i3_LQ_YHS_c5156/f1p1/3350	25.52456973	369.2639806	-3.8547	1.30E-12	7.38E-11
i2_LQ_YHS_c10581/f1p1/2210	9.017937873	208.2383674	-4.5293	1.32E-12	7.45E-11
i2_LQ_YHS_c20995/f1p13/1996	133.1766183	2739.207972	-4.3623	1.32E-12	7.45E-11
i2_LQ_YHS_c36237/f1p0/2948	18.75085901	293.2079134	-3.9669	1.32E-12	7.45E-11
i3_LQ_YHS_c3634/f1p5/3571	22.05964518	340.820989	-3.9495	1.33E-12	7.49E-11
i2_LQ_YHS_c50890/f1p6/3012	131.4526565	2301.211868	-4.1298	1.37E-12	7.74E-11
i1_LQ_YHS_c14221/f1p1/1234	97.2430504	1381.873761	-3.8289	1.40E-12	7.87E-11
i2_LQ_YHS_c13524/f1p3/2852	84.03806373	1128.176439	-3.7468	1.40E-12	7.87E-11
i2_LQ_YHS_c50664/f1p4/2770	4.085119479	147.1854541	-5.1711	1.40E-12	7.87E-11
i0_LQ_YHS_c2228/f1p0/875	137.5815265	3434.059366	-4.6416	1.44E-12	8.06E-11
i3_LQ_YHS_c6097/f1p0/3378	32.28826487	432.33017	-3.7431	1.46E-12	8.16E-11
i2_LQ_YHS_c50499/f1p0/2296	36.92554662	487.850991	-3.7237	1.47E-12	8.21E-11
i0_HQ_YHS_c383/f2p0/657	5220.10646	0.784522063	12.7	1.47E-12	8.22E-11
i2_LQ_YHS_c44265/f1p14/2031	100.5905861	1494.366722	-3.893	1.54E-12	8.58E-11
i1_LQ_YHS_c24874/f1p11/2019	6.517745707	178.2547691	-4.7734	1.55E-12	8.64E-11
i0_LQ_YHS_c1065/f1p0/978	94.53013648	7791.039587	-6.3649	1.55E-12	8.65E-11
i4_HQ_YHS_c2411/f2p0/4555	19.07151538	305.6380657	-4.0023	1.56E-12	8.69E-11
i3_LQ_YHS_c14823/f1p1/3035	29.53416012	852.1827084	-4.8507	1.61E-12	8.95E-11
i2_LQ_YHS_c9943/f1p1/2153	92.88693714	1569.547751	-4.0787	1.62E-12	8.99E-11
i3_LQ_YHS_c6515/f1p0/3110	14.9666783	261.1331488	-4.125	1.63E-12	9.04E-11
i5_LQ_YHS_c4249/f1p0/5273	4.085119479	144.9639202	-5.1492	1.63E-12	9.04E-11
i0_LQ_YHS_c2338/f1p8/772	91.85694737	1142.727855	-3.6369	1.69E-12	9.34E-11
i0_LQ_YHS_c609/f1p0/622	124.4537827	2327.646915	-4.2252	1.70E-12	9.39E-11
i2_LQ_YHS_c50274/f1p3/2243	3.4242172	134.3810465	-5.2944	1.77E-12	9.76E-11
i3_LQ_YHS_c18161/f1p0/3368	92.50336423	1269.104683	-3.7782	1.92E-12	1.06E-10
i4_LQ_YHS_c6392/f1p0/4864	18.77644546	302.7845387	-4.0113	1.96E-12	1.08E-10
i1_LQ_YHS_c23324/f1p0/1431	139.449889	3067.330777	-4.4592	2.01E-12	1.10E-10
i1_LQ_YHS_c35417/f1p0/1909	136.8765672	3005.632085	-4.4567	2.05E-12	1.13E-10
i2_LQ_YHS_c10697/f1p4/2137	24.51995216	2193.555655	-6.4832	2.05E-12	1.13E-10
i1_HQ_YHS_c17223/f2p0/1458	62.72498518	954.4304428	-3.9275	2.07E-12	1.13E-10
i5_LQ_YHS_c3427/f1p1/5510	33.50480717	442.7445614	-3.724	2.11E-12	1.15E-10
i4_LQ_YHS_c5004/f1p0/4509	5.243322752	172.5779187	-5.0406	2.19E-12	1.20E-10
i2_LQ_YHS_c22243/f1p0/2256	0	72.11296021	-Inf	2.22E-12	1.21E-10
i2_LQ_YHS_c24494/f1p0/2897	2.876998694	115.4145619	-5.3261	2.25E-12	1.23E-10
i2_HQ_YHS_c24174/f2p4/2398	42.38334675	517.3068233	-3.6095	2.26E-12	1.23E-10
i1_LQ_YHS_c26058/f1p0/1909	61.6244687	753.8457761	-3.6127	2.27E-12	1.24E-10

i1_LQ_YHS_c6095/f2p0/2035	79.80204764	995.5507292	-3.641	2.29E-12	1.25E-10
i3_LQ_YHS_c6087/f1p0/3529	10.43625288	632.7078304	-5.9219	2.30E-12	1.25E-10
i2_LQ_YHS_c34816/f1p0/2916	1.90638909	224.5574835	-6.8801	2.33E-12	1.26E-10
i1_LQ_YHS_c8484/f1p3/2006	4.629802076	150.7748086	-5.0253	2.37E-12	1.28E-10
i3_LQ_YHS_c9575/f1p0/3343	40.28393676	502.718718	-3.6415	2.49E-12	1.35E-10
i0_LQ_YHS_c1256/f1p0/590	0	116.2717121	-Inf	2.54E-12	1.37E-10
i1_LQ_YHS_c39557/f1p2/1318	11.87729651	219.1950579	-4.2059	2.56E-12	1.38E-10
i1_LQ_YHS_c3694/f1p17/1987	1186.205526	0	Inf	2.60E-12	1.40E-10
i1_LQ_YHS_c14484/f1p0/1070	138.879797	4504.37709	-5.0194	2.60E-12	1.40E-10
i2_LQ_YHS_c21811/f1p5/2595	88.29741999	1145.270317	-3.6972	2.61E-12	1.40E-10
i4_LQ_YHS_c13192/f1p3/4208	1.07887451	137.0689343	-6.9892	2.63E-12	1.41E-10
i2_LQ_YHS_c19745/f1p1/2512	29.50141593	645.9069922	-4.4525	2.66E-12	1.43E-10
i3_LQ_YHS_c4200/f1p1/3791	128.3093134	2.887731779	5.4735	2.72E-12	1.46E-10
i2_LQ_YHS_c38630/f1p27/2483	12.52769973	327.3649525	-4.7077	2.73E-12	1.46E-10
i2_HQ_YHS_c10357/f2p1/2421	107.7148898	1428.996517	-3.7297	2.75E-12	1.47E-10
i2_LQ_YHS_c12287/f1p0/2774	5.020907339	148.7846733	-4.8891	2.76E-12	1.48E-10
i3_HQ_YHS_c10866/f2p2/3764	129.5133449	5837.010817	-5.4941	2.79E-12	1.49E-10
i1_LQ_YHS_c14465/f1p0/1094	5.863526061	337.941465	-5.8489	2.82E-12	1.51E-10
i1_LQ_YHS_c18716/f1p3/1752	21.59095841	316.7853207	-3.875	2.83E-12	1.51E-10
i2_LQ_YHS_c39615/f1p2/2340	0.359624837	231.7361986	-9.3318	2.87E-12	1.53E-10
i4_LQ_YHS_c10823/f1p0/4716	32.36665179	425.9557755	-3.7181	2.89E-12	1.54E-10
i2_LQ_YHS_c52003/f1p6/2322	0	70.60019699	-Inf	3.02E-12	1.61E-10
i2_LQ_YHS_c27106/f1p2/2094	89.97376082	1179.759521	-3.7128	3.06E-12	1.62E-10
i2_LQ_YHS_c34298/f1p9/2988	138.1815287	2809.802413	-4.3458	3.08E-12	1.63E-10
i3_LQ_YHS_c8841/f1p0/3351	54.54426391	658.8156676	-3.5944	3.22E-12	1.70E-10
i2_LQ_YHS_c33918/f1p4/2931	8.987262853	1520.829952	-7.4028	3.33E-12	1.76E-10
i0_LQ_YHS_c2687/f1p0/717	1.623557107	769.2796001	-8.8882	3.35E-12	1.77E-10
i2_LQ_YHS_c41839/f1p4/2156	9.237817376	200.661236	-4.4411	3.35E-12	1.77E-10
i1_HQ_YHS_c29382/f2p0/1298	1084.142923	86.98685184	3.6396	3.39E-12	1.79E-10
i1_LQ_YHS_c23034/f1p1/1573	116.5194692	1708.66733	-3.8742	3.41E-12	1.80E-10
i1_LQ_YHS_c7025/f1p4/1922	385.1202061	8.522949343	5.4978	3.44E-12	1.81E-10
i2_LQ_YHS_c19141/f1p2/2787	80.48774771	959.1720842	-3.5749	3.51E-12	1.84E-10
i1_HQ_YHS_c9830/f2p1/1964	15.5113609	258.4661506	-4.0586	3.54E-12	1.86E-10
i0_LQ_YHS_c3021/f1p0/892	234.64287	7.999961374	4.8743	3.61E-12	1.89E-10
i2_LQ_YHS_c56321/f1p2/2981	122.214017	2.501592453	5.6104	3.62E-12	1.90E-10
i2_LQ_YHS_c58727/f1p1/2042	134.4033952	2307.367433	-4.1016	3.63E-12	1.90E-10
i1_LQ_YHS_c11714/f1p2/1733	123.4385392	2.539981251	5.6028	3.63E-12	1.90E-10
i3_HQ_YHS_c1771/f3p0/3593	38.29186657	470.4214811	-3.6188	3.77E-12	1.97E-10
i1_HQ_YHS_c14566/f2p0/1373	113.9237419	1516.570693	-3.7347	3.80E-12	1.98E-10
i3_LQ_YHS_c2619/f2p7/3266	11.12529427	415.4458117	-5.2227	3.80E-12	1.98E-10
i2_LQ_YHS_c14427/f1p5/1986	148.6972719	3383.661942	-4.5081	4.00E-12	2.08E-10
i2_HQ_YHS_c42050/f2p1/2411	40.12366841	493.7129873	-3.6211	4.03E-12	2.10E-10
i2_LQ_YHS_c6476/f6p0/2868	58.59884481	684.8232704	-3.5468	4.04E-12	2.10E-10
i4_LQ_YHS_c9022/f1p3/4723	37.37880733	508.0546819	-3.7647	4.11E-12	2.13E-10
i2_LQ_YHS_c35712/f1p0/2515	923.4974572	0	Inf	4.19E-12	2.17E-10
i1_LQ_YHS_c32939/f1p14/1630	202.4741662	1.82611508	6.7928	4.27E-12	2.21E-10
i1_HQ_YHS_c11190/f4p0/1555	106.4727275	1400.791154	-3.7177	4.42E-12	2.28E-10
i0_LQ_YHS_c3056/f1p2/699	41.47856421	908.5944656	-4.4532	4.45E-12	2.30E-10
i2_HQ_YHS_c20792/f2p3/2282	148.3139683	3426.772944	-4.5301	4.51E-12	2.33E-10
i3_LQ_YHS_c17173/f1p0/3865	4.116599905	135.360316	-5.0392	4.64E-12	2.39E-10
i2_HQ_YHS_c23479/f2p0/2175	26.98913064	361.9395757	-3.7453	4.74E-12	2.44E-10
i1_HQ_YHS_c9459/f2p12/1457	151.7650536	2728.416788	-4.1682	4.77E-12	2.45E-10
i2_LQ_YHS_c37384/f1p2/2225	4.619311392	1080.134889	-7.8693	4.88E-12	2.51E-10
i4_LQ_YHS_c13032/f1p0/4132	92.55344331	1120.792009	-3.5981	4.95E-12	2.54E-10
i2_HQ_YHS_c45261/f3p1/2641	221.8697331	11.31834298	4.293	5.04E-12	2.59E-10
i2_HQ_YHS_c32619/f2p17/2969	51.89382735	615.4654922	-3.568	5.06E-12	2.59E-10
i2_HQ_YHS_c19001/f2p6/2123	149.0694399	3211.385458	-4.4291	5.09E-12	2.60E-10
i2_LQ_YHS_c51019/f1p22/2809	97.02065174	1155.688236	-3.5743	5.13E-12	2.62E-10
i1_LQ_YHS_c27868/f1p3/1165	75.87891863	901.8586806	-3.5711	5.28E-12	2.69E-10
i1_LQ_YHS_c4049/f1p19/1731	7.615065676	401.9847242	-5.7221	5.34E-12	2.72E-10
i1_LQ_YHS_c10338/f1p0/1621	61.38174927	695.3668774	-3.5019	5.36E-12	2.73E-10
i2_LQ_YHS_c51550/f1p0/2503	37.30743334	449.8914764	-3.592	5.48E-12	2.79E-10
i1_LQ_YHS_c17635/f1p0/1373	85.57067406	995.9058804	-3.5408	5.53E-12	2.81E-10
i1_HQ_YHS_c37881/f2p12/1098	13523.73327	0.359993938	15.197	5.54E-12	2.81E-10
i1_LQ_YHS_c23491/f1p0/1773	0	68.33896894	-Inf	5.69E-12	2.88E-10
i2_LQ_YHS_c5010/f1p1/2291	62.75743257	690.6866496	-3.4602	5.77E-12	2.92E-10
i2_LQ_YHS_c26789/f1p11/2288	97.88281932	1110.522637	-3.504	5.84E-12	2.96E-10
i1_LQ_YHS_c6435/f1p1/1988	117.5255528	1689.418914	-3.8455	5.85E-12	2.96E-10
i3_LQ_YHS_c8500/f1p1/3493	2.451071687	116.0264116	-5.5649	5.92E-12	2.99E-10
i2_LQ_YHS_c52633/f1p0/2378	10.6310043	206.0761524	-4.2768	6.12E-12	3.09E-10
i2_LQ_YHS_c9353/f1p7/2893	18.75085901	276.4337348	-3.8819	6.17E-12	3.11E-10
i1_HQ_YHS_c19397/f2p1/1432	51.32796953	1807.283561	-5.1379	6.17E-12	3.11E-10
i1_HQ_YHS_c2210/f2p0/1503	71.54898542	7040.224958	-6.6205	6.21E-12	3.12E-10
i3_LQ_YHS_c11845/f1p0/3718	96.41260382	1.176783094	6.3563	6.21E-12	3.12E-10
i1_LQ_YHS_c37966/f1p4/1224	5.190860957	145.6400299	-4.8103	6.28E-12	3.15E-10
i2_LQ_YHS_c34327/f1p0/2670	0.544682597	128.1814872	-7.8786	6.43E-12	3.22E-10
i2_LQ_YHS_c55770/f1p18/3167	29.43973559	380.3594908	-3.6915	6.49E-12	3.25E-10
i0_LQ_YHS_c2339/f1p0/983	50.05039912	1589.722659	-4.9892	6.50E-12	3.25E-10
i2_HQ_YHS_c20921/f7p1/2703	97.01201962	1166.386833	-3.5877	6.63E-12	3.31E-10
i2_LQ_YHS_c20834/f1p1/2411	189.9103594	9.476667359	4.3248	6.79E-12	3.39E-10
i2_LQ_YHS_c6337/f1p1/2427	5.80772295	151.8299097	-4.7083	6.86E-12	3.42E-10
i2_LQ_YHS_c20058/f1p0/2780	16.91251096	248.031302	-3.8744	6.91E-12	3.44E-10
i2_HQ_YHS_c29730/f4p2/2243	19.0462676	888.5906907	-5.5439	6.92E-12	3.44E-10
i1_LQ_YHS_c11077/f1p0/1758	11.25454892	207.6678715	-4.2057	7.07E-12	3.52E-10
i2_LQ_YHS_c49111/f1p0/2216	17.10504839	256.2744335	-3.9052	7.19E-12	3.57E-10
i3_LQ_YHS_c17555/f1p0/3630	29.61349726	385.1217197	-3.701	7.20E-12	3.57E-10
i5_LQ_YHS_c4530/f1p0/5315	59.13050081	655.9054707	-3.4715	7.24E-12	3.59E-10
i1_LQ_YHS_c38743/f1p0/1072	149.1624642	2896.994796	-4.2796	7.42E-12	3.67E-10
i1_LQ_YHS_c13493/f1p2/1284	47.36769347	535.1207782	-3.4979	7.44E-12	3.68E-10

i3_LQ_YHS_c3605/f1p0/3272	17.85958654	262.6472113	-3.8784	7.50E-12	3.71E-10
i5_HQ_YHS_c662/f2p0/5454	102.7630144	1224.413743	-3.5747	7.57E-12	3.73E-10
i1_LQ_YHS_c20135/f1p0/1576	63.74026336	700.8500439	-3.4588	7.60E-12	3.74E-10
i2_LQ_YHS_c20204/f1p0/2884	33.69942214	418.0662767	-3.6329	7.60E-12	3.74E-10
i2_LQ_YHS_c11136/f6p14/2310	43.85331815	505.0147793	-3.5256	7.76E-12	3.82E-10
i0_LQ_YHS_c2016/f1p2/506	1.623557107	97.08544002	-5.902	7.93E-12	3.90E-10
i1_HQ_YHS_c4229/f2p5/2013	24.55650441	636.8971099	-4.6969	8.10E-12	3.98E-10
i3_LQ_YHS_c5991/f1p3/3740	143.5276975	5557.517303	-5.275	8.25E-12	4.05E-10
i6_LQ_YHS_c781/f1p0/6522	65.22405839	1499.462281	-4.5229	8.26E-12	4.05E-10
i1_LQ_YHS_c27869/f1p0/1097	10.10142584	268.2637489	-4.731	8.29E-12	4.06E-10
i1_HQ_YHS_c2772/f2p2/1782	387.6760482	31.00556755	3.6443	8.36E-12	4.09E-10
i2_LQ_YHS_c34375/f1p3/2174	127.8876851	1928.875447	-3.9148	8.36E-12	4.09E-10
i2_LQ_YHS_c34313/f1p10/2597	101.9472459	1235.316718	-3.599	8.48E-12	4.14E-10
i5_LQ_YHS_c3944/f1p4/5598	120.8282762	1663.891894	-3.7835	8.63E-12	4.21E-10
i2_LQ_YHS_c22085/f1p0/2747	36.94573933	432.6026087	-3.5496	8.74E-12	4.26E-10
i2_LQ_YHS_c14774/f1p2/2048	48.34512215	529.7866668	-3.454	8.77E-12	4.26E-10
i2_LQ_YHS_c18114/f1p3/2660	51.30478255	570.9584354	-3.4762	8.77E-12	4.26E-10
i0_LQ_YHS_c2255/f1p0/417	281.0660573	9.510593019	4.8852	8.87E-12	4.31E-10
i3_LQ_YHS_c12321/f1p1/3371	9.336397005	188.552369	-4.336	9.02E-12	4.37E-10
i1_LQ_YHS_c12144/f1p0/1719	28.37008799	360.1422512	-3.6661	9.15E-12	4.43E-10
i1_LQ_YHS_c7711/f1p4/1936	0	66.87952273	-Inf	9.15E-12	4.43E-10
i1_LQ_YHS_c28629/f1p7/1153	141.2944039	2209.218086	-3.9668	9.17E-12	4.44E-10
i2_HQ_YHS_c2253/f6p0/2169	0	238.6977199	-Inf	9.52E-12	4.60E-10
i1_HQ_YHS_c2471/f6p3/1780	2176.56884	15.52144528	7.1316	9.59E-12	4.63E-10
i1_LQ_YHS_c23407/f1p0/1569	11.46393774	280.4925196	-4.6128	9.63E-12	4.65E-10
i0_LQ_YHS_c2990/f1p0/816	4.691490787	136.7514379	-4.8654	9.82E-12	4.73E-10
i1_LQ_YHS_c18763/f1p10/1411	463.2937576	12.15349725	5.2525	1.01E-11	4.88E-10
i3_HQ_YHS_c4212/f2p3/3336	63.25470844	706.9080998	-3.4823	1.03E-11	4.93E-10
i2_HQ_YHS_c32624/f2p3/2664	6.299943752	152.2483568	-4.5949	1.05E-11	5.04E-10
i3_LQ_YHS_c9448/f1p0/3239	8.538285304	179.9161514	-4.3972	1.07E-11	5.15E-10
i3_LQ_YHS_c8097/f1p3/3245	12.78748117	414.6187081	-5.019	1.08E-11	5.16E-10
i3_LQ_YHS_c3338/f1p0/3727	46.26462434	515.0785007	-3.4768	1.11E-11	5.30E-10
i1_LQ_YHS_c28467/f1p0/2005	166.6161561	3551.015919	-4.4136	1.12E-11	5.35E-10
i2_LQ_YHS_c37596/f1p3/2854	48.80425171	863.371205	-4.1449	1.13E-11	5.38E-10
i4_LQ_YHS_c7266/f1p0/4335	139.9385818	6017.469953	-5.4263	1.12E-11	5.38E-10
i1_HQ_YHS_c29633/f7p0/1631	36.74509394	2759.937531	-6.2309	1.14E-11	5.44E-10
i1_LQ_YHS_c12942/f1p1/1541	137.6473368	2110.524299	-3.9386	1.14E-11	5.45E-10
i2_LQ_YHS_c13175/f1p0/2108	70.67297866	750.4427503	-3.4085	1.15E-11	5.46E-10
i5_LQ_YHS_c2126/f1p0/5127	41.87394426	475.0019174	-3.5038	1.17E-11	5.59E-10
i1_LQ_YHS_c33099/f1p3/1560	21.94263684	446.9093529	-4.3482	1.18E-11	5.62E-10
i2_LQ_YHS_c20200/f1p1/2830	8.716193696	331.8005625	-5.2505	1.20E-11	5.68E-10
i1_HQ_YHS_c1567/f6p0/1646	152.9665408	5904.952734	-5.2706	1.22E-11	5.79E-10
i3_LQ_YHS_c11711/f1p6/3592	14.49671101	328.4226187	-4.5018	1.24E-11	5.89E-10
i0_LQ_YHS_c1395/f1p1/684	135.4220302	6772.89349	-5.6442	1.25E-11	5.91E-10
i1_LQ_YHS_c33944/f1p189/1760	24.9681243	324.8465387	-3.7016	1.26E-11	5.94E-10
i2_HQ_YHS_c48514/f2p0/2150	61.93309775	687.1901898	-3.4719	1.27E-11	5.99E-10
i2_LQ_YHS_c22338/f1p1/2841	30.0343357	378.9725986	-3.6574	1.28E-11	6.03E-10
i1_LQ_YHS_c35559/f1p1/1487	25.7298202	322.5022509	-3.6478	1.28E-11	6.05E-10
i2_LQ_YHS_c11993/f1p2/2572	2.451071687	481.110941	-7.6168	1.29E-11	6.10E-10
i1_LQ_YHS_c27404/f1p0/1215	126.9037522	1628.661471	-3.6819	1.32E-11	6.23E-10
i0_LQ_YHS_c2486/f1p1/633	23.97112284	929.0074596	-5.2763	1.33E-11	6.25E-10
i2_LQ_YHS_c7509/f1p3/2773	4.110722679	121.5572476	-4.8861	1.34E-11	6.28E-10
i1_LQ_YHS_c26592/f1p0/1932	149.5700989	2383.374342	-3.9941	1.34E-11	6.28E-10
i2_HQ_YHS_c32640/f2p0/2662	74.18478457	834.5030059	-3.4917	1.34E-11	6.28E-10
i3_LQ_YHS_c8798/f1p0/3897	36.75987616	427.9942092	-3.5414	1.34E-11	6.28E-10
i2_LQ_YHS_c5771/f1p0/2304	115.326808	1395.944887	-3.5974	1.36E-11	6.35E-10
i2_LQ_YHS_c34564/f1p3/2910	3.23662353	111.0722399	-5.1009	1.36E-11	6.35E-10
i2_LQ_YHS_c36800/f1p2/2352	21.72529325	300.5703071	-3.7903	1.38E-11	6.44E-10
i1_HQ_YHS_c25003/f2p3/1568	987.3473596	92.56634173	3.415	1.42E-11	6.62E-10
i3_HQ_YHS_c16499/f5p0/3191	73.09368887	790.9096236	-3.4357	1.42E-11	6.63E-10
i1_LQ_YHS_c26646/f1p3/1724	168.3924935	4659.913633	-4.7904	1.46E-11	6.80E-10
i1_LQ_YHS_c25718/f1p2/1746	9.70317121	188.3923389	-4.2791	1.47E-11	6.82E-10
i5_LQ_YHS_c2165/f1p1/5299	12.41609351	214.7682186	-4.1125	1.48E-11	6.87E-10
i2_HQ_YHS_c2378/f6p0/2562	95.28484456	1002.466765	-3.3952	1.49E-11	6.91E-10
i2_LQ_YHS_c38013/f1p2/2556	42.15168768	492.9674035	-3.5478	1.49E-11	6.92E-10
i1_LQ_YHS_c32480/f1p0/1452	96.98246356	9354.791699	-6.5918	1.51E-11	6.98E-10
i1_LQ_YHS_c5399/f1p0/1768	159.0573686	2573.270955	-4.016	1.52E-11	7.03E-10
i2_HQ_YHS_c41202/f2p1/2480	26.68944726	570.1557532	-4.417	1.52E-11	7.03E-10
i1_LQ_YHS_c41593/f1p0/1622	20.2535746	284.3220446	-3.8113	1.53E-11	7.05E-10
i2_HQ_YHS_c15485/f18p0/2445	57.17751878	624.5130117	-3.4492	1.53E-11	7.05E-10
i3_LQ_YHS_c18497/f1p0/3226	5.431721829	160.7937416	-4.8877	1.55E-11	7.16E-10
i2_HQ_YHS_c12255/f5p4/2971	68.34304528	745.2273654	-3.4468	1.56E-11	7.17E-10
i0_LQ_YHS_c1442/f1p0/848	32.26887757	390.2879788	-3.5963	1.59E-11	7.30E-10
i2_HQ_YHS_c40877/f2p2/2682	61.36631483	644.7881128	-3.3933	1.59E-11	7.30E-10
i3_LQ_YHS_c11458/f1p0/3110	64.66366847	694.442707	-3.4248	1.59E-11	7.33E-10
i1_LQ_YHS_c18962/f1p1/1806	24.74282594	327.2409043	-3.7253	1.61E-11	7.37E-10
i1_LQ_YHS_c39158/f1p1/1331	20.14404593	273.2294526	-3.7617	1.61E-11	7.38E-10
i1_HQ_YHS_c1821/f10p0/1937	161.8267128	3210.921581	-4.3105	1.61E-11	7.38E-10
i2_LQ_YHS_c49618/f1p0/2844	3.976854572	124.6796148	-4.9705	1.62E-11	7.43E-10
i4_LQ_YHS_c10507/f1p21/4355	11.43833454	208.3295606	-4.1869	1.63E-11	7.47E-10
i1_HQ_YHS_c37589/f5p0/1263	171.7971295	3916.521873	-4.5108	1.67E-11	7.63E-10
i1_LQ_YHS_c17959/f1p2/1665	60.92052541	11865.49971	-7.6056	1.67E-11	7.63E-10
i3_LQ_YHS_c9363/f1p3/3372	5.649532158	192.6520765	-5.0917	1.68E-11	7.65E-10
i3_LQ_YHS_c14604/f1p0/3070	44.6350452	511.118796	-3.5174	1.69E-11	7.69E-10
i1_LQ_YHS_c25208/f1p0/1665	35.36749465	404.0931036	-3.5142	1.73E-11	7.86E-10
i1_LQ_YHS_c26170/f1p9/1917	26.69072778	332.4630049	-3.6388	1.77E-11	8.03E-10
i1_HQ_YHS_c15102/f4p0/1802	72.65566037	790.9999126	-3.4445	1.87E-11	8.49E-10
i4_LQ_YHS_c13461/f1p2/4112	170.0689456	3908.318899	-4.5224	1.89E-11	8.60E-10
i1_LQ_YHS_c7954/f1p4/1584	91.96071851	1.144516001	6.3282	1.91E-11	8.68E-10

i2_LQ_YHS_c56224/f1p5/2070	58.63606602	635.0097058	-3.4369	1.93E-11	8.73E-10
i2_LQ_YHS_c53789/f1p0/2421	84.69498922	880.6703401	-3.3783	1.94E-11	8.76E-10
i2_HQ_YHS_c61646/f17p1/2327	165.6557308	3345.57958	-4.336	1.95E-11	8.80E-10
i3_LQ_YHS_c12604/f1p0/3669	108.6813191	1739.098636	-4.0002	1.98E-11	8.95E-10
i3_LQ_YHS_c3818/f1p0/3536	89.4375081	992.7402597	-3.4725	2.01E-11	9.06E-10
i2_LQ_YHS_c39689/f1p0/2354	2.178730389	400.8609373	-7.5235	2.02E-11	9.11E-10
i2_LQ_YHS_c11883/f1p2/2282	74.48526498	804.9672743	-3.4339	2.05E-11	9.24E-10
i2_HQ_YHS_c22994/f2p3/2473	60.09362237	632.8531779	-3.3966	2.07E-11	9.32E-10
i1_LQ_YHS_c35593/f1p0/1963	162.5429694	4.436752222	5.1952	2.08E-11	9.33E-10
i0_HQ_YHS_c412/f2p0/789	167.7924984	3455.757984	-4.3643	2.08E-11	9.34E-10
i2_LQ_YHS_c62594/f1p3/2675	0	82.28189059	-Inf	2.10E-11	9.40E-10
i0_HQ_YHS_c187/f5p0/684	90.24024164	10137.04636	-6.8117	2.10E-11	9.42E-10
i2_LQ_YHS_c19575/f1p3/2599	1.361706493	93.08206199	-6.095	2.11E-11	9.45E-10
i2_HQ_YHS_c61954/f103p13/2521	169.3110662	3421.219815	-4.3368	2.12E-11	9.50E-10
i0_LQ_YHS_c1071/f1p0/808	173.0631142	3864.30726	-4.4808	2.13E-11	9.51E-10
i4_LQ_YHS_c9186/f1p11/4585	170.9790106	3269.767781	-4.2573	2.17E-11	9.71E-10
i2_LQ_YHS_c37686/f1p1/3016	75.02916729	771.6695961	-3.3625	2.23E-11	9.93E-10
i1_LQ_YHS_c8339/f1p2/1630	44.30738427	471.3314358	-3.4111	2.23E-11	9.96E-10
i1_LQ_YHS_c8599/f1p0/1986	4.085119479	129.1559414	-4.9826	2.24E-11	9.97E-10
i0_LQ_YHS_c1489/f1p0/774	0	63.54056421	-Inf	2.25E-11	1.00E-09
i3_LQ_YHS_c2832/f1p0/3686	305.731631	8.279860583	5.2065	2.35E-11	1.04E-09
i1_LQ_YHS_c38627/f1p0/1123	371.1324104	2.578370049	7.1693	2.41E-11	1.07E-09
i2_HQ_YHS_c11016/f5p1/2826	15.54952395	234.0661845	-3.912	2.42E-11	1.07E-09
i3_LQ_YHS_c17107/f1p0/3960	9.532412185	186.5473055	-4.2906	2.43E-11	1.08E-09
i1_LQ_YHS_c19200/f1p0/1457	165.0752607	2661.107453	-4.0108	2.43E-11	1.08E-09
i2_HQ_YHS_c60756/f2p2/2185	86.68656755	937.7248879	-3.4353	2.49E-11	1.10E-09
i1_LQ_YHS_c44002/f1p0/1068	0	62.73274088	-Inf	2.51E-11	1.11E-09
i1_LQ_YHS_c22752/f1p6/1693	33.65538186	391.1858879	-3.5389	2.55E-11	1.13E-09
i0_HQ_YHS_c269/f3p0/555	122.6639687	8297.808532	-6.0799	2.56E-11	1.13E-09
i3_LQ_YHS_c9643/f1p1/3601	7.882793516	169.1628556	-4.4236	2.59E-11	1.14E-09
i3_LQ_YHS_c14330/f1p1/3019	9.957872456	183.4266709	-4.2032	2.62E-11	1.16E-09
i2_LQ_YHS_c11080/f1p1/3077	57.27370731	588.3945427	-3.3608	2.67E-11	1.17E-09
i3_LQ_YHS_c12688/f1p1/3478	151.4582125	2020.738631	-3.7379	2.68E-11	1.18E-09
i2_LQ_YHS_c17953/f1p1/2067	12.24613989	204.4504741	-4.0614	2.70E-11	1.19E-09
i3_LQ_YHS_c19256/f1p5/3361	48.73065536	531.3170848	-3.4467	2.74E-11	1.20E-09
i4_LQ_YHS_c8799/f1p0/4537	3.81277818	125.328354	-5.0387	2.74E-11	1.20E-09
i0_LQ_YHS_c449/f2p0/902	29.33575384	342.1740765	-3.544	2.78E-11	1.22E-09
i2_HQ_YHS_c60058/f2p2/2213	102.336629	1082.901264	-3.4035	2.83E-11	1.24E-09
i4_LQ_YHS_c7222/f1p0/4996	117.1599071	1254.70244	-3.4208	2.87E-11	1.25E-09
i0_LQ_YHS_c814/f1p0/794	136.7988409	7557.696096	-5.7878	2.87E-11	1.26E-09
i2_LQ_YHS_c23647/f1p1/2154	16.91630227	254.8679511	-3.9133	2.88E-11	1.26E-09
i2_LQ_YHS_c34045/f1p1/2383	16.43411379	243.878281	-3.8914	2.90E-11	1.27E-09
i2_HQ_YHS_c60347/f2p0/2089	59.13097592	596.9472336	-3.3356	3.04E-11	1.33E-09
i2_LQ_YHS_c8632/f1p0/2573	23.75078498	493.5658664	-4.3772	3.06E-11	1.33E-09
i1_HQ_YHS_c3766/f13p20/1928	72.77997126	768.1741185	-3.3998	3.11E-11	1.35E-09
i2_LQ_YHS_c24594/f1p1/3023	104.2413905	1159.138171	-3.4751	3.14E-11	1.36E-09
i1_LQ_YHS_c32637/f1p0/1734	45.7891185	476.4478137	-3.3792	3.14E-11	1.37E-09
i1_HQ_YHS_c3948/f2p0/1473	118.1263532	1309.067202	-3.4701	3.15E-11	1.37E-09
i1_LQ_YHS_c31889/f1p0/1813	0.991590972	81.61075659	-6.3629	3.21E-11	1.39E-09
i1_LQ_YHS_c33581/f1p0/1901	816.6236808	38.60813176	4.4027	3.25E-11	1.41E-09
i3_LQ_YHS_c2744/f1p0/3126	25.75159022	328.4924878	-3.6731	3.29E-11	1.42E-09
i1_LQ_YHS_c3353/f1p4/1750	208.519648	12.52186424	4.0577	3.33E-11	1.44E-09
i2_LQ_YHS_c55437/f1p1/2651	5.822827092	141.5943938	-4.6039	3.34E-11	1.44E-09
i2_LQ_YHS_c39674/f1p2/2251	82.29575103	868.2070461	-3.3991	3.35E-11	1.44E-09
i2_LQ_YHS_c55038/f1p2/2770	64.8803766	622.8897392	-3.2631	3.37E-11	1.45E-09
i1_LQ_YHS_c24741/f1p1/1648	154.1196053	6486.192708	-5.3952	3.39E-11	1.46E-09
i4_LQ_YHS_c14043/f1p1/4087	3.646632613	123.9996743	-5.0876	3.39E-11	1.46E-09
i1_LQ_YHS_c43732/f1p0/1023	115.675765	1224.592405	-3.4041	3.44E-11	1.48E-09
i2_LQ_YHS_c55075/f1p1/2430	0	61.62941825	-Inf	3.46E-11	1.49E-09
i1_HQ_YHS_c17290/f2p0/1889	84.79179648	897.5839764	-3.4041	3.47E-11	1.49E-09
i1_HQ_YHS_c40943/f31p1/1847	170.9947502	5032.469799	-4.8792	3.62E-11	1.55E-09
i2_LQ_YHS_c55300/f1p0/2282	11.17442313	231.5615155	-4.3731	3.62E-11	1.55E-09
i4_LQ_YHS_c4269/f1p0/4556	24.00006736	294.6291647	-3.6178	3.67E-11	1.57E-09
i0_LQ_YHS_c2550/f1p0/1084	33.12660881	507.7625799	-3.9381	3.69E-11	1.58E-09
i5_LQ_YHS_c926/f1p4/5300	17.12602976	241.3011349	-3.8166	3.69E-11	1.58E-09
i4_LQ_YHS_c11216/f1p2/4773	1.634047791	94.3334911	-5.8512	3.80E-11	1.62E-09
i4_LQ_YHS_c6385/f1p0/4616	43.29112357	462.6831269	-3.4179	3.85E-11	1.64E-09
i2_LQ_YHS_c41162/f1p1/2742	44.5410874	468.0412593	-3.3934	3.90E-11	1.66E-09
i2_LQ_YHS_c12449/f1p2/2138	35.37625483	384.9565139	-3.4438	3.92E-11	1.67E-09
i1_LQ_YHS_c28737/f1p2/1302	6.599152019	425.8446293	-6.0119	3.98E-11	1.69E-09
i0_LQ_YHS_c2784/f1p2/831	146.7896275	1857.119203	-3.6612	4.01E-11	1.70E-09
i2_LQ_YHS_c20216/f1p0/2363	6.263849867	149.1247238	-4.5733	4.04E-11	1.72E-09
i4_LQ_YHS_c12011/f1p0/4396	7.897897659	166.1265047	-4.3947	4.10E-11	1.74E-09
i1_LQ_YHS_c23444/f1p7/1763	29.21905905	340.1887971	-3.5414	4.12E-11	1.75E-09
i2_LQ_YHS_c53044/f1p0/2084	9.94864554	265.6137721	-4.7387	4.22E-11	1.79E-09
i1_HQ_YHS_c29954/f3p0/1372	120.0225652	1324.471283	-3.464	4.23E-11	1.79E-09
i1_LQ_YHS_c5884/f1p0/1639	128.0145989	1400.617309	-3.4517	4.29E-11	1.82E-09
i1_LQ_YHS_c38765/f1p3/1357	26.73062971	315.7898561	-3.5624	4.31E-11	1.82E-09
i1_HQ_YHS_c12525/f2p1/1584	1.089365194	84.71618495	-6.2811	4.33E-11	1.83E-09
i2_LQ_YHS_c54877/f1p0/2309	6.613450755	151.2885171	-4.5158	4.38E-11	1.85E-09
i4_LQ_YHS_c3689/f1p0/4302	47.89706132	503.7344854	-3.3947	4.41E-11	1.86E-09
i1_LQ_YHS_c32511/f1p4/1920	515.4102314	50.6686981	3.3466	4.44E-11	1.87E-09
i4_LQ_YHS_c11242/f1p0/4408	6.79804178	152.3699982	-4.4863	4.48E-11	1.88E-09
i4_LQ_YHS_c7666/f1p3/4644	30.64325129	354.8552572	-3.5336	4.52E-11	1.90E-09
i1_HQ_YHS_c1064/f5p0/1732	171.7744165	5415.88263	-4.9786	4.53E-11	1.90E-09
i2_LQ_YHS_c3956/f1p2/2568	7.712042866	156.7378728	-4.3451	4.58E-11	1.92E-09
i3_LQ_YHS_c8627/f1p0/3162	36.68596626	392.4930446	-3.4194	4.58E-11	1.92E-09
i0_LQ_YHS_c2830/f1p2/672	187.5969435	4262.222401	-4.5059	4.65E-11	1.95E-09
i1_LQ_YHS_c28317/f1p0/1250	139.9692915	1594.888544	-3.5103	4.71E-11	1.97E-09

i2_LQ_YHS_c44926/f1p3/2047	4.902143374	133.6472358	-4.7689	4.75E-11	1.99E-09
i2_LQ_YHS_c41894/f1p1/2347	111.0190792	1137.70087	-3.3572	4.80E-11	2.01E-09
i0_LQ_YHS_c2425/f1p6/626	16.59183784	759.7524252	-5.517	4.88E-11	2.04E-09
i1_HQ_YHS_c41026/f111p0/1681	167.2150549	6131.50129	-5.1965	4.91E-11	2.05E-09
i4_LQ_YHS_c10766/f1p32/4210	21.54438221	270.4496196	-3.65	5.15E-11	2.15E-09
i8_LQ_YHS_c118/f1p0/8264	148.5619701	7300.223777	-5.6188	5.16E-11	2.15E-09
i3_LQ_YHS_c19142/f1p4/3423	126.0466576	1354.026631	-3.4252	5.29E-11	2.20E-09
i2_LQ_YHS_c38242/f1p8/2301	92.7878154	944.6275708	-3.3477	5.33E-11	2.22E-09
i2_LQ_YHS_c38277/f1p1/2639	3.182084187	111.7526534	-5.1342	5.35E-11	2.22E-09
i3_LQ_YHS_c14927/f1p1/3070	4.357460777	126.3823498	-4.8582	5.40E-11	2.24E-09
i3_LQ_YHS_c4199/f1p0/3724	7.604574992	155.2546128	-4.3516	5.44E-11	2.25E-09
i5_LQ_YHS_c2208/f1p2/5160	43.6758765	451.8343818	-3.3709	5.45E-11	2.26E-09
i1_LQ_YHS_c14619/f1p0/1160	83.80831347	826.6346482	-3.3021	5.47E-11	2.26E-09
i5_LQ_YHS_c3679/f1p3/5533	182.4898465	4904.416365	-4.7482	5.52E-11	2.28E-09
i1_LQ_YHS_c28287/f1p0/1236	72.75711458	1494.985975	-4.3609	5.65E-11	2.33E-09
i1_LQ_YHS_c32917/f1p9/1536	93.6206998	928.4831807	-3.31	5.65E-11	2.33E-09
i2_LQ_YHS_c12299/f1p3/2328	26.65971408	320.9069834	-3.5894	5.69E-11	2.35E-09
i2_LQ_YHS_c6888/f1p6/2661	1.634047791	92.79335739	-5.8275	5.85E-11	2.41E-09
i2_LQ_YHS_c10676/f1p4/2133	82.92025425	805.1029374	-3.2794	5.93E-11	2.44E-09
i2_LQ_YHS_c3336/f2p1/2487	0	99.10697554	-Inf	5.97E-11	2.46E-09
i1_HQ_YHS_c16997/f2p3/1437	68.26752457	695.5280942	-3.3488	6.07E-11	2.49E-09
i1_LQ_YHS_c7332/f1p0/1655	629.7984954	26.98110005	4.5449	6.06E-11	2.49E-09
i2_LQ_YHS_c9848/f1p4/2611	10.07662805	745.9584243	-6.21	6.09E-11	2.50E-09
i1_LQ_YHS_c18627/f1p1/1584	33.84251717	368.892609	-3.4463	6.10E-11	2.50E-09
i1_LQ_YHS_c4524/f1p1/1887	153.4162496	1920.667822	-3.6461	6.21E-11	2.54E-09
i3_LQ_YHS_c11040/f1p1/3293	43.75266935	445.9159479	-3.3493	6.31E-11	2.58E-09
i1_LQ_YHS_c22431/f1p0/1552	12.92803191	198.6372146	-3.9416	6.36E-11	2.60E-09
i1_LQ_YHS_c32753/f1p0/1509	99.54369221	1008.273157	-3.3404	6.41E-11	2.62E-09
i1_LQ_YHS_c19009/f1p22/1802	69.07580503	682.4470315	-3.3045	6.50E-11	2.65E-09
i5_LQ_YHS_c2166/f1p2/5344	98.45864368	0	Inf	6.49E-11	2.65E-09
i2_LQ_YHS_c26329/f1p3/2734	65.40977674	658.6141711	-3.3319	6.50E-11	2.65E-09
i1_LQ_YHS_c26376/f1p1/1733	87.54081266	886.4864795	-3.3401	6.65E-11	2.71E-09
i1_LQ_YHS_c5461/f1p9/1855	16.42696442	230.4863094	-3.8105	6.71E-11	2.73E-09
i1_LQ_YHS_c17447/f1p3/1916	9.010796879	161.1629337	-4.1607	6.73E-11	2.73E-09
i3_LQ_YHS_c6407/f1p9/3383	8.928118425	167.9918409	-4.2339	6.83E-11	2.77E-09
i2_HQ_YHS_c38048/f3p4/2185	143.7387223	1710.792591	-3.5731	6.88E-11	2.79E-09
i1_LQ_YHS_c29005/f1p0/1168	109.9767893	1178.593283	-3.4218	7.01E-11	2.84E-09
i1_LQ_YHS_c18225/f1p0/1804	45.28544842	14422.96168	-8.3151	7.03E-11	2.85E-09
i0_LQ_YHS_c1825/f1p0/518	74.60976973	749.3472531	-3.3282	7.04E-11	2.85E-09
i3_LQ_YHS_c5074/f1p7/3778	47.52174592	472.0525697	-3.3123	7.12E-11	2.88E-09
i1_HQ_YHS_c3768/f2p16/1693	22.68112064	796.5248224	-5.1342	7.38E-11	2.98E-09
i2_LQ_YHS_c20101/f1p5/2680	187.1750268	5360.61337	-4.8399	7.51E-11	3.03E-09
i1_LQ_YHS_c7468/f1p4/1857	10.80177169	169.7077258	-3.9737	7.59E-11	3.06E-09
i3_LQ_YHS_c7729/f1p0/3650	10.15596518	172.1498806	-4.0833	7.76E-11	3.13E-09
i1_LQ_YHS_c35953/f1p0/1368	125.5627051	1318.818526	-3.3928	7.81E-11	3.14E-09
i2_LQ_YHS_c23349/f1p0/2450	10.20461893	173.9712128	-4.0916	7.85E-11	3.16E-09
i1_HQ_YHS_c31141/f8p0/1805	185.9671777	4926.542418	-4.7275	8.08E-11	3.24E-09
i1_LQ_YHS_c12117/f1p1/1754	9.268025661	174.009089	-4.2308	8.06E-11	3.24E-09
i2_LQ_YHS_c58204/f1p1/2072	27.25226177	407.6338878	-3.9028	8.08E-11	3.24E-09
i3_HQ_YHS_c8247/f2p0/3228	186.668305	2974.532831	-3.9941	8.17E-11	3.28E-09
i1_LQ_YHS_c33323/f1p3/1687	18.58852149	245.0822334	-3.7208	8.18E-11	3.28E-09
i1_LQ_YHS_c20203/f1p0/1694	243.1571139	18.18543899	3.741	8.61E-11	3.45E-09
i3_LQ_YHS_c2459/f1p3/3556	61.94752455	1012.861872	-4.0312	8.64E-11	3.46E-09
i2_LQ_YHS_c7719/f1p4/2561	26.20311206	1475.221939	-5.8151	8.66E-11	3.46E-09
i3_LQ_YHS_c12966/f1p0/3351	39.61922643	407.7011848	-3.3632	8.66E-11	3.46E-09
i2_LQ_YHS_c64826/f1p0/2024	140.7805985	1596.395423	-3.5033	8.80E-11	3.50E-09
i3_HQ_YHS_c3731/f2p3/4009	6.385149742	147.4520441	-4.5294	8.79E-11	3.50E-09
i3_LQ_YHS_c6398/f1p1/3277	85.68054141	2701.207351	-4.9785	8.80E-11	3.50E-09
i1_LQ_YHS_c35701/f1p0/1560	7.059892395	145.6940199	-4.3672	8.85E-11	3.52E-09
i1_LQ_YHS_c34528/f1p21/1483	196.3033694	3888.59041	-4.3081	9.10E-11	3.62E-09
i1_HQ_YHS_c25017/f2p0/1673	38.86228043	406.7056399	-3.3875	9.11E-11	3.62E-09
i1_LQ_YHS_c22327/f1p2/1562	145.7532531	1.852260467	6.2981	9.16E-11	3.64E-09
i3_LQ_YHS_c14549/f1p0/3064	63.13373887	729.7368066	-3.5309	9.39E-11	3.72E-09
i2_LQ_YHS_c41761/f1p0/3000	29.63401189	340.9428639	-3.5242	9.44E-11	3.74E-09
i1_LQ_YHS_c39075/f1p2/1133	115.0954469	1139.76833	-3.3078	9.50E-11	3.76E-09
i1_HQ_YHS_c17025/f2p0/1549	98.94307007	977.9180243	-3.305	9.91E-11	3.92E-09
i2_LQ_YHS_c14351/f1p1/2056	82.03036524	791.0029879	-3.2695	9.97E-11	3.94E-09
i3_LQ_YHS_c11911/f1p0/3557	9.424952685	167.398391	-4.1507	1.01E-10	3.98E-09
i2_LQ_YHS_c12504/f1p0/2547	15.68291695	225.7889296	-3.8477	1.01E-10	3.99E-09
i2_LQ_YHS_c41433/f1p2/2449	71.77283453	664.0322491	-3.2097	1.01E-10	3.99E-09
i1_HQ_YHS_c8058/f2p0/1903	72.19874479	683.0360556	-3.2419	1.02E-10	4.01E-09
i1_LQ_YHS_c13744/f1p0/1262	133.0943276	8922.328654	-6.0669	1.02E-10	4.02E-09
i5_LQ_YHS_c862/f1p0/5100	35.16941029	361.4883907	-3.3616	1.03E-10	4.07E-09
i3_LQ_YHS_c13608/f1p11/3717	4.327252492	123.0366779	-4.8295	1.07E-10	4.19E-09
i1_LQ_YHS_c34469/f1p0/1658	188.9805732	2918.00525	-3.9487	1.07E-10	4.22E-09
i0_LQ_YHS_c969/f1p0/835	144.2192928	5.073840797	4.829	1.12E-10	4.38E-09
i2_HQ_YHS_c22479/f3p0/2121	49.08661696	476.8920134	-3.2803	1.12E-10	4.40E-09
i1_LQ_YHS_c11561/f1p0/1852	26.42887716	304.5327313	-3.5264	1.13E-10	4.42E-09
i2_LQ_YHS_c18793/f1p9/2828	54.03950837	497.8413261	-3.2036	1.13E-10	4.43E-09
i3_LQ_YHS_c5428/f1p6/3419	23.3462977	291.8243718	-3.6438	1.15E-10	4.48E-09
i0_HQ_YHS_c479/f2p0/683	139.990152	1575.930172	-3.4928	1.15E-10	4.49E-09
i1_LQ_YHS_c5378/f1p0/1565	54.66891347	522.4684661	-3.2566	1.16E-10	4.55E-09
i2_HQ_YHS_c17391/f2p8/2372	194.2108108	4536.763386	-4.546	1.18E-10	4.62E-09
i2_HQ_YHS_c60421/f2p1/2086	138.1770517	1560.235849	-3.4972	1.19E-10	4.63E-09
i2_LQ_YHS_c63454/f1p0/2285	3.268095583	283.8248081	-6.4404	1.20E-10	4.67E-09
i0_HQ_YHS_c388/f2p0/555	96.55528116	11152.0492	-6.8517	1.21E-10	4.70E-09
i5_LQ_YHS_c3729/f1p1/5608	24.77972523	290.2409933	-3.55	1.23E-10	4.79E-09
i2_LQ_YHS_c38912/f1p6/2712	143.5388165	1629.040572	-3.5045	1.25E-10	4.86E-09
i0_LQ_YHS_c416/f2p0/608	118.709005	1167.522685	-3.2979	1.28E-10	4.98E-09

i1_LQ_YHS_c25398/f1p0/1628	180.6812412	10.80254251	4.064	1.28E-10	4.98E-09
i2_LQ_YHS_c18054/f1p35/2345	7140.704925	0	Inf	1.30E-10	5.04E-09
i1_LQ_YHS_c19963/f1p0/1597	0.272341299	65.28871614	-7.9053	1.31E-10	5.06E-09
i2_LQ_YHS_c35555/f1p2/2724	42.16392561	428.7338675	-3.346	1.31E-10	5.06E-09
i1_LQ_YHS_c6200/f1p0/1607	6.217273672	165.1980256	-4.7318	1.31E-10	5.07E-09
i2_HQ_YHS_c17302/f3p1/2133	57.1640087	565.4090426	-3.3061	1.34E-10	5.19E-09
i3_LQ_YHS_c8968/f1p4/3633	32.8388247	350.7703643	-3.4171	1.35E-10	5.20E-09
i2_HQ_YHS_c11293/f2p3/2383	853.3392486	4.327707532	7.6234	1.35E-10	5.20E-09
i2_LQ_YHS_c8368/f1p1/2253	98.57483824	930.273672	-3.2384	1.35E-10	5.20E-09
i4_LQ_YHS_c7396/f1p3/4977	4.629802076	125.4690731	-4.7602	1.35E-10	5.20E-09
i3_LQ_YHS_c2955/f1p1/3429	19.44370845	241.8664216	-3.6368	1.38E-10	5.32E-09
i1_HQ_YHS_c20826/f2p90/1882	183.9699866	6276.055815	-5.0923	1.41E-10	5.44E-09
i1_LQ_YHS_c18406/f1p0/2032	202.444713	4661.332313	-4.5251	1.42E-10	5.46E-09
i1_LQ_YHS_c14557/f1p0/1317	137.4638743	1391.597323	-3.3396	1.43E-10	5.49E-09
i1_LQ_YHS_c6569/f1p0/1420	62.2189575	581.3073812	-3.2239	1.43E-10	5.50E-09
i1_HQ_YHS_c2802/f3p3/1828	122.6585331	1299.496614	-3.4052	1.44E-10	5.54E-09
i2_LQ_YHS_c20545/f1p3/2852	0.817023896	164.3575831	-7.6522	1.45E-10	5.56E-09
i4_LQ_YHS_c13365/f1p1/4168	55.27034103	516.131785	-3.2232	1.46E-10	5.59E-09
i3_LQ_YHS_c4403/f1p0/3157	41.59953379	864.2651999	-4.3768	1.48E-10	5.65E-09
i4_HQ_YHS_c2348/f2p0/4601	188.8290806	5769.451327	-4.9333	1.48E-10	5.67E-09
i1_LQ_YHS_c6978/f1p2/1797	645.7340971	4.778381044	7.0783	1.50E-10	5.74E-09
i3_LQ_YHS_c5019/f1p3/3678	21.33371288	462.4947358	-4.4382	1.51E-10	5.76E-09
i3_LQ_YHS_c14420/f1p5/3054	109.6457786	3419.782819	-4.963	1.52E-10	5.81E-09
i2_LQ_YHS_c53399/f1p5/2535	119.9586553	1117.01478	-3.219	1.54E-10	5.87E-09
i2_LQ_YHS_c12667/f1p0/2607	8.743060665	669.9968253	-6.2599	1.56E-10	5.93E-09
i4_LQ_YHS_c13287/f1p1/4091	213.7452649	5.671947797	5.2359	1.56E-10	5.95E-09
i2_LQ_YHS_c25786/f1p5/2495	56.96179437	756.5174216	-3.7313	1.58E-10	6.02E-09
i2_LQ_YHS_c28993/f1p0/2031	15.04046013	214.5606734	-3.8345	1.62E-10	6.16E-09
i2_LQ_YHS_c13681/f1p6/2646	344.0944394	7.559872706	5.5083	1.64E-10	6.21E-09
i2_LQ_YHS_c64728/f1p0/2029	49.91446183	481.7296739	-3.2707	1.64E-10	6.22E-09
i1_LQ_YHS_c29203/f12p0/1776	189.8166613	2860.49195	-3.9136	1.70E-10	6.46E-09
i2_LQ_YHS_c3190/f1p8/2422	27.50647116	412.9593079	-3.9082	1.72E-10	6.52E-09
i1_LQ_YHS_c36542/f1p0/1585	16.1692689	284.316118	-4.1362	1.73E-10	6.55E-09
i2_LQ_YHS_c19213/f1p7/2954	0	56.64953461	-Inf	1.74E-10	6.59E-09
i2_LQ_YHS_c23940/f1p1/2525	12.80004103	197.5710556	-3.9482	1.75E-10	6.63E-09
i2_HQ_YHS_c61633/f4p0/2396	4.31803395	507.5713176	-6.8771	1.76E-10	6.65E-09
i2_LQ_YHS_c13253/f1p14/2355	34.50434454	368.7735723	-3.4179	1.77E-10	6.67E-09
i3_LQ_YHS_c18840/f1p1/3220	50.4058856	479.3819492	-3.2495	1.77E-10	6.67E-09
i2_LQ_YHS_c52973/f1p23/2789	118.123487	1112.872059	-3.2359	1.77E-10	6.67E-09
i1_LQ_YHS_c12427/f1p3/1650	128.3668877	1345.054191	-3.3893	1.78E-10	6.70E-09
i2_LQ_YHS_c41656/f1p2/2103	94.42631629	960.5394641	-3.3466	1.84E-10	6.91E-09
i2_HQ_YHS_c22829/f2p2/2449	64.36286614	590.8403679	-3.1985	1.89E-10	7.10E-09
i2_LQ_YHS_c8695/f1p25/2261	187.7385139	2689.164358	-3.8404	1.90E-10	7.14E-09
i4_LQ_YHS_c4176/f1p0/4817	51.50909117	483.8001386	-3.2315	1.91E-10	7.16E-09
i2_LQ_YHS_c11472/f1p4/2509	58.73813178	541.8765375	-3.2056	1.91E-10	7.17E-09
i1_LQ_YHS_c24173/f1p0/1963	13998.73362	0	Inf	1.95E-10	7.29E-09
i1_LQ_YHS_c25100/f1p13/1579	139.8214717	1358.367837	-3.2802	1.99E-10	7.43E-09
i2_LQ_YHS_c22469/f1p4/2561	43.15839116	424.3871219	-3.2977	2.01E-10	7.51E-09
i1_LQ_YHS_c24287/f1p3/1508	4.72884844	121.6245457	-4.6848	2.02E-10	7.55E-09
i1_LQ_YHS_c19687/f1p11/1627	19.25022918	235.8366521	-3.6148	2.06E-10	7.71E-09
i1_LQ_YHS_c10744/f1p1/1585	139.0262011	1401.76059	-3.3338	2.08E-10	7.78E-09
i0_HQ_YHS_c457/f2p0/606	140.5642542	9300.29551	-6.048	2.09E-10	7.80E-09
i0_LQ_YHS_c1658/f1p1/913	0	56.17097693	-Inf	2.10E-10	7.82E-09
i2_LQ_YHS_c52944/f1p1/2460	170.9445192	2079.464845	-3.6046	2.11E-10	7.87E-09
i2_HQ_YHS_c60097/f11p4/2410	111.0942684	1120.208469	-3.3339	2.13E-10	7.91E-09
i0_LQ_YHS_c1746/f1p0/647	164.211371	1941.796358	-3.5638	2.14E-10	7.93E-09
i1_LQ_YHS_c33746/f1p3/1762	146.6484484	8977.783603	-5.9359	2.13E-10	7.93E-09
i1_LQ_YHS_c42277/f1p0/1336	78.19646088	881.8793952	-3.4954	2.14E-10	7.95E-09
i1_LQ_YHS_c19560/f1p1/1491	132.2319829	1402.275244	-3.4066	2.16E-10	8.00E-09
i2_LQ_YHS_c36028/f1p6/2445	61.69894463	1291.525	-4.3877	2.20E-10	8.15E-09
i2_LQ_YHS_c37526/f1p6/2675	102.6183002	1034.121736	-3.333	2.24E-10	8.30E-09
i4_LQ_YHS_c6351/f1p1/4450	9.916368081	229.0375667	-4.5296	2.25E-10	8.31E-09
i2_LQ_YHS_c21308/f1p0/2353	136.8089773	6.246160752	4.453	2.25E-10	8.31E-09
i1_HQ_YHS_c3911/f5p3/1618	1832.362442	165.5500416	3.4684	2.26E-10	8.34E-09
i3_LQ_YHS_c3676/f1p4/3145	22.56952279	445.4606086	-4.3029	2.28E-10	8.42E-09
i3_LQ_YHS_c17674/f1p0/3619	8.791714408	158.1116163	-4.1687	2.30E-10	8.49E-09
i3_LQ_YHS_c16862/f1p0/3553	21.54645976	344.4380662	-3.9987	2.31E-10	8.53E-09
i1_HQ_YHS_c31573/f2p0/1477	175.4809989	2304.74008	-3.7152	2.32E-10	8.55E-09
i1_LQ_YHS_c9197/f1p2/1886	60.95690054	0	Inf	2.39E-10	8.78E-09
i1_LQ_YHS_c12054/f1p0/1904	210.3522792	4162.007729	-4.3064	2.39E-10	8.80E-09
i0_LQ_YHS_c3038/f1p3/347	4.608820707	115.5813065	-4.6484	2.40E-10	8.82E-09
i2_LQ_YHS_c20080/f1p0/2139	38.79457805	386.0763491	-3.315	2.42E-10	8.89E-09
i1_LQ_YHS_c36835/f1p2/1994	137.3641745	1451.346141	-3.4013	2.48E-10	9.12E-09
i1_LQ_YHS_c9039/f1p2/1543	19.64593952	338.2434118	-4.1058	2.54E-10	9.32E-09
i2_LQ_YHS_c6195/f1p1/2589	126.4129232	1236.03576	-3.2895	2.54E-10	9.32E-09
i2_LQ_YHS_c33743/f1p5/2092	130.5482043	1243.894915	-3.2522	2.57E-10	9.39E-09
i1_LQ_YHS_c33785/f1p0/1715	29.32618825	428.0371463	-3.8675	2.63E-10	9.62E-09
i1_HQ_YHS_c11701/f2p0/1716	142.8736311	1508.511815	-3.4003	2.64E-10	9.66E-09
i3_LQ_YHS_c5529/f1p8/3151	48.75671692	471.8919072	-3.2748	2.64E-10	9.66E-09
i2_HQ_YHS_c25324/f2p4/2618	146.6620949	1532.735309	-3.3855	2.65E-10	9.69E-09
i1_LQ_YHS_c14148/f1p0/1321	161.0173552	1721.762918	-3.4186	2.68E-10	9.77E-09
i4_HQ_YHS_c2274/f2p1/4442	139.8060277	1464.363317	-3.3888	2.68E-10	9.77E-09
i3_LQ_YHS_c14578/f1p4/3042	44.79671374	436.9828405	-3.2861	2.72E-10	9.90E-09
i4_LQ_YHS_c13288/f1p9/4095	19.07232078	233.8296113	-3.6159	2.72E-10	9.90E-09
i1_LQ_YHS_c35894/f1p3/1890	106.9973706	978.2117908	-3.1926	2.73E-10	9.92E-09
i2_LQ_YHS_c10411/f1p2/2225	61.39445394	539.54462	-3.1356	2.75E-10	1.00E-08
i0_LQ_YHS_c1730/f1p3/854	156.8514661	1584.576239	-3.3366	2.77E-10	1.00E-08
i2_LQ_YHS_c44524/f1p2/2324	43.91465145	438.0858495	-3.3184	2.77E-10	1.01E-08
i1_LQ_YHS_c3445/f1p1/1544	8.802205092	157.6493712	-4.1627	2.80E-10	1.01E-08

i1_LQ_YHS_c8592/f1p0/1395	31.89255453	321.5889407	-3.3339	2.83E-10	1.03E-08
i4_LQ_YHS_c3840/f1p0/4469	42.43800579	529.7790458	-3.642	2.85E-10	1.03E-08
i3_LQ_YHS_c8305/f1p3/3880	117.9468424	1087.364111	-3.2046	2.87E-10	1.04E-08
i2_LQ_YHS_c40568/f1p2/2141	90.49474902	804.0024171	-3.1513	2.91E-10	1.05E-08
i2_LQ_YHS_c7181/f1p13/2533	186.0752499	12.23979391	3.9262	2.96E-10	1.07E-08
i1_LQ_YHS_c8095/f1p0/1411	1126.19465	120.3777006	3.2258	2.96E-10	1.07E-08
i4_LQ_YHS_c3652/f1p12/5070	28.78598268	308.3073142	-3.4209	3.01E-10	1.09E-08
i2_LQ_YHS_c33923/f1p2/2248	13.11355641	271.1693728	-4.3701	3.03E-10	1.09E-08
i3_LQ_YHS_c4712/f1p0/3285	186.1835388	7226.360864	-5.2785	3.04E-10	1.09E-08
i1_LQ_YHS_c16825/f3p0/1518	106.1853072	935.811413	-3.1396	3.07E-10	1.10E-08
i1_LQ_YHS_c24375/f1p0/1363	169.3702776	1795.511548	-3.4061	3.09E-10	1.11E-08
i1_LQ_YHS_c3365/f1p0/1879	26.11376772	280.037389	-3.4227	3.12E-10	1.12E-08
i0_LQ_YHS_c3034/f1p0/986	0	148.9396882	-Inf	3.15E-10	1.13E-08
i1_HQ_YHS_c15159/f4p0/1489	152.0977935	1691.715996	-3.4754	3.22E-10	1.15E-08
i1_LQ_YHS_c10630/f1p2/1652	16.30900586	213.0518201	-3.7075	3.22E-10	1.16E-08
i2_LQ_YHS_c4445/f1p8/2179	32.97429524	336.2001868	-3.3499	3.23E-10	1.16E-08
i2_LQ_YHS_c3230/f1p0/2261	232.6929445	18.83090061	3.6273	3.26E-10	1.17E-08
i3_LQ_YHS_c14302/f1p2/3037	8.170238957	153.8077226	-4.2346	3.27E-10	1.17E-08
i2_LQ_YHS_c33511/f1p1/2892	3.802287496	107.6565037	-4.8234	3.28E-10	1.17E-08
i2_LQ_YHS_c13448/f1p6/2852	71.48713634	617.5664111	-3.1108	3.29E-10	1.18E-08
i2_LQ_YHS_c2526/f7p2/2293	188.2662589	2298.727669	-3.61	3.35E-10	1.20E-08
i2_HQ_YHS_c23845/f3p7/2740	194.5207299	2315.151259	-3.5731	3.37E-10	1.20E-08
i3_LQ_YHS_c13020/f1p4/3215	47.97868661	429.1477724	-3.161	3.37E-10	1.20E-08
i2_HQ_YHS_c18383/f2p0/2396	338.519321	33.58219882	3.3335	3.41E-10	1.21E-08
i3_LQ_YHS_c8268/f1p0/3525	209.5280808	5498.516506	-4.7138	3.42E-10	1.22E-08
i1_LQ_YHS_c22918/f1p0/1801	155.96446	4.314871345	5.1758	3.44E-10	1.22E-08
i2_LQ_YHS_c21394/f1p12/2149	3.540436881	115.3466315	-5.0259	3.44E-10	1.22E-08
i2_LQ_YHS_c25147/f1p0/2790	78.24130658	703.8849324	-3.1693	3.47E-10	1.23E-08
i4_LQ_YHS_c8061/f1p0/4902	13.86129224	173.6842854	-3.6473	3.50E-10	1.24E-08
i1_LQ_YHS_c9783/f1p1/1626	97.02184971	2.572248344	5.2372	3.53E-10	1.25E-08
i4_LQ_YHS_c13940/f1p0/4198	18.84448651	232.1594632	-3.6229	3.55E-10	1.26E-08
i1_LQ_YHS_c5503/f1p0/1745	17.0156873	305.5428439	-4.1664	3.60E-10	1.27E-08
i2_LQ_YHS_c38687/f1p7/3029	152.7079406	1607.175283	-3.3957	3.61E-10	1.28E-08
i2_HQ_YHS_c51757/f2p4/2459	60.85597886	533.5436796	-3.1321	3.72E-10	1.31E-08
i3_LQ_YHS_c10758/f1p3/3606	6.21853744	134.4139459	-4.434	3.72E-10	1.31E-08
i4_LQ_YHS_c6211/f1p2/4353	169.941437	8053.774476	-5.5666	3.74E-10	1.32E-08
i1_LQ_YHS_c8843/f1p225/1455	50.66566704	454.5343932	-3.1653	3.76E-10	1.33E-08
i2_LQ_YHS_c49225/f1p5/1938	218.7886687	3809.441045	-4.122	3.82E-10	1.35E-08
i5_LQ_YHS_c4108/f1p0/5206	0	53.89031798	-Inf	3.87E-10	1.36E-08
i1_LQ_YHS_c14142/f1p1/1122	12.52769973	1104.284739	-6.4618	3.91E-10	1.38E-08
i1_LQ_YHS_c38802/f1p0/1077	119.9597743	1061.738035	-3.1458	3.91E-10	1.38E-08
i2_LQ_YHS_c50353/f1p6/2736	23.52086478	416.8243704	-4.1474	3.94E-10	1.38E-08
i1_HQ_YHS_c13415/f2p2/1377	146.2698958	1378.391807	-3.2363	3.95E-10	1.39E-08
i1_HQ_YHS_c2636/f7p21/1895	148.4716433	1494.293585	-3.3312	3.96E-10	1.39E-08
i2_LQ_YHS_c64810/f1p25/2051	167.0371226	1810.480306	-3.4381	3.96E-10	1.39E-08
i3_LQ_YHS_c16571/f2p0/3318	43.13661276	404.2802274	-3.2284	3.99E-10	1.40E-08
i2_LQ_YHS_c13116/f1p16/2524	187.3217181	2502.81027	-3.74	4.01E-10	1.40E-08
i2_LQ_YHS_c17829/f1p1/2156	48.2397067	453.3503051	-3.2323	4.06E-10	1.42E-08
i1_HQ_YHS_c3405/f3p0/1790	78.07830847	690.6125892	-3.1449	4.07E-10	1.42E-08
i1_LQ_YHS_c9606/f1p7/1827	28.6874198	286.7046805	-3.3211	4.14E-10	1.45E-08
i3_LQ_YHS_c17454/f1p0/3433	83.6138756	746.7261882	-3.1588	4.34E-10	1.52E-08
i2_HQ_YHS_c13045/f2p1/2747	138.1230042	1336.433147	-3.2744	4.46E-10	1.56E-08
i1_LQ_YHS_c14424/f1p0/1059	145.4835637	1331.107003	-3.1937	4.47E-10	1.56E-08
i3_HQ_YHS_c13857/f3p0/3211	19.39804897	242.5871231	-3.6445	4.49E-10	1.56E-08
i3_LQ_YHS_c4043/f1p4/3344	12.45472331	182.6788384	-3.8745	4.53E-10	1.58E-08
i5_LQ_YHS_c3183/f1p0/5123	1.90638909	86.86489074	-5.5099	4.56E-10	1.59E-08
i3_LQ_YHS_c4560/f1p4/3131	30.85135959	440.6154409	-3.8361	4.69E-10	1.63E-08
i5_LQ_YHS_c1497/f1p3/5140	0	67.13671345	-Inf	4.70E-10	1.63E-08
i1_LQ_YHS_c14590/f1p2/1385	693.0277407	0	Inf	4.75E-10	1.65E-08
i4_LQ_YHS_c6659/f1p0/4849	180.447842	2130.426793	-3.5615	4.84E-10	1.68E-08
i0_LQ_YHS_c898/f1p0/899	158.2751138	1441.364773	-3.1869	4.88E-10	1.69E-08
i3_HQ_YHS_c9014/f2p0/3711	112.8484972	1043.925276	-3.2096	4.94E-10	1.71E-08
i2_LQ_YHS_c55868/f1p3/1999	213.7035774	5920.217239	-4.792	4.97E-10	1.72E-08
i2_LQ_YHS_c39870/f1p45/2147	24584.73095	7.199878768	11.738	4.99E-10	1.72E-08
i1_LQ_YHS_c22627/f1p0/1366	74.70723878	624.5360806	-3.0635	5.00E-10	1.73E-08
i2_LQ_YHS_c36416/f1p1/2634	144.2125934	7.761255536	4.2158	5.02E-10	1.73E-08
i1_LQ_YHS_c3778/f1p0/1697	146.0379388	1464.05988	-3.3256	5.04E-10	1.74E-08
i2_HQ_YHS_c11164/f2p15/2828	147.1234708	9720.432291	-6.0459	5.08E-10	1.75E-08
i1_LQ_YHS_c12616/f1p0/1833	8.225245036	145.4859621	-4.1447	5.11E-10	1.76E-08
i3_HQ_YHS_c2684/f3p0/3884	232.2410804	3416.84762	-3.879	5.20E-10	1.79E-08
i2_HQ_YHS_c12049/f2p1/2656	80.98919543	685.7074379	-3.0818	5.24E-10	1.80E-08
i2_LQ_YHS_c51605/f1p1/2824	10.9255991	170.8471858	-3.9669	5.37E-10	1.85E-08
i3_LQ_YHS_c13220/f1p0/3413	0.544682597	65.15198726	-6.9022	5.41E-10	1.86E-08
i1_LQ_YHS_c32043/f1p0/1855	3.81277818	107.6504622	-4.8194	5.46E-10	1.88E-08
i3_LQ_YHS_c13507/f1p5/3129	37.16147211	354.0231075	-3.252	5.51E-10	1.89E-08
i1_HQ_YHS_c29345/f3p0/1470	211.0648714	2797.77619	-3.7285	5.58E-10	1.91E-08
i2_LQ_YHS_c14506/f1p3/2074	978.6209624	115.3646362	3.0845	5.61E-10	1.92E-08
i2_LQ_YHS_c37961/f1p1/2658	17.17769452	212.4285137	-3.6284	5.64E-10	1.93E-08
i2_LQ_YHS_c27088/f1p44/2366	216.3926701	15.76508727	3.7788	5.66E-10	1.94E-08
i3_LQ_YHS_c4952/f1p3/3347	0	66.43208614	-Inf	5.67E-10	1.94E-08
i2_LQ_YHS_c23107/f1p1/2099	13.61292658	187.1432018	-3.7811	5.72E-10	1.95E-08
i1_LQ_YHS_c14413/f1p1/1199	122.1171762	1064.083666	-3.1233	5.75E-10	1.96E-08
i2_LQ_YHS_c33137/f1p0/2120	54.45792222	477.8285123	-3.1333	5.80E-10	1.98E-08
i0_LQ_YHS_c1199/f1p0/580	202.0688639	7000.163607	-5.1145	5.85E-10	2.00E-08
i1_LQ_YHS_c22875/f1p0/1641	74.31899135	2606.144659	-5.132	5.87E-10	2.00E-08
i1_HQ_YHS_c8660/f4p0/1862	0	141.766035	-Inf	5.87E-10	2.00E-08
i1_LQ_YHS_c20926/f1p0/1575	52.55440771	465.7090149	-3.1475	5.88E-10	2.00E-08
i1_HQ_YHS_c14848/f36p3/1979	151.0273405	1526.213576	-3.3371	5.90E-10	2.01E-08
i1_HQ_YHS_c2455/f10p0/1768	114.0027476	11443.78819	-6.6494	5.94E-10	2.01E-08

i1_LQ_YHS_c9860/f1p0/1845	49.99125469	445.799045	-3.1566	5.92E-10	2.01E-08
i2_HQ_YHS_c61568/f11p1/2017	174.8886295	1909.125429	-3.4484	5.94E-10	2.01E-08
i2_LQ_YHS_c63143/f1p8/2893	45.01024091	421.3857637	-3.2268	5.94E-10	2.01E-08
i3_LQ_YHS_c5996/f1p3/3101	57.14573317	484.2588296	-3.0831	5.93E-10	2.01E-08
i0_LQ_YHS_c3012/f1p3/835	2388.967738	202.7890546	3.5583	5.96E-10	2.02E-08
i2_HQ_YHS_c2757/f3p0/2263	336.8930915	34.702228	3.2792	5.96E-10	2.02E-08
i2_LQ_YHS_c38847/f1p4/2348	3.052829538	148.4321866	-5.6035	6.08E-10	2.06E-08
i6_LQ_YHS_c226/f1p0/6798	13.28304329	184.2640765	-3.7941	6.18E-10	2.09E-08
i1_LQ_YHS_c34387/f1p0/1646	58.02651496	0	Inf	6.26E-10	2.11E-08
i2_LQ_YHS_c33170/f1p4/2313	44.23726568	1927.457245	-5.4453	6.29E-10	2.12E-08
i2_LQ_YHS_c10560/f1p0/2195	32.44807485	302.2688184	-3.2196	6.30E-10	2.13E-08
i3_LQ_YHS_c9127/f1p0/3118	226.3241076	4211.572244	-4.2179	6.46E-10	2.18E-08
i1_LQ_YHS_c35761/f1p0/1790	60.20094544	521.7217784	-3.1154	6.57E-10	2.21E-08
i2_HQ_YHS_c46028/f17p3/2120	2039.146631	186.3245145	3.4521	6.59E-10	2.22E-08
i2_LQ_YHS_c23574/f1p1/2219	37.27006731	356.7705933	-3.2589	6.65E-10	2.24E-08
i2_LQ_YHS_c15091/f1p22/2097	175.0498565	9107.747803	-5.7013	6.67E-10	2.24E-08
i3_LQ_YHS_c3682/f1p5/3163	2.178730389	219.8135022	-6.6566	6.70E-10	2.25E-08
i1_LQ_YHS_c13925/f1p0/1118	8563.339475	0	Inf	6.77E-10	2.27E-08
i4_LQ_YHS_c10141/f1p3/4706	33.16155862	324.3525809	-3.29	6.77E-10	2.27E-08
i1_LQ_YHS_c18980/f1p1/1356	123.6234689	1021.598553	-3.0468	6.81E-10	2.28E-08
i1_LQ_YHS_c24560/f1p0/1412	79.08742818	644.2070705	-3.026	6.95E-10	2.33E-08
i2_LQ_YHS_c25319/f1p1/2192	15.33632708	200.0293627	-3.7052	7.05E-10	2.36E-08
i1_LQ_YHS_c12674/f1p1/1668	184.6825872	1968.630355	-3.4141	7.11E-10	2.38E-08
i1_HQ_YHS_c17954/f2p0/1464	100.5232152	1019.705752	-3.3426	7.13E-10	2.38E-08
i2_LQ_YHS_c20804/f1p7/2100	52.24264796	485.0752738	-3.2149	7.21E-10	2.40E-08
i3_LQ_YHS_c19374/f1p0/3590	228.4690778	5024.781891	-4.459	7.20E-10	2.40E-08
i2_LQ_YHS_c10894/f1p1/2730	0	51.45724984	-Inf	7.32E-10	2.44E-08
i1_LQ_YHS_c39104/f1p5/1203	103.0563119	914.0615382	-3.1489	7.34E-10	2.45E-08
i1_LQ_YHS_c12574/f1p15/1650	87.62382142	749.3812507	-3.0963	7.56E-10	2.52E-08
i1_LQ_YHS_c8473/f1p0/1834	143.2264297	1429.133324	-3.3188	7.78E-10	2.59E-08
i0_HQ_YHS_c3735/f22p0/883	14.82185277	21967.34408	-10.533	7.82E-10	2.60E-08
i1_HQ_YHS_c14975/f27p0/1902	173.6043609	1686.678618	-3.2803	7.86E-10	2.61E-08
i2_LQ_YHS_c52285/f1p0/2965	30.37678722	331.1780094	-3.4466	7.88E-10	2.62E-08
i2_LQ_YHS_c64645/f1p1/2017	156.989913	1590.648348	-3.3409	8.15E-10	2.70E-08
i0_LQ_YHS_c1104/f1p0/804	637.1382291	65.41190346	3.284	8.17E-10	2.71E-08
i2_LQ_YHS_c14501/f1p0/2048	0	58.62687333	-Inf	8.21E-10	2.72E-08
i1_LQ_YHS_c28967/f1p0/1256	159.3112884	9710.193948	-5.9296	8.24E-10	2.73E-08
i2_LQ_YHS_c40060/f1p0/2529	3.596248367	381.8576433	-6.7304	8.29E-10	2.75E-08
i2_HQ_YHS_c2040/f2p3/2630	159.0940489	1504.733719	-3.2416	8.33E-10	2.76E-08
i1_LQ_YHS_c15061/f12p0/1923	90.31143851	731.7817571	-3.0184	8.46E-10	2.79E-08
i1_LQ_YHS_c37042/f1p2/1676	49.36564926	424.74996	-3.105	8.46E-10	2.79E-08
i2_LQ_YHS_c59475/f1p2/2052	118.5883741	998.8732263	-3.0743	8.48E-10	2.80E-08
i2_LQ_YHS_c64839/f1p2/2026	146.4153652	1334.235022	-3.1879	8.54E-10	2.82E-08
i2_LQ_YHS_c35364/f1p1/2108	9.094272366	139.8812333	-3.9431	8.59E-10	2.83E-08
i2_LQ_YHS_c6644/f1p4/2469	9.76947338	150.5475595	-3.9458	8.68E-10	2.86E-08
i1_HQ_YHS_c23668/f2p5/1711	0	455.3133765	-Inf	8.76E-10	2.88E-08
i2_LQ_YHS_c14368/f1p29/2040	86.52999595	0	Inf	9.03E-10	2.97E-08
i1_LQ_YHS_c36667/f1p5/1935	2540.286418	0	Inf	9.30E-10	3.06E-08
i1_LQ_YHS_c28377/f1p2/1206	142.7213761	1227.776552	-3.1048	9.33E-10	3.07E-08
i4_LQ_YHS_c13216/f1p3/4032	237.0373332	3431.932899	-3.8558	9.38E-10	3.08E-08
i2_LQ_YHS_c36046/f1p2/2314	44.4480547	403.6587796	-3.1829	9.60E-10	3.15E-08
i2_LQ_YHS_c40766/f1p3/2962	111.118414	974.781833	-3.133	9.60E-10	3.15E-08
i2_LQ_YHS_c55222/f1p2/2746	8.720807154	143.9796738	-4.0453	9.59E-10	3.15E-08
i1_LQ_YHS_c22286/f1p0/1883	1492.829467	157.6722808	3.243	9.83E-10	3.22E-08
i0_LQ_YHS_c3207/f1p0/981	19.74037243	795.2531559	-5.3322	9.88E-10	3.23E-08
i2_LQ_YHS_c19200/f1p0/2480	38.93432339	359.5744797	-3.2072	9.90E-10	3.24E-08
i1_HQ_YHS_c40224/f2p0/1836	232.2928624	4240.059952	-4.1901	9.99E-10	3.26E-08
i1_LQ_YHS_c36971/f1p2/1368	0.272341299	96.59926138	-8.4705	1.00E-09	3.26E-08
i2_LQ_YHS_c38678/f1p68/2481	0	51.00148086	-Inf	9.99E-10	3.26E-08
i2_LQ_YHS_c10013/f1p0/2461	0	91.42356339	-Inf	1.01E-09	3.28E-08
i1_LQ_YHS_c24256/f1p0/1902	73.34979032	608.4678742	-3.0523	1.01E-09	3.29E-08
i2_LQ_YHS_c38172/f1p6/2727	46.60613402	801.4650921	-4.104	1.03E-09	3.36E-08
i2_LQ_YHS_c19612/f1p0/2483	6.197556072	130.420166	-4.3953	1.04E-09	3.40E-08
i1_HQ_YHS_c12132/f2p0/1896	79.14910851	640.3395902	-3.0162	1.07E-09	3.47E-08
i4_LQ_YHS_c3062/f1p0/5057	12.50257164	202.2636537	-4.0159	1.08E-09	3.51E-08
i1_LQ_YHS_c19557/f1p2/1976	227.581556	3327.700519	-3.8701	1.08E-09	3.52E-08
i2_LQ_YHS_c53678/f1p4/2804	104.8372963	842.5067996	-3.0065	1.10E-09	3.58E-08
i3_HQ_YHS_c21157/f3p3/3098	19.65181675	228.0629532	-3.5367	1.12E-09	3.64E-08
i0_LQ_YHS_c979/f1p0/693	66.73141256	542.4282754	-3.023	1.13E-09	3.66E-08
i1_LQ_YHS_c28793/f1p0/1337	23.1666588	250.5433596	-3.4349	1.15E-09	3.72E-08
i4_HQ_YHS_c14623/f4p0/4166	133.4754005	1199.24193	-3.1675	1.16E-09	3.74E-08
i1_HQ_YHS_c37718/f3p1/1300	79.02941945	6597.827181	-6.3835	1.17E-09	3.77E-08
i2_HQ_YHS_c29653/f16p3/2543	53.65123582	479.1025648	-3.1587	1.17E-09	3.78E-08
i3_LQ_YHS_c3328/f1p0/3579	12.5071851	210.7692702	-4.0748	1.18E-09	3.82E-08
i0_LQ_YHS_c1310/f1p0/533	395.4202895	30.19877047	3.7108	1.20E-09	3.89E-08
i1_LQ_YHS_c12338/f1p0/1863	183.7475473	13.89671303	3.7249	1.21E-09	3.91E-08
i2_LQ_YHS_c6314/f1p1/2212	9.651981557	148.2412282	-3.941	1.22E-09	3.92E-08
i4_LQ_YHS_c9860/f1p0/4382	1.089365194	71.64119723	-6.0392	1.22E-09	3.93E-08
i1_LQ_YHS_c27376/f2p3/1110	119.566108	1006.121845	-3.0729	1.22E-09	3.94E-08
i3_LQ_YHS_c17499/f1p0/3561	11.62974464	168.9547582	-3.8607	1.23E-09	3.95E-08
i3_LQ_YHS_c3873/f1p1/3456	54.63042849	453.424077	-3.0531	1.23E-09	3.95E-08
i2_LQ_YHS_c37756/f1p5/2075	172.6623895	1690.870056	-3.2917	1.24E-09	3.99E-08
i1_HQ_YHS_c39980/f3p1/1306	246.8760695	4190.731234	-4.0853	1.25E-09	4.02E-08
i2_LQ_YHS_c8460/f1p7/2789	10.92179105	163.1280302	-3.9007	1.29E-09	4.15E-08
i3_LQ_YHS_c17603/f1p0/3304	5.589920995	111.4883949	-4.3179	1.31E-09	4.19E-08
i2_LQ_YHS_c59421/f1p0/2086	3.66969153	100.0794921	-4.7693	1.35E-09	4.33E-08
i1_LQ_YHS_c43892/f1p4/1074	251.59285	4020.346302	-3.9982	1.36E-09	4.34E-08
i1_LQ_YHS_c32389/f1p6/1469	35.46605753	1628.377197	-5.5209	1.37E-09	4.37E-08
i3_HQ_YHS_c13854/f2p0/3038	55.80371079	481.0608714	-3.1078	1.38E-09	4.40E-08

i3_LQ_YHS_c14950/f1p1/3005	7.706157265	136.9450404	-4.1514	1.38E-09	4.40E-08
i2_LQ_YHS_c13299/f1p6/2794	112.8916997	1.769361165	5.9956	1.39E-09	4.43E-08
i3_LQ_YHS_c7826/f1p0/3362	92.95112826	712.4817015	-2.9383	1.39E-09	4.44E-08
i3_LQ_YHS_c6585/f1p2/3273	2.178730389	84.75954945	-5.2818	1.40E-09	4.48E-08
i1_LQ_YHS_c8261/f1p5/1564	16.45590056	200.8461112	-3.6094	1.42E-09	4.51E-08
i1_LQ_YHS_c24828/f1p1/1854	853.5484136	105.8955167	3.0108	1.44E-09	4.59E-08
i2_LQ_YHS_c48797/f1p2/2946	0	49.56043932	-Inf	1.47E-09	4.68E-08
i4_LQ_YHS_c13848/f1p0/4100	6.881984002	127.4399764	-4.2108	1.47E-09	4.68E-08
i1_HQ_YHS_c16835/f4p0/1792	58.79187409	474.5645459	-3.0129	1.49E-09	4.74E-08
i2_LQ_YHS_c49932/f1p1/2745	6.63523753	128.3223259	-4.2735	1.49E-09	4.74E-08
i4_HQ_YHS_c12856/f3p4/4174	150.2133444	1364.5488	-3.1833	1.50E-09	4.75E-08
i3_LQ_YHS_c19591/f1p0/3667	50.80461534	422.1276733	-3.0546	1.50E-09	4.77E-08
i4_LQ_YHS_c9398/f1p21/4565	91.75865617	750.9841908	-3.0329	1.51E-09	4.79E-08
i3_LQ_YHS_c10772/f1p4/3315	15.7983396	195.4894875	-3.6292	1.53E-09	4.85E-08
i0_LQ_YHS_c2546/f1p0/647	109.9701066	2204.468409	-4.3252	1.56E-09	4.93E-08
i3_LQ_YHS_c18962/f1p1/3369	24.85905399	554.601007	-4.4796	1.56E-09	4.94E-08
i3_LQ_YHS_c18507/f1p0/3124	126.3973356	1099.20479	-3.1204	1.60E-09	5.08E-08
i5_LQ_YHS_c3650/f1p0/5125	190.605649	3234.860521	-4.085	1.62E-09	5.13E-08
i4_LQ_YHS_c12570/f1p0/4261	9.295698036	145.2676332	-3.966	1.62E-09	5.13E-08
i3_LQ_YHS_c5817/f1p0/3773	18.29265454	210.3805191	-3.5237	1.66E-09	5.23E-08
i1_HQ_YHS_c29292/f5p5/1497	207.7174973	2107.931558	-3.3431	1.67E-09	5.27E-08
i2_LQ_YHS_c27003/f1p1/2502	20.12178404	642.624548	-4.9971	1.68E-09	5.30E-08
i3_LQ_YHS_c4426/f1p4/3846	79.93496553	655.2419538	-3.0351	1.72E-09	5.40E-08
i2_HQ_YHS_c1610/f3p2/2315	18.65563744	195.2375527	-3.3875	1.74E-09	5.48E-08
i1_LQ_YHS_c33329/f1p32/1839	554.3141407	2.15996363	8.0036	1.75E-09	5.49E-08
i2_LQ_YHS_c26615/f1p0/2141	58.64878744	509.5880247	-3.1192	1.76E-09	5.52E-08
i1_LQ_YHS_c18894/f1p0/1507	132.4106811	1084.778232	-3.0343	1.77E-09	5.55E-08
i1_LQ_YHS_c21334/f1p0/1685	1.634047791	92.15753346	-5.8176	1.77E-09	5.57E-08
i2_LQ_YHS_c35690/f1p3/2406	36.06715478	410.2364573	-3.5077	1.80E-09	5.64E-08
i4_LQ_YHS_c4171/f1p1/4669	8.711580238	143.6808563	-4.0438	1.82E-09	5.70E-08
i2_LQ_YHS_c13810/f1p7/2721	9.223518641	145.1690536	-3.9763	1.83E-09	5.73E-08
i1_LQ_YHS_c12599/f1p43/1894	10.13243953	612.2731304	-5.9171	1.83E-09	5.74E-08
i2_HQ_YHS_c60133/f11p1/2419	89.97393075	695.7882944	-2.9511	1.83E-09	5.74E-08
i3_LQ_YHS_c6385/f1p0/3498	67.91632962	546.4516018	-3.0083	1.84E-09	5.76E-08
i2_LQ_YHS_c18100/f1p0/2793	4.696104245	108.5661447	-4.531	1.84E-09	5.76E-08
i2_LQ_YHS_c51519/f1p0/2384	27.12300712	259.0524872	-3.2557	1.87E-09	5.85E-08
i2_LQ_YHS_c54173/f1p2/2204	0.817023896	138.49157	-7.4052	1.88E-09	5.88E-08
i2_LQ_YHS_c51225/f1p3/2331	93.05986828	686.1168461	-2.8822	1.90E-09	5.94E-08
i1_LQ_YHS_c34654/f1p0/1996	161.0179823	1492.14947	-3.2121	1.91E-09	5.95E-08
i1_HQ_YHS_c17079/f2p0/1715	72.65868814	576.1263125	-2.9872	1.91E-09	5.95E-08
i2_LQ_YHS_c59086/f1p1/2090	312.4804792	34.08516311	3.1966	1.97E-09	6.13E-08
i1_HQ_YHS_c1969/f4p0/1829	51.43229832	419.7999783	-3.029	1.98E-09	6.16E-08
i2_LQ_YHS_c39083/f1p1/2291	28.53510623	268.8539977	-3.236	2.00E-09	6.23E-08
i0_LQ_YHS_c2955/f1p1/840	130.1141609	1074.802206	-3.0462	2.04E-09	6.35E-08
i1_LQ_YHS_c28009/f1p8/1346	64.56508047	2951.151683	-5.5144	2.06E-09	6.41E-08
i1_HQ_YHS_c2688/f6p3/1568	217.7005745	2691.937359	-3.6282	2.08E-09	6.46E-08
i1_LQ_YHS_c17613/f1p11/1736	28.16993447	262.0215794	-3.2175	2.12E-09	6.57E-08
i0_LQ_YHS_c1365/f1p0/941	16.23730158	187.1051651	-3.5265	2.19E-09	6.80E-08
i2_LQ_YHS_c22950/f1p1/2101	53.10511234	431.3333666	-3.0219	2.21E-09	6.83E-08
i2_LQ_YHS_c7423/f1p0/2493	108.2430429	1100.53036	-3.3459	2.21E-09	6.84E-08
i1_HQ_YHS_c22313/f2p2/1370	157.5346458	1349.301369	-3.0985	2.23E-09	6.88E-08
i0_LQ_YHS_c2667/f1p6/993	215.8996355	2186.874286	-3.3404	2.24E-09	6.92E-08
i2_LQ_YHS_c26773/f1p2/2475	50.94754881	724.086373	-3.8291	2.25E-09	6.96E-08
i2_HQ_YHS_c60716/f21p14/2481	126.9867358	2631.68365	-4.3732	2.26E-09	6.98E-08
i1_LQ_YHS_c20058/f1p0/1936	1.623557107	127.6213751	-6.2966	2.27E-09	7.01E-08
i2_HQ_YHS_c19506/f2p6/2214	198.2877193	2038.785622	-3.362	2.31E-09	7.12E-08
i2_LQ_YHS_c54228/f1p1/2237	126.1341099	1024.915262	-3.0225	2.35E-09	7.25E-08
i2_LQ_YHS_c35567/f1p0/2461	378.6170643	45.03075618	3.0718	2.39E-09	7.35E-08
i4_LQ_YHS_c13559/f1p0/4127	24.09195598	245.2879608	-3.3479	2.39E-09	7.36E-08
i1_LQ_YHS_c5471/f1p3/1737	257.8740503	4045.022712	-3.9714	2.43E-09	7.47E-08
i1_LQ_YHS_c32226/f1p0/1974	161.7690272	1359.048456	-3.0706	2.45E-09	7.54E-08
i2_HQ_YHS_c60082/f5p6/2685	254.524023	4181.38955	-4.0381	2.47E-09	7.59E-08
i2_LQ_YHS_c56288/f1p1/2433	70.74165517	560.807523	-2.9869	2.51E-09	7.72E-08
i2_HQ_YHS_c19163/f2p1/2318	29.70413049	276.22852	-3.2171	2.52E-09	7.74E-08
i2_LQ_YHS_c33781/f1p12/2652	127.2980216	999.6176174	-2.9732	2.54E-09	7.80E-08
i0_LQ_YHS_c3063/f1p0/628	29.34082566	1509.334863	-5.6849	2.61E-09	8.00E-08
i3_LQ_YHS_c2845/f1p6/3253	14.23854038	278.1745388	-4.2881	2.68E-09	8.20E-08
i2_LQ_YHS_c58711/f1p3/2078	1128.462339	138.862902	3.0226	2.69E-09	8.24E-08
i3_LQ_YHS_c3643/f1p2/3328	3.858557342	101.7487349	-4.7208	2.71E-09	8.29E-08
i2_HQ_YHS_c43178/f2p7/1806	3.23662353	305.5436405	-6.5607	2.75E-09	8.41E-08
i2_LQ_YHS_c59747/f1p8/2060	111.5120971	860.7815756	-2.9484	2.76E-09	8.42E-08
i3_LQ_YHS_c6545/f1p3/3193	56.21740823	2043.045291	-5.1836	2.78E-09	8.48E-08
i1_HQ_YHS_c29644/f14p0/1850	145.6454621	1586.476923	-3.4453	2.79E-09	8.52E-08
i2_HQ_YHS_c60228/f4p0/2995	12.90671187	383.3772815	-4.8926	2.81E-09	8.56E-08
i1_LQ_YHS_c11316/f1p0/1594	168.8066661	1421.615319	-3.0741	2.83E-09	8.63E-08
i2_LQ_YHS_c51206/f1p0/2529	3.858557342	174.6626496	-5.5004	2.86E-09	8.72E-08
i2_LQ_YHS_c29386/f1p1/2014	16.8608462	349.0376125	-4.3716	2.88E-09	8.77E-08
i1_HQ_YHS_c2824/f8p1/1978	0	131.8114028	-Inf	2.89E-09	8.79E-08
i2_LQ_YHS_c19338/f1p3/2278	57.06543744	467.3587465	-3.0338	2.96E-09	9.00E-08
i1_LQ_YHS_c1409/f6p0/1495	233.7029259	2822.697744	-3.5943	2.97E-09	9.01E-08
i3_LQ_YHS_c5263/f1p0/3209	29.47631296	273.1307928	-3.212	2.99E-09	9.08E-08
i1_LQ_YHS_c10884/f1p2/1892	64.91308729	510.2032393	-2.9745	3.11E-09	9.43E-08
i1_LQ_YHS_c7011/f1p51/1673	5.446825971	114.3093412	-4.3914	3.14E-09	9.51E-08
i2_LQ_YHS_c54278/f1p1/2108	3.540436881	96.60964737	-4.7702	3.15E-09	9.55E-08
i1_LQ_YHS_c33958/f1p0/1513	248.2528227	7335.837288	-4.8851	3.19E-09	9.66E-08
i4_LQ_YHS_c6772/f1p4/5065	4.629802076	105.7682267	-4.5138	3.22E-09	9.73E-08
i1_LQ_YHS_c33672/f1p0/1880	18.57088144	278.537462	-3.9068	3.24E-09	9.81E-08
i1_LQ_YHS_c5230/f1p0/2021	242.08775	13.35476717	4.1801	3.25E-09	9.83E-08
i3_LQ_YHS_c4234/f1p0/3906	26.67690415	244.8464915	-3.1982	3.26E-09	9.86E-08

i1_LQ_YHS_c6721/f1p0/1585	116.1499975	944.6965391	-3.0239	3.31E-09	9.98E-08
i1_LQ_YHS_c37055/f1p1/1910	92.82866756	695.4995107	-2.9054	3.32E-09	1.00E-07
i2_LQ_YHS_c25051/f1p8/2191	4.039807051	104.3758786	-4.6914	3.35E-09	1.01E-07
i2_LQ_YHS_c21663/f1p10/2268	267.8765088	5956.737854	-4.4749	3.38E-09	1.02E-07
i0_LQ_YHS_c3122/f1p23/1008	88.00458918	1254.146657	-3.833	3.44E-09	1.03E-07
i2_LQ_YHS_c11476/f1p11/2534	63.04104484	2001.996414	-4.989	3.44E-09	1.03E-07
i2_LQ_YHS_c9121/f1p5/2120	53.02864141	628.75485	-3.5677	3.44E-09	1.03E-07
i3_LQ_YHS_c6776/f1p1/3381	7.38850354	128.0304228	-4.1151	3.47E-09	1.04E-07
i1_LQ_YHS_c14345/f1p0/1190	144.7908005	1168.36441	-3.0124	3.50E-09	1.05E-07
i2_LQ_YHS_c50220/f1p1/2351	18.73909618	236.3226368	-3.6566	3.52E-09	1.06E-07
i3_LQ_YHS_c3154/f1p0/3572	81.80079209	627.2452171	-2.9388	3.52E-09	1.06E-07
i2_HQ_YHS_c2333/f7p4/2235	159.7434027	1403.533643	-3.1352	3.57E-09	1.07E-07
i2_LQ_YHS_c9278/f1p6/1927	14.09211241	176.0560574	-3.6431	3.66E-09	1.10E-07
i1_HQ_YHS_c28459/f3p0/1452	175.9337761	1624.212315	-3.2066	3.67E-09	1.10E-07
i1_LQ_YHS_c26337/f1p2/1510	146.318041	1202.952616	-3.0394	3.67E-09	1.10E-07
i2_LQ_YHS_c11295/f1p6/2212	15.49913133	621.7825187	-5.3262	3.74E-09	1.12E-07
i1_LQ_YHS_c34560/f1p0/1880	95.6892481	1074.156373	-3.4887	3.80E-09	1.14E-07
i2_LQ_YHS_c7042/f1p0/2427	526.4956382	57.06083376	3.2058	3.82E-09	1.14E-07
i1_LQ_YHS_c34882/f1p0/1962	124.1089819	932.6035046	-2.9097	3.84E-09	1.15E-07
i1_LQ_YHS_c33233/f1p0/1844	268.3199466	5400.887968	-4.3312	3.92E-09	1.17E-07
i1_LQ_YHS_c6684/f1p3/1950	255.9590662	3451.679341	-3.7533	3.93E-09	1.17E-07
i0_LQ_YHS_c3048/f1p11/949	227.1519609	8434.12494	-5.2145	3.94E-09	1.17E-07
i1_HQ_YHS_c13388/f2p5/1383	176.1495424	2323.383818	-3.7214	3.94E-09	1.17E-07
i2_LQ_YHS_c52382/f1p2/2600	35.62425669	313.4498013	-3.1373	3.96E-09	1.18E-07
i0_LQ_YHS_c3000/f1p0/872	17.8465432	3511.096018	-7.6201	3.99E-09	1.19E-07
i1_LQ_YHS_c28568/f1p0/1183	0.631966135	58.30147804	-6.5275	4.03E-09	1.20E-07
i1_HQ_YHS_c5358/f2p0/1494	2009.608997	0	Inf	4.04E-09	1.20E-07
i1_LQ_YHS_c4158/f1p0/1693	0	46.44063597	-Inf	4.05E-09	1.20E-07
i1_LQ_YHS_c8023/f1p21/1773	93.17034717	663.2652549	-2.8316	4.09E-09	1.21E-07
i3_LQ_YHS_c13172/f1p0/3355	0	46.68261939	-Inf	4.17E-09	1.24E-07
i1_LQ_YHS_c28961/f1p4/1173	254.3197156	3003.650492	-3.562	4.32E-09	1.28E-07
i1_LQ_YHS_c3561/f1p7/2055	32.2408749	290.2211685	-3.1702	4.33E-09	1.28E-07
i1_LQ_YHS_c35509/f1p0/1429	69.00108973	523.2394394	-2.9228	4.35E-09	1.29E-07
i2_HQ_YHS_c45851/f6p3/2760	92.77649419	724.4633959	-2.9651	4.37E-09	1.29E-07
i3_HQ_YHS_c21666/f66p0/3262	33.72976687	2673.669082	-6.3087	4.39E-09	1.30E-07
i1_HQ_YHS_c9173/f2p1/1483	71.63622828	2729.999185	-5.2521	4.40E-09	1.30E-07
i0_LQ_YHS_c2413/f1p6/721	139.8198693	1040.012527	-2.895	4.45E-09	1.31E-07
i1_LQ_YHS_c25315/f1p4/1851	164.2545987	1296.953259	-2.9811	4.48E-09	1.32E-07
i1_LQ_YHS_c6552/f1p0/1907	23324.63282	0	Inf	4.48E-09	1.32E-07
i1_LQ_YHS_c3868/f1p5/1627	0	140.9029966	-Inf	4.49E-09	1.32E-07
i3_LQ_YHS_c5236/f1p0/3085	22.5661731	227.512281	-3.3337	4.50E-09	1.32E-07
i2_LQ_YHS_c34433/f1p7/2381	33.44807896	286.7845369	-3.1	4.51E-09	1.33E-07
i2_LQ_YHS_c40891/f1p3/2247	52.50194591	410.0032842	-2.9652	4.60E-09	1.35E-07
i2_LQ_YHS_c20690/f3p11/2619	149.7020092	1222.170331	-3.0293	4.60E-09	1.35E-07
i1_LQ_YHS_c17873/f1p1/1446	0	46.38996421	-Inf	4.64E-09	1.36E-07
i1_LQ_YHS_c29069/f1p1/1089	248.4658581	2899.823025	-3.5448	4.65E-09	1.37E-07
i2_LQ_YHS_c35611/f1p3/2307	109.9755961	849.1898061	-2.9489	4.82E-09	1.41E-07
i5_LQ_YHS_c2708/f1p1/5887	0.544682597	57.34728701	-6.7182	4.83E-09	1.42E-07
i3_LQ_YHS_c12439/f1p0/3434	78.68917354	1029.412625	-3.7095	4.85E-09	1.42E-07
i1_LQ_YHS_c11026/f1p3/1601	0	45.53376001	-Inf	4.91E-09	1.44E-07
i2_LQ_YHS_c18541/f1p17/2242	270.1172904	5928.459869	-4.456	4.92E-09	1.44E-07
i3_LQ_YHS_c22508/f1p0/3040	24.33154471	238.8367457	-3.2951	4.94E-09	1.44E-07
i4_LQ_YHS_c4034/f1p1/4278	0.97648683	61.49197683	-5.9767	4.96E-09	1.45E-07
i1_LQ_YHS_c43977/f1p0/1035	238.7392593	8062.805889	-5.0778	4.97E-09	1.45E-07
i2_HQ_YHS_c61105/f7p4/2135	264.1535991	3311.478477	-3.648	4.98E-09	1.45E-07
i0_LQ_YHS_c3152/f1p0/838	22989.75501	22.94660092	9.9685	4.99E-09	1.46E-07
i2_LQ_YHS_c23739/f1p1/2081	488.623935	64.47804869	2.9218	5.00E-09	1.46E-07
i2_LQ_YHS_c3874/f1p5/2424	0	45.80520596	-Inf	5.04E-09	1.47E-07
i3_LQ_YHS_c19869/f1p0/3684	10.23736312	163.2220675	-3.9949	5.18E-09	1.51E-07
i2_HQ_YHS_c17766/f6p21/2598	273.3342789	4827.287003	-4.1425	5.19E-09	1.51E-07
i2_HQ_YHS_c8551/f2p8/2169	196.5337061	1822.56855	-3.2131	5.24E-09	1.52E-07
i1_LQ_YHS_c26129/f1p1/1760	93.3230856	684.4934527	-2.8747	5.27E-09	1.53E-07
i2_LQ_YHS_c49140/f1p2/2142	0.514474312	57.12580028	-6.7949	5.28E-09	1.53E-07
i0_LQ_YHS_c2364/f1p1/980	135.0032526	12904.34536	-6.5787	5.32E-09	1.55E-07
i1_LQ_YHS_c7381/f1p0/1387	2.178730389	79.06307528	-5.1814	5.39E-09	1.57E-07
i2_LQ_YHS_c50169/f1p14/2428	267.0150018	3602.659107	-3.7541	5.42E-09	1.57E-07
i2_LQ_YHS_c33034/f1p0/2410	36.73508674	304.5173301	-3.0513	5.50E-09	1.59E-07
i0_LQ_YHS_c644/f1p0/884	17805.53083	1.433854048	13.6	5.53E-09	1.60E-07
i2_LQ_YHS_c26981/f1p1/2178	123.1191382	895.582654	-2.8628	5.55E-09	1.61E-07
i2_LQ_YHS_c49829/f1p3/2473	17.94893925	191.348868	-3.4142	5.59E-09	1.62E-07
i1_LQ_YHS_c35986/f1p1/1724	0	117.1691492	-Inf	5.61E-09	1.62E-07
i3_LQ_YHS_c10602/f1p0/3485	5.174484673	106.8303955	-4.3678	5.65E-09	1.63E-07
i2_HQ_YHS_c60840/f13p1/2278	219.0283495	1914.060284	-3.1274	5.66E-09	1.64E-07
i2_LQ_YHS_c20069/f1p1/3050	55.73267547	432.6249629	-2.9565	5.67E-09	1.64E-07
i1_HQ_YHS_c16903/f5p0/1420	241.1287392	2408.047639	-3.32	5.71E-09	1.65E-07
i2_LQ_YHS_c41043/f1p0/2249	81.23894455	665.039083	-3.0332	5.73E-09	1.65E-07
i2_LQ_YHS_c38037/f1p3/2518	153.5826932	0.353872233	8.7616	5.74E-09	1.65E-07
i1_LQ_YHS_c19662/f1p18/1525	268.4848941	6808.125435	-4.6643	5.90E-09	1.70E-07
i2_LQ_YHS_c52152/f1p2/2550	807.1677635	108.3265324	2.8975	5.93E-09	1.71E-07
i3_HQ_YHS_c21221/f41p4/3719	259.4383373	6869.551417	-4.7268	5.95E-09	1.71E-07
i2_LQ_YHS_c20303/f1p10/2225	30.76963136	376.4415497	-3.6128	5.98E-09	1.72E-07
i1_LQ_YHS_c36878/f1p5/1851	69.37155229	541.7080491	-2.9651	6.02E-09	1.73E-07
i2_LQ_YHS_c36653/f1p0/2124	9.572652793	135.4487838	-3.8227	6.04E-09	1.73E-07
i2_LQ_YHS_c39283/f1p0/2899	94.55682633	672.7632991	-2.8308	6.04E-09	1.73E-07
i2_LQ_YHS_c18354/f1p0/2743	14.62504056	167.307751	-3.516	6.09E-09	1.75E-07
i2_LQ_YHS_c5012/f1p3/2852	4.584489648	103.229704	-4.493	6.10E-09	1.75E-07
i2_LQ_YHS_c54377/f1p3/2834	1.418781746	98.14216012	-6.1121	6.11E-09	1.75E-07
i2_LQ_YHS_c40916/f1p6/2360	10.61336424	144.4012381	-3.7661	6.12E-09	1.75E-07
i2_LQ_YHS_c41702/f1p3/2612	17.69505179	181.5806113	-3.3592	6.12E-09	1.75E-07

i3_LQ_YHS_c12692/f1p1/3441	30.88458727	273.0725803	-3.1443	6.15E-09	1.76E-07
i2_LQ_YHS_c18484/f1p0/2141	417.5499241	45.85864274	3.1867	6.19E-09	1.77E-07
i2_LQ_YHS_c39718/f1p7/2715	212.8093406	1940.313	-3.1887	6.20E-09	1.77E-07
i1_LQ_YHS_c31659/f2p0/1919	580.8225813	0	Inf	6.24E-09	1.78E-07
i2_LQ_YHS_c52236/f1p1/2339	509.9839188	32.12780853	3.9886	6.27E-09	1.79E-07
i3_LQ_YHS_c11829/f1p0/3125	51.02402811	387.6335426	-2.9254	6.29E-09	1.79E-07
i2_LQ_YHS_c14804/f1p2/2054	179.1036487	15.55477816	3.5254	6.31E-09	1.80E-07
i3_HQ_YHS_c2167/f2p1/3899	521.3309895	70.28424054	2.8909	6.35E-09	1.81E-07
i1_LQ_YHS_c8022/f1p0/1441	167.6053225	1353.414937	-3.0135	6.41E-09	1.82E-07
i1_LQ_YHS_c21734/f1p0/1847	85.64063947	633.3741563	-2.8867	6.47E-09	1.84E-07
i2_LQ_YHS_c40721/f1p7/2764	5.507709276	116.88021	-4.4074	6.59E-09	1.87E-07
i1_LQ_YHS_c3642/f1p5/1856	31527.05719	0.353872233	16.443	6.61E-09	1.88E-07
i1_LQ_YHS_c33923/f1p2/1823	65.6852655	659.2689951	-3.3272	6.66E-09	1.89E-07
i1_LQ_YHS_c7779/f1p0/1723	7.036833478	119.3121322	-4.0837	6.66E-09	1.89E-07
i3_LQ_YHS_c12984/f1p7/3149	15.51089416	178.9272024	-3.528	6.67E-09	1.89E-07
i2_LQ_YHS_c49547/f1p12/2170	112.7688465	841.5978723	-2.8998	6.71E-09	1.90E-07
i2_LQ_YHS_c5221/f1p0/2550	9.793796064	333.4073594	-5.0893	6.73E-09	1.91E-07
i1_HQ_YHS_c5925/f2p3/1626	160.7742481	1215.357723	-2.9183	6.74E-09	1.91E-07
i1_HQ_YHS_c2258/f3p0/1690	141.7729782	1091.327288	-2.9444	6.76E-09	1.91E-07
i3_LQ_YHS_c11415/f1p2/3155	0	44.59011428	-Inf	6.84E-09	1.93E-07
i3_LQ_YHS_c14997/f1p0/3065	4.286553523	96.70996576	-4.4958	6.88E-09	1.94E-07
i2_LQ_YHS_c42113/f1p2/2725	153.2402991	1154.359043	-2.9132	6.89E-09	1.95E-07
i2_LQ_YHS_c2871/f1p11/2406	218.925768	1855.922401	-3.0836	6.95E-09	1.96E-07
i0_LQ_YHS_c1588/f1p2/967	98.33158507	757.0867055	-2.9447	7.04E-09	1.99E-07
i1_LQ_YHS_c33567/f1p0/1417	27.54176801	395.5006801	-3.844	7.09E-09	2.00E-07
i3_LQ_YHS_c17715/f1p2/3316	279.3819497	5854.609369	-4.3893	7.12E-09	2.01E-07
i2_LQ_YHS_c7199/f1p6/2144	60.81940987	462.1430407	-2.9257	7.13E-09	2.01E-07
i2_HQ_YHS_c61784/f11p5/2705	258.9889684	7761.016488	-4.9053	7.17E-09	2.02E-07
i1_LQ_YHS_c34250/f1p6/1511	34.12248294	283.6148138	-3.0551	7.20E-09	2.03E-07
i2_HQ_YHS_c19099/f2p2/2391	30.64879822	382.3546019	-3.641	7.21E-09	2.03E-07
i2_LQ_YHS_c64483/f1p0/2009	14.09084027	734.5490684	-5.704	7.35E-09	2.07E-07
i1_LQ_YHS_c35729/f1p642/1863	0	44.89157598	-Inf	7.47E-09	2.10E-07
i2_HQ_YHS_c29867/f10p6/2428	24.07223001	301.5528949	-3.647	7.57E-09	2.13E-07
i2_LQ_YHS_c20539/f1p0/2155	150.9197458	4.746113951	4.9909	7.59E-09	2.13E-07
i2_LQ_YHS_c27060/f1p0/2435	7.954972912	182.4177032	-4.5192	7.58E-09	2.13E-07
i2_LQ_YHS_c39039/f1p1/2150	2.795592381	165.4269801	-5.8869	7.61E-09	2.13E-07
i3_LQ_YHS_c9152/f1p0/3594	10.66663145	143.6311306	-3.7512	7.66E-09	2.15E-07
i2_LQ_YHS_c42222/f1p17/2665	294.9349477	5409.271439	-4.197	7.77E-09	2.17E-07
i2_LQ_YHS_c52985/f1p0/2283	87.93476739	907.5128576	-3.3674	7.82E-09	2.19E-07
i1_HQ_YHS_c10650/f2p0/1385	104.9734897	751.8510913	-2.8404	7.88E-09	2.20E-07
i2_LQ_YHS_c19472/f1p3/2526	279.0366331	32.77599451	3.0897	7.92E-09	2.21E-07
i2_LQ_YHS_c51863/f1p0/2102	49.24816581	0	Inf	7.93E-09	2.21E-07
i2_LQ_YHS_c11712/f1p7/2182	0	218.117846	-Inf	7.96E-09	2.22E-07
i1_LQ_YHS_c10310/f1p0/1531	97.77737875	710.05772	-2.8604	7.97E-09	2.22E-07
i1_LQ_YHS_c24119/f1p0/1539	258.1701159	8210.948247	-4.9912	7.99E-09	2.23E-07
i4_LQ_YHS_c11819/f1p2/4284	18.76642988	194.9448975	-3.3768	8.18E-09	2.28E-07
i1_LQ_YHS_c3149/f1p0/1996	31.4766431	279.1097373	-3.1485	8.22E-09	2.29E-07
i2_LQ_YHS_c55941/f1p0/2077	10.93101797	142.9808525	-3.7093	8.29E-09	2.31E-07
i0_LQ_YHS_c2181/f1p9/677	213.5244232	1789.469339	-3.0671	8.34E-09	2.32E-07
i2_LQ_YHS_c10545/f2p2/2661	55.04872265	412.1171996	-2.9043	8.35E-09	2.32E-07
i1_LQ_YHS_c43659/f1p0/1039	306.9609497	4.430630517	6.1144	8.40E-09	2.33E-07
i2_LQ_YHS_c51869/f1p3/1925	19.0638909	209.4885307	-3.458	8.42E-09	2.34E-07
i2_LQ_YHS_c41592/f1p0/2186	32.32134774	275.8573496	-3.0934	8.48E-09	2.35E-07
i5_LQ_YHS_c1416/f1p1/6108	54.7286527	0.359993938	7.2482	8.56E-09	2.37E-07
i2_LQ_YHS_c24399/f1p0/2616	224.6968143	12.22415315	4.2002	8.58E-09	2.38E-07
i1_LQ_YHS_c39355/f1p0/1111	48.37277777	920.1442259	-4.2496	8.59E-09	2.38E-07
i4_LQ_YHS_c3134/f1p2/4491	3.576530766	114.1903835	-4.9967	8.66E-09	2.40E-07
i1_HQ_YHS_c10235/f3p4/1773	427.4610492	5.757098441	6.2143	8.67E-09	2.40E-07
i2_LQ_YHS_c21100/f1p4/2806	176.8587681	1445.013506	-3.0304	8.81E-09	2.43E-07
i2_LQ_YHS_c49923/f1p16/1926	2.980650142	85.24916499	-4.838	8.85E-09	2.45E-07
i1_LQ_YHS_c24387/f1p0/1759	177.5759403	1466.38813	-3.0458	8.94E-09	2.47E-07
i3_LQ_YHS_c4117/f1p4/3373	20.50875096	199.5365102	-3.2823	8.94E-09	2.47E-07
i3_LQ_YHS_c20468/f1p0/3032	18.81508363	192.505194	-3.3549	9.02E-09	2.49E-07
i2_HQ_YHS_c4394/f2p2/2904	90.88377674	649.9372666	-2.8382	9.31E-09	2.57E-07
i2_LQ_YHS_c32505/f2p9/2987	13.82564834	167.3792392	-3.5977	9.36E-09	2.58E-07
i1_HQ_YHS_c1980/f3p1/1854	625.9499393	87.15825959	2.8443	9.45E-09	2.60E-07
i2_LQ_YHS_c55379/f1p10/2266	267.4121865	3104.566266	-3.5373	9.64E-09	2.65E-07
i3_LQ_YHS_c17150/f1p0/3088	88.79517097	662.8039977	-2.9	9.70E-09	2.67E-07
i5_LQ_YHS_c3145/f1p2/5589	48.9942616	518.8977546	-3.4048	9.74E-09	2.68E-07
i2_LQ_YHS_c55866/f1p0/2880	5.991508569	180.7413878	-4.9149	9.79E-09	2.69E-07
i2_LQ_YHS_c38899/f1p0/2340	45.84064682	352.6161449	-2.9434	9.88E-09	2.71E-07
i2_LQ_YHS_c40474/f1p4/2643	81.07311253	591.2461453	-2.8665	9.92E-09	2.72E-07
i1_LQ_YHS_c19765/f1p1/1354	568.4216216	79.81367281	2.8323	9.98E-09	2.74E-07
i3_LQ_YHS_c19785/f1p0/3990	42.71415514	342.4071721	-3.0029	9.99E-09	2.74E-07
i3_LQ_YHS_c8273/f1p0/3175	70.76345032	510.7109793	-2.8514	9.99E-09	2.74E-07
i2_LQ_YHS_c37679/f1p1/2732	66.79297321	468.6533413	-2.8108	1.01E-08	2.75E-07
i3_LQ_YHS_c14450/f1p0/3032	2.780488239	81.8439335	-4.8795	1.01E-08	2.76E-07
i0_LQ_YHS_c1604/f1p1/647	0	135.2299028	-Inf	1.01E-08	2.76E-07
i1_LQ_YHS_c14643/f1p0/1186	254.9872131	2674.857472	-3.391	1.01E-08	2.77E-07
i2_HQ_YHS_c17625/f4p2/2224	73.24245889	527.5373696	-2.8485	1.03E-08	2.80E-07
i1_HQ_YHS_c29471/f8p0/1394	251.5363767	8776.14905	-5.1247	1.03E-08	2.82E-07
i3_HQ_YHS_c13827/f3p0/3058	49.4138279	513.6541251	-3.3778	1.04E-08	2.83E-07
i2_LQ_YHS_c8349/f1p0/2758	76.9711919	527.9704647	-2.7781	1.04E-08	2.83E-07
i2_LQ_YHS_c53041/f1p0/2197	83.93488739	1142.18519	-3.7664	1.04E-08	2.85E-07
i1_LQ_YHS_c38367/f1p1/1144	101.8515576	707.1608597	-2.7956	1.05E-08	2.87E-07
i3_LQ_YHS_c12101/f1p5/3312	84.68018188	617.8689841	-2.8672	1.06E-08	2.87E-07
i1_LQ_YHS_c28446/f1p0/1069	218.1081853	1919.638997	-3.1377	1.06E-08	2.89E-07
i1_LQ_YHS_c34494/f1p0/1660	8.290275064	122.120362	-3.8807	1.06E-08	2.89E-07
i2_LQ_YHS_c8021/f1p4/2211	68.91254242	488.6803817	-2.8261	1.07E-08	2.91E-07

i4_HQ_YHS_c12946/f2p1/4162	9.456424738	138.2046437	-3.8694	1.07E-08	2.91E-07
i1_LQ_YHS_c9211/f1p0/1597	49.89601638	380.405466	-2.9305	1.09E-08	2.96E-07
i0_LQ_YHS_c3427/f1p1/238	235.5111255	10.46869396	4.4916	1.10E-08	2.99E-07
i3_LQ_YHS_c18329/f1p0/3762	3.454425485	86.59285202	-4.6477	1.12E-08	3.03E-07
i1_HQ_YHS_c14306/f2p0/1375	411.7137904	56.91272727	2.8548	1.12E-08	3.05E-07
i2_HQ_YHS_c43634/f3p0/2239	5.982290026	220.1369313	-5.2016	1.13E-08	3.05E-07
i1_HQ_YHS_c29245/f15p0/1627	2540.323289	177.8873866	3.836	1.13E-08	3.06E-07
i3_LQ_YHS_c18738/f1p1/3612	3.084309964	81.2875731	-4.72	1.14E-08	3.08E-07
i2_HQ_YHS_c29961/f3p1/2027	369.5843514	10.14934016	5.1864	1.15E-08	3.10E-07
i1_LQ_YHS_c9573/f1p1/1880	197.6844871	1686.965555	-3.0932	1.17E-08	3.16E-07
i3_LQ_YHS_c12855/f1p0/3112	131.4273585	945.8882817	-2.8474	1.18E-08	3.19E-07
i2_LQ_YHS_c12092/f1p1/2276	16.57212862	176.5061381	-3.4129	1.19E-08	3.21E-07
i3_LQ_YHS_c8629/f1p2/3177	0.272341299	49.69610241	-7.5116	1.22E-08	3.29E-07
i2_LQ_YHS_c23033/f1p1/2177	34.5618949	370.3391042	-3.4216	1.22E-08	3.30E-07
i1_HQ_YHS_c17169/f2p0/1620	90.64292424	623.3303415	-2.7817	1.23E-08	3.32E-07
i3_LQ_YHS_c1140/f9p0/3612	299.7447394	4634.99992	-3.9508	1.25E-08	3.36E-07
i3_LQ_YHS_c17105/f1p0/3643	138.4621851	0	Inf	1.26E-08	3.39E-07
i4_HQ_YHS_c2302/f2p2/4419	0.817023896	91.47925151	-6.8069	1.27E-08	3.43E-07
i3_LQ_YHS_c5637/f1p0/3924	30.58375982	271.3463095	-3.1493	1.28E-08	3.43E-07
i0_LQ_YHS_c1760/f1p0/717	1327.594351	0	Inf	1.28E-08	3.44E-07
i0_HQ_YHS_c3660/f8p9/613	207.5526227	10924.88375	-5.718	1.29E-08	3.47E-07
i1_LQ_YHS_c28424/f1p0/1165	175.1431872	1279.735452	-2.8692	1.31E-08	3.52E-07
i2_LQ_YHS_c36134/f1p8/2369	7.080873763	118.0568338	-4.0594	1.31E-08	3.52E-07
i3_HQ_YHS_c15359/f9p0/3080	146.5062797	1031.645272	-2.8159	1.34E-08	3.59E-07
i1_LQ_YHS_c3283/f1p5/1776	9.930200081	138.8711553	-3.8058	1.35E-08	3.63E-07
i5_LQ_YHS_c3005/f1p0/5382	17.11265612	186.3475812	-3.4449	1.36E-08	3.63E-07
i3_LQ_YHS_c8912/f1p0/3433	60.18505264	513.5740996	-3.0931	1.36E-08	3.64E-07
i2_LQ_YHS_c33046/f1p1/2185	11.26504798	328.3424475	-4.8653	1.36E-08	3.65E-07
i1_LQ_YHS_c6146/f1p0/1663	69.47833445	497.3737845	-2.8397	1.38E-08	3.68E-07
i3_LQ_YHS_c19584/f1p2/3267	15.36653537	171.8097499	-3.4829	1.38E-08	3.69E-07
i0_LQ_YHS_c1202/f1p1/884	105.6853171	728.5602615	-2.7853	1.40E-08	3.73E-07
i1_LQ_YHS_c22709/f1p1/1734	46.90105075	368.6085269	-2.9744	1.41E-08	3.75E-07
i2_LQ_YHS_c24012/f1p2/2820	3.596248367	80.86028107	-4.4909	1.42E-08	3.79E-07
i3_HQ_YHS_c13577/f2p2/3253	55.30149116	531.2527473	-3.264	1.44E-08	3.84E-07
i3_LQ_YHS_c19705/f1p3/3795	62.79522346	470.0194615	-2.904	1.45E-08	3.87E-07
i1_LQ_YHS_c13500/f1p0/1221	181.3232229	1340.417553	-2.886	1.46E-08	3.90E-07
i1_LQ_YHS_c2939/f1p4/1777	196.6232849	11221.41665	-5.8347	1.46E-08	3.90E-07
i2_HQ_YHS_c2759/f4p9/2707	145.3226168	1085.294364	-2.9008	1.46E-08	3.90E-07
i1_LQ_YHS_c18668/f1p0/1916	21885.59226	0.353872233	15.916	1.48E-08	3.94E-07
i2_LQ_YHS_c8308/f1p1/2278	39.02795926	306.8793891	-2.9751	1.50E-08	3.98E-07
i0_LQ_YHS_c391/f2p0/675	249.6262549	2229.544276	-3.1589	1.51E-08	4.00E-07
i1_LQ_YHS_c12442/f1p0/1604	22.57285573	659.8095072	-4.8694	1.51E-08	4.00E-07
i2_HQ_YHS_c32534/f3p2/2681	51.37105959	396.0544755	-2.9467	1.53E-08	4.07E-07
i2_HQ_YHS_c57895/f2p0/2055	135.3477817	1782.635438	-3.7193	1.55E-08	4.10E-07
i0_LQ_YHS_c3102/f1p1/501	414.75139	1.864503878	7.7973	1.55E-08	4.11E-07
i5_LQ_YHS_c3855/f1p0/5230	10.89746837	137.147689	-3.6537	1.55E-08	4.11E-07
i2_LQ_YHS_c53790/f1p1/2501	252.5803361	28.94852691	3.1252	1.56E-08	4.12E-07
i1_HQ_YHS_c4312/f3p9/1858	0	83.33718672	-Inf	1.59E-08	4.21E-07
i2_LQ_YHS_c54414/f1p0/2325	222.4236426	22.42080838	3.3104	1.59E-08	4.21E-07
i2_LQ_YHS_c38795/f1p0/2678	107.2102372	2.477105631	5.4356	1.60E-08	4.22E-07
i3_LQ_YHS_c6172/f1p0/3560	14.36906717	179.7133424	-3.6447	1.60E-08	4.22E-07
i3_LQ_YHS_c11743/f1p0/3278	248.5505518	2311.184792	-3.217	1.61E-08	4.26E-07
i1_LQ_YHS_c38923/f1p0/1273	114.7026446	733.9846529	-2.6779	1.62E-08	4.26E-07
i5_LQ_YHS_c3098/f1p9/5817	47.46893708	358.2618731	-2.916	1.62E-08	4.28E-07
i2_LQ_YHS_c32767/f1p6/2359	95.50979589	627.3080543	-2.7155	1.63E-08	4.30E-07
i2_LQ_YHS_c24775/f1p6/2351	107.5532212	721.346718	-2.7456	1.64E-08	4.33E-07
i2_LQ_YHS_c14389/f1p4/2042	230.4112568	24.93183967	3.2081	1.65E-08	4.35E-07
i2_HQ_YHS_c29614/f18p16/2708	316.3005336	5224.160886	-4.0458	1.66E-08	4.37E-07
i3_LQ_YHS_c17342/f1p0/3919	103.5952956	685.0359039	-2.7252	1.68E-08	4.41E-07
i2_LQ_YHS_c22350/f1p0/2230	39.40316335	301.8994648	-2.9377	1.70E-08	4.47E-07
i2_HQ_YHS_c10845/f4p0/2477	0	50.60969284	-Inf	1.70E-08	4.48E-07
i1_LQ_YHS_c38732/f1p2/1765	5.863526061	652.1502121	-6.7973	1.71E-08	4.50E-07
i2_LQ_YHS_c29136/f1p5/1927	1.316394066	65.12959247	-5.6286	1.72E-08	4.51E-07
i2_LQ_YHS_c59433/f1p78/2041	39916.10649	0	Inf	1.72E-08	4.51E-07
i1_LQ_YHS_c8514/f2p0/1596	303.4822398	4546.162512	-3.905	1.73E-08	4.53E-07
i1_LQ_YHS_c36744/f1p0/1866	1646.90607	7.061291341	7.8656	1.73E-08	4.54E-07
i2_LQ_YHS_c20143/f1p2/2211	65.37224198	750.9801578	-3.522	1.74E-08	4.56E-07
i2_LQ_YHS_c50959/f1p9/2486	297.4995416	7272.830464	-4.6116	1.74E-08	4.56E-07
i1_HQ_YHS_c2579/f3p0/1720	1.361706493	286.7017946	-7.718	1.75E-08	4.59E-07
i3_HQ_YHS_c2323/f2p1/3637	71.0799516	1933.280685	-4.7655	1.76E-08	4.60E-07
i2_LQ_YHS_c7612/f1p1/2363	233.376877	25.96565242	3.168	1.77E-08	4.62E-07
i3_LQ_YHS_c19666/f1p19/3505	110.288397	738.8703003	-2.744	1.79E-08	4.68E-07
i1_LQ_YHS_c35898/f1p2/1922	15.75048289	325.0636376	-4.3673	1.81E-08	4.71E-07
i1_LQ_YHS_c32220/f1p0/1605	256.3451583	2349.144232	-3.196	1.82E-08	4.74E-07
i3_LQ_YHS_c3332/f1p0/3938	217.1914783	1879.364927	-3.1132	1.85E-08	4.82E-07
i1_LQ_YHS_c14373/f1p0/1362	5.333947607	101.130997	-4.2449	1.85E-08	4.82E-07
i1_HQ_YHS_c39898/f3p0/1865	164.8466533	1252.321386	-2.9254	1.85E-08	4.82E-07
i10_LQ_YHS_c5/f1p0/10576	11.55756524	147.7201729	-3.676	1.85E-08	4.82E-07
i2_LQ_YHS_c33547/f1p2/2092	88.06865224	816.7931618	-3.2133	1.86E-08	4.83E-07
i5_LQ_YHS_c3094/f1p3/5645	96.02328648	674.3486114	-2.812	1.86E-08	4.84E-07
i1_LQ_YHS_c4589/f1p1/1820	2.610534621	716.5902064	-8.1007	1.86E-08	4.85E-07
i2_HQ_YHS_c60386/f6p3/2981	1.361706493	224.9215862	-7.3679	1.90E-08	4.93E-07
i2_LQ_YHS_c37292/f1p3/2286	311.3595654	5853.716823	-4.2327	1.91E-08	4.95E-07
i1_LQ_YHS_c31838/f1p0/1868	36.09214644	377.0910696	-3.3852	1.95E-08	5.07E-07
i1_LQ_YHS_c26370/f1p0/1832	100.1093646	1411.919437	-3.818	1.97E-08	5.11E-07
i4_LQ_YHS_c8459/f1p0/4671	931.643759	133.8726324	2.7989	1.99E-08	5.17E-07
i1_LQ_YHS_c14330/f1p1/1374	46.64002229	21273.70335	-8.8333	2.00E-08	5.18E-07
i1_LQ_YHS_c21631/f1p0/1812	21.70638106	318.7781458	-3.8764	2.03E-08	5.27E-07
i1_LQ_YHS_c3080/f1p0/1547	14.67416104	309.7414869	-4.3997	2.04E-08	5.28E-07

i3_LQ_YHS_c12241/f1p0/4182	7.866425606	163.9168621	-4.3811	2.04E-08	5.28E-07
i1_LQ_YHS_c13548/f1p0/1158	6071.317112	205.8368225	4.8824	2.07E-08	5.35E-07
i0_LQ_YHS_c1945/f1p1/969	169.5780377	1297.964655	-2.9362	2.08E-08	5.37E-07
i2_LQ_YHS_c19516/f1p3/2514	29.69109552	313.6485329	-3.401	2.10E-08	5.44E-07
i6_LQ_YHS_c632/f1p0/6405	2.632788131	77.95426327	-4.888	2.11E-08	5.44E-07
i0_LQ_YHS_c2064/f1p0/692	129.8769729	840.3108134	-2.6938	2.11E-08	5.44E-07
i1_LQ_YHS_c34183/f1p0/1719	35.98355123	486.4539501	-3.7569	2.11E-08	5.45E-07
i2_HQ_YHS_c2277/f3p3/2231	313.317338	5515.655937	-4.1378	2.15E-08	5.54E-07
i3_LQ_YHS_c4637/f1p0/3262	183.9512695	1304.891429	-2.8265	2.28E-08	5.88E-07
i1_LQ_YHS_c27444/f1p0/1081	501.5832821	48.6534436	3.3659	2.33E-08	6.00E-07
i1_LQ_YHS_c11083/f1p4/1541	72.50891048	497.2348438	-2.7777	2.33E-08	6.00E-07
i2_HQ_YHS_c10038/f2p5/2602	2.995754284	263.933906	-6.4611	2.34E-08	6.02E-07
i2_LQ_YHS_c53602/f1p2/2338	305.1457168	7551.425979	-4.6292	2.36E-08	6.06E-07
i2_LQ_YHS_c22428/f1p2/2288	130.9232049	924.9509834	-2.8207	2.38E-08	6.12E-07
i2_LQ_YHS_c38578/f1p5/2386	163.1475504	1225.767308	-2.9094	2.39E-08	6.14E-07
i1_LQ_YHS_c38222/f1p0/1253	173.446595	1262.781821	-2.864	2.40E-08	6.17E-07
i2_LQ_YHS_c40376/f1p0/2417	167.119971	12.12123016	3.7853	2.41E-08	6.17E-07
i2_LQ_YHS_c59443/f1p10/1805	321.3456021	4703.248893	-3.8715	2.41E-08	6.17E-07
i2_LQ_YHS_c55352/f1p0/2540	154.5369014	13.59959471	3.5063	2.44E-08	6.25E-07
i2_LQ_YHS_c49535/f1p31/2129	66.2546262	1.427732343	5.5362	2.45E-08	6.27E-07
i2_LQ_YHS_c35391/f1p1/2474	61.26981275	421.9949285	-2.784	2.46E-08	6.30E-07
i2_LQ_YHS_c26848/f1p0/2462	10.8181396	132.207933	-3.6113	2.46E-08	6.30E-07
i3_LQ_YHS_c5467/f1p0/3307	8.145916272	118.2036745	-3.8591	2.48E-08	6.34E-07
i1_LQ_YHS_c32757/f1p0/1698	598.3722665	90.55282602	2.7242	2.48E-08	6.34E-07
i1_LQ_YHS_c32784/f1p0/1941	4.856830947	589.6868157	-6.9238	2.51E-08	6.40E-07
i2_LQ_YHS_c39220/f1p2/2697	5.360814575	98.79034722	-4.2038	2.51E-08	6.41E-07
i2_LQ_YHS_c62908/f1p4/2026	0.272341299	256.4826879	-9.8792	2.51E-08	6.41E-07
i2_HQ_YHS_c20758/f2p1/2126	1.891284948	69.80058739	-5.2058	2.53E-08	6.44E-07
i2_HQ_YHS_c26070/f2p0/2256	89.73700599	595.7811491	-2.731	2.53E-08	6.44E-07
i1_LQ_YHS_c17419/f1p0/1473	290.7737452	8070.105695	-4.7946	2.55E-08	6.51E-07
i1_LQ_YHS_c5460/f1p10/1478	126.4994516	4.790624455	4.7228	2.56E-08	6.51E-07
i1_LQ_YHS_c22373/f1p1/1656	32.39733519	258.0833284	-2.9939	2.59E-08	6.59E-07
i2_LQ_YHS_c56038/f1p4/2138	215.7061754	1806.68831	-3.0662	2.65E-08	6.75E-07
i3_LQ_YHS_c4511/f1p0/3874	5.613438273	103.6687269	-4.207	2.66E-08	6.77E-07
i3_LQ_YHS_c9658/f1p9/3369	5.71916727	103.5135132	-4.1779	2.67E-08	6.78E-07
i2_HQ_YHS_c60454/f51p10/2279	18.84022847	5419.295341	-8.1681	2.68E-08	6.80E-07
i3_LQ_YHS_c4528/f1p4/3218	137.8075645	7.95545087	4.1146	2.68E-08	6.81E-07
i2_LQ_YHS_c59572/f1p1/2060	60.71302028	421.4424027	-2.7953	2.69E-08	6.82E-07
i2_LQ_YHS_c12729/f1p0/2088	9.915095939	131.3794932	-3.728	2.70E-08	6.85E-07
i1_HQ_YHS_c4201/f2p0/1889	0	115.760928	-Inf	2.73E-08	6.91E-07
i2_LQ_YHS_c57738/f2p72/2025	40796.54528	0	Inf	2.73E-08	6.92E-07
i2_HQ_YHS_c57772/f4p8/2071	334.9467503	5030.311238	-3.9086	2.74E-08	6.92E-07
i3_LQ_YHS_c22564/f1p0/3003	19.92543856	184.8062213	-3.2133	2.75E-08	6.95E-07
i2_LQ_YHS_c56265/f1p3/2710	255.1628585	2280.229316	-3.1597	2.76E-08	6.99E-07
i2_LQ_YHS_c36830/f1p7/2351	180.615839	1294.405675	-2.8413	2.78E-08	7.02E-07
i3_LQ_YHS_c11984/f1p100/3321	56.89181221	406.8594286	-2.8382	2.78E-08	7.02E-07
i2_LQ_YHS_c60030/f1p1/2074	374.372708	14.13707744	4.7269	2.81E-08	7.10E-07
i1_LQ_YHS_c40688/f17p0/1373	199.0323939	12088.17747	-5.9244	2.82E-08	7.12E-07
i1_LQ_YHS_c28133/f1p0/1119	170.1179058	1190.285677	-2.8067	2.82E-08	7.12E-07
i1_LQ_YHS_c7919/f1p1/1602	23.39749573	214.07181	-3.1937	2.83E-08	7.13E-07
i1_LQ_YHS_c32223/f1p3/1827	36.14566976	296.5694188	-3.0365	2.89E-08	7.27E-07
i3_HQ_YHS_c15734/f2p0/3046	32.90926522	264.4795272	-3.0066	2.89E-08	7.29E-07
i1_HQ_YHS_c40179/f21p4/1892	275.6225212	2648.422258	-3.2644	2.91E-08	7.31E-07
i2_LQ_YHS_c59664/f1p0/2029	0.529578455	51.33137883	-6.5989	2.91E-08	7.32E-07
i1_LQ_YHS_c35252/f1p0/1413	7.157666617	364.2447143	-5.6693	2.92E-08	7.33E-07
i2_LQ_YHS_c40886/f1p2/2607	231.2850484	1980.793521	-3.0983	2.92E-08	7.35E-07
i2_LQ_YHS_c20964/f1p14/2147	134.1146082	832.0619673	-2.6332	2.99E-08	7.52E-07
i1_HQ_YHS_c2465/f5p0/1911	168.1406668	1156.783618	-2.7824	3.00E-08	7.52E-07
i1_HQ_YHS_c37893/f2p0/1314	310.2687808	3200.73283	-3.3668	3.03E-08	7.60E-07
i1_LQ_YHS_c9028/f1p0/2000	6.710758242	153.0282169	-4.5112	3.03E-08	7.61E-07
i1_LQ_YHS_c33379/f1p6/2001	5.293248638	118.689106	-4.4869	3.12E-08	7.83E-07
i2_LQ_YHS_c14373/f1p0/2046	45.16683764	329.68669	-2.8678	3.14E-08	7.86E-07
i1_LQ_YHS_c23276/f1p2/1856	319.6395352	6673.747214	-4.384	3.14E-08	7.87E-07
i1_HQ_YHS_c17181/f3p11/1783	236.8277254	1910.644303	-3.0121	3.15E-08	7.88E-07
i1_LQ_YHS_c3097/f1p1/1490	8.110289123	121.5132089	-3.9052	3.17E-08	7.94E-07
i3_HQ_YHS_c1812/f3p0/3318	10.07662805	229.5039612	-4.5094	3.18E-08	7.96E-07
i2_HQ_YHS_c11284/f2p7/2146	1321.897868	100.9367226	3.7111	3.19E-08	7.97E-07
i2_LQ_YHS_c21573/f1p4/3132	32.87189919	268.8211378	-3.0317	3.20E-08	7.99E-07
i3_LQ_YHS_c7350/f1p0/3855	4.357460777	91.41124088	-4.3908	3.20E-08	8.00E-07
i4_LQ_YHS_c9889/f1p1/5016	37.97007449	439.0819524	-3.5316	3.22E-08	8.04E-07
i2_LQ_YHS_c38829/f1p7/2482	299.600985	2990.764207	-3.3194	3.24E-08	8.07E-07
i2_LQ_YHS_c6604/f1p0/2228	3.896720402	86.27239289	-4.4686	3.24E-08	8.07E-07
i1_LQ_YHS_c34311/f1p0/1804	30.66089134	739.1906924	-4.5915	3.24E-08	8.07E-07
i3_HQ_YHS_c7787/f2p0/3573	77.71425566	1079.710786	-3.7963	3.25E-08	8.08E-07
i5_LQ_YHS_c2351/f1p1/5623	103.3491188	1024.620443	-3.3095	3.36E-08	8.36E-07
i2_LQ_YHS_c9826/f1p5/2703	79.51204117	547.5017652	-2.7836	3.37E-08	8.38E-07
i2_LQ_YHS_c15174/f1p1/1938	141.3656665	1023.676976	-2.8563	3.39E-08	8.44E-07
i3_LQ_YHS_c4725/f1p0/3377	16.0883377	155.3770853	-3.2717	3.41E-08	8.47E-07
i2_LQ_YHS_c22114/f1p2/2315	31.06503157	249.8568243	-3.0077	3.43E-08	8.51E-07
i2_LQ_YHS_c36331/f1p1/2643	0	51.07434855	-Inf	3.45E-08	8.57E-07
i1_LQ_YHS_c11348/f1p8/1807	10.96710348	135.7186909	-3.6294	3.47E-08	8.61E-07
i1_LQ_YHS_c17980/f1p0/1533	68.95149414	681.512779	-3.3051	3.47E-08	8.61E-07
i3_LQ_YHS_c10001/f1p1/3722	28.18977176	236.2044658	-3.0668	3.50E-08	8.68E-07
i2_HQ_YHS_c61515/f5p3/2150	2433.279927	273.2152776	3.1548	3.56E-08	8.82E-07
i4_LQ_YHS_c11698/f1p0/4935	2.153135562	67.51665085	-4.9707	3.57E-08	8.83E-07
i1_HQ_YHS_c33768/f2p0/1911	654.9572854	10.87825431	5.9119	3.57E-08	8.84E-07
i2_LQ_YHS_c10328/f7p3/2320	153.3291025	1019.312515	-2.7329	3.62E-08	8.95E-07
i1_LQ_YHS_c6835/f1p2/1908	114.1485652	722.9562407	-2.663	3.63E-08	8.97E-07
i2_LQ_YHS_c1624/f24p2/2259	202.2465713	1479.324295	-2.8708	3.63E-08	8.97E-07

i1_LQ_YHS_c5246/f1p59/1414	110.5076159	701.2629214	-2.6658	3.64E-08	8.99E-07
i2_LQ_YHS_c48663/f1p0/2603	0	39.58246624	-Inf	3.64E-08	8.99E-07
i4_LQ_YHS_c12693/f1p0/4795	16.49914382	177.5037332	-3.4274	3.64E-08	8.99E-07
i2_LQ_YHS_c4122/f1p1/3058	37.95624249	285.2144676	-2.9096	3.77E-08	9.30E-07
i3_HQ_YHS_c2471/f2p3/3144	0	265.6092419	-Inf	3.78E-08	9.31E-07
i2_HQ_YHS_c46497/f5p82/2023	6773.662187	0	Inf	3.78E-08	9.32E-07
i1_LQ_YHS_c7952/f1p2/1926	9.386789626	124.2762959	-3.7268	3.80E-08	9.36E-07
i1_LQ_YHS_c33999/f1p1/1670	25.15411554	220.0670215	-3.1291	3.82E-08	9.41E-07
i3_HQ_YHS_c1221/f7p4/3416	137.0105885	901.3377873	-2.7178	3.83E-08	9.42E-07
i2_HQ_YHS_c35171/f4p1/2522	24.16285486	259.0505088	-3.4224	3.85E-08	9.45E-07
i1_LQ_YHS_c18054/f1p35/1812	330.5447322	4107.022874	-3.6352	3.85E-08	9.46E-07
i2_LQ_YHS_c56457/f1p6/2215	47.3123571	336.0021211	-2.8282	3.86E-08	9.48E-07
i2_LQ_YHS_c2826/f1p1/2723	0	477.7303794	-Inf	3.90E-08	9.57E-07
i2_LQ_YHS_c23582/f1p6/2881	49.57996509	550.1398075	-3.472	3.92E-08	9.62E-07
i2_LQ_YHS_c51419/f1p3/2117	1156.198669	166.5022999	2.7958	3.93E-08	9.64E-07
i1_LQ_YHS_c38736/f2p0/1195	289.3311134	2587.273896	-3.1606	3.95E-08	9.67E-07
i2_LQ_YHS_c7322/f1p0/2096	4.508968936	92.56851286	-4.3597	3.96E-08	9.71E-07
i1_LQ_YHS_c23947/f1p0/1748	133.4939908	907.2524224	-2.7647	3.97E-08	9.71E-07
i2_LQ_YHS_c39294/f1p2/2135	0	39.45560966	-Inf	3.98E-08	9.73E-07
i1_LQ_YHS_c12832/f1p0/1576	70.72001321	469.1928407	-2.73	4.00E-08	9.79E-07
i2_LQ_YHS_c18996/f1p8/2815	323.3400143	3765.189299	-3.5416	4.01E-08	9.79E-07
i1_LQ_YHS_c35074/f1p2/1676	236.6315247	1811.297138	-2.9363	4.08E-08	9.97E-07
i2_LQ_YHS_c43724/f1p2/2055	173.6450803	1233.962628	-2.8291	4.08E-08	9.97E-07
i2_LQ_YHS_c21067/f1p3/2390	302.6533394	3159.760305	-3.3841	4.08E-08	9.97E-07
i1_LQ_YHS_c33850/f1p4/1428	187.7987869	20.34540262	3.2064	4.11E-08	1.00E-06
i1_LQ_YHS_c5707/f1p3/1575	588.0426269	90.10432363	2.7063	4.16E-08	1.01E-06
i2_HQ_YHS_c17673/f3p3/2381	0	39.12120788	-Inf	4.18E-08	1.02E-06
i2_LQ_YHS_c49702/f1p2/2930	2.118313819	142.1502405	-6.0684	4.21E-08	1.03E-06
i0_HQ_YHS_c207/f4p0/930	162.1823931	14464.97261	-6.4788	4.26E-08	1.04E-06
i2_LQ_YHS_c9797/f1p0/2369	50.42526453	351.1400773	-2.7998	4.30E-08	1.05E-06
i3_HQ_YHS_c1720/f4p0/3384	231.5809165	1769.696341	-2.9339	4.32E-08	1.05E-06
i2_LQ_YHS_c34994/f1p3/2777	167.6662058	1148.065845	-2.7755	4.33E-08	1.05E-06
i2_LQ_YHS_c3277/f1p0/2773	21.10876156	363.9148581	-4.1077	4.35E-08	1.06E-06
i2_LQ_YHS_c3970/f1p2/2122	433.7688938	63.92844232	2.7624	4.38E-08	1.06E-06
i4_LQ_YHS_c13556/f1p4/4058	25.93124587	225.8778308	-3.1228	4.42E-08	1.07E-06
i1_HQ_YHS_c1242/f8p0/1740	224.3985718	1716.149967	-2.935	4.45E-08	1.08E-06
i3_LQ_YHS_c5550/f1p0/3205	22.52420199	203.0588002	-3.1723	4.48E-08	1.09E-06
i2_LQ_YHS_c53556/f1p1/2815	5.240786843	214.5906568	-5.3557	4.49E-08	1.09E-06
i2_LQ_YHS_c39601/f1p2/2654	25.89595739	219.1565888	-3.0812	4.63E-08	1.12E-06
i2_LQ_YHS_c12329/f1p8/2154	286.7933853	2563.70783	-3.1601	4.65E-08	1.13E-06
i1_LQ_YHS_c23251/f1p0/1734	137.2447344	0	Inf	4.72E-08	1.14E-06
i1_LQ_YHS_c33041/f1p0/1592	13.05727819	226.4993193	-4.1166	4.73E-08	1.14E-06
i1_HQ_YHS_c40970/f14p1/1343	177.9991698	13852.25846	-6.2821	4.74E-08	1.15E-06
i4_LQ_YHS_c4567/f1p15/4388	16.5629017	166.3340895	-3.3281	4.77E-08	1.15E-06
i6_LQ_YHS_c882/f1p0/6294	19.94895584	186.9388144	-3.2282	4.78E-08	1.15E-06
i4_LQ_YHS_c12066/f1p1/4492	91.80271321	585.1589478	-2.6722	4.88E-08	1.18E-06
i3_LQ_YHS_c19845/f1p0/3095	3.06793368	77.67088762	-4.662	4.94E-08	1.19E-06
i1_LQ_YHS_c38230/f1p26/1149	71.83545672	460.0516805	-2.679	4.95E-08	1.19E-06
i2_HQ_YHS_c17162/f3p0/2729	53.76507278	638.3259102	-3.5696	5.00E-08	1.20E-06
i5_HQ_YHS_c660/f2p0/5263	1275.996026	5.039915138	7.984	5.00E-08	1.20E-06
i2_HQ_YHS_c60460/f7p0/2082	6.337301405	136.7436972	-4.4315	5.07E-08	1.22E-06
i0_LQ_YHS_c1447/f1p1/660	67.99475841	624.7258018	-3.1997	5.11E-08	1.23E-06
i3_LQ_YHS_c6074/f1p0/3788	11.98301714	629.40442	-5.7149	5.11E-08	1.23E-06
i2_LQ_YHS_c11014/f1p1/2547	32.96473803	318.2667282	-3.2712	5.12E-08	1.23E-06
i0_LQ_YHS_c541/f1p0/923	345.7706953	6056.283824	-4.1305	5.14E-08	1.24E-06
i2_LQ_YHS_c51602/f1p2/2961	0	38.62874822	-Inf	5.16E-08	1.24E-06
i1_HQ_YHS_c19660/f2p0/1830	225.599311	12062.96907	-5.7407	5.29E-08	1.27E-06
i1_LQ_YHS_c14556/f1p2/1369	28.44815299	224.8399945	-2.9825	5.30E-08	1.27E-06
i2_LQ_YHS_c18515/f1p2/2771	29.94705216	440.3633061	-3.8782	5.32E-08	1.28E-06
i3_LQ_YHS_c11236/f1p1/3155	352.6447353	50.95250722	2.791	5.34E-08	1.28E-06
i1_LQ_YHS_c21617/f1p3/1390	0	78.91101935	-Inf	5.40E-08	1.29E-06
i1_LQ_YHS_c14269/f1p0/1072	397.8257807	60.75571587	2.711	5.41E-08	1.30E-06
i2_LQ_YHS_c36784/f1p0/2596	76.91109726	485.3405095	-2.6577	5.41E-08	1.30E-06
i1_LQ_YHS_c17113/f2p0/1960	167.5811601	1455.330365	-3.1184	5.43E-08	1.30E-06
i1_LQ_YHS_c25982/f1p4/1772	147.6159453	0	Inf	5.45E-08	1.30E-06
i2_LQ_YHS_c11081/f1p1/2175	0	80.03717023	-Inf	5.45E-08	1.30E-06
i1_LQ_YHS_c35939/f1p0/1945	128.4502029	10.36411241	3.6315	5.50E-08	1.31E-06
i2_LQ_YHS_c37332/f1p0/2683	0	90.59042206	-Inf	5.55E-08	1.32E-06
i1_HQ_YHS_c26739/f17p0/1213	53.71133047	6941.481804	-7.0139	5.56E-08	1.33E-06
i1_HQ_YHS_c8374/f2p1/1969	9.03846088	135.5972831	-3.9071	5.56E-08	1.33E-06
i3_LQ_YHS_c6618/f1p3/3242	182.7493599	20.69315315	3.1426	5.56E-08	1.33E-06
i2_LQ_YHS_c52363/f1p5/2512	322.6396278	3795.174435	-3.5562	5.57E-08	1.33E-06
i4_LQ_YHS_c11853/f1p0/4988	96.98627998	621.1660345	-2.6791	5.59E-08	1.33E-06
i1_LQ_YHS_c25805/f1p0/1403	20.24182015	180.8803345	-3.1596	5.63E-08	1.34E-06
i7_LQ_YHS_c228/f1p0/7434	100.1557301	646.9910877	-2.6915	5.65E-08	1.34E-06
i3_LQ_YHS_c11098/f1p3/3227	112.3654003	2232.626238	-4.3125	5.66E-08	1.35E-06
i5_LQ_YHS_c4877/f1p0/5069	322.4546597	3508.932054	-3.4439	5.68E-08	1.35E-06
i1_LQ_YHS_c14079/f1p3/1296	537.3180393	10.51486303	5.6753	5.74E-08	1.36E-06
i2_LQ_YHS_c7194/f1p0/2236	150.1142981	1002.093146	-2.7389	5.81E-08	1.38E-06
i1_LQ_YHS_c19923/f1p0/1958	71.83117356	458.0866259	-2.6729	5.83E-08	1.38E-06
i1_LQ_YHS_c27877/f1p0/1083	301.7116037	9612.58594	-4.9937	5.84E-08	1.39E-06
i2_LQ_YHS_c51327/f1p3/2579	0.719249673	48.36920102	-6.0715	5.86E-08	1.39E-06
i4_LQ_YHS_c13123/f1p0/4059	220.8715241	1700.022788	-2.9443	5.89E-08	1.40E-06
i1_LQ_YHS_c34754/f1p1/1459	0	176.9777418	-Inf	5.92E-08	1.40E-06
i1_HQ_YHS_c2586/f2p3/1851	213.4725311	26.33389964	3.0191	5.97E-08	1.41E-06
i1_HQ_YHS_c37675/f3p0/1260	342.9032227	3681.698012	-3.4245	5.97E-08	1.41E-06
i3_LQ_YHS_c10195/f1p3/3018	161.0490822	1111.844818	-2.7874	6.02E-08	1.42E-06
i2_LQ_YHS_c58267/f1p0/2069	225.3953304	0	Inf	6.02E-08	1.42E-06
i2_LQ_YHS_c29385/f1p5/2029	294.8739986	2655.377869	-3.1707	6.11E-08	1.44E-06

i2_LQ_YHS_c38868/f1p1/3001	85.92934868	555.1888455	-2.6918	6.12E-08	1.45E-06
i4_LQ_YHS_c3305/f1p0/4780	99.22030607	654.9548698	-2.7227	6.17E-08	1.46E-06
i2_HQ_YHS_c62587/f2p2/2435	111.9038139	1.061616699	6.7199	6.20E-08	1.46E-06
i1_LQ_YHS_c6201/f1p0/1703	161.809652	11.70282374	3.7894	6.25E-08	1.47E-06
i2_LQ_YHS_c21095/f1p0/2207	58.84975475	377.8131149	-2.6826	6.26E-08	1.47E-06
i0_LQ_YHS_c2081/f1p0/849	55.33028249	371.7500916	-2.7482	6.34E-08	1.49E-06
i1_LQ_YHS_c22482/f1p1/1821	351.1470484	4674.48168	-3.7347	6.36E-08	1.50E-06
i1_LQ_YHS_c36470/f1p4/1811	359.5900464	5912.566955	-4.0394	6.40E-08	1.51E-06
i2_LQ_YHS_c50364/f1p3/2250	233.6753995	1679.287309	-2.8453	6.43E-08	1.51E-06
i1_HQ_YHS_c1374/f13p0/1977	267.9858232	2118.009479	-2.9825	6.47E-08	1.52E-06
i2_HQ_YHS_c10893/f3p2/2913	20.62243473	178.4153118	-3.113	6.54E-08	1.53E-06
i3_LQ_YHS_c15591/f3p2/3148	99.91842957	622.2553761	-2.6387	6.53E-08	1.53E-06
i5_LQ_YHS_c3308/f1p3/6041	26.63999648	220.9090111	-3.0518	6.53E-08	1.53E-06
i1_LQ_YHS_c5043/f1p9/1917	38.54082702	275.6710927	-2.8385	6.55E-08	1.54E-06
i3_HQ_YHS_c2436/f2p0/3673	17.77897726	317.5309749	-4.1587	6.57E-08	1.54E-06
i1_LQ_YHS_c38274/f1p1/1214	0	98.90527409	-Inf	6.77E-08	1.59E-06
i2_LQ_YHS_c26771/f1p12/2606	2.723412986	85.26014263	-4.9684	6.78E-08	1.59E-06
i2_LQ_YHS_c5801/f1p0/2491	42.76857479	293.0993021	-2.7768	6.81E-08	1.60E-06
i1_HQ_YHS_c7485/f4p0/1887	28.08438143	223.8500192	-2.9947	6.89E-08	1.61E-06
i1_LQ_YHS_c12225/f1p0/1656	298.9037494	2567.416342	-3.1026	6.91E-08	1.62E-06
i3_LQ_YHS_c19267/f1p0/3348	291.010634	2476.917315	-3.0894	6.91E-08	1.62E-06
i1_HQ_YHS_c17175/f3p0/1716	127.1152101	793.2565879	-2.6417	6.98E-08	1.63E-06
i2_LQ_YHS_c37746/f1p9/2934	38.46265071	2908.279643	-6.2406	7.01E-08	1.64E-06
i0_LQ_YHS_c3450/f1p0/283	5.848421919	96.50633046	-4.0445	7.10E-08	1.66E-06
i2_HQ_YHS_c4687/f2p2/2239	8.714921554	117.339217	-3.7511	7.11E-08	1.66E-06
i1_HQ_YHS_c2960/f2p0/1561	248.671939	1875.245127	-2.9148	7.13E-08	1.66E-06
i2_LQ_YHS_c25823/f1p6/2714	297.9124541	2836.648994	-3.2512	7.16E-08	1.67E-06
i1_LQ_YHS_c19261/f1p0/1739	155.8877953	1.112248908	7.1309	7.17E-08	1.67E-06
i1_LQ_YHS_c26362/f1p0/1521	100.2034755	612.756793	-2.6124	7.25E-08	1.69E-06
i2_LQ_YHS_c14271/f1p1/2048	28.0176209	220.2694317	-2.9749	7.30E-08	1.70E-06
i3_LQ_YHS_c3765/f1p10/3635	5.114068103	93.73641034	-4.1961	7.33E-08	1.71E-06
i4_LQ_YHS_c8373/f1p0/4184	64.92485849	421.8765241	-2.7	7.33E-08	1.71E-06
i1_LQ_YHS_c9709/f2p9/1763	66.1678094	424.210227	-2.6806	7.39E-08	1.72E-06
i3_LQ_YHS_c13524/f1p3/3547	0	187.2889707	-Inf	7.43E-08	1.73E-06
i4_LQ_YHS_c10332/f1p2/4314	4.995312513	86.41421723	-4.1126	7.43E-08	1.73E-06
i2_LQ_YHS_c35206/f1p2/2349	206.3428576	2073.796577	-3.3292	7.53E-08	1.75E-06
i0_LQ_YHS_c2223/f1p9/734	733.8244823	34.43232086	4.4136	7.54E-08	1.75E-06
i2_LQ_YHS_c23422/f1p0/2195	15.79419288	526.876866	-5.06	7.54E-08	1.75E-06
i1_LQ_YHS_c28708/f1p5/1157	0	220.7492084	-Inf	7.57E-08	1.75E-06
i2_LQ_YHS_c19702/f1p18/1955	333.7576242	8795.249042	-4.7199	7.56E-08	1.75E-06
i0_LQ_YHS_c973/f1p0/629	31.20892363	235.7961713	-2.9175	7.59E-08	1.76E-06
i2_LQ_YHS_c25982/f1p4/2658	17.54273822	169.761396	-3.2746	7.67E-08	1.78E-06
i1_LQ_YHS_c21185/f1p10/1890	34.76906974	291.2077403	-3.0662	7.68E-08	1.78E-06
i2_LQ_YHS_c27020/f1p3/2174	80.53036267	1994.963505	-4.6307	7.71E-08	1.78E-06
i2_LQ_YHS_c12407/f1p3/2249	73.22500432	1824.345787	-4.6389	7.75E-08	1.79E-06
i1_HQ_YHS_c1412/f10p6/1754	0	707.724394	-Inf	7.77E-08	1.79E-06
i2_LQ_YHS_c36112/f1p2/2351	11.96123036	137.6996676	-3.5251	7.81E-08	1.80E-06
i1_HQ_YHS_c19868/f2p0/1839	3.955873204	121.1794802	-4.937	7.90E-08	1.82E-06
i1_LQ_YHS_c39657/f1p0/1261	0.874099149	52.06084622	-5.8963	7.90E-08	1.82E-06
i5_LQ_YHS_c2006/f1p0/5834	10.63054593	119.0717689	-3.4855	7.90E-08	1.82E-06
i4_LQ_YHS_c9706/f1p0/4581	16.79246648	215.9048741	-3.6845	7.99E-08	1.84E-06
i2_LQ_YHS_c53530/f1p2/2587	10.06072687	122.898603	-3.6107	8.05E-08	1.86E-06
i1_HQ_YHS_c40870/f129p0/1813	263.4459495	11118.34622	-5.3993	8.09E-08	1.86E-06
i2_HQ_YHS_c60434/f12p1/2508	38.71619113	275.0646929	-2.8288	8.10E-08	1.87E-06
i4_LQ_YHS_c13793/f1p0/4092	2.723412986	73.00435584	-4.7445	8.11E-08	1.87E-06
i3_LQ_YHS_c5820/f1p0/3861	83.91117626	510.2809618	-2.6044	8.17E-08	1.88E-06
i2_LQ_YHS_c21606/f1p8/2724	13.99688248	166.2733059	-3.5704	8.18E-08	1.88E-06
i2_LQ_YHS_c5588/f1p0/2241	58.12842753	369.8781678	-2.6697	8.20E-08	1.89E-06
i2_LQ_YHS_c5851/f1p0/2791	103.3844156	6.978392039	3.889	8.22E-08	1.89E-06
i3_LQ_YHS_c7930/f1p4/3310	71.60589194	439.2407564	-2.6169	8.25E-08	1.89E-06
i3_LQ_YHS_c6029/f1p0/3456	314.4125553	3137.184141	-3.3187	8.30E-08	1.90E-06
i1_LQ_YHS_c39177/f1p11/1223	67.34746915	420.8365957	-2.6436	8.37E-08	1.92E-06
i2_LQ_YHS_c58836/f1p4/2064	0	181.7162397	-Inf	8.45E-08	1.94E-06
i1_LQ_YHS_c8563/f2p0/1752	72.49701959	452.6287366	-2.6423	8.50E-08	1.95E-06
i1_LQ_YHS_c13483/f1p1/1170	96.65113939	584.9320854	-2.5974	8.53E-08	1.95E-06
i0_LQ_YHS_c2850/f1p0/771	236.4602355	12384.89305	-5.7108	8.58E-08	1.96E-06
i4_LQ_YHS_c9074/f1p1/4774	689.3488414	44.07312826	3.9673	8.60E-08	1.97E-06
i1_LQ_YHS_c33611/f1p0/1767	0	49.54195444	-Inf	8.67E-08	1.98E-06
i2_LQ_YHS_c7115/f1p0/2481	29.68187698	224.8599379	-2.9214	8.72E-08	1.99E-06
i4_LQ_YHS_c5561/f1p1/4543	2.995754284	74.89445187	-4.6439	8.77E-08	2.00E-06
i1_LQ_YHS_c7326/f1p3/2023	81.4435751	774.1485911	-3.2487	8.79E-08	2.01E-06
i2_LQ_YHS_c19896/f1p2/2260	67.29613468	426.6066847	-2.6643	8.88E-08	2.03E-06
i1_LQ_YHS_c34173/f1p0/1888	164.239035	1059.804738	-2.6899	9.06E-08	2.07E-06
i3_LQ_YHS_c17579/f1p3/3200	87.14508557	554.7328442	-2.6703	9.12E-08	2.08E-06
i1_LQ_YHS_c24208/f1p0/1849	367.8255188	7644.050389	-4.3772	9.15E-08	2.09E-06
i2_LQ_YHS_c64636/f1p3/2013	253.3519877	2026.33442	-2.9997	9.18E-08	2.09E-06
i2_LQ_YHS_c29491/f1p1/2065	12.26632423	337.6056084	-4.7826	9.19E-08	2.09E-06
i1_LQ_YHS_c28712/f1p0/1316	7.311252325	1057.106764	-7.1758	9.20E-08	2.09E-06
i2_LQ_YHS_c14586/f1p15/2061	375.191107	6546.724779	-4.1251	9.26E-08	2.11E-06
i4_LQ_YHS_c11412/f1p0/4751	36.14093662	268.0615349	-2.8909	9.39E-08	2.13E-06
i1_LQ_YHS_c18460/f1p0/1936	112.2136121	0	Inf	9.43E-08	2.14E-06
i2_LQ_YHS_c28301/f1p0/2053	24.14615665	199.27774	-3.0449	9.43E-08	2.14E-06
i2_LQ_YHS_c39443/f1p1/2327	27.50614923	217.3194159	-2.982	9.43E-08	2.14E-06
i2_LQ_YHS_c23599/f1p2/2138	165.2253519	1062.441913	-2.6849	9.45E-08	2.14E-06
i1_HQ_YHS_c11193/f3p0/1933	1340.996426	199.0139953	2.7524	9.47E-08	2.15E-06
i1_LQ_YHS_c10530/f1p1/1613	116.0698885	853.2901409	-2.878	9.47E-08	2.15E-06
i2_LQ_YHS_c52049/f1p4/2766	3.519455513	132.4524012	-5.234	9.65E-08	2.19E-06
i5_HQ_YHS_c5117/f3p0/5126	67.60072832	788.8003377	-3.5445	9.66E-08	2.19E-06
i1_LQ_YHS_c19500/f1p3/1734	10.62131064	164.0543766	-3.9491	9.69E-08	2.19E-06

i1_LQ_YHS_c32774/f1p0/1947	184.0839935	20.75993868	3.1485	9.75E-08	2.21E-06
i1_LQ_YHS_c35881/f1p0/1846	398.5615395	48.5739021	3.0365	9.81E-08	2.22E-06
i2_HQ_YHS_c17163/f5p1/2846	127.8732261	776.0562693	-2.6014	9.83E-08	2.22E-06
i1_HQ_YHS_c4311/f2p28/1776	0	89.51490338	-Inf	9.85E-08	2.23E-06
i2_LQ_YHS_c39670/f1p5/2649	306.2424468	2586.5675	-3.0783	1.01E-07	2.27E-06
i1_LQ_YHS_c18153/f1p2/1986	407.6534003	5.4738821	6.2186	1.01E-07	2.27E-06
i3_LQ_YHS_c4877/f1p0/3794	317.3409231	2816.263259	-3.1497	1.01E-07	2.28E-06
i1_LQ_YHS_c35386/f1p4/1837	0	36.91388963	-Inf	1.02E-07	2.29E-06
i2_LQ_YHS_c4906/f8p1/2813	117.5632252	700.2464091	-2.5744	1.02E-07	2.30E-06
i2_LQ_YHS_c5741/f1p2/2407	306.0496843	2766.333244	-3.1761	1.05E-07	2.37E-06
i3_LQ_YHS_c8377/f1p5/3915	14.57049284	151.7409294	-3.3805	1.06E-07	2.40E-06
i2_HQ_YHS_c7826/f2p0/2767	334.0747993	50.89800465	2.7145	1.07E-07	2.40E-06
i2_LQ_YHS_c29039/f1p6/2054	172.0095617	1607.601704	-3.2243	1.07E-07	2.41E-06
i1_LQ_YHS_c5839/f1p0/1760	0	55.18941541	-Inf	1.08E-07	2.43E-06
i1_LQ_YHS_c4680/f2p1/1968	85.783999	526.7719587	-2.6184	1.08E-07	2.44E-06
i1_LQ_YHS_c32716/f1p4/1956	5543.76484	0.392261031	13.787	1.10E-07	2.48E-06
i1_HQ_YHS_c781/f20p0/1602	3828.227893	367.5858138	3.3805	1.11E-07	2.49E-06
i3_LQ_YHS_c2040/f3p3/3902	155.3695536	957.9018048	-2.6242	1.12E-07	2.51E-06
i3_LQ_YHS_c12951/f1p0/3690	48.44924033	317.9534566	-2.7143	1.13E-07	2.55E-06
i1_HQ_YHS_c9653/f2p4/1745	80.38060176	502.189571	-2.6433	1.15E-07	2.58E-06
i2_LQ_YHS_c25791/f1p140/2085	13379.67811	0	Inf	1.16E-07	2.59E-06
i2_LQ_YHS_c13303/f1p1/2410	37.47738695	385.1078188	-3.3612	1.17E-07	2.61E-06
i2_LQ_YHS_c24068/f1p0/2473	0	97.21980384	-Inf	1.17E-07	2.62E-06
i1_HQ_YHS_c37918/f2p0/1397	311.0706635	47.27081454	2.7182	1.20E-07	2.69E-06
i3_LQ_YHS_c13234/f1p9/3751	44.76349444	306.7269392	-2.7766	1.20E-07	2.69E-06
i2_HQ_YHS_c61023/f7p0/2172	11.63515513	238.4039632	-4.3568	1.21E-07	2.71E-06
i2_LQ_YHS_c18035/f1p2/2313	342.4855233	3119.546099	-3.1872	1.22E-07	2.73E-06
i1_HQ_YHS_c37922/f2p2/1249	607.2140455	3.730666322	7.3466	1.24E-07	2.78E-06
i4_LQ_YHS_c9246/f1p0/4624	490.4200319	21.66574884	4.5005	1.25E-07	2.80E-06
i1_LQ_YHS_c33292/f1p1/1728	3.900061718	79.9221236	-4.357	1.26E-07	2.81E-06
i2_LQ_YHS_c37024/f1p3/2204	67.6271788	400.1872021	-2.565	1.27E-07	2.84E-06
i4_LQ_YHS_c12029/f1p0/4299	5.9705272	92.16017872	-3.9482	1.28E-07	2.85E-06
i2_LQ_YHS_c57977/f1p1/2239	2.178730389	65.58863903	-4.9119	1.29E-07	2.87E-06
i2_HQ_YHS_c8760/f9p1/2390	69.61889357	424.5275272	-2.6083	1.31E-07	2.91E-06
i3_HQ_YHS_c21295/f4p0/3192	23.22961128	201.8774737	-3.1194	1.31E-07	2.91E-06
i4_LQ_YHS_c3668/f1p0/4861	6.602493335	95.94811152	-3.8612	1.31E-07	2.92E-06
i0_HQ_YHS_c217/f4p0/618	159.0763048	16410.21871	-6.6887	1.32E-07	2.93E-06
i2_HQ_YHS_c32531/f2p2/2819	24.07859072	194.5961207	-3.0147	1.33E-07	2.96E-06
i2_HQ_YHS_c19245/f4p3/2965	414.4553437	58.13536686	2.8337	1.33E-07	2.96E-06
i2_HQ_YHS_c63633/f2p1/2598	267.7984605	38.41907256	2.8013	1.34E-07	2.97E-06
i1_HQ_YHS_c29183/f20p0/1734	83.74581935	508.1227705	-2.6011	1.34E-07	2.98E-06
i3_LQ_YHS_c7034/f1p9/3832	369.2137009	4532.232012	-3.6177	1.35E-07	2.99E-06
i2_HQ_YHS_c60841/f4p0/2103	64.30339142	394.1731909	-2.6159	1.35E-07	2.99E-06
i1_LQ_YHS_c23747/f1p2/1405	182.075699	1174.542313	-2.6895	1.36E-07	3.02E-06
i0_LQ_YHS_c2912/f1p0/625	1.903047774	91.05626219	-5.5804	1.36E-07	3.02E-06
i1_LQ_YHS_c6481/f1p1/1736	0.544682597	45.67953494	-6.39	1.37E-07	3.04E-06
i1_LQ_YHS_c33964/f1p2/1872	34.35364179	248.8627068	-2.8568	1.39E-07	3.07E-06
i1_LQ_YHS_c34606/f1p0/1890	0	488.482499	-Inf	1.39E-07	3.07E-06
i2_LQ_YHS_c8451/f1p3/2297	15.19197667	206.917747	-3.7677	1.39E-07	3.07E-06
i3_LQ_YHS_c17735/f1p2/3617	0	111.1780883	-Inf	1.39E-07	3.08E-06
i3_HQ_YHS_c1409/f4p0/3317	45.41898623	306.8515444	-2.7562	1.41E-07	3.11E-06
i2_HQ_YHS_c1741/f9p12/2206	379.5839041	6448.369759	-4.0864	1.41E-07	3.11E-06
i2_HQ_YHS_c5506/f2p1/2533	64.01087416	501.1075021	-2.9687	1.41E-07	3.12E-06
i3_LQ_YHS_c9307/f1p0/3520	123.7679139	739.1904815	-2.5783	1.41E-07	3.12E-06
i1_LQ_YHS_c25299/f1p10/1593	477.5842021	64.72686636	2.8833	1.41E-07	3.12E-06
i3_LQ_YHS_c17716/f1p3/3684	30.7629571	224.0415704	-2.8645	1.41E-07	3.12E-06
i4_LQ_YHS_c3598/f1p3/4353	18.00647287	168.680388	-3.2277	1.44E-07	3.17E-06
i6_LQ_YHS_c717/f1p0/6980	10.24785381	121.1488321	-3.5634	1.45E-07	3.19E-06
i2_HQ_YHS_c2107/f6p0/2633	25.10245077	205.7340901	-3.0349	1.46E-07	3.22E-06
i1_LQ_YHS_c19595/f1p0/1993	80.83829882	3.92320309	4.3649	1.47E-07	3.23E-06
i3_LQ_YHS_c9083/f1p3/3131	35.187839	350.0583122	-3.3144	1.47E-07	3.24E-06
i3_LQ_YHS_c3683/f1p0/3106	200.1922954	1316.092454	-2.7168	1.49E-07	3.28E-06
i2_LQ_YHS_c20252/f1p1/2757	9.577724612	235.2892959	-4.6186	1.50E-07	3.31E-06
i1_LQ_YHS_c18792/f1p0/1753	312.1963643	2709.830963	-3.1177	1.52E-07	3.35E-06
i2_LQ_YHS_c21517/f1p0/2081	43.86854199	0	Inf	1.53E-07	3.36E-06
i4_LQ_YHS_c9584/f1p0/4400	89.19787032	3.563209152	4.6458	1.55E-07	3.41E-06
i2_LQ_YHS_c49676/f1p18/2721	207.9253447	1339.874876	-2.688	1.57E-07	3.45E-06
i1_LQ_YHS_c14183/f1p0/1271	2.266013927	82.08828803	-5.1789	1.58E-07	3.47E-06
i1_LQ_YHS_c32327/f1p0/1689	174.5247048	1104.458754	-2.6618	1.58E-07	3.47E-06
i1_LQ_YHS_c4863/f1p5/1974	1.263932271	158.6701071	-6.972	1.58E-07	3.47E-06
i2_HQ_YHS_c17768/f4p0/2260	382.2792845	60.86199553	2.651	1.59E-07	3.48E-06
i1_LQ_YHS_c26304/f1p1/1858	1080.078688	0	Inf	1.59E-07	3.49E-06
i5_LQ_YHS_c2433/f1p2/5436	14.75221766	144.9242261	-3.2963	1.60E-07	3.51E-06
i3_LQ_YHS_c9390/f1p1/3379	12.13453367	141.7827405	-3.5465	1.61E-07	3.52E-06
i3_LQ_YHS_c11957/f1p1/3625	61.83213539	414.1140039	-2.7436	1.62E-07	3.55E-06
i1_LQ_YHS_c5732/f1p0/1913	552.6269465	7.485819465	6.206	1.63E-07	3.56E-06
i2_HQ_YHS_c1522/f9p2/2827	14.26794326	833.1432978	-5.8677	1.64E-07	3.58E-06
i3_HQ_YHS_c1591/f4p0/3628	198.6770534	1247.600504	-2.6507	1.64E-07	3.58E-06
i3_LQ_YHS_c2242/f2p1/3263	109.2678699	632.9894349	-2.5343	1.64E-07	3.58E-06
i3_LQ_YHS_c5916/f1p2/3467	9.630991814	116.8166614	-3.6004	1.66E-07	3.63E-06
i2_LQ_YHS_c4200/f3p1/2359	183.0276203	16.60861459	3.4621	1.67E-07	3.65E-06
i2_LQ_YHS_c23439/f1p0/2711	0.97648683	49.04680997	-5.6504	1.67E-07	3.65E-06
i1_HQ_YHS_c15063/f7p11/1822	685.6603227	118.5276903	2.5323	1.68E-07	3.67E-06
i1_HQ_YHS_c39850/f4p2/1874	205.2633153	1288.558652	-2.6502	1.69E-07	3.67E-06
i2_LQ_YHS_c3086/f1p4/2332	105.314541	610.7043699	-2.5358	1.69E-07	3.67E-06
i1_LQ_YHS_c21691/f1p0/2057	13.66238573	136.6694857	-3.3224	1.69E-07	3.68E-06
i1_HQ_YHS_c16996/f3p0/1664	0	284.721881	-Inf	1.71E-07	3.71E-06
i1_LQ_YHS_c28723/f1p0/1247	135.3305916	826.3150967	-2.6102	1.71E-07	3.71E-06
i1_LQ_YHS_c3196/f1p0/1904	4.14680819	78.11382041	-4.2355	1.71E-07	3.71E-06

i2_LQ_YHS_c25526/f1p1/2195	412.2023682	62.00833054	2.7328	1.71E-07	3.72E-06
i2_LQ_YHS_c58594/f1p0/2316	376.8987332	7820.806625	-4.3751	1.72E-07	3.73E-06
i0_LQ_YHS_c556/f1p0/756	30299.33517	16.86959545	10.811	1.72E-07	3.74E-06
i3_LQ_YHS_c10875/f1p23/3241	167.1270952	19.37612406	3.1086	1.72E-07	3.74E-06
i3_LQ_YHS_c13337/f1p0/3340	136.2914752	808.9565097	-2.5694	1.72E-07	3.74E-06
i1_HQ_YHS_c25465/f2p0/1648	194.2200867	15236.80857	-6.2937	1.73E-07	3.75E-06
i1_LQ_YHS_c3167/f1p0/1765	2.538355225	66.4405394	-4.7101	1.73E-07	3.75E-06
i3_HQ_YHS_c15517/f3p0/3510	432.8601298	19.26881815	4.4896	1.73E-07	3.75E-06
i0_LQ_YHS_c1802/f1p0/401	0.272341299	61.866226	-7.8276	1.74E-07	3.76E-06
i2_LQ_YHS_c52207/f1p4/2264	37.58820452	253.5651365	-2.754	1.74E-07	3.77E-06
i2_LQ_YHS_c43617/f1p0/2080	11.95154508	1824.237889	-7.254	1.75E-07	3.79E-06
i2_LQ_YHS_c25038/f1p1/2280	37.4412847	268.4377229	-2.8419	1.76E-07	3.81E-06
i2_LQ_YHS_c9766/f1p6/2233	46.89693752	346.5919081	-2.8857	1.79E-07	3.87E-06
i2_HQ_YHS_c2811/f2p0/2126	42.68094421	287.0449645	-2.7496	1.79E-07	3.88E-06
i2_LQ_YHS_c40565/f1p0/2252	5.795960124	362.2969722	-5.966	1.80E-07	3.88E-06
i1_LQ_YHS_c28399/f1p0/1281	83.16170992	488.8877247	-2.5555	1.81E-07	3.90E-06
i2_LQ_YHS_c36428/f1p0/2472	49.98250288	319.2064256	-2.675	1.82E-07	3.93E-06
i1_LQ_YHS_c9462/f1p4/1580	43.9075272	285.0630022	-2.6987	1.84E-07	3.96E-06
i1_HQ_YHS_c40267/f2p2/1313	190.5464686	1140.277499	-2.5812	1.84E-07	3.97E-06
i3_LQ_YHS_c7466/f1p0/3079	36.42584615	253.9819635	-2.8017	1.88E-07	4.05E-06
i2_HQ_YHS_c60824/f4p0/2076	49.92589436	322.2635462	-2.6904	1.89E-07	4.08E-06
i2_LQ_YHS_c58325/f1p1/2052	149.0870883	901.104294	-2.5955	1.91E-07	4.11E-06
i1_LQ_YHS_c44018/f1p0/1068	179.0505514	1029.061446	-2.5229	1.93E-07	4.15E-06
i1_LQ_YHS_c25523/f1p4/1893	21.2095468	178.2394816	-3.071	1.94E-07	4.17E-06
i1_LQ_YHS_c33220/f1p119/1829	5.251277527	85.29375571	-4.0217	1.95E-07	4.19E-06
i2_LQ_YHS_c10444/f1p1/2531	293.9375095	44.72198723	2.7165	1.97E-07	4.22E-06
i1_HQ_YHS_c1542/f15p0/1849	403.5634245	5717.684379	-3.8246	1.98E-07	4.25E-06
i1_HQ_YHS_c31610/f2p0/1921	479.9092528	74.42707181	2.6889	2.00E-07	4.30E-06
i2_LQ_YHS_c50454/f1p2/2369	67.50264892	404.9682668	-2.5848	2.01E-07	4.30E-06
i2_LQ_YHS_c55808/f1p4/2762	63.26025537	2.212254406	4.8377	2.01E-07	4.31E-06
i5_LQ_YHS_c3066/f1p0/5434	116.7545366	844.5411999	-2.8547	2.03E-07	4.35E-06
i0_LQ_YHS_c1808/f1p2/704	161.0510652	17103.79363	-6.7307	2.04E-07	4.37E-06
i2_HQ_YHS_c9944/f6p0/2356	11.65995292	127.3047863	-3.4487	2.05E-07	4.39E-06
i1_HQ_YHS_c17198/f2p0/1470	119.3880141	690.6503841	-2.5323	2.05E-07	4.40E-06
i3_LQ_YHS_c5165/f1p0/3273	10.36534563	118.2044266	-3.5114	2.07E-07	4.44E-06
i3_LQ_YHS_c20303/f1p10/3046	40.1600926	274.8897285	-2.775	2.08E-07	4.44E-06
i1_LQ_YHS_c27679/f1p0/1134	203.3187966	1215.042517	-2.5792	2.09E-07	4.48E-06
i2_LQ_YHS_c23098/f1p6/2436	0	36.25622414	-Inf	2.10E-07	4.48E-06
i1_LQ_YHS_c31602/f2p0/1793	353.798926	5.819381284	5.9259	2.12E-07	4.53E-06
i1_LQ_YHS_c14596/f1p2/1316	378.7106259	9137.252079	-4.5926	2.13E-07	4.56E-06
i3_LQ_YHS_c9715/f1p0/3174	97.99867521	555.9956475	-2.5042	2.14E-07	4.56E-06
i2_HQ_YHS_c60820/f3p2/2102	74.12821792	3.530349282	4.3921	2.15E-07	4.58E-06
i5_HQ_YHS_c364/f6p0/5237	288.2140017	3.935446501	6.1945	2.16E-07	4.60E-06
i3_LQ_YHS_c3528/f1p10/3202	17.75385754	155.6338772	-3.132	2.19E-07	4.66E-06
i2_LQ_YHS_c41502/f1p1/2626	24.54393618	197.1622485	-3.0059	2.20E-07	4.68E-06
i4_LQ_YHS_c8195/f1p1/4485	7.256712981	98.44141114	-3.7619	2.21E-07	4.70E-06
i2_LQ_YHS_c53076/f1p0/2431	35.252072	245.0060091	-2.797	2.21E-07	4.71E-06
i2_LQ_YHS_c36449/f1p1/2386	206.2135874	1287.5043	-2.6424	2.22E-07	4.72E-06
i4_LQ_YHS_c10252/f1p6/4998	56.6269422	634.5878723	-3.4863	2.23E-07	4.74E-06
i3_HQ_YHS_c21165/f10p0/3807	323.6986734	2604.98738	-3.0086	2.24E-07	4.76E-06
i6_LQ_YHS_c395/f1p0/6170	7.988989248	106.492637	-3.7366	2.24E-07	4.76E-06
i3_LQ_YHS_c7699/f1p4/3660	28.14559503	354.918218	-3.6565	2.25E-07	4.79E-06
i1_LQ_YHS_c6575/f1p7/1517	175.2741388	1046.017614	-2.5772	2.26E-07	4.79E-06
i2_LQ_YHS_c50180/f1p3/2267	248.720819	1704.108472	-2.7764	2.26E-07	4.79E-06
i0_LQ_YHS_c2646/f1p2/995	2.168239704	60.4285818	-4.8006	2.28E-07	4.83E-06
i1_LQ_YHS_c17371/f1p0/1753	40.98379913	531.9376224	-3.6981	2.28E-07	4.84E-06
i2_LQ_YHS_c54343/f1p0/2116	32.5475712	233.2023544	-2.841	2.29E-07	4.84E-06
i2_LQ_YHS_c32724/f1p2/2126	31.47077424	223.8826791	-2.8307	2.29E-07	4.85E-06
i2_HQ_YHS_c4136/f3p8/2737	47.35892492	685.6827808	-3.8558	2.29E-07	4.86E-06
i3_LQ_YHS_c19287/f1p0/3237	37.47579289	247.5105259	-2.7235	2.29E-07	4.86E-06
i1_HQ_YHS_c7085/f2p1/1921	16.60232853	952.3583194	-5.842	2.32E-07	4.91E-06
i3_LQ_YHS_c9680/f1p2/3977	79.00519971	467.1608797	-2.5639	2.33E-07	4.93E-06
i2_LQ_YHS_c10325/f1p0/2437	3.268095583	72.46079096	-4.4707	2.35E-07	4.98E-06
i1_HQ_YHS_c29319/f5p0/1565	329.050632	2710.989155	-3.0424	2.36E-07	4.99E-06
i0_HQ_YHS_c415/f2p0/754	423.1475652	5863.347141	-3.7925	2.42E-07	5.11E-06
i2_LQ_YHS_c24317/f1p2/2706	2.163626246	62.71540201	-4.8573	2.42E-07	5.11E-06
i2_LQ_YHS_c18154/f1p3/2499	189.3025556	1.961305157	6.5927	2.43E-07	5.12E-06
i5_LQ_YHS_c3043/f1p1/5090	54.74444256	331.4804171	-2.5981	2.44E-07	5.14E-06
i2_HQ_YHS_c10469/f8p4/2851	332.2598548	10463.52629	-4.9769	2.44E-07	5.15E-06
i4_LQ_YHS_c7098/f1p0/4939	5.991508569	92.2870353	-3.9451	2.45E-07	5.16E-06
i2_LQ_YHS_c49330/f1p1/2991	27.74655174	210.5172887	-2.9236	2.46E-07	5.18E-06
i2_LQ_YHS_c52977/f1p0/2086	28.35846998	203.4389364	-2.8427	2.46E-07	5.18E-06
i2_HQ_YHS_c43186/f2p1/2065	599.0861319	41.86138641	3.8391	2.48E-07	5.22E-06
i5_LQ_YHS_c3398/f1p3/5323	187.576235	23.94087886	2.9699	2.53E-07	5.33E-06
i0_HQ_YHS_c262/f4p0/888	288.6344954	12356.01267	-5.4198	2.55E-07	5.37E-06
i1_LQ_YHS_c21438/f1p0/1976	48.75372265	309.212019	-2.665	2.56E-07	5.37E-06
i2_LQ_YHS_c44854/f1p1/2074	2.723412986	67.04650689	-4.6217	2.58E-07	5.43E-06
i2_LQ_YHS_c7739/f1p0/2174	1.418781746	53.9209265	-5.2481	2.59E-07	5.43E-06
i1_LQ_YHS_c36616/f1p0/1457	177.5572399	1056.745552	-2.5733	2.59E-07	5.43E-06
i2_LQ_YHS_c4898/f1p8/2281	12.05232195	130.7300821	-3.4392	2.59E-07	5.44E-06
i2_LQ_YHS_c10239/f1p3/2211	143.2603096	786.7320008	-2.4572	2.59E-07	5.44E-06
i1_LQ_YHS_c6371/f1p1/1507	13.59608356	306.1018891	-4.4927	2.63E-07	5.52E-06
i3_LQ_YHS_c17238/f1p49/3568	18.34050288	160.5745062	-3.1301	2.64E-07	5.53E-06
i3_LQ_YHS_c19223/f1p6/3582	169.7501646	1013.561768	-2.5779	2.66E-07	5.57E-06
i1_LQ_YHS_c5231/f1p0/1926	10.43291157	118.7556915	-3.5088	2.68E-07	5.61E-06
i1_HQ_YHS_c27023/f3p1/1156	240.5327616	14079.21394	-5.8712	2.71E-07	5.67E-06
i2_LQ_YHS_c44169/f1p12/1718	409.2483086	4502.637572	-3.4597	2.72E-07	5.68E-06
i1_HQ_YHS_c18752/f2p0/1847	34.74809674	233.1360084	-2.7462	2.72E-07	5.69E-06
i2_LQ_YHS_c34690/f1p3/2703	40.59029439	309.8054234	-2.9322	2.75E-07	5.75E-06

i1_LQ_YHS_c36174/f1p0/1826	96.94747308	540.863067	-2.48	2.77E-07	5.78E-06
i2_LQ_YHS_c6242/f1p1/2858	306.0467284	50.1869035	2.6084	2.79E-07	5.82E-06
i0_LQ_YHS_c3144/f1p13/976	307.9890617	11848.8521	-5.2657	2.79E-07	5.83E-06
i3_LQ_YHS_c14371/f1p0/3055	412.5955582	5061.489154	-3.6168	2.79E-07	5.83E-06
i1_LQ_YHS_c3870/f1p0/1709	171.0620816	1006.063153	-2.5561	2.82E-07	5.89E-06
i2_LQ_YHS_c23241/f1p0/2165	9.21222255	119.6554196	-3.6992	2.83E-07	5.90E-06
i7_LQ_YHS_c215/f1p0/7871	0.272341299	39.30197425	-7.173	2.83E-07	5.90E-06
i4_LQ_YHS_c6363/f1p0/4445	277.3376834	43.64026663	2.6679	2.85E-07	5.94E-06
i1_HQ_YHS_c20076/f2p0/1657	194.6337351	1210.836605	-2.6372	2.86E-07	5.95E-06
i1_HQ_YHS_c10911/f5p0/1710	24.01563824	308.1455046	-3.6816	2.88E-07	5.98E-06
i0_HQ_YHS_c3686/f3p0/941	416.8210908	6009.922022	-3.8498	2.88E-07	6.00E-06
i1_LQ_YHS_c24999/f1p0/1854	157.5664159	18.30672709	3.1055	2.89E-07	6.01E-06
i2_LQ_YHS_c24022/f1p3/2221	48.36529811	540.4625049	-3.4822	2.89E-07	6.01E-06
i1_LQ_YHS_c20767/f1p2/1505	13.82438457	133.9393784	-3.2763	2.90E-07	6.02E-06
i1_LQ_YHS_c12510/f1p0/1718	167.1035779	940.0110438	-2.4919	2.90E-07	6.02E-06
i3_LQ_YHS_c17531/f1p3/3148	64.08594488	520.4939989	-3.0218	2.90E-07	6.03E-06
i1_LQ_YHS_c11363/f2p0/1879	1.361706493	83.36858688	-5.936	2.93E-07	6.08E-06
i1_LQ_YHS_c20971/f1p0/1775	49.46169298	297.6663072	-2.5893	2.93E-07	6.08E-06
i1_HQ_YHS_c28714/f2p0/1701	61.99445616	371.5955497	-2.5835	2.94E-07	6.09E-06
i0_LQ_YHS_c1479/f1p0/842	256.8175645	2570.724759	-3.3234	2.97E-07	6.16E-06
i1_LQ_YHS_c28745/f1p0/1328	47.81794316	495.0247172	-3.3719	2.98E-07	6.17E-06
i2_HQ_YHS_c6482/f4p1/2944	105.2485942	814.2754945	-2.9517	2.98E-07	6.18E-06
i1_LQ_YHS_c4134/f1p0/1742	12.90624514	341.8933416	-4.7274	2.99E-07	6.18E-06
i2_LQ_YHS_c45076/f1p8/2025	232.7961783	1467.812044	-2.6565	3.00E-07	6.21E-06
i2_HQ_YHS_c60915/f4p2/2076	174.9479104	1016.936632	-2.5392	3.01E-07	6.21E-06
i2_LQ_YHS_c52145/f1p0/2177	47.76942586	0.784522063	5.9281	3.01E-07	6.21E-06
i2_HQ_YHS_c19996/f3p5/2404	59.80063838	350.9134962	-2.5529	3.01E-07	6.22E-06
i4_HQ_YHS_c1764/f5p0/4524	254.1314278	1664.542184	-2.7115	3.01E-07	6.22E-06
i1_LQ_YHS_c8951/f1p289/1928	48.4118743	310.3324026	-2.6804	3.04E-07	6.28E-06
i2_LQ_YHS_c20573/f1p3/2373	2.178730389	61.95196942	-4.8296	3.05E-07	6.29E-06
i1_LQ_YHS_c28126/f1p0/1323	0	71.10359474	-Inf	3.07E-07	6.32E-06
i2_HQ_YHS_c60616/f40p1/2360	211.7763554	5078.506954	-4.5838	3.07E-07	6.33E-06
i1_HQ_YHS_c1929/f4p1/1868	150.1120518	848.2891069	-2.4985	3.07E-07	6.33E-06
i7_LQ_YHS_c49/f2p0/7736	880.4641561	66.35033284	3.7301	3.07E-07	6.33E-06
i1_LQ_YHS_c21513/f1p4/1689	86.44967629	0	Inf	3.09E-07	6.37E-06
i2_LQ_YHS_c18849/f1p12/2259	280.4973058	12912.31465	-5.5246	3.09E-07	6.37E-06
i3_LQ_YHS_c3649/f1p0/3952	1.573631222	56.23890847	-5.1594	3.09E-07	6.37E-06
i1_LQ_YHS_c35754/f1p0/1937	233.4081552	33.59444223	2.7966	3.11E-07	6.40E-06
i2_LQ_YHS_c51960/f1p1/2290	55.75207115	333.2626551	-2.5796	3.13E-07	6.44E-06
i4_LQ_YHS_c7033/f1p21/4863	142.6085923	809.5743674	-2.5051	3.14E-07	6.45E-06
i2_LQ_YHS_c7791/f1p3/2157	11.71067584	247.7310617	-4.4029	3.14E-07	6.45E-06
i4_LQ_YHS_c9350/f1p3/4395	0	33.20774968	-Inf	3.17E-07	6.51E-06
i1_LQ_YHS_c6894/f1p0/1807	1510.32624	243.149847	2.6349	3.17E-07	6.51E-06
i2_HQ_YHS_c60719/f13p1/2431	1909.838972	277.6808601	2.782	3.21E-07	6.58E-06
i0_LQ_YHS_c1705/f1p0/662	28.3155654	239.6120228	-3.081	3.22E-07	6.61E-06
i4_LQ_YHS_c10784/f1p3/4369	20.54944155	171.0441055	-3.0572	3.25E-07	6.65E-06
i1_LQ_YHS_c18580/f1p4/1762	8.693940186	120.8522263	-3.7971	3.25E-07	6.66E-06
i2_LQ_YHS_c23788/f1p6/2283	89.46430927	6.058679899	3.8842	3.28E-07	6.72E-06
i1_LQ_YHS_c43847/f1p0/1030	0	32.98188002	-Inf	3.29E-07	6.73E-06
i0_LQ_YHS_c3037/f1p0/972	395.4371325	3546.171273	-3.1647	3.29E-07	6.73E-06
i2_LQ_YHS_c6622/f1p3/2466	71.69808573	657.9112969	-3.1979	3.30E-07	6.74E-06
i2_LQ_YHS_c50493/f1p7/2531	5816.96859	81.76944792	6.1526	3.32E-07	6.78E-06
i2_HQ_YHS_c55668/f2p4/2535	50.02192133	319.7686298	-2.6764	3.34E-07	6.82E-06
i2_LQ_YHS_c7518/f1p9/3070	104.901199	1364.15237	-3.7009	3.35E-07	6.85E-06
i5_LQ_YHS_c3665/f1p2/5319	34.58447871	583.7406444	-4.0771	3.37E-07	6.88E-06
i2_LQ_YHS_c41499/f1p5/2700	2.995754284	169.7852554	-5.8246	3.40E-07	6.94E-06
i1_LQ_YHS_c38920/f1p0/1232	2500.169403	0	Inf	3.41E-07	6.95E-06
i1_HQ_YHS_c15265/f2p0/1338	166.9703872	921.27576	-2.464	3.43E-07	7.00E-06
i2_LQ_YHS_c22943/f1p61/2765	645.9110015	6.161010107	6.712	3.44E-07	7.00E-06
i1_LQ_YHS_c22925/f1p0/1474	60.31682645	895.3118201	-3.8918	3.45E-07	7.03E-06
i2_HQ_YHS_c10203/f2p0/2098	150.3401673	831.9539071	-2.4683	3.45E-07	7.03E-06
i2_LQ_YHS_c38438/f1p0/2377	13.40434317	130.858044	-3.2872	3.47E-07	7.07E-06
i0_LQ_YHS_c371/f2p0/894	0	32.97350698	-Inf	3.54E-07	7.19E-06
i4_LQ_YHS_c5446/f1p2/4351	52.01718803	2524.671426	-5.601	3.54E-07	7.19E-06
i1_LQ_YHS_c9718/f1p0/1768	376.7439914	3455.816537	-3.1974	3.57E-07	7.26E-06
i2_HQ_YHS_c9308/f2p11/2431	34.62598309	239.0629686	-2.7875	3.57E-07	7.26E-06
i3_LQ_YHS_c3996/f1p0/3385	16.94651055	156.0908724	-3.2033	3.58E-07	7.28E-06
i2_LQ_YHS_c19368/f1p1/2117	26.0495431	223.2850847	-3.0996	3.64E-07	7.39E-06
i2_LQ_YHS_c33288/f1p1/2455	91.71334374	520.7287663	-2.5053	3.65E-07	7.40E-06
i1_LQ_YHS_c9704/f1p2/1456	123.3591853	675.0656727	-2.4522	3.65E-07	7.40E-06
i1_LQ_YHS_c33913/f1p0/1946	97.05799264	21860.1923	-7.8152	3.66E-07	7.43E-06
i1_HQ_YHS_c5664/f2p0/1885	253.8011532	38.45572258	2.7224	3.69E-07	7.47E-06
i1_HQ_YHS_c2943/f6p4/1962	340.2732924	2583.6543	-2.9246	3.70E-07	7.48E-06
i1_LQ_YHS_c6176/f1p0/1755	11.40144362	124.0847854	-3.444	3.70E-07	7.48E-06
i1_LQ_YHS_c9981/f1p4/1590	26.04031618	193.7714315	-2.8955	3.69E-07	7.48E-06
i2_HQ_YHS_c10028/f2p6/2382	96.77751946	840.0882312	-3.1178	3.72E-07	7.53E-06
i3_LQ_YHS_c16807/f1p0/3430	148.4965942	16.67705869	3.1545	3.72E-07	7.53E-06
i4_LQ_YHS_c13813/f1p0/4115	27.46450842	243.5469161	-3.1486	3.75E-07	7.58E-06
i1_LQ_YHS_c17480/f1p5/1547	14.26079389	137.7997058	-3.2724	3.77E-07	7.61E-06
i1_LQ_YHS_c19953/f1p0/1677	126.5218331	0	Inf	3.78E-07	7.65E-06
i2_LQ_YHS_c54681/f1p23/2945	270.6851911	1642.385329	-2.6011	3.79E-07	7.65E-06
i2_HQ_YHS_c60626/f73p9/2156	412.7579233	4567.654704	-3.4681	3.80E-07	7.67E-06
i2_LQ_YHS_c39860/f1p3/2264	119.7878293	8.255373762	3.859	3.80E-07	7.67E-06
i2_LQ_YHS_c36591/f1p2/2225	8.498391741	100.141775	-3.5587	3.80E-07	7.67E-06
i5_LQ_YHS_c2405/f1p6/6065	10.16057027	115.1768823	-3.5028	3.83E-07	7.72E-06
i2_HQ_YHS_c60123/f3p0/2294	59.15956502	354.0000051	-2.5811	3.83E-07	7.73E-06
i1_LQ_YHS_c8890/f1p0/1411	1303.276892	0	Inf	3.84E-07	7.74E-06
i2_LQ_YHS_c18216/f1p3/2489	0	44.32680186	-Inf	3.85E-07	7.75E-06
i1_HQ_YHS_c6120/f2p3/1756	22.87666908	2654.036898	-6.8582	3.87E-07	7.78E-06

i1_LQ_YHS_c20067/f1p0/1457	102.5466042	6.99063545	3.8747	3.88E-07	7.81E-06
i2_LQ_YHS_c27387/f1p3/2143	39.44051263	258.9991306	-2.7152	3.88E-07	7.81E-06
i2_LQ_YHS_c11132/f1p10/2659	412.5163372	4269.070056	-3.3714	3.92E-07	7.87E-06
i3_LQ_YHS_c14969/f1p6/3041	979.1175358	174.2340522	2.4905	3.92E-07	7.87E-06
i2_LQ_YHS_c35651/f1p0/2198	68.78042157	386.9594101	-2.4921	3.93E-07	7.88E-06
i2_LQ_YHS_c54191/f1p0/2427	0	35.56459345	-Inf	3.94E-07	7.91E-06
i2_LQ_YHS_c64443/f1p0/2017	6.485001513	89.40708379	-3.7852	3.99E-07	8.01E-06
i2_LQ_YHS_c37640/f1p4/2862	35.26810961	434.5089361	-3.6229	4.01E-07	8.04E-06
i2_LQ_YHS_c53032/f1p13/2140	1.089365194	57.28038172	-5.7165	4.03E-07	8.09E-06
i3_HQ_YHS_c11398/f2p0/3875	0	72.25423245	-Inf	4.04E-07	8.09E-06
i3_LQ_YHS_c18355/f1p15/3326	10.89411868	120.0994588	-3.4626	4.09E-07	8.20E-06
i2_LQ_YHS_c28342/f1p1/2035	5.991508569	89.40096209	-3.8993	4.10E-07	8.20E-06
i4_LQ_YHS_c13065/f1p0/4246	15.13743732	138.1379769	-3.1899	4.10E-07	8.20E-06
i2_LQ_YHS_c37489/f1p6/2768	0	82.07525298	-Inf	4.22E-07	8.45E-06
i3_LQ_YHS_c18341/f1p2/3224	50.32922919	1.079981815	5.5423	4.26E-07	8.53E-06
i3_LQ_YHS_c3671/f1p1/3521	84.17635981	467.7482832	-2.4742	4.28E-07	8.55E-06
i4_HQ_YHS_c14263/f23p0/4827	409.8692325	0	Inf	4.28E-07	8.55E-06
i4_LQ_YHS_c6612/f1p2/4465	187.27791	1094.749304	-2.5473	4.28E-07	8.55E-06
i2_LQ_YHS_c5479/f1p0/2712	41.62847831	258.912913	-2.6368	4.29E-07	8.57E-06
i2_LQ_YHS_c43139/f2p2/2070	38.84498742	243.1693092	-2.6462	4.37E-07	8.73E-06
i1_LQ_YHS_c35664/f1p0/1717	416.8995208	4045.426847	-3.2785	4.42E-07	8.82E-06
i2_LQ_YHS_c39850/f2p2/2519	53.42341829	326.9161771	-2.6134	4.51E-07	9.00E-06
i2_LQ_YHS_c23305/f1p6/2854	297.5846125	7.627724025	5.2859	4.51E-07	9.00E-06
i2_LQ_YHS_c58093/f1p2/2153	0	191.2798778	-Inf	4.53E-07	9.02E-06
i2_LQ_YHS_c34431/f1p1/2311	31.94709387	300.9564909	-3.2358	4.55E-07	9.07E-06
i2_LQ_YHS_c26364/f1p2/2227	48.08993742	337.7396475	-2.8121	4.61E-07	9.18E-06
i1_LQ_YHS_c14611/f1p0/1274	210.7215905	1201.008756	-2.5108	4.65E-07	9.25E-06
i2_LQ_YHS_c64684/f1p9/1973	92.26312328	506.6549584	-2.4572	4.66E-07	9.27E-06
i6_LQ_YHS_c996/f1p0/6113	392.4152664	18.8236329	4.3818	4.68E-07	9.30E-06
i8_LQ_YHS_c127/f1p0/8175	1.90638909	57.0562904	-4.9035	4.69E-07	9.32E-06
i2_LQ_YHS_c36257/f1p2/2733	325.3366943	2326.545637	-2.8382	4.71E-07	9.37E-06
i2_HQ_YHS_c57542/f3p57/2055	2534.269086	0	Inf	4.73E-07	9.39E-06
i1_HQ_YHS_c29276/f13p5/1586	192.7006562	17165.16902	-6.477	4.73E-07	9.40E-06
i1_LQ_YHS_c38589/f1p0/1331	14831.897	3.267749399	12.148	4.76E-07	9.44E-06
i1_HQ_YHS_c29456/f7p0/1400	441.0851128	5601.997455	-3.6668	4.84E-07	9.60E-06
i2_LQ_YHS_c26058/f1p0/2128	0	54.14616383	-Inf	4.89E-07	9.70E-06
i0_LQ_YHS_c2907/f1p10/1008	36.89327753	689.2641291	-4.2236	4.90E-07	9.70E-06
i1_LQ_YHS_c42092/f1p0/1524	171.5221494	912.0465259	-2.4107	4.92E-07	9.74E-06
i2_LQ_YHS_c39962/f1p1/2347	26.49230475	193.6889261	-2.8701	4.92E-07	9.75E-06
i3_LQ_YHS_c5260/f1p0/3331	14.27717017	158.7617806	-3.4751	4.97E-07	9.83E-06
i2_LQ_YHS_c4020/f1p3/2352	451.525486	4.84291523	6.5428	5.02E-07	9.94E-06
i2_HQ_YHS_c32548/f2p4/2580	23.89895182	173.5525729	-2.8604	5.04E-07	9.97E-06
i1_LQ_YHS_c41817/f1p0/1210	12.24741204	154.0015635	-3.6524	5.05E-07	9.99E-06
i3_HQ_YHS_c2321/f2p0/3380	251.8448993	1576.802789	-2.6464	5.05E-07	9.99E-06
i1_LQ_YHS_c4458/f1p0/1835	15.1533385	591.7946897	-5.2874	5.06E-07	1.00E-05
i1_LQ_YHS_c3682/f1p5/1400	2838.059458	292.9745365	3.2761	5.07E-07	1.00E-05
i1_LQ_YHS_c22134/f1p2/1992	1897.921729	304.1039268	2.6418	5.10E-07	1.01E-05
i2_LQ_YHS_c18494/f1p3/2372	7.300761641	88.03385402	-3.5919	5.18E-07	1.02E-05
i2_LQ_YHS_c50307/f1p0/2096	96.72806868	513.8758046	-2.4094	5.20E-07	1.02E-05
i1_LQ_YHS_c35427/f1p0/1978	70.41966087	3.53872233	4.3147	5.20E-07	1.03E-05
i2_LQ_YHS_c12588/f1p4/2675	49.40520415	1.100005497	5.4891	5.21E-07	1.03E-05
i1_LQ_YHS_c9682/f1p4/1834	63.6927537	353.3649283	-2.472	5.24E-07	1.03E-05
i2_LQ_YHS_c56175/f1p0/2874	80.38974614	623.6726076	-2.9557	5.34E-07	1.05E-05
i1_LQ_YHS_c36010/f1p66/1838	0	32.18001909	-Inf	5.36E-07	1.05E-05
i2_LQ_YHS_c24419/f1p2/2328	19.63672098	248.3610479	-3.6608	5.37E-07	1.06E-05
i3_LQ_YHS_c8416/f1p0/3255	148.1311077	825.8167922	-2.4789	5.37E-07	1.06E-05
i1_LQ_YHS_c41006/f1p29/1944	18318.63887	0	Inf	5.37E-07	1.06E-05
i2_LQ_YHS_c8503/f1p1/2373	7.353215061	109.596759	-3.8977	5.38E-07	1.06E-05
i3_LQ_YHS_c17717/f1p3/3317	71.13036934	394.3935711	-2.4711	5.39E-07	1.06E-05
i3_LQ_YHS_c2885/f1p6/3725	115.7523879	625.6928746	-2.4344	5.39E-07	1.06E-05
i4_LQ_YHS_c9366/f1p0/4846	5.717098096	84.01057206	-3.8772	5.39E-07	1.06E-05
i2_HQ_YHS_c60277/f3p4/2083	98.44335285	543.548192	-2.465	5.45E-07	1.07E-05
i1_LQ_YHS_c20966/f1p0/1748	49.83640521	299.4444748	-2.587	5.47E-07	1.07E-05
i1_LQ_YHS_c33513/f1p0/1898	132.4163717	753.4539809	-2.5084	5.52E-07	1.08E-05
i3_HQ_YHS_c2655/f2p2/3944	455.2057244	5040.40596	-3.4689	5.55E-07	1.09E-05
i4_LQ_YHS_c8357/f1p1/4808	3.889571034	70.76978574	-4.1855	5.58E-07	1.09E-05
i3_LQ_YHS_c11212/f1p0/3299	102.4418087	552.038872	-2.43	5.60E-07	1.10E-05
i5_HQ_YHS_c417/f3p0/5438	598.9390805	17.1611453	5.1252	5.61E-07	1.10E-05
i1_LQ_YHS_c31934/f1p0/1915	1.089365194	47.74767299	-5.4539	5.63E-07	1.10E-05
i1_LQ_YHS_c9440/f1p2/1780	1.336111666	48.09538397	-5.1698	5.62E-07	1.10E-05
i6_LQ_YHS_c271/f1p0/6759	11.2683893	111.9286034	-3.3122	5.63E-07	1.10E-05
i3_HQ_YHS_c21587/f215p4/3665	445.6032057	8151.549389	-4.1932	5.66E-07	1.11E-05
i4_LQ_YHS_c12737/f1p17/4521	128.0834203	684.7022493	-2.4184	5.67E-07	1.11E-05
i2_LQ_YHS_c49907/f1p0/2479	39.30952747	248.2761307	-2.659	5.68E-07	1.11E-05
i4_LQ_YHS_c13431/f1p0/4117	1.089365194	55.21947071	-5.6636	5.70E-07	1.11E-05
i2_LQ_YHS_c20522/f1p1/2833	10.75771466	117.094823	-3.4442	5.74E-07	1.12E-05
i3_LQ_YHS_c9290/f1p0/3480	11.79127674	301.2801843	-4.6753	5.74E-07	1.12E-05
i1_LQ_YHS_c5026/f1p0/1621	73.15409707	418.3002068	-2.5155	5.75E-07	1.12E-05
i5_HQ_YHS_c637/f4p0/5144	450.1393335	5720.540489	-3.6677	5.76E-07	1.12E-05
i3_LQ_YHS_c19236/f1p72/3430	341.7087807	2410.79032	-2.8187	5.76E-07	1.12E-05
i2_LQ_YHS_c24308/f1p0/2419	10.43418371	111.8635566	-3.4224	5.79E-07	1.13E-05
i2_LQ_YHS_c64878/f1p19/2023	133.6310566	14.7835653	3.1762	5.79E-07	1.13E-05
i2_LQ_YHS_c15150/f1p0/2080	42.77030529	263.0062592	-2.6204	5.80E-07	1.13E-05
i1_LQ_YHS_c13718/f1p0/1283	48.23350755	289.4107345	-2.585	5.80E-07	1.13E-05
i2_HQ_YHS_c20850/f2p0/2152	11.34771806	113.0374945	-3.3163	5.81E-07	1.13E-05
i3_LQ_YHS_c4546/f1p1/3571	33.38605995	216.845362	-2.6993	5.85E-07	1.14E-05
i1_HQ_YHS_c2661/f2p3/1573	99.85405176	1432.562481	-3.8426	5.85E-07	1.14E-05
i2_LQ_YHS_c50939/f1p2/2281	42.32210803	327.925775	-2.9539	5.87E-07	1.14E-05
i2_LQ_YHS_c19316/f1p6/2643	6.155584961	84.67665015	-3.782	5.92E-07	1.15E-05

i1_LQ_YHS_c35992/f1p2/2000	2.723412986	147.0110478	-5.7544	6.01E-07	1.17E-05
i1_LQ_YHS_c8689/f1p0/1517	274.2770067	44.34809131	2.6287	6.02E-07	1.17E-05
i1_LQ_YHS_c26381/f1p0/1848	244.8247436	1434.3888	-2.5506	6.04E-07	1.17E-05
i3_LQ_YHS_c10013/f1p0/3330	26.5821242	193.5459151	-2.8641	6.06E-07	1.17E-05
i2_LQ_YHS_c9522/f1p18/2163	23.31309514	198.0745336	-3.0868	6.07E-07	1.18E-05
i2_LQ_YHS_c27194/f1p0/2513	0.544682597	40.48499882	-6.2158	6.08E-07	1.18E-05
i1_LQ_YHS_c32590/f1p1/1617	40.1950424	259.7318744	-2.6919	6.13E-07	1.19E-05
i2_LQ_YHS_c52781/f1p0/2576	473.1655721	88.3082243	2.4217	6.13E-07	1.19E-05
i1_LQ_YHS_c9174/f1p3/1764	65.50197174	360.0372362	-2.4585	6.14E-07	1.19E-05
i0_HQ_YHS_c355/f2p0/514	94.47380921	1732.240815	-4.1966	6.16E-07	1.19E-05
i1_LQ_YHS_c3140/f1p0/1585	42.81593965	269.6133193	-2.6547	6.22E-07	1.20E-05
i1_LQ_YHS_c4860/f2p3/1667	103.3963975	535.4108672	-2.3725	6.23E-07	1.20E-05
i1_HQ_YHS_c2543/f3p0/1729	0	207.0678197	-Inf	6.29E-07	1.21E-05
i1_LQ_YHS_c18508/f1p0/1668	86.58212745	462.5737708	-2.4175	6.32E-07	1.22E-05
i2_LQ_YHS_c37475/f1p7/2671	77.50742787	3696.615018	-5.5757	6.34E-07	1.22E-05
i0_LQ_YHS_c3175/f1p0/926	14.72869201	131.4139721	-3.1574	6.35E-07	1.23E-05
i2_HQ_YHS_c4321/f2p2/2987	28.6362134	200.937817	-2.8108	6.36E-07	1.23E-05
i1_LQ_YHS_c10901/f1p0/1978	144.1894064	756.0683709	-2.3906	6.37E-07	1.23E-05
i2_HQ_YHS_c21094/f2p0/2271	0.991590972	44.38805846	-5.4843	6.40E-07	1.23E-05
i3_LQ_YHS_c3958/f1p18/3252	458.7374011	5000.310402	-3.4463	6.47E-07	1.25E-05
i1_HQ_YHS_c40542/f19p2/1838	10012.62425	92.91772309	6.7517	6.48E-07	1.25E-05
i2_LQ_YHS_c53426/f1p3/2213	179.8727573	1026.148833	-2.5122	6.49E-07	1.25E-05
i2_LQ_YHS_c38072/f1p21/2365	107.6444899	22302.88731	-7.6948	6.54E-07	1.26E-05
i2_HQ_YHS_c24198/f2p0/2963	172.9415247	964.654369	-2.4797	6.68E-07	1.28E-05
i4_LQ_YHS_c4121/f1p16/4251	1.263932271	55.66868564	-5.4609	6.71E-07	1.29E-05
i2_LQ_YHS_c35146/f1p0/2492	44.14250247	270.4067281	-2.6149	6.72E-07	1.29E-05
i1_LQ_YHS_c39627/f1p3/1163	1360.916877	232.3122257	2.5504	6.75E-07	1.30E-05
i1_LQ_YHS_c25321/f1p3/1905	18598.82976	0	Inf	6.80E-07	1.31E-05
i2_LQ_YHS_c42062/f1p2/2537	2.235805642	237.3184859	-6.7299	6.93E-07	1.33E-05
i1_LQ_YHS_c12831/f1p0/1923	103.6163966	541.9288354	-2.3869	6.97E-07	1.34E-05
i3_LQ_YHS_c8543/f1p0/3432	103.0288095	539.7666924	-2.3893	6.97E-07	1.34E-05
i2_LQ_YHS_c37385/f1p0/2377	0	31.38937532	-Inf	7.02E-07	1.34E-05
i2_LQ_YHS_c25404/f1p0/2467	0	31.34320625	-Inf	7.07E-07	1.35E-05
i3_HQ_YHS_c1352/f5p0/3545	296.4914607	31.89352516	3.2167	7.13E-07	1.37E-05
i5_LQ_YHS_c1921/f1p0/5395	3.125008933	98.51471117	-4.9784	7.18E-07	1.37E-05
i1_LQ_YHS_c13522/f1p0/1250	392.5011509	2969.12838	-2.9193	7.22E-07	1.38E-05
i2_LQ_YHS_c33292/f1p1/2386	152.640553	17.19613675	3.15	7.22E-07	1.38E-05
i1_LQ_YHS_c14528/f1p3/1217	349.1996899	2316.652756	-2.7299	7.23E-07	1.38E-05
i1_LQ_YHS_c13504/f1p2/1253	393.5208475	3046.941244	-2.9528	7.32E-07	1.40E-05
i1_HQ_YHS_c3982/f2p8/1823	169.0671692	1359.140553	-3.007	7.46E-07	1.42E-05
i2_LQ_YHS_c53442/f1p0/2108	53.36902376	310.4619489	-2.5403	7.46E-07	1.42E-05
i2_LQ_YHS_c13569/f1p0/2771	12.67920789	125.8486177	-3.3112	7.50E-07	1.43E-05
i2_LQ_YHS_c20240/f1p13/2886	484.7241497	7288.467738	-3.9104	7.51E-07	1.43E-05
i1_LQ_YHS_c27544/f1p0/1311	0	121.0895082	-Inf	7.54E-07	1.44E-05
i2_LQ_YHS_c26466/f1p0/2662	111.4241278	11.23038776	3.3106	7.58E-07	1.45E-05
i2_LQ_YHS_c26644/f1p0/2281	64.59754461	0	Inf	7.60E-07	1.45E-05
i1_HQ_YHS_c40264/f11p0/1301	256.1345547	1486.421349	-2.5369	7.62E-07	1.45E-05
i2_LQ_YHS_c6817/f1p0/2980	1.983181944	66.18117756	-5.0605	7.64E-07	1.45E-05
i1_LQ_YHS_c6839/f1p0/1641	0.631966135	40.07152855	-5.9866	7.72E-07	1.47E-05
i2_LQ_YHS_c41940/f1p0/2110	485.0677991	5203.797339	-3.4233	7.76E-07	1.48E-05
i1_LQ_YHS_c36830/f1p7/1798	2.440581003	61.27538671	-4.65	7.78E-07	1.48E-05
i2_LQ_YHS_c23303/f1p2/2371	3.268095583	66.69204188	-4.351	7.84E-07	1.49E-05
i1_HQ_YHS_c2254/f3p4/1573	1.089365194	322.8411531	-8.2112	7.98E-07	1.52E-05
i2_HQ_YHS_c17659/f3p0/2341	0	31.03384452	-Inf	7.99E-07	1.52E-05
i2_HQ_YHS_c12086/f3p1/2743	71.39428913	388.4626949	-2.4439	8.00E-07	1.52E-05
i2_HQ_YHS_c24468/f2p2/2130	35.23108225	228.3936043	-2.6966	8.02E-07	1.52E-05
i4_LQ_YHS_c13005/f1p2/4069	29.32224377	201.7814652	-2.7827	8.02E-07	1.52E-05
i2_HQ_YHS_c32668/f2p0/2445	32.18104475	226.2544176	-2.8137	8.02E-07	1.52E-05
i2_HQ_YHS_c20684/f2p5/2339	69.41550166	395.9883223	-2.5121	8.04E-07	1.52E-05
i1_LQ_YHS_c13913/f1p0/1154	718.5032749	138.0544859	2.3798	8.08E-07	1.53E-05
i3_LQ_YHS_c4608/f1p0/3251	4.696104245	72.04242408	-3.9393	8.08E-07	1.53E-05
i4_HQ_YHS_c2275/f2p13/4772	0	487.1687475	-Inf	8.08E-07	1.53E-05
i1_LQ_YHS_c29107/f1p0/1317	0	207.3566057	-Inf	8.11E-07	1.54E-05
i2_LQ_YHS_c24223/f2p0/2788	95.18636787	501.1533119	-2.3964	8.12E-07	1.54E-05
i2_LQ_YHS_c54352/f1p5/2442	0	247.9028242	-Inf	8.14E-07	1.54E-05
i2_LQ_YHS_c49012/f1p0/2299	0	30.40465598	-Inf	8.18E-07	1.55E-05
i1_LQ_YHS_c18736/f1p2/1647	177.0204463	3.646108454	5.6014	8.20E-07	1.55E-05
i2_LQ_YHS_c21691/f1p0/2794	27.22999988	187.3328131	-2.7823	8.21E-07	1.55E-05
i2_HQ_YHS_c2026/f2p0/2336	0	35.67197957	-Inf	8.25E-07	1.56E-05
i4_LQ_YHS_c3962/f1p0/4969	114.1093945	593.5799921	-2.379	8.30E-07	1.57E-05
i3_LQ_YHS_c8539/f1p0/3226	0	38.31488381	-Inf	8.37E-07	1.58E-05
i3_LQ_YHS_c5839/f1p0/3956	62.28998444	355.7523411	-2.5138	8.56E-07	1.61E-05
i2_LQ_YHS_c56364/f1p0/2420	20.48061185	156.0173317	-2.9294	8.61E-07	1.62E-05
i1_LQ_YHS_c14593/f1p27/1166	737.934676	140.2963633	2.395	8.66E-07	1.63E-05
i1_HQ_YHS_c7489/f2p0/1553	161.5824448	876.0922877	-2.4388	8.83E-07	1.66E-05
i1_LQ_YHS_c23619/f1p2/1910	167.1054616	1.530655327	6.7705	8.84E-07	1.66E-05
i1_LQ_YHS_c26033/f1p0/1766	38.49503949	240.0385688	-2.6405	8.87E-07	1.67E-05
i2_LQ_YHS_c53393/f1p0/2915	0	48.04688334	-Inf	8.95E-07	1.68E-05
i1_LQ_YHS_c12799/f1p13/1807	10.64150335	131.9973041	-3.6327	9.11E-07	1.71E-05
i1_LQ_YHS_c24523/f1p0/1812	431.0786415	3559.239618	-3.0455	9.11E-07	1.71E-05
i2_LQ_YHS_c15040/f1p4/2222	27.29709071	191.6421962	-2.8116	9.11E-07	1.71E-05
i1_LQ_YHS_c23161/f1p0/1656	107.3371498	547.1181936	-2.3497	9.15E-07	1.72E-05
i2_LQ_YHS_c54902/f1p0/2189	6.898360286	94.58961603	-3.7774	9.18E-07	1.72E-05
i1_LQ_YHS_c14083/f1p47/1350	50.83148231	289.2738452	-2.5086	9.43E-07	1.77E-05
i2_LQ_YHS_c56317/f1p5/1950	293.5003175	2582.450145	-3.1373	9.43E-07	1.77E-05
i2_HQ_YHS_c61598/f6p3/2062	381.7824395	2943.792912	-2.9469	9.45E-07	1.77E-05
i2_LQ_YHS_c34164/f1p0/2501	24.0735189	168.9480438	-2.8111	9.48E-07	1.78E-05
i4_LQ_YHS_c4461/f1p1/4565	78.27270446	439.1634613	-2.4882	9.48E-07	1.78E-05
i3_HQ_YHS_c1739/f3p0/3632	195.7699745	1058.315312	-2.4345	9.51E-07	1.78E-05

i1_LQ_YHS_c21562/f1p6/1733	32.97903677	473.5466914	-3.8439	9.52E-07	1.78E-05
i4_LQ_YHS_c12992/f1p0/4116	113.3845811	11.77347963	3.2676	9.63E-07	1.80E-05
i3_LQ_YHS_c17360/f1p0/3899	102.092811	9.68583046	3.3979	9.69E-07	1.81E-05
i1_LQ_YHS_c8626/f1p0/1625	201.6712543	1091.565451	-2.4363	9.73E-07	1.82E-05
i1_LQ_YHS_c3155/f1p2/1905	187.7527097	13.00595085	3.8516	9.76E-07	1.82E-05
i1_LQ_YHS_c8276/f1p0/1757	15.40010172	146.6101742	-3.251	9.81E-07	1.83E-05
i4_LQ_YHS_c15780/f1p0/4067	0.514474312	51.95579166	-6.658	9.83E-07	1.84E-05
i2_LQ_YHS_c44239/f1p0/2093	152.2909102	794.6832616	-2.3836	9.83E-07	1.84E-05
i1_LQ_YHS_c18183/f1p0/1933	13.70689275	124.3797315	-3.1818	9.87E-07	1.84E-05
i4_LQ_YHS_c10849/f1p7/4282	4.989426912	76.77744063	-3.9437	9.88E-07	1.84E-05
i0_HQ_YHS_c235/f4p0/510	5064.925257	3.215458624	10.621	9.92E-07	1.85E-05
i1_LQ_YHS_c4433/f1p2/1912	28.16277672	206.3804187	-2.8734	9.95E-07	1.86E-05
i4_LQ_YHS_c4326/f1p0/4816	532.6567294	100.4446618	2.4068	9.95E-07	1.86E-05
i1_LQ_YHS_c39069/f1p8/1301	209.5202014	1130.175432	-2.4314	1.00E-06	1.87E-05
i1_HQ_YHS_c2838/f7p0/1694	1853.419538	5.987431231	8.274	1.01E-06	1.88E-05
i1_LQ_YHS_c7266/f1p0/1607	185.217551	995.0735175	-2.4256	1.02E-06	1.90E-05
i2_LQ_YHS_c19231/f1p0/2534	8.54162662	98.65507693	-3.5298	1.02E-06	1.90E-05
i2_LQ_YHS_c21546/f1p5/2465	0	29.87546609	-Inf	1.03E-06	1.92E-05
i2_LQ_YHS_c53355/f1p3/2762	22.43025256	156.3879907	-2.8016	1.03E-06	1.93E-05
i1_LQ_YHS_c35349/f1p0/2014	125.6541019	657.9638919	-2.3886	1.04E-06	1.93E-05
i1_LQ_YHS_c25512/f1p0/1890	0	41.72840813	-Inf	1.04E-06	1.93E-05
i1_LQ_YHS_c13687/f1p25/1357	136.6344103	689.4512518	-2.3351	1.04E-06	1.94E-05
i1_LQ_YHS_c35041/f1p0/1777	3.514842055	281.703147	-6.3246	1.04E-06	1.94E-05
i2_LQ_YHS_c27285/f1p16/2797	9.500473397	118.3700264	-3.6392	1.04E-06	1.94E-05
i2_LQ_YHS_c26582/f1p1/2795	12.11562148	111.2137109	-3.1984	1.05E-06	1.94E-05
i2_HQ_YHS_c6956/f2p8/2688	196.5477822	1032.251126	-2.3928	1.05E-06	1.95E-05
i2_LQ_YHS_c55087/f1p2/2609	111.1562468	584.0560586	-2.3935	1.06E-06	1.96E-05
i1_LQ_YHS_c20520/f1p3/1923	97.5200758	513.1729183	-2.3957	1.07E-06	1.98E-05
i2_HQ_YHS_c32520/f2p0/2530	0	30.12025409	-Inf	1.07E-06	1.98E-05
i4_LQ_YHS_c4295/f1p3/4774	0	132.4662686	-Inf	1.07E-06	1.99E-05
i2_LQ_YHS_c37358/f1p1/2317	52.88254374	306.9013048	-2.5369	1.08E-06	1.99E-05
i2_HQ_YHS_c32417/f2p15/2430	3.344888437	62.91737761	-4.2334	1.08E-06	2.01E-05
i3_LQ_YHS_c12170/f1p7/4427	162.6787116	850.2870142	-2.3859	1.09E-06	2.01E-05
i2_LQ_YHS_c15108/f1p0/2034	39.36579732	240.4392029	-2.6107	1.09E-06	2.01E-05
i2_HQ_YHS_c1380/f8p0/2198	9.940690765	868.231403	-6.4486	1.09E-06	2.02E-05
i1_LQ_YHS_c23607/f1p0/1570	35.06413966	220.1450648	-2.6504	1.10E-06	2.04E-05
i3_LQ_YHS_c4504/f1p0/3143	19.54101593	148.887716	-2.9296	1.10E-06	2.04E-05
i2_LQ_YHS_c3596/f1p3/2513	38.47152221	231.3765997	-2.5884	1.11E-06	2.05E-05
i2_LQ_YHS_c44819/f1p0/2090	50.82145836	1.415488932	5.1661	1.11E-06	2.05E-05
i2_LQ_YHS_c2358/f2p1/2239	52.35933437	292.911189	-2.4839	1.12E-06	2.06E-05
i2_HQ_YHS_c1191/f17p13/2959	387.9695623	2952.280959	-2.9278	1.12E-06	2.06E-05
i2_LQ_YHS_c23366/f1p1/2979	4.781310235	76.4802421	-3.9996	1.12E-06	2.07E-05
i2_LQ_YHS_c19226/f1p2/2141	68.73306509	345.8083728	-2.3309	1.12E-06	2.07E-05
i5_LQ_YHS_c2124/f1p0/5400	3.674304989	64.96158207	-4.144	1.13E-06	2.07E-05
i1_LQ_YHS_c19215/f1p8/1891	472.4550011	8049.859099	-4.0907	1.13E-06	2.08E-05
i2_LQ_YHS_c14421/f1p3/2051	545.8226266	0	Inf	1.13E-06	2.08E-05
i1_LQ_YHS_c24867/f1p2/1696	59.57869807	317.1490641	-2.4123	1.13E-06	2.08E-05
i1_HQ_YHS_c5088/f2p5/1757	103.0137137	516.6048473	-2.3262	1.13E-06	2.08E-05
i2_LQ_YHS_c44506/f1p0/2076	0	97.0718913	-Inf	1.14E-06	2.10E-05
i4_LQ_YHS_c8783/f1p0/4550	18.191539	144.5470525	-2.9902	1.14E-06	2.10E-05
i2_LQ_YHS_c50813/f1p1/2230	20.89349551	159.5995383	-2.9333	1.14E-06	2.10E-05
i2_LQ_YHS_c27039/f1p44/2506	250.6437124	1451.726962	-2.5341	1.15E-06	2.11E-05
i1_LQ_YHS_c35601/f1p0/1677	2.678100558	61.13127035	-4.5126	1.15E-06	2.12E-05
i2_LQ_YHS_c4996/f1p2/2321	747.5648589	17.00865589	5.4579	1.16E-06	2.12E-05
i2_LQ_YHS_c11672/f1p1/2897	9.853415602	123.6218278	-3.6492	1.16E-06	2.13E-05
i2_LQ_YHS_c22313/f1p2/2389	49.05260899	274.7641772	-2.4858	1.17E-06	2.14E-05
i5_LQ_YHS_c1972/f1p0/5451	63.99990836	345.5709673	-2.4328	1.17E-06	2.15E-05
i1_HQ_YHS_c29947/f2p0/1355	66.8929363	358.8656299	-2.4235	1.17E-06	2.15E-05
i3_LQ_YHS_c14609/f1p1/3034	10.58061168	381.6033841	-5.1726	1.17E-06	2.15E-05
i2_LQ_YHS_c14769/f1p100/2080	12304.04206	0	Inf	1.18E-06	2.16E-05
i2_LQ_YHS_c51946/f1p1/2671	18.62748996	142.9512703	-2.94	1.18E-06	2.16E-05
i0_LQ_YHS_c1330/f1p0/907	2475.898485	0.353872233	12.772	1.19E-06	2.18E-05
i3_LQ_YHS_c19552/f1p2/3156	152.9343747	790.039474	-2.369	1.20E-06	2.19E-05
i2_HQ_YHS_c1504/f10p2/2331	178.11993	912.0925011	-2.3563	1.20E-06	2.20E-05
i3_HQ_YHS_c1874/f2p0/3163	30.38774464	206.4801443	-2.7644	1.21E-06	2.22E-05
i2_LQ_YHS_c36668/f1p4/3001	115.3966908	593.2585941	-2.3621	1.22E-06	2.22E-05
i2_LQ_YHS_c15202/f1p4/2173	182.9605952	27.69484645	2.7238	1.23E-06	2.24E-05
i2_HQ_YHS_c8216/f2p4/2447	34.32804696	216.6257734	-2.6577	1.23E-06	2.24E-05
i2_LQ_YHS_c26544/f1p4/2206	197.4746411	29.39864719	2.7478	1.23E-06	2.24E-05
i1_LQ_YHS_c10543/f1p0/1424	0	122.2968998	-Inf	1.23E-06	2.24E-05
i3_LQ_YHS_c3983/f1p0/3938	6.960040624	89.92063292	-3.6915	1.24E-06	2.26E-05
i1_LQ_YHS_c14680/f1p0/1097	46.81139286	264.3569733	-2.4976	1.24E-06	2.26E-05
i2_HQ_YHS_c41123/f2p0/2538	0	54.51559661	-Inf	1.25E-06	2.28E-05
i2_LQ_YHS_c54924/f1p2/2294	46.8215365	272.3142421	-2.54	1.26E-06	2.29E-05
i4_LQ_YHS_c8920/f1p1/4561	2.662996416	60.05357943	-4.4951	1.26E-06	2.29E-05
i2_LQ_YHS_c21579/f1p0/2575	28.51365813	410.1825596	-3.8465	1.27E-06	2.30E-05
i1_LQ_YHS_c36513/f1p2/1953	0.359624837	71.26173171	-7.6305	1.28E-06	2.32E-05
i2_LQ_YHS_c8574/f1p2/2194	183.4088009	916.2750611	-2.3207	1.28E-06	2.33E-05
i5_LQ_YHS_c4360/f1p2/5941	221.5226263	1460.482825	-2.7209	1.28E-06	2.33E-05
i2_LQ_YHS_c26738/f1p4/2264	13.45091936	124.1829316	-3.2067	1.29E-06	2.34E-05
i1_LQ_YHS_c39372/f1p0/1227	463.6580306	4174.926191	-3.1706	1.29E-06	2.34E-05
i3_HQ_YHS_c21563/f80p3/3380	0	957.990037	-Inf	1.30E-06	2.36E-05
i1_LQ_YHS_c10680/f1p0/1854	343.9593912	1.799969692	7.5781	1.31E-06	2.37E-05
i2_LQ_YHS_c18875/f1p1/2806	4.705789523	76.47068349	-4.0224	1.31E-06	2.37E-05
i1_LQ_YHS_c2644/f2p0/1929	35.16812977	466.1885928	-3.7286	1.31E-06	2.38E-05
i1_LQ_YHS_c23482/f1p0/1985	387.4225987	67.29469224	2.5253	1.31E-06	2.38E-05
i3_HQ_YHS_c1232/f6p0/3492	465.6819176	16.90739148	4.7836	1.32E-06	2.39E-05
i3_HQ_YHS_c878/f2p0/3244	163.0125561	831.9555608	-2.3515	1.32E-06	2.40E-05
i0_HQ_YHS_c428/f2p0/536	371.2666675	12697.22144	-5.0959	1.32E-06	2.40E-05

i2_LQ_YHS_c38698/f1p0/2404	0	40.18104625	-Inf	1.34E-06	2.42E-05
i3_HQ_YHS_c2245/f2p9/3610	206.1103608	1119.464477	-2.4413	1.35E-06	2.45E-05
i1_HQ_YHS_c1837/f6p0/1758	385.2818734	73.63544247	2.3874	1.36E-06	2.46E-05
i1_LQ_YHS_c13778/f1p0/1195	111.953145	547.2003754	-2.2892	1.37E-06	2.47E-05
i2_LQ_YHS_c29551/f1p2/2061	0	143.1507957	-Inf	1.38E-06	2.49E-05
i1_LQ_YHS_c5337/f1p1/1933	26.63159172	175.5564102	-2.7207	1.38E-06	2.50E-05
i2_LQ_YHS_c38320/f1p4/2725	23.63375152	223.4429844	-3.241	1.40E-06	2.53E-05
i1_LQ_YHS_c12385/f1p3/1985	42.07838932	0.707744466	5.8937	1.41E-06	2.54E-05
i2_LQ_YHS_c18669/f1p3/2261	50.50444011	1510.634574	-4.9026	1.41E-06	2.55E-05
i4_LQ_YHS_c8074/f1p0/4480	290.1477819	50.98489408	2.5086	1.42E-06	2.56E-05
i1_LQ_YHS_c27923/f1p25/1216	240.0316047	1284.571701	-2.42	1.42E-06	2.57E-05
i2_LQ_YHS_c19444/f1p5/2107	258.5955307	1436.696941	-2.474	1.42E-06	2.57E-05
i4_HQ_YHS_c2271/f2p34/4322	135.1385628	669.8787636	-2.3095	1.43E-06	2.58E-05
i1_LQ_YHS_c14435/f1p10/1078	244.7666117	36.47273517	2.7465	1.43E-06	2.58E-05
i3_LQ_YHS_c17367/f1p0/3328	215.3682356	34.48362606	2.6428	1.44E-06	2.59E-05
i1_LQ_YHS_c29102/f1p2/1218	0	29.1899571	-Inf	1.44E-06	2.59E-05
i1_LQ_YHS_c25781/f1p0/1535	37.21601145	417.1601875	-3.4866	1.44E-06	2.60E-05
i2_LQ_YHS_c10626/f1p13/2744	517.7358636	6992.26624	-3.7555	1.45E-06	2.61E-05
i2_LQ_YHS_c43225/f2p1/2046	52.82641033	296.0996353	-2.4868	1.45E-06	2.61E-05
i1_LQ_YHS_c10647/f1p1/1621	82.15742266	421.5631398	-2.3593	1.45E-06	2.61E-05
i2_LQ_YHS_c51185/f1p1/2306	64.10483195	338.4233051	-2.4003	1.46E-06	2.62E-05
i4_LQ_YHS_c9608/f1p5/4975	245.7950301	1355.489249	-2.4633	1.46E-06	2.62E-05
i0_LQ_YHS_c1105/f1p0/903	127.5916901	22762.02834	-7.479	1.47E-06	2.64E-05
i2_LQ_YHS_c55245/f1p2/2133	136.9983254	704.4438688	-2.3623	1.47E-06	2.64E-05
i4_HQ_YHS_c2121/f3p0/4818	141.9211043	15.80572741	3.1666	1.47E-06	2.64E-05
i4_LQ_YHS_c13249/f1p0/4051	153.6928992	20.11341126	2.9338	1.47E-06	2.64E-05
i1_LQ_YHS_c19032/f1p8/1818	7.020457193	215.8430247	-4.9423	1.47E-06	2.64E-05
i1_LQ_YHS_c24015/f1p0/1859	19.78903455	145.9982466	-2.8832	1.48E-06	2.66E-05
i1_LQ_YHS_c21399/f1p0/1722	222.5952477	1279.841239	-2.5235	1.49E-06	2.67E-05
i0_LQ_YHS_c1769/f1p0/718	478.1446734	4052.370221	-3.0832	1.50E-06	2.68E-05
i3_LQ_YHS_c19165/f1p1/3849	9.768667973	177.6915327	-4.1851	1.50E-06	2.68E-05
i1_LQ_YHS_c24443/f1p2/1605	625.9695779	124.0454506	2.3352	1.51E-06	2.70E-05
i3_LQ_YHS_c18550/f1p2/3916	50.27756443	283.2043877	-2.4939	1.51E-06	2.70E-05
i2_LQ_YHS_c27801/f3p0/2023	0	335.1606809	-Inf	1.52E-06	2.71E-05
i2_LQ_YHS_c19135/f1p0/2123	29.50316318	192.228613	-2.7039	1.52E-06	2.73E-05
i4_LQ_YHS_c10622/f1p0/4957	35.46158051	227.1560783	-2.6794	1.53E-06	2.73E-05
i4_LQ_YHS_c8534/f1p0/4432	24.4561859	215.4230795	-3.1389	1.54E-06	2.75E-05
i2_LQ_YHS_c19838/f1p0/2100	17.7732281	145.3327602	-3.0316	1.55E-06	2.77E-05
i1_LQ_YHS_c28437/f1p0/1183	99.01492754	494.8427306	-2.3213	1.55E-06	2.77E-05
i1_LQ_YHS_c18058/f1p2/1933	0	29.17712091	-Inf	1.56E-06	2.78E-05
i4_LQ_YHS_c13012/f1p0/4120	0	29.09256305	-Inf	1.58E-06	2.81E-05
i1_LQ_YHS_c18292/f1p0/1861	15.98132819	131.2819387	-3.0382	1.58E-06	2.82E-05
i2_LQ_YHS_c34415/f1p2/2167	860.779618	169.9113204	2.3409	1.58E-06	2.82E-05
i1_LQ_YHS_c17827/f1p2/1996	0	29.04805254	-Inf	1.59E-06	2.83E-05
i3_LQ_YHS_c13098/f1p7/3337	70.57784328	383.7749935	-2.443	1.59E-06	2.84E-05
i1_LQ_YHS_c14655/f1p0/1237	64.24205813	326.4277065	-2.3452	1.61E-06	2.86E-05
i10_LQ_YHS_c9/f1p0/10378	8.051483366	90.24666165	-3.4865	1.61E-06	2.86E-05
i3_LQ_YHS_c4671/f1p0/3676	1.588735364	49.85862343	-4.9719	1.61E-06	2.86E-05
i4_LQ_YHS_c13894/f1p0/4169	5.885779572	81.81277287	-3.797	1.62E-06	2.88E-05
i1_LQ_YHS_c22586/f1p0/1450	92.23962275	451.5742272	-2.2915	1.62E-06	2.88E-05
i2_LQ_YHS_c20962/f1p2/2142	0.817023896	40.97512692	-5.6482	1.62E-06	2.89E-05
i2_HQ_YHS_c7876/f2p2/2427	49.44035619	277.8032919	-2.4903	1.63E-06	2.90E-05
i2_LQ_YHS_c5117/f1p0/2406	10.13036198	176.90223	-4.1262	1.64E-06	2.90E-05
i1_LQ_YHS_c9651/f1p1/1419	155.3977872	912.1161062	-2.5533	1.64E-06	2.91E-05
i1_LQ_YHS_c34623/f1p0/1387	336.2141938	2024.099001	-2.5898	1.64E-06	2.91E-05
i2_LQ_YHS_c5305/f1p2/2597	37.57310038	219.9485391	-2.5494	1.66E-06	2.95E-05
i4_HQ_YHS_c2343/f2p2/4908	39.20078746	234.8649629	-2.5829	1.66E-06	2.95E-05
i3_LQ_YHS_c19824/f1p1/3963	4.286553523	69.11065481	-4.011	1.67E-06	2.96E-05
i3_LQ_YHS_c8663/f1p0/3285	15.82139852	130.879567	-3.0483	1.68E-06	2.98E-05
i4_LQ_YHS_c11313/f1p11/4374	14.72454528	126.0969216	-3.0982	1.69E-06	2.98E-05
i2_HQ_YHS_c17820/f3p4/2878	147.8956657	750.1730827	-2.3426	1.69E-06	2.99E-05
i2_HQ_YHS_c1632/f4p1/2546	213.3587815	15.68609788	3.7657	1.70E-06	3.00E-05
i2_HQ_YHS_c11324/f2p0/2828	152.7465955	745.869395	-2.2878	1.70E-06	3.01E-05
i3_LQ_YHS_c7273/f1p0/3938	63.00830902	337.8397671	-2.4227	1.72E-06	3.05E-05
i3_LQ_YHS_c12415/f1p1/3325	75.3431494	5.079962502	3.8906	1.74E-06	3.07E-05
i2_HQ_YHS_c61051/f12p0/2154	280.5791788	2818.277863	-3.3283	1.76E-06	3.11E-05
i2_LQ_YHS_c49526/f1p0/2698	69.94878523	356.2526194	-2.3485	1.76E-06	3.11E-05
i2_LQ_YHS_c18701/f1p31/2486	2.15774902	49.61932481	-4.5233	1.76E-06	3.12E-05
i4_LQ_YHS_c13462/f1p0/4104	5.221069242	74.13552197	-3.8277	1.77E-06	3.13E-05
i0_LQ_YHS_c2306/f1p1/880	1.089365194	58.6346536	-5.7502	1.79E-06	3.16E-05
i2_LQ_YHS_c18344/f1p1/2256	8.83703521	116.4543359	-3.7201	1.79E-06	3.16E-05
i2_LQ_YHS_c10065/f1p0/2422	11.57220265	173.294908	-3.9045	1.80E-06	3.17E-05
i2_LQ_YHS_c23090/f1p2/2121	17.50329465	140.5760218	-3.0057	1.80E-06	3.17E-05
i2_LQ_YHS_c54315/f1p0/2160	186.798638	940.6869832	-2.3322	1.80E-06	3.17E-05
i2_LQ_YHS_c38349/f1p0/2145	209.386301	33.76967967	2.6324	1.80E-06	3.18E-05
i1_HQ_YHS_c31448/f3p0/1688	457.8176748	3751.264757	-3.0345	1.81E-06	3.18E-05
i3_LQ_YHS_c4238/f1p2/4027	9.427488595	99.78063503	-3.4038	1.81E-06	3.18E-05
i4_LQ_YHS_c12731/f1p0/4661	2163.922691	351.1414974	2.6235	1.83E-06	3.22E-05
i1_LQ_YHS_c7001/f1p1/1550	122.9696657	605.1502771	-2.299	1.83E-06	3.23E-05
i3_LQ_YHS_c11314/f1p0/3098	2.887489378	56.03637963	-4.2785	1.84E-06	3.24E-05
i2_LQ_YHS_c23141/f1p1/2195	126.2860848	623.4517889	-2.3036	1.85E-06	3.25E-05
i3_LQ_YHS_c13952/f1p0/3070	227.695041	1208.007419	-2.4075	1.85E-06	3.25E-05
i2_HQ_YHS_c41950/f2p0/2605	61.60716732	330.2629148	-2.4224	1.86E-06	3.27E-05
i2_LQ_YHS_c35902/f1p0/2587	42.19873898	248.9728964	-2.5607	1.87E-06	3.28E-05
i1_LQ_YHS_c34537/f1p0/1511	54.36350606	303.5126661	-2.481	1.87E-06	3.28E-05
i1_HQ_YHS_c17187/f2p0/1637	8583.548624	6.456469859	10.377	1.88E-06	3.30E-05
i1_LQ_YHS_c7877/f1p0/1605	37.61300231	216.8615956	-2.5275	1.88E-06	3.30E-05
i1_LQ_YHS_c22361/f1p2/1429	0	111.3302173	-Inf	1.89E-06	3.31E-05
i2_LQ_YHS_c48861/f1p0/2331	84.75634764	445.9008197	-2.3953	1.90E-06	3.33E-05

i1_LQ_YHS_c18573/f1p0/1836	5.068755676	347.4757125	-6.0991	1.92E-06	3.36E-05
i2_HQ_YHS_c12872/f2p0/2801	33.92645101	210.1937915	-2.6312	1.92E-06	3.36E-05
i1_LQ_YHS_c23384/f1p2/1874	2.512760399	53.78233908	-4.4198	1.95E-06	3.41E-05
i2_HQ_YHS_c41584/f2p1/2571	462.8641159	3582.485107	-2.9523	1.95E-06	3.42E-05
i2_LQ_YHS_c25118/f1p1/2297	57.40883081	310.6660561	-2.436	1.96E-06	3.42E-05
i1_HQ_YHS_c2757/f2p0/1959	207.1555708	1.569044125	7.0447	1.96E-06	3.43E-05
i1_LQ_YHS_c17914/f1p3/1853	235.9259706	40.55727373	2.5403	1.96E-06	3.43E-05
i3_LQ_YHS_c5198/f1p0/3100	2.451071687	56.40411429	-4.5243	1.96E-06	3.43E-05
i3_LQ_YHS_c17477/f1p0/4026	9.412384453	109.0474268	-3.5343	1.97E-06	3.44E-05
i0_LQ_YHS_c772/f1p0/901	29547.27922	55.86777758	9.0468	1.97E-06	3.44E-05
i2_LQ_YHS_c29086/f1p0/2059	105.2557185	511.4312463	-2.2806	1.97E-06	3.45E-05
i4_HQ_YHS_c2180/f3p3/4489	86.06162272	433.7999687	-2.3336	1.97E-06	3.45E-05
i4_LQ_YHS_c7740/f1p0/5051	22.7781062	157.23752	-2.7872	1.99E-06	3.47E-05
i5_LQ_YHS_c1221/f1p4/5229	16.53397393	125.0107786	-2.9185	1.99E-06	3.47E-05
i3_LQ_YHS_c2907/f1p10/3134	13.53106191	133.9452605	-3.3073	2.00E-06	3.48E-05
i1_HQ_YHS_c31582/f2p0/2009	228.5293675	1167.75571	-2.3533	2.00E-06	3.48E-05
i2_HQ_YHS_c15557/f10p0/2783	161.2398987	785.3518566	-2.2841	2.03E-06	3.53E-05
i2_LQ_YHS_c38279/f1p3/2578	27.4026091	240.2966672	-3.1324	2.04E-06	3.55E-05
i1_LQ_YHS_c33482/f1p0/1785	2.292880895	54.12673293	-4.5611	2.04E-06	3.56E-05
i1_LQ_YHS_c6002/f1p0/1937	29.90636994	248.6240345	-3.0554	2.05E-06	3.56E-05
i2_LQ_YHS_c25571/f1p0/3007	0	28.2880173	-Inf	2.08E-06	3.63E-05
i2_HQ_YHS_c2318/f8p0/2243	311.4711405	58.14018212	2.4215	2.09E-06	3.63E-05
i3_LQ_YHS_c15081/f1p1/3045	4.804369152	71.52437227	-3.896	2.09E-06	3.64E-05
i1_HQ_YHS_c2880/f2p4/1995	7.678026529	117.3089211	-3.9334	2.10E-06	3.65E-05
i4_LQ_YHS_c8103/f1p6/4786	7.983112022	89.06549451	-3.4798	2.12E-06	3.69E-05
i2_LQ_YHS_c7672/f1p5/2295	195.6330715	2.976752786	6.0383	2.13E-06	3.70E-05
i2_LQ_YHS_c26737/f1p2/2792	16.60232853	147.78139	-3.154	2.15E-06	3.73E-05
i5_LQ_YHS_c3091/f1p2/5626	38.96132679	231.0182248	-2.5679	2.15E-06	3.74E-05
i2_LQ_YHS_c55873/f1p1/2766	37.3808765	223.1568102	-2.5777	2.17E-06	3.76E-05
i2_LQ_YHS_c34178/f1p4/3493	9.904605255	100.3898801	-3.3414	2.19E-06	3.80E-05
i1_LQ_YHS_c27703/f1p0/1065	19.030011	146.9456825	-2.9489	2.19E-06	3.80E-05
i1_HQ_YHS_c39826/f13p0/1903	1249.001656	0	Inf	2.20E-06	3.81E-05
i1_LQ_YHS_c28020/f1p3/1100	140.8491398	657.6418522	-2.2232	2.21E-06	3.83E-05
i3_LQ_YHS_c7217/f1p4/3444	37.90171152	219.1679269	-2.5317	2.22E-06	3.85E-05
i0_LQ_YHS_c3521/f1p0/305	452.8552703	13.95287417	5.0204	2.24E-06	3.88E-05
i1_LQ_YHS_c36471/f1p0/1904	0.272341299	32.94842738	-6.9187	2.24E-06	3.88E-05
i1_LQ_YHS_c20677/f1p7/1373	223.211941	1111.426323	-2.3159	2.25E-06	3.90E-05
i5_LQ_YHS_c3706/f1p6/5436	1025.091587	88.39013691	3.5357	2.26E-06	3.91E-05
i2_LQ_YHS_c58998/f1p1/2150	1.438499347	43.15673325	-4.9069	2.27E-06	3.92E-05
i1_LQ_YHS_c13537/f1p0/1105	245.5811654	1264.02862	-2.3638	2.28E-06	3.94E-05
i4_LQ_YHS_c7964/f1p0/4615	154.3071834	747.3396618	-2.276	2.28E-06	3.94E-05
i2_LQ_YHS_c44743/f1p0/2035	0	28.12162592	-Inf	2.31E-06	3.99E-05
i1_HQ_YHS_c2419/f5p1/1963	227.3344253	1184.649353	-2.3816	2.33E-06	4.02E-05
i3_LQ_YHS_c12014/f1p1/3176	97.60342323	808.2815152	-3.0499	2.33E-06	4.02E-05
i1_LQ_YHS_c26044/f1p0/1827	70.174889	364.9788064	-2.3788	2.33E-06	4.02E-05
i2_LQ_YHS_c22821/f1p0/2851	118.7295113	14.09643729	3.0743	2.33E-06	4.02E-05
i2_LQ_YHS_c4011/f1p0/2897	245.4423117	43.60191738	2.4929	2.35E-06	4.05E-05
i1_HQ_YHS_c3130/f2p0/1859	245.1202851	0	Inf	2.36E-06	4.07E-05
i3_LQ_YHS_c14143/f1p0/3051	151.804794	741.0901334	-2.2874	2.37E-06	4.08E-05
i2_LQ_YHS_c23270/f1p2/2414	44.0979787	256.9523957	-2.5427	2.38E-06	4.09E-05
i1_HQ_YHS_c29305/f4p0/1518	249.3289052	1290.460706	-2.3718	2.38E-06	4.10E-05
i2_LQ_YHS_c51623/f1p0/2406	30.71717794	0	Inf	2.39E-06	4.11E-05
i2_HQ_YHS_c25636/f2p5/2615	148.8333373	730.5861404	-2.2954	2.41E-06	4.15E-05
i4_LQ_YHS_c5933/f1p0/4252	308.5870582	59.06597642	2.3853	2.42E-06	4.17E-05
i0_LQ_YHS_c2323/f1p1/565	81.82672559	452.7792572	-2.4682	2.44E-06	4.19E-05
i2_LQ_YHS_c49397/f1p4/2277	351.0880704	69.78340783	2.3309	2.44E-06	4.20E-05
i2_LQ_YHS_c14407/f1p0/2026	233.9919008	1189.953095	-2.3464	2.46E-06	4.22E-05
i2_LQ_YHS_c19505/f1p0/2229	167.7682883	801.47338	-2.2562	2.46E-06	4.22E-05
i1_LQ_YHS_c11087/f1p17/1994	10.14293022	99.08742599	-3.2882	2.47E-06	4.24E-05
i2_LQ_YHS_c27234/f1p3/2990	13.72326903	115.9424058	-3.0787	2.48E-06	4.26E-05
i2_LQ_YHS_c49537/f1p5/2392	82.80541803	416.2755519	-2.3297	2.48E-06	4.26E-05
i2_HQ_YHS_c27272/f3p0/2796	59.84976723	301.8282161	-2.3343	2.49E-06	4.27E-05
i2_LQ_YHS_c26458/f1p0/2261	261.532472	12.64034777	4.3709	2.52E-06	4.32E-05
i5_LQ_YHS_c3675/f1p2/5115	0	27.54247681	-Inf	2.53E-06	4.34E-05
i2_HQ_YHS_c20564/f3p5/2513	56.07986851	306.5021304	-2.4503	2.54E-06	4.36E-05
i3_LQ_YHS_c5678/f1p0/3857	5.50183205	73.54527546	-3.7406	2.55E-06	4.36E-05
i2_LQ_YHS_c40827/f1p4/2164	83.49447617	6.932222969	3.5903	2.56E-06	4.38E-05
i3_LQ_YHS_c10961/f1p3/3213	1254.243536	139.9011768	3.1643	2.56E-06	4.38E-05
i4_LQ_YHS_c3630/f1p0/4211	31.69905851	408.3174927	-3.6872	2.56E-06	4.38E-05
i2_LQ_YHS_c52441/f1p7/2823	82.61493303	427.0243797	-2.3698	2.58E-06	4.41E-05
i2_LQ_YHS_c8184/f1p2/3268	30.65756677	184.4463087	-2.5889	2.59E-06	4.42E-05
i1_HQ_YHS_c9155/f2p1/1995	0	151.8968101	-Inf	2.59E-06	4.42E-05
i3_HQ_YHS_c15377/f9p0/3192	288.751913	1624.420051	-2.492	2.59E-06	4.42E-05
i1_LQ_YHS_c34645/f1p0/1639	74.74011941	369.3264623	-2.3049	2.59E-06	4.42E-05
i0_HQ_YHS_c319/f2p0/741	378.4209844	13998.75049	-5.2092	2.59E-06	4.42E-05
i2_LQ_YHS_c24639/f1p8/2159	5.171143357	73.46683929	-3.8285	2.61E-06	4.44E-05
i2_HQ_YHS_c19917/f2p3/3009	36.4870765	216.8707949	-2.5714	2.61E-06	4.45E-05
i3_LQ_YHS_c7420/f1p0/3712	569.6506376	81.17908164	2.8109	2.62E-06	4.47E-05
i2_HQ_YHS_c27357/f2p1/2654	60.58870938	320.4896428	-2.4032	2.63E-06	4.48E-05
i2_LQ_YHS_c10446/f1p6/2164	18.13987424	409.059394	-4.4951	2.67E-06	4.54E-05
i4_LQ_YHS_c1960/f1p0/4148	41.55088004	234.1068246	-2.4942	2.68E-06	4.56E-05
i0_LQ_YHS_c689/f1p0/938	25.92490191	419.7023003	-4.017	2.70E-06	4.59E-05
i1_HQ_YHS_c1846/f3p0/1899	301.6738081	1694.707256	-2.49	2.70E-06	4.59E-05
i1_LQ_YHS_c27872/f1p0/1257	4.701981471	70.75090806	-3.9114	2.70E-06	4.59E-05
i2_HQ_YHS_c7470/f2p0/2999	363.660824	9.621296274	5.2402	2.70E-06	4.60E-05
i1_LQ_YHS_c18973/f1p1/1740	356.062776	2127.482883	-2.5789	2.72E-06	4.63E-05
i5_LQ_YHS_c931/f1p0/5049	30.43100464	0	Inf	2.74E-06	4.65E-05
i1_LQ_YHS_c20775/f1p1/1764	99.60255659	0.353872233	8.1368	2.76E-06	4.68E-05
i2_LQ_YHS_c22099/f1p0/2950	8.951644077	221.255654	-4.6274	2.76E-06	4.69E-05

i4_LQ_YHS_c10629/f1p0/4990	55.02568048	281.9751138	-2.3574	2.77E-06	4.70E-05
i1_LQ_YHS_c43784/f1p0/1061	393.9485134	79.91548934	2.3015	2.78E-06	4.72E-05
i1_HQ_YHS_c12217/f3p0/1646	1070.328285	214.3176649	2.3202	2.80E-06	4.74E-05
i2_LQ_YHS_c24882/f1p1/2303	249.1316346	1245.325334	-2.3215	2.82E-06	4.77E-05
i1_LQ_YHS_c19991/f1p1/1654	17634.27259	0.719987877	14.58	2.82E-06	4.78E-05
i2_LQ_YHS_c19971/f1p1/2155	28.24336926	183.6620992	-2.7011	2.83E-06	4.79E-05
i2_HQ_YHS_c10051/f2p4/2339	562.1087997	105.8405412	2.409	2.84E-06	4.81E-05
i2_LQ_YHS_c35627/f1p6/2731	5.023443249	298.3923426	-5.8924	2.85E-06	4.82E-05
i2_HQ_YHS_c7138/f2p0/2640	53.16633432	2.212254406	4.5869	2.86E-06	4.84E-05
i1_LQ_YHS_c21611/f1p0/1445	369.6212578	14518.72272	-5.2957	2.86E-06	4.84E-05
i1_LQ_YHS_c36814/f1p0/1837	64.48798244	636.6433659	-3.3034	2.86E-06	4.84E-05
i1_LQ_YHS_c8358/f1p0/1949	113.0436291	529.6511581	-2.2282	2.87E-06	4.86E-05
i2_LQ_YHS_c7685/f1p1/2853	9.075818533	113.7295586	-3.6474	2.88E-06	4.86E-05
i1_LQ_YHS_c24220/f1p12/1992	28.02477027	531.5013638	-4.2453	2.88E-06	4.87E-05
i1_HQ_YHS_c2700/f3p4/1626	373.576486	2419.527647	-2.6952	2.89E-06	4.88E-05
i4_LQ_YHS_c7045/f1p4/4436	2.995754284	58.82892915	-4.2955	2.91E-06	4.91E-05
i2_LQ_YHS_c33708/f1p1/2088	50.1860061	270.2661684	-2.429	2.91E-06	4.91E-05
i4_LQ_YHS_c10193/f1p1/4676	130.5632426	13.99963602	3.2213	2.93E-06	4.95E-05
i1_LQ_YHS_c36100/f1p2/1536	40948.89086	7.125825527	12.488	2.94E-06	4.95E-05
i1_LQ_YHS_c36329/f1p25/1601	870.282681	178.0899572	2.2889	2.94E-06	4.96E-05
i2_LQ_YHS_c25447/f1p0/2119	487.3130676	1.439975754	8.4027	2.94E-06	4.96E-05
i1_HQ_YHS_c2641/f2p2/1388	118.1967854	558.8090888	-2.2412	2.95E-06	4.97E-05
i1_LQ_YHS_c42695/f1p0/1945	200.9806356	3.369013817	5.8986	2.95E-06	4.97E-05
i4_LQ_YHS_c7929/f1p1/4696	132.4137867	643.7488118	-2.2814	2.96E-06	4.98E-05
i2_LQ_YHS_c43957/f1p0/2049	28.45784664	176.06447	-2.6292	2.96E-06	4.99E-05
i1_HQ_YHS_c29879/f3p0/1867	317.8881811	16305.73642	-5.6807	2.98E-06	5.01E-05
i2_LQ_YHS_c37544/f1p2/2385	38.44847167	343.7730897	-3.1605	3.00E-06	5.04E-05
i3_LQ_YHS_c5265/f1p0/3483	5361.885617	5.787706968	9.8555	3.00E-06	5.04E-05
i3_LQ_YHS_c9701/f1p3/3126	1.089365194	42.08958762	-5.2719	3.00E-06	5.04E-05
i3_LQ_YHS_c7392/f1p0/3295	9.657858783	597.4444009	-5.951	3.01E-06	5.06E-05
i1_HQ_YHS_c2600/f3p8/1731	384.0370783	14052.2148	-5.1934	3.02E-06	5.07E-05
i1_LQ_YHS_c13939/f1p0/1303	177.5316236	884.3881016	-2.3166	3.03E-06	5.09E-05
i2_HQ_YHS_c6436/f6p1/2512	67.7649579	344.7154745	-2.3468	3.03E-06	5.09E-05
i1_HQ_YHS_c37778/f3p0/1338	244.3142473	1194.332539	-2.2894	3.04E-06	5.10E-05
i1_LQ_YHS_c17660/f1p0/1456	100.2361084	463.8761842	-2.2103	3.06E-06	5.14E-05
i2_LQ_YHS_c53603/f1p2/2400	235.3957232	1655.515308	-2.8141	3.07E-06	5.15E-05
i3_LQ_YHS_c8394/f1p0/4011	3.942032829	128.4269086	-5.0259	3.08E-06	5.16E-05
i2_LQ_YHS_c14949/f1p2/2054	249.658475	45.68281252	2.4502	3.10E-06	5.20E-05
i5_LQ_YHS_c4607/f1p0/5070	197.6594834	930.8270422	-2.2355	3.12E-06	5.23E-05
i2_LQ_YHS_c20756/f1p0/2302	164.5485401	794.6729927	-2.2718	3.13E-06	5.25E-05
i1_HQ_YHS_c14866/f17p0/1933	159.0435438	22876.03119	-7.1683	3.15E-06	5.28E-05
i3_LQ_YHS_c17495/f1p6/3198	563.3878877	6642.416435	-3.5595	3.16E-06	5.29E-05
i3_LQ_YHS_c10724/f1p4/3347	148.6844931	728.7911403	-2.2933	3.16E-06	5.29E-05
i2_LQ_YHS_c42175/f1p2/2023	25.23586052	164.2241653	-2.7021	3.16E-06	5.29E-05
i1_LQ_YHS_c28083/f1p0/1570	42.87556756	234.3275186	-2.4503	3.17E-06	5.30E-05
i1_LQ_YHS_c2972/f2p7/1717	115.5161405	1024.961254	-3.1494	3.18E-06	5.31E-05
i3_HQ_YHS_c1788/f4p0/3341	35.70878534	219.6359788	-2.6208	3.19E-06	5.33E-05
i1_LQ_YHS_c23861/f1p0/1922	24.09783321	161.6043684	-2.7455	3.20E-06	5.34E-05
i2_LQ_YHS_c37125/f1p3/2262	137.9618061	709.5656147	-2.3627	3.23E-06	5.39E-05
i3_LQ_YHS_c19301/f1p0/3521	5.840467144	77.61812383	-3.7322	3.23E-06	5.39E-05
i3_LQ_YHS_c6546/f1p1/3395	0	44.41135973	-Inf	3.23E-06	5.39E-05
i5_LQ_YHS_c3464/f1p13/5703	57.54983154	341.0374127	-2.567	3.24E-06	5.40E-05
i3_LQ_YHS_c10413/f1p0/3557	170.9980102	809.5615361	-2.2432	3.24E-06	5.40E-05
i3_LQ_YHS_c7130/f1p0/3359	161.7522129	22.51140774	2.8451	3.26E-06	5.43E-05
i2_LQ_YHS_c51897/f1p1/2292	59.55434189	312.3640488	-2.3909	3.26E-06	5.43E-05
i2_LQ_YHS_c55734/f1p4/2659	0	26.92872905	-Inf	3.26E-06	5.43E-05
i1_LQ_YHS_c13574/f1p0/1328	71.42241149	364.6675933	-2.3521	3.26E-06	5.43E-05
i2_HQ_YHS_c10128/f4p0/2493	97.66561935	611.6592772	-2.6468	3.27E-06	5.45E-05
i1_LQ_YHS_c5209/f1p0/1393	1629.252994	299.2211702	2.4449	3.28E-06	5.45E-05
i2_LQ_YHS_c53726/f1p13/2377	528.3018655	4978.383079	-3.2362	3.29E-06	5.47E-05
i3_LQ_YHS_c18545/f1p102/3324	10.87313731	103.090131	-3.2451	3.32E-06	5.51E-05
i2_HQ_YHS_c42555/f10p2/2984	629.2779081	125.5487354	2.3254	3.33E-06	5.53E-05
i4_LQ_YHS_c3034/f1p0/4863	33.20158024	207.5363518	-2.644	3.33E-06	5.54E-05
i1_LQ_YHS_c22754/f1p1/1940	101.1239893	3509.605416	-5.1171	3.34E-06	5.54E-05
i2_LQ_YHS_c3783/f1p20/3086	19.6085735	145.188724	-2.8884	3.35E-06	5.56E-05
i1_LQ_YHS_c43957/f1p0/1025	461.9052883	11830.03309	-4.6787	3.36E-06	5.58E-05
i1_LQ_YHS_c9865/f1p1/1825	1.089365194	41.58216019	-5.2544	3.38E-06	5.60E-05
i4_LQ_YHS_c10508/f1p0/4265	20.07232489	222.8782102	-3.473	3.40E-06	5.63E-05
i1_LQ_YHS_c12363/f1p3/1579	442.5566805	2797.137118	-2.66	3.40E-06	5.64E-05
i1_LQ_YHS_c29068/f1p0/1338	77.30774108	378.028832	-2.2898	3.40E-06	5.64E-05
i4_LQ_YHS_c3933/f1p0/4861	5.607561047	72.54866596	-3.6935	3.43E-06	5.68E-05
i2_LQ_YHS_c35585/f1p0/2510	114.1011585	632.4081087	-2.4705	3.45E-06	5.72E-05
i2_LQ_YHS_c35570/f1p11/2006	436.3944261	12636.6837	-4.8558	3.46E-06	5.72E-05
i4_LQ_YHS_c9158/f1p10/4211	7.74732297	89.77319943	-3.5345	3.46E-06	5.72E-05
i2_LQ_YHS_c27353/f1p0/2456	307.8875585	1682.675399	-2.4503	3.46E-06	5.72E-05
i2_LQ_YHS_c43819/f1p17/2044	465.2371741	11780.91357	-4.6623	3.48E-06	5.76E-05
i4_LQ_YHS_c6517/f1p1/4883	127.8877186	605.3563192	-2.2429	3.53E-06	5.84E-05
i2_LQ_YHS_c5736/f1p0/2542	0	30.82294264	-Inf	3.54E-06	5.84E-05
i3_LQ_YHS_c12606/f1p1/3295	18.21539495	301.8806255	-4.0507	3.54E-06	5.84E-05
i1_HQ_YHS_c5159/f2p0/1931	381.6619462	80.25703794	2.2496	3.55E-06	5.86E-05
i2_LQ_YHS_c43275/f1p2/2031	19.12016912	141.1996814	-2.8846	3.55E-06	5.87E-05
i1_LQ_YHS_c5683/f1p4/1946	0	26.84970011	-Inf	3.57E-06	5.89E-05
i1_LQ_YHS_c8140/f1p1/1469	4.707867072	64.80530252	-3.783	3.59E-06	5.93E-05
i1_LQ_YHS_c19393/f1p1/1921	0	30.82128407	-Inf	3.63E-06	5.99E-05
i3_LQ_YHS_c14410/f1p0/3092	77.70025492	373.9566566	-2.2669	3.63E-06	5.99E-05
i2_HQ_YHS_c27341/f2p0/2154	1185.378508	3.376794089	8.4555	3.64E-06	5.99E-05
i4_LQ_YHS_c2424/f2p0/4508	8.842107029	90.21281509	-3.3509	3.64E-06	5.99E-05
i2_LQ_YHS_c36984/f1p2/2572	29.75451473	185.4048013	-2.6395	3.66E-06	6.02E-05
i1_LQ_YHS_c5117/f1p0/1862	26.76624011	341.4829523	-3.6733	3.66E-06	6.03E-05

i1_LQ_YHS_c17549/f1p0/1780	2.876998694	143.8998186	-5.6444	3.67E-06	6.04E-05
i4_LQ_YHS_c8592/f1p0/4895	58.56561713	310.9013668	-2.4083	3.69E-06	6.07E-05
i1_HQ_YHS_c42186/f3p0/1491	492.5954532	3411.771492	-2.792	3.70E-06	6.08E-05
i1_LQ_YHS_c28431/f1p0/1113	359.7200597	15461.96162	-5.4257	3.70E-06	6.09E-05
i1_HQ_YHS_c27359/f2p2/1147	502.3505992	4214.289195	-3.0685	3.72E-06	6.12E-05
i1_LQ_YHS_c32415/f1p0/1451	179.0694385	823.5918944	-2.2014	3.73E-06	6.13E-05
i3_LQ_YHS_c18658/f1p0/3176	21.70385352	142.7634757	-2.7176	3.74E-06	6.15E-05
i4_LQ_YHS_c8517/f1p2/5131	310.7317759	63.4982655	2.2909	3.74E-06	6.15E-05
i0_LQ_YHS_c1435/f1p0/662	231.0541432	21.97179344	3.3945	3.75E-06	6.15E-05
i2_LQ_YHS_c24840/f1p1/2878	229.1205389	1127.10155	-2.2984	3.76E-06	6.16E-05
i1_LQ_YHS_c3751/f1p2/1874	0	333.709015	-Inf	3.77E-06	6.18E-05
i2_LQ_YHS_c23226/f1p6/2435	491.4436311	103.685	2.2448	3.78E-06	6.20E-05
i1_LQ_YHS_c18475/f1p0/1430	495.7863981	12.0922802	5.3576	3.79E-06	6.22E-05
i2_LQ_YHS_c20536/f1p2/2096	14.6086559	173.6700302	-3.5715	3.80E-06	6.23E-05
i2_LQ_YHS_c64681/f1p0/2018	2.723412986	55.0076228	-4.3361	3.80E-06	6.23E-05
i1_LQ_YHS_c43818/f1p0/1628	2517.423303	0	Inf	3.81E-06	6.24E-05
i2_HQ_YHS_c2796/f4p3/2843	55.48272412	1047.846707	-4.2392	3.82E-06	6.25E-05
i3_LQ_YHS_c11838/f1p0/3262	28.26228982	179.9965587	-2.671	3.83E-06	6.26E-05
i4_LQ_YHS_c11829/f1p0/4516	77.60485505	403.4309437	-2.3781	3.83E-06	6.26E-05
i2_LQ_YHS_c9400/f1p4/2560	167.5747671	802.9870497	-2.2606	3.86E-06	6.31E-05
i3_LQ_YHS_c13683/f1p0/3351	2810.111344	103.7178192	4.7599	3.86E-06	6.31E-05
i1_LQ_YHS_c14609/f1p1/1183	646.1582361	6.258404162	6.6899	3.91E-06	6.38E-05
i2_HQ_YHS_c41079/f2p1/2197	0	134.5148511	-Inf	3.92E-06	6.39E-05
i2_LQ_YHS_c36867/f1p3/3005	174.5741651	27.55357422	2.6635	3.91E-06	6.39E-05
i2_LQ_YHS_c5948/f1p13/2675	277.4242273	1432.442949	-2.3683	3.91E-06	6.39E-05
i4_LQ_YHS_c10301/f1p0/4750	9.320487456	96.32315344	-3.3694	3.92E-06	6.39E-05
i2_LQ_YHS_c9386/f2p0/2287	54.06890288	339.2184467	-2.6493	3.92E-06	6.40E-05
i6_LQ_YHS_c653/f1p0/6949	71.02198474	337.0872786	-2.2468	3.93E-06	6.41E-05
i2_LQ_YHS_c34236/f1p1/2128	13.48908242	114.5315404	-3.0859	3.97E-06	6.47E-05
i1_LQ_YHS_c10110/f1p4/1881	1.634047791	46.60750036	-4.834	3.97E-06	6.48E-05
i1_LQ_YHS_c9534/f1p0/2005	130.0692829	16.81564611	2.9514	4.00E-06	6.52E-05
i1_LQ_YHS_c27633/f1p0/1335	167.1487874	749.0639939	-2.164	4.01E-06	6.53E-05
i1_LQ_YHS_c5544/f1p2/1750	0	26.59097059	-Inf	4.02E-06	6.55E-05
i2_LQ_YHS_c38339/f1p0/2173	66.53108072	401.4965635	-2.5933	4.03E-06	6.56E-05
i1_LQ_YHS_c24151/f1p0/1838	44.20039151	229.4692823	-2.3762	4.05E-06	6.58E-05
i2_LQ_YHS_c10377/f1p21/2778	563.3577177	5467.723778	-3.2788	4.08E-06	6.64E-05
i2_LQ_YHS_c50242/f1p0/2784	0	26.49416932	-Inf	4.09E-06	6.64E-05
i0_LQ_YHS_c1913/f1p2/726	5037.649152	427.084259	3.5602	4.09E-06	6.65E-05
i1_LQ_YHS_c11664/f1p0/1649	117.8199956	541.9264583	-2.2015	4.11E-06	6.67E-05
i1_LQ_YHS_c24132/f1p0/1397	0	26.42683056	-Inf	4.13E-06	6.71E-05
i0_HQ_YHS_c505/f2p0/627	6472.240905	5.189007192	10.285	4.14E-06	6.73E-05
i1_LQ_YHS_c21807/f1p0/1387	132.7406997	788.2112598	-2.57	4.19E-06	6.80E-05
i1_HQ_YHS_c5041/f3p2/1689	141.0980584	650.8570243	-2.2056	4.19E-06	6.80E-05
i1_LQ_YHS_c5400/f1p0/1550	87.6080818	402.0556949	-2.1983	4.21E-06	6.82E-05
i2_LQ_YHS_c21021/f1p2/2637	137.1431342	671.0847719	-2.2908	4.22E-06	6.84E-05
i1_LQ_YHS_c28891/f1p0/1159	35.33602259	350.4699646	-3.3101	4.23E-06	6.85E-05
i1_LQ_YHS_c39720/f1p7/1127	11.42784386	548.9740728	-5.5861	4.23E-06	6.86E-05
i2_LQ_YHS_c22679/f1p0/2550	0	27.40610118	-Inf	4.23E-06	6.86E-05
i1_LQ_YHS_c43975/f1p16/1022	351.4814326	16064.45882	-5.5143	4.24E-06	6.86E-05
i5_LQ_YHS_c2452/f1p2/5793	29.24212634	0	Inf	4.25E-06	6.87E-05
i2_LQ_YHS_c33159/f1p0/2215	48.06562311	253.2641874	-2.3976	4.25E-06	6.88E-05
i1_LQ_YHS_c22058/f1p0/1689	416.9419695	2817.755546	-2.7566	4.27E-06	6.91E-05
i3_LQ_YHS_c17188/f1p0/3852	8.87819254	92.4395247	-3.3802	4.29E-06	6.93E-05
i1_LQ_YHS_c36183/f1p12/1489	366.9551786	56.0797848	2.7101	4.29E-06	6.94E-05
i2_LQ_YHS_c41823/f1p11/2306	146.6972314	1385.977829	-3.24	4.30E-06	6.95E-05
i1_LQ_YHS_c34909/f1p0/1837	8.142574956	208.5311412	-4.6786	4.32E-06	6.98E-05
i3_LQ_YHS_c3855/f1p0/3141	1490.858089	290.0958914	2.3615	4.33E-06	6.99E-05
i1_HQ_YHS_c1629/f15p1/1672	506.4775773	3708.641996	-2.8723	4.37E-06	7.05E-05
i2_LQ_YHS_c34731/f1p1/2670	80.79713312	6.839884829	3.5623	4.37E-06	7.06E-05
i4_LQ_YHS_c6647/f1p0/4577	145.8989665	665.4214668	-2.1893	4.38E-06	7.07E-05
i4_LQ_YHS_c4051/f1p0/4490	90.92668132	432.0426905	-2.2484	4.39E-06	7.08E-05
i4_HQ_YHS_c2444/f2p0/4910	171.6019269	1951.96638	-3.5078	4.40E-06	7.09E-05
i1_HQ_YHS_c17048/f2p0/1812	261.5805214	1293.774597	-2.3063	4.42E-06	7.13E-05
i1_HQ_YHS_c1408/f8p0/1979	1156.901213	75.58936015	3.9359	4.46E-06	7.18E-05
i1_LQ_YHS_c18372/f1p0/2001	3596.148835	5.524514309	9.3464	4.46E-06	7.18E-05
i1_LQ_YHS_c38820/f1p0/1254	228.5575485	1055.38311	-2.2071	4.46E-06	7.18E-05
i3_LQ_YHS_c12677/f1p4/3987	88.2186944	434.7770336	-2.3011	4.45E-06	7.18E-05
i2_LQ_YHS_c9299/f1p3/2564	226.2948173	41.08922752	2.4614	4.46E-06	7.18E-05
i2_LQ_YHS_c27082/f1p0/2893	200.7455778	35.51404146	2.4989	4.47E-06	7.19E-05
i4_LQ_YHS_c7916/f1p0/4593	8.21601812	90.03686511	-3.454	4.47E-06	7.19E-05
i2_LQ_YHS_c39260/f1p1/3125	9.489982713	88.70332945	-3.2245	4.48E-06	7.21E-05
i2_HQ_YHS_c10050/f2p2/2727	137.0202989	620.2565144	-2.1785	4.51E-06	7.26E-05
i1_LQ_YHS_c14665/f1p0/1127	74.38336916	353.4026837	-2.2483	4.54E-06	7.29E-05
i2_LQ_YHS_c18889/f1p1/2253	46.03158181	996.2417946	-4.4358	4.54E-06	7.29E-05
i1_LQ_YHS_c9684/f1p0/1743	274.7199527	1416.374903	-2.3662	4.54E-06	7.30E-05
i4_LQ_YHS_c3617/f1p0/4254	458.4614528	2730.742866	-2.5744	4.55E-06	7.31E-05
i1_LQ_YHS_c10599/f1p0/1830	229.3555968	1183.963543	-2.368	4.58E-06	7.35E-05
i0_HQ_YHS_c3676/f5p4/800	308.7322284	17600.94375	-5.8332	4.59E-06	7.36E-05
i2_LQ_YHS_c25873/f1p2/2384	24.74283431	160.1342176	-2.6942	4.61E-06	7.39E-05
i1_LQ_YHS_c10581/f1p1/1391	12.45091525	106.1458325	-3.0917	4.64E-06	7.43E-05
i3_LQ_YHS_c12126/f1p0/3240	16.66656152	123.6903521	-2.8917	4.67E-06	7.49E-05
i3_LQ_YHS_c11660/f1p7/3163	0	26.12193197	-Inf	4.69E-06	7.52E-05
i2_LQ_YHS_c25678/f1p14/2378	292.5948635	1444.769554	-2.3039	4.70E-06	7.52E-05
i3_HQ_YHS_c1961/f3p0/3947	49.85959219	263.5666098	-2.4022	4.70E-06	7.52E-05
i1_LQ_YHS_c23429/f1p2/1594	48.41396023	249.7637737	-2.3671	4.71E-06	7.53E-05
i1_LQ_YHS_c18266/f1p0/1967	0	42.16683823	-Inf	4.72E-06	7.56E-05
i1_LQ_YHS_c4121/f1p16/1796	193.2553304	916.280439	-2.2453	4.73E-06	7.56E-05
i4_HQ_YHS_c2089/f3p0/4391	0	30.84130775	-Inf	4.73E-06	7.56E-05
i2_LQ_YHS_c11793/f6p1/2423	367.7365981	2173.804167	-2.5635	4.74E-06	7.57E-05

i2_LQ_YHS_c21590/f1p9/2040	91.34371171	429.0211136	-2.2317	4.76E-06	7.61E-05
i1_LQ_YHS_c29007/f1p0/1274	13992.12915	443.4908634	4.9796	4.78E-06	7.63E-05
i2_LQ_YHS_c12397/f1p2/2575	29.1225486	178.8343506	-2.6184	4.80E-06	7.66E-05
i2_LQ_YHS_c52819/f1p1/2173	73.31130534	5.993552937	3.6126	4.80E-06	7.66E-05
i0_LQ_YHS_c2045/f1p0/821	366.828631	15927.11347	-5.4402	4.81E-06	7.67E-05
i2_LQ_YHS_c54915/f1p1/2416	87.04331781	414.1094655	-2.2502	4.86E-06	7.75E-05
i5_HQ_YHS_c745/f2p0/5435	0	25.85933208	-Inf	4.89E-06	7.80E-05
i2_HQ_YHS_c60702/f21p3/2568	133.934481	628.5481465	-2.2305	4.90E-06	7.81E-05
i2_LQ_YHS_c22656/f1p2/2614	124.513048	2.238399793	5.7977	4.92E-06	7.83E-05
i2_LQ_YHS_c19554/f1p1/2953	51.1281463	328.1728192	-2.6823	4.92E-06	7.84E-05
i1_LQ_YHS_c33931/f1p4/1753	44.93980877	239.6174381	-2.4147	4.92E-06	7.84E-05
i1_LQ_YHS_c33259/f1p2/1860	31.02385749	186.6018487	-2.5885	4.93E-06	7.85E-05
i0_LQ_YHS_c3218/f1p0/950	195.6913398	898.5893495	-2.1991	4.94E-06	7.85E-05
i1_HQ_YHS_c5577/f2p0/1891	567.9529457	124.1373157	2.1938	4.94E-06	7.85E-05
i3_LQ_YHS_c12917/f1p0/3316	94.58083547	421.6280646	-2.1564	4.95E-06	7.87E-05
i3_LQ_YHS_c17851/f1p0/3393	6.808532464	269.411509	-5.3063	4.96E-06	7.89E-05
i4_LQ_YHS_c3609/f1p0/4548	79.01156042	6.901614442	3.5171	4.98E-06	7.91E-05
i1_HQ_YHS_c27271/f2p0/1154	3130.301972	1.793847987	10.769	4.99E-06	7.92E-05
i3_HQ_YHS_c5087/f3p0/3482	229.2490014	43.25246874	2.4061	4.99E-06	7.92E-05
i3_LQ_YHS_c4625/f1p8/3487	69.44464841	491.1127324	-2.8221	5.01E-06	7.96E-05
i2_LQ_YHS_c22551/f1p2/2636	6.854311627	108.9362494	-3.9903	5.02E-06	7.96E-05
i3_LQ_YHS_c5338/f1p0/3785	484.149576	3300.303122	-2.7691	5.04E-06	7.99E-05
i1_LQ_YHS_c39359/f1p0/1069	151.1874653	674.5952606	-2.1577	5.05E-06	8.01E-05
i1_HQ_YHS_c37827/f2p5/1111	461.30222	12934.45383	-4.8094	5.09E-06	8.07E-05
i2_LQ_YHS_c34812/f1p0/2468	39.46436858	341.5324781	-3.1134	5.10E-06	8.09E-05
i2_LQ_YHS_c36165/f1p3/2415	42.70273936	227.490479	-2.4134	5.11E-06	8.10E-05
i4_LQ_YHS_c10091/f1p3/4395	61.87710915	305.4041417	-2.3032	5.11E-06	8.10E-05
i1_LQ_YHS_c35819/f1p2/1419	73.75488915	343.6305566	-2.22	5.14E-06	8.14E-05
i1_LQ_YHS_c1975/f5p2/1747	555.2865669	4273.617644	-2.9442	5.15E-06	8.15E-05
i1_LQ_YHS_c7472/f1p0/1729	227.7851262	1066.296002	-2.2269	5.16E-06	8.16E-05
i5_LQ_YHS_c1620/f1p0/5584	13.45680496	111.9135158	-3.056	5.20E-06	8.22E-05
i3_LQ_YHS_c12570/f1p0/3424	39.3083834	220.7392224	-2.4894	5.20E-06	8.22E-05
i3_LQ_YHS_c4164/f1p2/3194	0	130.0992685	-Inf	5.27E-06	8.33E-05
i1_HQ_YHS_c3620/f2p0/1584	44.12485405	686.162312	-3.9589	5.27E-06	8.33E-05
i4_LQ_YHS_c12627/f1p1/4323	98.74765806	458.1857922	-2.2141	5.29E-06	8.36E-05
i3_LQ_YHS_c19314/f1p0/3656	2.774611013	64.06193316	-4.5291	5.37E-06	8.47E-05
i1_HQ_YHS_c35624/f2p3/1411	3546.705364	141.2949835	4.6497	5.38E-06	8.49E-05
i1_LQ_YHS_c20612/f1p3/1740	1195.356841	251.1810085	2.2506	5.51E-06	8.69E-05
i2_LQ_YHS_c33886/f1p10/2508	158.0834177	720.1237944	-2.1876	5.56E-06	8.77E-05
i2_LQ_YHS_c26785/f1p4/2901	192.0280258	853.3964244	-2.1519	5.58E-06	8.80E-05
i1_HQ_YHS_c17080/f2p0/1626	350.5391417	1879.218874	-2.4225	5.60E-06	8.82E-05
i1_LQ_YHS_c14608/f1p4/1087	8.699817412	89.75424154	-3.3669	5.61E-06	8.84E-05
i2_LQ_YHS_c3441/f1p3/2194	461.6004542	0	Inf	5.63E-06	8.88E-05
i1_LQ_YHS_c6156/f1p0/1825	110.3607045	504.3007934	-2.1921	5.65E-06	8.90E-05
i1_HQ_YHS_c2898/f3p0/1859	9414.547994	103.1790718	6.5117	5.67E-06	8.92E-05
i3_LQ_YHS_c4541/f1p4/3800	100.8539901	537.4221289	-2.4138	5.68E-06	8.94E-05
i1_LQ_YHS_c34120/f1p1/1984	103.1135487	463.5829423	-2.1686	5.69E-06	8.95E-05
i2_LQ_YHS_c39438/f1p3/2805	1.84597252	47.37705466	-4.6817	5.69E-06	8.95E-05
i1_LQ_YHS_c24314/f1p1/1391	4.234091728	115.7351024	-4.7726	5.74E-06	9.02E-05
i1_LQ_YHS_c24825/f1p8/1638	454.9518477	0	Inf	5.75E-06	9.03E-05
i2_LQ_YHS_c19822/f1p13/2650	532.6948157	116.0399207	2.1987	5.74E-06	9.03E-05
i2_HQ_YHS_c30140/f3p6/2485	212.9513155	1019.203033	-2.2588	5.75E-06	9.03E-05
i1_LQ_YHS_c34711/f1p12/1961	139.9222402	20.38710855	2.7789	5.78E-06	9.07E-05
i2_LQ_YHS_c40389/f1p2/2880	132.6407533	875.6694467	-2.7229	5.81E-06	9.12E-05
i2_LQ_YHS_c26823/f1p1/2892	8.285661606	151.6660845	-4.1941	5.81E-06	9.12E-05
i1_LQ_YHS_c36523/f1p0/1781	51.14279208	252.7979929	-2.3054	5.83E-06	9.14E-05
i2_LQ_YHS_c51035/f1p3/2739	28.71554216	175.7017122	-2.6132	5.83E-06	9.15E-05
i2_LQ_YHS_c40148/f1p6/2371	81.44841956	915.9245565	-3.4913	5.84E-06	9.16E-05
i2_LQ_YHS_c51171/f1p3/2046	120.0478548	528.705661	-2.1389	5.89E-06	9.22E-05
i3_LQ_YHS_c14283/f1p1/3037	86.59847023	412.1499793	-2.2508	5.89E-06	9.22E-05
i1_LQ_YHS_c33028/f1p0/1596	503.8897713	111.7595283	2.1727	5.92E-06	9.27E-05
i1_HQ_YHS_c1607/f8p0/1716	346.258883	1813.330758	-2.3887	5.93E-06	9.28E-05
i3_LQ_YHS_c18888/f1p1/3526	0.817023896	36.71361214	-5.4898	5.94E-06	9.29E-05
i4_LQ_YHS_c9017/f1p0/4684	0.817023896	44.25780454	-5.7594	5.94E-06	9.30E-05
i4_LQ_YHS_c4575/f1p0/4858	9.697293984	137.757879	-3.8284	5.95E-06	9.31E-05
i2_LQ_YHS_c15106/f1p1/2097	14.24442598	112.5199563	-2.9817	5.99E-06	9.37E-05
i3_LQ_YHS_c19247/f1p4/3368	199.4625562	895.4891017	-2.1666	6.00E-06	9.38E-05
i1_LQ_YHS_c20513/f1p2/1731	5.818213634	130.1117899	-4.483	6.01E-06	9.39E-05
i2_LQ_YHS_c7887/f1p29/3111	253.7097385	19.8168055	3.6784	6.06E-06	9.46E-05
i1_LQ_YHS_c7081/f1p4/1818	1648.923847	177.0976109	3.2189	6.06E-06	9.47E-05
i0_LQ_YHS_c1531/f1p0/736	1474.076709	50.87912697	4.8566	6.10E-06	9.53E-05
i1_HQ_YHS_c36483/f2p1/1875	305.6444766	63.04249653	2.2775	6.14E-06	9.58E-05
i2_LQ_YHS_c3166/f1p1/2403	150.1852065	23.17412914	2.6962	6.14E-06	9.58E-05
i1_LQ_YHS_c3852/f1p9/1917	101.2207978	452.9199728	-2.1618	6.17E-06	9.63E-05
i0_LQ_YHS_c1473/f1p4/409	37.28071118	0	Inf	6.17E-06	9.63E-05
i4_LQ_YHS_c5212/f1p0/4489	13.69893798	111.3805358	-3.0234	6.21E-06	9.69E-05
i2_HQ_YHS_c7636/f4p3/2522	159.0975674	791.0743948	-2.3139	6.23E-06	9.71E-05
i2_LQ_YHS_c64618/f1p0/2005	0	330.0266889	-Inf	6.25E-06	9.73E-05
i1_LQ_YHS_c37007/f1p0/1993	16.39755317	275.0672233	-4.0682	6.25E-06	9.73E-05
i2_LQ_YHS_c12888/f1p0/2164	102.6542743	514.6953143	-2.3259	6.26E-06	9.75E-05
i2_LQ_YHS_c24793/f1p4/2458	260.4251855	1189.22268	-2.1911	6.28E-06	9.77E-05
i2_LQ_YHS_c40904/f1p3/2836	2.057897249	100.8307604	-5.6146	6.29E-06	9.79E-05
i2_LQ_YHS_c4228/f1p0/2612	14.58894667	165.9845211	-3.5081	6.31E-06	9.82E-05
i0_HQ_YHS_c435/f2p0/824	587.567048	9818.878776	-4.0627	6.33E-06	9.84E-05
i2_LQ_YHS_c36038/f1p3/2941	41.1698071	215.9009296	-2.3907	6.35E-06	9.86E-05
i2_LQ_YHS_c40642/f1p0/2619	26.64081026	159.3836607	-2.5808	6.35E-06	9.87E-05
i0_LQ_YHS_c1937/f1p0/395	51.38523864	283.7686827	-2.4653	6.36E-06	9.87E-05
i4_HQ_YHS_c12895/f3p4/4107	92.41084851	411.6090201	-2.1551	6.37E-06	9.90E-05
i2_HQ_YHS_c60561/f2p0/2057	34.26476419	229.6579834	-2.7447	6.38E-06	9.90E-05

i1_LQ_YHS_c22792/f1p0/1536	42.41274963	223.9708343	-2.4007	6.38E-06	9.91E-05
i2_LQ_YHS_c52933/f1p1/2469	22.75676941	204.1092777	-3.165	6.41E-06	9.94E-05
i4_LQ_YHS_c6030/f1p0/4963	2.440581003	60.90548092	-4.6413	6.43E-06	9.97E-05
i1_LQ_YHS_c10099/f1p3/1758	21.23594703	140.8326593	-2.7294	6.46E-06	0.00010016
i2_LQ_YHS_c26527/f1p1/2176	459.397346	101.1043786	2.1839	6.47E-06	0.00010025
i6_LQ_YHS_c402/f1p11/6530	56.77386203	3.261627694	4.1216	6.47E-06	0.00010028
i0_LQ_YHS_c1810/f1p4/711	66.03299944	319.4487612	-2.2743	6.49E-06	0.00010051
i1_LQ_YHS_c14639/f1p0/1233	507.7753188	3474.831702	-2.7747	6.51E-06	0.00010072
i1_LQ_YHS_c8227/f1p0/1875	110.5180815	501.1740503	-2.181	6.51E-06	0.00010077
i1_LQ_YHS_c24534/f1p5/1939	115.067291	516.3396824	-2.1658	6.53E-06	0.00010101
i1_LQ_YHS_c6975/f1p16/1645	1.351215809	39.4015801	-4.8659	6.56E-06	0.00010146
i2_LQ_YHS_c34062/f1p0/2153	26.77132868	164.4673343	-2.619	6.57E-06	0.00010152
i6_LQ_YHS_c656/f1p10/7066	0	83.81341285	-Inf	6.57E-06	0.00010157
i1_LQ_YHS_c24197/f1p0/1813	72.54835405	338.6453044	-2.2228	6.58E-06	0.00010167
i1_LQ_YHS_c13804/f1p0/1372	173.4565328	755.7107469	-2.1233	6.60E-06	0.0001019
i3_LQ_YHS_c10735/f1p0/3192	1.361706493	42.17801585	-4.953	6.66E-06	0.0001028
i1_LQ_YHS_c25266/f1p0/1989	607.980416	6340.974743	-3.3826	6.68E-06	0.00010306
i2_LQ_YHS_c19780/f1p4/2788	22.32197091	144.5812177	-2.6953	6.72E-06	0.00010368
i2_LQ_YHS_c7896/f1p0/2663	132.9898217	632.181928	-2.249	6.73E-06	0.00010388
i1_HQ_YHS_c1960/f9p0/2010	820.6917897	4.270953618	7.5861	6.74E-06	0.00010402
i3_LQ_YHS_c7025/f1p4/3978	0.529578455	33.30676275	-5.9748	6.81E-06	0.00010493
i3_LQ_YHS_c15073/f1p0/3051	36.97642272	198.5675849	-2.425	6.82E-06	0.00010506
i1_LQ_YHS_c34801/f1p0/1727	470.1086293	106.0038884	2.1489	6.82E-06	0.00010516
i2_LQ_YHS_c15132/f1p1/2079	204.1066487	37.33514017	2.4507	6.88E-06	0.00010592
i2_HQ_YHS_c4364/f3p0/2863	0	31.23356878	-Inf	6.88E-06	0.00010602
i1_LQ_YHS_c14386/f1p28/1371	693.2915527	91.5131783	2.9214	6.92E-06	0.00010658
i2_LQ_YHS_c15163/f1p3/2206	310.5926266	0	Inf	7.00E-06	0.00010774
i1_LQ_YHS_c28981/f1p2/1173	2.750279954	52.87708214	-4.265	7.02E-06	0.00010803
i2_LQ_YHS_c62770/f1p1/2295	272.9287936	1285.231375	-2.2354	7.02E-06	0.00010803
i2_LQ_YHS_c44925/f1p2/2067	132.6184089	616.004794	-2.2157	7.03E-06	0.00010811
i2_LQ_YHS_c8054/f1p1/2336	57.95051076	284.7432161	-2.2968	7.05E-06	0.00010843
i1_LQ_YHS_c22053/f1p0/1941	14.01278365	138.8559134	-3.3088	7.07E-06	0.00010868
i3_HQ_YHS_c21154/f20p0/3278	465.3439538	2889.521049	-2.6345	7.08E-06	0.00010879
i1_LQ_YHS_c38254/f1p0/1348	38.36832912	205.40601	-2.4205	7.16E-06	0.00011
i2_HQ_YHS_c6905/f2p3/2606	197.7344549	892.271188	-2.1739	7.17E-06	0.00011012
i1_LQ_YHS_c34578/f1p1/1749	14.65189915	114.4746668	-2.9659	7.18E-06	0.00011022
i1_LQ_YHS_c26250/f1p0/1984	100.007002	438.000306	-2.1308	7.19E-06	0.00011037
i3_LQ_YHS_c21363/f41p1/3831	581.5309189	5371.766747	-3.2075	7.21E-06	0.0001105
i4_LQ_YHS_c9628/f1p1/4327	262.616793	1276.308896	-2.2809	7.22E-06	0.00011065
i1_HQ_YHS_c37766/f3p4/1353	345.0481677	1791.451194	-2.3763	7.22E-06	0.00011068
i1_LQ_YHS_c13447/f1p0/1275	230.2584765	1036.614763	-2.1706	7.24E-06	0.0001109
i2_HQ_YHS_c30205/f23p8/2052	332.656124	1711.596364	-2.3632	7.26E-06	0.00011122
i1_LQ_YHS_c33769/f1p0/1666	407.0222648	15658.8634	-5.2657	7.28E-06	0.00011151
i3_LQ_YHS_c2889/f1p5/3322	34.96556003	197.046408	-2.4945	7.29E-06	0.00011152
i1_HQ_YHS_c1637/f12p2/1833	2443.875433	2.256764909	10.081	7.33E-06	0.00011213
i2_HQ_YHS_c32418/f2p0/2829	142.9868242	21.60287322	2.7266	7.34E-06	0.00011222
i3_LQ_YHS_c13446/f1p0/3603	21.02702495	142.7321953	-2.763	7.34E-06	0.00011222
i1_LQ_YHS_c35574/f1p0/1872	219.1997812	41.67896147	2.3949	7.39E-06	0.00011298
i2_HQ_YHS_c5031/f2p5/2823	65.62694323	308.401985	-2.2325	7.40E-06	0.00011307
i5_LQ_YHS_c2035/f1p0/5456	7.546694332	81.25534555	-3.4285	7.42E-06	0.00011335
i1_LQ_YHS_c11022/f1p3/2006	26.4213975	160.7722522	-2.6052	7.43E-06	0.0001135
i2_LQ_YHS_c14646/f1p0/2038	93.33408489	1.439975754	6.0183	7.43E-06	0.0001135
i1_HQ_YHS_c2664/f2p14/1993	177.8050311	31.05220964	2.5175	7.46E-06	0.00011379
i1_LQ_YHS_c11448/f1p0/1452	89.411364	1.112248908	6.3289	7.52E-06	0.00011478
i3_HQ_YHS_c21547/f14p23/3147	679.7497298	154.0803947	2.1413	7.53E-06	0.00011486
i3_LQ_YHS_c9216/f1p0/3543	18.77311252	128.4115865	-2.774	7.54E-06	0.00011491
i3_LQ_YHS_c14828/f1p0/3040	34.36218299	204.48428	-2.5731	7.54E-06	0.00011497
i1_HQ_YHS_c23763/f2p1/1427	368.0752595	78.57235328	2.2279	7.60E-06	0.00011571
i3_LQ_YHS_c15522/f5p0/3144	0	105.1191282	-Inf	7.59E-06	0.00011571
i4_LQ_YHS_c3080/f1p0/4725	32.52578443	185.0877395	-2.5086	7.64E-06	0.00011637
i2_LQ_YHS_c4693/f1p2/2482	113.2048226	494.5209722	-2.1271	7.65E-06	0.00011643
i1_LQ_YHS_c38966/f1p2/1194	610.9869888	5664.991524	-3.2129	7.67E-06	0.0001168
i6_HQ_YHS_c129/f8p0/6946	1383.094348	204.7129147	2.7562	7.68E-06	0.00011686
i2_LQ_YHS_c10406/f1p2/2510	45.31361265	229.6013097	-2.3411	7.72E-06	0.00011749
i3_LQ_YHS_c3538/f1p0/3380	37.30409202	221.5834216	-2.5704	7.73E-06	0.00011757
i2_LQ_YHS_c50497/f1p12/2434	358.5119389	17503.19923	-5.6095	7.83E-06	0.00011905
i1_HQ_YHS_c1099/f16p0/1745	459.2784025	2866.867136	-2.642	7.85E-06	0.00011931
i1_LQ_YHS_c8810/f1p0/1873	6.272271377	73.5141137	-3.551	7.99E-06	0.00012133
i1_HQ_YHS_c11971/f7p1/1657	245.1832244	1137.517002	-2.214	8.03E-06	0.0001219
i1_HQ_YHS_c17293/f3p8/1586	105.5927595	463.1437997	-2.1329	8.03E-06	0.00012193
i2_HQ_YHS_c3120/f2p0/2214	0	24.76319695	-Inf	8.04E-06	0.00012195
i1_LQ_YHS_c27706/f1p0/1149	150.2045148	1289.883401	-3.1022	8.07E-06	0.00012243
i1_LQ_YHS_c9004/f1p0/1916	646.7948133	6988.308381	-3.4336	8.09E-06	0.00012267
i3_LQ_YHS_c22534/f1p1/3011	157.3742459	5.589048495	4.8155	8.09E-06	0.00012273
i1_LQ_YHS_c17937/f1p0/1640	11.3569366	164.2629876	-3.8544	8.14E-06	0.00012343
i0_LQ_YHS_c1420/f1p0/551	598.4538187	87.83752711	2.7683	8.15E-06	0.00012345
i2_LQ_YHS_c61455/f1p1/2603	41.50510088	215.9458724	-2.3793	8.15E-06	0.00012345
i2_LQ_YHS_c24418/f1p23/2796	37.06276441	619.9265253	-4.0641	8.18E-06	0.00012383
i2_LQ_YHS_c50546/f1p0/2293	200.6597615	1.421610638	7.1411	8.23E-06	0.00012463
i3_LQ_YHS_c14966/f1p0/3064	3.75236161	59.15950012	-3.9787	8.25E-06	0.00012482
i0_LQ_YHS_c750/f1p0/700	633.5739206	6268.494326	-3.3065	8.25E-06	0.00012484
i2_LQ_YHS_c36156/f1p1/2226	40.07949169	217.1102133	-2.4375	8.33E-06	0.00012596
i2_LQ_YHS_c7105/f1p1/2607	6.505982881	147.1040936	-4.4989	8.33E-06	0.00012596
i3_LQ_YHS_c11222/f1p0/3306	0	29.72905884	-Inf	8.34E-06	0.00012611
i2_HQ_YHS_c60757/f3p3/2573	367.4011895	83.76842933	2.1329	8.38E-06	0.00012669
i3_LQ_YHS_c3248/f1p9/3177	208.7842272	1075.112904	-2.3644	8.47E-06	0.00012794
i2_HQ_YHS_c32347/f4p6/2599	11.43833454	100.2864039	-3.1322	8.48E-06	0.00012807
i2_LQ_YHS_c42079/f1p36/3028	4517.688111	11.15981209	8.6611	8.48E-06	0.00012809
i2_LQ_YHS_c19171/f1p4/2754	7.353215061	78.86453657	-3.4229	8.50E-06	0.00012828

i1_LQ_YHS_c6687/f2p0/1882	0	37.20429347	-Inf	8.53E-06	0.00012867
i4_LQ_YHS_c4748/f1p0/4480	396.3690262	8.218050753	5.5919	8.60E-06	0.00012964
i2_LQ_YHS_c18801/f1p1/2808	198.2751439	36.26128006	2.451	8.61E-06	0.00012983
i1_HQ_YHS_c2279/f2p3/1609	474.4865508	2801.740466	-2.5619	8.63E-06	0.0001301
i1_HQ_YHS_c13122/f2p0/1841	280.7943731	7.884202202	5.1544	8.63E-06	0.00013011
i3_LQ_YHS_c16893/f1p0/3074	122.5868049	17.48713336	2.8094	8.64E-06	0.00013013
i2_LQ_YHS_c55119/f1p0/2704	56.13012469	278.7458741	-2.3121	8.65E-06	0.00013027
i1_LQ_YHS_c17484/f1p1/1674	479.2885421	8.905771537	5.75	8.73E-06	0.00013135
i2_LQ_YHS_c37361/f1p4/2373	141.9722856	22.19379272	2.6774	8.75E-06	0.00013163
i1_LQ_YHS_c5007/f1p1/1487	402.6895635	86.72409264	2.2152	8.75E-06	0.00013168
i1_LQ_YHS_c24354/f1p1/1654	0	34.59480233	-Inf	8.79E-06	0.00013221
i1_LQ_YHS_c22226/f1p0/1667	37.94828772	202.8403169	-2.4182	8.80E-06	0.0001323
i2_LQ_YHS_c33071/f1p1/2759	1.301289923	40.62125468	-4.9642	8.89E-06	0.0001337
i3_LQ_YHS_c7994/f1p1/3231	61.5871278	292.4688885	-2.2476	8.90E-06	0.0001337
i2_HQ_YHS_c1981/f8p0/2858	212.9376451	959.3758332	-2.1717	8.94E-06	0.00013422
i1_LQ_YHS_c26449/f1p2/1935	10.3980982	136.459609	-3.7141	8.95E-06	0.00013437
i1_LQ_YHS_c5333/f1p7/1939	7.4661018	74.56230144	-3.32	9.02E-06	0.00013547
i1_LQ_YHS_c19606/f1p3/1589	16.19117537	166.3394245	-3.3609	9.03E-06	0.00013556
i1_LQ_YHS_c28919/f1p0/1091	18.51968341	208.5615174	-3.4933	9.04E-06	0.00013569
i2_LQ_YHS_c28557/f1p0/2040	97.0576456	455.1822293	-2.2295	9.06E-06	0.00013579
i2_LQ_YHS_c54601/f1p2/2399	858.2531093	123.3176022	2.799	9.05E-06	0.00013579
i2_LQ_YHS_c39579/f1p1/2118	18.3988419	162.7092694	-3.1446	9.07E-06	0.00013588
i3_LQ_YHS_c12093/f1p3/3440	25.07398137	158.5234675	-2.6604	9.08E-06	0.00013611
i2_LQ_YHS_c24021/f1p3/2437	52.88475773	261.3883612	-2.3053	9.14E-06	0.00013691
i2_LQ_YHS_c41405/f1p7/2184	12.50210491	103.2314824	-3.0456	9.16E-06	0.00013716
i4_LQ_YHS_c7768/f1p0/4410	18.57629193	124.9908747	-2.7503	9.21E-06	0.00013783
i1_LQ_YHS_c17756/f1p0/1621	149.178375	628.7480312	-2.0754	9.28E-06	0.00013886
i1_LQ_YHS_c26266/f1p1/1894	0	24.37259448	-Inf	9.28E-06	0.00013886
i1_LQ_YHS_c14192/f1p2/1330	195.5250004	847.0822997	-2.1151	9.36E-06	0.00014004
i2_LQ_YHS_c26306/f1p0/3010	1.438499347	85.6827787	-5.8964	9.39E-06	0.00014037
i2_HQ_YHS_c60530/f38p4/2212	1382.205748	0.707744466	10.931	9.45E-06	0.00014127
i1_HQ_YHS_c16848/f3p0/1614	516.5419889	3138.96617	-2.6033	9.53E-06	0.00014246
i2_LQ_YHS_c35967/f1p0/2413	0	26.29997398	-Inf	9.54E-06	0.00014251
i1_LQ_YHS_c28690/f1p0/1193	148.5995851	643.188932	-2.1138	9.55E-06	0.00014254
i1_LQ_YHS_c7805/f1p0/1508	128.8166384	555.40816	-2.1082	9.61E-06	0.0001434
i2_HQ_YHS_c45946/f20p3/2166	2911.571511	479.389891	2.6025	9.62E-06	0.00014364
i4_LQ_YHS_c12772/f1p8/5040	16.56083253	118.0522905	-2.8336	9.68E-06	0.0001444
i1_LQ_YHS_c18438/f1p0/1843	60.5187356	369.0135917	-2.6082	9.73E-06	0.00014514
i1_LQ_YHS_c5392/f1p3/1932	29645.06782	0.719987877	15.329	9.77E-06	0.00014564
i5_LQ_YHS_c4882/f1p0/5100	19.39378256	197.7767016	-3.3502	9.77E-06	0.00014564
i1_LQ_YHS_c24257/f1p1/1722	460.3237047	102.7231485	2.1639	9.78E-06	0.00014571
i1_LQ_YHS_c31983/f1p0/2001	117.2878477	520.9579888	-2.1511	9.78E-06	0.00014574
i1_LQ_YHS_c13992/f1p0/1082	2175.822168	12.57356224	7.435	9.85E-06	0.00014663
i1_LQ_YHS_c28002/f1p0/1330	576.8671914	4190.423371	-2.8608	9.85E-06	0.00014668
i1_LQ_YHS_c31985/f1p0/1892	59.15307624	290.0467167	-2.2938	9.87E-06	0.00014683
i2_LQ_YHS_c37312/f1p0/2849	0	23.97034138	-Inf	9.91E-06	0.00014738
i3_LQ_YHS_c9631/f1p0/3516	98.50396448	1.569044125	5.9722	9.97E-06	0.00014836
i2_HQ_YHS_c22379/f3p2/2290	605.9682644	95.18756372	2.6704	1.00E-05	0.00014875
i1_LQ_YHS_c8866/f2p1/1455	92.74359801	405.2047213	-2.1273	1.00E-05	0.0001488
i2_LQ_YHS_c41580/f1p4/2370	163.7922056	716.497953	-2.1291	1.00E-05	0.00014886
i0_LQ_YHS_c2091/f1p3/949	530.087097	3396.655262	-2.6798	1.01E-05	0.00015009
i2_LQ_YHS_c36374/f1p3/2205	602.3568126	65.42465943	3.2027	1.01E-05	0.00015059
i4_LQ_YHS_c6467/f1p0/4871	3.684795673	56.65565632	-3.9426	1.02E-05	0.00015078
i2_LQ_YHS_c55666/f1p6/2792	3.279858409	54.96141418	-4.0667	1.02E-05	0.00015166
i1_LQ_YHS_c7962/f1p344/1585	0	110.1050138	-Inf	1.02E-05	0.00015168
i3_LQ_YHS_c17976/f1p0/3142	351.7430344	35.8216644	3.2956	1.02E-05	0.00015168
i1_HQ_YHS_c19995/f2p9/1782	506.0529284	36.53999371	3.7917	1.03E-05	0.00015261
i0_LQ_YHS_c2406/f1p2/627	52.60318951	1256.371784	-4.578	1.04E-05	0.00015415
i1_HQ_YHS_c8136/f2p0/1913	0	48.26943586	-Inf	1.05E-05	0.00015546
i2_LQ_YHS_c14191/f1p6/2065	151.9861634	25.0431357	2.6015	1.05E-05	0.00015578
i1_LQ_YHS_c38744/f1p0/1184	28.78806023	226.608364	-2.9767	1.06E-05	0.0001563
i1_HQ_YHS_c16885/f3p0/1722	770.3656026	175.9087041	2.1307	1.06E-05	0.00015636
i0_LQ_YHS_c3118/f1p0/636	332.1098365	34.09183804	3.2842	1.06E-05	0.00015649
i2_LQ_YHS_c10141/f1p3/2422	36.65841357	802.2780169	-4.4519	1.06E-05	0.00015729
i2_LQ_YHS_c18017/f1p0/2371	0	24.03708737	-Inf	1.06E-05	0.00015729
i1_LQ_YHS_c22038/f1p1/1924	148.6912331	836.1042335	-2.4914	1.06E-05	0.00015747
i3_LQ_YHS_c4121/f1p16/3273	275.1038356	58.04491965	2.2447	1.07E-05	0.0001576
i2_LQ_YHS_c35954/f1p0/2993	56.92631203	3.639986748	3.9671	1.07E-05	0.00015767
i3_LQ_YHS_c5896/f1p30/3181	0.272341299	28.08608124	-6.6883	1.07E-05	0.00015833
i1_LQ_YHS_c13369/f2p11/1390	121.668079	509.4542943	-2.066	1.07E-05	0.00015859
i2_LQ_YHS_c35669/f1p1/2536	514.795843	117.4169802	2.1324	1.07E-05	0.00015866
i4_LQ_YHS_c7501/f1p0/4679	6.471169512	69.48455073	-3.4246	1.08E-05	0.00015871
i3_LQ_YHS_c13486/f1p0/3403	0	23.92357954	-Inf	1.08E-05	0.00015977
i2_LQ_YHS_c38593/f1p0/2300	354.8151973	77.57965482	2.1933	1.09E-05	0.00016116
i1_LQ_YHS_c3031/f1p0/1880	194.2678621	36.30587078	2.4198	1.10E-05	0.00016265
i6_LQ_YHS_c611/f1p0/6502	0	23.78673089	-Inf	1.11E-05	0.00016322
i1_LQ_YHS_c14650/f1p5/1224	88.57995517	380.7287308	-2.1037	1.11E-05	0.00016337
i2_LQ_YHS_c7312/f1p3/2155	840.6190605	97.11672154	3.1137	1.11E-05	0.00016337
i2_LQ_YHS_c9145/f1p0/2109	311.0007962	1499.281907	-2.2693	1.11E-05	0.00016337
i1_LQ_YHS_c12478/f1p0/1769	7.616337818	77.06938327	-3.339	1.12E-05	0.00016543
i1_LQ_YHS_c23677/f1p0/1911	75.68450589	380.8793293	-2.3313	1.13E-05	0.00016572
i1_LQ_YHS_c19180/f1p0/1978	246.6933622	50.34274959	2.2929	1.13E-05	0.00016587
i2_HQ_YHS_c17190/f4p0/2799	387.64244	88.58757028	2.1296	1.13E-05	0.00016587
i4_LQ_YHS_c4856/f1p1/4773	14.58894667	106.7135705	-2.8708	1.13E-05	0.00016587
i1_LQ_YHS_c11009/f1p0/1551	120.2127199	897.812193	-2.9008	1.13E-05	0.00016589
i1_LQ_YHS_c28911/f1p0/1294	436.2578198	2509.060038	-2.5239	1.13E-05	0.00016604
i2_LQ_YHS_c41945/f1p5/2774	30.35398443	184.7188193	-2.6054	1.13E-05	0.00016613
i2_LQ_YHS_c50399/f1p0/2139	10.75771466	94.98341586	-3.1423	1.13E-05	0.00016617
i2_HQ_YHS_c20674/f2p2/2210	538.1639563	129.243578	2.058	1.13E-05	0.00016653

i1_HQ_YHS_c29397/f4p22/1882	291.5615361	1392.930674	-2.2563	1.14E-05	0.00016726
i4_LQ_YHS_c5199/f1p6/4543	169.1621442	713.8282315	-2.0772	1.14E-05	0.00016742
i1_LQ_YHS_c6663/f1p0/1736	267.8858972	55.5957784	2.2686	1.14E-05	0.00016777
i2_LQ_YHS_c22531/f1p4/2849	99.87786704	12.16633344	3.0373	1.14E-05	0.00016777
i2_LQ_YHS_c7134/f1p0/2449	77.2592154	350.1250236	-2.1801	1.15E-05	0.00016859
i2_LQ_YHS_c5996/f1p3/2474	50.59649029	248.4257353	-2.2957	1.15E-05	0.00016866
i1_HQ_YHS_c17309/f2p0/1846	50.12017904	243.2241315	-2.2788	1.15E-05	0.00016893
i1_LQ_YHS_c6284/f1p3/1406	311.4885149	1506.440004	-2.2739	1.16E-05	0.00016988
i3_LQ_YHS_c5594/f1p0/3634	180.2329146	818.7873329	-2.1836	1.18E-05	0.00017251
i1_HQ_YHS_c31629/f2p0/1660	1600.180451	1.415488932	10.143	1.18E-05	0.00017313
i1_LQ_YHS_c32229/f1p0/1608	445.8809754	53.35184968	3.063	1.18E-05	0.00017319
i1_LQ_YHS_c8723/f3p1/1651	440.2968753	2527.556754	-2.5212	1.18E-05	0.00017333
i3_LQ_YHS_c19393/f1p1/3554	97.53887668	496.4423475	-2.3476	1.19E-05	0.00017343
i1_LQ_YHS_c18805/f1p0/1837	281.0458934	1289.409867	-2.1978	1.19E-05	0.00017359
i7_LQ_YHS_c120/f1p0/7719	2259.479507	104.3742991	4.4362	1.19E-05	0.00017373
i3_LQ_YHS_c9328/f1p0/3671	145.8569296	2.946144259	5.6296	1.21E-05	0.00017693
i1_LQ_YHS_c12081/f1p0/1885	578.4761482	3921.851924	-2.7612	1.21E-05	0.00017719
i2_LQ_YHS_c11301/f1p8/2178	413.1925841	2157.459826	-2.3844	1.21E-05	0.00017719
i0_LQ_YHS_c1277/f1p0/739	1449.447404	106.7462316	3.7632	1.22E-05	0.00017771
i2_LQ_YHS_c10604/f7p1/2672	660.9588083	157.9821886	2.0648	1.22E-05	0.00017809
i0_LQ_YHS_c998/f1p0/990	1086.357903	2.14772022	8.9825	1.23E-05	0.00017961
i2_LQ_YHS_c35216/f1p4/2846	5.822827092	67.51040939	-3.5353	1.23E-05	0.00017961
i3_LQ_YHS_c16923/f1p0/3621	58.00390603	274.9646942	-2.245	1.23E-05	0.00017961
i2_LQ_YHS_c23059/f1p0/2737	408.0555696	92.58861676	2.1399	1.23E-05	0.00017976
i1_HQ_YHS_c6282/f3p0/1516	342.6118244	1668.438719	-2.2839	1.24E-05	0.00018103
i1_LQ_YHS_c22113/f1p2/1710	421.0390564	2272.118412	-2.432	1.24E-05	0.00018111
i3_LQ_YHS_c11684/f1p5/3294	50.49376394	250.3225063	-2.3096	1.25E-05	0.00018154
i3_LQ_YHS_c4330/f1p0/3450	12.6343622	256.7513266	-4.3449	1.25E-05	0.00018176
i2_LQ_YHS_c2850/f1p0/2347	20.87170874	135.8265907	-2.7021	1.26E-05	0.00018328
i2_LQ_YHS_c5981/f1p3/2141	422.3226979	2269.104189	-2.4257	1.26E-05	0.00018373
i3_LQ_YHS_c11835/f1p0/3531	15.30692421	110.4073869	-2.8506	1.27E-05	0.00018443
i2_LQ_YHS_c64867/f1p1/2075	1047.081973	0	Inf	1.27E-05	0.00018461
i2_LQ_YHS_c33965/f1p1/2107	16.80964817	116.9717961	-2.7988	1.27E-05	0.00018488
i3_LQ_YHS_c13523/f1p3/3843	3.232010072	49.81024256	-3.9459	1.27E-05	0.00018509
i1_LQ_YHS_c11568/f1p1/1440	243.2386064	1075.88392	-2.1451	1.28E-05	0.00018528
i2_LQ_YHS_c21313/f1p1/2510	20.63292541	128.9395106	-2.6437	1.28E-05	0.00018555
i4_LQ_YHS_c2252/f2p3/4781	0	23.41003041	-Inf	1.28E-05	0.00018555
i5_LQ_YHS_c1454/f1p0/5110	267.9708973	16.01994643	4.0641	1.28E-05	0.00018638
i1_LQ_YHS_c29077/f1p2/1273	0	31.24747076	-Inf	1.29E-05	0.00018697
i2_HQ_YHS_c17434/f2p4/2450	247.8216875	52.7223414	2.2328	1.29E-05	0.0001873
i2_LQ_YHS_c49851/f1p0/2340	28.71555053	160.5342984	-2.483	1.29E-05	0.0001873
i1_LQ_YHS_c34858/f1p0/1803	15.63887667	167.5713436	-3.4216	1.30E-05	0.00018858
i1_HQ_YHS_c2327/f5p8/1727	479.2200008	58.02383018	3.046	1.30E-05	0.00018913
i0_LQ_YHS_c1034/f1p0/1001	402.5485676	17592.70183	-5.4497	1.31E-05	0.00018916
i4_LQ_YHS_c13042/f1p0/4358	153.4814077	651.5744411	-2.0859	1.31E-05	0.00018917
i2_LQ_YHS_c23264/f1p29/2702	178.0963588	7.44968201	4.5793	1.31E-05	0.00018993
i3_LQ_YHS_c3834/f1p4/3093	60.44657295	4.707132376	3.6827	1.32E-05	0.00019068
i1_LQ_YHS_c14350/f1p6/1199	0.719249673	30.68340918	-5.4148	1.32E-05	0.00019159
i1_LQ_YHS_c14399/f1p0/1257	543.94708	3350.365935	-2.6228	1.32E-05	0.00019159
i2_HQ_YHS_c60070/f13p3/2123	488.4761595	2852.278597	-2.5458	1.32E-05	0.00019159
i4_LQ_YHS_c10532/f1p0/4621	4.493864794	62.23411997	-3.7917	1.33E-05	0.00019176
i1_LQ_YHS_c3178/f1p23/1872	124.5544076	527.7806138	-2.0832	1.33E-05	0.00019181
i0_LQ_YHS_c3170/f1p0/937	1783.64574	159.3956251	3.4841	1.33E-05	0.00019275
i6_LQ_YHS_c752/f1p0/6207	31.06041811	175.2531702	-2.4963	1.34E-05	0.00019302
i2_HQ_YHS_c56949/f10p4/2323	311.5120991	1453.01816	-2.2217	1.34E-05	0.00019304
i2_LQ_YHS_c26447/f1p1/2370	102.6502641	480.4593215	-2.2267	1.34E-05	0.00019407
i3_HQ_YHS_c2411/f2p0/3662	469.6707492	23.21583507	4.3385	1.35E-05	0.00019415
i2_LQ_YHS_c58240/f1p3/2031	59.63334036	284.7486659	-2.2555	1.35E-05	0.00019463
i5_LQ_YHS_c3521/f1p0/5331	15.67162924	174.1333794	-3.474	1.36E-05	0.0001959
i1_LQ_YHS_c39188/f1p3/1333	52.91117471	245.1184104	-2.2118	1.36E-05	0.00019672
i4_LQ_YHS_c7930/f1p4/4264	0.97648683	34.13745388	-5.1276	1.36E-05	0.00019672
i0_LQ_YHS_c1201/f1p0/774	181.3295417	1241.553504	-2.7755	1.37E-05	0.00019708
i1_LQ_YHS_c18218/f1p0/1886	2167.688864	0	Inf	1.37E-05	0.0001977
i2_LQ_YHS_c59796/f1p2/1742	42.34886368	259.3988582	-2.6148	1.38E-05	0.00019853
i1_LQ_YHS_c24993/f1p6/1966	81.02780848	365.1227683	-2.1719	1.38E-05	0.00019857
i1_HQ_YHS_c41546/f2p2/1502	1986.678403	163.4674472	3.6033	1.38E-05	0.00019871
i3_LQ_YHS_c19410/f1p0/3574	7.388045179	69.32321526	-3.2301	1.38E-05	0.00019916
i2_LQ_YHS_c52674/f1p0/2561	203.8909805	1450.104891	-2.8303	1.39E-05	0.00020007
i2_LQ_YHS_c42009/f1p0/2504	53.8666718	3.530349282	3.9315	1.40E-05	0.00020099
i1_LQ_YHS_c14384/f1p1/1245	187.8355497	774.6816672	-2.0441	1.41E-05	0.00020217
i3_LQ_YHS_c5145/f1p7/3539	26.91902042	287.9896821	-3.4193	1.41E-05	0.00020222
i3_LQ_YHS_c14864/f1p0/3031	771.8408015	184.6953598	2.0632	1.41E-05	0.00020247
i4_LQ_YHS_c10178/f1p0/4616	376.1051297	52.90097619	2.8298	1.42E-05	0.00020455
i5_LQ_YHS_c1679/f1p0/5334	1.634047791	79.91307756	-5.6119	1.43E-05	0.00020467
i2_LQ_YHS_c49607/f1p26/2979	5.808995092	64.7051039	-3.4775	1.43E-05	0.00020479
i2_LQ_YHS_c3254/f1p0/2173	92.65233648	407.8167835	-2.138	1.43E-05	0.00020484
i0_LQ_YHS_c2039/f1p0/729	210.0072391	867.4753718	-2.0464	1.43E-05	0.00020562
i1_LQ_YHS_c3395/f1p0/1945	10.59698796	89.05333132	-3.071	1.44E-05	0.00020592
i1_HQ_YHS_c1502/f7p0/1856	308.5948275	1413.043312	-2.195	1.44E-05	0.0002063
i3_HQ_YHS_c9614/f2p2/3120	8.931459741	83.24773225	-3.2204	1.44E-05	0.00020634
i2_LQ_YHS_c12435/f1p10/2014	21.72228223	136.7837457	-2.6546	1.44E-05	0.00020697
i1_LQ_YHS_c20706/f1p1/1627	263.941709	7.678356234	5.1033	1.45E-05	0.000207
i2_LQ_YHS_c11141/f1p1/2659	158.4029551	650.2617196	-2.0374	1.45E-05	0.000207
i0_LQ_YHS_c3591/f1p2/283	0	23.11903379	-Inf	1.46E-05	0.00020826
i1_LQ_YHS_c17466/f1p7/1693	35.98437339	0.353872233	6.668	1.45E-05	0.00020826
i1_LQ_YHS_c24769/f1p7/1836	0	51.33520965	-Inf	1.46E-05	0.00020847
i6_LQ_YHS_c272/f1p0/6180	0.514474312	30.670573	-5.8976	1.46E-05	0.00020936
i1_LQ_YHS_c9025/f1p0/1877	31.47458229	213.7660395	-2.7638	1.48E-05	0.00021082
i2_HQ_YHS_c15838/f6p2/2179	67.04064478	299.2279243	-2.1581	1.47E-05	0.00021082

i2_LQ_YHS_c14079/f1p3/2076	281.9697724	1312.662642	-2.2189	1.47E-05	0.00021082
i4_LQ_YHS_c14136/f1p0/4130	6.830794349	68.51373337	-3.3263	1.48E-05	0.00021143
i2_LQ_YHS_c20987/f1p1/2575	53.80993522	247.9125394	-2.2039	1.48E-05	0.00021172
i3_LQ_YHS_c19809/f1p0/3106	5.33060629	64.46086913	-3.5961	1.48E-05	0.00021172
i4_LQ_YHS_c10368/f1p0/4338	6.325538579	68.98498379	-3.447	1.48E-05	0.00021172
i1_HQ_YHS_c11487/f2p9/1835	45.08624511	546.0743767	-3.5983	1.49E-05	0.00021254
i2_HQ_YHS_c8792/f2p1/2395	22.78651933	141.5325037	-2.6349	1.49E-05	0.000213
i2_LQ_YHS_c40656/f1p22/2769	54.35507617	255.4562588	-2.2326	1.49E-05	0.00021305
i2_HQ_YHS_c45754/f14p3/2407	27.07469205	187.0947023	-2.7888	1.49E-05	0.00021314
i3_LQ_YHS_c18935/f1p0/3129	54.85342196	268.5107487	-2.2913	1.50E-05	0.00021339
i2_LQ_YHS_c21711/f1p0/2171	3038.398388	10.62615906	8.1595	1.50E-05	0.0002137
i2_HQ_YHS_c61019/f7p0/2126	452.5603968	2511.343694	-2.4723	1.50E-05	0.00021418
i1_LQ_YHS_c34415/f1p2/1375	2.708308843	49.61991759	-4.1955	1.51E-05	0.00021469
i3_LQ_YHS_c10514/f1p5/3232	0	25.56327955	-Inf	1.52E-05	0.00021613
i1_LQ_YHS_c5620/f1p4/1612	33.71453466	208.7198483	-2.6301	1.52E-05	0.00021655
i1_LQ_YHS_c24520/f1p0/1749	99.51733385	11.77794277	3.0789	1.52E-05	0.00021679
i5_LQ_YHS_c1233/f1p3/5136	5.849694061	66.29357893	-3.5024	1.53E-05	0.00021693
i1_LQ_YHS_c7056/f1p24/1552	18481.19788	60.70354374	8.2501	1.53E-05	0.00021722
i1_HQ_YHS_c5405/f6p0/1751	959.018704	1.415488932	9.4041	1.54E-05	0.0002191
i3_LQ_YHS_c8542/f1p5/3327	4.486715426	60.14536546	-3.7447	1.54E-05	0.00021913
i2_HQ_YHS_c46737/f22p3/2898	7077.064747	22.77637873	8.2795	1.55E-05	0.00021954
i1_HQ_YHS_c4874/f3p8/1816	203.5001577	893.4931572	-2.1344	1.55E-05	0.00021997
i2_LQ_YHS_c39714/f1p0/2294	5.163993989	62.69877568	-3.6019	1.56E-05	0.00022083
i1_LQ_YHS_c11967/f1p2/1409	184.625537	764.1518985	-2.0493	1.58E-05	0.00022363
i2_LQ_YHS_c49486/f1p6/2308	22.87380287	139.0940264	-2.6043	1.58E-05	0.00022485
i1_LQ_YHS_c8888/f1p13/1771	16.69723654	114.9203239	-2.783	1.59E-05	0.0002254
i3_LQ_YHS_c4758/f1p0/3411	711.5849048	47.12223505	3.9166	1.59E-05	0.00022601
i3_LQ_YHS_c11329/f1p3/3852	54.64724639	264.6710388	-2.276	1.63E-05	0.00023135
i3_LQ_YHS_c11901/f1p0/3336	0	22.89422993	-Inf	1.64E-05	0.00023247
i2_HQ_YHS_c3784/f4p0/2701	59.83292421	268.2674611	-2.1647	1.64E-05	0.00023248
i3_LQ_YHS_c17153/f1p0/3178	12.42992551	138.6535389	-3.4796	1.64E-05	0.00023287
i1_HQ_YHS_c3200/f2p0/1570	316.3146109	1389.856546	-2.1355	1.65E-05	0.00023348
i2_LQ_YHS_c35905/f1p1/2370	44.73549177	226.3359709	-2.339	1.65E-05	0.00023348
i2_LQ_YHS_c9013/f1p0/2940	295.3456809	63.55904909	2.2162	1.65E-05	0.00023348
i2_HQ_YHS_c32482/f3p3/2265	180.3508002	11.83189211	3.9301	1.65E-05	0.00023363
i2_LQ_YHS_c26718/f1p1/2505	12.61625541	212.7099122	-4.0755	1.65E-05	0.00023391
i3_LQ_YHS_c9515/f1p0/3409	0	22.84193916	-Inf	1.65E-05	0.00023392
i2_LQ_YHS_c6364/f1p0/2156	96.37767076	11.78185268	3.0321	1.66E-05	0.00023409
i1_LQ_YHS_c23936/f1p0/1600	426.0131709	17496.52766	-5.36	1.66E-05	0.00023478
i2_LQ_YHS_c37470/f1p2/2456	5.129172246	64.38902768	-3.65	1.67E-05	0.00023553
i2_HQ_YHS_c3942/f2p0/2119	0	59.2794829	-Inf	1.67E-05	0.000236
i1_HQ_YHS_c15245/f3p0/1388	327.7738191	1485.452577	-2.1801	1.68E-05	0.00023656
i2_LQ_YHS_c33220/f1p119/2849	50.03486174	36239.91515	-9.5004	1.68E-05	0.00023656
i2_LQ_YHS_c36835/f1p2/2806	47.1198113	226.8062346	-2.2671	1.68E-05	0.00023656
i3_LQ_YHS_c11442/f1p7/3623	687.5825674	9299.268819	-3.7575	1.68E-05	0.00023683
i1_LQ_YHS_c17977/f1p1/1823	134.7420554	551.7292312	-2.0338	1.68E-05	0.00023746
i2_LQ_YHS_c18150/f1p0/2331	13.25744847	98.91657147	-2.8994	1.69E-05	0.0002382
i4_LQ_YHS_c3085/f1p2/4881	319.4339605	62.17404892	2.3611	1.70E-05	0.00023907
i4_LQ_YHS_c5503/f1p0/4589	1252.900657	285.9287255	2.1315	1.70E-05	0.00023907
i0_LQ_YHS_c2437/f1p1/836	243.4129999	1012.591677	-2.0566	1.70E-05	0.00023956
i4_LQ_YHS_c3869/f1p0/4306	131.4398526	554.8650692	-2.0777	1.70E-05	0.00023958
i4_HQ_YHS_c2394/f2p0/4816	107.2650984	455.089884	-2.085	1.71E-05	0.00024027
i1_LQ_YHS_c18856/f1p1/1882	0	26.70779556	-Inf	1.71E-05	0.00024031
i3_LQ_YHS_c8282/f1p0/3692	23.33962344	142.3304152	-2.6084	1.72E-05	0.00024253
i2_LQ_YHS_c60270/f2p1/2827	128.5473165	16.36710418	2.9734	1.73E-05	0.00024384
i1_LQ_YHS_c7122/f1p12/1702	66.81569346	289.9562371	-2.1176	1.73E-05	0.00024389
i1_HQ_YHS_c17332/f2p0/1526	240.9868373	1020.179579	-2.0818	1.74E-05	0.0002444
i1_LQ_YHS_c14197/f1p0/1360	514.4728579	2.135476809	7.9124	1.74E-05	0.0002444
i4_LQ_YHS_c4208/f1p8/4743	237.3773518	1007.920151	-2.0861	1.74E-05	0.00024475
i3_LQ_YHS_c20781/f1p0/3011	14.42741457	102.6372853	-2.8307	1.75E-05	0.00024527
i1_LQ_YHS_c10351/f1p7/1957	37.04779671	1.079981815	5.1003	1.75E-05	0.00024581
i2_LQ_YHS_c17841/f1p0/2354	0	22.49643997	-Inf	1.75E-05	0.00024581
i1_LQ_YHS_c9157/f1p0/1737	14.32582392	146.0213874	-3.3495	1.75E-05	0.00024609
i5_LQ_YHS_c3929/f1p1/5341	12.36950894	97.9768752	-2.9857	1.77E-05	0.00024824
i4_HQ_YHS_c2221/f2p3/5020	5.583229988	66.40440195	-3.5721	1.77E-05	0.0002487
i3_LQ_YHS_c8511/f1p0/3340	40.68301354	1.50450994	4.7571	1.78E-05	0.00024896
i3_LQ_YHS_c18351/f1p0/3319	375.2579956	1873.63904	-2.3199	1.78E-05	0.00024941
i3_LQ_YHS_c9854/f1p0/3294	59.4909478	262.0047938	-2.1389	1.78E-05	0.00024978
i3_LQ_YHS_c3974/f1p1/3720	19.82558679	125.4853857	-2.6621	1.79E-05	0.00025075
i2_LQ_YHS_c36925/f1p10/2433	245.1293911	1059.111621	-2.1112	1.79E-05	0.00025137
i1_LQ_YHS_c18926/f1p0/1438	8.051483366	418.5469615	-5.7	1.81E-05	0.00025302
i2_HQ_YHS_c13650/f7p18/3019	61.90971691	281.4360923	-2.1846	1.81E-05	0.00025394
i5_LQ_YHS_c2597/f1p0/5902	31.74150473	172.4058056	-2.4414	1.81E-05	0.00025394
i1_LQ_YHS_c12939/f1p4/1744	42.69351244	210.8233717	-2.3039	1.82E-05	0.00025396
i2_LQ_YHS_c37913/f1p3/2536	339.6154693	1.858382173	7.5137	1.82E-05	0.00025414
i3_LQ_YHS_c15048/f1p0/3037	7.626023095	78.55833267	-3.3648	1.82E-05	0.0002545
i5_LQ_YHS_c178/f1p0/6046	3.529946197	70.54889178	-4.3209	1.83E-05	0.00025585
i2_LQ_YHS_c10706/f1p0/2738	5.625201099	134.768017	-4.5824	1.84E-05	0.00025771
i2_LQ_YHS_c22342/f1p1/2172	0	121.5612712	-Inf	1.85E-05	0.000258
i1_HQ_YHS_c40516/f24p0/1855	698.1857952	8470.845455	-3.6008	1.85E-05	0.0002586
i2_HQ_YHS_c7561/f2p8/2192	45.18433288	240.6522375	-2.4131	1.85E-05	0.00025869
i3_LQ_YHS_c3819/f1p2/2958	376.5089899	2.572248344	7.1935	1.86E-05	0.00026028
i2_LQ_YHS_c36913/f1p1/2664	109.5953705	3.684497252	4.8946	1.87E-05	0.0002607
i2_LQ_YHS_c8376/f1p4/2400	1390.993774	1.769361165	9.6187	1.87E-05	0.00026086
i1_LQ_YHS_c28675/f1p1/1299	83.23029188	350.1271958	-2.0727	1.87E-05	0.00026112
i3_LQ_YHS_c7429/f1p9/3079	42.33689862	1.858382173	4.5098	1.88E-05	0.0002614
i4_LQ_YHS_c6673/f1p1/4828	147.4010299	25.98449055	2.504	1.88E-05	0.0002614
i1_LQ_YHS_c7166/f1p1/1871	234.1107761	49.42252489	2.244	1.88E-05	0.00026193
i2_HQ_YHS_c6211/f3p2/2886	160.346879	658.4479367	-2.0379	1.88E-05	0.00026228

i2_LQ_YHS_c12658/f1p1/2601	6.489614971	66.22963752	-3.3513	1.89E-05	0.00026299
i1_LQ_YHS_c10985/f1p1/1791	0	22.03245726	-Inf	1.89E-05	0.0002634
i2_HQ_YHS_c45532/f3p4/2161	184.4108239	767.129728	-2.0565	1.90E-05	0.000264
i4_HQ_YHS_c14189/f31p0/4552	276.3488138	1439.645476	-2.3812	1.90E-05	0.00026453
i2_HQ_YHS_c2630/f11p0/2148	429.4827987	101.117768	2.0866	1.90E-05	0.00026467
i5_LQ_YHS_c4512/f1p0/5234	0	22.46970181	-Inf	1.91E-05	0.00026576
i3_LQ_YHS_c5166/f1p0/4030	6.887869602	67.23789765	-3.2871	1.91E-05	0.0002661
i1_HQ_YHS_c22072/f2p0/1904	55.49864204	254.4409705	-2.1968	1.92E-05	0.00026623
i3_LQ_YHS_c10677/f1p0/3134	0.771711468	35.38335407	-5.5189	1.92E-05	0.00026655
i1_LQ_YHS_c32935/f1p0/1907	120.8958924	488.8729915	-2.0157	1.92E-05	0.00026666
i4_LQ_YHS_c10231/f1p11/4739	605.9646205	8.161296839	6.2143	1.92E-05	0.00026675
i3_HQ_YHS_c960/f6p0/3595	982.3683155	227.8783966	2.108	1.92E-05	0.00026687
i2_LQ_YHS_c29110/f1p1/2055	133.4432356	17.52611494	2.9286	1.92E-05	0.00026702
i2_LQ_YHS_c6254/f1p1/2478	10.18028787	86.27290545	-3.0831	1.92E-05	0.00026702
i1_LQ_YHS_c13794/f1p1/1241	290.9064751	64.62054603	2.1705	1.93E-05	0.00026738
i2_LQ_YHS_c20387/f1p1/2115	160.8694852	2375.898295	-3.8845	1.93E-05	0.00026738
i2_LQ_YHS_c2935/f1p6/2604	87.58755879	363.6411657	-2.0537	1.93E-05	0.00026754
i2_LQ_YHS_c39764/f1p3/2848	64.19163201	337.3290274	-2.3937	1.93E-05	0.00026783
i2_LQ_YHS_c6557/f1p0/2776	0	22.36231568	-Inf	1.95E-05	0.00026949
i1_LQ_YHS_c13735/f1p0/1234	1013.285738	1.176783094	9.75	1.95E-05	0.00027033
i5_LQ_YHS_c1184/f1p4/5469	811.8941503	115.9185524	2.8082	1.96E-05	0.00027114
i2_LQ_YHS_c28320/f1p2/2245	0	22.29446437	-Inf	1.97E-05	0.0002723
i1_LQ_YHS_c7628/f1p20/1827	489.2094385	1.439975754	8.4083	1.97E-05	0.00027256
i4_LQ_YHS_c9881/f1p5/4856	2.451071687	46.25315512	-4.2381	1.97E-05	0.00027256
i1_LQ_YHS_c6008/f1p0/1409	233.788905	993.2562974	-2.087	1.97E-05	0.00027275
i1_LQ_YHS_c21569/f1p2/1702	34.15428529	263.423085	-2.9472	1.97E-05	0.00027291
i1_LQ_YHS_c17571/f1p0/1940	94.43057433	12.31483272	2.9389	1.98E-05	0.00027323
i2_LQ_YHS_c35559/f1p1/2390	97.05573798	416.2527978	-2.1006	1.98E-05	0.00027392
i1_LQ_YHS_c17367/f1p0/1651	4246.786119	28.99011308	7.1947	1.99E-05	0.00027506
i2_LQ_YHS_c41249/f1p0/2645	176.8342851	715.9809681	-2.0175	1.99E-05	0.00027512
i2_LQ_YHS_c21069/f1p0/2137	42.94740828	209.6628221	-2.2874	2.00E-05	0.00027574
i2_LQ_YHS_c2929/f1p3/2945	302.5788275	7.722866737	5.292	2.01E-05	0.00027675
i1_HQ_YHS_c10023/f2p4/1501	127.9230155	522.7196883	-2.0308	2.02E-05	0.00027885
i3_LQ_YHS_c8965/f1p0/3810	139.2833102	555.9649599	-1.997	2.03E-05	0.00027947
i1_LQ_YHS_c34817/f1p0/1407	32.60291596	169.3359219	-2.3768	2.03E-05	0.00028013
i1_LQ_YHS_c7045/f1p4/1652	64.06873807	283.9437658	-2.1479	2.03E-05	0.00028013
i1_HQ_YHS_c37817/f2p0/1202	0.257237156	26.26774643	-6.674	2.03E-05	0.00028027
i1_LQ_YHS_c2559/f2p0/1621	24.94379325	304.2317487	-3.6084	2.04E-05	0.00028123
i2_HQ_YHS_c13127/f4p0/2168	50.29521285	235.7253561	-2.2286	2.06E-05	0.00028338
i2_HQ_YHS_c3704/f4p0/2559	0	43.3575233	-Inf	2.06E-05	0.00028338
i2_LQ_YHS_c20090/f1p6/2326	1.074261052	220.548943	-7.6816	2.07E-05	0.00028451
i5_LQ_YHS_c841/f1p0/5830	52.82721574	245.4062504	-2.2158	2.07E-05	0.0002847
i2_LQ_YHS_c10031/f1p2/2285	607.2447099	151.2866992	2.005	2.07E-05	0.00028504
i3_HQ_YHS_c1715/f5p1/3930	129.3546216	696.7314325	-2.4293	2.07E-05	0.0002854
i1_LQ_YHS_c13644/f1p0/1362	178.8592024	735.8443799	-2.0406	2.08E-05	0.00028604
i3_LQ_YHS_c4177/f1p0/3769	17.61249302	121.4877366	-2.7861	2.08E-05	0.00028604
i2_LQ_YHS_c2985/f1p0/2157	231.6194769	41.33180372	2.4864	2.10E-05	0.00028803
i1_LQ_YHS_c28720/f1p0/1061	672.1731179	5715.433401	-3.088	2.10E-05	0.00028805
i2_LQ_YHS_c8685/f1p0/2316	0	21.91223495	-Inf	2.10E-05	0.00028805
i3_LQ_YHS_c3259/f1p20/3622	1.90638909	42.22031456	-4.469	2.10E-05	0.00028874
i1_LQ_YHS_c39262/f1p0/1193	654.9681075	4690.091314	-2.8401	2.11E-05	0.00029018
i3_LQ_YHS_c10500/f1p0/3257	38.77752443	266.3315135	-2.7799	2.13E-05	0.00029227
i3_LQ_YHS_c3434/f1p17/3088	77.50182352	8.3861007	3.2082	2.13E-05	0.0002927
i2_LQ_YHS_c44918/f1p0/2048	420.9933275	101.181076	2.0569	2.14E-05	0.00029355
i2_LQ_YHS_c11853/f1p0/2724	303.9106142	70.12831424	2.1156	2.15E-05	0.00029471
i1_LQ_YHS_c21527/f1p0/2027	133.0903508	767.6424746	-2.528	2.16E-05	0.00029635
i0_LQ_YHS_c974/f1p0/472	345.1894855	0	Inf	2.17E-05	0.00029698
i3_LQ_YHS_c15074/f1p0/3075	666.3459778	164.1073799	2.0216	2.17E-05	0.00029727
i1_LQ_YHS_c33854/f1p0/1987	45.02836445	214.0814092	-2.2493	2.18E-05	0.00029922
i3_HQ_YHS_c1249/f8p0/3233	213.4140054	898.8854438	-2.0745	2.19E-05	0.0002997
i2_LQ_YHS_c19235/f1p0/2118	383.4471264	16.85790527	4.5075	2.19E-05	0.00030004
i5_LQ_YHS_c1867/f1p1/5904	16.93014264	128.6513977	-2.9258	2.19E-05	0.00030009
i1_LQ_YHS_c9023/f1p1/1741	38.70201209	199.6642733	-2.3671	2.20E-05	0.00030104
i3_LQ_YHS_c5825/f1p1/3358	78.13491698	679.2249078	-3.1199	2.20E-05	0.00030126
i0_LQ_YHS_c2717/f1p0/799	341.6924009	81.12181517	2.0745	2.21E-05	0.00030191
i1_LQ_YHS_c7355/f1p4/1900	280.2330952	3.382915794	6.3722	2.21E-05	0.00030191
i2_LQ_YHS_c18940/f1p0/2701	306.7464053	71.22504215	2.1066	2.21E-05	0.00030191
i3_LQ_YHS_c7205/f1p0/3863	2.178730389	44.10037898	-4.3392	2.21E-05	0.00030191
i1_HQ_YHS_c4972/f4p2/1586	83.07936177	826.9116792	-3.3152	2.23E-05	0.00030466
i1_LQ_YHS_c26486/f1p0/1657	13.61835382	161.0752203	-3.5641	2.23E-05	0.00030485
i2_LQ_YHS_c38317/f1p0/2717	0	22.02068686	-Inf	2.24E-05	0.0003055
i1_LQ_YHS_c18125/f1p0/1729	27.68947649	169.0060623	-2.6097	2.26E-05	0.00030827
i0_HQ_YHS_c252/f3p0/738	368.7006064	20151.52493	-5.7723	2.26E-05	0.0003089
i1_HQ_YHS_c17012/f2p0/1531	149.7240161	586.5267981	-1.9699	2.27E-05	0.00031001
i1_LQ_YHS_c41170/f3p1/1977	1561.799153	0	Inf	2.27E-05	0.00031008
i2_HQ_YHS_c17759/f2p5/2153	227.9588795	49.26770392	2.2101	2.28E-05	0.00031127
i2_LQ_YHS_c40311/f1p2/2636	165.198345	10.12094343	4.0288	2.29E-05	0.000312
i4_LQ_YHS_c5544/f1p2/4206	538.2478876	2898.623253	-2.429	2.29E-05	0.00031223
i2_LQ_YHS_c6595/f1p15/2169	610.4388237	13330.61883	-4.4488	2.31E-05	0.00031515
i1_LQ_YHS_c12798/f1p2/2001	15.13201846	113.802467	-2.9109	2.32E-05	0.00031589
i4_LQ_YHS_c10822/f1p0/4862	42.39765386	200.0510845	-2.2383	2.33E-05	0.00031743
i2_HQ_YHS_c9503/f2p2/2292	239.7529817	13.86223414	4.1123	2.34E-05	0.00031783
i1_LQ_YHS_c3786/f1p1/1879	51.80543323	234.4816281	-2.1783	2.36E-05	0.00032053
i2_LQ_YHS_c20815/f1p15/2324	394.2683991	1780.549587	-2.1751	2.36E-05	0.00032053
i4_LQ_YHS_c13572/f1p0/4129	3.976854572	96.86553277	-4.6063	2.36E-05	0.00032056
i3_LQ_YHS_c17821/f1p1/3116	16.01360565	110.3116514	-2.7842	2.36E-05	0.00032086
i2_HQ_YHS_c6339/f5p4/2455	279.0158696	1218.787763	-2.127	2.37E-05	0.0003214
i4_LQ_YHS_c8369/f1p0/4476	409.19704	2004.470697	-2.2924	2.37E-05	0.00032141
i5_LQ_YHS_c898/f1p0/5932	25.71344391	151.0690828	-2.5546	2.37E-05	0.00032175

i2_LQ_YHS_c54701/f1p4/2276	17.17516698	103.8586986	-2.5962	2.37E-05	0.00032221
i2_LQ_YHS_c11663/f1p2/2617	3.552199708	79.71130081	-4.488	2.38E-05	0.00032356
i4_LQ_YHS_c5765/f1p0/4417	30.61431515	182.2959025	-2.574	2.39E-05	0.00032383
i1_HQ_YHS_c6957/f2p0/1580	85.53613357	9.517780513	3.1678	2.40E-05	0.00032507
i1_HQ_YHS_c31601/f2p0/1900	564.3245456	1.890649266	8.2215	2.40E-05	0.00032511
i4_LQ_YHS_c3995/f1p5/4867	41.55168545	269.5337431	-2.6975	2.40E-05	0.00032511
i3_HQ_YHS_c16447/f4p0/3296	75.45300837	323.5829464	-2.1005	2.40E-05	0.00032585
i2_LQ_YHS_c22249/f1p0/2870	0	51.29401628	-Inf	2.41E-05	0.00032646
i3_LQ_YHS_c8498/f1p1/4001	173.3322255	723.6891412	-2.0618	2.41E-05	0.00032646
i2_HQ_YHS_c46072/f4p3/2155	3300.967632	146.5730919	4.4932	2.42E-05	0.00032765
i3_LQ_YHS_c7593/f1p3/3368	364.607403	86.86153406	2.0696	2.42E-05	0.00032814
i4_LQ_YHS_c13200/f1p7/4215	0.719249673	29.33458602	-5.35	2.43E-05	0.00032957
i3_HQ_YHS_c2686/f2p3/3595	0	36.96392906	-Inf	2.44E-05	0.00033061
i2_HQ_YHS_c57736/f2p0/2010	327.957625	1396.338427	-2.0901	2.44E-05	0.00033072
i2_LQ_YHS_c5183/f1p1/2990	13.07954007	95.46189333	-2.8676	2.46E-05	0.00033262
i5_LQ_YHS_c1351/f1p2/5598	124.4253719	493.8520429	-1.9888	2.46E-05	0.00033295
i4_LQ_YHS_c12675/f1p1/4684	14.21928951	104.858466	-2.8825	2.47E-05	0.00033359
i2_LQ_YHS_c40965/f1p1/2650	86.56909248	455.4793838	-2.3955	2.47E-05	0.00033368
i2_LQ_YHS_c44474/f1p3/2084	2.255523243	41.45913331	-4.2002	2.47E-05	0.00033403
i2_LQ_YHS_c5974/f1p2/2480	32.83675552	171.0046509	-2.3807	2.48E-05	0.00033438
i1_LQ_YHS_c34483/f1p0/1946	131.5138366	829.2085792	-2.6565	2.48E-05	0.00033508
i4_HQ_YHS_c2434/f2p0/4485	108.337976	427.7510056	-1.9812	2.49E-05	0.00033574
i3_LQ_YHS_c7074/f1p0/3251	443.8749571	109.4068675	2.0205	2.49E-05	0.00033601
i3_LQ_YHS_c4860/f1p3/3315	690.4727435	172.2434378	2.0031	2.49E-05	0.00033627
i1_LQ_YHS_c12278/f1p1/1499	74.88828625	479.8737001	-2.6798	2.50E-05	0.00033761
i1_LQ_YHS_c13665/f1p3/1279	128.6713629	505.3654135	-1.9736	2.51E-05	0.00033857
i1_LQ_YHS_c27757/f1p0/1145	78.13987749	326.1678705	-2.0615	2.51E-05	0.00033885
i1_HQ_YHS_c16849/f3p0/1837	228.1028469	4.481262726	5.6696	2.53E-05	0.00034026
i2_LQ_YHS_c49412/f1p1/2141	182.0472225	29.39580307	2.6306	2.53E-05	0.00034116
i1_LQ_YHS_c2512/f2p13/1858	20.08697905	117.7702201	-2.5516	2.53E-05	0.00034123
i1_LQ_YHS_c12503/f1p1/1515	0	21.74971393	-Inf	2.55E-05	0.00034287
i4_HQ_YHS_c2367/f2p0/4775	55.14809093	373.8344989	-2.761	2.55E-05	0.00034321
i3_LQ_YHS_c9748/f1p0/3360	53.97215669	336.1345522	-2.6388	2.55E-05	0.00034328
i1_LQ_YHS_c43712/f1p0/1035	565.2483874	14635.69246	-4.6945	2.56E-05	0.00034411
i3_LQ_YHS_c12683/f1p0/3155	1.993672628	41.2822373	-4.372	2.57E-05	0.00034541
i1_LQ_YHS_c21738/f1p0/1792	633.3741644	3840.252349	-2.6001	2.57E-05	0.00034562
i4_LQ_YHS_c3121/f1p3/4940	0	21.68071661	-Inf	2.58E-05	0.00034623
i1_LQ_YHS_c6601/f1p0/1999	29.77885417	156.0044955	-2.3892	2.58E-05	0.00034667
i2_LQ_YHS_c39007/f1p0/2730	113.655667	460.9034227	-2.0198	2.59E-05	0.00034766
i1_LQ_YHS_c8270/f1p0/1475	47.49026549	222.0332243	-2.2251	2.60E-05	0.00034857
i3_LQ_YHS_c6041/f1p1/3257	3.034384079	48.64736144	-4.0029	2.61E-05	0.00034999
i1_LQ_YHS_c35229/f1p641/1563	282.2954109	2156.6417	-2.9335	2.62E-05	0.0003513
i0_LQ_YHS_c1319/f1p0/588	296.7659609	1276.756533	-2.1051	2.62E-05	0.00035214
i3_LQ_YHS_c6505/f1p0/3456	242.4688648	45.58439222	2.4112	2.63E-05	0.00035321
i2_LQ_YHS_c9628/f1p1/2098	18.51841964	113.8026658	-2.6195	2.63E-05	0.00035335
i2_LQ_YHS_c11044/f1p11/2767	57.64751119	1194.017608	-4.3724	2.64E-05	0.00035424
i1_LQ_YHS_c21217/f1p0/1717	15.80676111	331.7673088	-4.3916	2.64E-05	0.00035435
i0_LQ_YHS_c596/f1p0/759	135.0757384	780.459581	-2.5306	2.65E-05	0.0003555
i4_LQ_YHS_c13790/f1p0/4154	38.79250888	438.9804771	-3.5003	2.66E-05	0.00035579
i2_LQ_YHS_c51062/f1p9/2368	407.998687	1960.026988	-2.2642	2.66E-05	0.00035622
i1_HQ_YHS_c25582/f3p0/1524	2836.792108	6.161602883	8.8467	2.66E-05	0.00035665
i3_LQ_YHS_c14833/f1p1/3065	133.6500885	18.49033758	2.8536	2.67E-05	0.00035684
i2_LQ_YHS_c24860/f1p6/2944	1.634047791	53.64327864	-5.0369	2.67E-05	0.00035727
i1_LQ_YHS_c38693/f1p0/1114	56.20679786	1154.093573	-4.3599	2.68E-05	0.00035896
i1_HQ_YHS_c7241/f2p0/1911	126.2111756	504.7018658	-1.9996	2.68E-05	0.00035903
i4_LQ_YHS_c4663/f1p0/4537	257.2744573	1062.503718	-2.0461	2.69E-05	0.00035903
i3_LQ_YHS_c8545/f1p0/3532	377.4966687	91.96377159	2.0373	2.69E-05	0.00035923
i2_LQ_YHS_c39950/f1p1/2520	16.05558513	118.020537	-2.8779	2.69E-05	0.00036005
i5_LQ_YHS_c3421/f1p0/5368	64.7590516	281.197699	-2.1184	2.71E-05	0.00036192
i1_HQ_YHS_c2060/f4p3/1847	443.4420675	4.038369485	6.7788	2.71E-05	0.00036195
i4_LQ_YHS_c9091/f1p1/4652	309.6280139	0	Inf	2.71E-05	0.00036195
i2_LQ_YHS_c33474/f1p168/2277	3880.492409	0.359993938	13.396	2.71E-05	0.00036223
i1_LQ_YHS_c13541/f1p1/1266	68.26385296	480.9154844	-2.8166	2.71E-05	0.00036225
i1_LQ_YHS_c4654/f1p0/1536	203.0082839	42.43674537	2.2582	2.72E-05	0.00036255
i2_LQ_YHS_c24538/f1p1/2313	994.8362222	178.433477	2.4791	2.72E-05	0.00036283
i2_LQ_YHS_c36729/f1p2/2428	103.1900197	14.90098304	2.7918	2.72E-05	0.00036293
i3_LQ_YHS_c10257/f1p0/3388	83.92466959	10.23836117	3.0351	2.74E-05	0.00036483
i2_LQ_YHS_c55718/f1p5/2338	33.65664563	177.853856	-2.4017	2.74E-05	0.00036516
i0_LQ_YHS_c2171/f1p0/569	176.2891274	704.1386862	-1.9979	2.74E-05	0.00036518
i3_LQ_YHS_c5875/f1p0/3109	87.5023528	354.1586904	-2.017	2.74E-05	0.00036555
i1_LQ_YHS_c24277/f1p399/1787	0	21.27514637	-Inf	2.76E-05	0.00036698
i1_LQ_YHS_c23625/f1p74/1988	89.95895467	370.9186472	-2.0438	2.78E-05	0.00036993
i2_HQ_YHS_c45474/f3p3/2098	195.1510266	798.3303985	-2.0324	2.78E-05	0.00036998
i3_LQ_YHS_c4035/f1p3/3862	31.93533104	163.4880241	-2.356	2.78E-05	0.00037046
i2_HQ_YHS_c24851/f4p8/2969	65.30151184	289.0582083	-2.1462	2.79E-05	0.00037119
i2_LQ_YHS_c5755/f1p3/2154	24.86112317	793.56128	-4.9964	2.80E-05	0.00037235
i3_LQ_YHS_c5392/f1p3/3577	45.91342939	2.507714158	4.1945	2.82E-05	0.00037487
i1_LQ_YHS_c23214/f1p2/1697	769.1006663	6937.752222	-3.1732	2.82E-05	0.0003751
i1_LQ_YHS_c4507/f2p0/1859	58.62398127	258.6691117	-2.1415	2.82E-05	0.0003751
i3_LQ_YHS_c8065/f1p18/3256	281.7015695	1224.939938	-2.1205	2.83E-05	0.00037532
i2_LQ_YHS_c3687/f4p1/2819	47.2881541	2.830977864	4.0621	2.83E-05	0.00037627
i0_LQ_YHS_c2099/f1p0/805	45.79325684	215.6985947	-2.2358	2.85E-05	0.00037812
i2_LQ_YHS_c54991/f1p0/2109	2.682714017	45.12747724	-4.0722	2.85E-05	0.0003788
i3_LQ_YHS_c7680/f1p0/3646	210.2029527	45.02017134	2.2231	2.87E-05	0.00038094
i3_LQ_YHS_c8729/f1p0/3897	7.622215044	74.18390283	-3.2828	2.87E-05	0.00038108
i1_LQ_YHS_c35856/f1p0/1708	49575.58955	4.816769843	13.329	2.88E-05	0.0003823
i2_LQ_YHS_c25973/f1p6/2597	30.50222544	458.4992205	-3.9099	2.89E-05	0.00038257
i2_LQ_YHS_c3484/f1p1/2601	678.660069	4.945838214	7.1003	2.90E-05	0.00038378
i2_LQ_YHS_c50010/f1p2/2501	131.7828785	526.0307604	-1.997	2.90E-05	0.00038378

i1_LQ_YHS_c32393/f1p0/1575	74.16206432	769.9687713	-3.376	2.90E-05	0.00038447
i2_LQ_YHS_c13694/f1p0/2268	3.953795655	65.41743126	-4.0484	2.91E-05	0.00038589
i2_LQ_YHS_c53913/f1p1/2630	33.05536288	171.1497935	-2.3723	2.92E-05	0.00038637
i1_LQ_YHS_c26556/f1p4/1545	15.33426628	119.6098433	-2.9635	2.92E-05	0.00038658
i2_LQ_YHS_c4269/f2p0/2314	257.3773285	57.49191593	2.1625	2.92E-05	0.00038696
i2_LQ_YHS_c23550/f1p11/2712	455.3476778	2390.023073	-2.392	2.93E-05	0.00038765
i3_LQ_YHS_c10768/f1p1/3833	1.778406583	38.54419035	-4.4379	2.94E-05	0.00038852
i1_LQ_YHS_c3383/f1p5/1455	303.9308978	1261.908668	-2.0538	2.94E-05	0.0003891
i1_LQ_YHS_c27537/f1p0/1203	2696.733187	35.09283047	6.2639	2.96E-05	0.00039073
i1_LQ_YHS_c20097/f1p0/1727	20.15119529	121.4147096	-2.591	2.96E-05	0.00039123
i4_LQ_YHS_c7344/f1p0/4439	8.12492653	81.321379	-3.3232	2.98E-05	0.00039305
i1_LQ_YHS_c32333/f1p0/1350	84.46158289	378.4322778	-2.1637	2.98E-05	0.00039344
i2_LQ_YHS_c35909/f1p6/2932	113.9517362	456.615523	-2.0026	2.98E-05	0.00039361
i3_LQ_YHS_c19614/f1p2/3780	16.17607122	112.1710993	-2.7938	2.98E-05	0.00039382
i0_HQ_YHS_c502/f2p0/924	615.8305897	3843.251689	-2.6417	2.98E-05	0.00039388
i2_LQ_YHS_c64764/f1p0/2025	8.978044311	77.15125633	-3.1032	2.99E-05	0.00039402
i2_LQ_YHS_c10824/f1p2/3002	37.79263283	186.6796514	-2.3044	3.00E-05	0.00039514
i2_LQ_YHS_c36643/f1p4/2383	55.56111104	253.406292	-2.1893	3.02E-05	0.00039795
i0_LQ_YHS_c2572/f1p0/565	0	21.22838453	-Inf	3.02E-05	0.00039828
i2_LQ_YHS_c41873/f1p8/2936	41.28888462	717.5279877	-4.1192	3.02E-05	0.00039828
i2_LQ_YHS_c38600/f1p99/1996	839.2133219	0	Inf	3.02E-05	0.00039845
i3_HQ_YHS_c10865/f2p0/3815	333.6888521	81.7872598	2.0286	3.03E-05	0.00039871
i1_LQ_YHS_c43940/f1p0/1041	700.2970075	4830.346269	-2.7861	3.04E-05	0.00040074
i2_LQ_YHS_c36271/f1p0/2167	122.4499425	475.2080352	-1.9564	3.04E-05	0.00040074
i1_LQ_YHS_c3160/f1p0/1976	12133.28809	90.46388523	7.0674	3.06E-05	0.00040311
i1_LQ_YHS_c35337/f1p1/1654	188.3288823	0	Inf	3.06E-05	0.00040311
i4_LQ_YHS_c8442/f1p2/4392	8.111094529	74.2285331	-3.194	3.07E-05	0.00040393
i5_LQ_YHS_c2505/f1p2/5851	228.0650417	939.9997102	-2.0432	3.07E-05	0.00040423
i2_LQ_YHS_c20696/f1p2/2645	230.2740222	50.25155745	2.1961	3.08E-05	0.0004051
i3_LQ_YHS_c6446/f1p1/3538	92.23323692	382.8926846	-2.0536	3.09E-05	0.00040639
i1_LQ_YHS_c11539/f1p3/1824	52.07316107	231.1927486	-2.1505	3.10E-05	0.00040661
i2_LQ_YHS_c23309/f1p1/3018	48.85845238	2.489349042	4.2948	3.10E-05	0.00040661
i3_LQ_YHS_c17961/f1p16/3299	58.99456353	619.6972011	-3.3929	3.10E-05	0.00040661
i1_LQ_YHS_c35718/f1p0/1664	0	21.04256224	-Inf	3.12E-05	0.00040921
i1_HQ_YHS_c2443/f2p1/1406	583.274682	3671.780573	-2.6542	3.16E-05	0.00041535
i1_LQ_YHS_c37839/f2p0/1354	487.8581869	2444.683458	-2.3251	3.17E-05	0.00041606
i2_LQ_YHS_c36875/f1p1/3099	199.0026703	785.6841603	-1.9812	3.18E-05	0.00041675
i1_HQ_YHS_c18624/f3p2/1634	193.6751361	1593.163296	-3.0402	3.19E-05	0.00041786
i1_LQ_YHS_c43578/f1p0/1109	334.2503381	1387.358274	-2.0533	3.19E-05	0.00041791
i2_LQ_YHS_c23593/f1p2/2348	184.4752843	899.7266286	-2.2861	3.19E-05	0.00041813
i1_LQ_YHS_c22750/f1p3/1761	1307.760041	0	Inf	3.19E-05	0.00041826
i2_LQ_YHS_c41493/f1p1/2560	308.7546542	71.93278662	2.1017	3.20E-05	0.00041911
i4_LQ_YHS_c8821/f1p16/4703	3.540436881	51.11834537	-3.8518	3.20E-05	0.00041911
i4_LQ_YHS_c11093/f1p2/4888	10.74389103	160.0404197	-3.8968	3.21E-05	0.00041979
i2_LQ_YHS_c2855/f2p0/2658	212.9036455	842.1282884	-1.9838	3.24E-05	0.00042398
i1_LQ_YHS_c28665/f1p0/1208	734.8595977	5180.581167	-2.8176	3.25E-05	0.00042556
i2_LQ_YHS_c59488/f1p1/2029	1130.127714	217.702716	2.3761	3.27E-05	0.00042776
i4_LQ_YHS_c5679/f1p0/4422	99.92143221	402.4602389	-2.01	3.27E-05	0.00042805
i2_LQ_YHS_c34157/f1p5/2120	207.2474954	0	Inf	3.28E-05	0.00042828
i1_LQ_YHS_c13070/f1p0/1546	17.13652044	900.5367387	-5.7156	3.29E-05	0.00042955
i1_LQ_YHS_c20460/f1p1/1885	23.47094727	136.8280957	-2.5434	3.29E-05	0.00042955
i1_LQ_YHS_c28868/f1p0/1312	98.68469721	399.0824197	-2.0158	3.29E-05	0.00042957
i3_LQ_YHS_c14011/f1p0/3004	35.73761017	185.5157441	-2.376	3.29E-05	0.0004303
i1_LQ_YHS_c25160/f1p2/1817	0	20.70598933	-Inf	3.30E-05	0.00043067
i2_LQ_YHS_c35759/f1p0/2430	0	40.70237566	-Inf	3.31E-05	0.00043214
i2_HQ_YHS_c6023/f11p6/2316	70.86772169	295.5632579	-2.0603	3.32E-05	0.00043287
i3_LQ_YHS_c8304/f1p1/3648	52.00467841	0	Inf	3.33E-05	0.00043424
i1_LQ_YHS_c32245/f1p0/1686	6.459406686	98.99354793	-3.9379	3.34E-05	0.00043571
i1_LQ_YHS_c33761/f1p0/1818	311.0410284	1324.342286	-2.0901	3.35E-05	0.00043643
i2_HQ_YHS_c60279/f4p3/2093	87.57050517	358.5538169	-2.0337	3.35E-05	0.00043643
i2_LQ_YHS_c14110/f1p1/2014	5.9705272	61.75895964	-3.3707	3.35E-05	0.00043661
i2_LQ_YHS_c33775/f1p7/2436	28.9449705	625.9948681	-4.4348	3.35E-05	0.00043661
i6_LQ_YHS_c511/f1p0/6696	11.15931898	83.55271106	-2.9044	3.35E-05	0.00043661
i1_LQ_YHS_c10424/f1p0/1774	1.448990031	35.73382895	-4.6242	3.35E-05	0.00043672
i1_LQ_YHS_c44039/f1p0/1101	20.5791831	333.5771843	-4.0188	3.37E-05	0.0004381
i5_LQ_YHS_c4303/f1p0/5763	3.602125593	49.53867685	-3.7816	3.37E-05	0.00043827
i6_LQ_YHS_c850/f1p0/6284	14.17016904	101.2050898	-2.8364	3.37E-05	0.00043877
i2_LQ_YHS_c23249/f1p0/2510	95.72646214	13.41483822	2.8351	3.37E-05	0.00043901
i3_LQ_YHS_c3940/f1p7/3274	1583.225959	342.0365944	2.2106	3.38E-05	0.00043983
i1_LQ_YHS_c31649/f2p0/1405	54.13391616	936.9535459	-4.1134	3.38E-05	0.00044004
i2_HQ_YHS_c32406/f3p0/2723	153.484299	587.0461579	-1.9354	3.39E-05	0.00044012
i0_LQ_YHS_c1363/f1p4/990	339.3915867	81.13117379	2.0646	3.40E-05	0.0004419
i2_LQ_YHS_c25901/f1p0/2761	47.94411262	0	Inf	3.40E-05	0.0004421
i0_LQ_YHS_c779/f1p0/929	765.7386129	6009.651529	-2.9724	3.41E-05	0.00044252
i1_LQ_YHS_c25662/f1p0/1798	127.0988339	499.300891	-1.974	3.41E-05	0.00044309
i2_HQ_YHS_c29628/f16p5/2305	124.8195983	955.7051188	-2.9367	3.41E-05	0.00044309
i3_LQ_YHS_c13086/f1p0/3680	17.0937523	108.3271252	-2.6639	3.41E-05	0.00044309
i1_LQ_YHS_c14095/f1p1/1212	614.6073445	3.729007756	7.3647	3.42E-05	0.00044418
i2_LQ_YHS_c46030/f11p13/2105	6911.678606	14.11373662	8.9358	3.42E-05	0.00044418
i2_LQ_YHS_c55177/f1p0/2137	112.9890646	457.8012324	-2.0185	3.43E-05	0.00044517
i2_LQ_YHS_c53078/f1p0/2633	81.24897687	334.9632931	-2.0436	3.44E-05	0.00044576
i3_LQ_YHS_c12531/f1p3/3618	512.3062362	98.21613427	2.383	3.44E-05	0.00044576
i3_LQ_YHS_c10971/f1p6/3683	66.33650762	274.6233384	-2.0496	3.45E-05	0.00044636
i2_LQ_YHS_c64715/f1p0/2015	0	37.33170327	-Inf	3.45E-05	0.00044709
i2_LQ_YHS_c15037/f1p4/1832	3.624379103	54.27748355	-3.9045	3.46E-05	0.00044752
i2_LQ_YHS_c29784/f8p2/2459	396.8881547	101.2161081	1.9713	3.46E-05	0.00044833
i2_LQ_YHS_c53919/f1p2/2537	304.7621534	1297.753636	-2.0903	3.47E-05	0.00044852
i1_LQ_YHS_c6848/f1p0/1535	129.4843849	2.919998872	5.4707	3.47E-05	0.00044866
i2_LQ_YHS_c45082/f1p4/2048	113.802892	18.32236785	2.6349	3.47E-05	0.00044918

i3_LQ_YHS_c3816/f1p2/3807	16.41520997	262.0925539	-3.997	3.48E-05	0.00045006
i2_LQ_YHS_c11560/f1p4/2877	34.12467181	176.861748	-2.3737	3.49E-05	0.00045043
i4_LQ_YHS_c14010/f1p0/4109	6.647000356	116.4224627	-4.1305	3.49E-05	0.00045043
i1_HQ_YHS_c15128/f3p0/1660	299.5319147	1268.42131	-2.0823	3.49E-05	0.00045076
i1_LQ_YHS_c34874/f1p27/1445	259.4467061	1071.716294	-2.0464	3.50E-05	0.00045183
i1_LQ_YHS_c34594/f1p1/1874	269.4456307	1.569044125	7.424	3.51E-05	0.0004535
i2_LQ_YHS_c25591/f1p1/2300	25.90310676	185.1993097	-2.8379	3.54E-05	0.00045669
i1_LQ_YHS_c20519/f1p0/1755	0	181.1578565	-Inf	3.54E-05	0.00045683
i1_LQ_YHS_c36367/f1p0/1364	180.1010176	37.62388544	2.2591	3.54E-05	0.00045683
i2_LQ_YHS_c20330/f1p5/2794	0	36.59781342	-Inf	3.54E-05	0.00045683
i2_LQ_YHS_c21074/f1p3/2455	56.60502737	866.8363764	-3.9368	3.54E-05	0.00045683
i2_LQ_YHS_c40074/f1p1/2765	62.2480217	271.7138848	-2.126	3.54E-05	0.00045683
i3_LQ_YHS_c7713/f1p0/3542	579.9212069	148.1497163	1.9688	3.55E-05	0.00045729
i4_LQ_YHS_c3167/f1p0/4412	2.995754284	47.56512829	-3.9889	3.55E-05	0.00045785
i0_LQ_YHS_c3618/f1p2/252	58.92621731	4.772259339	3.6262	3.56E-05	0.00045851
i2_LQ_YHS_c27320/f1p7/2369	136.6057098	16.47283174	3.0519	3.56E-05	0.00045869
i1_LQ_YHS_c13689/f1p0/1126	465.189072	2233.981857	-2.2637	3.57E-05	0.00045957
i2_HQ_YHS_c57689/f2p0/2038	0	20.73485908	-Inf	3.57E-05	0.00045983
i0_LQ_YHS_c469/f2p0/697	429.0606342	6834.569031	-3.9936	3.59E-05	0.00046137
i2_LQ_YHS_c38327/f1p5/2196	337.8236544	1446.317966	-2.098	3.59E-05	0.00046161
i3_LQ_YHS_c9031/f1p0/3279	69.89665373	287.6612031	-2.0411	3.59E-05	0.00046161
i2_LQ_YHS_c38337/f1p3/2459	10.88490014	85.23364399	-2.9691	3.59E-05	0.00046203
i2_LQ_YHS_c4552/f1p4/2809	376.4938306	1695.955997	-2.1714	3.59E-05	0.00046212
i3_LQ_YHS_c17824/f1p0/3914	2.168239704	75.95097311	-5.1305	3.60E-05	0.00046226
i2_LQ_YHS_c52599/f1p1/2442	24.33916919	137.0552323	-2.4934	3.61E-05	0.00046354
i1_HQ_YHS_c1727/f6p2/1998	366.3748317	89.6074415	2.0316	3.62E-05	0.00046457
i3_LQ_YHS_c14674/f1p0/3024	11.45390542	121.3425544	-3.4052	3.62E-05	0.00046457
i3_LQ_YHS_c8410/f1p1/3672	55.19180092	246.9407761	-2.1616	3.62E-05	0.00046457
i1_HQ_YHS_c5409/f2p0/1764	393.4676187	4.347731215	6.4998	3.67E-05	0.00047059
i1_HQ_YHS_c17147/f2p0/1871	466.1007035	18807.39425	-5.3345	3.67E-05	0.00047066
i3_HQ_YHS_c1846/f3p0/3660	109.605538	445.0492342	-2.0216	3.68E-05	0.00047177
i2_LQ_YHS_c8351/f1p1/2503	0	46.58305309	-Inf	3.68E-05	0.00047227
i0_LQ_YHS_c1297/f1p0/962	235.0150679	0	Inf	3.68E-05	0.00047233
i2_HQ_YHS_c48275/f3p0/2792	232.4242473	0.392261031	9.2107	3.68E-05	0.00047233
i1_LQ_YHS_c39193/f1p1/1307	183.0541031	703.9840629	-1.9433	3.70E-05	0.00047361
i2_LQ_YHS_c22516/f1p4/2856	37.07799662	181.3799014	-2.2904	3.70E-05	0.00047391
i2_LQ_YHS_c44002/f1p0/2019	106.1039176	15.90418726	2.738	3.70E-05	0.00047391
i2_LQ_YHS_c52227/f1p0/2239	220.0644763	29.46033726	2.9011	3.70E-05	0.00047421
i1_LQ_YHS_c28847/f1p0/1192	120.3771553	485.9396895	-2.0132	3.71E-05	0.00047476
i1_LQ_YHS_c10212/f1p0/1893	0	26.75842776	-Inf	3.71E-05	0.00047525
i3_LQ_YHS_c19438/f1p0/3516	4121.750116	676.7550407	2.6066	3.72E-05	0.00047594
i2_HQ_YHS_c21411/f4p5/2724	265.8773215	1097.584387	-2.0455	3.74E-05	0.00047886
i2_LQ_YHS_c23125/f1p0/2890	98.03984929	387.1246566	-1.9814	3.75E-05	0.00047941
i3_LQ_YHS_c13152/f1p13/3607	38.6960068	186.4268843	-2.2684	3.76E-05	0.00048018
i2_LQ_YHS_c38814/f1p1/2124	202.7626719	810.2992184	-1.9987	3.76E-05	0.00048093
i2_LQ_YHS_c26509/f1p2/2214	104.844904	414.8526755	-1.9843	3.77E-05	0.00048209
i2_LQ_YHS_c18353/f1p0/2431	22.38953685	129.791884	-2.5353	3.77E-05	0.0004822
i3_LQ_YHS_c19781/f1p0/3193	10.12194885	78.55165773	-2.9562	3.79E-05	0.00048368
i4_HQ_YHS_c2248/f2p1/4704	0	26.34167991	-Inf	3.80E-05	0.00048521
i3_LQ_YHS_c14953/f1p18/3033	13714.40842	30.98546366	8.7899	3.81E-05	0.00048674
i2_LQ_YHS_c9414/f1p4/2479	390.6229704	98.55381251	1.9868	3.83E-05	0.00048833
i2_HQ_YHS_c2699/f4p4/2495	267.4651318	1100.060621	-2.0402	3.84E-05	0.00048947
i1_LQ_YHS_c18172/f1p0/1518	245.5454258	990.1600778	-2.0117	3.84E-05	0.00049003
i1_HQ_YHS_c7354/f3p0/1941	342.9797296	1519.885609	-2.1478	3.85E-05	0.00049084
i3_LQ_YHS_c6168/f1p0/3448	70.52494825	290.0539844	-2.0401	3.85E-05	0.0004913
i2_HQ_YHS_c62014/f206p3/2416	660.3526896	13615.70987	-4.3659	3.86E-05	0.00049155
i2_LQ_YHS_c10914/f1p10/2777	0	504.1709725	-Inf	3.86E-05	0.00049228
i4_LQ_YHS_c6315/f1p0/4667	74.94613342	321.5926122	-2.1013	3.87E-05	0.00049264
i2_HQ_YHS_c17090/f4p0/2988	210.9340514	48.46588253	2.1218	3.87E-05	0.00049304
i2_LQ_YHS_c34716/f1p3/2755	503.6205356	131.1790169	1.9408	3.88E-05	0.0004939
i4_LQ_YHS_c4010/f1p5/4852	16.49198607	110.6099157	-2.7456	3.89E-05	0.00049457
i2_LQ_YHS_c7195/f1p2/2316	22.83311228	0	Inf	3.89E-05	0.00049552
i3_LQ_YHS_c16782/f1p0/3216	32.33657995	171.5144495	-2.4071	3.90E-05	0.00049589
i1_LQ_YHS_c39562/f1p0/1330	63.82755527	264.9246311	-2.0533	3.91E-05	0.0004967
i2_LQ_YHS_c18050/f1p0/2779	158.4137594	615.9209475	-1.959	3.92E-05	0.00049785
i2_LQ_YHS_c50567/f1p6/2886	119.6826496	904.8319415	-2.9184	3.93E-05	0.00050004
i2_LQ_YHS_c20265/f1p0/2270	140.3365241	22.84699507	2.6188	3.94E-05	0.00050024
i1_LQ_YHS_c22756/f1p5/1743	9.909218713	79.41023303	-3.0025	3.96E-05	0.00050289
i2_LQ_YHS_c33086/f1p0/2935	14.32964034	96.4148988	-2.7503	3.96E-05	0.00050289
i5_LQ_YHS_c2087/f1p0/5976	1.536273569	37.74849067	-4.6189	3.96E-05	0.00050289
i3_HQ_YHS_c1423/f6p5/3473	1425.082307	329.4508701	2.1129	3.97E-05	0.00050351
i3_LQ_YHS_c2153/f2p1/3574	36.02542778	157.5228679	-2.1285	3.98E-05	0.00050499
i4_LQ_YHS_c12101/f1p5/4795	2.519909767	85.38617407	-5.0826	3.99E-05	0.00050604
i3_LQ_YHS_c9955/f1p1/3630	383.9712489	1782.100017	-2.2145	4.00E-05	0.00050715
i3_LQ_YHS_c2298/f2p0/3658	4.887039232	58.01439134	-3.5694	4.02E-05	0.00050952
i1_LQ_YHS_c20241/f1p2/1376	325.1946285	78.41149083	2.0522	4.03E-05	0.00051054
i0_LQ_YHS_c1496/f1p1/933	0	20.51451836	-Inf	4.04E-05	0.00051159
i1_LQ_YHS_c13983/f1p0/1304	275.0025178	64.16493636	2.0996	4.04E-05	0.00051183
i2_HQ_YHS_c7251/f2p1/2563	11.06614984	171.0081274	-3.9498	4.04E-05	0.00051183
i2_LQ_YHS_c11699/f1p3/2163	0.272341299	24.72646672	-6.5045	4.04E-05	0.00051183
i2_LQ_YHS_c32943/f1p24/2993	0.359624837	24.63412858	-6.098	4.04E-05	0.00051183
i2_LQ_YHS_c51365/f1p3/2215	1114.112454	18.00905554	5.951	4.04E-05	0.00051183
i3_LQ_YHS_c5017/f1p6/3298	5.416617687	82.00684844	-3.9203	4.05E-05	0.00051183
i3_LQ_YHS_c6729/f1p1/3386	0	20.50061638	-Inf	4.05E-05	0.00051183
i0_LQ_YHS_c1973/f1p0/759	0.544682597	27.80061356	-5.6736	4.07E-05	0.00051469
i4_LQ_YHS_c7142/f1p0/4659	61.25723614	265.006346	-2.1131	4.07E-05	0.00051501
i2_HQ_YHS_c46036/f14p3/2134	207.1806199	830.4396815	-2.003	4.08E-05	0.0005158
i2_LQ_YHS_c36820/f1p5/2281	36.33984313	174.1019793	-2.2603	4.11E-05	0.00051982
i3_LQ_YHS_c11605/f1p2/3374	953.544396	207.8018354	2.1981	4.12E-05	0.00051998

i3_LQ_YHS_c8306/f1p89/3258	217.9358657	48.43701279	2.1697	4.12E-05	0.00051998
i3_LQ_YHS_c16922/f1p0/3745	216.9243955	858.6675822	-1.9849	4.16E-05	0.00052491
i3_LQ_YHS_c10338/f1p0/3109	77.87466881	323.9638705	-2.0566	4.16E-05	0.00052503
i4_LQ_YHS_c12594/f1p0/4334	15.39928794	166.9909276	-3.4388	4.17E-05	0.00052647
i1_LQ_YHS_c3058/f1p0/1836	1331.977521	55.09719704	4.5954	4.20E-05	0.00053026
i2_LQ_YHS_c38075/f1p0/2781	44.76268903	212.5661551	-2.2475	4.23E-05	0.00053412
i1_LQ_YHS_c11622/f1p1/1701	349.1461749	62.26145091	2.4874	4.24E-05	0.00053441
i1_LQ_YHS_c32800/f1p1/1823	89.50397183	12.11957159	2.8846	4.25E-05	0.00053637
i3_LQ_YHS_c4370/f1p0/3262	31.3517913	396.0933494	-3.6592	4.26E-05	0.00053687
i1_LQ_YHS_c10562/f1p12/1464	54.75460295	233.9971476	-2.0954	4.27E-05	0.00053845
i1_LQ_YHS_c11475/f1p2/1847	66.125058	1.530655327	5.433	4.29E-05	0.00054009
i2_LQ_YHS_c21054/f1p1/3008	108.2847005	419.4663633	-1.9537	4.30E-05	0.00054149
i4_LQ_YHS_c7599/f1p0/4675	110.7801343	433.5742595	-1.9686	4.30E-05	0.00054149
i1_LQ_YHS_c12652/f1p3/1698	1425.304051	113.7320506	3.6476	4.31E-05	0.00054306
i1_HQ_YHS_c17006/f3p0/1786	411.1577794	22.10477172	4.2173	4.32E-05	0.00054329
i3_HQ_YHS_c21085/f5p3/3509	486.6575339	19.92924754	4.6099	4.32E-05	0.00054393
i1_LQ_YHS_c1796/f5p6/1773	151.6739034	595.8709244	-1.974	4.33E-05	0.00054485
i1_LQ_YHS_c4649/f1p0/1621	160.9600945	610.0916065	-1.9223	4.36E-05	0.00054771
i0_LQ_YHS_c2712/f1p3/873	785.4333571	201.9174469	1.9597	4.36E-05	0.00054774
i2_LQ_YHS_c26833/f1p0/2321	253.3508951	58.61976605	2.1117	4.37E-05	0.00054884
i0_HQ_YHS_c297/f3p0/691	6350.277047	768.3790184	3.0469	4.38E-05	0.00055002
i2_LQ_YHS_c49856/f1p5/2920	34.02227575	175.8794405	-2.37	4.39E-05	0.00055145
i1_LQ_YHS_c7294/f1p0/1406	418.1840395	1856.695198	-2.1505	4.40E-05	0.00055288
i1_HQ_YHS_c23096/f2p0/1660	2690.528241	545.3164357	2.3027	4.41E-05	0.00055374
i2_LQ_YHS_c37983/f1p1/2186	26.43523787	135.7923514	-2.3609	4.42E-05	0.00055445
i1_LQ_YHS_c21566/f1p0/1944	469.7112446	0	Inf	4.42E-05	0.00055471
i1_LQ_YHS_c34053/f1p2/1978	15.75048289	106.1809438	-2.7531	4.42E-05	0.00055471
i2_HQ_YHS_c45354/f5p1/3018	97.51267031	383.956434	-1.9773	4.43E-05	0.00055579
i2_LQ_YHS_c14386/f1p28/2113	47.88297319	2664.661796	-5.7983	4.43E-05	0.00055581
i1_LQ_YHS_c10708/f1p12/1786	9.674235067	205.7170254	-4.4104	4.45E-05	0.0005574
i3_HQ_YHS_c2314/f2p0/3252	335.726748	77.84908894	2.1085	4.46E-05	0.00055908
i1_LQ_YHS_c3161/f1p4/1874	0	19.90641929	-Inf	4.47E-05	0.00056014
i4_LQ_YHS_c7932/f1p6/4560	3.858557342	53.38731414	-3.7904	4.48E-05	0.00056094
i2_LQ_YHS_c8858/f1p0/2724	609.312941	3358.697682	-2.4626	4.48E-05	0.00056095
i5_LQ_YHS_c3814/f1p2/5170	39.68471481	0	Inf	4.48E-05	0.00056132
i1_HQ_YHS_c6967/f2p0/1686	441.9667597	3817.06811	-3.1105	4.51E-05	0.00056455
i3_LQ_YHS_c5505/f1p2/3329	42.5294528	275.491507	-2.6955	4.52E-05	0.00056507
i1_LQ_YHS_c7386/f1p0/1481	5.285293863	59.04812387	-3.4818	4.52E-05	0.00056522
i1_LQ_YHS_c10590/f1p4/1793	0	24.74814896	-Inf	4.52E-05	0.00056525
i2_LQ_YHS_c22954/f1p3/2783	68.24542425	7.458015512	3.1939	4.55E-05	0.00056836
i2_LQ_YHS_c40066/f1p2/2522	19.26659709	116.5285496	-2.5965	4.55E-05	0.00056836
i4_LQ_YHS_c2352/f2p1/4355	15.84619631	101.0888181	-2.6734	4.55E-05	0.00056836
i4_LQ_YHS_c8513/f1p0/4889	2.615148079	40.89609798	-3.967	4.55E-05	0.00056836
i5_LQ_YHS_c3229/f1p2/5107	779.5201121	7613.044923	-3.2878	4.55E-05	0.00056855
i1_LQ_YHS_c25012/f1p3/1873	429.1932015	69.37159613	2.6292	4.58E-05	0.00057151
i1_LQ_YHS_c20435/f1p0/1579	0	26.0122945	-Inf	4.59E-05	0.00057311
i1_LQ_YHS_c3325/f1p0/1787	0	20.2574474	-Inf	4.59E-05	0.00057315
i1_LQ_YHS_c29023/f1p2/1269	68.87946793	280.4343862	-2.0255	4.61E-05	0.00057467
i2_LQ_YHS_c55273/f1p0/2462	0	20.23908229	-Inf	4.61E-05	0.00057467
i3_LQ_YHS_c10316/f1p0/3455	8.176591292	72.11695034	-3.1408	4.61E-05	0.00057522
i2_LQ_YHS_c54496/f1p2/2492	18.41475145	170.2437439	-3.2087	4.62E-05	0.0005764
i3_LQ_YHS_c17988/f1p0/3688	0	43.09211885	-Inf	4.67E-05	0.0005819
i2_HQ_YHS_c21078/f2p0/2915	36.91091758	176.8868683	-2.2607	4.68E-05	0.00058319
i6_LQ_YHS_c433/f1p0/6492	129.0508166	492.4696115	-1.9321	4.68E-05	0.00058334
i2_LQ_YHS_c7696/f1p2/2670	50.61399391	227.3641756	-2.1674	4.69E-05	0.00058351
i1_LQ_YHS_c9983/f1p1/1822	115.1879303	10.92888652	3.3978	4.70E-05	0.00058522
i1_LQ_YHS_c7207/f1p7/1872	80.43704154	318.3404233	-1.9846	4.70E-05	0.00058541
i1_LQ_YHS_c13679/f1p0/1303	118.1427044	674.313861	-2.5129	4.72E-05	0.0005876
i5_LQ_YHS_c1271/f1p0/5279	247.6259703	54.48404206	2.1843	4.74E-05	0.00059023
i3_LQ_YHS_c11332/f1p0/3174	3930.643043	2.15996363	10.83	4.77E-05	0.00059375
i3_LQ_YHS_c20898/f1p0/3055	494.1389015	2752.409508	-2.4777	4.79E-05	0.00059583
i1_LQ_YHS_c25328/f1p4/1886	11.73627904	85.01211772	-2.8567	4.79E-05	0.00059607
i3_LQ_YHS_c12078/f1p0/3146	3249.12375	615.6327769	2.3999	4.80E-05	0.00059659
i2_LQ_YHS_c52559/f1p1/2577	0.257237156	24.0654841	-6.5477	4.82E-05	0.00059862
i1_LQ_YHS_c22438/f1p0/1833	693.1019845	17.06987295	5.3435	4.84E-05	0.00060111
i2_LQ_YHS_c18020/f1p5/2347	1749.44503	46.93530742	5.2201	4.84E-05	0.00060111
i0_LQ_YHS_c2344/f1p0/978	0	19.93311791	-Inf	4.85E-05	0.00060191
i2_LQ_YHS_c50591/f1p36/2892	4663.010141	22.73230169	7.6804	4.86E-05	0.000603
i1_LQ_YHS_c21705/f1p0/1769	85.13141529	330.5009081	-1.9569	4.87E-05	0.00060446
i2_LQ_YHS_c48638/f1p4/2975	86.56195986	10.98840434	2.9778	4.88E-05	0.00060531
i3_LQ_YHS_c9597/f1p0/3214	102.5634305	411.9859948	-2.0061	4.88E-05	0.00060531
i2_LQ_YHS_c39894/f1p10/2184	46.23441606	209.5130571	-2.18	4.91E-05	0.00060847
i1_HQ_YHS_c7184/f4p22/1949	345.1583043	1437.671553	-2.0584	4.91E-05	0.00060884
i1_HQ_YHS_c11890/f2p0/1920	139.3740631	522.0556677	-1.9052	4.92E-05	0.00061002
i4_LQ_YHS_c2832/f1p0/4784	17.16131824	218.8348255	-3.6726	4.92E-05	0.00061002
i2_LQ_YHS_c25895/f1p1/2559	42.77076366	2.519957569	4.0852	4.93E-05	0.00061056
i4_HQ_YHS_c1982/f3p3/4388	191.1904119	749.8488296	-1.9716	4.95E-05	0.00061245
i3_LQ_YHS_c12018/f1p0/3229	58.9686384	5.851648377	3.333	4.97E-05	0.00061549
i2_HQ_YHS_c61717/f4p2/2731	789.1226095	6548.462669	-3.0528	4.98E-05	0.00061601
i2_LQ_YHS_c5888/f1p3/2370	48.61791343	222.2868573	-2.1929	4.98E-05	0.00061614
i2_HQ_YHS_c17802/f2p2/2856	4.003721541	204.8312427	-5.677	4.98E-05	0.00061618
i1_LQ_YHS_c13495/f1p0/1349	0	46.79885158	-Inf	4.99E-05	0.00061713
i2_LQ_YHS_c29290/f1p1/2049	67.35497394	2.984533058	4.4962	5.00E-05	0.00061839
i3_LQ_YHS_c22473/f1p9/3032	111.7720233	9.567346933	3.5463	5.01E-05	0.00061967
i4_LQ_YHS_c11995/f1p1/4938	7.147175933	818.2967472	-6.8391	5.02E-05	0.00062016
i1_LQ_YHS_c35607/f1p0/1891	200.8688762	0	Inf	5.04E-05	0.00062301
i2_HQ_YHS_c60752/f3p2/2573	925.2894542	237.506169	1.9619	5.04E-05	0.00062301
i6_LQ_YHS_c418/f1p0/6670	52.93515035	230.9002527	-2.125	5.05E-05	0.00062326
i1_LQ_YHS_c39686/f1p1/1301	542.2983745	2682.016788	-2.3062	5.06E-05	0.00062483

i2_LQ_YHS_c51845/f1p1/2568	267.0996429	65.60254101	2.0256	5.06E-05	0.00062483
i3_LQ_YHS_c5005/f1p0/3825	13.09337207	91.01795361	-2.7973	5.07E-05	0.00062511
i1_LQ_YHS_c20258/f1p1/1730	163.4548104	0	Inf	5.07E-05	0.00062555
i4_LQ_YHS_c5525/f1p3/4799	65.52855746	328.5414197	-2.3259	5.07E-05	0.00062563
i1_LQ_YHS_c11610/f1p0/1613	1.963464343	42.27592247	-4.4284	5.08E-05	0.00062582
i2_HQ_YHS_c17471/f3p0/2679	71.26391552	283.1091647	-1.9901	5.11E-05	0.00062956
i2_LQ_YHS_c17899/f1p1/2556	2.467447971	40.32413636	-4.0306	5.14E-05	0.00063288
i3_LQ_YHS_c10008/f1p7/3888	27.43810819	145.6794449	-2.4085	5.14E-05	0.00063333
i1_LQ_YHS_c14015/f1p0/1063	538.0654305	17883.96023	-5.0547	5.15E-05	0.00063483
i4_LQ_YHS_c13479/f1p0/4054	120.5120562	459.8785758	-1.9321	5.17E-05	0.00063688
i3_HQ_YHS_c1735/f5p0/3196	333.4890491	1390.273222	-2.0597	5.18E-05	0.0006373
i4_LQ_YHS_c3327/f1p2/4307	6.536191166	64.91154264	-3.312	5.18E-05	0.00063783
i2_LQ_YHS_c52482/f1p0/2374	0	25.63839858	-Inf	5.18E-05	0.00063792
i3_LQ_YHS_c14402/f1p0/3082	42.93021822	1.961305157	4.4521	5.20E-05	0.00063926
i3_LQ_YHS_c19363/f1p6/3669	249.4187821	1235.358728	-2.3083	5.21E-05	0.00064039
i5_LQ_YHS_c4820/f1p0/5084	32.83581368	374.3266784	-3.511	5.21E-05	0.00064039
i2_LQ_YHS_c55341/f1p1/2146	27.85227237	143.369363	-2.3639	5.21E-05	0.00064062
i2_LQ_YHS_c5777/f1p8/2113	0.771711468	40.51655337	-5.7143	5.21E-05	0.00064062
i2_HQ_YHS_c60354/f2p3/2062	217.0271913	857.6100061	-1.9824	5.22E-05	0.00064179
i2_LQ_YHS_c11614/f1p31/2787	277.2320573	2508.787631	-3.1778	5.23E-05	0.00064229
i1_LQ_YHS_c19316/f1p6/1575	175.4155033	656.7451238	-1.9046	5.26E-05	0.00064535
i2_HQ_YHS_c60195/f3p2/2339	357.2716425	9.954552048	5.1655	5.26E-05	0.00064535
i1_HQ_YHS_c1649/f6p0/1694	696.6526427	4171.762644	-2.5821	5.28E-05	0.0006481
i1_HQ_YHS_c16769/f6p0/1640	362.0842009	1527.894838	-2.0771	5.29E-05	0.00064856
i2_HQ_YHS_c10577/f2p3/2330	39.59790639	349.0828438	-3.1401	5.30E-05	0.00065043
i2_LQ_YHS_c14531/f1p8/2128	6.639850988	64.22038496	-3.2738	5.31E-05	0.00065068
i4_LQ_YHS_c4320/f1p2/4872	5.295784547	139.4029091	-4.7183	5.31E-05	0.00065068
i4_HQ_YHS_c2295/f2p0/4776	163.78677	637.6340905	-1.9609	5.31E-05	0.00065089
i1_LQ_YHS_c34365/f1p0/2140	323.980293	0.353872233	9.8385	5.34E-05	0.00065372
i2_LQ_YHS_c41086/f1p0/2154	12.26377995	89.93457445	-2.8745	5.34E-05	0.00065372
i1_LQ_YHS_c43639/f1p0/1069	44.93266777	199.9484356	-2.1538	5.35E-05	0.0006549
i0_LQ_YHS_c497/f2p0/464	7766.462949	155.1065843	5.6459	5.38E-05	0.00065854
i2_LQ_YHS_c41301/f1p0/3017	51.10047392	224.3178328	-2.1341	5.38E-05	0.00065913
i1_LQ_YHS_c34355/f1p1/1577	72.03690751	0	Inf	5.39E-05	0.00065915
i2_LQ_YHS_c10821/f1p2/2976	108.7722994	433.3886278	-1.9944	5.39E-05	0.00065915
i2_LQ_YHS_c23146/f1p1/2532	81.43751239	10.26229476	2.9883	5.39E-05	0.00065915
i0_LQ_YHS_c2929/f1p3/954	0.817023896	29.75413844	-5.1866	5.41E-05	0.00066207
i2_LQ_YHS_c53180/f1p3/2979	455.086702	7.11192355	5.9998	5.42E-05	0.00066231
i2_LQ_YHS_c5034/f1p6/2551	2.065852024	53.86196079	-4.7045	5.43E-05	0.00066327
i1_HQ_YHS_c1617/f7p4/1545	621.4163568	3335.391776	-2.4242	5.43E-05	0.00066404
i1_LQ_YHS_c23422/f1p0/1824	78.83385426	313.691743	-1.9925	5.44E-05	0.00066472
i1_LQ_YHS_c28135/f1p0/1348	332.6515046	2.926120577	6.8289	5.45E-05	0.00066577
i3_LQ_YHS_c17228/f1p0/3234	29.81125131	0.713866171	5.3841	5.45E-05	0.00066577
i1_HQ_YHS_c2554/f2p2/1830	555.4370016	145.1754148	1.9358	5.46E-05	0.00066636
i5_LQ_YHS_c4318/f1p7/5745	48.36100657	390.4285495	-3.0131	5.46E-05	0.00066679
i3_HQ_YHS_c2556/f5p0/3798	797.5851585	7974.97601	-3.3218	5.47E-05	0.00066713
i2_HQ_YHS_c2315/f2p0/2552	0	21.06811485	-Inf	5.47E-05	0.00066764
i2_LQ_YHS_c6661/f1p44/2080	18393.93697	0	Inf	5.49E-05	0.00066934
i1_LQ_YHS_c17633/f1p1/1858	545.9186667	4.699944881	6.8599	5.50E-05	0.00067021
i2_LQ_YHS_c33153/f1p1/2896	57.06039075	233.0257386	-2.0299	5.50E-05	0.00067021
i2_LQ_YHS_c5115/f1p2/2290	618.4163912	162.6630212	1.9267	5.50E-05	0.00067021
i2_LQ_YHS_c33122/f1p2/2449	240.7890414	935.4026477	-1.9578	5.52E-05	0.00067268
i3_LQ_YHS_c8458/f1p2/3454	0	19.67158381	-Inf	5.52E-05	0.00067268
i2_LQ_YHS_c26006/f1p1/2596	66.46079339	7.259949815	3.1945	5.53E-05	0.00067383
i2_LQ_YHS_c3155/f1p2/2488	5.840467144	133.2556466	-4.512	5.54E-05	0.00067401
i1_LQ_YHS_c7786/f1p0/1620	132.2209358	4.366096331	4.9205	5.54E-05	0.00067407
i2_LQ_YHS_c20722/f1p4/2579	0	19.64097529	-Inf	5.55E-05	0.00067541
i1_HQ_YHS_c37833/f2p0/1243	648.3800757	3929.475861	-2.5994	5.55E-05	0.00067549
i1_LQ_YHS_c8522/f1p0/1714	217.047822	0	Inf	5.57E-05	0.0006769
i2_LQ_YHS_c27114/f1p3/2306	50.45468416	214.2498915	-2.0862	5.59E-05	0.00067914
i1_LQ_YHS_c5741/f1p2/1892	109.846594	17.96675683	2.6121	5.59E-05	0.00067935
i3_HQ_YHS_c2400/f2p1/3966	264.7748639	1052.289887	-1.9907	5.62E-05	0.00068247
i1_LQ_YHS_c28777/f1p1/1279	40.52399222	3069.459764	-6.2431	5.62E-05	0.00068278
i3_HQ_YHS_c15728/f2p0/3076	41.30113093	188.1812777	-2.1879	5.62E-05	0.00068278
i2_HQ_YHS_c9778/f3p0/2338	48.60363145	208.6679108	-2.1021	5.68E-05	0.00068929
i0_HQ_YHS_c294/f3p0/1016	5062.927038	729.7648451	2.7945	5.70E-05	0.00069152
i1_LQ_YHS_c14668/f1p0/1412	893.4163193	230.1185759	1.957	5.71E-05	0.00069318
i4_LQ_YHS_c3148/f1p5/4870	183.3835986	0.75225497	7.9294	5.71E-05	0.00069328
i2_LQ_YHS_c54471/f1p0/2531	36.03937109	164.8490895	-2.1935	5.72E-05	0.000694
i1_LQ_YHS_c10259/f1p0/1611	82.73851269	333.5401426	-2.0112	5.73E-05	0.00069497
i1_LQ_YHS_c3407/f1p2/1751	0	82.22987397	-Inf	5.75E-05	0.00069781
i1_HQ_YHS_c13442/f2p4/1306	506.3619057	19705.94255	-5.2823	5.76E-05	0.00069862
i1_LQ_YHS_c12141/f1p9/1753	1168.854606	291.3365752	2.0043	5.76E-05	0.00069862
i2_LQ_YHS_c22444/f1p0/2863	4.071287478	118.2374793	-4.8601	5.77E-05	0.00069937
i4_LQ_YHS_c10182/f1p0/4569	110.0581717	412.04737	-1.9045	5.79E-05	0.00070094
i1_HQ_YHS_c2032/f5p0/1858	1024.145778	261.6179815	1.9689	5.81E-05	0.00070364
i1_HQ_YHS_c16927/f4p0/1571	636.411513	3861.873548	-2.6013	5.81E-05	0.0007038
i2_LQ_YHS_c20052/f1p0/2952	86.59813994	346.8929375	-2.0021	5.84E-05	0.00070654
i1_LQ_YHS_c32330/f1p0/1766	0.272341299	23.24762915	-6.4155	5.86E-05	0.00070864
i1_LQ_YHS_c7172/f1p0/1896	59.00346852	5.524514309	3.4169	5.86E-05	0.00070864
i2_LQ_YHS_c36272/f1p1/2176	59.75611461	250.9702599	-2.0704	5.89E-05	0.00071245
i2_LQ_YHS_c13713/f1p11/2583	249.8767018	957.285152	-1.9377	5.90E-05	0.00071398
i3_LQ_YHS_c18456/f1p0/3689	116.322207	439.0051293	-1.9161	5.91E-05	0.00071454
i4_LQ_YHS_c12317/f1p0/4883	2.103209677	39.72768793	-4.2395	5.91E-05	0.00071481
i2_HQ_YHS_c2219/f9p3/2426	86.18895301	331.9307374	-1.9453	5.93E-05	0.00071612
i2_LQ_YHS_c58766/f1p0/2075	196.0339529	708.9805383	-1.8546	5.96E-05	0.00072048
i0_LQ_YHS_c2814/f1p1/567	159.639215	33.71620334	2.2433	5.98E-05	0.00072211
i2_HQ_YHS_c29712/f3p3/3004	736.2510804	194.924481	1.9173	5.98E-05	0.00072211
i3_LQ_YHS_c18018/f1p0/3110	3.374291315	48.50213975	-3.8454	5.99E-05	0.00072337

i5_LQ_YHS_c3430/f1p4/5198	410.5191306	19.35057145	4.407	5.99E-05	0.00072348
i5_LQ_YHS_c1983/f1p3/6278	154.8351762	31.5446693	2.2953	6.00E-05	0.00072407
i7_LQ_YHS_c218/f1p0/7766	199.6441685	758.0818949	-1.9249	6.00E-05	0.00072407
i1_LQ_YHS_c14157/f1p0/1180	132.0547363	485.8787466	-1.8795	6.04E-05	0.00072823
i2_HQ_YHS_c5982/f4p1/2244	362.1038575	92.15571558	1.9743	6.05E-05	0.00072869
i3_HQ_YHS_c2269/f2p1/3632	480.691151	2310.837657	-2.2652	6.05E-05	0.00072869
i1_LQ_YHS_c28988/f1p0/1166	306.9775928	1230.07608	-2.0025	6.06E-05	0.00073004
i4_HQ_YHS_c2393/f2p5/4388	71.50715075	294.2461486	-2.0409	6.08E-05	0.00073299
i2_LQ_YHS_c2158/f1p1/2600	342.641195	88.87674902	1.9468	6.09E-05	0.00073304
i1_LQ_YHS_c14502/f1p2/1295	164.7441304	599.9292434	-1.8646	6.09E-05	0.00073305
i1_LQ_YHS_c6086/f1p0/1459	201.2438374	45.81519803	2.135	6.09E-05	0.00073305
i3_LQ_YHS_c6942/f1p0/3124	26.12679431	140.465279	-2.4266	6.09E-05	0.00073305
i4_LQ_YHS_c7765/f1p2/5031	2.517373857	90.83967207	-5.1733	6.09E-05	0.00073305
i1_HQ_YHS_c1279/f5p0/1800	445.4668662	2055.502842	-2.2061	6.11E-05	0.00073497
i3_LQ_YHS_c5395/f1p1/3916	47.9385657	211.6060819	-2.1421	6.12E-05	0.00073571
i3_LQ_YHS_c15077/f1p8/3101	145.9407677	555.7043741	-1.9289	6.12E-05	0.00073581
i1_LQ_YHS_c25067/f1p0/1551	151.4183968	584.8768725	-1.9496	6.12E-05	0.0007361
i2_LQ_YHS_c40853/f1p3/2499	9.996502251	84.89146194	-3.0861	6.13E-05	0.00073662
i3_LQ_YHS_c5107/f1p0/3421	324.6658375	1347.02209	-2.0527	6.13E-05	0.00073697
i3_LQ_YHS_c6292/f1p0/3113	21.10254566	121.3619458	-2.5238	6.15E-05	0.00073813
i3_LQ_YHS_c6854/f1p1/3170	51.61560045	337.5582104	-2.7093	6.16E-05	0.00073943
i1_LQ_YHS_c10034/f1p2/1863	126.6847487	1.427732343	6.4714	6.17E-05	0.00074033
i3_LQ_YHS_c2999/f1p1/3580	7.57102539	66.41324801	-3.1329	6.17E-05	0.0007408
i1_LQ_YHS_c43812/f1p2/1065	311.1525378	2.501592453	6.9586	6.21E-05	0.00074522
i2_LQ_YHS_c11139/f1p2/2601	8.954985394	74.09827918	-3.0487	6.21E-05	0.00074553
i1_HQ_YHS_c17075/f2p6/1749	63.23072443	356.25104	-2.4942	6.22E-05	0.00074631
i2_LQ_YHS_c19366/f1p0/2134	24.02739269	184.0077566	-2.937	6.23E-05	0.00074749
i1_HQ_YHS_c9839/f3p0/1747	170.7017591	626.6043324	-1.8761	6.26E-05	0.00075086
i2_LQ_YHS_c28907/f1p145/2026	819.7542728	0	Inf	6.27E-05	0.00075107
i1_LQ_YHS_c28055/f1p0/1240	34.25379839	163.5545761	-2.2554	6.28E-05	0.00075205
i5_LQ_YHS_c1220/f1p2/5728	33.81404776	161.1924369	-2.2531	6.30E-05	0.00075508
i3_LQ_YHS_c10178/f1p0/3379	2.395268576	39.40161965	-4.04	6.32E-05	0.00075638
i3_LQ_YHS_c1944/f1p0/3881	176.3824078	666.4386159	-1.9178	6.32E-05	0.00075669
i3_LQ_YHS_c11661/f1p0/3536	86.28291081	335.0404696	-1.9572	6.32E-05	0.00075691
i1_LQ_YHS_c35081/f1p0/1723	86.96354743	331.6678991	-1.9313	6.34E-05	0.00075911
i4_LQ_YHS_c6844/f1p0/4240	0.257237156	23.25315808	-6.4982	6.35E-05	0.00076025
i5_LQ_YHS_c3070/f1p1/5317	40.68045251	186.5905909	-2.1975	6.36E-05	0.00076065
i3_LQ_YHS_c4782/f1p0/3632	94.1288469	13.2773968	2.8257	6.37E-05	0.00076156
i1_HQ_YHS_c30230/f13p0/1125	335.7544419	24624.7995	-6.1966	6.39E-05	0.0007634
i2_LQ_YHS_c10161/f1p3/2731	198.081454	45.12194831	2.1342	6.39E-05	0.00076419
i1_LQ_YHS_c14714/f1p3/1179	35.07382493	169.3061061	-2.2712	6.40E-05	0.00076497
i1_LQ_YHS_c20776/f1p0/1617	108.110472	405.4083566	-1.9069	6.40E-05	0.00076497
i1_LQ_YHS_c19486/f1p0/1507	846.3491229	220.6753216	1.9393	6.44E-05	0.00076902
i2_LQ_YHS_c22503/f1p0/2146	24.70294075	129.8786536	-2.3944	6.44E-05	0.00076902
i2_LQ_YHS_c53165/f1p3/2063	384.5647755	1618.182449	-2.0731	6.48E-05	0.00077389
i2_LQ_YHS_c62687/f1p1/2351	146.6703477	541.0173298	-1.8831	6.48E-05	0.00077389
i2_LQ_YHS_c25282/f1p4/2680	30.88713993	146.2452046	-2.2433	6.53E-05	0.00077954
i2_LQ_YHS_c9721/f1p1/2435	2.220701499	64.19878181	-4.8535	6.54E-05	0.00078068
i3_LQ_YHS_c7580/f1p2/3205	20.12940852	114.6799595	-2.5102	6.55E-05	0.00078122
i1_HQ_YHS_c2566/f2p0/1587	577.2582883	2858.023702	-2.3077	6.55E-05	0.00078141
i2_LQ_YHS_c59931/f1p8/2083	12.66411213	86.29182379	-2.7685	6.62E-05	0.0007892
i3_LQ_YHS_c6836/f1p0/3860	144.0257633	540.679417	-1.9084	6.64E-05	0.0007909
i0_HQ_YHS_c301/f3p0/896	346.6082422	24618.98567	-6.1503	6.67E-05	0.00079517
i3_LQ_YHS_c13312/f1p0/3131	1397.658973	306.5679304	2.1887	6.69E-05	0.00079681
i2_HQ_YHS_c7442/f5p3/2283	38.33443248	262.057996	-2.7732	6.70E-05	0.00079836
i3_LQ_YHS_c11890/f1p0/3729	0.544682597	26.6799916	-5.6142	6.71E-05	0.00079852
i1_LQ_YHS_c32616/f1p0/1876	0	262.3717308	-Inf	6.72E-05	0.00079939
i2_LQ_YHS_c3374/f1p30/2282	176.8941069	10.04416583	4.1385	6.73E-05	0.00080025
i3_HQ_YHS_c21160/f2p0/3112	372.3815774	0.746133264	8.9631	6.74E-05	0.00080165
i1_LQ_YHS_c36218/f1p1/1412	202.8302809	21.3825325	3.2458	6.76E-05	0.00080341
i2_LQ_YHS_c11575/f1p0/2495	117.6913609	10.93161088	3.4284	6.76E-05	0.00080417
i2_LQ_YHS_c40451/f1p1/2103	408.3444715	38.64438898	3.4015	6.76E-05	0.00080417
i3_LQ_YHS_c17406/f1p0/3370	226.6051972	890.9784468	-1.9752	6.78E-05	0.00080546
i1_HQ_YHS_c16826/f5p0/1657	1760.798986	308.443218	2.5132	6.80E-05	0.00080771
i2_LQ_YHS_c27042/f1p6/2188	23.99719278	127.5873703	-2.4105	6.80E-05	0.00080809
i2_LQ_YHS_c52598/f1p6/2106	266.2855845	1016.262258	-1.9322	6.82E-05	0.00080934
i4_LQ_YHS_c2660/f1p7/4830	64.11282023	0	Inf	6.82E-05	0.00080934
i4_LQ_YHS_c7120/f1p0/4530	63.74199386	7.150312348	3.1562	6.82E-05	0.00080934
i5_HQ_YHS_c607/f3p0/5409	0	24.51390627	-Inf	6.82E-05	0.00080934
i1_LQ_YHS_c24568/f1p7/1908	4.172403017	51.49938923	-3.6256	6.84E-05	0.00081207
i2_LQ_YHS_c50285/f1p6/2173	291.776315	1134.687976	-1.9594	6.88E-05	0.00081646
i1_LQ_YHS_c25444/f1p0/1794	129.3076193	865.0448962	-2.742	6.91E-05	0.00081924
i4_LQ_YHS_c13718/f1p0/4182	205.6165639	3.433548003	5.9041	6.96E-05	0.00082504
i1_LQ_YHS_c19313/f1p0/1580	86.33109781	333.7499391	-1.9508	6.98E-05	0.00082723
i1_LQ_YHS_c3043/f1p1/1992	676.6888389	181.450598	1.8989	7.00E-05	0.00082961
i2_LQ_YHS_c41678/f1p8/2979	326.6477724	1330.540165	-2.0262	7.00E-05	0.00082987
i5_HQ_YHS_c674/f2p0/6004	0	58.30688721	-Inf	7.01E-05	0.00083
i2_HQ_YHS_c7033/f5p21/2211	594.7613149	3182.973386	-2.42	7.02E-05	0.00083072
i2_LQ_YHS_c54365/f1p0/2222	38.61714477	212.6270178	-2.461	7.10E-05	0.00084008
i3_HQ_YHS_c1794/f3p0/3419	235.303545	56.47710175	2.0588	7.10E-05	0.00084015
i2_LQ_YHS_c39435/f2p6/2234	13.37460162	104.3511583	-2.9639	7.15E-05	0.00084645
i1_HQ_YHS_c27206/f2p1/1237	749.1906574	4643.556987	-2.6318	7.17E-05	0.00084781
i2_LQ_YHS_c54298/f1p3/2835	58.18342523	240.702829	-2.0486	7.20E-05	0.00085188
i1_LQ_YHS_c23932/f1p0/1907	204.6112594	48.76422708	2.069	7.21E-05	0.00085196
i1_HQ_YHS_c37458/f7p0/1346	748.30326	4496.062187	-2.587	7.22E-05	0.00085348
i2_LQ_YHS_c8425/f1p3/2398	92.88599894	361.9102664	-1.9621	7.24E-05	0.00085548
i2_LQ_YHS_c53524/f1p0/2404	6.606301387	61.79849444	-3.2257	7.25E-05	0.00085644
i2_LQ_YHS_c50000/f1p3/2266	1.90638909	37.50480913	-4.2982	7.27E-05	0.00085845
i2_HQ_YHS_c17319/f9p2/2904	218.8469826	837.5186638	-1.9362	7.28E-05	0.0008593

i2_HQ_YHS_c5826/f3p2/2104	475.7299279	0.353872233	10.393	7.32E-05	0.00086368
i2_LQ_YHS_c39964/f1p1/2454	73.12103095	289.2457287	-1.9839	7.32E-05	0.00086431
i1_LQ_YHS_c23028/f1p3/1388	108.1778692	0.392261031	8.1074	7.35E-05	0.00086656
i1_LQ_YHS_c35092/f1p20/1475	477.8842972	129.1182652	1.888	7.35E-05	0.00086656
i3_LQ_YHS_c9695/f1p0/3705	21.10761748	125.0051706	-2.5662	7.39E-05	0.00087133
i3_LQ_YHS_c9913/f1p0/3500	21.53180561	123.919067	-2.5249	7.41E-05	0.00087324
i2_LQ_YHS_c39460/f1p8/2119	4.357460777	51.71538658	-3.569	7.41E-05	0.00087397
i3_LQ_YHS_c16643/f1p0/3410	13.56507824	88.37343148	-2.7037	7.42E-05	0.00087397
i2_LQ_YHS_c5021/f1p0/2201	113.238517	415.3668174	-1.875	7.42E-05	0.00087422
i2_LQ_YHS_c51250/f1p0/2136	0	18.94547423	-Inf	7.42E-05	0.00087465
i5_LQ_YHS_c3235/f1p0/5538	66.84094124	267.3189979	-1.9998	7.46E-05	0.00087857
i1_LQ_YHS_c28265/f1p4/1255	125.0467971	1261.67327	-3.3348	7.49E-05	0.00088141
i3_LQ_YHS_c13252/f1p0/3434	125.0397435	3.465815096	5.173	7.50E-05	0.00088263
i4_LQ_YHS_c3535/f1p4/4684	338.3577877	1363.076911	-2.0102	7.51E-05	0.00088444
i7_LQ_YHS_c108/f1p0/7888	9.897455887	104.7675469	-3.404	7.53E-05	0.0008855
i1_LQ_YHS_c34885/f1p0/1552	404.9493448	105.9495452	1.9344	7.54E-05	0.00088722
i1_LQ_YHS_c4655/f1p2/1586	160.5196833	572.5727422	-1.8347	7.54E-05	0.00088722
i2_LQ_YHS_c10725/f1p75/2658	142.2624572	591.6323132	-2.0561	7.58E-05	0.00089135
i2_LQ_YHS_c41364/f1p1/2256	31.00254583	151.1532468	-2.2856	7.63E-05	0.00089645
i2_LQ_YHS_c59070/f1p3/2128	1205.853031	161.4956781	2.9005	7.63E-05	0.00089656
i4_HQ_YHS_c14872/f361p0/4752	1085.833326	279.550105	1.9576	7.63E-05	0.00089659
i1_LQ_YHS_c10971/f1p6/1601	195.6938267	21.90998361	3.1589	7.64E-05	0.00089693
i1_LQ_YHS_c39545/f1p0/1081	11.53992519	149.1833697	-3.6924	7.65E-05	0.00089873
i5_LQ_YHS_c4824/f1p0/5021	0.257237156	22.519861	-6.452	7.66E-05	0.00089918
i0_HQ_YHS_c366/f2p0/877	608.9087289	3156.541043	-2.374	7.66E-05	0.00089928
i2_HQ_YHS_c32509/f2p1/2262	74.5595303	294.7088271	-1.9828	7.67E-05	0.00089992
i2_LQ_YHS_c9494/f1p4/2757	4.56350828	276.7197485	-5.9221	7.69E-05	0.00090216
i2_LQ_YHS_c38340/f1p0/2146	1605.100867	327.500381	2.2931	7.71E-05	0.00090384
i1_HQ_YHS_c17004/f3p0/1905	8.027152308	172.7059201	-4.4273	7.72E-05	0.00090503
i3_HQ_YHS_c15375/f7p0/3328	348.5805385	1401.942118	-2.0079	7.74E-05	0.00090686
i1_LQ_YHS_c14214/f1p1/1323	4293.761072	716.9579071	2.5823	7.82E-05	0.00091621
i5_LQ_YHS_c5556/f1p0/5053	778.9166226	215.9045209	1.8511	7.82E-05	0.00091674
i2_LQ_YHS_c64679/f1p0/2000	154.1637665	32.62962681	2.2402	7.84E-05	0.00091827
i1_LQ_YHS_c6467/f1p0/1750	0	25.65842227	-Inf	7.85E-05	0.00091983
i2_HQ_YHS_c13712/f4p1/2392	327.9081121	1292.72024	-1.979	7.87E-05	0.0009215
i1_LQ_YHS_c28545/f1p2/1149	549.5802323	2527.700817	-2.2014	7.88E-05	0.00092253
i1_LQ_YHS_c20995/f1p13/1408	290.1628537	3.581574268	6.3401	7.90E-05	0.00092508
i1_LQ_YHS_c26275/f1p82/1464	20.42273119	0	Inf	7.91E-05	0.00092514
i1_HQ_YHS_c40323/f7p0/1529	1115.629509	1.864503878	9.2249	7.94E-05	0.00092865
i3_LQ_YHS_c9852/f1p0/3649	0	43.00530963	-Inf	7.95E-05	0.00092936
i2_HQ_YHS_c45753/f5p2/2227	46.02502724	388.7027841	-3.0782	7.96E-05	0.00093061
i1_LQ_YHS_c18703/f1p0/1528	2.995754284	44.41807421	-3.8902	7.96E-05	0.00093084
i2_LQ_YHS_c3808/f1p0/2367	95.53884334	363.1356699	-1.9263	7.99E-05	0.00093337
i5_LQ_YHS_c1723/f1p0/5150	0.817023896	28.26744039	-5.1126	7.99E-05	0.00093351
i2_LQ_YHS_c40879/f1p0/2919	4.881162006	51.81100231	-3.408	8.00E-05	0.00093446
i2_LQ_YHS_c9273/f1p0/2697	18.2410149	94.4532404	-2.3724	8.00E-05	0.00093446
i1_LQ_YHS_c33226/f1p0/1530	438.8347402	1897.011749	-2.112	8.01E-05	0.00093523
i0_LQ_YHS_c765/f1p0/898	392.4146299	1712.336091	-2.1255	8.03E-05	0.00093749
i3_HQ_YHS_c1401/f5p0/3876	162.0815433	576.3419265	-1.8302	8.04E-05	0.00093835
i3_LQ_YHS_c4421/f1p0/3236	8.258794637	68.50701889	-3.0522	8.07E-05	0.00094167
i1_LQ_YHS_c36135/f1p2/1605	556.0256993	19281.69239	-5.1159	8.09E-05	0.00094327
i1_LQ_YHS_c23893/f1p0/1491	24.65890884	121.8454396	-2.3049	8.09E-05	0.00094347
i2_LQ_YHS_c33273/f1p1/2921	63.43724704	525.4101438	-3.05	8.09E-05	0.00094347
i2_LQ_YHS_c49550/f1p24/2168	20.50286536	0	Inf	8.10E-05	0.00094358
i1_LQ_YHS_c24272/f1p0/2013	244.7593258	61.15070126	2.0009	8.12E-05	0.00094569
i1_LQ_YHS_c23266/f1p1/2007	21.19064298	113.6522685	-2.4231	8.18E-05	0.00095255
i3_LQ_YHS_c22568/f1p0/3029	11.78713002	86.53617833	-2.8761	8.19E-05	0.00095338
i3_LQ_YHS_c18957/f1p0/3425	273.0988477	1.961305157	7.1215	8.30E-05	0.00096665
i1_HQ_YHS_c9542/f2p0/1717	752.1726275	118.972836	2.6604	8.34E-05	0.00097112
i7_LQ_YHS_c156/f1p0/7765	4.039807051	50.11123124	-3.6328	8.34E-05	0.00097119
i2_LQ_YHS_c54583/f1p0/2437	1.634047791	35.24579288	-4.4309	8.35E-05	0.00097155
i3_LQ_YHS_c17941/f1p0/3514	8.711580238	72.46742522	-3.0563	8.35E-05	0.00097184
i1_LQ_YHS_c9129/f1p0/1873	159.8114401	572.9873596	-1.8421	8.36E-05	0.00097206
i3_LQ_YHS_c2989/f1p4/3541	259.2100794	980.6640564	-1.9196	8.39E-05	0.000975
i4_LQ_YHS_c6164/f1p0/4792	235.1248814	57.02224498	2.0438	8.42E-05	0.00097834
i2_HQ_YHS_c30540/f13p3/2060	649.5966516	3604.626323	-2.4722	8.43E-05	0.00097969
i3_LQ_YHS_c12702/f1p0/3712	30.24973818	152.8786111	-2.3374	8.44E-05	0.00098023
i2_LQ_YHS_c39672/f1p3/2152	806.3649711	16.33317852	5.6256	8.46E-05	0.00098331
i3_LQ_YHS_c6347/f1p0/3873	0	18.65779475	-Inf	8.51E-05	0.00098818
i2_LQ_YHS_c14815/f1p0/2053	194.4154018	0.359993938	9.077	8.52E-05	0.00098938
i3_LQ_YHS_c3529/f1p0/3771	2.057897249	38.39063516	-4.2215	8.52E-05	0.00098938
i3_HQ_YHS_c1860/f3p2/3404	334.1251309	26.3678253	3.6635	8.53E-05	0.00099031
i3_HQ_YHS_c21333/f2p5/3147	258.0331673	63.59565956	2.0206	8.56E-05	0.00099267
i1_LQ_YHS_c21305/f1p0/2031	167.0040648	35.5029836	2.2339	8.56E-05	0.00099311
i1_LQ_YHS_c11875/f1p0/1710	158.007331	590.9821538	-1.9031	8.61E-05	0.00099841
i2_LQ_YHS_c37197/f1p1/2185	526.9480072	15.6660742	5.0719	8.65E-05	0.0010028
i0_LQ_YHS_c3186/f1p0/857	177.9151677	5.504490626	5.0144	8.65E-05	0.0010031
i1_LQ_YHS_c34878/f1p2/1712	4.730120582	51.45713007	-3.4434	8.69E-05	0.0010072
i2_LQ_YHS_c24194/f1p3/2262	15.72489644	94.72136921	-2.5906	8.70E-05	0.001008
i4_LQ_YHS_c9559/f1p0/4264	61.5061966	6.400308722	3.2645	8.78E-05	0.0010174
i3_HQ_YHS_c13422/f2p2/3463	66.75857633	277.6100844	-2.056	8.82E-05	0.0010212
i1_LQ_YHS_c23394/f1p2/1588	0	18.4380468	-Inf	8.83E-05	0.001022
i2_LQ_YHS_c41366/f1p0/2220	22.63962463	121.9643961	-2.4295	8.84E-05	0.0010227
i1_LQ_YHS_c23907/f1p0/1512	234.461071	58.18512609	2.0106	8.84E-05	0.0010231
i1_LQ_YHS_c17755/f1p0/1767	509.5663044	29.24671102	4.1229	8.85E-05	0.0010238
i2_LQ_YHS_c3407/f1p2/2305	113.3758628	13.19615606	3.1029	8.87E-05	0.0010263
i2_LQ_YHS_c51765/f1p0/2547	107.3339616	11.15641474	3.2662	8.89E-05	0.0010281
i4_LQ_YHS_c11871/f1p0/4860	622.246521	18163.347	-4.8674	8.95E-05	0.0010345
i1_LQ_YHS_c38456/f1p0/1329	828.1264242	10.81032278	6.2594	8.99E-05	0.0010384

i1_LQ_YHS_c42734/f2p0/1369	377.6541856	54.28925394	2.7983	8.99E-05	0.0010384
i3_HQ_YHS_c1921/f3p0/3506	35.69530876	163.3601808	-2.1942	8.99E-05	0.0010384
i4_HQ_YHS_c2422/f2p2/5010	39.73844875	183.1345283	-2.2043	8.99E-05	0.0010384
i4_LQ_YHS_c10172/f1p3/4596	40.38712147	184.5261232	-2.1919	9.01E-05	0.0010398
i4_LQ_YHS_c6401/f1p5/5038	58.90440541	6.256745596	3.2349	9.01E-05	0.0010398
i2_LQ_YHS_c34170/f1p1/2763	165.3179227	13.5489625	3.609	9.06E-05	0.0010456
i3_LQ_YHS_c14641/f1p0/3063	35.51773904	160.2856016	-2.174	9.06E-05	0.0010458
i1_LQ_YHS_c20077/f1p0/1615	832.8712792	131.6294904	2.6616	9.07E-05	0.0010464
i2_LQ_YHS_c9044/f1p2/2918	141.6352613	29.67842664	2.2547	9.08E-05	0.0010474
i1_LQ_YHS_c9613/f3p0/1870	109.8084142	396.9734404	-1.8541	9.08E-05	0.0010475
i2_LQ_YHS_c8310/f1p3/2235	24.47924482	369.8809197	-3.9174	9.09E-05	0.001048
i1_HQ_YHS_c2255/f7p0/1821	247.3252505	1107.378046	-2.1627	9.10E-05	0.0010487
i1_LQ_YHS_c28423/f1p0/1286	122.1065324	450.0684538	-1.882	9.11E-05	0.0010501
i5_LQ_YHS_c781/f1p0/5365	5.989439394	58.40878395	-3.2857	9.16E-05	0.001055
i1_LQ_YHS_c13806/f1p0/1350	7.62555636	66.46230187	-3.1236	9.16E-05	0.0010554
i2_LQ_YHS_c56501/f1p2/2784	48.41314645	233.6440274	-2.2708	9.17E-05	0.0010558
i5_LQ_YHS_c1342/f1p3/6072	465.9154508	131.131582	1.8291	9.20E-05	0.0010591
i3_LQ_YHS_c13126/f1p2/3318	35.02805414	161.6486394	-2.2063	9.21E-05	0.0010605
i1_LQ_YHS_c35732/f1p384/1830	12.69893387	367.4766828	-4.8549	9.24E-05	0.001063
i2_LQ_YHS_c7015/f1p0/2763	214.2901498	776.4758228	-1.8574	9.26E-05	0.0010647
i1_HQ_YHS_c40379/f13p2/1359	379.630369	24791.20247	-6.0291	9.27E-05	0.0010656
i2_LQ_YHS_c56494/f1p2/2360	62.56027331	249.6151546	-1.9964	9.27E-05	0.0010661
i1_LQ_YHS_c9256/f1p0/1968	0	134.3217204	-Inf	9.30E-05	0.0010687
i2_HQ_YHS_c60293/f19p6/2626	520.3500962	144.4283295	1.8491	9.33E-05	0.0010726
i2_LQ_YHS_c55907/f1p0/2374	27.18802877	136.5582293	-2.3285	9.34E-05	0.001073
i1_LQ_YHS_c8772/f1p0/1694	11.08586745	78.98005622	-2.8328	9.35E-05	0.0010741
i4_LQ_YHS_c7723/f1p0/4316	3.889571034	46.34995639	-3.5749	9.36E-05	0.0010744
i1_LQ_YHS_c11697/f1p0/1918	84.78956694	326.885534	-1.9468	9.36E-05	0.0010746
i1_LQ_YHS_c6642/f1p0/1961	355.0806274	87.51698776	2.0205	9.36E-05	0.0010746
i1_LQ_YHS_c12097/f1p4/1985	40.67805304	178.5544514	-2.134	9.39E-05	0.0010773
i4_HQ_YHS_c2049/f3p3/4350	301.4792756	1166.03706	-1.9515	9.39E-05	0.0010773
i4_LQ_YHS_c7471/f1p3/4414	20.15119529	226.4228988	-3.4901	9.40E-05	0.0010777
i1_LQ_YHS_c42783/f1p0/1770	51382.09833	8.998702453	12.479	9.40E-05	0.0010781
i2_LQ_YHS_c22975/f1p0/2484	209.1039358	38.32444241	2.4479	9.41E-05	0.0010793
i1_LQ_YHS_c10059/f1p0/1769	79.23208377	372.5648283	-2.2333	9.42E-05	0.0010797
i1_LQ_YHS_c35636/f1p0/1734	223.7161017	50.89310917	2.1361	9.43E-05	0.0010804
i6_LQ_YHS_c232/f1p0/6105	26.17799234	133.13803	-2.3465	9.44E-05	0.001081
i1_LQ_YHS_c25083/f1p0/1681	0	62.89885319	-Inf	9.44E-05	0.0010817
i2_LQ_YHS_c32788/f1p1/2705	87.02568613	12.94753837	2.7488	9.45E-05	0.0010818
i6_LQ_YHS_c501/f1p0/6742	0.257237156	23.97259273	-6.5421	9.47E-05	0.001084
i2_LQ_YHS_c52752/f1p5/2142	4432.26531	202.2786599	4.4536	9.48E-05	0.0010847
i2_LQ_YHS_c58721/f1p1/2035	430.1423689	0.359993938	10.223	9.48E-05	0.0010849
i1_LQ_YHS_c32725/f1p0/1999	370.7961833	1.061616699	8.4482	9.49E-05	0.001086
i1_LQ_YHS_c3070/f1p1/1729	146.849326	9.235156948	3.9911	9.50E-05	0.0010862
i2_HQ_YHS_c5516/f3p0/2757	114.9897012	422.2217898	-1.8765	9.54E-05	0.0010908
i3_LQ_YHS_c18576/f1p0/3181	170.146003	39.33538735	2.1129	9.55E-05	0.001092
i1_HQ_YHS_c23730/f2p0/1758	156.8032875	11.88864603	3.7213	9.57E-05	0.0010936
i0_HQ_YHS_c351/f2p0/901	348.602743	1352.637325	-1.9561	9.62E-05	0.0010996
i2_HQ_YHS_c2456/f6p4/2665	137.3143444	503.7400094	-1.8752	9.63E-05	0.0011
i1_HQ_YHS_c1016/f17p0/1838	3970.750889	719.1583731	2.465	9.64E-05	0.0011011
i3_LQ_YHS_c18338/f1p0/3700	20.42019528	235.8139387	-3.5296	9.65E-05	0.0011013
i4_LQ_YHS_c4881/f1p4/4644	0	18.43745403	-Inf	9.65E-05	0.0011013
i4_LQ_YHS_c9458/f1p0/5030	5.432993971	55.38254496	-3.3496	9.65E-05	0.0011013
i3_LQ_YHS_c19783/f1p0/3588	63.84947011	1.492266529	5.4191	9.66E-05	0.0011027
i1_LQ_YHS_c23103/f1p9/1480	0.544682597	25.15605076	-5.5293	9.70E-05	0.0011066
i1_LQ_YHS_c32372/f1p0/1697	158.4220863	34.19807816	2.2118	9.72E-05	0.0011089
i4_LQ_YHS_c4299/f1p0/4812	1.993672628	36.2801984	-4.1857	9.75E-05	0.0011116
i4_LQ_YHS_c9847/f1p0/4481	400.0639895	111.8801829	1.8383	9.76E-05	0.0011131
i1_LQ_YHS_c2242/f4p1/1815	197.6097886	715.4177783	-1.8561	9.77E-05	0.0011138
i1_LQ_YHS_c4053/f1p0/1483	0.817023896	27.79729643	-5.0884	9.77E-05	0.0011138
i2_HQ_YHS_c18885/f3p4/2135	319.0018044	1236.398562	-1.9545	9.79E-05	0.0011152
i1_LQ_YHS_c19905/f1p0/1869	265.2432623	981.2845137	-1.8874	9.81E-05	0.0011177
i2_LQ_YHS_c22267/f1p2/2939	169.2303588	0	Inf	9.84E-05	0.001121
i0_LQ_YHS_c1947/f1p4/896	2.40575926	39.6658781	-4.0433	9.86E-05	0.0011224
i0_LQ_YHS_c157/f5p0/754	414.5655676	23951.62262	-5.8524	9.87E-05	0.0011228
i1_LQ_YHS_c23572/f2p0/1871	75.16351051	9.935121143	2.9194	9.87E-05	0.0011233
i2_LQ_YHS_c28978/f1p1/2063	92.79732357	342.6024332	-1.8844	9.89E-05	0.0011247
i2_LQ_YHS_c39370/f1p0/2282	108.578515	18.66565524	2.5403	9.91E-05	0.0011271
i1_LQ_YHS_c22652/f1p0/1407	33.66047043	1.466121141	4.521	9.92E-05	0.0011276
i2_LQ_YHS_c40754/f1p0/2157	79.45661023	293.3944075	-1.8846	9.93E-05	0.0011288
i3_LQ_YHS_c4296/f1p3/3188	81.65659486	10.37189268	2.9769	9.94E-05	0.0011292
i3_HQ_YHS_c2133/f3p0/3871	334.765753	2157.224912	-2.688	9.96E-05	0.0011318
i3_LQ_YHS_c12091/f1p3/3273	0	38.88238228	-Inf	9.98E-05	0.0011338
i2_LQ_YHS_c29418/f1p5/2047	184.6619528	644.692897	-1.8037	1.00E-04	0.001135
i4_LQ_YHS_c6144/f1p1/4346	135.2426152	27.66376491	2.2895	1.00E-04	0.001135
i2_LQ_YHS_c64713/f1p6/2025	56.44942273	1.112248908	5.6654	1.00E-04	0.0011351
i1_LQ_YHS_c29443/f6p1/1720	2185.634024	294.0618338	2.8939	0.00010041	0.0011395
i1_LQ_YHS_c5426/f1p0/1460	93.36473479	342.4633332	-1.875	0.0001005	0.0011403
i2_LQ_YHS_c33614/f1p1/2470	131.8681874	26.27998985	2.3271	0.00010096	0.0011452
i1_HQ_YHS_c40729/f3p0/1173	323.1362729	1212.767072	-1.9081	0.00010124	0.0011481
i2_LQ_YHS_c29267/f1p3/2071	1014.397622	90.1337466	3.4924	0.00010168	0.0011527
i2_LQ_YHS_c3690/f1p0/3024	100.259287	364.414903	-1.8618	0.00010169	0.0011527
i3_LQ_YHS_c18966/f1p0/3338	140.8572717	503.305531	-1.8372	0.00010171	0.0011527
i1_LQ_YHS_c42048/f1p1/1397	1066.253178	285.5620182	1.9007	0.00010197	0.0011554
i2_LQ_YHS_c42146/f1p4/2572	4.373031655	51.96969363	-3.571	0.00010206	0.0011561
i1_LQ_YHS_c18877/f1p1/1469	5.633155874	56.26280251	-3.3202	0.00010269	0.0011629
i2_LQ_YHS_c7425/f1p0/2568	355.3435898	96.24025414	1.8845	0.00010292	0.0011654
i2_LQ_YHS_c11194/f1p0/2113	18.31237214	104.8087798	-2.5169	0.00010333	0.0011696
i3_LQ_YHS_c13260/f1p0/3879	119.9465694	441.634565	-1.8805	0.00010337	0.0011698

i1_HQ_YHS_c9826/f2p5/1976	1485.390293	377.8751186	1.9749	0.00010348	0.0011709
i2_LQ_YHS_c24358/f1p1/2044	513.5178203	15.68716367	5.0328	0.00010355	0.001171
i2_LQ_YHS_c43380/f1p99/2264	4338.923048	1.961305157	11.111	0.00010354	0.001171
i5_LQ_YHS_c1901/f1p1/5122	7.426666599	63.40537414	-3.0938	0.00010382	0.0011739
i3_LQ_YHS_c4753/f1p0/3731	0	60.57309095	-Inf	0.00010501	0.001187
i2_LQ_YHS_c64793/f1p6/2009	3240.845887	411.0295117	2.9791	0.00010505	0.0011871
i4_LQ_YHS_c4835/f1p0/4870	154.7813142	33.5933369	2.204	0.0001051	0.0011875
i2_LQ_YHS_c64474/f1p6/2011	279.9195918	1018.920283	-1.864	0.00010565	0.0011935
i2_LQ_YHS_c24135/f1p1/2121	223.7139416	823.5225411	-1.8802	0.00010589	0.0011959
i1_LQ_YHS_c12591/f1p3/1624	112.4019119	13.74927954	3.0312	0.00010599	0.0011967
i3_LQ_YHS_c7704/f1p22/3637	131.5671745	4.752235657	4.791	0.00010618	0.0011986
i1_LQ_YHS_c3945/f1p0/1933	341.1197324	72.10964308	2.242	0.00010632	0.0011998
i1_LQ_YHS_c33344/f1p0/1385	221.4692143	815.3973927	-1.8804	0.00010665	0.0012033
i3_LQ_YHS_c8535/f1p2/3886	71.22031685	9.645783096	2.8843	0.00010733	0.0012107
i3_HQ_YHS_c2426/f2p1/3321	135.0374113	455.084827	-1.7528	0.00010747	0.001212
i3_LQ_YHS_c5750/f1p2/3462	80.92115439	305.8716019	-1.9183	0.00010751	0.0012122
i3_LQ_YHS_c5715/f1p6/3820	313.8830115	26.26162473	3.5792	0.00010777	0.0012148
i2_LQ_YHS_c39790/f1p0/2702	59.49994736	6.394187016	3.2181	0.00010797	0.0012165
i3_LQ_YHS_c3864/f1p0/3084	186.8019123	694.5872195	-1.8946	0.00010797	0.0012165
i4_LQ_YHS_c7676/f1p5/4587	162.5068085	587.6010224	-1.8543	0.00010814	0.0012181
i1_LQ_YHS_c39595/f1p1/1223	154.6506019	29.95836539	2.368	0.0001082	0.0012185
i2_LQ_YHS_c63298/f1p4/2080	459.3664353	128.4676674	1.8382	0.00010848	0.0012213
i2_LQ_YHS_c50814/f1p3/2201	365.4583809	99.84797379	1.8719	0.00010871	0.0012237
i5_LQ_YHS_c4016/f1p2/5197	67.30548966	8.475121707	2.9894	0.00010876	0.001224
i3_HQ_YHS_c21118/f5p1/3430	394.8689291	73.99590942	2.4159	0.00010891	0.0012253
i2_LQ_YHS_c18951/f1p0/2434	49.43669295	2.353566188	4.3927	0.00010927	0.0012291
i6_LQ_YHS_c862/f1p0/6425	122.9667086	17.43815972	2.8179	0.00010993	0.0012363
i3_LQ_YHS_c11292/f1p0/3142	23.3597994	180.7653274	-2.952	0.00011003	0.0012368
i3_LQ_YHS_c13481/f1p2826/3077	3.90386977	48.31691024	-3.6296	0.00011	0.0012368
i1_LQ_YHS_c10987/f1p0/1787	63.68558758	253.0758743	-1.9905	0.00011027	0.0012392
i2_HQ_YHS_c13584/f3p0/2687	97.02252705	353.3849938	-1.8648	0.00011105	0.0012477
i2_LQ_YHS_c39354/f1p0/2492	0	18.0897035	-Inf	0.00011183	0.0012562
i1_LQ_YHS_c19249/f1p1/1997	22.16920736	138.6001021	-2.6443	0.00011186	0.0012563
i2_LQ_YHS_c24754/f1p0/2514	0	20.70812091	-Inf	0.00011223	0.0012601
i2_LQ_YHS_c53774/f1p1/2158	2.517373857	106.7330805	-5.4059	0.00011226	0.0012602
i2_HQ_YHS_c12747/f3p0/2288	65.52441911	253.5222044	-1.952	0.00011242	0.0012616
i1_LQ_YHS_c25422/f1p0/2007	0.544682597	195.3814333	-8.4867	0.00011275	0.001265
i1_LQ_YHS_c8065/f1p18/1981	2.610534621	39.34482619	-3.9138	0.0001128	0.0012653
i2_LQ_YHS_c7185/f1p0/2489	208.2342467	754.9591265	-1.8582	0.00011294	0.0012666
i2_LQ_YHS_c56278/f1p1/2541	215.2717168	53.27048918	2.0148	0.00011312	0.0012683
i2_LQ_YHS_c37074/f1p1/2469	1.623557107	31.4656799	-4.2765	0.00011372	0.0012747
i4_LQ_YHS_c2627/f1p2/4929	11.53531173	172.5482968	-3.9029	0.00011401	0.0012777
i5_LQ_YHS_c2025/f1p2/5091	97.14791623	0	Inf	0.00011419	0.0012794
i1_LQ_YHS_c32589/f1p3/1455	191.243052	45.7028362	2.0651	0.00011498	0.0012876
i1_LQ_YHS_c9189/f1p1/1690	106.085897	440.9520666	-2.0554	0.000115	0.0012876
i4_LQ_YHS_c3580/f1p3/4950	0	24.88168048	-Inf	0.000115	0.0012876
i3_HQ_YHS_c10484/f2p6/3262	2109.933946	492.9913778	2.0976	0.00011507	0.0012881
i1_LQ_YHS_c13006/f1p1/1986	63.58273316	414.3075274	-2.704	0.00011534	0.0012908
i2_LQ_YHS_c58664/f1p0/2021	0	17.89775951	-Inf	0.00011547	0.0012919
i4_LQ_YHS_c13010/f1p0/4097	18.64847133	129.9405426	-2.8007	0.00011559	0.001293
i1_HQ_YHS_c7023/f2p0/1952	0	17.86549241	-Inf	0.00011609	0.0012983
i1_LQ_YHS_c9483/f1p0/2022	21.39461293	144.2509198	-2.7533	0.00011638	0.0013012
i1_HQ_YHS_c13727/f4p0/1526	1384.248739	334.717085	2.0481	0.00011649	0.0013022
i2_LQ_YHS_c54287/f1p1/2237	906.6539353	178.5370729	2.3443	0.00011698	0.0013074
i2_LQ_YHS_c44724/f1p2/2048	1112.230701	182.636899	2.6064	0.00011702	0.0013075
i1_LQ_YHS_c32687/f1p2/1648	675.0358968	24.62081938	4.777	0.0001172	0.0013092
i1_LQ_YHS_c24976/f1p0/1494	229.0729395	56.97386411	2.0074	0.00011757	0.001313
i1_LQ_YHS_c26015/f1p0/1427	776.2970536	4183.452144	-2.43	0.00011773	0.0013146
i4_LQ_YHS_c6041/f1p1/4678	39.72334461	175.9612344	-2.1472	0.00011786	0.0013156
i1_LQ_YHS_c6612/f1p2/1494	152.1093601	496.8808123	-1.7078	0.0001181	0.0013178
i3_LQ_YHS_c17867/f1p0/3089	6.289453068	54.8885465	-3.1255	0.00011808	0.0013178
i1_LQ_YHS_c19705/f1p3/1680	24.15538357	121.6957943	-2.3329	0.00011842	0.0013207
i4_LQ_YHS_c13306/f1p2/4154	326.1565031	87.66225012	1.8955	0.00011842	0.0013207
i3_LQ_YHS_c6516/f1p0/3370	18.8436811	108.6263751	-2.5272	0.00011849	0.0013211
i0_LQ_YHS_c2051/f1p3/783	412.9934167	1689.226095	-2.0322	0.0001186	0.001322
i4_LQ_YHS_c6127/f1p4/4674	546.447958	153.900893	1.8281	0.00011863	0.0013221
i3_LQ_YHS_c5001/f1p2/3420	211.225573	768.3541817	-1.863	0.00011911	0.0013272
i3_LQ_YHS_c18030/f1p0/3675	263.1366778	966.0470753	-1.8763	0.00011917	0.0013275
i1_HQ_YHS_c40425/f31p0/1613	887.3221094	8280.600387	-3.2222	0.00011923	0.0013279
i3_LQ_YHS_c18315/f1p0/3774	17.04716773	99.28829514	-2.5421	0.00011946	0.0013302
i3_LQ_YHS_c19182/f1p2/4052	184.0876233	658.4957678	-1.8388	0.00011958	0.0013312
i2_LQ_YHS_c35234/f1p0/2962	3.237887298	59.29231909	-4.1947	0.00011962	0.0013313
i3_LQ_YHS_c12904/f1p8/3629	0	18.07746009	-Inf	0.0001197	0.0013315
i4_LQ_YHS_c9595/f1p2/4809	10.45262917	123.6861285	-3.5647	0.00011967	0.0013315
i1_LQ_YHS_c10669/f1p5/1401	476.5045054	131.9463187	1.8525	0.00012025	0.0013374
i3_LQ_YHS_c17030/f1p0/3189	45.55778972	292.863202	-2.6845	0.00012036	0.0013383
i0_LQ_YHS_c602/f1p0/601	508.1321289	103.6171091	2.2939	0.0001206	0.0013407
i2_LQ_YHS_c56338/f1p7/3006	10.50717689	67.78150208	-2.6895	0.00012073	0.0013418
i1_LQ_YHS_c26101/f1p0/1963	46.62792917	193.4994335	-2.0531	0.00012104	0.001345
i3_HQ_YHS_c20108/f5p0/3031	0.771711468	831.2071051	-10.073	0.00012151	0.0013499
i1_LQ_YHS_c11237/f1p2/2016	1261.730373	299.1930142	2.0763	0.00012184	0.0013532
i1_HQ_YHS_c29546/f42p0/1851	4837.297576	799.528543	2.597	0.00012191	0.0013534
i2_LQ_YHS_c38570/f1p6/2540	154.6658748	549.7750367	-1.8297	0.00012191	0.0013534
i2_LQ_YHS_c49906/f1p30/2316	758.0402294	0	Inf	0.00012237	0.0013581
i2_LQ_YHS_c18776/f1p5/2570	1006.48003	218.844383	2.2013	0.00012246	0.0013588
i1_LQ_YHS_c3061/f1p0/1955	1.361706493	31.63586143	-4.5381	0.00012302	0.0013647
i2_LQ_YHS_c3135/f1p5/2290	72.86507431	272.3899945	-1.9024	0.00012305	0.0013647
i4_LQ_YHS_c3420/f1p2/4812	470.7120482	68.52100108	2.7802	0.00012322	0.0013663
i2_LQ_YHS_c34398/f1p8/2088	315.8661683	1194.301778	-1.9188	0.00012341	0.0013681

i2_LQ_YHS_c21513/f1p4/2716	64.73218463	389.2199207	-2.588	0.00012349	0.0013687
i1_LQ_YHS_c37066/f1p2/1821	892.2955525	243.3018155	1.8748	0.00012376	0.0013714
i3_LQ_YHS_c9413/f1p0/3286	0	35.50558819	-Inf	0.00012411	0.001375
i0_LQ_YHS_c2609/f1p11/532	53.10624805	214.6240616	-2.0149	0.00012423	0.0013759
i3_HQ_YHS_c1830/f4p0/4016	472.3685721	83.61053076	2.4982	0.00012429	0.0013764
i1_LQ_YHS_c20740/f1p3/1593	18.98757316	106.897654	-2.4931	0.00012457	0.0013788
i1_LQ_YHS_c6972/f1p2/1742	0	17.44767877	-Inf	0.00012455	0.0013788
i1_LQ_YHS_c7808/f1p0/1776	105.4464847	5.615193882	4.231	0.00012499	0.0013831
i2_LQ_YHS_c13660/f1p5/2887	185.7984807	653.5369725	-1.8145	0.00012521	0.0013853
i3_LQ_YHS_c6310/f1p7/3592	3.540436881	157.9521729	-5.4794	0.00012543	0.0013874
i1_LQ_YHS_c39277/f1p0/1311	150.4356318	641.6664114	-2.0927	0.00012568	0.0013898
i2_LQ_YHS_c15258/f1p0/2086	103.1793842	368.3236915	-1.8358	0.0001259	0.0013916
i3_LQ_YHS_c14162/f1p1/3057	176.0768807	602.6360077	-1.7751	0.0001259	0.0013916
i1_LQ_YHS_c18749/f1p1/1913	285.5110315	1054.816607	-1.8854	0.00012599	0.0013922
i2_LQ_YHS_c34583/f1p0/2203	3.257604899	41.81806146	-3.6822	0.00012608	0.001393
i4_LQ_YHS_c12695/f1p2/4197	201.2611304	49.34061116	2.0282	0.00012618	0.0013938
i1_LQ_YHS_c13163/f1p0/1559	0	17.35253606	-Inf	0.00012658	0.0013978
i1_HQ_YHS_c40307/f20p0/1422	1896.909205	107.376565	4.1429	0.00012663	0.001398
i2_HQ_YHS_c21633/f2p3/2831	496.4375877	144.4150598	1.7814	0.00012672	0.0013988
i1_LQ_YHS_c20396/f1p0/1650	9.953258998	72.96154345	-2.8739	0.00012721	0.0014038
i2_LQ_YHS_c44967/f1p0/2021	123.9585604	14.50200753	3.0955	0.00012754	0.0014072
i3_LQ_YHS_c2773/f1p0/3938	29.75578687	160.174344	-2.4284	0.00012758	0.0014073
i3_LQ_YHS_c11311/f1p0/3129	130.9749894	494.0877129	-1.9155	0.00012765	0.0014077
i4_LQ_YHS_c13704/f1p0/4264	223.1278121	804.0771049	-1.8495	0.00012795	0.0014106
i2_LQ_YHS_c39491/f1p6/2446	261.3157137	65.84365863	1.9887	0.00012836	0.0014149
i2_LQ_YHS_c36733/f1p0/2126	88.84178904	14.57159763	2.6081	0.00012842	0.0014152
i1_LQ_YHS_c4168/f1p2/1514	0	35.91009263	-Inf	0.00012884	0.0014195
i1_LQ_YHS_c31854/f1p0/1636	1532.755494	387.822882	1.9827	0.00012907	0.0014218
i2_LQ_YHS_c3153/f1p1/2151	102.3271747	381.7271928	-1.8994	0.00012939	0.0014249
i2_LQ_YHS_c28845/f1p0/2046	137.0506855	4.802867866	4.8347	0.00012961	0.0014271
i2_LQ_YHS_c34434/f1p1/2947	0	18.81747165	-Inf	0.00013005	0.0014315
i5_HQ_YHS_c706/f2p0/5285	253.2528408	3.395159205	6.221	0.00013007	0.0014315
i1_LQ_YHS_c33792/f1p0/1846	2.32308918	36.84382651	-3.9873	0.00013019	0.0014321
i3_LQ_YHS_c12630/f1p0/3176	0	33.62106063	-Inf	0.00013018	0.0014321
i1_HQ_YHS_c39909/f2p3/1961	311.3637875	11.54148828	4.7537	0.00013049	0.0014351
i2_HQ_YHS_c29876/f6p4/2793	20.45214244	300.8641851	-3.8788	0.00013085	0.0014387
i2_LQ_YHS_c41741/f1p0/2247	0	17.68519906	-Inf	0.00013093	0.0014393
i2_LQ_YHS_c12111/f1p5/2897	166.9495004	582.4901377	-1.8028	0.0001311	0.0014402
i2_LQ_YHS_c36201/f1p0/2536	19.32748877	0	Inf	0.00013109	0.0014402
i2_LQ_YHS_c41188/f1p0/2382	0	17.67741879	-Inf	0.0001311	0.0014402
i3_LQ_YHS_c7147/f1p0/3725	70.30283801	274.9035909	-1.9673	0.00013123	0.0014413
i3_LQ_YHS_c6619/f1p2/3171	0	17.65905367	-Inf	0.0001315	0.0014439
i2_LQ_YHS_c52023/f1p5/2925	86.82301344	306.9903258	-1.822	0.00013165	0.0014451
i3_LQ_YHS_c10084/f1p4/3325	687.2793094	89.20194924	2.9457	0.00013167	0.0014451
i2_LQ_YHS_c3490/f1p1/2124	1.543422937	34.82138452	-4.4958	0.00013188	0.001447
i1_LQ_YHS_c21225/f1p1/1604	36.23109474	163.3663432	-2.1728	0.00013247	0.0014532
i2_LQ_YHS_c38536/f1p0/2215	69.56930635	262.5177095	-1.9159	0.00013249	0.0014532
i1_LQ_YHS_c21361/f1p2/1743	226.9996162	40.96683409	2.4702	0.00013311	0.0014596
i0_LQ_YHS_c231/f4p0/674	586.0843912	20655.03227	-5.1392	0.00013317	0.0014599
i2_LQ_YHS_c40947/f1p4/2510	479.093282	23258.89956	-5.6013	0.00013336	0.0014617
i2_LQ_YHS_c13779/f1p4/2534	0	34.57426496	-Inf	0.00013342	0.001462
i2_LQ_YHS_c11669/f1p1/2447	7.81903563	183.9110701	-4.5559	0.00013381	0.0014659
i2_LQ_YHS_c11822/f1p13/2798	114.5326491	487.7185279	-2.0903	0.00013393	0.0014669
i0_LQ_YHS_c3093/f1p1/476	1927.339543	6.566107325	8.1974	0.00013419	0.0014693
i4_LQ_YHS_c15746/f1p0/4041	41.00111725	2.879951507	3.8315	0.00013421	0.0014693
i2_LQ_YHS_c53109/f1p1/2846	73.77745621	280.1862822	-1.9251	0.00013507	0.0014784
i2_LQ_YHS_c6731/f1p11/2358	20.78777489	107.776253	-2.3742	0.00013541	0.0014818
i4_LQ_YHS_c10119/f1p0/4609	311.942946	1115.493916	-1.8383	0.00013573	0.0014849
i2_LQ_YHS_c43360/f1p1/2052	154.7536908	549.5546553	-1.8283	0.00013596	0.0014871
i2_LQ_YHS_c54523/f1p2/2234	196.3817731	685.3653695	-1.8032	0.00013638	0.0014914
i1_LQ_YHS_c37028/f1p0/1712	23.6954486	0.359993938	6.0405	0.00013667	0.0014942
i3_LQ_YHS_c3230/f1p0/3482	2.517373857	157.5854644	-5.9681	0.00013697	0.0014972
i1_HQ_YHS_c27161/f3p0/1335	728.1087316	3750.505836	-2.3649	0.00013714	0.0014987
i3_LQ_YHS_c18764/f1p23/3113	426.0430107	1.427732343	8.2211	0.0001372	0.001499
i2_HQ_YHS_c60845/f4p3/2219	323.1909642	74.26395802	2.1217	0.00013727	0.0014994
i4_LQ_YHS_c13104/f1p0/4075	13.40053512	189.0095186	-3.8181	0.00013755	0.0015021
i2_LQ_YHS_c38796/f1p1/2741	6.406944891	54.86674449	-3.0982	0.00013783	0.0015048
i1_LQ_YHS_c8724/f1p0/1499	11.77697801	159.4406492	-3.759	0.00013791	0.0015054
i1_LQ_YHS_c3556/f1p0/1588	317.0942461	1454.915	-2.198	0.00013804	0.0015064
i2_LQ_YHS_c50321/f1p1/2977	358.9370414	1359.083182	-1.9208	0.00013829	0.0015089
i2_HQ_YHS_c17770/f3p3/2739	45.42326939	190.6573966	-2.0695	0.00013843	0.0015099
i2_LQ_YHS_c25961/f1p1/2514	21.5213233	112.1400178	-2.3815	0.00013845	0.0015099
i2_LQ_YHS_c40767/f1p0/2722	18.89487913	0	Inf	0.00013866	0.0015118
i2_LQ_YHS_c6305/f1p3/2933	205.2172058	51.31190838	1.9998	0.00013884	0.0015135
i3_LQ_YHS_c7667/f1p2/3208	48.53813468	194.5988857	-2.0033	0.000139	0.0015149
i3_LQ_YHS_c18392/f1p7/3607	5.446825971	54.44837761	-3.3214	0.00013946	0.0015196
i2_LQ_YHS_c53698/f1p1/2682	0	24.0787933	-Inf	0.00013998	0.0015249
i3_LQ_YHS_c11438/f1p0/3110	9.621773272	70.19675833	-2.867	0.00014005	0.0015249
i8_LQ_YHS_c32/f1p0/8915	1.07887451	55.06042614	-5.6734	0.00014004	0.0015249
i2_HQ_YHS_c2160/f2p3/2569	93.62582067	788.359524	-3.0739	0.00014024	0.0015267
i1_HQ_YHS_c2428/f4p0/1773	110.8823282	394.3126848	-1.8303	0.00014095	0.0015337
i3_LQ_YHS_c8838/f1p0/3812	285.2913267	30.39560994	3.2305	0.00014095	0.0015337
i1_LQ_YHS_c9465/f1p0/1526	150.6022908	0	Inf	0.00014105	0.0015342
i2_LQ_YHS_c34774/f1p3/2476	526.7685921	150.8883956	1.8037	0.00014105	0.0015342
i3_LQ_YHS_c16757/f1p0/3214	53.54346277	211.9074239	-1.9847	0.0001411	0.0015343
i3_LQ_YHS_c12096/f1p0/3815	15.98928296	93.57898479	-2.5491	0.00014122	0.0015353
i1_LQ_YHS_c4907/f1p1/1997	13.11909496	88.97481494	-2.7617	0.00014183	0.0015415
i2_HQ_YHS_c15742/f4p1/2182	317.4589452	2.282910297	7.1196	0.00014201	0.0015431
i3_LQ_YHS_c16655/f1p0/3616	0	33.61493893	-Inf	0.0001423	0.001546

i4_LQ_YHS_c7209/f1p1/4812	656.1675124	3219.346606	-2.2946	0.00014234	0.0015461
i2_HQ_YHS_c2667/f2p6/2095	7.244950155	61.20970652	-3.0787	0.00014249	0.0015474
i4_LQ_YHS_c10813/f1p0/4846	0	18.72232894	-Inf	0.00014277	0.00155
i4_LQ_YHS_c4931/f1p3/4216	203.1984913	738.3130642	-1.8613	0.00014306	0.0015528
i2_LQ_YHS_c50967/f1p1/2574	136.2269203	472.8136361	-1.7953	0.00014322	0.0015542
i6_LQ_YHS_c205/f1p0/6090	48.19534449	197.824176	-2.0373	0.00014333	0.0015551
i1_LQ_YHS_c20333/f1p16/1928	0	28.30298507	-Inf	0.00014349	0.0015557
i2_HQ_YHS_c9631/f2p0/2525	0	28.30298507	-Inf	0.00014349	0.0015557
i3_LQ_YHS_c17986/f1p3/3213	453.7642069	128.5539245	1.8196	0.00014352	0.0015557
i4_LQ_YHS_c10609/f1p0/4396	71.50048486	10.06253095	2.829	0.00014342	0.0015557
i3_HQ_YHS_c7942/f2p0/3910	41.05865087	179.1281924	-2.1252	0.00014368	0.0015571
i2_LQ_YHS_c21752/f1p0/2772	35.35446805	204.080809	-2.5292	0.0001438	0.0015579
i4_LQ_YHS_c8589/f1p0/4255	15.28433202	93.30975064	-2.61	0.00014381	0.0015579
i4_LQ_YHS_c12637/f1p2/4890	66.18592456	8.547436165	2.953	0.00014393	0.0015588
i2_LQ_YHS_c48988/f1p11/2703	1584.426487	394.649307	2.0053	0.00014451	0.0015647
i2_LQ_YHS_c43069/f2p1/2021	166.5954966	5.162861804	5.012	0.00014461	0.0015654
i2_LQ_YHS_c13472/f1p1/2680	50.92002124	206.9004081	-2.0226	0.00014501	0.0015694
i2_LQ_YHS_c27118/f1p10/2235	179.9090283	901.3218434	-2.3248	0.00014555	0.0015749
i2_LQ_YHS_c54896/f1p1/2418	122.1335525	24.58069179	2.3129	0.00014576	0.0015768
i3_LQ_YHS_c5854/f1p0/3130	1237.900322	10.22280062	6.92	0.00014632	0.0015825
i1_LQ_YHS_c35267/f1p9/1381	408.7665916	112.9947969	1.855	0.00014672	0.0015865
i2_LQ_YHS_c8621/f1p0/2827	23.59765763	481.2126031	-4.35	0.0001468	0.001587
i2_LQ_YHS_c35006/f1p2/2918	1306.371464	253.7004871	2.3644	0.00014688	0.0015876
i2_LQ_YHS_c18160/f1p0/2281	0	24.54617336	-Inf	0.00014719	0.0015905
i1_LQ_YHS_c19367/f1p0/1733	0.544682597	23.69218096	-5.4429	0.00014761	0.0015947
i1_HQ_YHS_c2202/f6p0/1967	166.1824179	1304.663678	-2.9728	0.00014786	0.0015969
i1_LQ_YHS_c17099/f2p0/1730	112.3701419	395.4418391	-1.8152	0.00014788	0.0015969
i0_LQ_YHS_c2271/f1p34/408	89.06885978	1.769361165	5.6536	0.000148	0.0015978
i2_LQ_YHS_c25280/f1p3/2182	69.24462295	265.0844289	-1.9367	0.00014813	0.0015989
i1_HQ_YHS_c37611/f5p0/1088	429.6251434	4751.898499	-3.4674	0.00014849	0.0016017
i2_LQ_YHS_c42163/f1p1/2223	1583.270367	406.0798834	1.9631	0.00014848	0.0016017
i4_LQ_YHS_c11375/f1p8/4604	55.50960783	5.80773065	3.2567	0.00014848	0.0016017
i1_LQ_YHS_c33002/f1p2/1940	114.1885784	494.9237451	-2.1158	0.00014865	0.0016031
i2_LQ_YHS_c13597/f1p2/2899	215.5068859	54.98921814	1.9705	0.00014874	0.0016037
i2_LQ_YHS_c39763/f1p3/2190	4.630268811	164.4979093	-5.1508	0.00014881	0.0016041
i1_LQ_YHS_c24299/f1p5/1620	1751.448903	256.152312	2.7735	0.00014966	0.0016128
i4_LQ_YHS_c9843/f1p0/5017	584.2189201	2516.157081	-2.1066	0.00015039	0.0016204
i4_LQ_YHS_c8324/f1p5/4330	50.46804942	4.733870541	3.4143	0.00015058	0.0016221
i4_LQ_YHS_c10895/f1p4/4583	502.3468606	143.9664042	1.803	0.00015073	0.0016233
i2_HQ_YHS_c45865/f18p7/2220	1648.361506	429.9819818	1.9387	0.00015092	0.0016247
i3_HQ_YHS_c3800/f2p1/3984	271.6972872	42.77837419	2.667	0.0001509	0.0016247
i1_LQ_YHS_c24839/f1p0/1741	295.5672646	1101.680451	-1.8981	0.00015171	0.0016328
i3_LQ_YHS_c17365/f1p0/3249	64.21911052	247.8475322	-1.9484	0.0001518	0.0016334
i1_LQ_YHS_c4973/f2p0/1722	128.032239	27.01158881	2.2449	0.00015196	0.0016348
i1_LQ_YHS_c11630/f1p2/1784	52.53150198	0	Inf	0.00015206	0.0016354
i3_LQ_YHS_c11922/f1p0/3331	213.9747268	33.05135036	2.6947	0.00015233	0.001638
i2_LQ_YHS_c9738/f1p0/2551	0	23.38941395	-Inf	0.00015239	0.0016383
i1_LQ_YHS_c7958/f1p0/1843	57.54416492	291.9310454	-2.3429	0.00015251	0.0016389
i3_HQ_YHS_c2510/f2p4/3785	354.1467784	2.604515437	7.0872	0.00015252	0.0016389
i2_LQ_YHS_c56614/f1p3/2247	450.9177167	0	Inf	0.00015338	0.0016479
i1_LQ_YHS_c19885/f1p0/1703	69.56883962	256.5327296	-1.8826	0.00015345	0.0016482
i1_LQ_YHS_c33681/f1p6/1907	633.3376194	2.2061327	8.1653	0.00015399	0.0016537
i5_LQ_YHS_c2840/f1p0/5861	428.0883995	40.70581255	3.3946	0.00015405	0.001654
i2_LQ_YHS_c43333/f1p38/1838	660.5221358	3307.40069	-2.324	0.0001544	0.0016572
i5_LQ_YHS_c4696/f1p0/5045	0.817023896	211.5445402	-8.0164	0.00015442	0.0016572
i2_LQ_YHS_c13588/f1p0/2148	22.04294698	114.3344603	-2.3749	0.00015466	0.0016593
i2_LQ_YHS_c42001/f1p2/2706	470.0147277	1888.010056	-2.0061	0.00015469	0.0016593
i0_LQ_YHS_c1403/f1p2/470	534.6002725	47.73772159	3.4853	0.00015485	0.0016607
i4_LQ_YHS_c6782/f1p3/4727	311.8767802	84.33276998	1.8868	0.00015503	0.0016622
i0_LQ_YHS_c3926/f1p19/206	0	17.19167361	-Inf	0.00015567	0.0016687
i1_HQ_YHS_c41566/f2p4/1442	4110.795898	277.1808597	3.8905	0.0001563	0.0016751
i4_LQ_YHS_c9579/f1p11/4965	0	17.16552822	-Inf	0.00015635	0.0016753
i1_LQ_YHS_c35323/f1p0/1795	268.7793633	55.60347846	2.2732	0.00015658	0.0016774
i2_HQ_YHS_c25610/f2p1/2105	262.7719775	26.75515018	3.2959	0.00015663	0.0016775
i2_LQ_YHS_c52466/f1p1/2548	147.1196137	3.973835299	5.2103	0.00015678	0.0016788
i2_LQ_YHS_c50277/f1p3/2102	335.6298151	1254.062785	-1.9017	0.00015691	0.0016798
i2_HQ_YHS_c3535/f5p4/3045	194.3121369	694.6455963	-1.8379	0.00015735	0.0016841
i2_HQ_YHS_c45294/f4p0/2590	181.6426155	42.99381943	2.0789	0.00015823	0.0016931
i1_HQ_YHS_c16650/f7p0/1737	703.5408881	203.4210048	1.7902	0.00015836	0.0016942
i2_LQ_YHS_c48695/f1p2/2756	657.1938952	188.3644163	1.8028	0.00015852	0.0016955
i2_LQ_YHS_c26309/f1p4/3391	241.5808798	842.7302741	-1.8026	0.00015864	0.0016964
i3_LQ_YHS_c3465/f1p1/3345	76.0792421	9.62741798	2.9823	0.00016024	0.0017132
i7_LQ_YHS_c273/f1p0/7861	0.272341299	20.81163667	-6.2558	0.00016037	0.0017141
i1_LQ_YHS_c34945/f1p0/1441	64.1199361	240.0104116	-1.9043	0.00016059	0.0017161
i1_LQ_YHS_c19919/f1p0/1443	18.54735579	99.71396927	-2.4266	0.00016084	0.0017185
i5_HQ_YHS_c5139/f15p0/5051	957.2127256	7995.00476	-3.0622	0.00016101	0.0017198
i2_LQ_YHS_c49847/f1p4/2885	1.07887451	25.76478215	-4.5778	0.00016124	0.001722
i1_HQ_YHS_c7508/f2p0/1591	73.40386293	276.1201472	-1.9114	0.00016156	0.0017249
i1_LQ_YHS_c35838/f1p0/1503	155.8230333	2712.087867	-4.1214	0.00016159	0.0017249
i1_LQ_YHS_c23555/f1p0/1568	14.41103828	86.33530806	-2.5828	0.0001617	0.0017257
i2_HQ_YHS_c32587/f2p0/2575	0	42.03717708	-Inf	0.00016196	0.0017275
i2_LQ_YHS_c49353/f1p0/2695	0	42.03717708	-Inf	0.00016196	0.0017275
i6_LQ_YHS_c633/f1p0/6868	788.5693219	17928.27222	-4.5069	0.00016197	0.0017275
i2_LQ_YHS_c33725/f1p1/2988	81.05007874	296.5973437	-1.8716	0.00016221	0.0017296
i1_LQ_YHS_c5845/f1p7/1759	51.26694141	210.5713578	-2.0382	0.00016291	0.0017367
i2_LQ_YHS_c26598/f1p12/2590	356.1584081	1301.780593	-1.8699	0.00016304	0.0017377
i1_LQ_YHS_c28207/f1p0/1312	97.44621495	360.8678889	-1.8888	0.00016336	0.0017407
i1_HQ_YHS_c2642/f2p0/1662	366.0225597	1293.520401	-1.8213	0.00016355	0.0017423
i2_LQ_YHS_c6355/f1p1/2166	369.1525053	1417.138794	-1.9407	0.00016361	0.0017425

i3_LQ_YHS_c2641/f2p2/3382	29.83511564	164.3127122	-2.4614	0.00016363	0.0017425
i3_LQ_YHS_c9726/f1p0/3788	80.12592564	292.3746105	-1.8675	0.00016421	0.0017483
i3_LQ_YHS_c14561/f1p0/3056	257.9155307	67.77312903	1.9281	0.00016501	0.0017564
i1_LQ_YHS_c3776/f1p0/1972	356.1706042	1345.190632	-1.9172	0.00016555	0.0017618
i3_LQ_YHS_c5598/f1p0/3303	27.99837003	131.9668956	-2.2368	0.00016586	0.0017647
i1_LQ_YHS_c28441/f1p0/1351	67.49296364	252.1178129	-1.9013	0.00016606	0.0017664
i1_LQ_YHS_c38977/f1p2/1289	111.2764935	389.73869	-1.8084	0.0001674	0.0017802
i2_LQ_YHS_c10347/f1p0/2148	0	44.19738024	-Inf	0.00016883	0.001795
i1_LQ_YHS_c5850/f1p0/1886	0	22.65552409	-Inf	0.00016896	0.001796
i2_LQ_YHS_c53292/f1p1/2494	45.11391748	180.834005	-2.003	0.00016909	0.0017966
i3_LQ_YHS_c5046/f1p0/3753	6.048583822	56.13870984	-3.2143	0.00016905	0.0017966
i1_LQ_YHS_c9353/f1p7/1885	603.2856617	146.5691808	2.0413	0.00016926	0.0017981
i3_LQ_YHS_c2833/f1p1/3150	265.5452793	71.42038349	1.8946	0.00016969	0.0018018
i5_LQ_YHS_c3832/f1p0/5745	191.5712287	1505.239055	-2.974	0.00016969	0.0018018
i8_LQ_YHS_c78/f1p0/8229	12.16934704	80.89191472	-2.7327	0.00016993	0.0018039
i1_LQ_YHS_c34340/f1p0/1618	139.8078084	476.5565014	-1.7692	0.00017017	0.0018062
i1_LQ_YHS_c21016/f1p0/1792	772.2235917	27.12458295	4.8313	0.00017026	0.0018066
i2_LQ_YHS_c21318/f1p105/2345	3674.299774	0	Inf	0.00017042	0.001808
i1_LQ_YHS_c13688/f1p8/1342	94.80388635	1271.250964	-3.7452	0.00017056	0.0018086
i3_LQ_YHS_c6748/f1p0/3550	3.854749291	68.56108799	-4.1527	0.00017054	0.0018086
i3_HQ_YHS_c21105/f6p1/3118	201.6757864	9.209011561	4.4528	0.00017073	0.00181
i0_HQ_YHS_c474/f2p0/831	1417.360105	5.073840797	8.1259	0.00017077	0.0018101
i2_HQ_YHS_c60462/f7p2/2100	399.8956837	1508.801304	-1.9157	0.000171	0.0018121
i2_LQ_YHS_c12323/f1p30/2345	0.817023896	26.17256418	-5.0015	0.00017107	0.0018121
i3_LQ_YHS_c12670/f1p4/3876	15.93935708	92.3977781	-2.5353	0.00017106	0.0018121
i2_LQ_YHS_c22226/f1p0/2176	0	17.32520512	-Inf	0.0001712	0.0018127
i2_LQ_YHS_c53971/f1p0/2319	17.799967	106.9660981	-2.5872	0.00017121	0.0018127
i4_LQ_YHS_c11246/f1p0/4960	530.9869383	145.6860001	1.8658	0.00017159	0.0018164
i1_LQ_YHS_c36986/f1p0/1445	880.2987357	5088.743457	-2.5312	0.00017185	0.0018188
i1_LQ_YHS_c19024/f1p3/1705	123.4996702	2.879951507	5.4223	0.00017242	0.0018244
i1_LQ_YHS_c37624/f4p0/1152	423.3548992	25955.68542	-5.938	0.00017254	0.0018249
i3_LQ_YHS_c13556/f1p4/3407	120.8050486	420.7939783	-1.8004	0.00017251	0.0018249
i2_LQ_YHS_c24000/f1p17/2344	165.0147005	13.26128302	3.6373	0.00017274	0.0018266
i0_LQ_YHS_c2705/f1p1/698	2.615148079	46.17459919	-4.1421	0.00017286	0.0018273
i3_LQ_YHS_c17639/f1p3/3279	81.2783965	12.23086763	2.7323	0.00017288	0.0018273
i2_LQ_YHS_c3048/f1p11/2546	438.3074703	1749.531814	-1.997	0.00017325	0.0018308
i3_LQ_YHS_c18482/f1p3/3157	28.34255206	0	Inf	0.00017377	0.0018359
i2_LQ_YHS_c36808/f1p84/2300	2682.943701	1.498388234	10.806	0.00017436	0.0018416
i1_LQ_YHS_c26660/f1p0/1897	207.3426762	941.3660617	-2.1827	0.00017494	0.001847
i1_LQ_YHS_c39444/f1p5/1275	450.0050932	1682.399453	-1.9025	0.00017491	0.001847
i2_LQ_YHS_c38067/f1p3/2371	9.258807119	67.97166774	-2.876	0.00017503	0.0018475
i4_LQ_YHS_c2880/f1p4/4781	117.1377984	12.27537813	3.2544	0.00017508	0.0018477
i2_LQ_YHS_c55992/f1p1/2202	106.4304345	19.79966661	2.4264	0.00017542	0.0018509
i5_LQ_YHS_c3837/f1p0/5753	199.076159	51.16506767	1.9601	0.00017592	0.0018557
i1_LQ_YHS_c18910/f1p0/1861	98.3158622	572.9133497	-2.5428	0.00017605	0.0018567
i1_LQ_YHS_c15112/f4p0/1405	294.0343491	1061.249768	-1.8517	0.00017622	0.001858
i4_LQ_YHS_c10157/f1p1/4253	279.2714229	77.4171338	1.8509	0.00017629	0.0018585
i4_LQ_YHS_c13082/f1p0/4126	256.377766	67.75097377	1.92	0.00017697	0.0018652
i3_LQ_YHS_c13531/f2p0/3841	7.664186155	176.8559005	-4.5283	0.00017707	0.0018659
i2_LQ_YHS_c10381/f1p0/2557	336.483347	94.18048914	1.837	0.00017722	0.001867
i1_LQ_YHS_c19308/f1p2/2029	39.79091892	168.4134447	-2.0815	0.00017808	0.0018748
i1_LQ_YHS_c4990/f1p0/1884	169.5166146	40.92445516	2.0504	0.00017801	0.0018748
i2_LQ_YHS_c4212/f2p3/2817	19.5087301	537.0509314	-4.7829	0.00017807	0.0018748
i2_LQ_YHS_c36985/f1p1/2673	0	107.8604121	-Inf	0.00017833	0.001877
i1_HQ_YHS_c9134/f2p3/1722	312.6850188	88.0588534	1.8282	0.00017894	0.0018831
i2_LQ_YHS_c13644/f1p0/2315	28.49138787	134.8328254	-2.2426	0.00017914	0.0018848
i2_LQ_YHS_c49524/f1p5/2282	6.606301387	56.18499867	-3.0883	0.00017931	0.0018862
i1_LQ_YHS_c8470/f1p3/1888	1.748198298	31.87350148	-4.1884	0.00018033	0.0018961
i4_LQ_YHS_c7531/f1p0/4477	0	73.67288524	-Inf	0.0001803	0.0018961
i1_LQ_YHS_c10074/f1p3/1941	0	16.84672765	-Inf	0.00018068	0.0018993
i2_LQ_YHS_c24878/f1p1/2683	16.34222517	91.10484304	-2.4789	0.00018075	0.0018997
i2_LQ_YHS_c53508/f1p3/2656	42.50350255	179.944269	-2.0819	0.00018099	0.0019017
i2_LQ_YHS_c43798/f1p0/2040	0	16.8300211	-Inf	0.00018118	0.0019034
i2_HQ_YHS_c9724/f2p0/2113	510.314634	7.559872706	6.0769	0.00018198	0.0019114
i6_LQ_YHS_c575/f1p0/6236	69.81745303	259.1514607	-1.8921	0.00018286	0.0019202
i1_HQ_YHS_c10636/f3p0/1676	319.8874186	1152.724002	-1.8494	0.00018314	0.0019227
i1_LQ_YHS_c14470/f1p0/1115	510.2191036	2.539981251	7.6502	0.00018342	0.0019251
i2_LQ_YHS_c38587/f1p0/2530	24.62200117	122.2464665	-2.3118	0.00018353	0.0019259
i1_LQ_YHS_c36246/f1p3/1594	16.94478005	329.0333234	-4.2793	0.00018401	0.0019301
i4_LQ_YHS_c6740/f1p13/4292	478.6319624	1915.718506	-2.0009	0.000184	0.0019301
i1_HQ_YHS_c1445/f9p0/1566	251.0206637	871.6050114	-1.7959	0.00018431	0.0019329
i1_LQ_YHS_c7379/f1p0/1830	141.8006266	17.73583127	2.9991	0.00018444	0.0019338
i4_LQ_YHS_c4057/f1p1/4442	25.8569973	125.8919426	-2.2836	0.00018478	0.0019369
i2_HQ_YHS_c60405/f2p4/2272	37.34143292	177.9803988	-2.2529	0.00018499	0.0019387
i2_LQ_YHS_c33691/f1p43/3107	1.90638909	33.48930745	-4.1348	0.0001852	0.0019405
i1_LQ_YHS_c38262/f1p0/1327	147.8140296	10.30735849	3.842	0.00018526	0.0019407
i1_LQ_YHS_c26092/f1p0/1467	337.0740851	94.57662053	1.8335	0.00018592	0.0019472
i3_HQ_YHS_c11492/f2p0/3823	179.2083497	888.2673666	-2.3094	0.00018604	0.0019481
i2_LQ_YHS_c23828/f1p2/2744	32.60511319	145.2968227	-2.1558	0.00018612	0.0019484
i2_LQ_YHS_c12589/f1p3/2579	7.300761641	124.9642969	-4.0973	0.00018626	0.0019495
i5_LQ_YHS_c777/f1p0/5455	33.26936516	146.0082375	-2.1338	0.0001868	0.0019547
i2_LQ_YHS_c43413/f1p1/2107	785.3881008	225.2805166	1.8017	0.00018717	0.0019581
i1_LQ_YHS_c26222/f1p0/1847	121.009502	9.215133266	3.715	0.00018752	0.0019614
i2_LQ_YHS_c18483/f1p1/2697	357.2331491	5.987431231	5.8988	0.00018822	0.0019683
i1_HQ_YHS_c40268/f7p0/1389	700.7554465	19055.41923	-4.7651	0.00018831	0.0019688
i1_LQ_YHS_c36532/f1p3/1627	204.8824123	673.1422905	-1.7161	0.00018854	0.0019708
i1_LQ_YHS_c6103/f1p4/1575	78.33542593	12.27198078	2.6743	0.00018858	0.0019708
i3_LQ_YHS_c11806/f1p0/3467	184.6602918	2047.463905	-3.4709	0.00018868	0.0019714
i2_LQ_YHS_c4455/f1p1/2478	0	16.5813232	-Inf	0.00018888	0.0019731

i0_LQ_YHS_c600/f1p0/972	576.1637363	2367.693856	-2.0389	0.00018901	0.001974
i2_LQ_YHS_c43607/f1p3/2063	566.2114707	133.4469989	2.0851	0.00018942	0.0019778
i3_LQ_YHS_c17792/f1p11/3835	17.08372835	95.80229705	-2.4874	0.00019008	0.0019843
i2_LQ_YHS_c20708/f1p2/2529	76.13362825	376.6050958	-2.3064	0.00019043	0.0019875
i2_LQ_YHS_c52409/f1p6/2166	713.6829399	3573.825297	-2.3241	0.0001907	0.0019899
i0_LQ_YHS_c1615/f1p0/874	1096.317089	111.0234662	3.3037	0.00019163	0.0019986
i1_LQ_YHS_c10442/f1p0/1680	531.1726231	30.20094159	4.1365	0.00019166	0.0019986
i2_LQ_YHS_c53392/f1p4/2485	50.13113646	199.0784888	-1.9896	0.00019161	0.0019986
i1_LQ_YHS_c28100/f1p6/1198	0.529578455	22.88483064	-5.4334	0.00019193	0.002001
i4_LQ_YHS_c8086/f1p1/4351	33.55218877	149.8714893	-2.1592	0.000192	0.0020013
i2_LQ_YHS_c9295/f1p0/2909	2.88287592	285.0454348	-6.6275	0.00019208	0.0020017
i3_LQ_YHS_c8362/f1p2/3239	151.3911911	545.0853991	-1.8482	0.00019212	0.0020017
i3_HQ_YHS_c10809/f2p0/3140	121.8025168	418.9730785	-1.7823	0.00019286	0.002009
i3_LQ_YHS_c13374/f1p0/3285	28.47341752	131.7842311	-2.2105	0.00019323	0.0020124
i1_LQ_YHS_c35042/f1p0/1852	186.0658698	639.6552607	-1.7815	0.00019362	0.0020152
i2_LQ_YHS_c3158/f1p1/2827	0	16.43669428	-Inf	0.00019353	0.0020152
i2_LQ_YHS_c50841/f1p4/2173	80.10331671	297.2965953	-1.892	0.00019363	0.0020152
i1_LQ_YHS_c23089/f1p0/1963	78.312817	290.4067513	-1.8908	0.00019394	0.0020181
i1_LQ_YHS_c17495/f1p6/1835	69.40538315	1.106127203	5.9715	0.00019448	0.0020233
i2_LQ_YHS_c41833/f1p1/2447	70.40556436	259.7515449	-1.8834	0.00019475	0.0020256
i1_LQ_YHS_c42471/f1p0/1405	0	16.39384234	-Inf	0.00019494	0.0020272
i1_LQ_YHS_c34684/f1p0/2019	602.1007554	2617.194916	-2.1199	0.0001956	0.0020336
i2_LQ_YHS_c37435/f1p1/2098	239.8463782	32.00584744	2.9057	0.00019583	0.0020355
i2_LQ_YHS_c48609/f1p12/2786	1005.476543	283.5721212	1.8261	0.00019614	0.0020383
i2_LQ_YHS_c51440/f1p4/2201	200.1267998	665.9967215	-1.7346	0.00019671	0.0020438
i3_HQ_YHS_c899/f14p0/3353	556.0356432	2391.173759	-2.1045	0.0001971	0.0020474
i0_HQ_YHS_c446/f2p0/859	1369.189103	368.8738105	1.8921	0.00019737	0.0020493
i2_LQ_YHS_c34276/f1p1/2415	305.5060189	1074.993404	-1.8151	0.00019733	0.0020493
i0_LQ_YHS_c2580/f1p2/526	0.272341299	28.65354017	-6.7172	0.00019763	0.0020516
i1_HQ_YHS_c1640/f9p0/1696	889.4851683	4869.65155	-2.4528	0.00019791	0.0020536
i2_LQ_YHS_c24156/f1p0/2072	0	100.5661845	-Inf	0.00019787	0.0020536
i1_LQ_YHS_c18344/f1p1/1689	185.3837839	622.4985895	-1.7476	0.00019818	0.0020556
i1_LQ_YHS_c32987/f1p0/1947	68.55766748	9.498349608	2.8516	0.00019823	0.0020556
i3_HQ_YHS_c21852/f3p2/3088	149.6708268	508.5934353	-1.7647	0.00019822	0.0020556
i2_LQ_YHS_c22627/f1p0/2814	0	33.68393625	-Inf	0.00019886	0.0020617
i3_LQ_YHS_c22492/f1p0/3041	53.09161901	214.6843315	-2.0157	0.00019902	0.002063
i2_LQ_YHS_c5395/f1p1/2722	75.65491753	269.9882068	-1.8354	0.00019915	0.0020638
i1_LQ_YHS_c24421/f1p1/1511	110.9579853	516.8284299	-2.2197	0.00019932	0.0020652
i1_HQ_YHS_c11159/f3p2/1592	317.8294723	1141.465085	-1.8446	0.0001996	0.0020676
i2_LQ_YHS_c55818/f1p2/2378	9.260537622	206.0682573	-4.4759	0.00019966	0.0020678
i1_HQ_YHS_c1652/f9p2/2008	1231.335539	340.811865	1.8532	0.00020001	0.002071
i1_LQ_YHS_c21965/f1p0/1486	336.9044295	87.85755079	1.9391	0.00020101	0.0020809
i2_LQ_YHS_c42258/f1p0/2781	81.85741736	291.11169	-1.8304	0.00020162	0.0020868
i1_HQ_YHS_c17060/f2p0/1825	114.9097034	23.42286659	2.2945	0.00020172	0.0020873
i2_LQ_YHS_c11764/f1p216/2691	501.3424345	44.51827284	3.4933	0.00020189	0.0020887
i2_HQ_YHS_c13365/f2p1/2535	28.55227117	133.7952225	-2.2283	0.00020199	0.0020893
i2_LQ_YHS_c20746/f1p0/2135	285.0742068	58.22576623	2.2916	0.0002024	0.002093
i0_LQ_YHS_c1988/f1p3/717	6989.571962	11.27936141	9.2754	0.00020249	0.0020936
i1_HQ_YHS_c41027/f109p6/1763	851.6816176	183.0282475	2.2182	0.00020262	0.0020945
i2_LQ_YHS_c64460/f1p4/2021	271.2958145	960.4820763	-1.8239	0.00020329	0.0021009
i1_HQ_YHS_c8504/f2p0/1840	141.724036	465.0000107	-1.7141	0.00020345	0.0021021
i2_HQ_YHS_c24534/f2p5/3016	87.32107797	310.4380153	-1.8299	0.00020443	0.0021118
i4_LQ_YHS_c4925/f1p0/5003	51.06296308	270.6649834	-2.4062	0.00020452	0.0021123
i2_LQ_YHS_c41108/f1p0/2588	27.58801391	169.2372225	-2.6169	0.00020466	0.0021133
i1_HQ_YHS_c2384/f2p0/1814	463.9858711	135.1234292	1.7798	0.00020497	0.002116
i2_HQ_YHS_c10980/f2p0/2117	188.0487466	47.00315874	2.0003	0.0002052	0.002118
i1_LQ_YHS_c12540/f1p7/1380	55.13486211	215.1996996	-1.9646	0.00020546	0.0021195
i1_LQ_YHS_c27611/f1p0/1158	154.7200479	0	Inf	0.00020549	0.0021195
i2_LQ_YHS_c49218/f1p0/2123	0	42.79721232	-Inf	0.00020541	0.0021195
i1_LQ_YHS_c21385/f1p0/1874	85.35049776	13.03987651	2.7105	0.00020615	0.0021259
i4_LQ_YHS_c4918/f1p0/4999	50.85025807	198.8840946	-1.9676	0.00020645	0.0021286
i1_LQ_YHS_c6298/f1p2/1736	18.42652265	129.5178334	-2.8133	0.00020754	0.0021393
i2_LQ_YHS_c26748/f1p0/2331	0	16.54680476	-Inf	0.00020828	0.0021465
i3_LQ_YHS_c2957/f1p0/3969	0	30.23202313	-Inf	0.00020939	0.0021575
i1_LQ_YHS_c36750/f1p2/1933	28.09233621	129.4485966	-2.2041	0.00020966	0.0021593
i2_LQ_YHS_c33894/f1p0/2122	0	17.72192929	-Inf	0.00020962	0.0021593
i1_LQ_YHS_c6659/f1p0/1967	0	16.50063569	-Inf	0.00020988	0.0021612
i4_LQ_YHS_c13924/f1p0/4139	216.3453496	733.3905767	-1.7612	0.00021005	0.0021624
i1_HQ_YHS_c31580/f2p0/1973	318.131402	1136.787663	-1.8373	0.00021035	0.0021644
i3_HQ_YHS_c9184/f2p0/3425	171.0701106	577.5219382	-1.7553	0.00021038	0.0021644
i3_LQ_YHS_c14747/f1p0/3033	166.9460561	548.0305985	-1.7149	0.0002103	0.0021644
i2_HQ_YHS_c32574/f2p1/2232	570.0320122	62.87002299	3.1806	0.00021108	0.0021712
i4_HQ_YHS_c2497/f2p0/4342	21.25739513	111.5291153	-2.3914	0.00021138	0.0021734
i4_LQ_YHS_c7468/f1p4/4466	6.188329155	62.55683045	-3.3375	0.00021137	0.0021734
i2_LQ_YHS_c5683/f1p4/2942	325.21603	85.02878472	1.9354	0.00021157	0.0021748
i3_LQ_YHS_c19356/f1p0/3077	510.3813753	6208.948372	-3.6047	0.00021173	0.002176
i2_LQ_YHS_c19379/f1p0/2819	0	16.44388178	-Inf	0.00021187	0.002177
i1_LQ_YHS_c22675/f1p0/1764	111.4340237	380.6816146	-1.7724	0.00021192	0.0021771
i2_LQ_YHS_c11000/f1p0/2354	19.2561064	101.6875574	-2.4008	0.00021198	0.0021772
i2_LQ_YHS_c18577/f1p0/2805	58.35973956	218.1650353	-1.9024	0.00021274	0.0021845
i1_LQ_YHS_c14584/f1p0/1339	8549.658468	740.2020994	3.5299	0.00021293	0.002186
i1_LQ_YHS_c36880/f1p4/1816	469.4607332	137.0317706	1.7765	0.00021332	0.0021896
i1_HQ_YHS_c23781/f2p14/1917	34.2428326	174.1436457	-2.3464	0.00021359	0.0021919
i1_HQ_YHS_c8401/f2p3/1565	234.6276295	772.0953482	-1.7184	0.00021381	0.0021937
i2_LQ_YHS_c3822/f1p3/2740	74.47016084	278.5975281	-1.9034	0.00021425	0.0021977
i1_LQ_YHS_c7136/f1p1/1626	51.43738689	194.790316	-1.921	0.0002144	0.0021988
i2_LQ_YHS_c34191/f1p3/2715	145.0267176	495.4092447	-1.7723	0.00021445	0.0021989
i2_LQ_YHS_c58109/f1p2/2408	137.174471	461.9077707	-1.7516	0.00021481	0.0022016
i3_LQ_YHS_c17806/f1p1/3778	69.68646788	290.7016962	-2.0606	0.00021481	0.0022016

i2_LQ_YHS_c37058/f1p1/2484	81.45356675	0	Inf	0.00021531	0.0022061
i5_LQ_YHS_c2655/f1p2/5370	159.3563495	38.98317369	2.0313	0.00021535	0.0022061
i2_LQ_YHS_c11192/f1p1/2962	53.25487325	209.1425992	-1.9735	0.00021553	0.0022075
i2_LQ_YHS_c14081/f1p0/2078	203.048789	15.44127034	3.717	0.00021567	0.0022085
i1_LQ_YHS_c43850/f1p0/1033	417.269902	1530.197508	-1.8747	0.00021601	0.0022116
i2_LQ_YHS_c7192/f1p0/2272	56.22504108	215.6401427	-1.9393	0.00021662	0.0022173
i4_LQ_YHS_c10401/f1p1/4800	217.2495577	762.2915836	-1.811	0.00021676	0.0022183
i0_LQ_YHS_c423/f2p0/947	5390.586561	27.35053282	7.6227	0.00021712	0.0022215
i3_LQ_YHS_c12150/f1p5/3393	56.26955648	7.060698564	2.9945	0.00021758	0.0022258
i2_LQ_YHS_c54934/f1p1/2156	0.904307434	60.96456527	-6.075	0.00021769	0.0022263
i6_LQ_YHS_c295/f1p0/6176	126.4776145	439.5074612	-1.797	0.00021816	0.0022307
i2_HQ_YHS_c30121/f30p3/2167	2412.685688	335.2925648	2.8471	0.00021825	0.0022312
i1_LQ_YHS_c14254/f1p0/1295	2822.280143	630.2726714	2.1628	0.00021847	0.0022325
i1_LQ_YHS_c4702/f1p13/1539	157.5300084	36.43434637	2.1123	0.00021847	0.0022325
i1_LQ_YHS_c19365/f1p0/1901	140.8307195	7.811887745	4.1721	0.00021861	0.0022334
i1_LQ_YHS_c11214/f1p2/1968	381.0744381	1417.714537	-1.8954	0.00021884	0.0022353
i1_LQ_YHS_c24473/f1p3/1779	841.0521034	229.4612637	1.8739	0.00021895	0.002236
i1_LQ_YHS_c28894/f1p0/1332	39.19917664	168.4730043	-2.1036	0.00021905	0.0022365
i1_HQ_YHS_c29169/f25p1/1703	4964.329492	869.4417291	2.5134	0.00021938	0.0022394
i1_LQ_YHS_c2564/f2p5/1794	182.2197539	1.492266529	6.932	0.00021957	0.0022404
i2_LQ_YHS_c41647/f1p0/2606	51.05147313	266.8919849	-2.3862	0.00021953	0.0022404
i4_HQ_YHS_c2361/f2p7/4941	0	42.78496891	-Inf	0.00021987	0.002243
i1_LQ_YHS_c34966/f1p0/1977	767.4900796	3917.456892	-2.3517	0.0002201	0.0022449
i2_LQ_YHS_c60398/f1p1/2642	124.483736	22.3228611	2.4794	0.00022056	0.0022489
i4_LQ_YHS_c12149/f1p14/4534	324.45937	94.97326448	1.7724	0.00022059	0.0022489
i1_LQ_YHS_c32287/f1p0/1809	309.5122118	54.24754801	2.5124	0.00022099	0.0022525
i4_LQ_YHS_c3588/f1p1/4594	55.32232771	212.0895758	-1.9387	0.00022165	0.0022587
i5_LQ_YHS_c4330/f1p0/5126	257.2720183	782.5480503	-1.6049	0.00022178	0.0022596
i1_LQ_YHS_c20288/f1p0/1677	283.9627078	62.47776196	2.1843	0.00022222	0.0022636
i3_LQ_YHS_c13128/f1p0/3542	4266.910638	808.2726687	2.4003	0.00022242	0.0022651
i1_HQ_YHS_c17104/f2p0/1762	3713.941254	753.2179297	2.3018	0.00022253	0.0022657
i1_LQ_YHS_c10302/f1p0/1492	390.7160534	1393.40029	-1.8344	0.00022261	0.0022657
i3_LQ_YHS_c9359/f1p7/3789	22.96489446	131.0214704	-2.5123	0.00022258	0.0022657
i2_LQ_YHS_c20912/f1p3/2504	16.68005485	121.3025477	-2.8624	0.00022306	0.0022698
i2_LQ_YHS_c44254/f1p0/2068	39.65048787	172.1041834	-2.1179	0.00022323	0.002271
i5_LQ_YHS_c3477/f1p0/5575	17.5738716	0	Inf	0.00022374	0.0022757
i2_LQ_YHS_c39372/f1p0/2924	325.9106039	32.1344428	3.3423	0.00022393	0.0022772
i2_LQ_YHS_c56298/f1p3/2776	600.5300035	177.9379002	1.7549	0.00022405	0.0022779
i3_LQ_YHS_c11529/f1p3/3701	0	27.519136	-Inf	0.00022435	0.0022805
i3_LQ_YHS_c9027/f1p2/3691	297.1380726	62.06263313	2.2593	0.00022448	0.0022813
i1_LQ_YHS_c36604/f1p0/1630	231.6574293	796.6813749	-1.782	0.00022453	0.0022814
i2_HQ_YHS_c8007/f3p3/2140	717.3614119	126.5725957	2.5027	0.0002247	0.0022826
i3_LQ_YHS_c14362/f1p0/3053	0	31.78716528	-Inf	0.00022522	0.0022874
i2_LQ_YHS_c18457/f1p2/2630	46.01373952	4.501286408	3.3537	0.00022591	0.002294
i3_HQ_YHS_c21321/f5p0/3108	82.3446944	289.5894891	-1.8143	0.00022638	0.0022982
i1_LQ_YHS_c36315/f1p0/1996	91.80623283	2.353566188	5.2857	0.00022673	0.0023009
i2_LQ_YHS_c51226/f1p1/2651	142.0864612	12.00712956	3.5648	0.00022673	0.0023009
i2_LQ_YHS_c61754/f1p20/2869	6409.458441	44.0435053	7.1851	0.00022738	0.002307
i2_HQ_YHS_c24573/f4p1/2382	855.0984246	249.5478972	1.7768	0.00022767	0.0023091
i3_HQ_YHS_c21864/f3p0/3430	285.9130044	81.69089199	1.8073	0.00022769	0.0023091
i4_LQ_YHS_c6457/f1p0/5019	20.96026442	105.6736361	-2.3339	0.00022802	0.0023119
i3_LQ_YHS_c19331/f1p0/3475	138.7433775	31.08672807	2.158	0.00022855	0.0023164
i4_HQ_YHS_c14470/f4p0/4356	79.8926725	286.855946	-1.8442	0.00022854	0.0023164
i3_LQ_YHS_c4517/f1p3/3231	110.4453157	373.2585114	-1.7568	0.00022886	0.0023191
i1_LQ_YHS_c34605/f1p0/1663	54.27540874	205.8516276	-1.9232	0.00022975	0.0023276
i2_LQ_YHS_c8603/f1p12/2838	189.8063992	43.31135423	2.1317	0.00023019	0.0023315
i2_LQ_YHS_c21086/f1p0/2688	19.14577232	117.4210099	-2.6166	0.00023051	0.0023339
i2_LQ_YHS_c3178/f1p23/2209	157.5667462	516.6127078	-1.7131	0.00023052	0.0023339
i1_LQ_YHS_c24356/f1p11/1932	7.847980147	59.0798784	-2.9123	0.00023064	0.0023346
i2_LQ_YHS_c39263/f1p0/2141	126.2895876	26.30506944	2.2633	0.00023082	0.0023359
i1_LQ_YHS_c7249/f1p7/1564	110.1675555	376.3180844	-1.7723	0.00023177	0.0023451
i3_LQ_YHS_c7276/f1p0/3955	0	15.90312147	-Inf	0.00023203	0.0023472
i1_LQ_YHS_c20407/f1p0/1532	0.991590972	24.63416812	-4.6348	0.00023251	0.0023516
i2_LQ_YHS_c53859/f1p13/2805	309.8102137	87.69941269	1.8207	0.00023326	0.0023586
i2_LQ_YHS_c52524/f1p0/2387	119.9040992	25.30182568	2.2446	0.00023414	0.0023671
i2_LQ_YHS_c9402/f1p0/2333	0	33.24882328	-Inf	0.00023428	0.002368
i1_HQ_YHS_c6618/f4p3/1882	134.8266331	457.1762735	-1.7616	0.0002351	0.0023758
i1_LQ_YHS_c24437/f1p0/1902	68.59695787	249.16251	-1.8609	0.00023553	0.0023797
i2_LQ_YHS_c14860/f1p1/2050	4.413272263	482.6361762	-6.7729	0.00023572	0.0023805
i3_LQ_YHS_c8533/f1p0/3893	47.00980751	4.314871345	3.4456	0.00023569	0.0023805
i2_LQ_YHS_c25551/f1p49/2597	353.192648	3.184850097	6.7931	0.0002361	0.0023839
i2_LQ_YHS_c35465/f1p0/2639	54.66157862	0	Inf	0.00023631	0.0023845
i2_LQ_YHS_c39976/f1p1/2326	89.59840474	310.0175108	-1.7908	0.00023631	0.0023845
i4_LQ_YHS_c13483/f1p1/4185	508.1872081	153.2313769	1.7296	0.00023622	0.0023845
i2_LQ_YHS_c23314/f1p0/2255	479.4614193	140.4255107	1.7716	0.00023694	0.0023904
i1_LQ_YHS_c23585/f1p1/1941	67.1293369	0.359993938	7.5428	0.00023743	0.0023948
i2_LQ_YHS_c11618/f1p1/2440	30.04151019	130.422179	-2.1182	0.00023774	0.0023975
i1_HQ_YHS_c22136/f2p2/1918	0.272341299	33.13156486	-6.9266	0.00023805	0.0024
i1_LQ_YHS_c43893/f1p0/1042	2303.281833	214.5503292	3.4243	0.00023821	0.0024007
i2_LQ_YHS_c4754/f1p2/2817	23.46713921	115.1424836	-2.2947	0.00023821	0.0024007
i3_LQ_YHS_c16869/f1p0/3511	32.46283195	142.2479889	-2.1315	0.00023926	0.0024108
i3_LQ_YHS_c11649/f1p0/3477	97.7649972	6.232258775	3.9715	0.0002397	0.0024147
i3_HQ_YHS_c21638/f27p2/3806	1015.847995	10860.01124	-3.4183	0.00024087	0.002426
i1_LQ_YHS_c33770/f1p2/1845	22.54312255	110.7316768	-2.2963	0.00024136	0.0024299
i5_LQ_YHS_c3110/f1p3/5741	156.73601	37.57771637	2.0604	0.00024134	0.0024299
i2_LQ_YHS_c64775/f1p2/2009	357.6714445	89.19243243	2.0036	0.00024264	0.0024423
i2_LQ_YHS_c11908/f1p5/2715	65.23074102	243.9589278	-1.903	0.00024305	0.0024458
i2_LQ_YHS_c25617/f1p0/2810	0	16.15900687	-Inf	0.00024379	0.0024524
i3_LQ_YHS_c12565/f1p0/3274	17.74877735	206.8748902	-3.543	0.0002438	0.0024524

i2_HQ_YHS_c3679/f2p3/2743	232.5619678	63.65734963	1.8692	0.00024401	0.0024535
i2_LQ_YHS_c5237/f1p0/2775	845.1727806	145.1174754	2.542	0.00024399	0.0024535
i2_LQ_YHS_c21647/f1p0/2598	10.5252753	102.2996861	-3.2809	0.00024412	0.0024538
i2_LQ_YHS_c38809/f1p14/2108	3.601320186	183.6250097	-5.6721	0.00024414	0.0024538
i3_LQ_YHS_c12236/f1p2/3815	0	16.14842202	-Inf	0.00024422	0.0024541
i1_LQ_YHS_c19412/f1p0/2001	2.451071687	35.93801635	-3.874	0.00024522	0.0024637
i2_LQ_YHS_c8361/f1p1/2163	83.15964912	291.7387492	-1.8107	0.00024534	0.0024643
i2_LQ_YHS_c8201/f1p2/2412	224.881726	1371.54917	-2.6086	0.00024554	0.0024659
i3_LQ_YHS_c3936/f1p0/3890	1.933256058	32.47220126	-4.0701	0.000246	0.0024699
i3_LQ_YHS_c14409/f1p0/3075	34.19465396	146.4050013	-2.0981	0.00024626	0.002472
i2_LQ_YHS_c53439/f1p1/2311	129.6172944	427.3930642	-1.7213	0.00024645	0.0024734
i2_LQ_YHS_c10088/f1p0/2615	98.51628861	13.21957709	2.8977	0.00024671	0.0024755
i1_HQ_YHS_c17095/f2p0/1812	201.1597587	51.74765367	1.9588	0.000247	0.0024774
i3_HQ_YHS_c21484/f4p0/3287	330.7563554	1193.822161	-1.8517	0.00024699	0.0024774
i0_LQ_YHS_c2032/f1p0/888	852.7858512	225.0998293	1.9216	0.00024713	0.0024782
i4_LQ_YHS_c8868/f1p0/4396	115.0130736	399.9271266	-1.7979	0.00024815	0.0024878
i2_HQ_YHS_c1489/f5p0/2382	487.5622015	45.32226535	3.4273	0.0002482	0.0024879
i0_HQ_YHS_c3683/f4p0/760	17926.71795	606.5620089	4.8853	0.00024859	0.0024913
i2_LQ_YHS_c33007/f1p6/2484	2385.671274	462.7465223	2.3661	0.0002493	0.0024974
i2_LQ_YHS_c9439/f1p1/3031	123.1963643	411.5498605	-1.7401	0.00024928	0.0024974
i3_LQ_YHS_c18384/f1p0/3207	92.34038287	318.5442959	-1.7865	0.00024958	0.0024996
i1_HQ_YHS_c17938/f3p4/1928	447.0877007	131.5373116	1.7651	0.00024984	0.0025017
i2_LQ_YHS_c5712/f1p0/2986	151.977639	498.5029757	-1.7137	0.00025037	0.0025065
i4_LQ_YHS_c15777/f1p0/4014	0	15.99320827	-Inf	0.00025061	0.0025084
i2_LQ_YHS_c22431/f1p0/2327	15.219302	89.22525163	-2.5515	0.00025074	0.0025092
i1_LQ_YHS_c24930/f1p0/1746	627.1444638	2791.90286	-2.1544	0.00025081	0.0025094
i2_LQ_YHS_c21831/f1p0/2824	186.120968	2.256764909	6.3658	0.00025098	0.0025105
i2_HQ_YHS_c5393/f3p0/2351	40.08331649	162.6843513	-2.021	0.00025137	0.0025139
i0_HQ_YHS_c219/f4p0/914	209.3335484	35158.48849	-7.3919	0.0002522	0.0025217
i2_LQ_YHS_c50840/f1p5/2758	14.70643012	88.45340644	-2.5885	0.00025253	0.0025245
i2_LQ_YHS_c20473/f1p0/2614	93.14279448	13.13833636	2.8257	0.00025355	0.0025341
i1_HQ_YHS_c29394/f5p0/1978	61930.67379	1.799969692	15.07	0.00025372	0.0025353
i3_LQ_YHS_c19120/f1p3/3148	173.2192275	582.349767	-1.7493	0.00025433	0.0025409
i3_LQ_YHS_c7848/f1p2/3921	136.8441377	451.3307803	-1.7217	0.00025452	0.0025422
i1_LQ_YHS_c13814/f1p0/1186	181.0999937	587.0573389	-1.6967	0.00025464	0.002543
i2_LQ_YHS_c19818/f1p1/2948	36.79262873	153.2102479	-2.058	0.00025534	0.0025494
i2_LQ_YHS_c24470/f1p2/2761	87.62648539	313.3105398	-1.8382	0.00025679	0.0025634
i2_LQ_YHS_c24434/f1p2/2468	195.2619111	25.83484526	2.918	0.00025715	0.0025664
i1_LQ_YHS_c14215/f1p0/1359	441.774701	132.2901604	1.7396	0.000258	0.0025741
i2_LQ_YHS_c51085/f1p2/2228	409.466588	1520.684663	-1.8929	0.00025803	0.0025741
i2_LQ_YHS_c6546/f1p1/2388	12.82611097	77.85074751	-2.6016	0.0002581	0.0025743
i2_LQ_YHS_c25624/f1p1/2935	185.9283385	612.140322	-1.7191	0.00025916	0.0025843
i2_LQ_YHS_c23884/f1p3/2707	135.8746722	30.74501903	2.1439	0.00025937	0.0025859
i5_LQ_YHS_c2896/f1p2/5933	7.495504678	57.67280206	-2.9438	0.00025947	0.0025864
i2_LQ_YHS_c6846/f1p3/2112	521.393815	101.0782332	2.3669	0.00026079	0.0025989
i2_HQ_YHS_c11930/f2p3/2239	387.290217	114.596507	1.7569	0.00026169	0.0026074
i2_LQ_YHS_c23018/f1p2/2673	4.841726805	48.59447789	-3.3272	0.00026365	0.0026264
i3_HQ_YHS_c1939/f4p0/3415	796.2631207	101.554027	2.971	0.00026392	0.0026285
i5_HQ_YHS_c725/f2p0/5291	641.4240995	2.353566188	8.0903	0.00026408	0.0026296
i2_HQ_YHS_c27321/f3p4/2261	145.4112599	487.9020605	-1.7465	0.00026419	0.0026302
i2_LQ_YHS_c10026/f1p0/2407	11.22099932	90.23102089	-3.0074	0.00026476	0.0026347
i2_LQ_YHS_c32838/f1p3/2206	0	15.66773276	-Inf	0.00026471	0.0026347
i3_LQ_YHS_c3151/f1p4/4002	12.52563056	78.74087737	-2.6522	0.00026547	0.0026413
i0_LQ_YHS_c2193/f1p2/753	747.3632189	51.98699296	3.8456	0.00026582	0.0026437
i1_LQ_YHS_c26514/f1p1/1507	199.372939	641.3293308	-1.6856	0.00026577	0.0026437
i2_LQ_YHS_c49788/f1p2/2974	16.81012328	113.8594593	-2.7599	0.00026612	0.0026461
i2_LQ_YHS_c34995/f1p0/2108	375.8880876	112.8827548	1.7355	0.00026643	0.0026486
i1_LQ_YHS_c33979/f1p1/1985	78.63809521	279.6956404	-1.8306	0.0002667	0.0026502
i4_LQ_YHS_c10443/f1p0/4557	1.634047791	57.07295628	-5.1263	0.00026669	0.0026502
i2_LQ_YHS_c55771/f1p3/2348	248.669031	69.70505189	1.8349	0.00026711	0.0026538
i3_LQ_YHS_c13605/f1p7/3453	6.347792089	54.1752731	-3.0933	0.00026841	0.0026661
i1_LQ_YHS_c28006/f1p0/1297	178.2039308	0.784522063	7.8275	0.00026871	0.0026685
i1_LQ_YHS_c14558/f1p0/1220	205.1441504	672.1095759	-1.7121	0.00026895	0.0026702
i2_LQ_YHS_c22684/f1p0/2664	23.19400088	150.5051399	-2.698	0.00026898	0.0026702
i1_LQ_YHS_c13851/f1p0/1242	123.9851582	17.64353267	2.813	0.00026968	0.0026765
i3_LQ_YHS_c18711/f1p0/3429	17.38074775	96.43658105	-2.4721	0.00026981	0.0026773
i2_LQ_YHS_c48810/f1p0/2191	177.591656	587.9683643	-1.7272	0.00027004	0.002679
i3_LQ_YHS_c4964/f1p0/3438	63.60959176	236.5119701	-1.8946	0.00027025	0.0026806
i2_LQ_YHS_c52457/f1p4/2319	5619.075405	56.07986389	6.6467	0.00027044	0.0026819
i3_HQ_YHS_c2330/f2p0/3745	51.32623065	198.347837	-1.9503	0.0002708	0.0026849
i2_LQ_YHS_c21665/f1p2/2414	4.871935089	93.93080453	-4.269	0.00027089	0.0026853
i1_HQ_YHS_c25754/f2p4/1677	931.4042121	271.1860003	1.7801	0.00027128	0.0026886
i3_LQ_YHS_c12265/f1p0/3963	237.2866683	16.91576452	3.8102	0.00027199	0.002695
i4_LQ_YHS_c12449/f1p2/4410	8.474527418	63.99676665	-2.9168	0.00027262	0.0027008
i4_LQ_YHS_c6393/f1p3/4616	76.44011395	276.9189256	-1.8571	0.00027282	0.0027021
i1_LQ_YHS_c14717/f1p1/1156	29.54640643	131.6313489	-2.1554	0.00027313	0.0027047
i3_LQ_YHS_c18960/f1p4/3346	1671.631213	352.6603812	2.2449	0.0002743	0.0027157
i3_HQ_YHS_c1460/f7p0/3194	163.173588	544.8609557	-1.7395	0.00027496	0.0027217
i1_LQ_YHS_c14498/f1p0/1287	287.4823488	80.66999565	1.8334	0.00027534	0.0027248
i2_LQ_YHS_c22054/f1p13/2653	659.0339045	3063.63759	-2.2168	0.00027548	0.0027257
i5_LQ_YHS_c983/f1p0/5564	90.22933811	318.4724556	-1.8195	0.00027564	0.0027268
i1_LQ_YHS_c39272/f1p0/1627	31.02513801	135.4092501	-2.1258	0.00027667	0.0027364
i6_LQ_YHS_c671/f1p0/6894	2.255523243	43.52561279	-4.2703	0.00027695	0.0027386
i1_HQ_YHS_c2980/f2p0/1949	166.0137197	789.6226893	-2.2499	0.00027714	0.0027399
i4_LQ_YHS_c11222/f1p0/4548	157.8259616	520.4403742	-1.7214	0.00027724	0.0027403
i2_LQ_YHS_c20460/f1p1/2175	0.544682597	21.85994417	-5.3267	0.00027739	0.0027412
i2_LQ_YHS_c34495/f1p1/2237	86.83436223	2685.719062	-4.9509	0.00027768	0.0027435
i2_LQ_YHS_c23721/f1p2/2579	6.219809582	52.83656177	-3.0866	0.00027865	0.002752
i2_LQ_YHS_c54743/f1p1/2289	0.801919753	24.57903323	-4.9378	0.00027871	0.002752

i3_LQ_YHS_c14227/f1p0/3076	115.874808	1.07386011	6.7536	0.00027869	0.002752
i1_HQ_YHS_c17118/f3p0/1991	931.6827155	230.0015114	2.0182	0.0002792	0.0027552
i1_LQ_YHS_c37832/f1p1/1233	44.96174035	0	Inf	0.00027917	0.0027552
i2_LQ_YHS_c23426/f1p12/2547	383.9465504	1379.30524	-1.845	0.00027916	0.0027552
i0_LQ_YHS_c1867/f1p1/431	7.431280057	63.98337724	-3.106	0.00027974	0.0027588
i3_LQ_YHS_c9858/f1p0/3177	10.18283215	66.73374781	-2.7123	0.00027972	0.0027588
i5_LQ_YHS_c4531/f1p1/5445	32.61860652	144.6640368	-2.1489	0.00027966	0.0027588
i1_LQ_YHS_c27491/f1p0/1150	549.556088	113.9039303	2.2704	0.00028023	0.002763
i4_LQ_YHS_c13634/f1p0/4189	120.4454405	24.859445	2.2765	0.00028028	0.002763
i3_LQ_YHS_c5063/f1p0/3359	483.1638419	127.0028131	1.9277	0.00028039	0.0027635
i3_LQ_YHS_c17672/f1p0/3453	241.5840524	67.83868946	1.8323	0.00028053	0.0027644
i1_LQ_YHS_c9708/f1p0/1742	146.8429737	30.80635585	2.253	0.00028108	0.0027692
i4_LQ_YHS_c7220/f1p3/4736	4.755715408	47.40872897	-3.3174	0.00028125	0.0027703
i3_LQ_YHS_c7093/f1p1/3366	168.0390355	41.87090546	2.0048	0.00028146	0.0027718
i2_HQ_YHS_c56115/f2p0/2566	276.9739118	78.85114716	1.8125	0.0002836	0.0027923
i5_LQ_YHS_c2845/f1p6/5125	0	29.07526372	-Inf	0.00028438	0.0027995
i1_LQ_YHS_c35723/f1p0/1689	2647.472087	629.1200565	2.0732	0.00028489	0.0028038
i3_LQ_YHS_c10507/f1p21/3447	179.1856414	2295.664015	-3.6794	0.00028542	0.0028085
i1_LQ_YHS_c18301/f1p2/1987	488.285209	133.7846376	1.8678	0.0002855	0.0028088
i1_LQ_YHS_c20680/f1p3/1724	613.8203159	2492.405946	-2.0217	0.00028591	0.0028122
i1_LQ_YHS_c22026/f1p3/1964	7.775800752	57.44689286	-2.8852	0.00028609	0.0028134
i2_HQ_YHS_c15716/f5p0/2709	373.4825845	111.5241791	1.7437	0.00028678	0.0028196
i4_LQ_YHS_c10079/f1p1/4446	7.897897659	61.56531753	-2.9626	0.00028865	0.0028374
i2_LQ_YHS_c13296/f1p1/2096	346.5462208	103.6120928	1.7419	0.00028985	0.0028487
i1_LQ_YHS_c8259/f1p0/2019	11.06821902	72.38239434	-2.7092	0.00029073	0.0028567
i1_LQ_YHS_c19883/f1p0/1406	2595.331579	382.5861285	2.7621	0.00029092	0.002858
i2_LQ_YHS_c29504/f1p0/2045	82.9738027	5.723172782	3.8578	0.00029112	0.0028592
i4_HQ_YHS_c2506/f2p3/4545	0	15.65489658	-Inf	0.00029116	0.0028592
i1_HQ_YHS_c2462/f2p1/1988	22.92372876	110.5848757	-2.2702	0.00029123	0.0028593
i1_LQ_YHS_c22329/f1p0/2007	163.9345778	536.3567604	-1.7101	0.00029192	0.0028655
i3_LQ_YHS_c19886/f1p0/3186	33.33993374	143.6881647	-2.1076	0.00029198	0.0028655
i2_LQ_YHS_c25323/f1p6/2270	284.0532657	945.2029392	-1.7345	0.00029238	0.0028689
i2_LQ_YHS_c52182/f1p0/2666	0	15.62709262	-Inf	0.00029252	0.0028697
i2_HQ_YHS_c8318/f2p1/2277	149.9467116	491.4668502	-1.7126	0.00029313	0.0028751
i2_LQ_YHS_c18719/f1p5/2500	493.5377096	22.74292608	4.4397	0.00029338	0.0028762
i2_LQ_YHS_c3327/f1p2/2880	461.6630417	126.6438037	1.8661	0.00029343	0.0028762
i2_LQ_YHS_c39234/f1p4/2667	135.1896783	451.8344571	-1.7408	0.00029332	0.0028762
i2_LQ_YHS_c21145/f1p50/2127	105.553663	354.9437261	-1.7496	0.00029356	0.0028769
i2_LQ_YHS_c23278/f1p0/2284	9.789649341	70.29577141	-2.8441	0.00029432	0.0028838
i1_LQ_YHS_c4954/f1p0/2022	113.7278716	24.09715841	2.2387	0.00029499	0.0028897
i1_LQ_YHS_c21508/f1p10/1820	1058.641755	8800.903716	-3.0554	0.00029535	0.0028926
i1_LQ_YHS_c21896/f1p15/1995	547.9896384	2095.589918	-1.9351	0.00029627	0.0029007
i3_LQ_YHS_c18346/f1p2/3555	0.272341299	18.90767821	-6.1174	0.00029629	0.0029007
i2_LQ_YHS_c25549/f1p6/2677	14.23185775	369.2047298	-4.6972	0.00029785	0.0029154
i1_LQ_YHS_c25748/f1p1/1682	140.9179863	31.70604431	2.152	0.00029832	0.0029194
i1_HQ_YHS_c29855/f3p0/1974	645.2521037	200.258038	1.688	0.0002994	0.0029294
i3_LQ_YHS_c8943/f1p0/3264	22.15916666	109.7013021	-2.3076	0.00029963	0.002931
i0_LQ_YHS_c3307/f1p0/288	262.1815947	73.5573991	1.8336	0.00030002	0.0029341
i1_LQ_YHS_c13845/f1p0/1279	101.6803067	502.1123671	-2.304	0.00030007	0.0029341
i2_LQ_YHS_c21754/f1p2/2648	1.361706493	28.21680818	-4.3731	0.00030033	0.0029361
i3_LQ_YHS_c4072/f1p12/3386	16.99470593	91.37616922	-2.4267	0.0003006	0.0029382
i2_LQ_YHS_c37433/f1p1/3072	15.4118478	87.21659185	-2.5006	0.00030114	0.0029428
i2_LQ_YHS_c22964/f1p0/2586	153.9562781	4.790624455	5.0062	0.00030155	0.0029462
i1_LQ_YHS_c23616/f1p0/1698	2287.230324	178.6279525	3.6786	0.00030232	0.0029531
i2_HQ_YHS_c45446/f5p6/2193	356.9516407	1249.583897	-1.8076	0.0003032	0.0029612
i2_LQ_YHS_c26727/f1p1/2912	1.089365194	26.5203147	-4.6055	0.0003047	0.0029752
i1_HQ_YHS_c27274/f2p3/1264	184.5423261	586.373406	-1.6679	0.00030527	0.0029795
i2_LQ_YHS_c18282/f1p1/2075	56.95892816	209.2883741	-1.8775	0.00030527	0.0029795
i3_LQ_YHS_c16542/f2p0/3454	150.6379131	0	Inf	0.00030581	0.0029843
i1_LQ_YHS_c10319/f1p0/1516	303.9502851	87.39348786	1.7982	0.0003061	0.0029865
i1_LQ_YHS_c33717/f1p0/1602	3031.308865	567.2180896	2.418	0.00030627	0.0029875
i2_LQ_YHS_c28402/f1p0/2049	119.4285599	401.3838891	-1.7488	0.00030689	0.0029929
i3_LQ_YHS_c10559/f1p39/3983	147.3318532	23.86844464	2.6259	0.00030702	0.0029931
i3_LQ_YHS_c10680/f1p0/3801	174.4373148	46.058367	1.9212	0.00030703	0.0029931
i1_HQ_YHS_c6125/f3p0/1997	403.4912535	5.233517695	6.2686	0.00030781	0.0030001
i2_LQ_YHS_c13421/f1p0/2415	0	15.3216408	-Inf	0.00030801	0.0030015
i3_LQ_YHS_c12564/f1p3/3629	223.6928657	722.3259024	-1.6911	0.00030808	0.0030015
i2_LQ_YHS_c5799/f1p2/2174	69.41269288	484.5626993	-2.8034	0.00030832	0.0030033
i3_LQ_YHS_c9070/f1p7/3310	126.8449225	0.75225497	7.3976	0.00030885	0.0030078
i3_HQ_YHS_c1694/f4p0/3503	50.51750857	186.8820605	-1.8873	0.00030905	0.0030092
i2_LQ_YHS_c51919/f1p3/2401	45.38616421	268.8441589	-2.5664	0.00030941	0.0030121
i6_LQ_YHS_c695/f1p0/6331	0	59.20227296	-Inf	0.00030951	0.0030125
i2_LQ_YHS_c58662/f1p0/2021	162.1642134	27.47122815	2.5615	0.0003107	0.0030234
i1_LQ_YHS_c28590/f1p1/1269	333.7546826	1156.986983	-1.7935	0.00031119	0.0030275
i2_LQ_YHS_c50075/f1p5/2447	32.43054612	192.9722208	-2.573	0.00031133	0.0030283
i1_LQ_YHS_c20729/f1p1/1453	317.6262408	1124.501658	-1.8239	0.00031171	0.0030314
i2_LQ_YHS_c55762/f1p13/2013	941.700777	4973.888404	-2.401	0.00031331	0.0030464
i3_LQ_YHS_c12024/f1p0/3983	132.111431	443.9889239	-1.7488	0.00031366	0.0030491
i4_LQ_YHS_c15426/f1p6/4560	14.43917739	154.2955982	-3.4176	0.00031425	0.0030543
i2_LQ_YHS_c38020/f1p0/2610	175.9159183	47.03258172	1.9032	0.00031471	0.0030581
i2_HQ_YHS_c5508/f2p0/2813	148.7247839	35.92956309	2.0494	0.00031637	0.0030736
i2_LQ_YHS_c28230/f1p18/2047	350.3891797	42.38555993	3.0473	0.00031681	0.0030773
i2_LQ_YHS_c14260/f1p1/2027	144.9640703	490.7150204	-1.7592	0.00031698	0.0030783
i3_LQ_YHS_c6489/f1p19/3250	34.58529249	142.9358295	-2.0471	0.00031722	0.0030801
i1_LQ_YHS_c7700/f1p0/1876	200.5851802	1054.861924	-2.3948	0.00031776	0.0030847
i3_LQ_YHS_c6995/f1p0/3112	201.008989	18.5782928	3.4356	0.00031963	0.0031022
i1_LQ_YHS_c21304/f1p0/1791	139.545095	449.0788378	-1.6862	0.00032228	0.0031266
i1_LQ_YHS_c4529/f2p0/1840	323.8998274	95.6760728	1.7593	0.00032227	0.0031266
i2_LQ_YHS_c36151/f1p1/2599	79.89826967	0	Inf	0.00032299	0.0031329

i1_LQ_YHS_c28946/f1p0/1310	224.9159793	710.4346902	-1.6593	0.00032353	0.0031376
i0_LQ_YHS_c1683/f1p3/721	1.233723986	38.41168509	-4.9605	0.00032451	0.0031463
i2_LQ_YHS_c14785/f1p1/2074	4635.951743	190.1123671	4.6079	0.00032463	0.0031463
i4_LQ_YHS_c13096/f1p6/4232	189.123965	618.7157879	-1.7099	0.00032459	0.0031463
i1_LQ_YHS_c28700/f1p0/1343	349.6648223	1236.334893	-1.822	0.00032566	0.003155
i3_LQ_YHS_c6780/f1p3/3443	48.1713521	182.680224	-1.9231	0.00032563	0.003155
i1_HQ_YHS_c6043/f2p0/1513	156.5175055	39.00331713	2.0047	0.00032577	0.0031555
i2_HQ_YHS_c17511/f2p3/2773	90.33035071	300.9690876	-1.7363	0.00032673	0.0031641
i2_LQ_YHS_c38547/f1p3/2913	20.76932105	104.690889	-2.3336	0.00032712	0.0031666
i3_LQ_YHS_c4281/f1p2/3507	0	20.40488089	-Inf	0.00032707	0.0031666
i5_LQ_YHS_c998/f1p0/5276	316.5181872	96.02224498	1.7208	0.0003275	0.0031696
i1_LQ_YHS_c26425/f1p0/1412	59.55435027	216.7438246	-1.8637	0.00032781	0.003172
i2_LQ_YHS_c29056/f1p0/2057	94.04308326	0	Inf	0.00032847	0.0031778
i3_LQ_YHS_c3070/f1p1/3673	189.959377	617.372853	-1.7005	0.00032905	0.0031828
i2_LQ_YHS_c41106/f1p5/2632	93.87570622	325.6363908	-1.7944	0.00033041	0.0031953
i2_LQ_YHS_c50686/f1p10/2636	736.1075857	20895.60845	-4.8271	0.00033084	0.0031987
i2_HQ_YHS_c21594/f2p3/2523	158.0815101	28.98130656	2.4475	0.00033098	0.0031995
i2_LQ_YHS_c13281/f1p0/2631	0	26.4446029	-Inf	0.00033193	0.0032074
i4_LQ_YHS_c10164/f1p1/5067	77.32459247	276.492618	-1.8382	0.00033193	0.0032074
i2_LQ_YHS_c22424/f1p2/2783	4.902143374	71.97046288	-3.8759	0.0003323	0.0032102
i3_LQ_YHS_c12608/f1p4/3665	107.8265127	22.35121828	2.2703	0.00033235	0.0032102
i1_HQ_YHS_c1620/f8p0/1890	499.4601479	155.869267	1.68	0.00033277	0.0032136
i1_HQ_YHS_c2683/f5p0/1446	688.009666	3213.061903	-2.2234	0.00033338	0.0032188
i0_LQ_YHS_c1078/f1p0/639	0	15.40841047	-Inf	0.00033346	0.0032189
i1_LQ_YHS_c14093/f1p1/1102	1549.168466	15.37673615	6.6546	0.00033425	0.003226
i1_LQ_YHS_c25529/f1p0/1690	15.53903327	85.37937937	-2.458	0.00033438	0.0032265
i4_LQ_YHS_c5030/f1p0/4466	69.35037707	8.026106761	3.1111	0.00033628	0.0032442
i2_LQ_YHS_c60850/f4p1/2394	128.573597	403.5908134	-1.6503	0.0003369	0.0032495
i5_LQ_YHS_c2325/f1p4/5298	308.7710843	6969.479051	-4.4964	0.00033754	0.0032551
i2_LQ_YHS_c14309/f1p1/2109	287.4276551	966.4750365	-1.7495	0.00033764	0.0032554
i3_LQ_YHS_c14647/f1p3/3068	0	15.33163287	-Inf	0.00033777	0.003256
i3_LQ_YHS_c10980/f1p0/3200	5.092281328	147.4968276	-4.8562	0.00033812	0.0032587
i2_LQ_YHS_c20224/f1p6/2378	269.3655862	78.73151875	1.7746	0.00033872	0.0032639
i1_LQ_YHS_c12672/f1p0/1806	320.684847	5.480003805	5.8708	0.00034022	0.0032777
i1_LQ_YHS_c3092/f1p0/1874	13.78114132	80.04353146	-2.5381	0.00034093	0.0032838
i2_LQ_YHS_c49346/f1p3/2809	83.81305499	291.7203039	-1.7993	0.00034121	0.0032859
i1_HQ_YHS_c11802/f2p5/1947	187.8485679	599.4082277	-1.674	0.00034153	0.003287
i1_LQ_YHS_c43745/f1p0/1049	4598.254641	865.497832	2.4095	0.00034152	0.003287
i3_LQ_YHS_c22589/f1p0/3015	424.2702347	19.95420738	4.4102	0.00034145	0.003287
i5_LQ_YHS_c1740/f1p0/5220	0	15.26097698	-Inf	0.00034181	0.003289
i2_LQ_YHS_c59978/f1p4/2047	186.2968599	49.60040647	1.9092	0.00034229	0.003293
i5_LQ_YHS_c3866/f1p0/5760	4.024702909	43.04606954	-3.4189	0.00034235	0.003293
i0_LQ_YHS_c1036/f1p4/778	530.0102024	125.9732636	2.0729	0.00034266	0.0032952
i2_HQ_YHS_c10968/f2p0/2618	0	18.10972718	-Inf	0.00034291	0.003297
i5_LQ_YHS_c3969/f1p0/5103	8.998228646	89.72884824	-3.3179	0.00034326	0.0032997
i4_LQ_YHS_c13889/f1p0/4199	193.4118912	1210.674308	-2.6461	0.00034503	0.0033161
i1_LQ_YHS_c5549/f1p3/1651	1415.704999	403.8798795	1.8095	0.00034573	0.0033221
i1_LQ_YHS_c14701/f1p0/1376	152.4254892	36.80990086	2.0499	0.00034613	0.0033254
i1_LQ_YHS_c26609/f1p0/1990	267.7042298	871.9872035	-1.7037	0.00034651	0.0033282
i2_LQ_YHS_c28629/f1p7/2101	484.9662923	2042.515059	-2.0744	0.00034656	0.0033282
i0_LQ_YHS_c2113/f1p0/924	196.7474606	642.9889309	-1.7084	0.00034682	0.0033286
i1_LQ_YHS_c19848/f1p0/1848	7.670071755	56.44866434	-2.8796	0.00034671	0.0033286
i2_LQ_YHS_c14557/f1p0/2082	73.64201078	358.7647907	-2.2844	0.0003468	0.0033286
i2_LQ_YHS_c28477/f1p4/2018	75.05050408	499.9164874	-2.7358	0.00034748	0.0033343
i1_LQ_YHS_c5476/f1p0/1816	302.858962	88.32777497	1.7777	0.00034782	0.0033363
i2_LQ_YHS_c21988/f1p0/2439	0	16.56682844	-Inf	0.00034781	0.0033363
i2_LQ_YHS_c41506/f1p1/2773	204.7990565	26.26936545	2.9628	0.00034802	0.0033376
i1_LQ_YHS_c27979/f1p0/1226	125.2412182	416.2989741	-1.7329	0.00034901	0.0033464
i4_HQ_YHS_c14633/f3p13/4069	12.1273843	157.5210549	-3.6992	0.00034928	0.0033483
i1_LQ_YHS_c7114/f1p0/1784	165.4252002	531.686678	-1.6844	0.00035015	0.0033559
i2_HQ_YHS_c32521/f2p0/2435	0	177.8889216	-Inf	0.00035152	0.0033684
i2_LQ_YHS_c43655/f1p0/2058	467.6693551	2.843221275	7.3618	0.00035215	0.0033737
i2_LQ_YHS_c24505/f1p5/3168	101.0050566	334.6197711	-1.7281	0.00035278	0.0033791
i2_LQ_YHS_c26240/f1p0/2688	141.8309211	26.9983587	2.3932	0.00035323	0.0033828
i3_LQ_YHS_c17838/f1p0/3695	0	39.82492268	-Inf	0.00035332	0.0033829
i5_LQ_YHS_c3790/f1p0/5453	84.04643619	15.47800057	2.441	0.00035418	0.0033906
i0_LQ_YHS_c2378/f1p0/874	0	15.04735074	-Inf	0.0003544	0.003392
i1_LQ_YHS_c13559/f1p0/1242	82.85997412	14.54604502	2.51	0.00035528	0.0033994
i3_LQ_YHS_c5408/f1p0/3545	43.81863285	4.301562145	3.3486	0.00035532	0.0033994
i2_LQ_YHS_c11493/f1p0/2239	1.634047791	29.80362465	-4.189	0.00035547	0.0034002
i2_LQ_YHS_c62796/f1p3/2782	156.5674565	502.0821826	-1.6811	0.00035613	0.0034052
i3_LQ_YHS_c8556/f1p0/3515	31.4141741	129.8754563	-2.0476	0.00035609	0.0034052
i2_LQ_YHS_c36977/f1p5/2883	186.6075414	853.4341409	-2.1933	0.00035675	0.0034104
i1_LQ_YHS_c21688/f1p11/1551	7.101058099	189.415998	-4.7374	0.00035984	0.0034387
i3_LQ_YHS_c22497/f1p3/3044	49.1656238	0.75225497	6.0303	0.00035985	0.0034387
i6_LQ_YHS_c939/f1p3/6477	18.83653173	97.58958986	-2.3732	0.00036005	0.0034399
i2_HQ_YHS_c28025/f2p0/2024	85.31247114	0.353872233	7.9134	0.00036015	0.0034401
i0_LQ_YHS_c1161/f1p0/789	4297.602695	52.11436209	6.3657	0.00036088	0.0034465
i1_HQ_YHS_c40418/f88p0/1724	1077.316306	12529.31741	-3.5398	0.00036214	0.0034571
i1_LQ_YHS_c32125/f1p0/1898	102.8059189	488.640291	-2.2488	0.00036208	0.0034571
i2_LQ_YHS_c21944/f1p3/2670	28.70078506	134.2624025	-2.2259	0.00036227	0.0034577
i2_LQ_YHS_c17844/f1p0/2911	159.8369152	498.0878913	-1.6398	0.00036256	0.0034598
i1_LQ_YHS_c22733/f1p0/1928	687.0519225	114.914995	2.5799	0.0003627	0.00346
i3_LQ_YHS_c14999/f1p2/3041	52.31147766	193.9154677	-1.8902	0.00036273	0.00346
i2_LQ_YHS_c38247/f1p1/2113	372.7865972	17.00253419	4.4545	0.0003631	0.0034629
i1_HQ_YHS_c27083/f3p0/1341	12115.80019	772.6967647	3.9708	0.00036376	0.0034685
i1_LQ_YHS_c21301/f1p1/1758	127.6383178	0	Inf	0.00036394	0.0034689
i2_LQ_YHS_c5017/f1p6/2622	4.115794499	67.61163426	-4.038	0.00036391	0.0034689
i2_LQ_YHS_c49056/f1p1/2184	571.7367399	68.21377093	3.0672	0.00036548	0.0034829

i2_LQ_YHS_c43599/f1p0/2092	60.42832973	385.3262976	-2.6728	0.00036562	0.0034835
i3_LQ_YHS_c9005/f1p3/3632	71.22481899	239.9207572	-1.7521	0.00036612	0.0034876
i3_LQ_YHS_c4324/f1p0/3449	6.836671575	58.61147209	-3.0998	0.00036672	0.0034926
i2_LQ_YHS_c23436/f1p0/2358	24.69832729	0.392261031	5.9765	0.00036721	0.0034959
i2_LQ_YHS_c8103/f1p6/2911	8.659118443	62.02270553	-2.8405	0.00036718	0.0034959
i1_LQ_YHS_c20371/f1p0/1880	198.4397457	8.689340724	4.5133	0.00036766	0.0034995
i2_LQ_YHS_c33640/f1p3/2816	252.8230639	63.83104827	1.9858	0.00036882	0.0035098
i2_LQ_YHS_c34384/f1p7/2334	0	14.81255481	-Inf	0.00036894	0.0035103
i2_LQ_YHS_c40814/f1p0/2162	74.049831	3.678375547	4.3314	0.00037021	0.0035217
i3_LQ_YHS_c14906/f1p0/3034	241.1333443	69.35263824	1.7978	0.00037149	0.0035331
i2_LQ_YHS_c22993/f1p76/2109	70632.40968	0	Inf	0.00037176	0.003535
i2_HQ_YHS_c37683/f2p3/2838	41.78250563	185.2410958	-2.1484	0.00037227	0.0035392
i1_HQ_YHS_c681/f26p0/1896	1066.338926	8822.011154	-3.0484	0.00037322	0.0035475
i3_HQ_YHS_c5293/f2p1/3144	834.6113385	258.9868872	1.6882	0.00037392	0.0035534
i2_LQ_YHS_c64827/f1p0/2002	112.8693349	24.39933265	2.2097	0.00037478	0.0035609
i2_LQ_YHS_c36873/f1p0/2388	243.0726726	1.781604576	7.0921	0.000376	0.0035719
i2_LQ_YHS_c14762/f1p3/2035	165.4291447	526.467181	-1.6701	0.00037618	0.0035728
i2_LQ_YHS_c2910/f1p0/2804	79.10700096	266.8656407	-1.7542	0.00037654	0.0035755
i1_LQ_YHS_c42404/f1p0/1889	460.4524506	0	Inf	0.00037724	0.0035814
i2_LQ_YHS_c53950/f1p3/2718	85.93663449	297.7501932	-1.7928	0.00037815	0.0035894
i1_LQ_YHS_c22471/f1p0/1764	566.3585042	84.47025207	2.7452	0.00037843	0.0035913
i1_LQ_YHS_c32815/f1p1/1886	69.87137245	248.4884912	-1.8304	0.00037874	0.0035936
i2_LQ_YHS_c26684/f1p33/2500	292.2962082	35.34934819	3.0477	0.00037943	0.0035994
i2_LQ_YHS_c20312/f1p4/2781	233.7578422	67.28288117	1.7967	0.00038091	0.0036121
i3_LQ_YHS_c3001/f1p3/3430	91.73308647	306.508924	-1.7404	0.00038087	0.0036121
i2_LQ_YHS_c19212/f1p0/2215	402.632876	1350.761173	-1.7462	0.00038106	0.0036128
i2_LQ_YHS_c20725/f1p0/2067	27.98406292	125.0908338	-2.1603	0.00038125	0.0036139
i1_LQ_YHS_c1135/f11p0/1866	5942.843782	1009.795396	2.5571	0.00038168	0.0036172
i2_LQ_YHS_c5743/f1p3/2622	12.33342343	77.96370211	-2.6602	0.00038303	0.0036294
i4_LQ_YHS_c7839/f1p3/4774	0	28.38588437	-Inf	0.00038382	0.0036361
i0_HQ_YHS_c3654/f10p0/951	473.9598936	28366.82413	-5.9033	0.00038425	0.0036388
i1_HQ_YHS_c40172/f18p1/1920	4509.179962	223.2144288	4.3364	0.00038439	0.0036388
i1_LQ_YHS_c20012/f1p2/1441	176.5321161	29.93723637	2.5599	0.00038421	0.0036388
i1_LQ_YHS_c7254/f1p5/1932	32.32100907	161.6155471	-2.322	0.00038441	0.0036388
i1_LQ_YHS_c43830/f1p0/1026	296.02747	82.11897677	1.8499	0.00038494	0.0036431
i3_LQ_YHS_c3781/f1p0/3857	474.8600652	150.3888693	1.6588	0.00038583	0.0036509
i5_LQ_YHS_c3804/f1p0/5923	143.2159222	33.86047901	2.0805	0.00038695	0.0036607
i2_LQ_YHS_c32792/f1p0/2439	0	15.08068362	-Inf	0.00038751	0.0036653
i2_HQ_YHS_c61573/f19p48/2015	299.5944327	0	Inf	0.00038825	0.0036716
i4_LQ_YHS_c12411/f1p0/4436	0	15.06678164	-Inf	0.00038841	0.0036724
i2_LQ_YHS_c40998/f1p7/1937	2052.799419	534.0683596	1.9425	0.00038875	0.0036748
i3_HQ_YHS_c2146/f3p1/3270	286.2997894	916.75564	-1.679	0.0003895	0.0036813
i2_HQ_YHS_c61093/f15p2/2094	1825.383844	462.3457718	1.9812	0.00038997	0.0036849
i3_LQ_YHS_c10054/f1p9/3623	0	15.03451455	-Inf	0.00039052	0.0036894
i2_LQ_YHS_c58658/f1p0/2046	0	15.85742541	-Inf	0.0003923	0.0037055
i4_LQ_YHS_c3491/f1p0/4499	74.51242157	0.784522063	6.5695	0.0003925	0.0037067
i1_LQ_YHS_c42152/f1p9/1809	126.4902079	402.1960607	-1.6689	0.00039277	0.0037085
i5_LQ_YHS_c3283/f1p5/5154	126.356323	29.97613773	2.0756	0.00039301	0.00371
i1_LQ_YHS_c28231/f2p0/1179	61.44422664	2444.896111	-5.3144	0.0003933	0.0037121
i2_LQ_YHS_c27085/f1p1/2201	54.94185795	271.027582	-2.3025	0.00039375	0.0037156
i2_HQ_YHS_c32447/f2p0/2874	300.4406429	84.63656324	1.8277	0.00039432	0.0037203
i2_HQ_YHS_c27377/f5p7/2155	3865.586673	670.5797567	2.5272	0.00039599	0.0037352
i2_LQ_YHS_c42161/f1p1/2411	32.57074981	156.8499209	-2.2677	0.00039685	0.0037427
i3_LQ_YHS_c8911/f1p0/3361	8.064976695	928.7441511	-6.8475	0.00039703	0.0037436
i3_LQ_YHS_c11007/f3p1/3609	0	14.929933	-Inf	0.00039746	0.0037469
i2_LQ_YHS_c40329/f1p1/2381	72.77919098	12.16574066	2.5807	0.0003976	0.0037475
i3_LQ_YHS_c13621/f1p0/3320	117.5315258	26.84863432	2.1301	0.00039772	0.0037479
i2_LQ_YHS_c53387/f1p2/2203	5.153503305	45.68221975	-3.148	0.00039781	0.0037481
i1_HQ_YHS_c40427/f116p0/1650	1035.435626	5889.932867	-2.508	0.00039815	0.0037505
i3_LQ_YHS_c18380/f1p0/3706	19.43527856	99.3556339	-2.3539	0.00039844	0.0037525
i2_LQ_YHS_c9272/f1p0/2510	118.4592308	389.9785776	-1.719	0.00039877	0.0037549
i5_LQ_YHS_c4866/f1p0/5049	7.772459435	1393.975531	-7.4866	0.0003991	0.0037573
i2_LQ_YHS_c5353/f1p1/2600	179.3113274	576.3841773	-1.6846	0.00039923	0.0037578
i1_LQ_YHS_c3716/f1p0/1801	360.9508876	51.56337019	2.8074	0.00039944	0.003759
i1_HQ_YHS_c29311/f3p0/1738	669.0792817	5.722580006	6.8694	0.00039959	0.0037597
i2_LQ_YHS_c6813/f1p4/2511	35.89086558	143.0692808	-1.995	0.00039994	0.0037623
i3_LQ_YHS_c14500/f1p0/3070	376.0152277	18.68121578	4.3311	0.00040026	0.0037645
i1_LQ_YHS_c38414/f1p1/1333	367.7647635	1224.432576	-1.7353	0.00040072	0.0037681
i2_LQ_YHS_c21652/f1p2/2106	23.18858202	118.9500066	-2.3589	0.00040093	0.0037684
i2_LQ_YHS_c8838/f1p0/2238	32.89462781	140.0808378	-2.0903	0.00040093	0.0037684
i3_LQ_YHS_c6035/f1p1/3894	17.15589937	141.2268531	-3.0412	0.00040098	0.0037684
i0_LQ_YHS_c2863/f1p0/1010	10774.91751	0	Inf	0.00040215	0.0037781
i2_LQ_YHS_c20903/f1p2/2729	24.87418326	156.6256319	-2.6546	0.00040217	0.0037781
i1_LQ_YHS_c36117/f1p1/1528	32.64720399	377.606646	-3.5319	0.00040283	0.0037828
i4_LQ_YHS_c4780/f1p0/4334	748.9901797	3324.151059	-2.15	0.00040281	0.0037828
i2_LQ_YHS_c10420/f1p2/2283	302.3634478	1014.196526	-1.746	0.00040557	0.0038078
i2_LQ_YHS_c53024/f1p0/2774	44.41818509	169.1712682	-1.9293	0.00040603	0.0038114
i2_LQ_YHS_c22824/f1p5/2966	9.350245754	80.27730115	-3.1019	0.00040632	0.0038134
i6_LQ_YHS_c378/f1p0/6123	232.0080248	748.9115429	-1.6906	0.00040672	0.0038164
i2_LQ_YHS_c44603/f1p3/2053	380.3066628	0	Inf	0.00040691	0.0038174
i2_LQ_YHS_c58120/f1p6/2160	256.034551	61.1228973	2.0666	0.00040767	0.0038238
i2_LQ_YHS_c12047/f1p0/2734	7.081340498	56.76861092	-3.003	0.00040815	0.0038276
i1_LQ_YHS_c6364/f1p0/1451	1.089365194	25.11659617	-4.5271	0.00040851	0.0038302
i3_LQ_YHS_c8327/f1p0/3582	118.1133805	0	Inf	0.00041007	0.0038441
i6_LQ_YHS_c317/f1p0/6107	151.9259742	476.3950055	-1.6488	0.00041022	0.0038448
i2_LQ_YHS_c8837/f1p2/2542	0	17.38195903	-Inf	0.00041166	0.0038568
i3_HQ_YHS_c13856/f2p0/3066	134.2828349	468.842524	-1.8038	0.00041173	0.0038568
i4_LQ_YHS_c5100/f1p3/4490	971.8858804	262.8896282	1.8863	0.00041175	0.0038568
i3_LQ_YHS_c3476/f1p0/3384	169.0148199	43.64413699	1.9533	0.00041194	0.0038578

i3_LQ_YHS_c5042/f1p4/3193	242.8041215	71.30395133	1.7677	0.00041249	0.0038622
i2_LQ_YHS_c14876/f1p1/2076	37.63571418	213.405066	-2.5034	0.00041372	0.003873
i2_HQ_YHS_c17729/f3p5/2954	11.92007303	68.18647954	-2.5161	0.00041432	0.0038779
i4_LQ_YHS_c13099/f1p3/4154	11.23149	71.16777568	-2.6637	0.0004149	0.0038818
i5_LQ_YHS_c2663/f1p1/5172	15.06986301	85.31760908	-2.5012	0.00041489	0.0038818
i1_LQ_YHS_c25806/f1p0/1914	1942.971504	418.3714148	2.2154	0.00041507	0.0038827
i2_HQ_YHS_c8152/f3p3/2236	44.91007559	168.7851288	-1.9101	0.00041616	0.0038921
i1_HQ_YHS_c27036/f4p6/1229	7447.019804	40.31801466	7.5291	0.00041667	0.0038946
i2_LQ_YHS_c28396/f1p4/2032	129.0912343	416.5916699	-1.6902	0.0004166	0.0038946
i4_LQ_YHS_c2699/f1p4/4683	24.40165493	111.8630836	-2.1967	0.00041656	0.0038946
i2_LQ_YHS_c19820/f1p0/2149	34.60709602	134.1929729	-1.9552	0.00041687	0.0038957
i3_LQ_YHS_c17743/f1p0/3238	569.063607	177.4923617	1.6808	0.00041833	0.0039086
i1_LQ_YHS_c33842/f1p0/1707	87.12889597	16.43163837	2.4067	0.00041862	0.0039105
i2_HQ_YHS_c46006/f26p8/2666	560.0170705	2120.145646	-1.9206	0.00041884	0.0039118
i2_HQ_YHS_c40078/f3p13/3004	420.5759321	1491.873617	-1.8267	0.00042005	0.0039224
i2_HQ_YHS_c2201/f6p6/2761	2.070465482	125.2431292	-5.9186	0.00042066	0.0039273
i4_LQ_YHS_c7046/f1p16/4538	248.7023579	783.6733294	-1.6558	0.0004226	0.0039447
i3_LQ_YHS_c14076/f1p0/3015	0.359624837	18.13034364	-5.6558	0.0004239	0.003956
i1_LQ_YHS_c22033/f1p1/1922	0	51.66400228	-Inf	0.00042399	0.0039562
i2_HQ_YHS_c57905/f2p2/2042	71.61591588	242.7776816	-1.7613	0.00042446	0.0039597
i3_LQ_YHS_c9221/f1p1/3625	113.5149966	25.5738644	2.1501	0.00042503	0.0039636
i4_HQ_YHS_c2308/f2p2/4966	148.1744719	36.18446291	2.0339	0.00042501	0.0039636
i1_LQ_YHS_c43640/f1p1/1029	702.6262994	23065.51665	-5.0368	0.00042614	0.0039731
i4_LQ_YHS_c11578/f1p1/4922	0	14.51543649	-Inf	0.0004266	0.0039767
i4_LQ_YHS_c12102/f1p5/4564	0	27.23524666	-Inf	0.00042681	0.0039779
i3_LQ_YHS_c9650/f1p3/3640	0.272341299	18.13812391	-6.0575	0.00042875	0.0039951
i2_LQ_YHS_c49924/f1p1/2467	62.26728932	213.4609877	-1.7774	0.00042985	0.0040042
i2_LQ_YHS_c9734/f1p2/2524	32.03310527	130.8145993	-2.0299	0.00042989	0.0040042
i2_HQ_YHS_c25924/f2p1/2198	40.87522067	153.7154635	-1.911	0.00043056	0.0040097
i2_LQ_YHS_c39048/f1p2/2647	378.7352335	118.365483	1.6779	0.000431	0.004013
i2_HQ_YHS_c43151/f2p1/2046	80.43749991	268.5042749	-1.739	0.00043111	0.0040132
i4_LQ_YHS_c5764/f1p0/4674	176.0635777	1.769361165	6.6367	0.00043279	0.0040281
i2_HQ_YHS_c17583/f7p5/2646	139.4617882	454.0537397	-1.703	0.00043348	0.0040338
i2_LQ_YHS_c25802/f1p7/2891	38.15985703	437.5018087	-3.5192	0.00043383	0.0040363
i9_LQ_YHS_c37/f1p0/9107	280.9881157	83.66519264	1.7478	0.00043414	0.0040384
i3_LQ_YHS_c13200/f1p7/3164	97.43776952	10.54938146	3.2073	0.00043547	0.00405
i2_LQ_YHS_c24128/f1p0/2217	42.53579676	2.938363988	3.8556	0.00043592	0.0040534
i1_HQ_YHS_c39969/f2p0/1962	109.7152534	347.5070385	-1.6633	0.00043844	0.0040752
i3_LQ_YHS_c19163/f1p1/3491	180.4256101	18.75522835	3.266	0.00043839	0.0040752
i2_LQ_YHS_c33183/f1p3/2748	149.4430344	11.07683257	3.754	0.00043921	0.0040816
i1_LQ_YHS_c20489/f1p0/1783	115.0544751	4.770600773	4.592	0.00043967	0.0040851
i2_LQ_YHS_c23276/f1p2/2825	241.1657905	776.0617142	-1.6861	0.00044059	0.0040929
i2_LQ_YHS_c59755/f1p2/2063	116.8645069	24.91185554	2.2299	0.00044118	0.0040975
i3_LQ_YHS_c19648/f1p0/3205	27.55734726	118.0068339	-2.0984	0.00044176	0.0041021
i3_LQ_YHS_c11219/f1p0/3194	58.21410863	204.3030406	-1.8113	0.00044323	0.0041151
i1_LQ_YHS_c19398/f1p0/1698	102.1022234	334.8392461	-1.7135	0.00044392	0.0041199
i1_LQ_YHS_c19622/f1p3/1938	62.3575755	215.2320074	-1.7873	0.0004439	0.0041199
i2_LQ_YHS_c35356/f1p2/2325	40.79794433	165.6535167	-2.0216	0.00044488	0.0041279
i2_LQ_YHS_c22139/f1p3/2104	408.9577827	127.424969	1.6823	0.00044565	0.0041343
i3_LQ_YHS_c16883/f1p0/3088	91.12256844	303.2523929	-1.7346	0.00044578	0.0041347
i4_LQ_YHS_c6491/f1p0/4361	15.33552168	85.47009849	-2.4785	0.00044604	0.0041363
i1_LQ_YHS_c5774/f1p0/1769	597.2248865	2298.240792	-1.9442	0.00044652	0.0041397
i2_LQ_YHS_c21614/f1p1/2506	113.6502733	25.06090804	2.1811	0.00044658	0.0041397
i2_LQ_YHS_c21114/f1p0/2614	409.7257807	129.6594589	1.6599	0.00044755	0.0041479
i1_LQ_YHS_c25256/f1p0/1653	131.5421122	419.0592949	-1.6716	0.00044766	0.0041481
i2_LQ_YHS_c38482/f1p2/2580	0	44.25358092	-Inf	0.00044809	0.0041498
i3_LQ_YHS_c11811/f1p0/3355	339.4841229	0	Inf	0.00044802	0.0041498
i4_LQ_YHS_c9190/f1p0/4336	14.82313329	86.91283814	-2.5517	0.0004481	0.0041498
i2_LQ_YHS_c60734/f9p10/2183	991.1344793	5609.900181	-2.5008	0.00044861	0.0041538
i3_LQ_YHS_c18141/f1p0/3108	6.208046756	95.0921073	-3.9371	0.00044872	0.004154
i5_LQ_YHS_c4806/f1p0/5012	8.175319151	71.88590501	-3.1364	0.00044884	0.0041543
i2_LQ_YHS_c33314/f1p1/2676	83.42083077	1.061616699	6.2961	0.00044905	0.0041555
i1_LQ_YHS_c4500/f1p1/1884	61.83755426	215.7936176	-1.8031	0.00044939	0.004157
i2_LQ_YHS_c25943/f1p1/2685	0.631966135	33.74084948	-5.7385	0.00044944	0.004157
i4_HQ_YHS_c2415/f2p2/4575	236.0625482	4.978698084	5.5673	0.00044947	0.004157
i3_LQ_YHS_c7003/f1p1/3556	209.3142748	9.626825203	4.4425	0.00044964	0.0041578
i1_LQ_YHS_c4102/f1p0/1897	13.17985021	76.61886907	-2.5394	0.00045264	0.0041847
i4_LQ_YHS_c11537/f1p4/4750	15.20119521	82.10436226	-2.4333	0.00045289	0.0041862
i1_LQ_YHS_c25857/f1p0/1490	4.543790679	43.77932707	-3.2683	0.00045392	0.0041949
i1_LQ_YHS_c18942/f1p0/1513	377.9044111	115.9168939	1.7049	0.00045578	0.0042105
i2_LQ_YHS_c37256/f1p0/2857	0	14.68842259	-Inf	0.00045571	0.0042105
i3_LQ_YHS_c9241/f1p1/3761	77.51434151	322.8922282	-2.0585	0.0004559	0.0042108
i1_LQ_YHS_c9066/f1p5/1839	0	14.68230088	-Inf	0.00045618	0.0042126
i3_LQ_YHS_c10285/f1p13/3081	307.0189333	66.0104428	2.2176	0.00045724	0.0042215
i4_HQ_YHS_c2379/f2p6/4432	532.9512344	171.1379825	1.6388	0.0004584	0.0042315
i3_LQ_YHS_c7573/f1p0/3978	223.2723612	34.82955759	2.6804	0.00045857	0.0042322
i3_LQ_YHS_c13559/f1p0/3522	76.18448761	262.0290411	-1.7822	0.00045888	0.0042343
i2_HQ_YHS_c23005/f2p13/2893	87.4397055	288.382337	-1.7216	0.00045975	0.0042415
i1_HQ_YHS_c20761/f3p0/1538	23.47763827	537.0875264	-4.5158	0.00046051	0.0042477
i1_LQ_YHS_c14013/f1p0/1234	248.6076738	18.01351868	3.7867	0.00046131	0.0042542
i0_HQ_YHS_c96/f4p0/788	32755.9882	371.5269813	6.4621	0.00046155	0.0042557
i1_LQ_YHS_c2340/f2p3/1599	3237.982913	498.854835	2.6984	0.00046194	0.0042584
i2_LQ_YHS_c55445/f1p0/2973	0	14.60552329	-Inf	0.00046214	0.0042595
i1_LQ_YHS_c24489/f1p0/1722	1369.484058	174.8260375	2.9696	0.0004627	0.0042638
i2_LQ_YHS_c55197/f1p12/2156	0	14.59608445	-Inf	0.00046288	0.0042646
i3_LQ_YHS_c3533/f1p1/3871	143.2317205	33.02180763	2.1169	0.00046326	0.0042674
i2_LQ_YHS_c12818/f1p1/2179	228.0810099	65.50573973	1.7999	0.00046365	0.0042688
i2_LQ_YHS_c28882/f1p0/2067	242.4146558	73.24531302	1.7267	0.00046369	0.0042688
i3_LQ_YHS_c13560/f1p1/3113	14.27302345	81.80171501	-2.5188	0.00046356	0.0042688

i2_LQ_YHS_c56143/f1p0/2691	0	14.5793779	-Inf	0.00046419	0.0042727
i1_LQ_YHS_c9012/f1p1/1531	543.1147426	2056.873525	-1.9211	0.00046549	0.0042838
i2_LQ_YHS_c56624/f1p1/2782	6.854311627	152.8089469	-4.4786	0.00046744	0.0043009
i2_LQ_YHS_c22880/f1p1/2894	37.17195442	152.630979	-2.0378	0.00046806	0.0043058
i2_HQ_YHS_c19675/f2p3/2835	7.887406974	283.5613733	-5.168	0.00046845	0.0043086
i2_LQ_YHS_c53868/f1p1/2425	41.52529359	4.012224098	3.3715	0.00046859	0.004309
i3_LQ_YHS_c2996/f1p0/3377	0	14.51930685	-Inf	0.00046895	0.0043115
i3_LQ_YHS_c3571/f1p0/3332	184.829239	4.78450275	5.2717	0.00046973	0.0043179
i1_LQ_YHS_c26302/f2p0/1934	288.813184	0	Inf	0.00047024	0.0043213
i1_LQ_YHS_c28416/f1p1/1073	0	14.5026003	-Inf	0.00047028	0.0043213
i1_LQ_YHS_c18983/f2p0/1809	184.5698692	934.1583149	-2.3395	0.00047086	0.0043259
i1_LQ_YHS_c12753/f1p2/1735	4.486715426	43.10057211	-3.264	0.00047099	0.0043262
i2_LQ_YHS_c50571/f1p7/3012	256.4515885	816.9970405	-1.6716	0.00047147	0.0043298
i1_HQ_YHS_c9702/f2p0/1762	10.17694655	168.6173519	-4.0504	0.00047186	0.0043325
i2_HQ_YHS_c3810/f2p0/2399	284.61513	901.0570954	-1.6626	0.00047216	0.0043339
i2_LQ_YHS_c36298/f1p0/2145	0.359624837	33.92390786	-6.5597	0.00047219	0.0043339
i2_LQ_YHS_c35942/f1p0/2728	27.93541756	1.415488932	4.3027	0.00047238	0.0043349
i2_HQ_YHS_c30208/f2p6/2389	1135.206656	10672.68786	-3.2329	0.00047345	0.0043439
i1_LQ_YHS_c18448/f1p0/1720	68.78963173	239.2427901	-1.7982	0.00047382	0.0043464
i6_LQ_YHS_c997/f1p0/6110	99.75591494	1.061616699	6.5541	0.0004742	0.0043491
i1_HQ_YHS_c9240/f2p2/1524	1165.128052	353.6003159	1.7203	0.00047678	0.0043719
i2_LQ_YHS_c41239/f1p1041/2832	33.59314387	11579.44654	-8.4292	0.0004777	0.0043795
i2_LQ_YHS_c64752/f1p0/2014	109.4183453	360.3149236	-1.7194	0.00047952	0.0043953
i2_LQ_YHS_c11023/f1p2/2453	50.39239227	306.5452268	-2.6048	0.00047967	0.0043959
i1_HQ_YHS_c2278/f2p1/1971	512.9120916	163.1898795	1.6522	0.00047978	0.0043961
i2_LQ_YHS_c26107/f1p0/2095	73.77357034	0	Inf	0.00048012	0.0043983
i2_LQ_YHS_c23742/f2p1/2918	449.6698617	13.12162981	5.0988	0.00048104	0.0044059
i3_LQ_YHS_c18571/f1p3/3691	15.07367944	81.02390743	-2.4263	0.00048236	0.0044172
i4_LQ_YHS_c13235/f1p0/4129	0	14.34904511	-Inf	0.00048278	0.0044202
i3_LQ_YHS_c3984/f1p2/3217	8.482482192	58.49073722	-2.7856	0.0004837	0.0044278
i5_LQ_YHS_c4146/f1p0/5543	0	24.9951883	-Inf	0.00048431	0.0044326
i1_LQ_YHS_c21750/f1p3/1751	316.806499	0	Inf	0.0004849	0.0044371
i2_LQ_YHS_c7234/f1p1/2167	30.88631777	150.4051819	-2.2838	0.00048536	0.0044405
i3_LQ_YHS_c7302/f1p0/3115	21.87713171	163.3376663	-2.9004	0.00048557	0.0044416
i0_LQ_YHS_c1950/f1p0/956	36.29039235	0	Inf	0.00048568	0.0044417
i2_HQ_YHS_c45699/f19p2/2421	551.8714079	2108.814814	-1.934	0.00048599	0.0044437
i1_LQ_YHS_c9822/f1p1/1862	180.7052252	48.82812781	1.8879	0.00048644	0.004447
i2_LQ_YHS_c20712/f1p3/2221	1.588735364	37.06195544	-4.544	0.00048662	0.0044478
i1_HQ_YHS_c37372/f9p1/1297	4842.519496	157.5179257	4.9422	0.00048692	0.0044497
i1_LQ_YHS_c33494/f1p0/1740	90.29759814	293.6138753	-1.7012	0.0004875	0.0044542
i1_LQ_YHS_c27720/f1p0/1205	274.2702906	861.3290124	-1.651	0.00048763	0.0044545
i5_LQ_YHS_c2262/f1p0/5054	1.089365194	24.83164104	-4.5106	0.00048888	0.0044651
i2_LQ_YHS_c53092/f1p3/2320	421.8753479	1441.220781	-1.7724	0.00048963	0.0044711
i3_LQ_YHS_c11402/f1p1/3121	186.3544342	591.5685695	-1.6665	0.00049006	0.0044741
i4_LQ_YHS_c6269/f1p13/4904	124.0758081	22.57933927	2.4581	0.00049029	0.0044754
i3_LQ_YHS_c19524/f1p4/3693	11.18491381	67.6884514	-2.5974	0.0004906	0.0044774
i1_LQ_YHS_c33269/f1p0/1573	25.86161076	114.1686222	-2.1423	0.00049092	0.0044791
i3_LQ_YHS_c7364/f1p4/3881	170.6859357	40.78148481	2.0654	0.00049098	0.0044791
i1_LQ_YHS_c35452/f1p2/1809	288.3854536	915.5538135	-1.6666	0.00049182	0.004486
i2_HQ_YHS_c11478/f2p13/2693	1149.820081	8601.247713	-2.9031	0.00049208	0.0044875
i1_LQ_YHS_c7004/f1p25/1980	71.89125145	245.4606727	-1.7716	0.00049225	0.0044883
i1_HQ_YHS_c40977/f3p3/1223	12478.06969	903.5380213	3.7877	0.00049287	0.004493
i2_LQ_YHS_c51723/f1p2/2418	2.467447971	31.44731479	-3.6718	0.00049432	0.0045054
i1_LQ_YHS_c25828/f1p0/1870	138.9325652	435.7416837	-1.6491	0.00049482	0.0045091
i0_HQ_YHS_c369/f2p0/572	755.8226888	22664.55313	-4.9062	0.00049499	0.0045098
i1_HQ_YHS_c5992/f6p0/1902	6528.821535	0	Inf	0.00049539	0.0045126
i2_HQ_YHS_c7012/f2p7/2329	3.87908035	63.58827208	-4.035	0.00049561	0.0045137
i2_LQ_YHS_c50940/f1p3/2150	588.898228	2269.327787	-1.9462	0.00049647	0.0045208
i1_LQ_YHS_c21709/f1p1/1752	289.873351	86.95774257	1.737	0.00049696	0.0045243
i2_HQ_YHS_c30164/f4p13/2681	1122.461716	7124.541507	-2.6661	0.00049859	0.0045383
i0_LQ_YHS_c2824/f1p1/916	572.0494342	59.43434341	3.2668	0.00050053	0.0045543
i2_LQ_YHS_c52842/f1p16/2752	2213.406705	11.37556991	7.6042	0.00050053	0.0045543
i1_LQ_YHS_c29139/f1p5/1327	1.361706493	26.61605019	-4.2888	0.00050131	0.0045596
i3_LQ_YHS_c4972/f1p2/3282	32.16790684	0.719987877	5.4815	0.00050127	0.0045596
i1_LQ_YHS_c33832/f1p0/1964	32.84009684	135.6791573	-2.0467	0.00050182	0.0045634
i2_LQ_YHS_c40835/f1p2/3159	4.902143374	45.40018896	-3.2112	0.00050264	0.00457
i2_LQ_YHS_c53669/f1p0/2541	192.0988983	1163.88251	-2.599	0.00050288	0.0045713
i2_LQ_YHS_c38564/f1p0/2737	6.265122009	49.95933462	-2.9953	0.00050317	0.0045731
i0_HQ_YHS_c3688/f2p0/650	1162.937008	9203.997534	-2.9845	0.00050385	0.0045784
i1_LQ_YHS_c26493/f1p1/1647	42.06582947	1.439975754	4.8685	0.00050413	0.0045801
i2_LQ_YHS_c53263/f1p0/2702	18.63048423	98.08821078	-2.3964	0.0005045	0.0045826
i2_LQ_YHS_c54256/f1p6/2472	6101.009041	963.4910265	2.6627	0.00050487	0.0045851
i2_HQ_YHS_c2393/f2p5/2708	219.465514	678.2961002	-1.6279	0.00050749	0.0046072
i2_LQ_YHS_c25257/f1p1/2917	3.052829538	35.93023608	-3.557	0.0005074	0.0046072
i1_LQ_YHS_c12530/f1p0/1503	339.0596103	6.828707208	5.6338	0.00050764	0.0046077
i2_LQ_YHS_c24039/f1p15/2738	0.817023896	31.28585956	-5.259	0.00050954	0.004624
i2_LQ_YHS_c6660/f1p0/2420	299.1606898	69.5724664	2.1043	0.00051067	0.0046334
i3_LQ_YHS_c13525/f1p4/3499	220.7017667	17.49546686	3.657	0.00051104	0.0046359
i2_LQ_YHS_c44621/f4p1/3033	26.59975588	121.597847	-2.1926	0.00051194	0.0046432
i3_LQ_YHS_c11523/f1p0/3879	104.5088048	333.2838248	-1.6731	0.00051241	0.0046466
i2_LQ_YHS_c36717/f1p17/2675	1000.142354	5387.427765	-2.4294	0.00051478	0.0046672
i3_LQ_YHS_c13611/f1p5/3505	168.0932601	527.0769386	-1.6488	0.00051514	0.0046696
i2_LQ_YHS_c20638/f1p17/2991	58.3848509	209.2804741	-1.8418	0.00051718	0.0046863
i3_LQ_YHS_c5448/f1p2/3412	57.75911741	199.9107198	-1.7912	0.00051712	0.0046863
i3_LQ_YHS_c11239/f1p12/3593	141.4920299	445.6027505	-1.655	0.00051782	0.0046912
i2_HQ_YHS_c8910/f2p2/2139	72.66123242	243.5276841	-1.7448	0.00051797	0.0046917
i3_HQ_YHS_c15612/f6p1/3113	374.0228559	119.8684146	1.6417	0.00051901	0.0047002
i4_LQ_YHS_c8914/f1p19/4444	553.2061038	60.03750632	3.2039	0.00052045	0.0047124
i2_LQ_YHS_c41625/f1p10/2173	7662.541719	110.1798982	6.1199	0.00052097	0.0047156

i3_LQ_YHS_c14204/f1p2/3071	445.8228241	1425.091401	-1.6765	0.00052109	0.0047156
i4_HQ_YHS_c2240/f2p1/4938	315.9804183	1073.8811	-1.7649	0.00052104	0.0047156
i1_LQ_YHS_c5526/f1p2/1465	114.1023899	2.2061327	5.6927	0.00052128	0.0047164
i2_HQ_YHS_c988/f12p4/2814	282.9823999	84.35891537	1.7461	0.00052271	0.0047284
i1_LQ_YHS_c22900/f1p3/1561	51.35804975	184.39437	-1.8441	0.00052341	0.0047339
i3_LQ_YHS_c17398/f1p0/3253	23.35139464	0	Inf	0.00052406	0.0047389
i2_LQ_YHS_c19547/f1p4/2283	1.623557107	26.64938307	-4.0369	0.00052511	0.0047475
i3_LQ_YHS_c4220/f1p3/3089	13.40768448	74.8962302	-2.4818	0.00052571	0.004752
i2_LQ_YHS_c6520/f1p0/3007	0	37.46258952	-Inf	0.00052759	0.0047681
i1_HQ_YHS_c39859/f65p0/1521	298.4554552	35193.91861	-6.8817	0.0005281	0.0047719
i5_LQ_YHS_c2360/f1p0/5374	444.2673992	148.6482988	1.5795	0.00052824	0.0047723
i2_LQ_YHS_c5172/f1p2/2665	9.208422872	55.56461665	-2.5931	0.00053028	0.0047898
i3_LQ_YHS_c14212/f1p3/3045	87.58801715	283.6882732	-1.6955	0.00053166	0.0048014
i2_LQ_YHS_c20838/f1p0/2122	820.1263472	153.452272	2.4181	0.00053205	0.004804
i2_LQ_YHS_c24650/f1p0/2406	133.994675	837.0877251	-2.6432	0.00053233	0.0048047
i2_LQ_YHS_c37004/f1p3/2229	45.10468219	169.3325641	-1.9085	0.0005323	0.0048047
i1_LQ_YHS_c17753/f1p3/1486	820.0463542	112.768101	2.8623	0.00053338	0.0048133
i2_LQ_YHS_c50473/f1p0/2166	4.419149489	40.87666707	-3.2094	0.00053378	0.0048151
i4_LQ_YHS_c13247/f1p0/4150	0	14.32677008	-Inf	0.00053377	0.0048151
i1_LQ_YHS_c8460/f1p7/1795	4.411194714	76.81910589	-4.1222	0.00053434	0.0048192
i2_LQ_YHS_c23966/f1p4/2592	0	18.4452343	-Inf	0.00053673	0.0048399
i2_LQ_YHS_c24333/f1p1/2617	34.80437496	134.1124842	-1.9461	0.00053708	0.0048421
i3_LQ_YHS_c8240/f1p3/3387	36.48548243	0	Inf	0.00053812	0.0048506
i1_LQ_YHS_c27828/f1p1/1356	2903.270891	708.5449029	2.0347	0.00053868	0.0048548
i1_LQ_YHS_c32630/f1p3/1693	254.41035	6.135457496	5.3738	0.00054042	0.0048695
i2_HQ_YHS_c12374/f2p0/2199	94.28942042	303.3864765	-1.686	0.00054141	0.0048775
i2_LQ_YHS_c25233/f1p0/2220	15.16048787	83.51372948	-2.4617	0.00054158	0.0048782
i1_LQ_YHS_c13846/f1p0/1374	38.84703985	347.980454	-3.1631	0.00054183	0.0048795
i5_LQ_YHS_c1178/f1p0/5808	2.693204701	34.61376022	-3.6839	0.00054285	0.0048878
i4_LQ_YHS_c12974/f2p2/4030	38.68690794	236.9957815	-2.6149	0.00054348	0.0048916
i4_LQ_YHS_c3878/f1p2/4301	141.6579732	36.25962149	1.966	0.00054343	0.0048916
i3_LQ_YHS_c12475/f1p0/3947	28.41967521	122.5692978	-2.1086	0.00054362	0.0048919
i2_LQ_YHS_c10011/f1p5/2138	20.2611907	99.33845433	-2.2936	0.00054502	0.0049036
i3_LQ_YHS_c18991/f1p0/3617	0	14.20101885	-Inf	0.00054529	0.0049052
i7_LQ_YHS_c45/f2p0/7429	4.306271124	56.36461904	-3.7103	0.00054575	0.0049084
i2_LQ_YHS_c40498/f1p0/2936	258.5466675	6.913857853	5.2248	0.0005468	0.0049169
i1_LQ_YHS_c25082/f1p0/1821	275.8954657	877.9160272	-1.67	0.00054835	0.004929
i2_LQ_YHS_c43445/f1p1/2038	10.06787624	141.8909578	-3.817	0.00054835	0.004929
i3_LQ_YHS_c9136/f1p0/3412	0	27.67035963	-Inf	0.00054876	0.0049318
i2_LQ_YHS_c19094/f1p0/2437	0	14.15484978	-Inf	0.0005496	0.0049384
i1_LQ_YHS_c13147/f1p2/1931	178.8305762	3.376794089	5.7268	0.00054974	0.0049387
i1_LQ_YHS_c12547/f1p0/1800	5360.283912	1010.518473	2.4072	0.00055017	0.0049417
i2_LQ_YHS_c28195/f1p4/2110	33.98688433	131.6452102	-1.9536	0.00055066	0.0049452
i1_LQ_YHS_c12102/f1p5/1790	3.083037822	62.78151454	-4.3479	0.00055116	0.0049487
i3_LQ_YHS_c7446/f1p0/3241	79.34195949	343.1813846	-2.1128	0.00055232	0.0049583
i2_LQ_YHS_c5154/f1p5/2161	58.48112563	0	Inf	0.00055264	0.0049602
i2_LQ_YHS_c3038/f1p3/2766	196.2732557	58.83220674	1.7382	0.00055282	0.0049609
i2_LQ_YHS_c17951/f1p5/2906	620.5124826	201.8121133	1.6204	0.00055333	0.0049646
i2_LQ_YHS_c4435/f1p2/2887	3.149339992	33.01517336	-3.39	0.00055462	0.0049752
i1_HQ_YHS_c26223/f3p0/1690	624.2247736	2387.129837	-1.9351	0.00055474	0.0049754
i1_LQ_YHS_c6954/f1p2/1618	54.58336044	2579.230689	-5.5623	0.00055491	0.004976
i2_LQ_YHS_c18063/f1p6/2305	65.61961555	298.9393383	-2.1877	0.00055561	0.0049813
i1_HQ_YHS_c2271/f3p34/1783	1319.034225	361.1460867	1.8688	0.00055604	0.0049837
i2_LQ_YHS_c19664/f1p0/2195	75.55680463	250.5301701	-1.7294	0.00055608	0.0049837
i0_LQ_YHS_c863/f1p1/373	30.23545619	1.961305157	3.9464	0.00055631	0.004984
i1_LQ_YHS_c10141/f1p3/1779	0	17.75585495	-Inf	0.00055633	0.004984
i1_LQ_YHS_c17837/f1p0/1983	590.509687	0	Inf	0.00055787	0.0049969
i1_HQ_YHS_c17007/f2p0/1579	305.8375502	975.8771441	-1.6739	0.00055823	0.0049984
i1_LQ_YHS_c10330/f1p0/2006	120.0659449	28.73762502	2.0628	0.00055824	0.0049984
i1_LQ_YHS_c29083/f1p0/1210	5.550485794	45.54319885	-3.0365	0.00055835	0.0049985
i3_LQ_YHS_c7199/f1p6/3487	40.36928756	0	Inf	0.00055866	0.0050002
i3_LQ_YHS_c7263/f1p3/3809	605.3891765	196.1382674	1.626	0.00055875	0.0050002
i3_HQ_YHS_c6229/f2p0/3979	0	14.04580509	-Inf	0.00055996	0.0050101
i1_LQ_YHS_c25831/f1p2/1654	126.2921068	31.29372005	2.0128	0.00056039	0.005013
i1_LQ_YHS_c35922/f1p2/1796	28.5010899	117.1454145	-2.0392	0.00056135	0.0050207
i1_HQ_YHS_c10687/f2p1/1613	124.4348298	392.8404815	-1.6586	0.00056405	0.0050429
i3_LQ_YHS_c11727/f1p0/3746	17.4546409	89.43879765	-2.3573	0.00056399	0.0050429
i1_LQ_YHS_c4149/f1p0/1706	274.51318	83.2066191	1.7221	0.00056462	0.0050471
i3_LQ_YHS_c10676/f1p4/3543	21.63673757	141.891233	-2.7132	0.00056824	0.0050782
i4_LQ_YHS_c10300/f1p0/4661	86.91695449	279.7630198	-1.6865	0.0005683	0.0050782
i3_LQ_YHS_c12997/f1p0/4005	86.85238282	285.9110333	-1.7189	0.00056914	0.0050847
i1_LQ_YHS_c3608/f1p4/1567	156.8902156	34.95377003	2.1662	0.00057107	0.005101
i1_LQ_YHS_c32983/f1p4/1701	184.9714867	26.61158705	2.7972	0.00057155	0.0051044
i2_HQ_YHS_c5525/f2p3/2611	14.45520663	213.7746186	-3.8864	0.00057262	0.0051129
i2_LQ_YHS_c12791/f1p0/2772	65.33903942	10.45645055	2.6436	0.0005738	0.0051226
i1_HQ_YHS_c8404/f2p7/1785	192.8708144	7.009000566	4.7823	0.00057582	0.0051396
i0_LQ_YHS_c1214/f1p0/908	1266.956736	378.180337	1.7442	0.00057628	0.0051428
i1_LQ_YHS_c24156/f1p0/1754	3552.910117	729.2796807	2.2845	0.00057681	0.0051466
i1_HQ_YHS_c8923/f5p0/1519	638.3092136	2482.915693	-1.9597	0.00057768	0.0051534
i4_LQ_YHS_c6326/f1p0/4773	21.88093976	200.9286523	-3.1989	0.00057838	0.0051587
i2_LQ_YHS_c64824/f1p0/2013	589.4697716	2148.189617	-1.8656	0.00057877	0.0051612
i2_LQ_YHS_c24383/f1p2/2477	39.21300865	153.8375444	-1.972	0.00057942	0.0051661
i1_LQ_YHS_c36922/f1p421/1436	19.20491675	92.55516411	-2.2688	0.00057968	0.0051673
i2_LQ_YHS_c1510/f4p1/2695	1285.924774	372.9461451	1.7858	0.00057978	0.0051673
i1_LQ_YHS_c36322/f1p3/1643	14.97797439	79.89104206	-2.4152	0.00057991	0.0051675
i3_HQ_YHS_c1247/f8p0/3534	114.5998848	353.5013842	-1.6251	0.00058034	0.0051705
i1_LQ_YHS_c18286/f1p0/1726	17.42984311	182.4823929	-3.3881	0.00058075	0.0051731
i3_HQ_YHS_c21048/f15p11/3484	1930.926233	533.191661	1.8566	0.00058276	0.0051901
i2_LQ_YHS_c64560/f1p1/1969	62.25855426	0	Inf	0.00058341	0.0051949

i1_LQ_YHS_c4251/f1p1/1519	2003.916687	555.8251507	1.8501	0.00058354	0.0051951
i1_LQ_YHS_c35433/f1p0/1588	90.87822981	291.4691201	-1.6813	0.0005845	0.0052027
i1_LQ_YHS_c3295/f1p17/1880	0.272341299	17.19226638	-5.9802	0.00058505	0.0052066
i3_LQ_YHS_c2776/f1p0/3950	9.053565023	59.07043956	-2.7059	0.00058551	0.0052098
i2_LQ_YHS_c3436/f1p1/2740	10.06613736	238.0252904	-4.5635	0.00058599	0.0052131
i2_LQ_YHS_c24062/f1p1/2190	83.00067805	1.176783094	6.1402	0.00058634	0.0052152
i3_HQ_YHS_c4042/f3p1/3855	324.6952512	9.972917165	5.0249	0.0005871	0.005221
i2_HQ_YHS_c61058/f2p1/2129	152.1708657	475.6935887	-1.6443	0.00058726	0.0052215
i2_LQ_YHS_c19095/f1p2/2167	0	13.76484009	-Inf	0.00058786	0.0052254
i2_LQ_YHS_c55564/f1p1/2579	14.82566082	81.51067885	-2.4589	0.00058792	0.0052254
i3_HQ_YHS_c2125/f2p0/3496	66.61945092	231.5371424	-1.7972	0.00058885	0.0052327
i0_LQ_YHS_c1606/f1p7/664	1208.208911	368.830641	1.7118	0.0005893	0.0052358
i1_LQ_YHS_c17679/f1p0/1996	33.90974443	136.36364	-2.0077	0.00059057	0.0052461
i3_HQ_YHS_c15557/f4p0/3666	344.2043068	21.55895549	3.9969	0.00059327	0.0052691
i1_LQ_YHS_c7276/f1p0/1881	28.88804006	1.799969692	4.0044	0.00059482	0.005281
i4_HQ_YHS_c1765/f4p0/4471	633.5874822	171.4358145	1.8859	0.00059475	0.005281
i2_LQ_YHS_c49649/f1p0/2172	91.89602716	19.62482197	2.2273	0.00059503	0.0052818
i0_LQ_YHS_c1966/f1p0/689	4063.408619	25.510396	7.3155	0.00059715	0.0052997
i2_LQ_YHS_c4225/f1p2/2052	104.409123	317.5566138	-1.6048	0.00059745	0.0053004
i2_LQ_YHS_c51878/f1p20/2224	6.710758242	50.86747634	-2.9222	0.00059738	0.0053004
i1_LQ_YHS_c23437/f1p2/2006	10562.36461	0.707744466	13.865	0.00059937	0.0053159
i1_LQ_YHS_c3961/f1p4/1915	0	40.54822769	-Inf	0.00059942	0.0053159
i2_LQ_YHS_c33808/f1p2/2265	48.50089672	178.8531096	-1.8827	0.00060372	0.0053531
i3_LQ_YHS_c9306/f1p0/3434	38.24367956	171.6570617	-2.1662	0.00060389	0.0053536
i3_LQ_YHS_c5193/f1p0/3547	150.4780206	464.7554551	-1.6269	0.00060444	0.0053575
i1_HQ_YHS_c16830/f3p0/1718	757.0749349	241.8044524	1.6466	0.00060539	0.0053649
i2_HQ_YHS_c17587/f2p3/2782	52.1876335	255.1651029	-2.2897	0.0006067	0.0053755
i1_LQ_YHS_c23830/f1p4/1830	22.15122026	0.719987877	4.9433	0.00060693	0.0053766
i2_LQ_YHS_c12359/f1p41/2881	116.9141443	0	Inf	0.00060719	0.0053776
i3_LQ_YHS_c5373/f1p16/3452	610.9780133	2283.851183	-1.9023	0.00060727	0.0053776
i1_LQ_YHS_c25415/f1p0/1926	607.8118985	198.7779347	1.6125	0.00060742	0.005378
i2_LQ_YHS_c5815/f1p1/2298	103.2119596	1.07386011	6.5867	0.00060772	0.0053796
i1_LQ_YHS_c11457/f1p0/1402	34.33312716	137.7662532	-2.0045	0.00060938	0.0053933
i1_HQ_YHS_c2730/f3p1/1491	502.0609756	68.54852596	2.8727	0.00060982	0.0053962
i1_LQ_YHS_c3223/f1p0/1720	48.25081731	4.881304028	3.3052	0.00061295	0.0054219
i2_LQ_YHS_c49148/f1p1/2452	99.38921855	330.5334949	-1.7336	0.00061285	0.0054219
i1_LQ_YHS_c14379/f1p17/1213	143.947417	468.9638479	-1.7039	0.00061379	0.0054284
i0_LQ_YHS_c1166/f1p1/666	259.5744325	1949.132032	-2.9086	0.00061396	0.0054289
i2_LQ_YHS_c8475/f1p8/2565	42.80341328	0	Inf	0.00061507	0.0054378
i1_LQ_YHS_c5354/f1p0/1461	331.3127098	1833.543307	-2.4684	0.00061543	0.0054399
i2_LQ_YHS_c23132/f1p2/2167	27.40791665	1.439975754	4.2505	0.00061577	0.0054419
i1_HQ_YHS_c7147/f3p0/1532	273.9428439	870.8300848	-1.6685	0.00061672	0.0054494
i1_LQ_YHS_c36107/f1p0/1795	37.5830992	254.4224928	-2.7591	0.00061746	0.0054549
i3_LQ_YHS_c8615/f1p0/3255	21.221318	0.353872233	5.9061	0.00061857	0.0054637
i2_LQ_YHS_c25351/f1p3/2740	557.7454893	183.7797564	1.6016	0.00061877	0.0054645
i0_LQ_YHS_c1428/f1p0/756	5872.149091	16.56682844	8.4694	0.00062004	0.0054747
i2_LQ_YHS_c33966/f1p0/2952	326.4571032	102.5003971	1.6713	0.00062231	0.0054937
i2_LQ_YHS_c6630/f1p0/2485	69.24240896	236.0892192	-1.7696	0.00062659	0.0055305
i2_LQ_YHS_c4254/f1p14/2617	124.514745	958.3425741	-2.9442	0.00062677	0.005531
i1_LQ_YHS_c3779/f1p0/1687	29.17248286	118.6605488	-2.0242	0.00062787	0.0055397
i2_LQ_YHS_c22103/f1p2/2152	15.77735823	80.80526482	-2.3566	0.00062832	0.0055427
i1_LQ_YHS_c6867/f1p0/1942	356.6431289	1101.567935	-1.627	0.00062856	0.0055428
i3_LQ_YHS_c8041/f1p0/3262	48.94988264	174.6457837	-1.8351	0.00062844	0.0055428
i2_HQ_YHS_c6221/f5p4/2668	137.6105417	429.2480106	-1.6412	0.00062916	0.0055471
i4_LQ_YHS_c7685/f1p1/4969	0.257237156	41.57667081	-7.3365	0.00062965	0.0055504
i2_HQ_YHS_c13728/f3p0/2560	0	58.06281287	-Inf	0.00063093	0.0055606
i3_LQ_YHS_c20459/f1p0/3070	60.57294465	9.587370615	2.6595	0.00063111	0.0055612
i2_LQ_YHS_c18254/f1p0/2460	63.37762751	10.26450655	2.6263	0.00063132	0.0055621
i1_HQ_YHS_c1566/f12p6/1614	1782.137441	509.5166596	1.8064	0.00063158	0.0055633
i2_LQ_YHS_c49870/f1p13/2109	246.062995	1.079981815	7.8319	0.00063205	0.0055664
i4_LQ_YHS_c6391/f1p0/4274	0	13.89777882	-Inf	0.00063312	0.0055749
i4_LQ_YHS_c8184/f1p2/4314	72.14401996	0.359993938	7.6468	0.00063385	0.0055803
i1_HQ_YHS_c17054/f2p0/1952	627.4811447	206.3956666	1.6042	0.00063473	0.005587
i3_LQ_YHS_c14954/f1p3/3028	8.367059544	58.59247466	-2.8079	0.0006374	0.0056095
i3_LQ_YHS_c12919/f1p3/3383	173.9966642	25.5856348	2.7657	0.0006381	0.0056136
i4_LQ_YHS_c11987/f1p11/4532	10.00952884	65.12959247	-2.7019	0.00063808	0.0056136
i3_LQ_YHS_c18881/f1p0/3571	962.1373666	242.7526811	1.9868	0.00063932	0.0056233
i2_LQ_YHS_c34047/f1p2/2282	106.3894136	15.2915855	2.7985	0.00064035	0.0056314
i2_LQ_YHS_c6777/f1p1/2228	40.44087215	147.7604994	-1.8694	0.00064066	0.0056331
i2_HQ_YHS_c25872/f3p0/2861	0	36.79141598	-Inf	0.00064169	0.005638
i2_LQ_YHS_c19932/f1p0/2493	0	13.82100122	-Inf	0.00064148	0.005638
i2_LQ_YHS_c5885/f1p0/2205	59.63083795	0	Inf	0.0006416	0.005638
i6_LQ_YHS_c732/f1p0/6665	0	13.81934266	-Inf	0.00064166	0.005638
i2_LQ_YHS_c6820/f1p0/2460	2.897980062	33.84033556	-3.5456	0.00064216	0.0056411
i1_LQ_YHS_c3404/f1p0/1753	349.8703193	46.45793529	2.9128	0.00064229	0.0056412
i1_LQ_YHS_c10607/f1p0/1768	132.5925257	33.96328223	1.965	0.00064274	0.0056435
i5_HQ_YHS_c588/f3p0/5399	0	27.29812228	-Inf	0.00064278	0.0056435
i2_HQ_YHS_c18043/f2p0/2822	324.2200337	92.09236695	1.8158	0.00064324	0.0056463
i2_LQ_YHS_c29412/f1p0/2055	46.76895502	164.6221146	-1.8155	0.00064333	0.0056463
i2_LQ_YHS_c40922/f1p2/2582	74.33791192	246.6480418	-1.7303	0.0006435	0.0056467
i2_HQ_YHS_c11556/f3p0/2434	114.9227958	360.1245987	-1.6478	0.00064447	0.0056542
i1_LQ_YHS_c34026/f1p5/1535	106.1353897	324.1709009	-1.6108	0.00064524	0.0056589
i2_LQ_YHS_c22935/f1p2/2756	127.5431489	31.497947	2.0177	0.00064519	0.0056589
i0_LQ_YHS_c2768/f1p0/794	0	13.77483215	-Inf	0.00064658	0.0056692
i2_LQ_YHS_c50490/f1p1/2795	0	13.77317359	-Inf	0.00064676	0.0056692
i3_LQ_YHS_c3337/f1p0/3106	0	27.27975717	-Inf	0.00064673	0.0056692
i2_HQ_YHS_c9234/f3p4/2345	614.6780675	203.7015374	1.5934	0.00064783	0.0056775
i2_LQ_YHS_c14538/f1p0/2060	267.1249493	14.368003	4.2166	0.00064861	0.0056833
i6_LQ_YHS_c1043/f1p0/6049	0	15.87744909	-Inf	0.0006488	0.005684

i2_HQ_YHS_c62271/f4p7/2692	306.0059971	88.76774387	1.7855	0.00065103	0.0057014
i2_LQ_YHS_c29048/f1p1/2069	48.14842962	4.378339741	3.459	0.00065101	0.0057014
i2_HQ_YHS_c1412/f6p6/2725	13.86047008	171.3047282	-3.6275	0.00065128	0.0057026
i2_LQ_YHS_c39166/f1p11/3704	502.7849441	1807.66091	-1.8461	0.00065197	0.0057068
i2_LQ_YHS_c59954/f1p1/2164	31.13466668	127.2799067	-2.0314	0.000652	0.0057068
i4_HQ_YHS_c1770/f5p0/4901	49.41475299	198.7076705	-2.0076	0.00065249	0.0057101
i2_LQ_YHS_c3662/f1p0/2442	96.06702997	20.68537288	2.2154	0.00065269	0.0057108
i4_LQ_YHS_c7417/f1p2/4358	2.754893412	48.53428708	-4.1389	0.00065316	0.0057138
i2_LQ_YHS_c58359/f1p3/2339	159.2627543	808.9438438	-2.3446	0.00065338	0.0057148
i3_HQ_YHS_c3074/f2p1/3255	51.91968668	4.681579765	3.4712	0.00065357	0.0057154
i1_LQ_YHS_c20649/f1p7/1434	106.5026306	24.18843076	2.1385	0.00065449	0.0057219
i2_LQ_YHS_c38461/f1p0/2145	189.8275588	3.266090833	5.861	0.00065456	0.0057219
i2_LQ_YHS_c39906/f2p1/2475	493.6669056	93.71871822	2.3971	0.00065472	0.0057223
i3_LQ_YHS_c6334/f1p3/3246	85.91835059	273.9511782	-1.6729	0.00065588	0.0057315
i2_LQ_YHS_c33013/f1p4/2431	1050.450719	35.95416967	4.8687	0.00065657	0.0057364
i1_LQ_YHS_c10776/f1p3/1808	37.8857936	147.8416203	-1.9643	0.00065725	0.0057402
i2_LQ_YHS_c21149/f1p1/2514	1.968077802	27.28094272	-3.793	0.00065735	0.0057402
i3_LQ_YHS_c6323/f1p1/3122	120.0322672	371.568021	-1.6302	0.00065733	0.0057402
i3_LQ_YHS_c14166/f1p1/3040	59.67090025	592.7534394	-3.3123	0.00065772	0.0057423
i2_LQ_YHS_c50558/f1p0/2659	121.2040057	570.060833	-2.2337	0.00065978	0.0057593
i2_LQ_YHS_c61870/f61p6/2249	2943.729515	363.2180555	3.0187	0.00066043	0.0057639
i1_LQ_YHS_c4740/f1p9/1785	45.79927888	5.453858417	3.07	0.00066101	0.0057679
i2_HQ_YHS_c60184/f2p1/2770	181.3583331	36.00650112	2.3325	0.00066135	0.0057698
i1_LQ_YHS_c43519/f1p0/1039	1162.996972	1.427732343	9.6699	0.00066222	0.0057764
i4_LQ_YHS_c3011/f1p0/4357	4.081778162	40.46485537	-3.3094	0.0006625	0.0057778
i3_LQ_YHS_c10466/f1p0/3345	19.11475863	129.0181863	-2.7548	0.00066296	0.0057808
i3_LQ_YHS_c14516/f1p1/3040	747.9204172	226.5943483	1.7228	0.00066361	0.0057854
i1_LQ_YHS_c36978/f1p0/1661	391.7253932	29.95445548	3.709	0.00066526	0.0057987
i1_LQ_YHS_c39592/f1p3/1293	380.3832413	123.2068594	1.6264	0.00066574	0.0058001
i2_LQ_YHS_c2848/f1p7/2341	2314.808816	25.99396893	6.4766	0.00066578	0.0058001
i3_LQ_YHS_c6072/f1p4/3816	94.29704489	296.1160221	-1.6509	0.00066566	0.0058001
i1_LQ_YHS_c12288/f1p4/1942	242.8497235	73.25262028	1.7291	0.00066906	0.0058276
i2_LQ_YHS_c51242/f1p1/2779	494.7423765	162.2914568	1.6081	0.0006714	0.0058469
i3_HQ_YHS_c13872/f2p0/3094	318.577493	99.04066302	1.6856	0.0006716	0.0058476
i1_LQ_YHS_c32778/f1p3/1850	0	23.85845257	-Inf	0.00067198	0.0058499
i4_LQ_YHS_c4116/f1p3/4855	12.63597301	71.14542044	-2.4932	0.00067344	0.0058616
i2_LQ_YHS_c41380/f1p3/2178	18.83145154	91.42305083	-2.2794	0.0006738	0.0058637
i0_LQ_YHS_c2420/f1p0/960	0	13.52613425	-Inf	0.00067498	0.0058715
i2_LQ_YHS_c14479/f1p1/2047	3.737257468	38.39734964	-3.361	0.00067506	0.0058715
i2_LQ_YHS_c9256/f1p0/2130	79.22016058	15.15805399	2.3858	0.0006749	0.0058715
i2_LQ_YHS_c37504/f1p1/2917	36.19930076	136.2071209	-1.9118	0.00067526	0.0058721
i4_LQ_YHS_c11668/f1p0/4640	0	13.52001254	-Inf	0.00067569	0.0058748
i1_LQ_YHS_c7536/f1p1/1804	113.2518404	362.6087732	-1.6789	0.00067871	0.0059
i1_LQ_YHS_c33116/f1p0/1819	119.1321149	364.2344503	-1.6123	0.00067904	0.0059018
i2_LQ_YHS_c54373/f1p6/2583	1943.288042	441.01134	2.1396	0.00067951	0.0059048
i3_LQ_YHS_c12216/f1p1/3151	0	20.43714798	-Inf	0.00068181	0.0059237
i1_HQ_YHS_c27071/f3p0/1696	1041.534026	27.74717678	5.2302	0.00068206	0.0059248
i3_LQ_YHS_c18788/f1p4/3454	214.6333732	650.8654056	-1.6005	0.00068477	0.0059473
i5_LQ_YHS_c2629/f1p1/5239	393.3980363	44.8693405	3.1322	0.00068623	0.0059589
i3_LQ_YHS_c12999/f1p13/3313	7.846708005	81.41541637	-3.3751	0.00068666	0.0059616
i2_LQ_YHS_c51822/f1p0/3001	67.68819016	11.1831529	2.5976	0.00068734	0.0059656
i2_LQ_YHS_c6442/f1p0/2316	35.2517417	137.3705948	-1.9623	0.00068737	0.0059656
i2_LQ_YHS_c50539/f1p1/2226	211.9292362	631.910604	-1.5761	0.00068754	0.005966
i2_LQ_YHS_c40692/f1p0/2435	0	32.5672242	-Inf	0.00068949	0.0059797
i2_LQ_YHS_c49860/f1p1/2291	108.4366921	24.92678376	2.1211	0.00068942	0.0059797
i3_LQ_YHS_c10585/f1p1/3204	5.267653811	42.41285133	-3.0093	0.00068946	0.0059797
i4_LQ_YHS_c2625/f1p0/4942	378.3996657	118.8058914	1.6713	0.00068966	0.0059801
i2_LQ_YHS_c6345/f1p0/2230	122.2112956	7.516427992	4.0232	0.00069315	0.0060093
i3_HQ_YHS_c1742/f3p1/3266	259.3909497	80.6015911	1.6862	0.00069362	0.0060123
i2_LQ_YHS_c4375/f1p4/2401	6.07545079	69.52341254	-3.5164	0.00069377	0.0060125
i2_LQ_YHS_c43736/f1p20/2097	168.224828	6.244502185	4.7517	0.00069639	0.0060341
i1_LQ_YHS_c5439/f1p0/1823	272.8424603	84.0967885	1.6979	0.00069688	0.0060373
i3_LQ_YHS_c20531/f1p0/3055	836.5279849	77.55524821	3.4311	0.00069739	0.0060396
i4_LQ_YHS_c5663/f1p3/4941	6.96926754	50.63868235	-2.8612	0.00069736	0.0060396
i1_LQ_YHS_c29864/f1p2/1460	627.0370868	202.1976598	1.6328	0.00069913	0.0060535
i2_LQ_YHS_c33802/f1p7/2262	252.3248989	1197.635699	-2.2468	0.00069935	0.0060544
i5_LQ_YHS_c1479/f1p0/5233	0.719249673	29.24280111	-5.3454	0.00070017	0.0060603
i2_LQ_YHS_c33331/f1p0/2602	396.0296191	1217.789214	-1.6206	0.00070166	0.0060712
i5_LQ_YHS_c4422/f1p7/5752	2.50814694	32.21394475	-3.683	0.00070167	0.0060712
i3_LQ_YHS_c4077/f1p0/3266	1001.614878	294.7544825	1.7647	0.00070217	0.0060744
i1_LQ_YHS_c34459/f1p11/1667	217.7908714	18.80582101	3.5337	0.00070538	0.0061
i1_LQ_YHS_c42865/f1p3/1780	306.2777987	36378.78548	-6.8921	0.00070538	0.0061
i1_LQ_YHS_c23806/f1p0/1959	208.0782615	633.8761723	-1.6071	0.00070623	0.0061054
i2_LQ_YHS_c25012/f1p3/2172	68.58150668	267.1316763	-1.9617	0.00070626	0.0061054
i1_LQ_YHS_c19808/f1p2/1588	311.63818	36352.28083	-6.866	0.0007079	0.0061185
i2_LQ_YHS_c25595/f1p0/2215	76.13873357	14.08419388	2.4346	0.0007084	0.0061217
i3_HQ_YHS_c22456/f4p0/3268	1124.274972	15904.11753	-3.8223	0.00071003	0.0061347
i4_LQ_YHS_c12499/f1p3/4463	100.2012783	306.3979873	-1.6125	0.00071059	0.0061385
i1_LQ_YHS_c36733/f1p0/1954	66.29881967	2.539981251	4.7061	0.00071257	0.0061545
i1_HQ_YHS_c2819/f9p8/1626	264.4212192	833.4587737	-1.6563	0.0007134	0.0061605
i2_LQ_YHS_c56489/f1p4/2461	17.46259568	86.63128149	-2.3106	0.00071472	0.0061708
i2_LQ_YHS_c24959/f1p3/2412	21.35470262	100.3236874	-2.232	0.00071514	0.0061733
i0_LQ_YHS_c1235/f1p0/668	24.36938585	1.100005497	4.4695	0.00071544	0.0061748
i3_HQ_YHS_c8666/f2p0/3180	7.576902616	210.525153	-4.7962	0.00071896	0.0062041
i1_LQ_YHS_c10529/f1p0/1880	21.31654794	96.07011216	-2.1721	0.00072223	0.0062312
i1_HQ_YHS_c8246/f2p0/1613	361.0348371	9.838319864	5.1976	0.00072549	0.0062582
i2_LQ_YHS_c52657/f1p4/2834	62.10956526	204.1913123	-1.717	0.00072607	0.0062621
i2_LQ_YHS_c50654/f1p3/2325	771.6861758	172.4203338	2.1621	0.00072669	0.0062653
i4_LQ_YHS_c13785/f1p0/4120	107.1489122	6.539369161	4.0343	0.0007267	0.0062653

i2_LQ_YHS_c12443/f1p0/2220	68.91685908	0	Inf	0.00072723	0.0062677
i2_LQ_YHS_c25978/f1p1/2702	471.0379882	7.119703822	6.0479	0.00072724	0.0062677
i1_HQ_YHS_c7675/f2p1/1652	308.8762629	978.9018684	-1.6641	0.00072806	0.0062736
i3_LQ_YHS_c18755/f1p1/3328	131.899474	13.87834792	3.2485	0.00072926	0.0062829
i2_HQ_YHS_c27322/f2p0/2522	116.6919348	6.23838048	4.2254	0.00073055	0.0062929
i3_LQ_YHS_c12805/f1p0/3641	0.544682597	19.41004972	-5.1552	0.00073091	0.0062949
i1_LQ_YHS_c23165/f1p1/2004	3009.414334	753.5673866	1.9977	0.00073112	0.0062956
i3_LQ_YHS_c8945/f1p0/3298	170.675743	36.67964693	2.2182	0.00073265	0.0063076
i2_HQ_YHS_c61543/f3p3/2071	203.7289996	605.2086857	-1.5708	0.00073315	0.0063108
i2_LQ_YHS_c41187/f1p3/2519	791.2202769	263.436508	1.5866	0.00073362	0.0063137
i3_HQ_YHS_c10496/f2p4/3425	2267.033376	619.8178052	1.8709	0.0007341	0.0063167
i2_LQ_YHS_c29247/f1p2/2108	346.1958552	1092.50633	-1.658	0.0007354	0.0063268
i2_LQ_YHS_c3728/f1p1/2736	124.5973289	20.18457971	2.6259	0.00073772	0.0063456
i0_LQ_YHS_c2324/f1p0/519	1085.29584	43.73762114	4.6331	0.00073823	0.0063489
i2_HQ_YHS_c27965/f2p2/2106	3.802287496	36.76317856	-3.2733	0.00073837	0.0063489
i1_LQ_YHS_c28780/f1p0/1183	273.2422922	824.5150061	-1.5934	0.00073883	0.0063497
i2_LQ_YHS_c12933/f1p0/2961	296.9252013	89.22572464	1.7346	0.00073886	0.0063497
i2_LQ_YHS_c59463/f1p1/2053	21.02195313	127.409648	-2.5995	0.00073874	0.0063497
i2_LQ_YHS_c19471/f1p0/2327	0	19.68323445	-Inf	0.00073952	0.0063543
i1_LQ_YHS_c12163/f1p0/1960	0	13.54390659	-Inf	0.00074231	0.0063769
i3_LQ_YHS_c4737/f1p0/3573	62.45533297	211.7601101	-1.7615	0.00074241	0.0063769
i2_LQ_YHS_c23625/f1p74/2669	0	34.39744917	-Inf	0.00074274	0.0063785
i2_HQ_YHS_c3923/f3p1/2183	42.33549004	157.2507896	-1.8931	0.00074417	0.0063897
i1_LQ_YHS_c11107/f1p4/1428	57.64369477	193.1138869	-1.7442	0.00074479	0.0063925
i2_HQ_YHS_c9974/f2p3/2534	5.148889846	56.61624128	-3.4589	0.00074463	0.0063925
i2_LQ_YHS_c54783/f1p1/2404	480.2768612	74.86392356	2.6815	0.00074489	0.0063925
i3_LQ_YHS_c18701/f1p31/3626	3.782569895	147.7232148	-5.2874	0.00074544	0.006396
i1_LQ_YHS_c18105/f1p6/1870	3.81277818	38.69383564	-3.3432	0.00074572	0.0063973
i2_LQ_YHS_c3443/f1p0/2948	1563.533463	221.1720851	2.8216	0.00074622	0.0064005
i0_LQ_YHS_c3402/f1p0/353	0	13.51163949	-Inf	0.00074643	0.0064011
i3_LQ_YHS_c8671/f1p10/3482	0	13.50998093	-Inf	0.00074664	0.0064018
i2_LQ_YHS_c13679/f1p0/2848	33.305112	2.84934298	3.547	0.00074856	0.0064172
i1_LQ_YHS_c27582/f1p0/1192	248.5712902	77.00437607	1.6906	0.00075034	0.0064313
i2_LQ_YHS_c21534/f1p0/2794	0	45.20919099	-Inf	0.00075118	0.006435
i2_LQ_YHS_c40933/f1p6/2091	20.44451796	95.52859976	-2.2242	0.00075115	0.006435
i2_LQ_YHS_c7572/f1p2/2941	11.39348885	69.18139092	-2.6022	0.00075109	0.006435
i2_LQ_YHS_c3196/f1p0/2093	62.86746148	0	Inf	0.00075136	0.0064354
i1_LQ_YHS_c33948/f1p5/1423	15.23521155	78.5913123	-2.367	0.00075179	0.006438
i4_LQ_YHS_c13567/f1p0/4117	361.7818907	1149.147715	-1.6674	0.00075235	0.0064417
i2_LQ_YHS_c43381/f1p0/2079	190.0052399	4.533553501	5.3893	0.00075252	0.0064419
i4_LQ_YHS_c8440/f1p0/4179	432.4146862	146.6358082	1.5602	0.00075369	0.0064508
i2_LQ_YHS_c13151/f1p4/2733	218.4014134	639.6425168	-1.5503	0.00075462	0.0064553
i2_LQ_YHS_c36890/f1p4/2424	25.57415694	1.439975754	4.1506	0.00075451	0.0064553
i2_LQ_YHS_c54534/f1p1/2552	27.09659851	114.4168866	-2.0781	0.00075458	0.0064553
i1_LQ_YHS_c14358/f1p161/1325	210.2644105	615.2239005	-1.5489	0.00075489	0.0064554
i1_LQ_YHS_c35796/f1p0/1883	137.8118476	417.1352326	-1.5978	0.00075489	0.0064554
i2_LQ_YHS_c18090/f4p2/2145	177.2486803	539.8951295	-1.6069	0.00075677	0.0064703
i1_LQ_YHS_c7565/f1p7/1761	96.31490377	326.0069361	-1.7591	0.00075774	0.0064775
i1_LQ_YHS_c11913/f4p0/1897	535.4475335	45.52206983	3.5561	0.00075792	0.0064778
i2_HQ_YHS_c37173/f2p39/2269	771.1334678	256.3515237	1.5889	0.00075896	0.0064856
i2_LQ_YHS_c34426/f1p0/2355	0	13.40871651	-Inf	0.00075975	0.00649
i5_LQ_YHS_c790/f1p0/5371	0	13.40871651	-Inf	0.00075975	0.00649
i1_LQ_YHS_c24847/f1p1/1444	2427.505775	663.4169214	1.8715	0.00076	0.006491
i2_HQ_YHS_c12201/f2p2/2382	1047.407419	332.3769093	1.6559	0.00076067	0.0064956
i2_LQ_YHS_c26957/f1p3/2757	41.38797285	0	Inf	0.00076156	0.0065021
i2_LQ_YHS_c40641/f1p5/2213	330.110562	1019.401655	-1.6267	0.00076279	0.0065114
i2_LQ_YHS_c18039/f1p0/2232	0	13.38257112	-Inf	0.00076319	0.0065136
i2_LQ_YHS_c20965/f2p3/2240	160.8629976	42.91368403	1.9063	0.00076382	0.0065179
i1_HQ_YHS_c17020/f4p0/1793	101.7494344	314.7105473	-1.629	0.00076534	0.0065298
i1_HQ_YHS_c17145/f2p0/1882	75.06399741	237.0105504	-1.6588	0.00076765	0.0065483
i4_LQ_YHS_c2794/f1p0/4609	78.45766765	249.8918169	-1.6713	0.00076941	0.0065621
i4_LQ_YHS_c13652/f1p0/4128	179.7191644	51.47889254	1.8037	0.00077044	0.0065698
i4_LQ_YHS_c6749/f1p1/4286	1108.231174	117.0787092	3.2427	0.00077095	0.0065729
i2_HQ_YHS_c24531/f2p2/2222	104.5029862	0.784522063	7.0575	0.00077117	0.0065736
i1_LQ_YHS_c3899/f1p0/1678	5655.554135	1089.214836	2.3764	0.00077396	0.0065963
i1_LQ_YHS_c10667/f1p2/1595	531.7969349	90.75085104	2.5509	0.00077495	0.0066035
i2_LQ_YHS_c52510/f1p3/2833	141.8565733	438.41429	-1.6279	0.00077654	0.0066159
i0_LQ_YHS_c2354/f1p1/585	485.4849827	160.8322837	1.5939	0.00077714	0.0066199
i2_LQ_YHS_c33638/f1p0/3002	265.5561003	84.52352842	1.6516	0.00077851	0.0066304
i2_LQ_YHS_c46351/f2p1/2569	70.22318733	232.4734402	-1.727	0.00077873	0.0066311
i4_LQ_YHS_c2937/f1p0/4519	0	16.89735986	-Inf	0.00077995	0.0066403
i3_LQ_YHS_c10707/f1p8/3210	0	13.25516132	-Inf	0.00078022	0.0066414
i1_LQ_YHS_c24133/f1p9/1752	0.616861993	82.07012289	-7.0558	0.00078092	0.0066462
i2_LQ_YHS_c5208/f1p3/2369	26.98751982	156.3509814	-2.5344	0.00078238	0.0066575
i3_LQ_YHS_c3472/f1p0/3320	218.5612772	66.09500067	1.7254	0.00078344	0.0066653
i3_LQ_YHS_c19750/f1p1/3257	721.6694781	8.282584939	6.4451	0.00078383	0.0066674
i3_LQ_YHS_c9098/f1p0/3201	13.1660182	72.19929641	-2.4552	0.00078403	0.006668
i4_LQ_YHS_c2806/f1p0/4695	147.5354593	34.25874198	2.1065	0.00078462	0.0066719
i2_LQ_YHS_c41186/f1p1/2888	174.3308294	870.785682	-2.3205	0.00078515	0.0066752
i2_HQ_YHS_c27769/f3p0/2037	474.0718373	159.2578315	1.5737	0.00078614	0.0066824
i2_LQ_YHS_c42166/f1p2/2555	277.3143695	11.0618648	4.6479	0.00078637	0.0066831
i3_LQ_YHS_c3449/f1p9/3342	3.344888437	39.32989797	-3.5556	0.00078726	0.0066896
i3_LQ_YHS_c13915/f1p0/3025	260.1092468	3.395159205	6.2595	0.00078828	0.0066971
i2_LQ_YHS_c29383/f1p1/2074	171.7287666	48.70020545	1.8181	0.00078843	0.0066972
i1_LQ_YHS_c41623/f1p2/1363	2075.081779	146.6250294	3.823	0.00078892	0.0066992
i2_LQ_YHS_c24422/f1p64/1929	2318.879806	0	Inf	0.00078895	0.0066992
i2_LQ_YHS_c35353/f1p1/3007	0	13.18896857	-Inf	0.00078927	0.0067007
i2_LQ_YHS_c54939/f1p0/2579	29.04322821	116.9267323	-2.0093	0.00078995	0.0067054
i4_LQ_YHS_c11954/f1p0/4655	82.61128654	16.52231794	2.3219	0.00079017	0.006706

i1_HQ_YHS_c40210/f4p0/1890	374.7819129	121.6606831	1.6232	0.00079059	0.0067084
i2_HQ_YHS_c32600/f2p3/2526	188.9859765	4.392241718	5.4272	0.00079208	0.0067199
i0_LQ_YHS_c2920/f1p0/555	2507.697435	634.1122291	1.9836	0.00079531	0.0067461
i2_LQ_YHS_c40544/f1p0/2197	185.2067061	52.24165214	1.8259	0.00079694	0.0067587
i4_LQ_YHS_c10125/f1p0/4895	188.279051	7.356751094	4.6777	0.00079724	0.0067601
i3_LQ_YHS_c14663/f1p0/3038	136.7301069	422.0377014	-1.626	0.00079933	0.0067762
i4_LQ_YHS_c3409/f1p0/5054	24.51627217	107.0478526	-2.1264	0.00079942	0.0067762
i1_LQ_YHS_c36136/f1p2/1787	8.871043172	57.82576448	-2.7045	0.00080051	0.0067842
i4_LQ_YHS_c10961/f1p3/4594	258.0788963	82.44314696	1.6463	0.00080139	0.0067906
i1_LQ_YHS_c13978/f1p0/1186	34.1350428	130.3972982	-1.9336	0.00080366	0.0068085
i2_LQ_YHS_c3856/f1p0/2935	122.3971504	22.91484639	2.4172	0.00080533	0.0068215
i2_LQ_YHS_c33928/f1p0/2212	10.26423009	62.39320409	-2.6038	0.0008059	0.0068252
i3_LQ_YHS_c3627/f1p0/3877	6.505982881	49.98157009	-2.9416	0.00080682	0.0068318
i2_HQ_YHS_c48197/f4p4/2894	91.7064505	298.5739363	-1.703	0.00080746	0.0068359
i4_LQ_YHS_c9975/f1p0/4774	146.4295275	442.2860692	-1.5948	0.00080763	0.0068362
i5_HQ_YHS_c5124/f3p1/5129	996.3311651	329.614887	1.5958	0.00080778	0.0068363
i2_LQ_YHS_c7277/f1p1/2307	21.67617277	112.1013164	-2.3706	0.00080862	0.0068422
i5_LQ_YHS_c1609/f1p1/5217	22.02104051	90.38872552	-2.0373	0.0008099	0.0068518
i4_LQ_YHS_c7913/f1p0/4391	89.73285927	19.33220633	2.2146	0.00081091	0.0068591
i3_LQ_YHS_c3932/f1p0/3343	4.532027853	50.98035072	-3.4917	0.00081321	0.0068774
i4_LQ_YHS_c12030/f1p2/4989	40.40203175	0	Inf	0.00081514	0.0068926
i2_LQ_YHS_c20874/f1p0/2450	107.9577456	2.74582722	5.2971	0.00081578	0.0068967
i2_LQ_YHS_c48599/f1p1/2274	12.25535844	70.88949549	-2.5322	0.00081657	0.0069022
i0_LQ_YHS_c3066/f1p0/475	55.15492675	354.9043149	-2.6859	0.00081763	0.006908
i1_LQ_YHS_c32298/f1p1/1507	201.85678	59.04263561	1.7735	0.00081768	0.006908
i2_LQ_YHS_c39608/f1p0/2672	133.2691352	20.18572572	2.7229	0.00081739	0.006908
i3_LQ_YHS_c3499/f1p1/3843	42.29063598	159.6004854	-1.9161	0.00081926	0.0069201
i2_LQ_YHS_c53744/f1p2/2756	11.71067584	70.93396532	-2.5987	0.00082093	0.006933
i2_LQ_YHS_c35947/f1p16/2580	694.9125573	2764.063412	-1.9919	0.00082197	0.0069405
i3_LQ_YHS_c20632/f1p0/3045	256.4465802	3.465815096	6.2093	0.00082414	0.0069577
i5_LQ_YHS_c4658/f1p0/5159	472.014493	1546.295061	-1.7119	0.00082553	0.0069682
i2_LQ_YHS_c53039/f1p0/2863	19.19870085	160.1292803	-3.0602	0.00082634	0.0069738
i0_HQ_YHS_c357/f2p0/676	16354.64077	95.38673587	7.4217	0.00082713	0.0069793
i1_HQ_YHS_c40305/f16p0/1465	1297.838105	9841.34211	-2.9227	0.00082774	0.0069832
i3_LQ_YHS_c9857/f1p5/3158	258.1052223	82.85653701	1.6393	0.00082858	0.006989
i1_LQ_YHS_c27551/f1p0/1315	1026.970911	332.8828386	1.6253	0.00082935	0.0069934
i3_HQ_YHS_c10247/f2p0/3658	532.8729671	179.5171363	1.5697	0.00082939	0.0069934
i1_LQ_YHS_c12725/f1p0/1813	140.562933	422.6267277	-1.5882	0.00082979	0.0069956
i4_LQ_YHS_c13386/f1p7/4049	853.6598787	286.2785691	1.5762	0.00083107	0.0070052
i1_LQ_YHS_c5153/f1p2/1938	138.9809724	0	Inf	0.00083296	0.007018
i2_LQ_YHS_c60069/f14p6/2304	502.6939651	1723.619534	-1.7777	0.00083294	0.007018
i3_LQ_YHS_c17608/f1p1/3606	59.36182959	5.170642076	3.5211	0.00083302	0.007018
i2_HQ_YHS_c40470/f2p23/2101	47.6856117	1.061616699	5.4892	0.00083334	0.0070194
i0_LQ_YHS_c539/f1p0/967	883.939661	287.1250208	1.6223	0.00083674	0.0070444
i1_LQ_YHS_c27450/f1p0/1299	631.8742074	2383.861493	-1.9156	0.00083672	0.0070444
i1_LQ_YHS_c5203/f1p0/1851	53.89686333	188.1317113	-1.8035	0.00083674	0.0070444
i3_LQ_YHS_c3906/f1p0/3192	72.14596945	13.29967182	2.4395	0.00083698	0.0070451
i3_LQ_YHS_c11958/f1p3/3356	194.1159291	38.78676656	2.3233	0.00083937	0.0070641
i1_LQ_YHS_c35319/f1p1/1490	124.5032263	31.10568597	2.0009	0.00083959	0.0070646
i2_LQ_YHS_c13806/f1p0/2202	0	15.8896925	-Inf	0.00084059	0.0070718
i3_LQ_YHS_c8025/f1p1/3718	1.618943649	42.17260669	-4.7032	0.00084077	0.0070722
i2_HQ_YHS_c32277/f5p4/2638	94.03024215	294.697016	-1.648	0.0008417	0.0070775
i2_LQ_YHS_c38568/f1p1/2846	277.1630469	81.34373424	1.7686	0.00084161	0.0070775
i3_HQ_YHS_c2542/f2p0/3497	1658.193944	23.84960651	6.1195	0.0008445	0.0070986
i3_LQ_YHS_c11027/f1p0/3558	3841.684422	933.3035929	2.0413	0.00084439	0.0070986
i1_HQ_YHS_c11336/f2p0/1880	229.4787145	17.74313853	3.693	0.00084484	0.0070997
i1_LQ_YHS_c39811/f21p1/1987	286.6563362	1044.663047	-1.8656	0.00084493	0.0070997
i2_HQ_YHS_c9750/f3p0/2554	1279.763509	402.6373816	1.6683	0.00084521	0.0070998
i2_LQ_YHS_c59766/f1p2/2077	214.8729871	64.4036422	1.7383	0.00084524	0.0070998
i2_LQ_YHS_c15149/f1p1/2087	8.939881251	57.50084221	-2.6853	0.00084624	0.007107
i7_LQ_YHS_c79/f1p0/8003	10.79127264	123.2423228	-3.5136	0.00084743	0.0071157
i2_LQ_YHS_c55432/f1p4/2888	284.8351376	93.00192771	1.6148	0.00084856	0.007124
i1_LQ_YHS_c13817/f1p0/1394	63.88161951	208.2306278	-1.7047	0.00085087	0.0071409
i2_HQ_YHS_c9462/f2p4/2766	213.6487629	48.92939223	2.1265	0.00085087	0.0071409
i4_LQ_YHS_c10271/f1p0/4560	55.00225777	194.3573347	-1.8211	0.00085105	0.0071412
i4_LQ_YHS_c11470/f1p0/5044	66.57830196	11.3116285	2.5572	0.00085298	0.0071561
i3_LQ_YHS_c6831/f1p2/3484	16.25160031	78.98187409	-2.2809	0.0008536	0.0071601
i2_LQ_YHS_c52085/f1p3/2061	224.2507688	828.1477549	-1.8848	0.00085764	0.0071928
i3_LQ_YHS_c4340/f1p3/3324	0	18.88200583	-Inf	0.00085833	0.0071973
i4_LQ_YHS_c9432/f1p0/4524	245.037883	77.15674571	1.6671	0.00086008	0.0072107
i3_LQ_YHS_c8105/f1p0/3338	126.778442	380.4858014	-1.5855	0.00086036	0.0072118
i3_LQ_YHS_c9879/f1p1/3831	188.7056458	568.0350464	-1.5898	0.00086166	0.0072215
i2_LQ_YHS_c13624/f1p143/2219	3297.175958	0.359993938	13.161	0.00086216	0.0072244
i2_HQ_YHS_c30432/f3p1/2564	251.488563	76.36839283	1.7194	0.00086489	0.007246
i3_LQ_YHS_c22209/f1p2/3301	200.2110891	59.53833218	1.7496	0.00086506	0.0072462
i1_LQ_YHS_c29578/f15p0/1853	430.1481984	1356.571764	-1.6571	0.00086688	0.0072601
i5_LQ_YHS_c3404/f1p0/5621	142.6685099	9.023189275	3.9829	0.00086751	0.0072642
i4_LQ_YHS_c3423/f1p1/4340	0	18.54649872	-Inf	0.00086771	0.0072646
i2_HQ_YHS_c61738/f40p4/2643	6880.575183	83.18114558	6.3701	0.00086787	0.0072647
i1_LQ_YHS_c13543/f1p0/1233	159.9160166	46.06670051	1.7955	0.00086804	0.0072649
i2_HQ_YHS_c60953/f12p7/2229	205.474145	617.0325663	-1.5864	0.0008685	0.0072674
i1_HQ_YHS_c15190/f4p2/1408	5475.27468	519.1702218	3.3987	0.00086938	0.0072736
i2_LQ_YHS_c26491/f1p0/2171	37.39598902	137.1971752	-1.8753	0.0008699	0.0072766
i1_LQ_YHS_c28001/f1p6/1315	344.6326524	1061.605897	-1.6231	0.00087011	0.0072771
i2_LQ_YHS_c49769/f1p1/2425	4.511046485	37.34240788	-3.0493	0.00087203	0.0072919
i1_LQ_YHS_c11783/f1p0/1875	248.3038437	35.45448297	2.8081	0.00087378	0.0073052
i1_LQ_YHS_c26080/f1p0/1929	9.790454748	60.90986384	-2.6372	0.00087552	0.0073186
i5_LQ_YHS_c4027/f1p1/5161	1.16154459	28.35752719	-4.6096	0.00087637	0.0073244
i4_LQ_YHS_c11519/f1p0/4226	10.11780213	60.41858973	-2.5781	0.00087948	0.0073491

i1_HQ_YHS_c4346/f2p1/1862	383.7990705	126.1785958	1.6049	0.00088156	0.0073652
i1_LQ_YHS_c18665/f1p2/1530	214.5730069	65.08567475	1.7211	0.00088405	0.0073848
i4_LQ_YHS_c11766/f1p0/4971	0	13.09935478	-Inf	0.00088507	0.0073913
i4_LQ_YHS_c13850/f1p0/4096	236.0758931	72.7167159	1.6989	0.00088513	0.0073913
i2_LQ_YHS_c42044/f1p0/2685	234.97061	72.05798463	1.7053	0.00088679	0.0074038
i2_LQ_YHS_c52196/f1p0/2473	75.51103385	15.1836066	2.3142	0.0008871	0.0074051
i2_LQ_YHS_c18837/f1p2/3003	207.0303384	823.5406031	-1.992	0.00088732	0.0074057
i5_LQ_YHS_c4661/f1p73/5052	0	13.08098967	-Inf	0.00088788	0.0074091
i2_LQ_YHS_c21451/f1p1/2756	25.2463512	175.877504	-2.8004	0.00088905	0.0074176
i3_LQ_YHS_c7203/f1p4/3438	518.8537486	151.1476783	1.7794	0.00088979	0.0074224
i2_LQ_YHS_c64647/f1p0/2009	110.7583727	500.2277484	-2.1752	0.00089027	0.0074251
i2_LQ_YHS_c23901/f1p1/2068	98.23299826	17.31296171	2.5044	0.00089145	0.0074337
i1_LQ_YHS_c26008/f1p0/2115	4274.712773	0.353872233	13.56	0.00089241	0.0074397
i3_LQ_YHS_c14030/f1p0/3047	37.84718893	0.707744466	5.7408	0.00089247	0.0074397
i1_LQ_YHS_c22182/f1p0/1785	116.6055057	11.94705851	3.2869	0.00089304	0.0074418
i2_LQ_YHS_c55543/f1p13/2157	3568.893309	65.22418196	5.7739	0.000893	0.0074418
i3_LQ_YHS_c5225/f1p0/3140	1.07887451	43.7514429	-5.3417	0.00089402	0.0074488
i2_LQ_YHS_c24102/f1p0/2414	15.75383258	77.36164565	-2.2959	0.00089438	0.0074504
i1_LQ_YHS_c36155/f1p0/1776	87.93059674	13.34252376	2.7203	0.00089538	0.0074575
i1_HQ_YHS_c2860/f2p8/1500	570.8816641	190.4601979	1.5837	0.00089841	0.0074814
i4_LQ_YHS_c6602/f1p4/4925	71.12970875	3.904837974	4.1871	0.00089877	0.0074831
i1_LQ_YHS_c14247/f1p0/1267	141.5322956	408.9321891	-1.5307	0.0009008	0.0074987
i2_HQ_YHS_c5281/f2p4/2744	533.5457148	1788.503456	-1.7451	0.00090126	0.0075013
i2_LQ_YHS_c48887/f1p1/2148	237.993698	74.17671534	1.6819	0.00090378	0.007521
i2_LQ_YHS_c7845/f1p0/2307	105.6934251	24.53231093	2.1071	0.00090907	0.0075637
i2_LQ_YHS_c21597/f1p0/2574	0	12.93801932	-Inf	0.00091018	0.0075716
i4_LQ_YHS_c7136/f1p1/4290	84.90325071	14.50872201	2.5489	0.00091169	0.0075829
i2_LQ_YHS_c54512/f1p0/2758	43.7291437	5.569024812	2.9731	0.00091241	0.0075876
i1_LQ_YHS_c20343/f1p0/1446	34.42249662	133.2313944	-1.9525	0.00091325	0.0075933
i1_LQ_YHS_c23119/f1p0/1828	139.7573668	402.571535	-1.5263	0.00091474	0.0076043
i3_LQ_YHS_c18093/f1p3/3435	5.591184763	44.65571426	-2.9976	0.00091518	0.0076066
i2_LQ_YHS_c3342/f1p2/2401	42.09972611	143.9268298	-1.7735	0.00091609	0.0076116
i3_LQ_YHS_c17862/f1p0/3241	253.6426823	5.201250602	5.6078	0.00091596	0.0076116
i2_LQ_YHS_c49991/f1p3/2392	0	33.16110759	-Inf	0.00091674	0.0076157
i4_HQ_YHS_c1720/f6p0/4914	0	40.97275581	-Inf	0.0009188	0.0076315
i0_LQ_YHS_c177/f1p0/760	0	12.8812654	-Inf	0.00091923	0.0076338
i3_LQ_YHS_c14295/f1p0/3054	52.53293448	220.1599942	-2.0673	0.0009194	0.0076339
i2_LQ_YHS_c13752/f1p0/2099	152.8796331	43.29310888	1.8202	0.00092084	0.0076445
i4_LQ_YHS_c8718/f1p0/4514	0	12.86902199	-Inf	0.0009212	0.0076462
i2_LQ_YHS_c56151/f1p2/2096	849.3084818	275.175317	1.6259	0.00092265	0.0076569
i4_LQ_YHS_c7394/f1p2/4385	0	25.37686449	-Inf	0.00092359	0.0076634
i1_LQ_YHS_c33669/f1p0/1547	98.05113701	299.7461365	-1.6121	0.0009269	0.0076895
i3_LQ_YHS_c5579/f1p3/3143	128.9952864	1.067738404	6.9166	0.00092708	0.0076897
i2_LQ_YHS_c38078/f1p0/2429	61.05329968	190.7851597	-1.6438	0.00092789	0.0076951
i2_LQ_YHS_c54683/f1p2/2554	371.3426766	1182.443339	-1.6709	0.00092809	0.0076954
i1_HQ_YHS_c4157/f2p0/1555	46.25954415	163.7947406	-1.8241	0.00092848	0.007696
i7_LQ_YHS_c177/f1p0/7252	6.787551096	56.78239313	-3.0645	0.00092842	0.007696
i0_LQ_YHS_c652/f1p0/919	1137.622086	17243.74672	-3.922	0.00093509	0.0077495
i1_LQ_YHS_c18725/f1p3/1989	110.9638625	336.7711211	-1.6017	0.00093579	0.0077527
i4_LQ_YHS_c3775/f1p0/4175	31.91146672	122.9170879	-1.9455	0.00093579	0.0077527
i2_LQ_YHS_c38275/f1p2/2268	1.438499347	22.59770439	-3.9735	0.00093783	0.0077682
i1_LQ_YHS_c23985/f1p27/1735	79.40601537	247.6968212	-1.6413	0.00094271	0.0078073
i1_LQ_YHS_c25216/f1p0/1787	107.9068863	16.02713392	2.7512	0.00094295	0.0078079
i1_LQ_YHS_c25878/f1p2/1779	115.5152777	12.07671966	3.2578	0.00094333	0.0078097
i2_HQ_YHS_c33927/f2p0/2750	219.2747933	657.8434312	-1.585	0.00094499	0.0078221
i1_LQ_YHS_c31718/f1p0/1690	98.41887818	3.999980687	4.6209	0.00094625	0.0078301
i2_LQ_YHS_c2475/f4p0/2196	120.416496	30.66998022	1.9731	0.00094627	0.0078301
i3_LQ_YHS_c13520/f1p2/3279	241.1920459	830.2276599	-1.7833	0.00094763	0.0078387
i3_LQ_YHS_c9565/f1p0/3258	256.0295449	81.31912765	1.6546	0.00094764	0.0078387
i4_LQ_YHS_c9418/f1p15/4363	159.9607007	480.6344045	-1.5872	0.00094935	0.0078515
i1_HQ_YHS_c10984/f2p3/1670	18.82811022	196.2746012	-3.3819	0.00094977	0.0078537
i1_HQ_YHS_c17073/f2p0/2010	176.2013436	521.5660544	-1.5656	0.0009525	0.0078749
i3_LQ_YHS_c4651/f1p0/3515	9.396008168	59.91613801	-2.6728	0.00095306	0.0078782
i1_LQ_YHS_c10142/f1p0/1963	3.442662659	55.36706351	-4.0074	0.00095415	0.0078858
i1_LQ_YHS_c6181/f1p0/1846	71.26458449	220.9284013	-1.6323	0.00095501	0.0078916
i3_LQ_YHS_c19536/f1p1/3451	3.355379121	34.83295494	-3.3759	0.00095843	0.0079185
i1_LQ_YHS_c5737/f1p0/1849	23.51291838	181.4040339	-2.9477	0.00095915	0.0079231
i2_LQ_YHS_c4519/f1p5/2763	467.1759831	160.5645142	1.5408	0.00095945	0.0079242
i4_LQ_YHS_c3604/f1p5/4350	4.584489648	40.90999995	-3.1576	0.00096193	0.0079433
i4_LQ_YHS_c11624/f1p3/4360	39.01538265	330.8566328	-3.0841	0.000963	0.0079508
i1_HQ_YHS_c15154/f3p3/1696	331.1552574	4.339950943	6.2537	0.00096473	0.0079633
i2_HQ_YHS_c60276/f4p3/2104	109.8371313	334.6209183	-1.6072	0.00096485	0.0079633
i1_LQ_YHS_c23112/f1p0/1709	3298.968513	817.0843072	2.0135	0.0009665	0.0079756
i2_LQ_YHS_c58211/f1p5/1968	1161.448423	101.5027613	3.5163	0.00096911	0.0079958
i2_LQ_YHS_c41717/f1p1/2463	0	33.43910871	-Inf	0.00097188	0.0080172
i5_LQ_YHS_c4697/f1p3/5090	152.3976469	450.3078721	-1.5631	0.00097451	0.0080376
i2_LQ_YHS_c13273/f1p0/2820	33.42801431	131.9083633	-1.9804	0.00097512	0.0080412
i2_LQ_YHS_c24934/f1p0/2664	173.7508643	2.9122186	5.8988	0.00097625	0.0080451
i3_HQ_YHS_c15524/f2p0/3303	231.6823588	12.61029247	4.1995	0.00097607	0.0080451
i5_LQ_YHS_c3346/f1p0/6014	83.42323024	258.0811166	-1.6293	0.00097581	0.0080451
i5_LQ_YHS_c4217/f1p0/5161	344.6994058	116.9343533	1.5596	0.00097613	0.0080451
i1_LQ_YHS_c22231/f1p0/1747	410.8916001	1281.749259	-1.6413	0.00097654	0.0080461
i2_LQ_YHS_c6078/f1p5/2388	1126.205476	228.4356251	2.3016	0.00097798	0.0080566
i4_LQ_YHS_c8775/f1p0/5018	2.133417961	28.98908683	-3.7643	0.00097987	0.0080708
i2_HQ_YHS_c57889/f2p4/1967	44.12739833	156.3516928	-1.825	0.00098194	0.0080865
i2_LQ_YHS_c9457/f1p4/2307	43.66618284	157.7633125	-1.8532	0.00098402	0.0081022
i1_LQ_YHS_c22352/f1p0/1969	1.089365194	22.44580776	-4.3649	0.00098612	0.0081181
i4_LQ_YHS_c9480/f1p1/4472	55.09371315	8.13906136	2.759	0.00098786	0.008131
i3_LQ_YHS_c17417/f1p0/3965	0.801919753	20.34931253	-4.6654	0.00098916	0.0081404

i3_LQ_YHS_c3342/f1p2/3368	27.98994852	112.4688907	-2.0065	0.00099115	0.0081554
i1_LQ_YHS_c20961/f1p1/1810	58.61650161	204.4223911	-1.8022	0.00099236	0.0081639
i1_LQ_YHS_c5742/f5p0/1616	949.163291	309.3577544	1.6174	0.0009931	0.0081686
i2_LQ_YHS_c26291/f1p0/2118	284.6891273	31.43779573	3.1788	0.00099336	0.0081693
i3_LQ_YHS_c8743/f1p0/3949	4.629802076	74.7447275	-4.0129	0.00099441	0.0081766
i3_HQ_YHS_c20282/f2p0/3111	210.120224	633.2993175	-1.5917	0.0009949	0.0081792
i1_LQ_YHS_c18691/f1p0/1767	14.28559168	73.62315839	-2.3656	0.0009953	0.0081812
i2_LQ_YHS_c7280/f1p0/2411	0	31.66710228	-Inf	0.00099618	0.008187
i3_LQ_YHS_c18842/f1p611/3258	56.57943254	273.1731729	-2.2715	0.0010018	0.0082318
i1_LQ_YHS_c24393/f1p456/1842	1115.353686	0	Inf	0.0010068	0.00827
i2_LQ_YHS_c26742/f1p0/2273	0	17.79258518	-Inf	0.0010068	0.00827
i3_LQ_YHS_c11616/f1p2/3274	182.0088237	33.83208228	2.4275	0.0010083	0.0082813
i1_LQ_YHS_c34840/f1p0/1690	253.6426405	43.19184446	2.554	0.0010104	0.0082968
i3_LQ_YHS_c4772/f1p43/3264	51.86885245	7.793522629	2.7345	0.0010107	0.008298
i2_LQ_YHS_c50453/f1p0/2475	45.96462742	3.433548003	3.7428	0.0010131	0.0083161
i2_LQ_YHS_c53396/f1p2/2691	116.6221549	344.8615297	-1.5642	0.001014	0.0083218
i2_LQ_YHS_c50231/f1p0/2235	29.40203926	1.852260467	3.9886	0.0010176	0.0083502
i2_HQ_YHS_c23939/f2p1/2914	33.78302569	130.9504217	-1.9547	0.0010192	0.008362
i2_LQ_YHS_c50517/f1p0/2861	13.82485131	0	Inf	0.0010219	0.0083824
i4_LQ_YHS_c8581/f1p6/4199	18.40345536	111.8303028	-2.6033	0.0010226	0.0083867
i3_LQ_YHS_c18432/f1p0/3293	49.68421971	173.9408834	-1.8077	0.0010247	0.0084026
i4_LQ_YHS_c13033/f1p4/4209	68.03476329	236.4784384	-1.7974	0.0010251	0.0084048
i1_LQ_YHS_c10677/f1p0/1804	0	12.82226014	-Inf	0.0010265	0.0084136
i2_HQ_YHS_c36278/f4p0/2516	240.2609422	903.1822412	-1.9104	0.0010265	0.0084136
i0_LQ_YHS_c1460/f1p0/984	108.2417707	327.79991	-1.5986	0.0010268	0.0084137
i2_HQ_YHS_c57704/f2p4/2029	247.965394	78.99241939	1.6504	0.0010271	0.0084137
i4_LQ_YHS_c9347/f1p0/4874	431.0100236	73.80443844	2.5459	0.001027	0.0084137
i1_HQ_YHS_c10172/f2p3/1610	193.4652195	559.4360666	-1.5319	0.0010276	0.0084149
i3_LQ_YHS_c7758/f1p5/3216	11.32212323	61.44454199	-2.4401	0.0010274	0.0084149
i3_LQ_YHS_c5631/f1p3/3185	263.9335963	64.14704426	2.0407	0.0010285	0.0084209
i1_LQ_YHS_c33658/f1p0/1845	7.461488342	49.28219868	-2.7235	0.0010289	0.0084232
i2_LQ_YHS_c22390/f1p2/3097	465.017978	163.4675274	1.5083	0.0010293	0.0084247
i2_LQ_YHS_c55464/f1p13/2666	4470.028075	86.09826078	5.6982	0.001032	0.0084451
i2_LQ_YHS_c19522/f1p0/2377	377.8300716	70.76208568	2.4167	0.0010331	0.0084518
i4_LQ_YHS_c5908/f1p0/4623	0	12.78552991	-Inf	0.0010331	0.0084518
i2_LQ_YHS_c24303/f1p0/2630	58.02986465	9.162842491	2.6629	0.0010346	0.0084621
i1_HQ_YHS_c2976/f3p0/1690	103.5996039	1.112248908	6.5414	0.0010358	0.0084684
i2_LQ_YHS_c11794/f1p1/2869	61.11844102	10.36130783	2.5604	0.0010359	0.0084684
i2_LQ_YHS_c54306/f1p0/2901	0	12.77162794	-Inf	0.0010356	0.0084684
i3_LQ_YHS_c18239/f1p0/3494	12.28683886	67.66846726	-2.4614	0.0010373	0.0084787
i2_LQ_YHS_c7538/f1p1/2639	63.25090039	209.3936682	-1.7271	0.0010387	0.008489
i0_LQ_YHS_c2671/f1p2/845	1733.366775	521.0634373	1.734	0.0010392	0.0084905
i5_LQ_YHS_c1872/f1p0/5319	8.227322585	50.85452039	-2.6279	0.0010393	0.0084905
i2_LQ_YHS_c64736/f1p0/2018	0	12.74714111	-Inf	0.00104	0.0084947
i2_LQ_YHS_c12477/f1p4/2106	162.2511989	474.1769491	-1.5472	0.0010435	0.0085224
i1_LQ_YHS_c11216/f1p2/1889	1592.9334	156.4118045	3.3483	0.0010449	0.0085323
i2_LQ_YHS_c21538/f1p18/2383	325.9113972	2429.071319	-2.8979	0.0010465	0.0085437
i1_LQ_YHS_c9867/f1p1/1996	0	12.70875232	-Inf	0.0010469	0.0085456
i2_LQ_YHS_c18023/f1p12/2215	10.15008796	55.52788642	-2.4517	0.001049	0.0085617
i1_LQ_YHS_c34287/f1p0/1548	57.00804864	189.1821503	-1.7305	0.0010508	0.0085743
i2_LQ_YHS_c53053/f1p0/2874	34.85809215	132.9979038	-1.9318	0.0010512	0.0085761
i2_HQ_YHS_c19503/f2p2/2272	43.96841888	1.569044125	4.8085	0.0010523	0.0085812
i2_HQ_YHS_c29300/f2p7/2140	719.4931366	221.8577139	1.6973	0.0010522	0.0085812
i2_HQ_YHS_c3608/f5p4/2745	442.1608494	153.7561443	1.5239	0.0010523	0.0085812
i3_LQ_YHS_c11883/f1p2/3682	237.6737477	37.92321555	2.6478	0.0010531	0.0085861
i2_HQ_YHS_c61284/f12p0/2591	9674.520406	60.97119953	7.3099	0.0010534	0.008587
i3_LQ_YHS_c4942/f1p0/3958	106.4531212	329.9660755	-1.6321	0.0010578	0.0086213
i4_LQ_YHS_c8075/f1p0/4774	0	12.63643786	-Inf	0.0010601	0.0086392
i1_LQ_YHS_c38634/f1p0/1226	0	12.63031615	-Inf	0.0010613	0.0086469
i4_LQ_YHS_c10992/f1p1/4774	46.50308574	0.392261031	6.8894	0.0010629	0.0086584
i4_LQ_YHS_c13199/f1p9/4146	0	21.23391346	-Inf	0.001063	0.0086584
i1_LQ_YHS_c23826/f1p0/1656	497.304754	1539.516811	-1.6303	0.0010648	0.0086709
i1_LQ_YHS_c14640/f1p0/1316	959.6015389	284.693531	1.753	0.0010666	0.0086848
i2_HQ_YHS_c48462/f2p4/2362	177.2205579	513.332242	-1.5343	0.0010672	0.0086862
i2_LQ_YHS_c46386/f2p7/2341	150.0730582	558.9684207	-1.8971	0.001067	0.0086862
i2_LQ_YHS_c8651/f1p4/2982	56.0676557	185.7296851	-1.728	0.001068	0.0086913
i2_LQ_YHS_c55875/f1p24/2444	5315.531277	663.3072587	3.0025	0.0010692	0.0086999
i1_LQ_YHS_c33697/f1p0/1736	24.92615319	105.2016336	-2.0774	0.0010694	0.0087003
i0_LQ_YHS_c2545/f1p3/364	166.4546324	48.32911411	1.7842	0.0010696	0.0087005
i2_HQ_YHS_c26213/f4p4/2128	90.26736473	429.0523209	-2.2489	0.0010717	0.008716
i5_LQ_YHS_c2558/f1p1/5281	14.53522111	214.0708172	-3.8805	0.0010721	0.0087174
i2_HQ_YHS_c61977/f63p1/2894	6242.459197	154.922703	5.3325	0.001073	0.008723
i2_LQ_YHS_c23882/f1p2/2778	13.62341726	0	Inf	0.0010733	0.0087246
i4_LQ_YHS_c8625/f1p0/4802	28.96818261	0.353872233	6.3551	0.0010736	0.0087253
i1_HQ_YHS_c2968/f6p0/1719	214.1016466	725.9697309	-1.7616	0.0010744	0.0087304
i1_LQ_YHS_c32990/f1p0/1839	19.4751805	89.34985686	-2.1978	0.0010768	0.0087471
i4_LQ_YHS_c13741/f1p0/4161	216.4819151	67.44437707	1.6825	0.0010767	0.0087471
i1_HQ_YHS_c40378/f14p0/1295	1285.939666	8941.714459	-2.7977	0.0010774	0.0087507
i3_LQ_YHS_c16879/f1p0/3571	6.702803467	151.3928245	-4.4974	0.0010777	0.0087511
i2_HQ_YHS_c6208/f3p1/2487	0.514474312	92.19457852	-7.4854	0.0010804	0.0087718
i1_LQ_YHS_c39013/f1p1/1147	1337.009457	11441.57694	-3.0972	0.0010815	0.008779
i2_LQ_YHS_c8450/f1p0/2783	0	12.52127146	-Inf	0.0010817	0.0087793
i3_LQ_YHS_c18124/f1p0/3111	2142.672822	33.41359564	6.0028	0.0010819	0.0087797
i2_HQ_YHS_c35074/f3p2/2867	468.2328171	6.015827962	6.2823	0.0010837	0.0087928
i2_HQ_YHS_c17334/f3p5/2171	1331.897697	360.2992829	1.8862	0.001086	0.0088082
i5_LQ_YHS_c4615/f1p0/5005	80.48120152	17.4915965	2.202	0.001086	0.0088082
i1_HQ_YHS_c2350/f3p6/1695	247.4517407	726.5891224	-1.554	0.0010882	0.0088246
i2_LQ_YHS_c7208/f1p4/2178	6.808532464	283.5317503	-5.38	0.0010891	0.0088306
i1_LQ_YHS_c13556/f1p4/1280	325.4071468	989.987718	-1.6052	0.0010894	0.0088312

i2_HQ_YHS_c7557/f2p1/2722	187.1839689	57.22374757	1.7098	0.0010901	0.0088353
i2_LQ_YHS_c3469/f1p0/2139	1816.999866	174.3959014	3.3811	0.0010907	0.0088363
i2_LQ_YHS_c56641/f1p0/2137	0	12.47510239	-Inf	0.0010905	0.0088363
i5_LQ_YHS_c3288/f1p2/5467	1.536273569	35.83777818	-4.544	0.0010906	0.0088363
i4_LQ_YHS_c10062/f1p0/4773	0	13.64295921	-Inf	0.0010926	0.0088496
i1_LQ_YHS_c10439/f1p3/1595	14.76017243	72.67552253	-2.2998	0.0010952	0.0088697
i1_LQ_YHS_c14473/f1p0/1315	424.1440701	1341.073976	-1.6608	0.0010966	0.0088777
i1_LQ_YHS_c36853/f1p0/1526	32.77381142	2.990654763	3.454	0.0010965	0.0088777
i4_HQ_YHS_c14593/f3p27/4220	332.2513197	87.23649577	1.9293	0.0010988	0.0088944
i2_LQ_YHS_c22549/f1p7/2226	21.53388316	146.7624648	-2.7688	0.0011006	0.0089073
i0_LQ_YHS_c2311/f1p0/971	459.0155717	96.14554489	2.2553	0.0011011	0.0089102
i2_LQ_YHS_c39320/f1p6/2770	78.91598343	407.7601471	-2.3693	0.0011019	0.0089146
i0_LQ_YHS_c905/f1p0/542	5026.796195	66.39156576	6.2425	0.001103	0.0089222
i3_LQ_YHS_c6206/f1p0/3763	0.771711468	20.11341126	-4.704	0.0011049	0.008936
i2_LQ_YHS_c33130/f1p1/2674	203.9421725	4.309342416	5.5645	0.0011063	0.0089459
i3_LQ_YHS_c8456/f1p1/3084	2.15774902	94.20090674	-5.4481	0.0011071	0.0089513
i2_LQ_YHS_c20345/f1p1/2241	25.30262942	103.0571909	-2.0261	0.0011075	0.0089523
i1_LQ_YHS_c29058/f1p0/1223	89.80829379	2.153841925	5.3819	0.0011088	0.0089619
i1_LQ_YHS_c20398/f1p2/1581	455.8882077	80.38516029	2.5037	0.001109	0.0089621
i2_LQ_YHS_c38389/f1p0/2249	26.9560394	317.6700463	-3.5588	0.0011097	0.0089661
i2_LQ_YHS_c39945/f1p7/2516	85.62140535	653.9515789	-2.9331	0.0011109	0.008974
i1_LQ_YHS_c43883/f1p2/1013	301.6057047	904.1207014	-1.5839	0.0011122	0.0089831
i2_HQ_YHS_c32643/f2p6/2404	36.57274922	134.7226347	-1.8812	0.0011134	0.008991
i2_HQ_YHS_c2643/f5p8/2743	321.9083831	1570.521226	-2.2865	0.0011166	0.0090144
i2_LQ_YHS_c26453/f1p1/2628	188.030945	546.5417662	-1.5394	0.0011165	0.0090144
i1_LQ_YHS_c35426/f1p0/1785	1.131336305	21.87996786	-4.2735	0.0011237	0.0090701
i1_LQ_YHS_c19899/f1p2/1786	376.342223	0	Inf	0.0011252	0.0090808
i1_LQ_YHS_c34278/f1p14/1966	106.8881555	320.4143298	-1.5838	0.0011268	0.009091
i4_LQ_YHS_c5183/f1p1/4945	433.9787733	1337.696284	-1.6241	0.0011269	0.009091
i3_LQ_YHS_c18566/f1p0/3095	27.54971441	168.2747771	-2.6107	0.0011271	0.0090913
i1_LQ_YHS_c26119/f1p0/1913	0	17.40644585	-Inf	0.0011275	0.0090928
i1_LQ_YHS_c14209/f1p0/1311	162.5667667	495.7251642	-1.6085	0.0011286	0.0091002
i2_LQ_YHS_c55704/f1p4/2173	36.9889742	137.0356805	-1.8894	0.0011288	0.0091002
i1_LQ_YHS_c28810/f1p0/1366	611.6766405	74.30132057	3.0413	0.00113	0.0091086
i3_LQ_YHS_c17524/f1p0/3461	41.71702561	148.5214806	-1.832	0.0011302	0.0091086
i1_LQ_YHS_c3489/f1p0/1587	75.82520982	233.9520048	-1.6255	0.0011329	0.009127
i2_HQ_YHS_c60363/f2p1/2069	179.1576472	53.242132	1.7506	0.0011327	0.009127
i3_LQ_YHS_c10495/f1p0/3089	0	28.88403227	-Inf	0.0011339	0.009134
i1_LQ_YHS_c20941/f1p1/1802	2097.386087	28.22067854	6.2157	0.0011347	0.009139
i1_LQ_YHS_c4666/f1p0/1544	148.2921659	425.2785977	-1.52	0.0011352	0.0091415
i3_LQ_YHS_c8146/f1p3/3119	215.8728141	46.53246155	2.2139	0.0011356	0.009143
i1_LQ_YHS_c24538/f1p1/1734	259.4490578	83.57107617	1.6344	0.0011373	0.009155
i6_LQ_YHS_c436/f1p0/6693	498.2126662	54.13897521	3.202	0.0011382	0.0091612
i2_HQ_YHS_c1627/f6p4/2627	340.844234	1043.844901	-1.6147	0.0011404	0.0091775
i1_HQ_YHS_c11319/f2p1/1712	443.728212	79.96667365	2.4722	0.001141	0.0091786
i1_LQ_YHS_c22418/f1p3/1880	3.052829538	82.37434849	-4.754	0.0011408	0.0091786
i1_LQ_YHS_c21673/f1p8/1803	20.85787673	133.3457334	-2.6765	0.0011415	0.0091807
i1_LQ_YHS_c8398/f1p1/1764	152.5313127	444.4656219	-1.543	0.0011416	0.0091807
i3_LQ_YHS_c5602/f1p1/3159	240.2638502	77.02439975	1.6412	0.001143	0.00919
i3_LQ_YHS_c5786/f1p136/3094	957.2047026	4147.159106	-2.1152	0.0011432	0.0091901
i3_LQ_YHS_c8102/f1p11/3746	83.68726134	18.31510014	2.192	0.0011437	0.0091929
i2_LQ_YHS_c35863/f1p2/2405	38.05020029	139.872307	-1.8781	0.0011441	0.0091944
i4_LQ_YHS_c14029/f1p0/4178	71.34898508	335.8606478	-2.2349	0.0011461	0.0092095
i2_LQ_YHS_c10480/f1p1/2392	22.14280712	0	Inf	0.0011481	0.0092241
i3_LQ_YHS_c11179/f1p1/3817	6.606301387	46.13526436	-2.804	0.0011491	0.0092299
i1_LQ_YHS_c32510/f1p0/1681	0	14.37906086	-Inf	0.0011517	0.009248
i3_LQ_YHS_c18202/f1p2/3199	0	14.37906086	-Inf	0.0011517	0.009248
i2_LQ_YHS_c59176/f1p4/2109	44.03690154	0.784522063	5.8108	0.001155	0.009273
i1_LQ_YHS_c17165/f2p2/1419	727.086279	2713.884499	-1.9002	0.0011578	0.009294
i1_LQ_YHS_c5948/f1p13/2054	11.97045728	64.76359659	-2.4357	0.0011584	0.0092972
i1_LQ_YHS_c36818/f1p1/1799	515.947159	1672.551139	-1.6968	0.0011594	0.0093033
i7_LQ_YHS_c129/f1p0/7282	15.41311994	81.74266908	-2.4069	0.0011599	0.009306
i1_LQ_YHS_c34706/f1p0/1607	133.6067256	378.8648197	-1.5037	0.0011605	0.0093093
i2_LQ_YHS_c9467/f1p2/2313	94.11003765	286.9324829	-1.6083	0.0011614	0.0093149
i2_LQ_YHS_c52585/f1p25/2398	1313.401021	268.4296217	2.2907	0.0011649	0.0093416
i2_LQ_YHS_c12862/f1p7/2845	46.88369195	1.176783094	5.3162	0.0011654	0.0093444
i2_HQ_YHS_c45726/f5p1/2100	196.2452087	22.19710986	3.1442	0.0011659	0.0093466
i3_HQ_YHS_c10101/f2p0/3499	1519.788951	127.4488631	3.5759	0.0011667	0.0093501
i3_LQ_YHS_c12613/f1p0/3133	77.14129033	5.215152579	3.8867	0.0011667	0.0093501
i0_LQ_YHS_c3579/f1p6/309	202.3618993	62.55675135	1.6937	0.001167	0.0093504
i1_LQ_YHS_c12285/f1p1/1813	2.682714017	29.54276355	-3.461	0.0011672	0.009351
i0_LQ_YHS_c2105/f1p2/594	897.8287118	3434.451423	-1.9356	0.0011678	0.0093538
i4_LQ_YHS_c4480/f1p0/4871	35.90549461	143.2734275	-1.9965	0.0011693	0.0093642
i1_LQ_YHS_c34272/f1p0/1442	170.439273	33.45759359	2.3489	0.0011702	0.0093699
i4_HQ_YHS_c12940/f2p0/4116	37.95416494	139.7910674	-1.8809	0.0011709	0.0093738
i2_HQ_YHS_c22005/f2p0/2231	13.36571337	134.8816397	-3.3351	0.0011713	0.0093755
i3_LQ_YHS_c19533/f1p0/3958	2.360446833	31.45556807	-3.7362	0.0011716	0.0093755
i4_LQ_YHS_c9710/f1p3/4393	38.33571299	141.0055256	-1.879	0.0011716	0.0093755
i1_LQ_YHS_c25804/f1p0/1450	4.532027853	38.72389094	-3.095	0.001175	0.0094002
i4_LQ_YHS_c3645/f1p0/4495	103.6995502	17.62125765	2.557	0.0011751	0.0094002
i2_LQ_YHS_c36765/f1p5/2087	9.032583654	52.20891203	-2.5311	0.0011758	0.0094041
i1_LQ_YHS_c43582/f1p0/1014	1279.032831	92.61239104	3.7877	0.0011781	0.0094211
i1_HQ_YHS_c17725/f2p2/1872	298.6858027	865.0281215	-1.5341	0.0011819	0.0094498
i4_LQ_YHS_c5994/f1p0/4464	40.9104924	5.039915138	3.021	0.0011839	0.0094644
i2_HQ_YHS_c48389/f2p7/3051	185.1496631	534.6433269	-1.5299	0.0011846	0.0094679
i1_LQ_YHS_c31928/f1p0/1726	125.3207169	361.3018943	-1.5276	0.0011861	0.0094788
i1_LQ_YHS_c18319/f1p0/1921	846.6366271	42.62821635	4.3119	0.0011867	0.0094816
i4_LQ_YHS_c10130/f1p0/4362	91.15613479	269.5061727	-1.5639	0.0011915	0.0095189
i1_LQ_YHS_c3073/f1p3/1639	0.719249673	18.04965613	-4.6493	0.0011918	0.0095194

i0_LQ_YHS_c1595/f1p0/793	610.9996252	208.6879762	1.5498	0.0011925	0.0095216
i1_LQ_YHS_c18967/f1p0/1779	460.4997713	157.933694	1.5439	0.0011924	0.0095216
i4_LQ_YHS_c7878/f1p3/4443	173.465433	54.0132251	1.6833	0.0011961	0.0095487
i5_LQ_YHS_c4070/f1p0/5145	158.060445	474.2796732	-1.5853	0.0011978	0.0095612
i2_LQ_YHS_c12970/f1p6/2324	1055.609677	115.7839551	3.1886	0.0012028	0.009599
i2_LQ_YHS_c8434/f1p0/2782	0	12.46838791	-Inf	0.001208	0.009639
i4_LQ_YHS_c6439/f1p3/4882	10.61796933	61.69501823	-2.5386	0.0012128	0.0096757
i5_LQ_YHS_c3498/f1p0/5776	0	15.3912309	-Inf	0.001214	0.0096843
i2_LQ_YHS_c20237/f1p8/2081	508.0513091	5.308083496	6.5806	0.0012146	0.0096852
i2_LQ_YHS_c9525/f1p0/2460	171.6433667	27.73607937	2.6296	0.0012145	0.0096852
i3_LQ_YHS_c18613/f1p0/3094	187.9014342	530.7492738	-1.4981	0.0012152	0.0096889
i4_LQ_YHS_c13681/f1p6/4048	0	12.43165768	-Inf	0.0012157	0.0096909
i3_LQ_YHS_c20666/f1p0/3058	92.75804873	453.7340349	-2.2903	0.0012163	0.0096946
i2_LQ_YHS_c33642/f1p0/2171	88.45201454	0	Inf	0.0012171	0.0096991
i4_LQ_YHS_c5252/f1p0/4660	13.91882585	0	Inf	0.0012193	0.0097147
i3_LQ_YHS_c10371/f1p0/4024	315.9323975	85.59067292	1.8841	0.0012206	0.009724
i3_LQ_YHS_c11815/f1p3/3263	1.800660093	40.48002312	-4.4906	0.0012218	0.0097316
i4_LQ_YHS_c6769/f1p0/4598	26.52792353	108.2312304	-2.0285	0.0012226	0.0097355
i5_LQ_YHS_c3144/f1p13/5794	118.8448142	372.557594	-1.6484	0.0012227	0.0097355
i2_LQ_YHS_c57947/f1p1/2054	867.3477227	12.59192736	6.106	0.0012239	0.0097434
i5_LQ_YHS_c2863/f1p0/5429	0	21.62005278	-Inf	0.0012241	0.0097435
i3_LQ_YHS_c3411/f1p4/3094	159.7165273	1207.046613	-2.9179	0.0012255	0.009753
i4_LQ_YHS_c8478/f1p0/4661	87.00687688	274.3714036	-1.6569	0.0012272	0.0097653
i2_LQ_YHS_c42039/f1p0/2376	914.93593	214.1046721	2.0954	0.0012317	0.0097965
i2_LQ_YHS_c62503/f2p2/2299	374.5261717	9.975168507	5.2306	0.0012318	0.0097965
i4_LQ_YHS_c2916/f1p0/4279	4.631074218	38.88131649	-3.0697	0.0012314	0.0097965
i2_HQ_YHS_c7237/f2p3/2513	42.08252767	147.6169758	-1.8106	0.0012321	0.0097976
i0_LQ_YHS_c3025/f1p0/389	6814.805964	53.12021158	7.0033	0.0012333	0.0098058
i1_LQ_YHS_c33430/f1p4/1546	16.59946232	154.1542073	-3.2152	0.0012364	0.0098285
i2_LQ_YHS_c8342/f1p1/2524	211.1804065	37.131506	2.5078	0.0012383	0.0098417
i2_LQ_YHS_c3269/f1p22/2749	1104.899835	5100.299491	-2.2067	0.0012386	0.0098432
i0_HQ_YHS_c340/f2p0/662	2382.457401	628.9380304	1.9215	0.0012405	0.009856
i2_HQ_YHS_c3513/f3p1/2475	303.1230649	1210.66696	-1.9978	0.0012424	0.0098701
i1_LQ_YHS_c11463/f1p8/1832	57533.38584	4.284855595	13.713	0.001243	0.0098728
i1_LQ_YHS_c38501/f1p0/1198	2681.160786	104.2757206	4.6844	0.0012443	0.0098815
i3_LQ_YHS_c14560/f1p0/3036	48.68007725	7.499721442	2.6984	0.0012456	0.0098902
i1_LQ_YHS_c28110/f1p0/1314	167.8427344	500.2897213	-1.5757	0.0012499	0.0099226
i8_LQ_YHS_c59/f1p0/8263	941.7678727	82.82711403	3.5072	0.001254	0.0099537
i2_HQ_YHS_c60222/f2p1/2114	83.74375017	255.535446	-1.6095	0.0012554	0.0099635
i2_HQ_YHS_c2211/f4p2/2514	69.22349677	223.7118653	-1.6923	0.0012581	0.0099785
i2_LQ_YHS_c54461/f1p3/2279	24.18766103	98.5016415	-2.0259	0.0012579	0.0099785
i3_LQ_YHS_c11862/f1p2/3196	215.0020562	54.0444264	1.9921	0.0012582	0.0099785
i3_LQ_YHS_c18642/f1p0/3240	6.945403216	57.42560341	-3.0476	0.0012579	0.0099785
i3_LQ_YHS_c2986/f1p0/3261	137.6902761	2.964509375	5.5375	0.0012584	0.0099785
i4_LQ_YHS_c13413/f1p2/4122	261.2438323	85.36326559	1.6137	0.0012586	0.0099785
i1_LQ_YHS_c43535/f1p0/1023	25.69580386	100.8712412	-1.9729	0.0012588	0.009979
i1_HQ_YHS_c2743/f3p0/1727	220.6527408	621.0996836	-1.493	0.0012591	0.0099794
i1_LQ_YHS_c22368/f1p1/1784	449.7623415	1430.294364	-1.6691	0.0012597	0.0099815
i1_LQ_YHS_c24597/f1p14/1918	21.95059162	91.53359477	-2.06	0.0012601	0.0099815
i3_LQ_YHS_c18699/f1p0/3236	102.3232147	2.186109018	5.5486	0.0012602	0.0099815
i5_LQ_YHS_c4465/f1p0/5707	160.756389	25.52548353	2.6549	0.0012599	0.0099815
i5_LQ_YHS_c4337/f1p0/5467	126.6581175	512.4597218	-2.0165	0.0012613	0.0099884
i3_HQ_YHS_c2501/f2p2/3736	157.8891247	454.499204	-1.5254	0.0012615	0.009989
i1_LQ_YHS_c37056/f1p0/1550	0	14.38518257	-Inf	0.0012627	0.0099963
i2_LQ_YHS_c28550/f1p1/2037	319.2521698	905.3747449	-1.5038	0.0012641	0.010005
i2_LQ_YHS_c35561/f1p0/2761	2.980650142	47.16520675	-3.984	0.0012642	0.010005
i3_HQ_YHS_c15404/f9p0/3253	273.2121629	20.77715778	3.717	0.0012656	0.010015
i2_LQ_YHS_c3936/f1p0/2403	112.2184482	324.686942	-1.5327	0.0012663	0.010019
i1_LQ_YHS_c17539/f1p0/1956	9.12955247	54.9825432	-2.5904	0.0012685	0.010034
i1_HQ_YHS_c3461/f4p1/1718	249.4272168	47.76765712	2.3845	0.001269	0.010037
i1_HQ_YHS_c13391/f2p0/1351	629.2566431	2089.905604	-1.7317	0.0012711	0.01005
i2_LQ_YHS_c55605/f1p4/2587	16.88771317	102.5595617	-2.6024	0.0012712	0.01005
i2_LQ_YHS_c58177/f1p8/2226	4521.340648	76.22937194	5.8903	0.0012714	0.01005
i2_HQ_YHS_c60925/f10p4/2352	305.293004	892.0744276	-1.547	0.0012744	0.010068
i2_LQ_YHS_c51962/f1p1/2357	0	12.16127753	-Inf	0.0012745	0.010068
i2_LQ_YHS_c9407/f1p0/2777	20.11048795	90.39413469	-2.1683	0.0012743	0.010068
i4_LQ_YHS_c7640/f1p1/4629	0	12.16127753	-Inf	0.0012745	0.010068
i2_LQ_YHS_c36380/f1p1/2226	53.77763263	329.3333627	-2.6145	0.0012777	0.010089
i2_LQ_YHS_c41877/f1p0/2262	82.25659708	254.1071626	-1.6272	0.0012777	0.010089
i3_LQ_YHS_c6924/f1p3/3621	86.38656225	19.8824857	2.1193	0.0012774	0.010089
i2_LQ_YHS_c11858/f1p1/2377	211.4889243	65.65317322	1.6876	0.0012788	0.010096
i2_LQ_YHS_c53475/f1p5/2799	149.5935923	422.4120741	-1.4976	0.0012805	0.010107
i2_LQ_YHS_c4444/f1p3/3046	37.65417639	0.359993938	6.7087	0.0012826	0.010122
i3_LQ_YHS_c5409/f1p0/3410	380.1512113	51.78319835	2.876	0.0012873	0.010158
i1_LQ_YHS_c26279/f1p0/1575	2.950441857	32.37255586	-3.4558	0.0012909	0.010185
i2_LQ_YHS_c26140/f1p1/2229	185.9378383	31.23250299	2.5737	0.0012921	0.010193
i3_LQ_YHS_c17166/f1p0/4080	110.2190838	325.6040842	-1.5627	0.001293	0.010198
i0_LQ_YHS_c840/f1p0/778	968.3541819	4094.660759	-2.0801	0.0012946	0.010208
i2_LQ_YHS_c60312/f10p4/2261	237.217695	669.7102406	-1.4973	0.0012947	0.010208
i2_HQ_YHS_c24365/f3p1/2138	27.60679804	108.1847081	-1.9704	0.0012975	0.010229
i1_LQ_YHS_c4527/f1p0/1509	121.2203652	31.24312739	1.956	0.0012979	0.01023
i2_HQ_YHS_c4910/f2p3/2493	130.3180818	379.6207055	-1.5425	0.0012992	0.010237
i5_LQ_YHS_c2288/f1p1/5639	951.1744269	190.3919922	2.3207	0.0012991	0.010237
i1_LQ_YHS_c23702/f1p0/1468	161.2208513	3.300016492	5.6104	0.0013001	0.010242
i1_HQ_YHS_c10551/f2p0/1426	340.3985488	1022.769087	-1.5872	0.0013006	0.010244
i1_LQ_YHS_c10842/f1p0/1919	26.27990491	127.2328657	-2.2754	0.0013018	0.010252
i2_HQ_YHS_c48307/f4p4/2576	867.9928054	305.1998356	1.5079	0.0013022	0.010252
i4_LQ_YHS_c4661/f1p73/4891	114.7229642	445.2891195	-1.9566	0.0013021	0.010252
i1_LQ_YHS_c37845/f2p0/1260	111.8545402	326.1639606	-1.544	0.0013025	0.010253

i1_LQ_YHS_c6035/f1p1/1406	205.5387873	588.5464416	-1.5177	0.0013038	0.010262
i2_LQ_YHS_c40385/f1p1/2752	5.345710433	144.6490679	-4.758	0.0013045	0.010265
i1_HQ_YHS_c2489/f3p0/1912	186.8973624	56.55052154	1.7246	0.0013054	0.010267
i5_LQ_YHS_c1353/f1p0/5133	127.5328437	370.395901	-1.5382	0.0013053	0.010267
i5_LQ_YHS_c3965/f1p0/5370	142.7041693	19.01391833	2.9079	0.0013052	0.010267
i4_LQ_YHS_c10536/f1p0/4296	309.1917781	94.88203168	1.7043	0.0013083	0.010289
i2_LQ_YHS_c39988/f1p9/2867	1.438499347	56.42235965	-5.2936	0.0013099	0.010299
i1_LQ_YHS_c19946/f1p19/1500	555.7064539	1807.29948	-1.7014	0.0013145	0.010334
i1_LQ_YHS_c39733/f1p0/1332	348.7978749	993.6074419	-1.5103	0.0013164	0.010347
i1_LQ_YHS_c14172/f1p0/1334	0	11.97545524	-Inf	0.0013171	0.010351
i2_LQ_YHS_c48924/f2p1/2113	1074.915551	358.3857263	1.5846	0.0013225	0.01039
i2_LQ_YHS_c64473/f1p2/2019	1480.465747	471.9554174	1.6493	0.0013225	0.01039
i4_LQ_YHS_c13007/f1p0/4110	223.7528239	71.376346	1.6484	0.0013231	0.010393
i3_LQ_YHS_c12060/f1p0/3161	59.64858931	0	Inf	0.0013239	0.010398
i1_LQ_YHS_c25726/f1p0/1711	388.1278141	131.751018	1.5587	0.0013279	0.010428
i1_LQ_YHS_c31948/f1p0/1673	2322.500305	680.1362811	1.7718	0.0013286	0.01043
i2_LQ_YHS_c36108/f1p2/2793	170.6641992	50.99097624	1.7428	0.0013285	0.01043
i2_HQ_YHS_c30150/f6p0/2569	86.2101905	2.566126639	5.0702	0.0013301	0.010439
i2_LQ_YHS_c34113/f1p4/2379	73.25522935	226.0396058	-1.6256	0.0013308	0.010443
i2_LQ_YHS_c9746/f1p2/2509	752.7797792	34.59211752	4.4437	0.0013352	0.010476
i3_LQ_YHS_c5681/f1p0/3495	13.69385778	133.6775652	-3.2872	0.0013385	0.0105
i2_LQ_YHS_c21079/f1p1/2422	14.5423621	99.83016078	-2.7792	0.001339	0.010502
i1_LQ_YHS_c33953/f1p2/1801	46.54987255	175.2446835	-1.9125	0.0013455	0.010551
i1_LQ_YHS_c8052/f1p1/1821	14.69975586	70.63191085	-2.2645	0.0013456	0.010551
i3_LQ_YHS_c22548/f1p1/3041	241.8687454	733.4883729	-1.6005	0.0013463	0.010554
i1_LQ_YHS_c24474/f1p2/1722	11.04263257	57.63990265	-2.384	0.0013469	0.010557
i2_LQ_YHS_c40683/f1p3/2648	43.98255605	169.891929	-1.9496	0.001349	0.010572
i2_HQ_YHS_c5775/f2p9/3121	172.6126623	53.63048312	1.6864	0.0013499	0.010578
i1_LQ_YHS_c9971/f1p0/1785	2607.815915	736.0176375	1.825	0.001351	0.010585
i1_HQ_YHS_c29187/f14p0/1807	544.5236309	1714.287228	-1.6545	0.0013532	0.010598
i2_LQ_YHS_c55634/f1p3/2456	355.3586892	1069.725284	-1.5899	0.0013532	0.010598
i2_LQ_YHS_c9993/f1p10/2549	1402.028368	14091.38157	-3.3292	0.001357	0.010626
i1_LQ_YHS_c31747/f1p0/1865	205.7875167	64.14032978	1.6819	0.0013585	0.010637
i3_LQ_YHS_c17068/f1p0/3317	19.30315771	0.719987877	4.7447	0.0013595	0.010642
i3_LQ_YHS_c8567/f1p1/3820	277.5646632	24.74542461	3.4876	0.0013596	0.010642
i1_HQ_YHS_c2019/f4p0/1944	788.7215734	270.4919985	1.5439	0.0013611	0.01065
i2_LQ_YHS_c13323/f1p1/2156	45.66859891	759.0096422	-4.0548	0.0013611	0.01065
i1_HQ_YHS_c31633/f2p0/1507	145.8729468	395.4415193	-1.4388	0.0013652	0.010675
i2_HQ_YHS_c25913/f2p2/2451	675.1718617	190.5077118	1.8254	0.001365	0.010675
i2_LQ_YHS_c33602/f1p0/2444	119.8487628	20.37320657	2.5565	0.0013647	0.010675
i4_LQ_YHS_c13251/f1p2/4127	99.90393697	25.57161306	1.966	0.0013648	0.010675
i2_HQ_YHS_c29660/f28p2/2266	404.4091608	9.354233252	5.4341	0.0013659	0.010679
i0_LQ_YHS_c1661/f1p0/602	98.91109061	14.08253532	2.8122	0.0013663	0.010681
i1_LQ_YHS_c19262/f1p2/1998	702.2738946	248.1692176	1.5007	0.0013682	0.010693
i2_LQ_YHS_c19860/f1p0/2458	61.08556877	10.72576491	2.5098	0.0013695	0.0107
i2_LQ_YHS_c49779/f1p2/2516	0.272341299	15.0044988	-5.7838	0.0013695	0.0107
i2_HQ_YHS_c18151/f2p3/2631	229.4913952	56.60996026	2.0193	0.0013704	0.010705
i3_HQ_YHS_c21661/f3p4/3334	2980.414609	800.3929609	1.8967	0.0013758	0.010746
i1_LQ_YHS_c8534/f2p0/1865	504.5756701	124.4548505	2.0194	0.0013766	0.01075
i1_LQ_YHS_c33921/f1p15/1547	93.96916499	4.984819789	4.2366	0.0013812	0.010784
i5_LQ_YHS_c2668/f1p0/5193	52.13437468	170.6212371	-1.7105	0.0013816	0.010786
i2_LQ_YHS_c34138/f1p2/3323	33.21482582	123.6943027	-1.8969	0.0013826	0.010792
i2_HQ_YHS_c17674/f6p0/2292	400.2262827	136.543774	1.5515	0.0013834	0.010796
i2_HQ_YHS_c4354/f4p0/2883	56.64221628	7.453552372	2.9259	0.0013847	0.010805
i2_LQ_YHS_c38316/f1p5/2310	99.637717	7.324484001	3.7659	0.0013875	0.010825
i2_LQ_YHS_c40013/f1p25/2784	268.7257609	7659.501917	-4.833	0.0013878	0.010825
i2_HQ_YHS_c33960/f2p2/2432	565.145472	198.1864213	1.5118	0.0013908	0.010847
i1_LQ_YHS_c11609/f1p0/1971	96.72010553	285.9467373	-1.5639	0.001392	0.010854
i3_LQ_YHS_c6602/f1p4/3123	125.4274045	3.312259903	5.2429	0.001393	0.010861
i2_HQ_YHS_c3459/f2p0/2243	43.44804222	728.9314169	-4.0684	0.0013934	0.010863
i2_LQ_YHS_c56596/f1p0/2278	97.55894133	294.0061758	-1.5915	0.0013942	0.010867
i5_HQ_YHS_c368/f8p0/5571	556.0546559	198.7373736	1.4844	0.0013954	0.010875
i2_LQ_YHS_c53881/f1p3/2513	9.067863758	55.58791792	-2.6159	0.0013967	0.010882
i3_LQ_YHS_c6271/f1p0/3533	10.54833422	59.1605659	-2.4876	0.0013988	0.010897
i1_LQ_YHS_c4057/f1p1/1608	187.037888	58.75827326	1.6705	0.0014013	0.010915
i2_LQ_YHS_c38938/f1p6/2738	44.95665179	155.3473437	-1.7889	0.0014052	0.010944
i3_LQ_YHS_c19556/f1p0/3708	202.8364754	66.10890265	1.6174	0.0014069	0.010955
i3_LQ_YHS_c12811/f1p1/3202	340.6037621	994.723525	-1.5462	0.0014074	0.010957
i4_LQ_YHS_c5925/f1p3/4822	12.64185024	67.05314116	-2.4071	0.001408	0.01096
i2_LQ_YHS_c38025/f1p1/2774	222.9254374	71.30181975	1.6446	0.0014084	0.010961
i4_LQ_YHS_c7611/f1p0/4917	10.15469304	279.575423	-4.783	0.0014091	0.010965
i3_LQ_YHS_c14441/f1p12/3052	219.3444535	625.5225338	-1.5119	0.0014115	0.010982
i0_LQ_YHS_c2669/f1p0/944	1046.289782	24.19739659	5.4343	0.0014136	0.010996
i1_LQ_YHS_c32154/f1p9/1825	56.01726308	179.0441076	-1.6764	0.0014157	0.011011
i2_LQ_YHS_c55187/f1p1/2671	3.87908035	50.87237182	-3.7131	0.0014172	0.011021
i8_LQ_YHS_c30/f1p0/8896	237.5702493	3336.849497	-3.8121	0.0014184	0.011028
i2_LQ_YHS_c7140/f1p1/2383	57.92445757	184.0960661	-1.6682	0.0014193	0.011034
i2_HQ_YHS_c2250/f6p0/2896	247.6606927	24.96130219	3.3106	0.0014199	0.011037
i2_HQ_YHS_c12139/f3p1/2764	136.0736649	551.9532544	-2.0202	0.0014208	0.011042
i2_LQ_YHS_c44391/f1p0/2053	174.7411365	53.68174765	1.7027	0.0014217	0.011048
i2_LQ_YHS_c53910/f1p4/2008	180.0599884	508.1463195	-1.4968	0.0014231	0.011054
i4_HQ_YHS_c1921/f4p0/4813	1599.821039	522.0269128	1.6157	0.0014229	0.011054
i1_HQ_YHS_c1923/f5p0/1509	388.0129013	2652.09214	-2.773	0.0014274	0.011085
i2_HQ_YHS_c57857/f2p0/2040	78.67753041	238.1982381	-1.5981	0.0014275	0.011085
i3_LQ_YHS_c11906/f1p0/3574	86.87514494	3.433548003	4.6612	0.0014284	0.01109
i7_LQ_YHS_c223/f1p0/7260	13.31244617	69.44837373	-2.3832	0.0014297	0.011099
i2_LQ_YHS_c32752/f1p0/2781	0	12.08224859	-Inf	0.0014313	0.011109
i4_LQ_YHS_c7635/f1p0/4224	34.5790766	125.0402411	-1.8544	0.0014336	0.011126
i2_LQ_YHS_c13241/f6p11/2238	13501.97204	354.3692021	5.2518	0.0014349	0.011132

i2_LQ_YHS_c7623/f1p4/2690	456.551793	1420.624244	-1.6377	0.0014347	0.011132
i4_LQ_YHS_c4090/f1p0/4253	191.7358508	61.14801645	1.6487	0.0014395	0.011164
i5_LQ_YHS_c1749/f1p3/5235	10.83577966	59.81882417	-2.4648	0.0014394	0.011164
i2_LQ_YHS_c58289/f1p0/2031	8.458151133	125.1578984	-3.8873	0.0014451	0.011205
i2_LQ_YHS_c40511/f1p0/2343	0	12.02549467	-Inf	0.0014455	0.011207
i2_LQ_YHS_c40868/f1p2/2096	558.9120243	196.8321494	1.5057	0.0014486	0.011229
i1_LQ_YHS_c36283/f1p1/1721	1872.04178	578.9456887	1.6931	0.0014491	0.011232
i2_LQ_YHS_c55112/f1p0/2188	117.1570828	0.359993938	8.3463	0.0014502	0.011237
i3_LQ_YHS_c8354/f1p1/3253	72.67123962	228.0932096	-1.6502	0.0014503	0.011237
i3_LQ_YHS_c22158/f1p1/3497	385.3024994	1148.892386	-1.5762	0.0014548	0.011266
i3_LQ_YHS_c9949/f1p36/3516	279.7082081	32.54680773	3.1033	0.0014543	0.011266
i5_LQ_YHS_c4463/f1p2/5694	0.544682597	17.30577421	-4.9897	0.0014545	0.011266
i5_LQ_YHS_c3537/f1p0/5120	38.90309909	0	Inf	0.001456	0.011273
i2_LQ_YHS_c51282/f1p0/2734	0	11.98098417	-Inf	0.0014568	0.011278
i3_LQ_YHS_c9179/f1p4/3598	612.2389034	216.6041725	1.499	0.001457	0.011278
i4_LQ_YHS_c13354/f1p54/4198	245.7783618	82.88438163	1.5682	0.0014584	0.011287
i6_LQ_YHS_c599/f1p0/6965	9.547057967	133.5478634	-3.8062	0.0014589	0.011289
i2_LQ_YHS_c51548/f1p1/2607	80.49031712	17.4915965	2.2022	0.0014594	0.011291
i2_LQ_YHS_c44710/f1p0/2019	137.2934623	377.979626	-1.461	0.0014606	0.011298
i1_LQ_YHS_c7988/f1p8/1555	485.0970907	169.7764836	1.5146	0.0014614	0.011303
i2_HQ_YHS_c6664/f3p0/2582	0	11.95649735	-Inf	0.001463	0.011314
i1_HQ_YHS_c40719/f3p5/1287	1220.218994	19284.58873	-3.9822	0.0014637	0.011317
i2_LQ_YHS_c13794/f1p1/3055	74.73122279	230.1934147	-1.6231	0.0014642	0.011319
i2_LQ_YHS_c39036/f1p11/2739	2.723412986	46.77294572	-4.1022	0.0014655	0.011325
i3_HQ_YHS_c2470/f2p0/3740	1311.216306	260.6310097	2.3308	0.0014653	0.011325
i2_LQ_YHS_c7302/f1p0/2654	138.7920396	383.4951338	-1.4663	0.0014681	0.011344
i4_HQ_YHS_c12959/f2p0/4084	276.9667373	801.2462073	-1.5325	0.0014687	0.011346
i1_LQ_YHS_c14702/f1p3/1211	30.89300878	2.976752786	3.3755	0.0014702	0.011356
i1_HQ_YHS_c29259/f10p0/1772	1250.896741	6746.429194	-2.4312	0.0014711	0.011362
i1_LQ_YHS_c7082/f1p0/1848	170.1330099	51.64579648	1.7199	0.0014732	0.011376
i3_LQ_YHS_c8492/f1p1/3144	356.9725539	20.93411033	4.0919	0.0014738	0.011378
i1_LQ_YHS_c43554/f1p0/1056	163.1798087	0	Inf	0.0014774	0.011403
i2_LQ_YHS_c52146/f1p6/2115	200.6146417	625.699704	-1.641	0.0014774	0.011403
i1_LQ_YHS_c21864/f1p0/1947	180.784901	55.08933655	1.7144	0.0014797	0.011419
i2_LQ_YHS_c40479/f2p2/2707	6.423312801	45.20046469	-2.8149	0.0014801	0.01142
i2_HQ_YHS_c2776/f2p0/2142	52.69049818	167.2310536	-1.6662	0.0014808	0.011423
i2_LQ_YHS_c51920/f1p4/2887	52.45584483	8.868448528	2.5643	0.0014813	0.011426
i3_LQ_YHS_c4970/f1p37/3138	75.50213723	228.3281253	-1.5965	0.0014823	0.011432
i1_HQ_YHS_c17078/f2p0/1685	586.5324342	208.6318546	1.4913	0.0014832	0.011435
i1_LQ_YHS_c11482/f1p0/1844	160.9386536	1256.03532	-2.9643	0.0014832	0.011435
i2_LQ_YHS_c12370/f1p4/2432	829.4360327	3010.86122	-1.86	0.0014835	0.011436
i1_LQ_YHS_c5541/f1p2/1598	29.21190131	114.3222971	-1.9685	0.0014839	0.011437
i3_LQ_YHS_c4004/f1p0/3086	235.4563792	55.49443377	2.085	0.0014862	0.011453
i2_LQ_YHS_c10184/f1p2/2326	18.22716615	217.9955339	-3.5801	0.0014891	0.01147
i2_LQ_YHS_c23477/f1p1/2172	868.4234713	299.5879984	1.5354	0.0014891	0.01147
i4_LQ_YHS_c9749/f1p0/4764	1.521169427	22.7290241	-3.9013	0.0014892	0.01147
i1_LQ_YHS_c8821/f1p16/1989	104.6278249	305.4922969	-1.5459	0.0014903	0.011477
i1_LQ_YHS_c33004/f1p27/2036	1.983181944	24.44550172	-3.6237	0.0014928	0.011495
i2_LQ_YHS_c59281/f1p2/2054	7.938605002	201.3473355	-4.6647	0.0014961	0.011518
i2_HQ_YHS_c6054/f2p0/2656	0	11.82742897	-Inf	0.0014966	0.01152
i1_HQ_YHS_c41021/f19p0/1913	322.308586	40238.7609	-6.964	0.0014971	0.011522
i4_LQ_YHS_c14012/f1p1/4040	0	35.70531246	-Inf	0.0014975	0.011523
i1_LQ_YHS_c17413/f1p0/1481	293.76266	4.089001694	6.1668	0.0015029	0.011563
i1_LQ_YHS_c10786/f1p0/1912	47.14669501	5.80773065	3.0211	0.0015043	0.011572
i1_LQ_YHS_c23641/f1p0/1853	171.734718	19.2125768	3.1601	0.0015054	0.011578
i2_LQ_YHS_c26548/f2p2/2287	3.529946197	55.77362044	-3.9819	0.0015057	0.011578
i2_LQ_YHS_c50270/f1p0/2241	0	19.72162324	-Inf	0.0015061	0.011578
i3_LQ_YHS_c5858/f1p0/3387	58.7549748	186.0664162	-1.663	0.0015059	0.011578
i0_LQ_YHS_c3111/f1p0/899	366.467728	127.0570751	1.5282	0.0015093	0.011599
i5_LQ_YHS_c3016/f1p1/5152	40.95072463	143.8671527	-1.8128	0.0015092	0.011599
i2_LQ_YHS_c22548/f1p1/2127	157.7643147	41.86810089	1.9138	0.0015109	0.011608
i2_LQ_YHS_c52583/f1p0/2919	252.2879505	44.50215907	2.5031	0.0015107	0.011608
i3_LQ_YHS_c12240/f1p0/3981	126.1728019	9.621296274	3.713	0.0015116	0.011611
i1_LQ_YHS_c12157/f1p0/1927	19.64466738	97.95250814	-2.3179	0.0015133	0.011623
i1_LQ_YHS_c6485/f1p9/1500	16.66114266	346.7024894	-4.3791	0.001515	0.011633
i1_LQ_YHS_c25571/f1p0/1863	19.8322778	82.36309178	-2.0541	0.0015164	0.011643
i4_LQ_YHS_c7956/f1p0/4984	10.5467234	61.72621953	-2.5491	0.0015278	0.011728
i0_LQ_YHS_c2090/f1p0/448	32.12753817	117.561057	-1.8715	0.0015319	0.011758
i1_LQ_YHS_c19713/f1p0/1911	100.9146591	24.96019686	2.0154	0.001533	0.011765
i2_HQ_YHS_c3486/f3p1/2629	117.1426345	16.78997374	2.8026	0.0015357	0.011781
i3_LQ_YHS_c6989/f1p0/3193	606.9919869	214.9563725	1.4976	0.0015355	0.011781
i6_LQ_YHS_c783/f1p0/6275	0	11.67999549	-Inf	0.0015363	0.011784
i2_LQ_YHS_c49388/f1p0/2861	0	11.67833692	-Inf	0.0015368	0.011785
i4_HQ_YHS_c2373/f2p0/5001	136.7141974	39.23191114	1.8011	0.0015369	0.011785
i2_HQ_YHS_c22905/f2p3/2417	94.79309887	18.88872032	2.3273	0.0015385	0.011795
i4_LQ_YHS_c13521/f1p5/4115	13.45554119	66.32359468	-2.3013	0.0015387	0.011795
i2_LQ_YHS_c41867/f1p2/2212	52.47519863	178.275067	-1.7644	0.0015428	0.011823
i2_LQ_YHS_c51982/f1p1/2500	3.839645148	35.32813895	-3.2018	0.0015429	0.011823
i2_HQ_YHS_c6621/f2p1/2224	94.27805016	6.159944317	3.9359	0.0015469	0.011852
i2_LQ_YHS_c28617/f1p0/2066	141.1366104	17.56896687	3.006	0.0015479	0.011856
i4_LQ_YHS_c4394/f1p2/5103	194.6088141	43.18512998	2.172	0.0015476	0.011856
i1_HQ_YHS_c6933/f3p3/1788	6466.20737	1260.928525	2.3584	0.0015517	0.011882
i3_LQ_YHS_c19769/f1p1/3484	9.271375352	51.7371095	-2.4803	0.0015517	0.011882
i1_LQ_YHS_c43989/f1p0/1057	412.6339092	1184.513698	-1.5214	0.0015536	0.011894
i2_LQ_YHS_c21045/f1p0/2922	0	15.09739017	-Inf	0.001554	0.011894
i2_LQ_YHS_c32786/f1p3/2742	147.5996384	413.9047271	-1.4876	0.001554	0.011894
i4_LQ_YHS_c8612/f1p0/4394	41.19714081	139.9452538	-1.7642	0.0015548	0.011898
i2_LQ_YHS_c19291/f1p3/2997	954.3738169	332.0941671	1.523	0.0015565	0.011909
i2_LQ_YHS_c34823/f1p3/2297	0.991590972	18.98891894	-4.2593	0.0015613	0.011944

i2_LQ_YHS_c56089/f1p2/2210	6.734283894	45.93767168	-2.7701	0.0015622	0.011949
i4_LQ_YHS_c5523/f1p0/4446	624.2601901	2052.897396	-1.7174	0.0015662	0.011977
i3_LQ_YHS_c18221/f1p0/3199	218.2328683	71.20497892	1.6158	0.0015674	0.011984
i1_HQ_YHS_c26573/f2p0/1736	88.88169097	20.66147883	2.1049	0.0015697	0.012
i1_LQ_YHS_c42144/f1p0/1867	147.6593584	0	Inf	0.0015744	0.012033
i2_LQ_YHS_c28750/f1p0/2008	37.12491986	130.0750608	-1.8089	0.0015745	0.012033
i4_HQ_YHS_c2515/f2p1/4643	94.96696348	23.69770989	2.0027	0.0015774	0.012054
i1_HQ_YHS_c15017/f10p0/1787	910.5003384	140.6200593	2.6949	0.001581	0.012079
i3_LQ_YHS_c17260/f1p4/3954	243.7956383	82.34192209	1.566	0.0015837	0.012097
i3_LQ_YHS_c7578/f1p3/3103	71.12081213	15.82456554	2.1681	0.0015854	0.012109
i2_LQ_YHS_c35815/f1p0/2380	30.75025243	114.7034201	-1.8992	0.0015859	0.012111
i2_LQ_YHS_c53117/f1p1/2372	49.02906659	166.1058883	-1.7604	0.0015873	0.012119
i2_LQ_YHS_c58762/f1p2/2120	65.23756846	202.6506649	-1.6352	0.0015888	0.012129
i3_LQ_YHS_c3224/f1p2/3199	17.89486664	81.86838077	-2.1938	0.0015893	0.012131
i1_HQ_YHS_c6361/f4p0/1914	260.0781661	711.971587	-1.4529	0.0015899	0.012133
i1_LQ_YHS_c19283/f1p0/1576	321.1761295	872.7862983	-1.4423	0.0015947	0.012164
i2_LQ_YHS_c21702/f1p2/2943	84.71609028	255.9509292	-1.5952	0.0015944	0.012164
i2_LQ_YHS_c7310/f1p0/2361	64.49372323	243.1679297	-1.9147	0.0015945	0.012164
i1_LQ_YHS_c28588/f1p5/1162	209.2064671	585.4741126	-1.4847	0.0016001	0.012203
i1_LQ_YHS_c7597/f1p1/1652	237.7552784	41.69957793	2.5114	0.0016016	0.012213
i2_LQ_YHS_c8856/f1p2/2560	320.2074109	85.71930895	1.9013	0.0016051	0.012238
i1_LQ_YHS_c39645/f1p3/1119	1989.980196	606.4724456	1.7142	0.0016057	0.012241
i2_LQ_YHS_c23473/f1p2/3187	323.3860173	114.0808263	1.5032	0.0016078	0.012255
i4_LQ_YHS_c13227/f1p3/4167	391.7640208	142.7326683	1.4567	0.0016103	0.012272
i2_LQ_YHS_c21578/f1p49/2579	5823.36924	98.01305108	5.8927	0.0016114	0.012278
i2_LQ_YHS_c24344/f1p8/2285	12.50671837	372.29092	-4.8957	0.0016127	0.012286
i1_HQ_YHS_c8264/f2p0/1582	1617.345471	462.0894842	1.8074	0.001617	0.012315
i3_LQ_YHS_c5240/f1p1/3350	97.26908804	353.153509	-1.8602	0.0016168	0.012315
i2_LQ_YHS_c44299/f1p4/1821	6.253359183	43.20468065	-2.7885	0.0016215	0.012348
i1_HQ_YHS_c2607/f3p3/1739	829.6419629	134.2576268	2.6275	0.0016306	0.012415
i1_LQ_YHS_c9419/f1p0/1461	863.5901457	3288.875865	-1.9292	0.0016347	0.01244
i3_HQ_YHS_c15314/f9p0/3805	491.8300953	1425.266207	-1.535	0.0016346	0.01244
i7_LQ_YHS_c161/f1p0/7951	46.69712267	661.231978	-3.8238	0.0016345	0.01244
i3_HQ_YHS_c15576/f4p0/3137	730.4066415	56.85466804	3.6833	0.0016367	0.012454
i1_HQ_YHS_c9967/f3p5/1501	102.0253192	322.9632302	-1.6624	0.0016401	0.012478
i1_LQ_YHS_c21144/f1p0/1509	155.4336956	441.4799429	-1.506	0.0016408	0.012481
i3_LQ_YHS_c3393/f2p0/3684	100.1115953	23.92871567	2.0648	0.0016417	0.012485
i1_LQ_YHS_c13191/f1p1/1403	186.8756079	522.8464669	-1.4843	0.0016439	0.012496
i3_LQ_YHS_c13296/f1p1/3161	109.2009155	306.6960912	-1.4898	0.0016436	0.012496
i4_LQ_YHS_c5476/f1p0/5025	17.44541399	81.36439024	-2.2215	0.0016434	0.012496
i2_LQ_YHS_c13327/f1p2/2659	7.313788234	54.37428483	-2.8942	0.0016489	0.012532
i3_LQ_YHS_c3467/f1p0/3825	72.02847763	13.52060532	2.4134	0.0016501	0.012539
i3_LQ_YHS_c3588/f1p1/3820	40.810649	5.498368921	2.8919	0.0016502	0.012539
i2_LQ_YHS_c18528/f1p37/2195	102.3585606	290.9219194	-1.507	0.0016528	0.012556
i2_LQ_YHS_c24217/f1p1/2713	85.35233958	20.19796913	2.0792	0.0016539	0.01256
i2_LQ_YHS_c64640/f1p0/2034	176.6786302	483.6420043	-1.4528	0.0016536	0.01256
i7_LQ_YHS_c233/f1p0/7429	1.710840645	34.87466087	-4.3494	0.0016556	0.012572
i2_LQ_YHS_c33524/f1p0/2675	253.7298821	85.20797162	1.5742	0.0016565	0.012577
i1_LQ_YHS_c29334/f1p3/1766	752.8863052	47.25916391	3.9938	0.0016593	0.012596
i1_LQ_YHS_c17672/f1p0/1501	454.6368531	93.75939903	2.2777	0.0016605	0.012603
i3_HQ_YHS_c1892/f3p1/3145	123.6408481	358.5048817	-1.5358	0.001662	0.012612
i3_HQ_YHS_c13878/f2p0/3060	142.0918382	401.6357486	-1.4991	0.0016693	0.012666
i2_LQ_YHS_c23150/f1p2/2653	42.1110222	135.9240243	-1.6905	0.0016723	0.012687
i4_LQ_YHS_c8231/f1p0/4848	9.795068206	119.5433308	-3.6093	0.0016734	0.012693
i2_LQ_YHS_c54043/f1p44/2722	0.719249673	16.69376524	-4.5367	0.0016739	0.012694
i2_HQ_YHS_c60241/f4p2/2580	350.228258	122.1697284	1.5194	0.0016758	0.012707
i2_LQ_YHS_c39274/f1p0/2619	4.249195871	46.15358993	-3.4412	0.001678	0.012722
i2_LQ_YHS_c41121/f1p4/2848	297.8294585	856.9141111	-1.5247	0.0016787	0.012725
i2_LQ_YHS_c64488/f1p1/2072	310.2700447	89.46652251	1.7941	0.0016793	0.012725
i3_LQ_YHS_c13431/f1p0/3175	120.1325199	12.0811828	3.3138	0.0016789	0.012725
i2_LQ_YHS_c10962/f1p3/2416	8.987262853	54.1440718	-2.5908	0.001681	0.012737
i1_LQ_YHS_c22625/f1p6/1943	36.82710342	133.4484971	-1.8574	0.0016826	0.012744
i2_LQ_YHS_c18645/f1p1/2189	169.0563577	19.4935418	3.1164	0.0016825	0.012744
i1_LQ_YHS_c20204/f1p0/1814	5.580694079	64.9166381	-3.5401	0.0016852	0.01276
i2_LQ_YHS_c24267/f1p2/2471	195.5167309	548.5759033	-1.4884	0.0016849	0.01276
i2_LQ_YHS_c33604/f1p1/2545	0.544682597	16.81224876	-4.948	0.0016854	0.01276
i2_HQ_YHS_c54477/f2p1/2866	97.20585431	278.1825981	-1.5169	0.0016863	0.012764
i4_LQ_YHS_c8707/f1p0/4471	165.9605039	47.78546901	1.7962	0.0016867	0.012765
i1_LQ_YHS_c4476/f1p0/1444	281.4888824	769.6805276	-1.4512	0.0016887	0.012778
i1_LQ_YHS_c41561/f2p6/1841	0	22.50311491	-Inf	0.0016905	0.012789
i3_LQ_YHS_c16709/f1p0/3615	93.25185642	22.89928585	2.0258	0.0016903	0.012789
i2_LQ_YHS_c5951/f1p54/2944	1693.841717	28.62952636	5.8867	0.0016914	0.012793
i2_HQ_YHS_c32556/f2p0/2732	0	12.07778545	-Inf	0.0016934	0.012807
i1_LQ_YHS_c6987/f1p0/1823	290.7885405	809.3399797	-1.4768	0.0016948	0.012815
i3_LQ_YHS_c6232/f1p0/3718	11.66710229	104.7530521	-3.1665	0.0016955	0.012818
i3_LQ_YHS_c11941/f1p8/3144	204.2905958	11.70001917	4.126	0.0016959	0.012819
i1_LQ_YHS_c10058/f1p0/1857	1025.508533	331.3652914	1.6298	0.0016963	0.01282
i2_LQ_YHS_c24158/f1p3/2188	30.4284436	134.9833425	-2.1493	0.0016968	0.012822
i0_LQ_YHS_c1212/f1p5/908	270.9571351	735.0197671	-1.4397	0.0016978	0.012827
i2_LQ_YHS_c25957/f1p0/2468	291.3387461	804.6902983	-1.4657	0.0016991	0.012835
i2_LQ_YHS_c27186/f1p0/2742	0	11.69164612	-Inf	0.0016996	0.012837
i0_LQ_YHS_c1443/f1p2/802	702.8764254	2247.224284	-1.6768	0.0017005	0.01284
i3_HQ_YHS_c21635/f17p3/3262	305.986337	857.3591695	-1.4864	0.0017002	0.01284
i4_LQ_YHS_c2958/f1p5/4708	9.323837147	52.48762569	-2.493	0.0017012	0.012843
i2_LQ_YHS_c39059/f1p2/2533	120.1375378	16.72105663	2.8449	0.0017033	0.012857
i2_LQ_YHS_c45765/f3p1/2072	728.4466344	258.5394517	1.4944	0.0017061	0.012876
i0_LQ_YHS_c2010/f1p1/660	151.7858651	537.2338233	-1.8235	0.0017068	0.01288
i0_LQ_YHS_c1372/f1p0/614	831.5372162	2968.879341	-1.8361	0.0017078	0.012885
i1_LQ_YHS_c28101/f1p0/1319	340.6838759	960.6309707	-1.4955	0.0017091	0.01289

i2_LQ_YHS_c17939/f1p164/2441	8605.083478	74.51952971	6.8514	0.001709	0.01289
i1_LQ_YHS_c13754/f1p0/1147	1227.292361	6.244502185	7.6187	0.0017101	0.012893
i1_LQ_YHS_c36891/f1p2/1399	464.3185511	164.2281159	1.4994	0.0017102	0.012893
i3_LQ_YHS_c11481/f1p1/3212	29.70459722	125.5937574	-2.08	0.00171	0.012893
i2_LQ_YHS_c4517/f1p3/2984	238.5171012	664.5782231	-1.4783	0.0017106	0.012894
i2_LQ_YHS_c14722/f1p0/2053	0	11.64713562	-Inf	0.0017129	0.012909
i1_HQ_YHS_c6775/f2p0/1581	566.1391681	203.4531988	1.4765	0.0017137	0.012913
i0_LQ_YHS_c2511/f1p1/817	0	20.5000236	-Inf	0.0017176	0.01294
i2_LQ_YHS_c51233/f1p5/2495	2121.679741	411.4673551	2.3664	0.0017177	0.01294
i1_LQ_YHS_c27459/f1p0/1347	343.7865093	9.388712142	5.1944	0.0017206	0.012958
i4_HQ_YHS_c2409/f2p0/4488	53.77293298	0	Inf	0.0017206	0.012958
i6_LQ_YHS_c519/f1p0/6197	0	11.61486852	-Inf	0.0017226	0.01297
i1_LQ_YHS_c26590/f1p0/1693	1384.761892	15369.86756	-3.4724	0.0017247	0.012981
i2_LQ_YHS_c21468/f1p0/2912	4.419149489	35.44283234	-3.0037	0.0017248	0.012981
i2_LQ_YHS_c50061/f1p0/2442	0	11.60874682	-Inf	0.0017244	0.012981
i5_LQ_YHS_c4630/f1p0/5046	150.6409576	10.21221578	3.8827	0.0017257	0.012986
i2_LQ_YHS_c19422/f1p2/2673	15.29943617	75.27577481	-2.2987	0.0017291	0.013009
i2_LQ_YHS_c5650/f1p0/2112	1.089365194	47.71216898	-5.4528	0.0017308	0.01302
i2_LQ_YHS_c12389/f1p0/2106	45.64061298	6.970611768	2.711	0.0017322	0.013026
i2_LQ_YHS_c14116/f1p0/2093	51.0099855	0	Inf	0.001732	0.013026
i1_HQ_YHS_c37518/f6p4/1114	18878.56486	1107.883591	4.0909	0.0017334	0.013032
i1_LQ_YHS_c8756/f1p0/1635	73.9259701	338.5153285	-2.1951	0.0017338	0.013032
i3_LQ_YHS_c10193/f1p1/3771	210.6552967	68.84580359	1.6134	0.0017343	0.013032
i3_LQ_YHS_c4198/f1p0/3493	0	11.57647973	-Inf	0.0017343	0.013032
i4_LQ_YHS_c9254/f1p0/4217	123.1843214	11.79962502	3.384	0.0017342	0.013032
i4_LQ_YHS_c6254/f1p1/4958	1.448990031	34.87466087	-4.5891	0.0017369	0.013049
i1_LQ_YHS_c32741/f1p0/2007	238.4033647	59.56388479	2.0009	0.0017374	0.013051
i2_LQ_YHS_c9418/f1p15/2537	1951.127661	620.5122751	1.6528	0.0017394	0.013065
i1_LQ_YHS_c19285/f1p3/1597	141.2055011	42.34559279	1.7375	0.0017411	0.013075
i1_LQ_YHS_c33046/f1p1/1664	34.75824038	372.7887708	-3.4229	0.0017422	0.013081
i3_LQ_YHS_c9059/f1p1/3540	0	11.54421263	-Inf	0.0017441	0.013094
i2_LQ_YHS_c53838/f1p0/2357	0	11.54255407	-Inf	0.0017446	0.013095
i2_LQ_YHS_c5828/f1p0/3000	175.4352042	480.4549819	-1.4535	0.001746	0.013104
i1_LQ_YHS_c26583/f1p19/1987	28.40249352	110.9505183	-1.9658	0.00175	0.013129
i2_LQ_YHS_c33372/f1p22/3003	4242.193545	201.6589124	4.3948	0.0017498	0.013129
i1_LQ_YHS_c22817/f1p0/1784	483.1027755	1469.231139	-1.6047	0.0017527	0.013147
i1_LQ_YHS_c9447/f1p0/1664	1079.568999	27.17640072	5.312	0.0017555	0.013167
i2_LQ_YHS_c21193/f1p2/2318	255.7710728	86.88720644	1.5576	0.0017574	0.013179
i1_LQ_YHS_c12198/f1p0/1759	356.3356847	127.7833044	1.4795	0.0017587	0.013185
i3_LQ_YHS_c13229/f1p3/3643	83.13415724	20.08892444	2.049	0.0017587	0.013185
i3_LQ_YHS_c18356/f1p0/3391	32.29335344	3.344526996	3.2714	0.0017605	0.013196
i2_LQ_YHS_c9970/f1p1/2571	115.1193112	31.12345831	1.8871	0.001766	0.013235
i2_HQ_YHS_c2687/f5p0/2337	122.4008053	348.6372141	-1.5101	0.0017665	0.013237
i4_LQ_YHS_c7084/f1p4/4598	427.6805135	1293.235765	-1.5964	0.00177	0.013261
i2_LQ_YHS_c4040/f1p4/2967	14.90579499	72.05454773	-2.2732	0.0017705	0.013263
i2_LQ_YHS_c64858/f1p6/2012	320.8460513	112.8889161	1.507	0.0017793	0.013327
i1_LQ_YHS_c24973/f1p0/1882	39.28696042	0	Inf	0.001781	0.013337
i1_LQ_YHS_c25924/f1p1/1751	19.25484263	82.148753	-2.093	0.0017829	0.013349
i3_LQ_YHS_c7296/f1p1/3447	167.5272575	468.3633374	-1.4832	0.001785	0.013363
i3_LQ_YHS_c9592/f1p2/3274	89.85451456	255.5810629	-1.5081	0.001786	0.013369
i1_HQ_YHS_c29304/f5p4/1718	604.1667521	1871.358464	-1.6311	0.0017873	0.013376
i2_LQ_YHS_c24091/f1p3/2665	100.5099529	26.6221719	1.9166	0.0017878	0.013378
i2_LQ_YHS_c58301/f1p0/2038	365.9104389	1052.598659	-1.5244	0.0017884	0.01338
i1_LQ_YHS_c20556/f1p649/1968	90.5130413	319.0067421	-1.8174	0.0017902	0.013389
i1_LQ_YHS_c26292/f1p1/1503	195.9864098	529.7271094	-1.4345	0.00179	0.013389
i2_LQ_YHS_c6778/f1p1/2268	35.91692714	2.161622197	4.0545	0.0017924	0.013404
i2_LQ_YHS_c20993/f1p2/2499	7.441770741	61.18577292	-3.0395	0.001793	0.013406
i1_LQ_YHS_c25789/f1p0/1560	30.66089972	112.7438142	-1.8786	0.0017952	0.013421
i4_LQ_YHS_c5047/f1p0/4991	2.789715155	26.6605607	-3.2565	0.0017991	0.013447
i1_LQ_YHS_c33603/f1p2/1750	234.2602808	641.4998739	-1.4533	0.0018053	0.013492
i2_LQ_YHS_c38162/f1p0/2712	165.6590901	438.8195058	-1.4054	0.0018057	0.013493
i2_LQ_YHS_c39792/f1p10/2824	32.53121167	3.184850097	3.3525	0.0018069	0.0135
i5_LQ_YHS_c4183/f1p0/5943	46.92289142	3235.715759	-6.1076	0.0018083	0.013508
i1_LQ_YHS_c22224/f1p1/1939	259.7716134	90.30238933	1.5244	0.0018134	0.013544
i4_HQ_YHS_c14403/f6p0/4696	582.0799002	214.0096085	1.4435	0.0018154	0.013557
i1_HQ_YHS_c10478/f2p0/1887	26.36465254	369.6854574	-3.8096	0.0018179	0.013573
i1_HQ_YHS_c13157/f3p1/2001	199.3840412	49.86355958	1.9995	0.0018189	0.013579
i2_LQ_YHS_c12025/f1p0/2794	150.0846774	27.3199243	2.4578	0.0018197	0.013582
i2_LQ_YHS_c45429/f5p7/2933	366.6951817	1029.431832	-1.4892	0.0018199	0.013582
i1_LQ_YHS_c17262/f2p4/1457	703.894163	13.86551173	5.6658	0.0018212	0.013583
i1_LQ_YHS_c20298/f1p0/1943	0	17.52666817	-Inf	0.0018211	0.013583
i1_LQ_YHS_c32830/f1p0/1502	48.68294346	183.7164089	-1.916	0.0018214	0.013583
i2_LQ_YHS_c6136/f1p7/2523	0	11.29997787	-Inf	0.0018213	0.013583
i4_LQ_YHS_c6613/f1p0/4552	342.6588961	999.1746112	-1.544	0.0018209	0.013583
i2_LQ_YHS_c48398/f2p2/3085	160.1312826	50.12402788	1.6757	0.0018221	0.013586
i2_LQ_YHS_c50596/f1p0/2284	74.48688417	221.9866207	-1.5754	0.0018225	0.013587
i3_LQ_YHS_c4887/f1p1/4023	4.826622662	72.87244335	-3.9163	0.0018243	0.013598
i3_LQ_YHS_c5630/f1p17/3735	65.33693675	13.41543099	2.284	0.0018257	0.013607
i2_LQ_YHS_c41978/f1p0/2200	33.66682276	2.843221275	3.5657	0.0018262	0.013608
i2_LQ_YHS_c20668/f1p2/2205	8.639400842	52.73916771	-2.6099	0.0018305	0.013639
i2_LQ_YHS_c19302/f1p2/2148	141.7979459	388.851318	-1.4554	0.0018344	0.013665
i1_LQ_YHS_c3845/f1p1/1702	432.1736864	153.4122642	1.4942	0.0018359	0.013672
i2_LQ_YHS_c7511/f1p0/2142	188.1182285	515.012854	-1.453	0.0018359	0.013672
i4_LQ_YHS_c4279/f1p0/4789	184.6571815	529.6267598	-1.5201	0.0018373	0.01368
i2_LQ_YHS_c25166/f1p68/2406	8405.363321	653.186441	3.6857	0.0018388	0.013689
i2_HQ_YHS_c2515/f7p1/2253	2469.786744	81.61952356	4.9193	0.0018403	0.013699
i3_LQ_YHS_c4255/f1p1/3081	8.960870994	51.30246954	-2.5173	0.0018409	0.013701
i2_HQ_YHS_c57752/f2p1/2060	222.8902937	35.97640515	2.6312	0.0018414	0.013702
i2_HQ_YHS_c60795/f6p7/2733	344.3862506	49.83804652	2.7887	0.0018425	0.013709

i2_LQ_YHS_c20511/f1p0/2663	209.2661656	39.77938593	2.3952	0.0018436	0.013714
i7_LQ_YHS_c283/f1p0/7602	2.133417961	26.96652508	-3.6599	0.0018438	0.013714
i1_HQ_YHS_c15266/f3p0/1433	582.5501092	198.4779711	1.5534	0.0018472	0.013737
i3_LQ_YHS_c2855/f1p0/3290	6.942867307	46.33048594	-2.7384	0.0018507	0.013761
i3_LQ_YHS_c6368/f1p2/3160	195.766997	63.1344012	1.6326	0.001855	0.013791
i1_LQ_YHS_c8656/f1p1/1627	1587.596444	362.1026055	2.1324	0.0018568	0.013802
i2_LQ_YHS_c41928/f1p23/2311	3.052829538	30.5576184	-3.3233	0.0018582	0.01381
i2_LQ_YHS_c13237/f1p1/2370	56.8772083	1.498388234	5.2464	0.0018606	0.013826
i1_HQ_YHS_c15258/f2p0/1377	673.8627593	73.57315963	3.1952	0.0018619	0.01383
i2_HQ_YHS_c60662/f5p1/2129	111.1822389	360.8922526	-1.6986	0.0018619	0.01383
i3_HQ_YHS_c1671/f3p0/3631	469.1286216	1414.01335	-1.5917	0.0018626	0.01383
i4_LQ_YHS_c7361/f1p1/4831	83.98322759	243.1414261	-1.5336	0.001862	0.01383
i7_LQ_YHS_c250/f1p0/7279	4.945386627	37.5760578	-2.9257	0.0018622	0.01383
i3_LQ_YHS_c15566/f3p0/3587	522.8669112	1602.243506	-1.6156	0.0018636	0.013834
i4_LQ_YHS_c6287/f1p0/4747	0	17.43424981	-Inf	0.0018633	0.013834
i4_LQ_YHS_c12105/f1p0/4440	1.618943649	23.89131244	-3.8834	0.001868	0.013864
i2_LQ_YHS_c36507/f1p1/2807	21.80081396	89.46608905	-2.037	0.0018689	0.013869
i4_LQ_YHS_c7691/f1p0/4479	151.251599	48.07978388	1.6534	0.0018707	0.01388
i1_HQ_YHS_c5701/f2p0/1804	198.3188707	532.2657458	-1.4243	0.0018731	0.013896
i2_LQ_YHS_c15591/f17p2/2539	243.9732989	674.9424085	-1.468	0.0018735	0.013897
i3_LQ_YHS_c18789/f1p29/3333	109.4073795	317.6296001	-1.5376	0.0018742	0.013899
i3_LQ_YHS_c20265/f2p0/3143	48.45545623	332.4332671	-2.7783	0.0018761	0.013911
i4_LQ_YHS_c2683/f1p0/4667	100.8580422	25.96225507	1.9578	0.0018787	0.013928
i2_LQ_YHS_c52338/f1p3/2249	38.23158643	134.0000026	-1.8094	0.0018794	0.01393
i3_HQ_YHS_c2195/f2p1/3446	150.4907587	44.82210564	1.7474	0.0018792	0.01393
i3_LQ_YHS_c17566/f1p0/3366	25.9455446	109.7159957	-2.0802	0.0018805	0.013936
i2_LQ_YHS_c40404/f1p0/2074	180.4018953	17.59171491	3.3582	0.0018814	0.01394
i1_LQ_YHS_c12033/f1p3/1925	138.4700453	11.6154613	3.5755	0.0018841	0.013955
i2_LQ_YHS_c32951/f1p0/2145	0	35.03429822	-Inf	0.001884	0.013955
i2_LQ_YHS_c48651/f1p1/2951	634.168759	38.593164	4.0384	0.0018848	0.013959
i2_LQ_YHS_c54767/f1p2/2526	283.01487	1455.955639	-2.363	0.0018861	0.013966
i4_LQ_YHS_c12392/f1p2/4291	32.42928235	122.8272346	-1.9213	0.0018864	0.013967
i2_LQ_YHS_c5818/f1p4/2387	0	11.1002536	-Inf	0.0018876	0.013973
i3_LQ_YHS_c9150/f1p0/3453	333.962913	36.72028707	3.185	0.0018904	0.013992
i1_LQ_YHS_c32338/f1p1/1669	469.1011455	170.1738005	1.4629	0.001891	0.013994
i1_LQ_YHS_c10774/f1p2/1753	83.53948342	1.415488932	5.8831	0.0018951	0.014022
i2_LQ_YHS_c3765/f1p10/2752	531.4805832	71.70403218	2.8899	0.0018974	0.014037
i3_LQ_YHS_c6766/f1p0/3228	29.43987203	3.138088251	3.2298	0.0018998	0.01405
i5_LQ_YHS_c3040/f1p5/5252	65.98496562	6.778074999	3.2832	0.0018997	0.01405
i2_LQ_YHS_c22750/f1p3/2164	15.8764046	70.88459888	-2.1586	0.0019031	0.014073
i2_LQ_YHS_c49223/f1p0/2421	63.82119456	196.0097918	-1.6188	0.0019085	0.014111
i1_LQ_YHS_c11859/f1p9/1849	244.011712	0	Inf	0.0019142	0.014151
i2_LQ_YHS_c21113/f1p2/2784	61.72466752	187.7575423	-1.605	0.0019156	0.014159
i1_LQ_YHS_c39537/f1p3/1094	0.631966135	16.37994037	-4.6959	0.0019182	0.014176
i1_LQ_YHS_c33242/f1p2/1471	378.1768014	1044.545888	-1.4657	0.0019192	0.01418
i1_LQ_YHS_c37952/f2p0/1349	1343.29371	6540.609563	-2.2837	0.0019194	0.01418
i4_LQ_YHS_c13701/f1p0/4129	94.51979898	268.6396578	-1.507	0.0019212	0.014191
i1_LQ_YHS_c16870/f1p0/1714	966.1791818	3620.092445	-1.9057	0.0019253	0.014219
i1_LQ_YHS_c39172/f1p0/1208	211.9488952	597.8193945	-1.496	0.0019262	0.014224
i3_LQ_YHS_c3825/f1p0/3121	320.1646474	112.8538444	1.5044	0.0019271	0.014229
i2_LQ_YHS_c23634/f1p0/2390	25.34298135	434.2511658	-4.0989	0.001929	0.014236
i2_LQ_YHS_c51157/f1p1/2851	50.36278716	8.763866978	2.5227	0.001929	0.014236
i3_LQ_YHS_c12313/f1p0/3425	198.9813742	622.5873276	-1.6456	0.0019289	0.014236
i2_HQ_YHS_c45746/f2p1/2128	170.617325	464.3791557	-1.4445	0.0019314	0.014251
i3_LQ_YHS_c9562/f1p3/3837	303.3395648	64.31789878	2.2376	0.0019341	0.014269
i2_LQ_YHS_c52537/f1p1/2638	291.4660237	3.599939384	6.3392	0.0019352	0.014275
i2_HQ_YHS_c45408/f3p2/2598	121.2396161	328.6372354	-1.4386	0.0019378	0.014292
i5_LQ_YHS_c2510/f1p4/5496	17.4597211	80.01608076	-2.1963	0.0019407	0.014311
i0_LQ_YHS_c892/f1p0/678	305.8227153	827.2740612	-1.4357	0.0019475	0.014357
i3_LQ_YHS_c13302/f1p0/3700	49.81141356	8.442854614	2.5607	0.0019475	0.014357
i1_HQ_YHS_c2595/f2p0/1840	179.9444711	57.12967065	1.6552	0.0019626	0.014464
i1_LQ_YHS_c4422/f2p7/1811	109.6781435	21.41819694	2.3564	0.0019627	0.014464
i2_LQ_YHS_c4660/f1p2/2168	60.18758854	186.3574942	-1.6305	0.0019629	0.014464
i3_LQ_YHS_c13300/f1p1/3737	652.2794387	214.5376919	1.6043	0.0019644	0.014473
i4_LQ_YHS_c15775/f1p0/4015	0.544682597	29.87202919	-5.7772	0.0019696	0.014509
i2_LQ_YHS_c12164/f1p29/2390	3669.043097	90.26178873	5.3451	0.0019726	0.014529
i2_HQ_YHS_c12613/f2p0/2110	88.17117636	258.7787492	-1.5533	0.0019759	0.014551
i3_LQ_YHS_c10888/f1p8/3355	27.74032747	2.598393732	3.4163	0.001977	0.014557
i2_LQ_YHS_c54719/f1p2/2335	148.6651728	397.0876224	-1.4174	0.0019785	0.014565
i3_LQ_YHS_c3827/f1p0/3710	302.4755591	107.8001471	1.4885	0.0019827	0.014595
i1_LQ_YHS_c14212/f1p3/1352	102.1993705	11.27217391	3.1805	0.001984	0.014602
i2_LQ_YHS_c40132/f1p2/2249	965.3366301	24.19455247	5.3183	0.0019854	0.014608
i2_LQ_YHS_c41684/f1p1/2728	65.48223739	199.2889176	-1.6057	0.0019851	0.014608
i3_LQ_YHS_c4640/f1p0/3251	2.395268576	34.85143982	-3.863	0.0019876	0.014621
i2_LQ_YHS_c40112/f1p0/2335	0	17.0725973	-Inf	0.0019897	0.014635
i2_LQ_YHS_c48464/f2p2/2131	696.7465515	250.4583287	1.4761	0.0019907	0.01464
i0_LQ_YHS_c2913/f1p3/991	266.3707043	705.8802076	-1.406	0.0019925	0.014651
i0_LQ_YHS_c1037/f1p0/898	642.3441977	163.6021642	1.9732	0.0019953	0.014667
i3_LQ_YHS_c14158/f1p1/3023	1108.747752	358.1841435	1.6302	0.0019951	0.014667
i0_LQ_YHS_c1737/f1p0/591	5489.590818	41.97157711	7.0311	0.0019979	0.014682
i3_HQ_YHS_c15703/f3p0/3081	385.1985775	1160.213062	-1.5907	0.0019976	0.014682
i3_LQ_YHS_c14277/f1p0/3086	2472.881982	741.8115464	1.7371	0.0019997	0.014691
i3_LQ_YHS_c4897/f1p3/3823	30.49429579	109.3673733	-1.8426	0.0019998	0.014691
i1_LQ_YHS_c32932/f1p0/1628	119.8422657	34.28603338	1.8054	0.0020012	0.014699
i1_HQ_YHS_c27003/f5p1/1237	1510.222507	13476.20275	-3.1576	0.0020024	0.014706
i2_LQ_YHS_c15573/f7p1/2579	781.9090761	254.3827569	1.62	0.0020083	0.014747
i1_LQ_YHS_c27449/f1p0/1324	2417.695637	657.0352151	1.8796	0.0020129	0.014778
i2_LQ_YHS_c8020/f1p5/2185	90.26104589	258.0124736	-1.5153	0.0020153	0.014794
i3_LQ_YHS_c9114/f1p4/3667	27.09150994	195.1044607	-2.8483	0.0020163	0.014799

i2_LQ_YHS_c5258/f1p2/2838	148.4149941	30.51030332	2.2823	0.0020186	0.014814
i2_LQ_YHS_c44408/f1p0/2023	0	11.30550679	-Inf	0.0020192	0.014815
i4_LQ_YHS_c5615/f1p0/4227	98.16065731	284.7963007	-1.5367	0.0020201	0.01482
i2_LQ_YHS_c25433/f1p23/3357	1731.689793	149.2781581	3.5361	0.0020214	0.014827
i2_LQ_YHS_c12390/f1p1/2406	547.4237471	200.2217401	1.4511	0.0020238	0.014842
i5_LQ_YHS_c2580/f1p2/5878	180.1513157	57.88579598	1.6379	0.0020241	0.014842
i1_LQ_YHS_c3147/f1p0/1658	34.19640122	3.755153143	3.1869	0.0020278	0.014867
i5_LQ_YHS_c3538/f1p0/5539	1.388573461	60.45705763	-5.4442	0.0020282	0.014868
i1_HQ_YHS_c2987/f5p3/1668	243.2828573	656.7803548	-1.4328	0.0020301	0.014877
i1_LQ_YHS_c11813/f1p4/1626	244.7576181	892.8658603	-1.8671	0.0020297	0.014877
i2_LQ_YHS_c51887/f1p0/2972	0	11.27158114	-Inf	0.0020312	0.014879
i3_HQ_YHS_c12521/f3p0/3442	0	11.2732397	-Inf	0.0020306	0.014879
i4_LQ_YHS_c4547/f1p7/4889	0	11.27158114	-Inf	0.0020312	0.014879
i2_LQ_YHS_c21584/f1p2/2384	134.5783632	374.963851	-1.4783	0.0020318	0.014881
i0_LQ_YHS_c885/f1p0/658	163.6165854	441.7006429	-1.4327	0.0020372	0.014918
i2_LQ_YHS_c40226/f1p0/2939	97.284039	277.7991831	-1.5138	0.0020393	0.014931
i3_LQ_YHS_c14010/f1p0/3017	4333.4916	202.5390502	4.4193	0.00204	0.014935
i2_LQ_YHS_c40571/f1p0/2840	0.272341299	14.15931291	-5.7002	0.002042	0.014946
i2_HQ_YHS_c5229/f2p0/2795	401.5922556	140.3106174	1.5171	0.0020472	0.01498
i3_LQ_YHS_c22570/f1p13/3171	59.7335308	183.0541594	-1.6157	0.002047	0.01498
i2_LQ_YHS_c20902/f1p13/2357	0	89.46585064	-Inf	0.0020481	0.014985
i2_HQ_YHS_c17861/f7p3/2596	477.5530867	1446.021168	-1.5984	0.00205	0.014996
i0_LQ_YHS_c3163/f1p1/928	638.2546156	2100.588912	-1.7186	0.0020508	0.015
i2_LQ_YHS_c44539/f1p0/2054	0	11.21482722	-Inf	0.0020515	0.015002
i1_LQ_YHS_c7084/f1p4/1747	99.96147059	26.67777981	1.9057	0.0020558	0.01503
i2_LQ_YHS_c19900/f1p0/2313	0	11.20258381	-Inf	0.0020559	0.01503
i2_LQ_YHS_c25523/f1p4/2944	16.37323048	74.48224738	-2.1856	0.0020585	0.015047
i1_LQ_YHS_c8168/f1p0/1497	403.8653447	44.39192882	3.1855	0.0020611	0.015064
i2_HQ_YHS_c11881/f3p1/2590	331.6601602	913.0689671	-1.461	0.002063	0.015074
i2_LQ_YHS_c20359/f1p52/2611	5.71916727	152.4020664	-4.7359	0.0020631	0.015074
i1_LQ_YHS_c6158/f1p1/1962	497.9046892	182.3514325	1.4491	0.0020642	0.015075
i3_LQ_YHS_c14349/f1p3/3045	548.4571907	1718.056744	-1.6473	0.0020637	0.015075
i3_LQ_YHS_c18135/f1p1/3275	0	11.356139	-Inf	0.0020642	0.015075
i9_LQ_YHS_c32/f1p0/9227	6.250017867	2379.924689	-8.5728	0.002069	0.015108
i3_LQ_YHS_c14271/f1p1/3085	0	11.16253645	-Inf	0.0020704	0.015113
i4_LQ_YHS_c9165/f1p5/4636	17.37243756	78.05880528	-2.1678	0.0020704	0.015113
i1_LQ_YHS_c6838/f1p5/1769	14257.46728	0	Inf	0.0020709	0.015115
i2_LQ_YHS_c39801/f1p1/2591	52.6265871	167.1386748	-1.6672	0.0020741	0.015136
i2_LQ_YHS_c3098/f1p9/2374	35.36590895	0	Inf	0.0020766	0.015152
i4_LQ_YHS_c12723/f1p1/4416	128.2301127	37.360773	1.7791	0.0020813	0.015183
i3_LQ_YHS_c3897/f1p1/3155	39.95372317	136.2049498	-1.7694	0.0020876	0.015227
i2_LQ_YHS_c19902/f1p0/2663	0	11.1135628	-Inf	0.0020883	0.01523
i5_LQ_YHS_c3388/f1p1/5554	7.174042901	44.74911819	-2.641	0.0020909	0.015247
i1_LQ_YHS_c24100/f1p0/1487	3650.933152	950.6061198	1.9413	0.002092	0.015251
i2_LQ_YHS_c38985/f1p3/2745	34.32438372	4.218070066	3.0246	0.0020921	0.015251
i2_LQ_YHS_c35506/f1p0/2575	64.83311468	0.707744466	6.5174	0.002093	0.015256
i1_HQ_YHS_c17049/f2p0/1424	3195.101425	63.03088544	5.6637	0.0020974	0.015285
i1_LQ_YHS_c25907/f1p9/1811	0	11.08741742	-Inf	0.002098	0.015287
i2_LQ_YHS_c51655/f1p1/2619	70.70443396	220.3316403	-1.6398	0.0021007	0.015305
i1_LQ_YHS_c31955/f1p0/1563	54.92027341	170.8338371	-1.6372	0.002103	0.015319
i2_LQ_YHS_c38244/f1p4/2581	143.0674	398.5191772	-1.478	0.0021039	0.015321
i3_LQ_YHS_c18999/f1p6/3465	60.8267208	12.00547099	2.341	0.0021039	0.015321
i1_LQ_YHS_c24686/f1p6/1515	202.5782676	541.6590804	-1.4189	0.0021081	0.015349
i1_HQ_YHS_c17273/f2p2/1864	8.236541127	147.8618022	-4.1661	0.0021123	0.015366
i1_LQ_YHS_c25470/f1p3/2004	147.3821177	44.33636046	1.733	0.0021118	0.015366
i2_LQ_YHS_c9452/f2p3/2233	174.8773095	464.0635118	-1.408	0.0021117	0.015366
i4_LQ_YHS_c4619/f1p0/4768	0	11.04902862	-Inf	0.0021122	0.015366
i5_LQ_YHS_c3134/f1p2/5813	151.9093873	409.7759175	-1.4316	0.0021114	0.015366
i7_LQ_YHS_c127/f1p0/7444	305.0558196	847.1161056	-1.4735	0.002111	0.015366
i3_LQ_YHS_c4765/f1p0/3330	193.8388044	522.036239	-1.4293	0.002117	0.015397
i1_HQ_YHS_c17045/f2p0/1678	456.2880576	7.93874432	5.8449	0.0021181	0.015403
i2_HQ_YHS_c61218/f3p0/2422	288.8552844	78.72140691	1.8755	0.002119	0.015407
i3_LQ_YHS_c19454/f1p0/3571	29.38071922	111.5642265	-1.9249	0.0021225	0.01543
i1_HQ_YHS_c40150/f3p0/1404	354.9499893	991.4346383	-1.4819	0.0021237	0.015437
i1_LQ_YHS_c28999/f1p0/1255	293.9540306	85.19794	1.7867	0.0021264	0.015454
i0_LQ_YHS_c1577/f1p0/600	128.512247	339.0438476	-1.3996	0.0021289	0.01547
i2_LQ_YHS_c5491/f2p1/2483	27.88501656	187.5227392	-2.7495	0.0021303	0.015478
i2_LQ_YHS_c59596/f1p1/2039	23.97827221	214.6772588	-3.1624	0.0021361	0.015518
i0_LQ_YHS_c1216/f1p0/876	180.7112963	483.5254941	-1.4199	0.0021412	0.015553
i3_LQ_YHS_c5595/f1p0/3100	65.60720888	200.9914142	-1.6152	0.0021421	0.015557
i2_LQ_YHS_c37833/f1p0/2393	0	21.26452198	-Inf	0.0021434	0.015564
i2_LQ_YHS_c21295/f1p0/2185	36.47115858	4.67545806	2.9636	0.0021462	0.015582
i2_HQ_YHS_c4651/f2p0/2334	163.1560521	6.08423251	4.745	0.0021473	0.015587
i2_HQ_YHS_c10674/f2p3/2581	110.5859945	308.6173896	-1.4807	0.0021487	0.015595
i1_HQ_YHS_c12229/f2p5/1831	433.871552	1260.8405	-1.539	0.0021496	0.015598
i1_HQ_YHS_c37581/f5p1/1105	5689.408111	1212.802371	2.2299	0.0021497	0.015598
i1_LQ_YHS_c7745/f1p1/1702	277.523349	95.70833989	1.5359	0.0021502	0.015599
i3_LQ_YHS_c3391/f1p0/3131	12.7270646	63.31410178	-2.3146	0.0021523	0.015612
i1_LQ_YHS_c33432/f1p2/1463	637.869001	220.3028843	1.5338	0.0021588	0.015652
i1_LQ_YHS_c9859/f1p1/2025	1176.287039	268.5125677	2.1312	0.0021584	0.015652
i3_LQ_YHS_c18293/f1p6/3077	243.7145251	83.7229728	1.5415	0.0021586	0.015652
i2_HQ_YHS_c32373/f2p6/2676	112.120674	30.64442761	1.8714	0.0021611	0.015665
i2_LQ_YHS_c55650/f1p10/2713	209.7963101	70.29130827	1.5776	0.002161	0.015665
i2_LQ_YHS_c38637/f1p1/2169	145.0664998	391.939174	-1.4339	0.002162	0.015669
i4_LQ_YHS_c13380/f1p0/4117	30.57108027	109.5694687	-1.8416	0.0021634	0.015676
i2_LQ_YHS_c34770/f1p2/2265	509.9292598	1493.89401	-1.5507	0.0021662	0.015694
i2_LQ_YHS_c37446/f1p1/2542	856.7880792	85.21306708	3.3298	0.0021679	0.015704
i4_HQ_YHS_c2559/f2p0/4592	97.23237423	275.0735796	-1.5003	0.0021701	0.015718
i3_HQ_YHS_c15549/f4p0/3210	775.1963876	2696.971901	-1.7987	0.0021713	0.015724

i2_LQ_YHS_c20441/f1p213/2582	280.8976715	7466.539628	-4.7323	0.0021728	0.015732
i1_LQ_YHS_c22200/f1p2/1879	33.47829562	2.123233398	3.9789	0.0021744	0.015742
i1_LQ_YHS_c6594/f1p0/1949	12.60035424	61.47408473	-2.2865	0.0021757	0.015749
i1_LQ_YHS_c42143/f1p0/1175	189.0007827	61.74387211	1.614	0.0021764	0.015752
i3_LQ_YHS_c14743/f1p0/3033	423.5639817	81.2986705	2.3813	0.0021791	0.015768
i2_LQ_YHS_c55908/f1p8/2440	1.603839507	29.38296688	-4.1954	0.0021795	0.015769
i2_HQ_YHS_c8493/f5p6/2201	477.4118347	1418.440941	-1.571	0.0021811	0.015779
i3_LQ_YHS_c6940/f1p176/3539	142.1748805	42.52083023	1.7414	0.0021822	0.015784
i1_LQ_YHS_c4864/f1p1/1909	164.3169145	16.48286335	3.3174	0.0021835	0.015791
i1_LQ_YHS_c34614/f1p1/1471	898.4304889	3326.684489	-1.8886	0.0021857	0.015804
i2_LQ_YHS_c41897/f1p4/2914	112.4737849	375.4616863	-1.7391	0.0021869	0.015811
i2_HQ_YHS_c23540/f2p4/2531	156.5793139	49.7162854	1.6551	0.0021901	0.015832
i5_LQ_YHS_c1745/f1p0/5739	162.4295657	453.5070615	-1.4813	0.0021929	0.01585
i1_LQ_YHS_c27856/f1p0/1313	88.26353172	272.9968696	-1.629	0.002194	0.015855
i1_LQ_YHS_c21581/f1p1/1382	126.8582087	1218.133387	-3.2634	0.0021958	0.015865
i1_LQ_YHS_c12153/f1p0/1644	169.0337452	27.72383596	2.6081	0.0021978	0.015878
i2_LQ_YHS_c21696/f1p23/2188	716.5055441	2352.134155	-1.7149	0.0021989	0.015883
i2_HQ_YHS_c45337/f3p1/2129	523.2129326	189.5767825	1.4646	0.0022043	0.015918
i2_LQ_YHS_c51502/f1p4/2543	3.084309964	28.94453678	-3.2303	0.0022047	0.015918
i4_LQ_YHS_c4944/f1p0/4761	20.68538721	83.10424934	-2.0063	0.0022044	0.015918
i1_LQ_YHS_c3403/f1p2/2004	109.263071	15.7105847	2.798	0.0022053	0.01592
i2_LQ_YHS_c35096/f1p1/2087	113.671263	29.44422348	1.9488	0.0022099	0.015951
i2_LQ_YHS_c33895/f1p1/2687	110.3723225	313.1968331	-1.5047	0.0022154	0.015988
i2_LQ_YHS_c44671/f1p5/1769	0	10.77864846	-Inf	0.0022161	0.015991
i2_LQ_YHS_c13328/f1p5/2444	5.098963961	38.5859765	-2.9198	0.0022195	0.016013
i3_HQ_YHS_c7016/f2p0/3620	0	62.29343893	-Inf	0.0022208	0.01602
i2_LQ_YHS_c7390/f1p1/2860	129.3614239	360.7502761	-1.4796	0.0022241	0.016041
i2_HQ_YHS_c60281/f3p0/2081	126.5011821	33.51154293	1.9164	0.0022267	0.016058
i4_LQ_YHS_c14004/f1p0/4150	10.50174965	57.17749828	-2.4448	0.0022307	0.016084
i3_LQ_YHS_c8811/f1p3/3400	61.49144787	3.376794089	4.1867	0.002232	0.016092
i3_LQ_YHS_c5234/f1p0/3210	32.77761947	2.893853484	3.5016	0.0022331	0.016097
i3_LQ_YHS_c13711/f1p0/3206	42.98824369	0	Inf	0.0022366	0.01612
i2_LQ_YHS_c21421/f1p0/2389	2.647892274	103.666241	-5.291	0.0022399	0.016141
i3_LQ_YHS_c2108/f2p4/3672	4.599593791	75.01562021	-4.0276	0.0022409	0.016146
i1_LQ_YHS_c19387/f1p735/1813	380.945911	136.4878931	1.4808	0.0022425	0.016155
i1_LQ_YHS_c19586/f1p1/1453	1001.984939	338.8209012	1.5643	0.002244	0.016163
i1_LQ_YHS_c5853/f1p0/1948	41.0968223	138.2663734	-1.7504	0.0022525	0.01622
i2_LQ_YHS_c59141/f1p2/2062	68.05863598	200.0837455	-1.5558	0.0022522	0.01622
i1_LQ_YHS_c33027/f1p0/1969	61.43389751	180.6325024	-1.556	0.0022532	0.016222
i1_LQ_YHS_c24754/f1p0/1825	114.7686081	3.292236221	5.1235	0.0022568	0.016244
i4_LQ_YHS_c6159/f1p0/4467	8.140497407	50.5641561	-2.6349	0.0022569	0.016244
i2_LQ_YHS_c3729/f1p1/2723	93.14278611	24.38649646	1.9334	0.0022598	0.016262
i1_LQ_YHS_c8657/f1p0/1749	73.202904	216.2961485	-1.563	0.0022628	0.016282
i4_LQ_YHS_c13329/f1p2/4030	46.35467952	1140.390166	-4.6207	0.0022705	0.016334
i2_HQ_YHS_c33822/f2p1/2620	209.3807745	32.86331628	2.6716	0.0022723	0.016345
i1_LQ_YHS_c17600/f1p4/1821	0	38.86788753	-Inf	0.0022737	0.016351
i3_LQ_YHS_c12239/f1p0/3075	9.701102035	68.90105825	-2.8283	0.0022737	0.016351
i1_LQ_YHS_c25717/f1p0/1556	205.1174737	550.8189255	-1.4251	0.0022749	0.016354
i3_LQ_YHS_c3110/f1p3/3275	986.1603779	249.2548086	1.9842	0.0022746	0.016354
i1_HQ_YHS_c17192/f2p0/1971	564.1369267	206.5195978	1.4498	0.0022813	0.016398
i2_LQ_YHS_c8542/f1p5/2128	16.75303966	73.9559016	-2.1422	0.002283	0.016407
i1_HQ_YHS_c16868/f5p0/1691	4.085119479	506.3481203	-6.9536	0.0022845	0.016416
i4_LQ_YHS_c11783/f1p0/4474	14.48655899	66.96747795	-2.2087	0.0022849	0.016416
i2_HQ_YHS_c4533/f2p1/2349	17.74843868	116.6383403	-2.7163	0.0022853	0.016417
i1_LQ_YHS_c21410/f1p6/1596	4741.120252	1149.184044	2.0446	0.002287	0.016427
i6_LQ_YHS_c962/f1p0/6792	2.168239704	38.70272125	-4.1578	0.0022875	0.016427
i2_HQ_YHS_c42332/f5p4/2154	645.7928179	2028.121364	-1.651	0.0022886	0.016433
i4_LQ_YHS_c10076/f1p3/4950	14.66953921	70.70584433	-2.269	0.0022897	0.016438
i4_LQ_YHS_c14001/f1p0/4078	15.46558173	71.4964881	-2.2088	0.0022902	0.01644
i1_LQ_YHS_c24707/f1p1/1998	107.2033152	28.41321531	1.9157	0.0023007	0.016512
i1_HQ_YHS_c5317/f3p2/1648	149.3306801	45.59438429	1.7116	0.0023039	0.016533
i2_LQ_YHS_c56385/f1p3/2622	31.16914138	121.8485974	-1.9669	0.0023046	0.016536
i2_LQ_YHS_c5740/f1p3/2254	286.941837	60.5555186	2.2444	0.0023087	0.016563
i3_LQ_YHS_c4727/f1p0/3506	251.2553159	933.7551658	-1.8939	0.0023167	0.016617
i3_LQ_YHS_c5120/f1p1/3152	200.2185771	66.57233213	1.5886	0.002317	0.016617
i1_LQ_YHS_c13020/f1p4/1988	15.60867676	68.30670185	-2.1297	0.0023183	0.016624
i1_LQ_YHS_c4472/f1p0/1427	198.7133711	3.730666322	5.7351	0.0023229	0.01665
i2_LQ_YHS_c10365/f1p59/2208	24704.58767	702.6269568	5.1359	0.0023228	0.01665
i2_LQ_YHS_c8770/f1p3/2685	248.4310866	86.96722095	1.5143	0.002323	0.01665
i2_LQ_YHS_c15197/f1p1/2024	741.4100051	2430.8243	-1.7131	0.0023244	0.016658
i2_LQ_YHS_c5298/f1p1/2366	671.2875754	2116.065134	-1.6564	0.0023258	0.016665
i1_HQ_YHS_c11207/f4p9/1867	32.47792772	217.8785496	-2.746	0.0023281	0.01668
i2_LQ_YHS_c52026/f1p0/2345	110.4031746	420.9653526	-1.9309	0.0023316	0.016702
i2_LQ_YHS_c4014/f1p0/2649	23.2838287	0.353872233	6.04	0.0023331	0.016711
i2_LQ_YHS_c3094/f1p3/2154	14.83822906	68.64556677	-2.2098	0.0023347	0.016719
i3_LQ_YHS_c16912/f1p0/3110	88.41152145	10.99673784	3.0072	0.0023406	0.016759
i0_LQ_YHS_c1557/f1p0/695	404.261913	131.6508996	1.6186	0.0023429	0.016768
i1_LQ_YHS_c19116/f1p0/2099	39.41954801	140.0002689	-1.8284	0.0023424	0.016768
i2_LQ_YHS_c5565/f1p0/2285	0	19.36940958	-Inf	0.0023429	0.016768
i3_LQ_YHS_c4434/f1p0/3153	0	14.03131033	-Inf	0.0023587	0.016879
i3_LQ_YHS_c13535/f1p0/3259	83.78918229	20.66538874	2.0195	0.00236	0.016886
i2_LQ_YHS_c13308/f2p1/2875	754.0833154	108.0386925	2.8032	0.0023607	0.016888
i1_HQ_YHS_c33951/f2p0/1975	1765.351677	200.7182283	3.1367	0.002362	0.016895
i2_HQ_YHS_c2235/f6p3/2770	117.6002442	321.3084091	-1.4501	0.0023655	0.016915
i2_LQ_YHS_c22735/f1p3/2611	763.0449524	286.0424344	1.4155	0.0023655	0.016915
i2_LQ_YHS_c18564/f1p3/2762	26.7411204	100.282021	-1.9069	0.0023664	0.016919
i0_HQ_YHS_c128/f7p0/661	1397.515336	6847.965632	-2.2928	0.0023688	0.016933
i1_HQ_YHS_c5040/f2p0/1550	1247.234011	432.795185	1.527	0.0023777	0.016995
i0_LQ_YHS_c3466/f1p1/309	0	28.91945718	-Inf	0.0023835	0.017034

i1_LQ_YHS_c38498/f1p0/1186	228.151144	612.3654384	-1.4244	0.0023859	0.017048
i0_HQ_YHS_c444/f2p0/788	2836.532401	817.8522508	1.7942	0.0023866	0.01705
i2_LQ_YHS_c51775/f1p10/3004	388.3958017	91.25444765	2.0896	0.0023881	0.017056
i4_LQ_YHS_c10537/f1p0/4523	0	10.95163456	-Inf	0.0023877	0.017056
i6_LQ_YHS_c382/f1p0/6167	73.78841363	219.2006263	-1.5708	0.0023905	0.017071
i1_LQ_YHS_c7418/f1p11/1529	1045.488176	376.6997211	1.4727	0.0023945	0.017097
i0_LQ_YHS_c1709/f1p0/904	336.42131	943.3613328	-1.4875	0.0023954	0.017099
i3_LQ_YHS_c9838/f1p0/3403	439.034378	1269.955938	-1.5324	0.0023955	0.017099
i1_LQ_YHS_c35934/f1p3/1713	40.22527581	3.696740663	3.4438	0.0024024	0.017146
i1_LQ_YHS_c5927/f1p5/1424	227.1003919	612.2911121	-1.4309	0.0024035	0.01715
i2_LQ_YHS_c25579/f1p21/2882	1032.352009	3986.476638	-1.9492	0.0024038	0.01715
i1_LQ_YHS_c4084/f1p1/1689	637.3616343	69.32827118	3.2006	0.0024062	0.017165
i4_LQ_YHS_c7552/f1p2/4714	31.1615504	3.209336919	3.2794	0.0024089	0.017182
i1_LQ_YHS_c18892/f1p0/1932	81.51549119	0	Inf	0.0024121	0.017202
i2_LQ_YHS_c5357/f1p0/2290	171.5918707	460.4122672	-1.4239	0.0024127	0.017204
i3_LQ_YHS_c14262/f1p1/3016	70.50775819	286.6432707	-2.0234	0.0024139	0.01721
i2_LQ_YHS_c19148/f1p0/2819	0	10.88710038	-Inf	0.0024146	0.017212
i1_LQ_YHS_c8079/f1p0/1839	10.50302179	55.66967121	-2.4061	0.0024233	0.01727
i2_LQ_YHS_c10384/f1p0/2680	0	12.07166374	-Inf	0.0024231	0.01727
i4_LQ_YHS_c12064/f1p3/4393	1.146440448	19.71609432	-4.1041	0.0024257	0.017284
i1_HQ_YHS_c2523/f6p1/1960	1.074261052	19.77616536	-4.2023	0.0024285	0.017301
i2_LQ_YHS_c53326/f1p0/2917	0	10.84871158	-Inf	0.0024308	0.017315
i2_LQ_YHS_c43912/f1p3/2066	49.29025661	8.219709319	2.5841	0.0024333	0.01733
i4_LQ_YHS_c11769/f1p0/4906	164.5992056	451.023198	-1.4542	0.0024349	0.017339
i2_LQ_YHS_c24744/f1p4/2952	123.0990484	338.7995722	-1.4606	0.0024359	0.017344
i2_LQ_YHS_c34992/f1p0/2298	170.1305602	56.38586894	1.5932	0.0024367	0.017347
i2_LQ_YHS_c64747/f1p1/2008	195.8568499	521.8404943	-1.4138	0.002438	0.017351
i3_LQ_YHS_c18542/f1p2/3199	801.2875991	2648.028757	-1.7245	0.002438	0.017351
i0_HQ_YHS_c289/f3p0/1016	3576.001117	959.0150487	1.8987	0.0024391	0.017356
i2_HQ_YHS_c2564/f5p5/2627	653.6520128	242.5634219	1.4302	0.0024403	0.017362
i2_LQ_YHS_c41983/f1p0/2353	0	10.81810305	-Inf	0.0024438	0.017385
i1_LQ_YHS_c9657/f1p12/1535	1136.905376	4627.001145	-2.025	0.0024466	0.017402
i1_LQ_YHS_c20452/f1p6/1860	1824.739995	347.6388719	2.392	0.0024497	0.017419
i5_LQ_YHS_c2289/f1p1/5224	0	16.77279418	-Inf	0.0024497	0.017419
i3_LQ_YHS_c10869/f1p42/3265	83.40559019	20.91290109	1.9958	0.0024507	0.017424
i2_LQ_YHS_c58827/f1p3/2069	165.9060508	448.4477501	-1.4346	0.0024539	0.017444
i2_LQ_YHS_c22622/f1p2/2988	4.978936228	36.63959957	-2.8795	0.0024556	0.017451
i2_LQ_YHS_c26578/f1p0/2392	0	10.7902991	-Inf	0.0024557	0.017451
i1_LQ_YHS_c36275/f1p0/1844	5.19005555	39.86615559	-2.9413	0.0024591	0.017473
i2_LQ_YHS_c49494/f1p28/2834	481.8207448	10.07983028	5.579	0.0024597	0.017475
i2_LQ_YHS_c40763/f1p1/2568	634.6451348	235.1466404	1.4324	0.0024603	0.017476
i1_LQ_YHS_c18234/f1p9/1774	18.84401977	79.32452916	-2.0737	0.0024616	0.017483
i1_LQ_YHS_c24536/f1p3/1828	0	10.76581228	-Inf	0.0024662	0.017513
i5_LQ_YHS_c4368/f1p4/5191	448.5841585	169.8347367	1.4012	0.0024667	0.017514
i1_HQ_YHS_c1867/f4p1/1531	336.3836316	98.78521109	1.7677	0.0024701	0.017534
i1_LQ_YHS_c35216/f1p4/1627	3.889571034	31.75774231	-3.0294	0.0024702	0.017534
i1_LQ_YHS_c23965/f1p1/1780	404.1358526	47.04036199	3.1029	0.0024714	0.01754
i0_LQ_YHS_c2539/f1p0/605	6595.368815	442.8132857	3.8967	0.002473	0.017546
i2_LQ_YHS_c50225/f1p2/2894	58.41232942	0.353872233	7.3669	0.0024731	0.017546
i2_LQ_YHS_c39276/f1p8/2548	108.4733771	344.7185544	-1.6681	0.0024766	0.017564
i3_LQ_YHS_c19204/f1p3/3966	42.511796	6.655128333	2.6753	0.0024762	0.017564
i4_LQ_YHS_c9644/f1p3/4632	3.129622392	28.10219502	-3.1666	0.002477	0.017564
i5_LQ_YHS_c1626/f1p3/5298	0	10.74132545	-Inf	0.0024768	0.017564
i3_HQ_YHS_c499/f16p0/3908	873.4169098	131.4305193	2.7324	0.0024784	0.017572
i2_LQ_YHS_c55024/f1p0/2167	57.23383887	0.392261031	7.1889	0.0024791	0.017574
i5_LQ_YHS_c891/f1p0/5811	121.9254023	336.9505882	-1.4665	0.0024803	0.01758
i1_LQ_YHS_c12190/f1p0/1760	65.6151804	337.6487405	-2.3634	0.0024831	0.017597
i1_HQ_YHS_c29224/f5p0/1775	740.6830124	96.16517465	2.9453	0.0024847	0.017605
i2_LQ_YHS_c24626/f1p4/2860	267.5004083	98.65618339	1.4391	0.002485	0.017605
i0_LQ_YHS_c1959/f1p0/428	634.8939971	182.1331024	1.8015	0.0024864	0.017612
i1_LQ_YHS_c4935/f1p2/1770	616.0397222	0	Inf	0.0024894	0.017631
i2_LQ_YHS_c7213/f1p0/2720	0	10.70905836	-Inf	0.0024908	0.017638
i1_HQ_YHS_c2647/f3p0/1796	413.6459573	152.1820443	1.4426	0.0024918	0.01764
i3_LQ_YHS_c17630/f1p3/3722	1499.37911	520.1434197	1.5274	0.0024918	0.01764
i5_LQ_YHS_c3760/f1p1/5247	40.91382534	138.6137311	-1.7604	0.0024937	0.017651
i3_LQ_YHS_c8459/f1p0/3344	108.7745218	30.81018667	1.8199	0.0024947	0.017656
i2_LQ_YHS_c34601/f1p5/2323	43.50592288	6.932222969	2.6498	0.0024965	0.017666
i5_LQ_YHS_c951/f1p0/5307	1.263932271	21.36136281	-4.079	0.0025007	0.017693
i2_HQ_YHS_c1543/f3p2/2367	507.4824522	86.59846117	2.5509	0.0025047	0.017719
i4_LQ_YHS_c13529/f1p0/4108	302.6597157	837.092441	-1.4677	0.0025052	0.01772
i4_LQ_YHS_c5689/f1p0/4739	137.6819324	42.02398765	1.7121	0.002507	0.01773
i2_LQ_YHS_c38991/f1p4/2936	76.23301329	6.764173022	3.4944	0.0025119	0.017762
i4_LQ_YHS_c11966/f1p0/4687	52.37443851	160.618224	-1.6167	0.0025132	0.017768
i2_LQ_YHS_c51308/f1p2/2343	35.58877435	4.869060618	2.8697	0.0025151	0.017779
i2_LQ_YHS_c55545/f1p1/2571	79.46354899	229.8518254	-1.5323	0.0025166	0.017787
i1_LQ_YHS_c34960/f1p0/1704	330.6011038	1366.395874	-2.0472	0.0025198	0.017804
i2_LQ_YHS_c55461/f1p4/2363	11.89493657	59.71977155	-2.3279	0.0025194	0.017804
i4_LQ_YHS_c9315/f1p2/4338	59.18816249	187.5018102	-1.6635	0.0025242	0.017833
i1_LQ_YHS_c36887/f1p0/1930	41.00780826	132.3236526	-1.6901	0.0025259	0.017843
i2_LQ_YHS_c14800/f1p101/2017	345.3412796	0	Inf	0.0025272	0.017849
i2_HQ_YHS_c19923/f2p0/2829	123.4810967	28.76262441	2.102	0.0025296	0.017863
i1_LQ_YHS_c11352/f2p0/1486	1149.250261	127.411539	3.1731	0.0025332	0.017887
i1_LQ_YHS_c33816/f1p0/1563	288.9376745	760.7136121	-1.3966	0.0025361	0.017904
i2_LQ_YHS_c41711/f1p8/2772	109.9559443	337.7831341	-1.6192	0.0025429	0.01795
i3_LQ_YHS_c18406/f1p0/3345	0	23.20746202	-Inf	0.0025459	0.017968
i4_LQ_YHS_c4887/f1p1/4647	115.8131971	320.6369614	-1.4691	0.0025471	0.017974
i1_LQ_YHS_c12705/f1p2/2004	27.24510402	99.31452074	-1.866	0.0025476	0.017975
i1_LQ_YHS_c29131/f1p0/1276	2105.197647	358.8358477	2.5526	0.0025489	0.01798
i2_HQ_YHS_c30430/f4p1/2193	518.6839278	188.565125	1.4598	0.0025491	0.01798

i2_LQ_YHS_c18964/f1p3/2875	8.212218442	65.63693968	-2.9987	0.0025494	0.01798
i2_HQ_YHS_c12069/f2p2/2669	70.58141196	204.4616518	-1.5345	0.0025519	0.017995
i2_HQ_YHS_c7746/f2p4/2150	113.2641285	30.92152225	1.873	0.0025562	0.01802
i2_LQ_YHS_c24377/f1p1/2937	212.9790502	75.94556395	1.4877	0.0025558	0.01802
i2_LQ_YHS_c20619/f1p3/2954	310.9480341	114.4051162	1.4425	0.0025573	0.018022
i3_LQ_YHS_c8549/f1p0/3154	169.2988127	55.61015339	1.6062	0.0025572	0.018022
i1_LQ_YHS_c38859/f1p0/1331	78.30953431	0.746133264	6.7136	0.0025635	0.018059
i2_LQ_YHS_c49842/f1p0/2452	0	29.31783992	-Inf	0.0025636	0.018059
i3_LQ_YHS_c19113/f1p1/3622	778.6030114	208.6163336	1.9	0.0025633	0.018059
i1_LQ_YHS_c17859/f1p4/1980	496.2335818	184.7771922	1.4252	0.0025652	0.018067
i1_LQ_YHS_c24336/f1p1/1925	14.85460534	66.27699214	-2.1576	0.0025679	0.018084
i1_LQ_YHS_c8230/f1p0/1999	11.31035203	537.9489507	-5.5718	0.0025683	0.018084
i1_HQ_YHS_c4732/f2p1/2028	10.38379946	409.7700632	-5.3024	0.0025717	0.018105
i4_HQ_YHS_c2553/f2p0/4792	684.5313991	172.8677753	1.9854	0.0025749	0.018125
i2_LQ_YHS_c9068/f1p3/2683	57.22840325	10.72023598	2.4164	0.0025756	0.018127
i2_LQ_YHS_c54000/f1p1/2820	13.95075627	114.6941406	-3.0394	0.0025777	0.018139
i2_LQ_YHS_c7621/f1p2/2995	785.1701971	294.1903418	1.4163	0.002579	0.018146
i2_LQ_YHS_c27391/f1p0/2091	7.792635397	49.28938617	-2.6611	0.0025813	0.01816
i2_LQ_YHS_c13156/f1p4/2378	15.3396684	79.31911999	-2.3704	0.0025824	0.018165
i5_LQ_YHS_c4640/f1p0/5078	43.22976515	144.4468946	-1.7404	0.0025843	0.018175
i1_LQ_YHS_c7012/f1p7/1916	36.90456525	276.397238	-2.9049	0.0025862	0.018185
i2_LQ_YHS_c55712/f1p0/2506	3.29957601	29.16712885	-3.144	0.0025864	0.018185
i1_LQ_YHS_c33748/f1p12/1699	2.168239704	23.72444805	-3.4518	0.0025889	0.0182
i1_LQ_YHS_c4862/f1p11/1890	4.151421648	40.99234603	-3.3037	0.0025904	0.018208
i2_LQ_YHS_c23562/f1p0/2976	312.9836586	7.601578637	5.3636	0.0025911	0.01821
i2_LQ_YHS_c4034/f1p1/2726	0	44.57158986	-Inf	0.002592	0.018213
i2_LQ_YHS_c28807/f1p2/2300	730.9169606	271.3140029	1.4297	0.0025944	0.018228
i3_LQ_YHS_c19414/f1p0/3553	6.874834634	67.65361926	-3.2988	0.0025957	0.018234
i2_LQ_YHS_c49131/f1p0/2152	76.45927025	15.51804793	2.3007	0.0025985	0.018252
i3_LQ_YHS_c9637/f1p0/3160	9.60413322	49.35013021	-2.3613	0.0025995	0.018256
i2_LQ_YHS_c27143/f1p0/2463	254.0321265	57.64768292	2.1397	0.0026	0.018257
i2_HQ_YHS_c27144/f2p2/2766	556.6172537	22.64940239	4.6191	0.0026031	0.018276
i3_LQ_YHS_c2940/f1p1/3364	0	10.43867821	-Inf	0.0026118	0.018334
i1_HQ_YHS_c7593/f4p3/1822	305.6995617	50.97869215	2.5841	0.0026146	0.018351
i1_LQ_YHS_c29908/f2p0/1425	1675.631749	557.0939093	1.5887	0.0026171	0.018366
i2_LQ_YHS_c20436/f1p1/2897	72.55217885	13.24291791	2.4538	0.0026174	0.018366
i2_LQ_YHS_c64696/f1p0/2022	5.74730638	39.10556712	-2.7664	0.0026227	0.0184
i1_LQ_YHS_c14047/f1p0/1255	195.4319833	522.5102906	-1.4188	0.00263	0.018449
i1_LQ_YHS_c18252/f1p0/1914	134.5575924	41.57544571	1.6944	0.0026313	0.018452
i2_LQ_YHS_c26862/f1p0/2827	29.61477777	104.91131	-1.8248	0.002631	0.018452
i2_LQ_YHS_c20105/f1p0/2994	20.24897789	78.68297745	-1.9582	0.0026323	0.018457
i1_HQ_YHS_c17334/f2p5/1844	22.92786711	229.9453921	-3.3261	0.0026336	0.018463
i1_LQ_YHS_c20494/f1p0/1459	37.55591031	127.1324336	-1.7592	0.0026342	0.018465
i2_LQ_YHS_c24612/f1p1/2386	33.27191782	1.530655327	4.4421	0.0026359	0.018474
i11_LQ_YHS_c4/f1p0/11076	4.696104245	33.97552564	-2.855	0.0026374	0.018482
i3_LQ_YHS_c12357/f1p0/3215	10.22225898	54.35710526	-2.4108	0.0026404	0.0185
i1_LQ_YHS_c10609/f1p0/1843	23.99638737	1.100005497	4.4472	0.0026425	0.018512
i2_LQ_YHS_c18389/f1p1/2343	1.634047791	24.64755754	-3.9149	0.0026462	0.01853
i2_LQ_YHS_c50155/f1p1/2027	55.37020955	9.699732437	2.5131	0.0026458	0.01853
i2_LQ_YHS_c5965/f1p1/2288	115.6391793	315.4644619	-1.4478	0.0026457	0.01853
i1_LQ_YHS_c21593/f1p1/1496	85.98583871	0	Inf	0.0026471	0.018533
i3_LQ_YHS_c12334/f1p0/4003	99.35753225	24.11951366	2.0424	0.0026484	0.01854
i2_HQ_YHS_c18027/f2p3/2344	135.468847	36.6162983	1.8874	0.0026503	0.018551
i2_LQ_YHS_c15198/f1p0/2023	2011.605846	660.1915174	1.6074	0.0026544	0.018576
i3_LQ_YHS_c13147/f1p2/3521	45.29989196	149.3922191	-1.7215	0.0026616	0.018625
i4_LQ_YHS_c14887/f1p0/4246	3.072547138	37.53818156	-3.6109	0.0026624	0.018627
i2_HQ_YHS_c15726/f13p2/2232	413.9682856	155.9962829	1.408	0.0026722	0.018693
i2_LQ_YHS_c29407/f1p1/2072	103.9099048	289.0922537	-1.4762	0.0026728	0.018694
i1_LQ_YHS_c5488/f1p1/1535	526.0676994	26.83358634	4.2931	0.0026786	0.018732
i1_HQ_YHS_c39970/f36p0/1817	5125.366624	786.3407193	2.7044	0.0026808	0.018745
i2_LQ_YHS_c24966/f1p3/2516	34.02933055	110.8079814	-1.7032	0.0026869	0.018785
i2_LQ_YHS_c8609/f1p2/2953	12.24580122	62.89344402	-2.3606	0.0026916	0.018815
i2_LQ_YHS_c24024/f1p1/2390	18.69760018	74.41873831	-1.9928	0.0026924	0.018818
i2_LQ_YHS_c41572/f1p0/2924	145.8668674	5.209030874	4.8075	0.0026968	0.018846
i2_LQ_YHS_c23332/f1p12/2282	168.0250001	457.4944395	-1.4451	0.0026985	0.018853
i2_LQ_YHS_c64508/f1p0/2038	112.0629872	15.47973935	2.8559	0.0026985	0.018853
i5_LQ_YHS_c1965/f1p0/5149	7.020457193	44.9389306	-2.6783	0.0026994	0.018856
i2_LQ_YHS_c28398/f1p0/2070	85.46955852	5.627437293	3.9249	0.0027037	0.018882
i3_LQ_YHS_c16849/f1p0/3786	10.14927418	56.09100197	-2.4664	0.0027039	0.018882
i2_LQ_YHS_c5150/f2p0/2273	230.993151	624.773123	-1.4355	0.0027072	0.018902
i2_LQ_YHS_c28269/f1p0/2076	328.187294	118.209243	1.4732	0.0027107	0.018924
i3_LQ_YHS_c17786/f1p0/3876	134.9218559	41.10135117	1.7149	0.0027116	0.018928
i3_LQ_YHS_c11707/f1p0/3691	106.2416967	30.47744345	1.8015	0.0027151	0.018948
i4_HQ_YHS_c1882/f4p1/4535	203.4446765	537.0835419	-1.4005	0.0027153	0.018948
i1_LQ_YHS_c6863/f1p0/1929	63.34757241	1.569044125	5.3353	0.0027164	0.018949
i2_HQ_YHS_c60921/f52p7/2248	1093.12095	398.0364784	1.4575	0.0027158	0.018949
i3_LQ_YHS_c11575/f1p0/3204	45.62839179	142.9602768	-1.6476	0.0027167	0.018949
i2_HQ_YHS_c19989/f2p0/2395	94.93569366	259.3585713	-1.4499	0.0027224	0.018984
i3_LQ_YHS_c5861/f1p0/3765	22.04755206	136.5557433	-2.6308	0.0027224	0.018984
i2_LQ_YHS_c3296/f1p2/2499	125.1588377	35.25803629	1.8277	0.002726	0.019006
i1_LQ_YHS_c27894/f1p0/1184	728.5076243	274.1394529	1.41	0.00273	0.019031
i2_HQ_YHS_c49455/f3p1/2308	293.7568618	799.3366803	-1.4442	0.0027333	0.019051
i0_LQ_YHS_c954/f1p0/767	49.20377848	8.792263708	2.4845	0.0027351	0.019061
i1_HQ_YHS_c7526/f2p0/1683	822.1668282	50.30929694	4.0305	0.0027365	0.019068
i2_LQ_YHS_c4022/f1p0/2250	307.6467395	810.4665964	-1.3975	0.0027376	0.019071
i2_LQ_YHS_c40722/f1p0/2255	96.03835833	11.58931591	3.0508	0.0027381	0.019071
i2_LQ_YHS_c40881/f1p2/2467	126.7999152	338.857666	-1.4181	0.0027378	0.019071
i2_LQ_YHS_c56446/f1p1/2167	79.4977843	17.80992405	2.1582	0.0027397	0.01908
i1_LQ_YHS_c10224/f1p9/1463	532.7799475	200.1816928	1.4122	0.0027418	0.01909

i1_LQ_YHS_c25412/f1p0/1470	16.76815217	0.353872233	5.5664	0.002742	0.01909
i4_LQ_YHS_c6976/f1p0/4894	143.8748415	395.0249726	-1.4571	0.0027447	0.019106
i1_LQ_YHS_c26465/f1p0/1920	1.634047791	25.06983432	-3.9394	0.0027507	0.019145
i2_HQ_YHS_c2232/f3p1/2275	175.3493209	464.4877608	-1.4054	0.0027547	0.019171
i2_HQ_YHS_c17322/f9p1/2374	584.7030785	219.6209715	1.4127	0.0027559	0.019176
i1_HQ_YHS_c3844/f2p3/1511	1420.259983	121.351634	3.5489	0.002759	0.019195
i4_LQ_YHS_c4191/f1p0/4396	12.27554277	62.11942659	-2.3393	0.0027626	0.019217
i0_LQ_YHS_c2122/f1p8/865	373.5033684	136.7730806	1.4493	0.0027761	0.019308
i1_LQ_YHS_c6661/f1p44/1746	28.47088161	112.3671928	-1.9807	0.0027803	0.019335
i2_HQ_YHS_c18585/f2p2/2691	223.7518857	80.58263321	1.4734	0.002784	0.019357
i2_HQ_YHS_c12503/f5p1/2849	368.4203918	137.9987978	1.4167	0.0027894	0.019392
i1_LQ_YHS_c32019/f1p0/1484	308.2758921	1141.057848	-1.8881	0.0027913	0.019401
i4_LQ_YHS_c6635/f1p0/4361	32.25724281	115.8107735	-1.8441	0.0027915	0.019401
i2_LQ_YHS_c54995/f1p2/2229	21.3879303	0	Inf	0.0028045	0.019488
i1_LQ_YHS_c33804/f1p1/1363	990.463047	363.5198046	1.4461	0.0028056	0.019491
i4_LQ_YHS_c12742/f1p0/4950	15.92044488	89.5755672	-2.4922	0.0028052	0.019491
i1_LQ_YHS_c3992/f1p0/1396	70.87652375	0	Inf	0.0028081	0.019503
i3_LQ_YHS_c19147/f1p2/3176	483.7990165	1371.488609	-1.5033	0.0028079	0.019503
i3_LQ_YHS_c11174/f1p1/3805	30.21779939	108.0527153	-1.8383	0.0028102	0.019514
i2_LQ_YHS_c25836/f1p1/2496	630.3996154	1935.378021	-1.6183	0.0028139	0.019536
i3_LQ_YHS_c12985/f1p10/3315	9.529876276	52.45196124	-2.4605	0.0028141	0.019536
i1_HQ_YHS_c2501/f2p2/1826	534.7802166	2425.551896	-2.1813	0.0028165	0.01955
i1_LQ_YHS_c28341/f1p0/1359	66.89327497	192.965347	-1.5284	0.0028175	0.019551
i3_HQ_YHS_c2585/f2p0/3142	22.73325213	89.41664239	-1.9757	0.0028172	0.019551
i2_LQ_YHS_c36749/f1p1/2474	67.64080019	15.47906636	2.1276	0.0028189	0.019558
i1_LQ_YHS_c39625/f1p2/1349	45.99816864	145.0095767	-1.6565	0.002824	0.019588
i2_LQ_YHS_c5930/f1p0/2802	109.4624191	26.81636723	2.0292	0.0028239	0.019588
i1_LQ_YHS_c35333/f1p1/2026	280.3261925	752.0711228	-1.4238	0.0028257	0.019597
i1_LQ_YHS_c43956/f1p1/1042	26242.87642	1078.669573	4.6046	0.0028285	0.019608
i2_LQ_YHS_c37480/f1p0/2486	0	16.37654302	-Inf	0.0028281	0.019608
i4_LQ_YHS_c2386/f2p0/4725	0	10.59164062	-Inf	0.0028285	0.019608
i2_LQ_YHS_c55493/f1p17/2765	171.5117198	467.5853633	-1.4469	0.0028343	0.019645
i8_HQ_YHS_c8/f6p0/8775	0	57090.7187	-Inf	0.0028352	0.019648
i3_HQ_YHS_c21169/f22p1/3531	874.9536334	133.3644182	2.7138	0.0028376	0.019663
i3_LQ_YHS_c10063/f1p0/3213	89.55428191	1.466121141	5.9327	0.0028406	0.01968
i3_LQ_YHS_c20698/f1p1/3040	409.6674896	1161.595093	-1.5036	0.0028468	0.019719
i4_LQ_YHS_c11441/f1p1/4844	3.81866378	29.90382327	-2.9692	0.0028471	0.019719
i1_LQ_YHS_c25567/f1p15/1973	97.9328649	25.45320862	1.9439	0.0028522	0.019752
i5_LQ_YHS_c1826/f1p0/5682	512.7829495	135.6484685	1.9185	0.0028534	0.019755
i5_LQ_YHS_c2865/f1p0/5273	39.42205879	241.3606478	-2.6141	0.0028535	0.019755
i4_HQ_YHS_c2247/f2p6/4529	0	10.53934985	-Inf	0.0028541	0.019756
i3_LQ_YHS_c4137/f1p2/3469	69.76670499	432.0916367	-2.6307	0.0028546	0.019758
i1_LQ_YHS_c33155/f1p0/1763	179.7516273	479.8968789	-1.4167	0.0028558	0.019763
i2_LQ_YHS_c34717/f1p3/2321	277.4059183	742.8657745	-1.4211	0.0028562	0.019763
i4_HQ_YHS_c2347/f2p5/4718	231.9004133	17.31189592	3.7437	0.0028604	0.019789
i0_LQ_YHS_c1627/f1p4/633	2103.115033	665.0395594	1.661	0.0028635	0.019808
i1_HQ_YHS_c1847/f4p3/1759	294.2359375	104.3392681	1.4957	0.0028654	0.019812
i2_LQ_YHS_c4970/f1p37/2856	0	55879.3391	-Inf	0.0028651	0.019812
i8_LQ_YHS_c94/f1p0/8191	83.20430096	1.459999436	5.8326	0.0028652	0.019812
i2_LQ_YHS_c54010/f1p4/2125	228.5777939	607.1330272	-1.4093	0.0028809	0.019916
i2_LQ_YHS_c13262/f1p0/2970	1.793510725	22.07803355	-3.6218	0.0028825	0.019925
i2_LQ_YHS_c52824/f1p0/2284	0	10.47647423	-Inf	0.0028851	0.01994
i2_HQ_YHS_c3635/f2p3/2143	2663.301401	249.5487273	3.4158	0.0028902	0.019972
i2_LQ_YHS_c49061/f1p0/2513	714.4678218	11.72450599	5.9293	0.0028913	0.019977
i3_LQ_YHS_c8133/f1p0/3302	0	10.46257225	-Inf	0.002892	0.019979
i2_LQ_YHS_c34102/f1p0/2124	174.3042149	47.42101193	1.878	0.002898	0.020018
i4_LQ_YHS_c7756/f1p3/4516	237.7064057	614.828363	-1.371	0.0029003	0.02003
i1_LQ_YHS_c19359/f1p7/1904	0	10.44420714	-Inf	0.0029012	0.020034
i2_LQ_YHS_c21157/f1p3/2359	109.2565989	287.0623045	-1.3936	0.0029021	0.020037
i2_LQ_YHS_c55676/f1p7/2938	847.0665728	80.15849788	3.4015	0.002904	0.020047
i3_LQ_YHS_c17279/f1p0/3394	344.2925202	49.49140132	2.7984	0.0029061	0.020059
i4_LQ_YHS_c2772/f1p2/4904	289.7820524	107.1747091	1.435	0.0029078	0.020068
i1_LQ_YHS_c33985/f1p4/1794	51.78237432	157.1400457	-1.6015	0.0029106	0.020084
i2_LQ_YHS_c3924/f1p18/3126	7.368785939	46.47902477	-2.6571	0.0029127	0.020096
i2_LQ_YHS_c10600/f4p2/2207	284.2396818	106.801879	1.4122	0.0029195	0.02014
i1_LQ_YHS_c13470/f1p2/1366	166.2000508	57.2714159	1.537	0.00292	0.020141
i0_LQ_YHS_c531/f1p0/776	0	10.39969663	-Inf	0.0029235	0.020161
i2_HQ_YHS_c27912/f2p1/2056	1372.736446	476.433041	1.5267	0.0029242	0.020161
i2_LQ_YHS_c55793/f1p0/2552	0	15.94810499	-Inf	0.0029238	0.020161
i1_LQ_YHS_c22972/f1p0/1566	220.0494057	15.82740966	3.7973	0.0029278	0.020183
i2_LQ_YHS_c28134/f2p2/2169	406.1510415	157.3038325	1.3685	0.002929	0.020188
i1_LQ_YHS_c12915/f1p4/1722	301.3515384	71.3067559	2.0793	0.00293	0.020193
i1_LQ_YHS_c34587/f1p0/1813	152.7259361	401.3902492	-1.3941	0.0029324	0.020206
i3_LQ_YHS_c9772/f1p0/3261	155.042132	51.78094701	1.5822	0.0029337	0.020212
i2_HQ_YHS_c5367/f2p1/2568	176.112314	58.72707196	1.5844	0.0029366	0.020229
i3_LQ_YHS_c7129/f1p0/3519	173.6502598	458.8047909	-1.4017	0.0029433	0.020273
i2_LQ_YHS_c39533/f1p3/2610	468.7028417	130.5213118	1.8444	0.002944	0.020275
i1_HQ_YHS_c1801/f3p0/2003	355.3576169	94.46030813	1.9115	0.0029485	0.020302
i2_LQ_YHS_c49097/f1p3/2326	106.724699	30.78960975	1.7934	0.0029547	0.02034
i4_LQ_YHS_c12119/f1p0/4905	262.4270846	699.9972359	-1.4154	0.0029552	0.02034
i4_LQ_YHS_c5918/f1p0/4668	2.266013927	60.60662269	-4.7412	0.0029548	0.02034
i2_LQ_YHS_c49786/f1p16/2377	314.8318966	1365.142738	-2.1164	0.002958	0.020357
i6_LQ_YHS_c1128/f1p0/6843	0.991590972	25.60332692	-4.6904	0.002961	0.020374
i1_LQ_YHS_c32426/f1p2/1783	0	21.30456935	-Inf	0.0029644	0.020395
i1_LQ_YHS_c12506/f1p3/1866	2.754893412	25.72805192	-3.2233	0.0029663	0.020405
i1_LQ_YHS_c20429/f1p0/1542	0.272341299	34.37280304	-6.9797	0.002969	0.020417
i2_HQ_YHS_c10564/f2p0/2210	38.5614781	1.864503878	4.3703	0.0029686	0.020417
i2_LQ_YHS_c50971/f1p0/2521	645.6111852	1900.232685	-1.5574	0.0029693	0.020417
i1_LQ_YHS_c33294/f1p1/1986	11.86219237	56.91493907	-2.2624	0.0029725	0.020436

i4_LQ_YHS_c13925/f1p0/4165	128.1037243	40.04759496	1.6775	0.0029753	0.020452
i2_HQ_YHS_c22723/f2p0/2523	0	13.27905536	-Inf	0.0029765	0.020455
i2_LQ_YHS_c36100/f1p2/2487	106.660813	29.79537136	1.8399	0.0029766	0.020455
i1_LQ_YHS_c39483/f1p0/1250	171.1211566	432.6033608	-1.338	0.0029788	0.020463
i2_LQ_YHS_c22355/f1p2/2145	0	10.29065194	-Inf	0.002979	0.020463
i2_LQ_YHS_c28103/f1p1/1911	232.2462228	6.090946992	5.2528	0.0029786	0.020463
i2_HQ_YHS_c45459/f8p0/2385	274.3792831	99.42561792	1.4645	0.0029817	0.020475
i2_LQ_YHS_c34404/f1p2/2961	24.02439004	117.8472768	-2.2943	0.0029816	0.020475
i2_LQ_YHS_c12426/f1p8/2297	57.93750092	11.40124228	2.3453	0.0029866	0.020506
i2_LQ_YHS_c40398/f1p1/2130	11.46901793	0	Inf	0.0029878	0.020512
i2_HQ_YHS_c4013/f3p8/2994	184.5079447	558.9474031	-1.599	0.0029909	0.02053
i2_LQ_YHS_c20569/f1p0/2780	0	10.25672628	-Inf	0.0029964	0.020565
i2_LQ_YHS_c59514/f1p0/2045	56.18896395	162.6031095	-1.533	0.003001	0.020594
i2_LQ_YHS_c5090/f1p0/3002	127.1365469	335.2956029	-1.3991	0.0030024	0.0206
i3_LQ_YHS_c16762/f1p0/3069	20.25578021	83.29844468	-2.04	0.0030056	0.020619
i3_LQ_YHS_c10234/f1p1/3402	29.14687966	104.1489827	-1.8372	0.0030082	0.020635
i1_HQ_YHS_c37906/f2p0/1139	470.0229278	1271.438421	-1.4357	0.0030122	0.020659
i2_LQ_YHS_c10439/f1p3/2957	27.34911089	0	Inf	0.0030188	0.020701
i1_LQ_YHS_c14663/f1p0/1217	69.48455872	200.2362744	-1.5269	0.0030215	0.020717
i1_LQ_YHS_c3507/f1p10/1949	74.04153756	1.793847987	5.3672	0.0030263	0.020741
i2_LQ_YHS_c11581/f1p0/2153	0	10.19997237	-Inf	0.0030259	0.020741
i2_LQ_YHS_c19070/f1p0/2975	133.8367475	666.6966363	-2.3166	0.0030258	0.020741
i2_LQ_YHS_c7093/f1p1/2768	50.17009655	157.1802535	-1.6475	0.0030273	0.020744
i2_LQ_YHS_c6274/f1p0/2156	99.93160935	27.87292802	1.8421	0.0030285	0.020749
i3_LQ_YHS_c7297/f1p2/3408	8.576448363	48.14487017	-2.4889	0.0030288	0.020749
i1_LQ_YHS_c38438/f1p0/1199	270.6602437	693.5151042	-1.3574	0.0030302	0.020756
i3_LQ_YHS_c12454/f1p0/3327	860.2508627	314.1233784	1.4534	0.0030342	0.02078
i1_LQ_YHS_c4952/f1p3/1393	181.7981878	20.90231625	3.1206	0.0030359	0.020789
i2_LQ_YHS_c41829/f1p0/2893	60.36645553	2.681293034	4.4927	0.003037	0.020793
i2_LQ_YHS_c50375/f1p0/2583	11.53197041	57.94089133	-2.3289	0.0030389	0.020803
i2_LQ_YHS_c38644/f1p2/2144	19.11763321	77.00662741	-2.0101	0.0030446	0.020839
i1_LQ_YHS_c36942/f1p0/1615	284.0926028	6.432575814	5.4648	0.0030483	0.020862
i3_LQ_YHS_c12362/f1p2/3077	742.358208	2349.670648	-1.6623	0.0030506	0.020875
i1_LQ_YHS_c32812/f1p0/1903	42.68108065	0	Inf	0.0030551	0.020902
i1_LQ_YHS_c33014/f1p18/1843	352.9597538	132.746915	1.4108	0.0030574	0.020915
i2_HQ_YHS_c42199/f2p0/2995	0	12.12229595	-Inf	0.0030609	0.020936
i2_LQ_YHS_c35677/f1p2/2218	180.2317885	59.70365777	1.594	0.0030626	0.020945
i2_LQ_YHS_c13687/f1p25/2573	1321.779207	84.58703749	3.9659	0.0030644	0.020954
i2_LQ_YHS_c1617/f9p4/2250	236.3016821	609.7965144	-1.3677	0.0030678	0.020971
i3_LQ_YHS_c16863/f1p0/3319	98.16064056	392.053313	-1.9978	0.0030676	0.020971
i1_LQ_YHS_c38894/f1p0/1202	712.3367552	118.3773732	2.5892	0.0030692	0.020978
i0_LQ_YHS_c1006/f1p0/995	653.613809	1982.816697	-1.601	0.003078	0.021035
i2_LQ_YHS_c38427/f1p3/2180	289.1673003	776.1627451	-1.4245	0.0030824	0.021058
i2_LQ_YHS_c39943/f1p1/2479	8.565957679	47.26461262	-2.4641	0.0030819	0.021058
i2_LQ_YHS_c8807/f1p3/2290	55.85699474	402.5470531	-2.8493	0.0030826	0.021058
i2_HQ_YHS_c2512/f4p13/2128	802.5823275	7.815204877	6.6822	0.0030891	0.021099
i1_LQ_YHS_c968/f16p0/1777	972.6237141	3430.900906	-1.8186	0.0030928	0.021119
i2_LQ_YHS_c26330/f1p1/2135	109.7476912	387.5026157	-1.82	0.0030927	0.021119
i4_LQ_YHS_c10487/f1p0/4301	21.24022182	85.44324056	-2.0082	0.0030936	0.021121
i2_LQ_YHS_c12957/f1p6/2110	20.23514589	0.719987877	4.8127	0.0030967	0.021139
i3_LQ_YHS_c5876/f1p4/3127	84.64437762	22.58099784	1.9063	0.0030972	0.02114
i2_LQ_YHS_c4883/f1p2/2714	52.52132484	309.4992711	-2.559	0.0031005	0.021156
i2_LQ_YHS_c8733/f1p1/2360	96.93904319	265.7944236	-1.4552	0.0031003	0.021156
i1_LQ_YHS_c32183/f1p0/1729	118.4357721	1.852260467	5.9987	0.0031044	0.02118
i3_LQ_YHS_c12193/f1p0/3255	6489.311719	206.491601	4.9739	0.0031057	0.021186
i1_LQ_YHS_c36571/f1p0/1661	131.5686656	40.42540078	1.7025	0.0031076	0.021196
i3_HQ_YHS_c2143/f2p0/3283	589.2257454	1748.058517	-1.5689	0.0031135	0.021233
i2_HQ_YHS_c11165/f2p0/2353	150.7527542	8.501267095	4.1484	0.003117	0.021254
i1_HQ_YHS_c29310/f5p0/1669	1347.122974	41.3278136	5.0266	0.0031197	0.021269
i2_LQ_YHS_c50802/f1p1/2696	100.0767735	27.8929517	1.8431	0.0031222	0.021283
i0_LQ_YHS_c2749/f1p0/799	49.42398828	9.255180631	2.4169	0.0031251	0.0213
i1_HQ_YHS_c11838/f2p0/1859	150.900173	387.6680611	-1.3612	0.0031286	0.021321
i1_LQ_YHS_c2480/f3p1/1916	245.4312023	88.01391055	1.4795	0.0031301	0.021328
i2_LQ_YHS_c9900/f1p2/2744	1.131336305	73.23318825	-6.0164	0.0031314	0.021331
i3_HQ_YHS_c8748/f2p0/3585	860.6342923	218.5228581	1.9776	0.0031313	0.021331
i2_LQ_YHS_c12846/f1p9/2224	0	62.77918411	-Inf	0.0031324	0.021335
i2_LQ_YHS_c32881/f1p2/2095	893.4236087	336.279296	1.4097	0.0031345	0.021346
i3_LQ_YHS_c6910/f1p0/3623	39.02474601	6.246160752	2.6433	0.0031366	0.021358
i1_LQ_YHS_c9659/f1p8/1932	11.36535811	56.93488254	-2.3247	0.0031424	0.021394
i2_HQ_YHS_c6729/f2p1/2601	78.95607085	228.975558	-1.5361	0.0031429	0.021394
i1_LQ_YHS_c24505/f1p5/1944	781.373277	292.4860274	1.4176	0.0031445	0.021402
i1_HQ_YHS_c1976/f12p5/1859	534.3895027	1511.197049	-1.4997	0.0031459	0.021409
i3_LQ_YHS_c13475/f1p3/3162	102.1989984	29.89157986	1.7736	0.003148	0.02142
i2_LQ_YHS_c14112/f1p1/2123	162.0738853	434.014463	-1.4211	0.0031525	0.021448
i3_LQ_YHS_c14024/f1p4/3018	92.45479788	2.224497816	5.3772	0.0031556	0.021466
i1_LQ_YHS_c11324/f1p0/1921	532.3647026	77.99976047	2.7709	0.003161	0.021499
i1_LQ_YHS_c35940/f1p1/1867	1.34660235	20.53454204	-3.9307	0.0031647	0.021519
i2_LQ_YHS_c50853/f1p12/2659	9.400621627	57.54543293	-2.6139	0.0031645	0.021519
i3_LQ_YHS_c6465/f1p1/3191	298.4020456	110.5898525	1.432	0.0031671	0.021532
i1_LQ_YHS_c9441/f1p0/1679	977.2350875	357.0248597	1.4527	0.0031703	0.021546
i2_HQ_YHS_c15713/f11p3/2699	21.17808312	138.4191012	-2.7084	0.0031702	0.021546
i2_LQ_YHS_c12976/f1p1/2302	65.73648027	14.8086449	2.1503	0.0031706	0.021546
i2_LQ_YHS_c44380/f1p0/2041	33.37602763	4.359974625	2.9364	0.0031726	0.021557
i3_LQ_YHS_c10920/f1p2/3314	165.3798794	432.1120076	-1.3856	0.0031747	0.021568
i1_LQ_YHS_c20629/f1p2/1384	522.4480067	198.919286	1.3931	0.0031824	0.021612
i2_LQ_YHS_c12035/f1p1/2510	75.80562028	18.69065462	2.02	0.0031826	0.021612
i3_HQ_YHS_c21230/f34p3/3897	949.7492519	3216.391732	-1.7598	0.0031823	0.021612
i2_HQ_YHS_c20175/f5p0/2247	679.1908943	255.1848118	1.4123	0.0031833	0.021614
i2_LQ_YHS_c20594/f1p2/2973	61.37232849	180.0205736	-1.5525	0.0031837	0.021614

i1_LQ_YHS_c32303/f1p0/1890	149.4705619	48.67453307	1.6186	0.0031843	0.021615
i1_LQ_YHS_c26139/f1p3/1729	494.8621733	188.5067927	1.3924	0.0031876	0.021635
i2_LQ_YHS_c20125/f1p0/2375	108.6695898	290.4568302	-1.4184	0.0031884	0.021637
i4_LQ_YHS_c10902/f1p1/4478	0	12.8116753	-Inf	0.0031889	0.021637
i3_LQ_YHS_c5886/f1p1/3252	30.44736417	108.0628272	-1.8275	0.003193	0.021662
i2_LQ_YHS_c51307/f1p18/2626	1411.665907	136.0461459	3.3752	0.0031938	0.021664
i8_LQ_YHS_c87/f1p0/8297	0.272341299	13.01811405	-5.579	0.0031985	0.021693
i1_LQ_YHS_c20724/f1p0/1508	2718.488489	836.0819702	1.7011	0.0031993	0.021696
i2_LQ_YHS_c14754/f1p0/2078	215.6826845	57.6098869	1.9045	0.0032046	0.021725
i2_LQ_YHS_c44753/f1p0/2069	172.9226389	28.46384752	2.6029	0.0032041	0.021725
i2_LQ_YHS_c39696/f1p0/2796	157.5951618	5.692564255	4.791	0.0032154	0.021795
i2_LQ_YHS_c34058/f1p1/2311	25.27576246	90.88438255	-1.8463	0.0032161	0.021797
i1_LQ_YHS_c23545/f1p33/1643	57.90580988	1.112248908	5.7022	0.0032174	0.0218
i3_LQ_YHS_c12111/f1p5/3331	153.9390796	30.56480589	2.3324	0.0032173	0.0218
i3_LQ_YHS_c2875/f1p9/3585	5.916454592	72.2934931	-3.6111	0.0032245	0.021845
i1_LQ_YHS_c2809/f2p2/1867	55.22884503	185.4174375	-1.7473	0.0032282	0.021866
i2_LQ_YHS_c36143/f1p5/2140	2082.736585	140.2066297	3.8929	0.0032284	0.021866
i2_LQ_YHS_c9913/f1p0/2498	76.55219528	219.0305239	-1.5166	0.0032347	0.021905
i1_LQ_YHS_c27577/f1p0/1138	47.76781505	8.560272352	2.4803	0.0032354	0.021907
i1_LQ_YHS_c12111/f1p5/1443	385.7998506	145.6522337	1.4053	0.0032378	0.02192
i2_LQ_YHS_c26673/f1p0/2401	170.9426283	436.8222571	-1.3535	0.003241	0.021939
i2_LQ_YHS_c28046/f1p1/2043	54.56032665	10.73966689	2.3449	0.0032429	0.021949
i3_LQ_YHS_c18868/f1p0/3082	10.08966302	209.7890513	-4.378	0.0032454	0.021962
i2_HQ_YHS_c24120/f2p15/2555	3192.252802	956.3052403	1.739	0.0032467	0.021967
i1_LQ_YHS_c22269/f1p6/1901	73.58478234	209.2517241	-1.5078	0.0032502	0.021989
i2_LQ_YHS_c64455/f1p1/2026	394.1902132	147.8238887	1.415	0.0032508	0.021989
i2_HQ_YHS_c42077/f3p1/2208	87.18066367	341.5278259	-1.9699	0.003256	0.022022
i5_LQ_YHS_c4746/f1p0/5034	111.5077649	6.682932287	4.0605	0.0032599	0.022045
i3_HQ_YHS_c21276/f6p3/3724	397.0571544	150.6413969	1.3982	0.003261	0.022046
i3_LQ_YHS_c19717/f1p3/3982	1.778406583	21.50441337	-3.596	0.0032606	0.022046
i4_LQ_YHS_c8816/f1p0/4389	499.9773412	12.37549654	5.3363	0.0032629	0.022056
i2_LQ_YHS_c14357/f1p9/2056	452.47299	1217.229268	-1.4277	0.0032662	0.022075
i1_LQ_YHS_c32014/f1p0/1782	40.86266081	125.2043009	-1.6154	0.0032698	0.022097
i3_HQ_YHS_c15574/f2p1/3211	222.0953032	589.539813	-1.4084	0.0032716	0.022102
i3_LQ_YHS_c5687/f1p1/3366	52.70511883	160.0378498	-1.6024	0.0032712	0.022102
i3_LQ_YHS_c8657/f1p0/3315	101.7432101	29.35022678	1.7935	0.0032736	0.022113
i2_LQ_YHS_c19065/f1p3/2407	1.800660093	43.37091272	-4.5901	0.0032745	0.022115
i2_LQ_YHS_c41906/f1p0/2529	113.6394104	31.94644826	1.8307	0.0032801	0.02215
i2_LQ_YHS_c49450/f1p2/2561	32.91734805	140.4244043	-2.0929	0.0032823	0.022162
i3_LQ_YHS_c5642/f1p9/3472	7.038911026	39.53680973	-2.4898	0.0032851	0.022178
i2_LQ_YHS_c33067/f1p0/2211	200.2740499	62.87894927	1.6713	0.003289	0.022197
i2_LQ_YHS_c37965/f1p14/1973	30.42165803	461.0736735	-3.9218	0.0032894	0.022197
i4_LQ_YHS_c3789/f1p0/4724	200.5379781	51.93458242	1.9491	0.0032885	0.022197
i1_HQ_YHS_c2006/f5p0/1685	463.0829926	179.0145648	1.3712	0.0032908	0.022201
i1_LQ_YHS_c25725/f1p15/1645	1.044052767	18.34961857	-4.1355	0.0032905	0.022201
i3_LQ_YHS_c5634/f1p0/3131	130.6954999	340.366838	-1.3809	0.0032932	0.022214
i1_HQ_YHS_c1899/f3p7/1768	47.89232817	148.2415419	-1.6301	0.0032953	0.022221
i1_LQ_YHS_c8895/f1p1/1914	175.5670534	41.48382011	2.0814	0.0032946	0.022221
i3_LQ_YHS_c9564/f1p2/3299	133.9498832	9.67358705	3.7915	0.0032959	0.022221
i5_LQ_YHS_c1534/f1p0/5816	9.251657751	48.21446027	-2.3817	0.0032962	0.022221
i4_LQ_YHS_c7368/f1p5/4419	306.6780219	113.88434	1.4292	0.0032984	0.022234
i1_LQ_YHS_c6604/f1p0/1874	9.456424738	51.76874315	-2.4527	0.0033011	0.022248
i3_LQ_YHS_c8173/f1p1/3140	1075.345203	369.1945905	1.5423	0.0033025	0.022255
i1_LQ_YHS_c14397/f1p0/1168	365.6065286	57.75680782	2.6622	0.0033032	0.022256
i3_LQ_YHS_c11471/f1p0/3753	61.68202744	12.94461403	2.2525	0.0033078	0.022284
i0_HQ_YHS_c380/f2p0/813	1230.776175	349.2836206	1.8171	0.0033091	0.02229
i3_LQ_YHS_c18747/f1p0/3143	19.32367234	135.7929035	-2.813	0.0033113	0.022302
i1_LQ_YHS_c19870/f1p0/1658	43.15009772	140.0242839	-1.6982	0.0033129	0.022306
i1_LQ_YHS_c32318/f1p0/1570	44.83582702	4.699944881	3.2539	0.0033127	0.022306
i1_LQ_YHS_c23361/f1p0/1676	1275.976258	203.2403977	2.6503	0.0033171	0.022331
i4_LQ_YHS_c13871/f1p0/4076	52.46933816	10.67453993	2.2973	0.0033224	0.022364
i1_LQ_YHS_c13861/f1p0/1303	0	18.6049112	-Inf	0.0033244	0.022371
i1_LQ_YHS_c27980/f1p0/1132	1670.491175	566.5239357	1.5601	0.003324	0.022371
i2_HQ_YHS_c1594/f4p0/2630	8655.870158	1427.464588	2.6002	0.0033271	0.022386
i1_LQ_YHS_c33286/f1p0/1966	86.4549336	242.2244383	-1.4863	0.003328	0.022389
i4_LQ_YHS_c13752/f1p0/4142	478.1514638	184.2259273	1.376	0.0033288	0.022392
i3_LQ_YHS_c3762/f1p6/3083	8.357035595	48.71351465	-2.5433	0.0033295	0.022393
i1_LQ_YHS_c33969/f1p1/2001	0	17.80814572	-Inf	0.0033336	0.022418
i2_LQ_YHS_c28486/f1p144/2063	266.5233709	0.353872233	9.5568	0.0033357	0.022429
i1_LQ_YHS_c34286/f1p0/1660	13.25537929	58.75499567	-2.1481	0.0033384	0.022444
i2_LQ_YHS_c18387/f1p0/2461	85.9241165	226.6111746	-1.3991	0.0033406	0.022455
i1_LQ_YHS_c14228/f1p4/1383	283.3393679	782.7001937	-1.4659	0.0033428	0.022464
i1_LQ_YHS_c34326/f1p0/1842	11.22688492	0	Inf	0.0033431	0.022464
i1_LQ_YHS_c37034/f1p0/1561	1358.693855	490.0423536	1.4712	0.0033433	0.022464
i3_LQ_YHS_c18990/f1p0/3874	17.21885185	0	Inf	0.0033441	0.022467
i2_LQ_YHS_c14588/f1p1/2111	362.7388179	136.2689307	1.4125	0.0033561	0.022543
i4_HQ_YHS_c1879/f3p9/4479	291.5104302	108.8828137	1.4208	0.0033565	0.022543
i5_LQ_YHS_c3232/f1p29/5196	60.79662383	13.39706588	2.1821	0.0033743	0.02266
i2_LQ_YHS_c26123/f1p0/2934	14.12566201	202.1681912	-3.8392	0.0033755	0.022661
i4_LQ_YHS_c13047/f1p1/4168	15.34602074	70.40868646	-2.1979	0.0033755	0.022661
i4_LQ_YHS_c11728/f1p1/5001	384.4893218	997.2735692	-1.375	0.0033776	0.022672
i1_LQ_YHS_c4583/f1p5/1910	253.1517169	18.46419219	3.7772	0.0033802	0.022683
i2_HQ_YHS_c3391/f3p0/2114	432.9781852	12.19354462	5.1501	0.0033804	0.022683
i2_LQ_YHS_c21971/f1p2/2331	6.474510828	75.92597373	-3.5518	0.0033806	0.022683
i3_HQ_YHS_c21146/f2p0/3066	109.1460291	29.68679968	1.8784	0.0033813	0.022684
i8_LQ_YHS_c108/f1p0/8650	0.529578455	18.0491029	-5.0909	0.0033816	0.022684
i3_LQ_YHS_c18816/f1p2/3940	330.8391438	2237.541052	-2.7577	0.0033865	0.022713
i3_HQ_YHS_c879/f13p0/3299	1108.581687	411.3064136	1.4304	0.0033908	0.022739
i2_HQ_YHS_c17592/f8p2/2317	604.3651334	1769.559094	-1.5499	0.0033934	0.022752

i2_LQ_YHS_c25671/f1p0/2284	0	10.15933223	-Inf	0.0033936	0.022752
i2_LQ_YHS_c37831/f1p12/2968	530.4538163	1528.746247	-1.527	0.0033947	0.022755
i2_LQ_YHS_c22168/f1p4/2563	5.946196141	39.64202473	-2.737	0.0033978	0.022773
i1_HQ_YHS_c40144/f6p1/1369	928.2270454	3070.910107	-1.7261	0.0034054	0.022821
i3_HQ_YHS_c21111/f3p3/3386	1139.738147	307.6719249	1.8892	0.0034062	0.022823
i7_LQ_YHS_c115/f1p0/7661	2.070465482	21.76361591	-3.3939	0.0034085	0.022836
i3_LQ_YHS_c7030/f1p2/3572	87.26446108	248.5195332	-1.5099	0.0034109	0.022849
i1_LQ_YHS_c20128/f1p14/1550	991.2416505	3469.61917	-1.8075	0.0034185	0.022893
i1_LQ_YHS_c27802/f1p0/1083	3102.614968	900.1529065	1.7852	0.003419	0.022893
i2_LQ_YHS_c51026/f1p24/2331	826.4593797	67.39022775	3.6163	0.0034189	0.022893
i2_HQ_YHS_c32642/f2p0/2716	222.6330901	8.913551808	4.6425	0.0034209	0.022903
i4_LQ_YHS_c14739/f1p0/4849	371.4194898	1026.597187	-1.4667	0.0034285	0.022951
i3_HQ_YHS_c5481/f2p0/3336	0	24.92409895	-Inf	0.0034335	0.022978
i4_LQ_YHS_c9160/f1p1/4983	0	10.0903349	-Inf	0.0034334	0.022978
i2_HQ_YHS_c17821/f2p1/2722	484.1653456	189.1143386	1.3562	0.0034383	0.023007
i2_LQ_YHS_c54238/f1p0/2186	37.31459108	119.5819602	-1.6802	0.0034396	0.023012
i1_LQ_YHS_c5879/f1p8/1983	52.28126938	157.6998441	-1.5928	0.0034459	0.023051
i4_LQ_YHS_c10804/f1p0/4374	0	10.06418951	-Inf	0.0034486	0.023066
i1_HQ_YHS_c7496/f3p11/1883	123.928199	322.121128	-1.3781	0.0034525	0.023089
i2_LQ_YHS_c10465/f1p3/2069	8.813967918	48.80091664	-2.469	0.0034534	0.023091
i2_LQ_YHS_c39072/f1p22/2371	0	10.05028754	-Inf	0.0034567	0.02311
i1_LQ_YHS_c35094/f1p0/1918	129.6135893	348.3237892	-1.4262	0.0034607	0.02313
i2_LQ_YHS_c20253/f1p1/2214	203.7703101	71.50541437	1.5108	0.0034604	0.02313
i1_LQ_YHS_c10421/f1p1/1602	281.4830793	90.7414122	1.6332	0.003463	0.023137
i2_LQ_YHS_c22673/f1p0/2962	286.4082937	107.5530682	1.413	0.0034631	0.023137
i6_LQ_YHS_c780/f1p0/6849	0	10.03970269	-Inf	0.0034629	0.023137
i2_HQ_YHS_c61402/f12p3/2562	154.4922663	395.9178268	-1.3577	0.0034664	0.023155
i2_LQ_YHS_c39529/f1p0/2478	89.69778977	241.536205	-1.4291	0.0034677	0.023155
i2_LQ_YHS_c52095/f1p3/2880	113.9208673	299.4611814	-1.3943	0.0034677	0.023155
i3_HQ_YHS_c15284/f8p0/3498	475.721833	33.50707979	3.8276	0.0034668	0.023155
i2_HQ_YHS_c11372/f3p1/2313	48.94685488	327.6407841	-2.7428	0.00347	0.023167
i1_LQ_YHS_c4994/f1p0/1552	291.3726176	108.9612092	1.4191	0.0034717	0.023175
i2_LQ_YHS_c26050/f1p0/2644	26.71137885	95.98887142	-1.8454	0.0034738	0.023186
i0_LQ_YHS_c1239/f1p0/868	142.6137515	2.218376111	6.0065	0.0034748	0.02319
i2_HQ_YHS_c2457/f5p13/2610	0	120.6291563	-Inf	0.0034771	0.023202
i1_LQ_YHS_c23163/f1p0/1430	1075.711238	397.3715441	1.4367	0.0034791	0.023212
i4_LQ_YHS_c10573/f1p0/4908	458.3931006	176.1010805	1.3802	0.0034818	0.023227
i1_LQ_YHS_c18205/f1p0/1646	375.0397569	99.03118464	1.9211	0.0034838	0.023237
i4_LQ_YHS_c11383/f1p19/4512	97.8439538	27.97924835	1.8061	0.0034846	0.023239
i2_LQ_YHS_c38864/f1p0/2072	0	9.993533623	-Inf	0.00349	0.023272
i3_LQ_YHS_c11470/f1p0/3686	4.465734058	30.83696438	-2.7877	0.0034953	0.023304
i2_LQ_YHS_c52521/f1p1/2177	5.449370255	90.42640066	-4.0526	0.0034963	0.023307
i1_LQ_YHS_c14624/f1p0/1296	266.1846138	97.93177192	1.4426	0.0035023	0.023344
i2_HQ_YHS_c17618/f2p8/2557	345.371542	927.085463	-1.4246	0.0035045	0.023352
i2_LQ_YHS_c21051/f1p1/1970	23.67271161	172.5893347	-2.866	0.003504	0.023352
i1_LQ_YHS_c35696/f1p1/1503	0	12.14066107	-Inf	0.0035127	0.023404
i3_HQ_YHS_c15496/f4p0/3158	334.2011662	883.8098112	-1.403	0.0035173	0.023431
i0_LQ_YHS_c1750/f1p1/630	161.7982446	51.03035061	1.6648	0.0035206	0.02345
i3_LQ_YHS_c5760/f1p2/3716	105.8023961	31.35217208	1.7547	0.0035278	0.023495
i2_LQ_YHS_c21776/f1p0/2381	59.82842207	200.9303158	-1.7478	0.0035317	0.023516
i2_LQ_YHS_c50065/f1p0/2808	14.88020017	66.07876713	-2.1508	0.003532	0.023516
i2_LQ_YHS_c6705/f1p0/2389	297.9565123	770.0471965	-1.3698	0.0035338	0.023522
i3_LQ_YHS_c14606/f1p3/3124	68.35211184	1.466121141	5.5429	0.0035344	0.023522
i9_LQ_YHS_c52/f1p0/9143	275.0372737	6771.763376	-4.6218	0.0035342	0.023522
i2_LQ_YHS_c51059/f1p1/2251	4.373031655	34.2999749	-2.9715	0.0035373	0.023539
i0_LQ_YHS_c1352/f1p0/894	138.7621532	44.49824916	1.6408	0.003538	0.02354
i2_LQ_YHS_c21924/f1p4/2681	707.5682203	278.0394673	1.3476	0.0035399	0.023549
i1_LQ_YHS_c17459/f1p4/1631	237.33489	51.90894959	2.1929	0.0035463	0.023589
i1_LQ_YHS_c6531/f1p0/1704	324.7345296	1256.372958	-1.9519	0.0035522	0.023624
i2_LQ_YHS_c49528/f1p2/2869	486.4659335	189.8621711	1.3574	0.0035529	0.023626
i5_LQ_YHS_c5557/f1p1/5041	0	9.884488934	-Inf	0.0035548	0.023635
i1_LQ_YHS_c26604/f1p5/1723	1453.54934	21897.84773	-3.9131	0.0035559	0.023639
i2_LQ_YHS_c41941/f1p1/2698	312.5294753	118.8887105	1.3944	0.0035605	0.023667
i2_LQ_YHS_c39733/f1p0/2776	294.5017577	22.10583751	3.7358	0.0035623	0.023675
i5_LQ_YHS_c1505/f1p0/5589	423.7028714	166.4742959	1.3478	0.0035629	0.023676
i1_LQ_YHS_c43998/f1p0/1034	188.2021707	483.0598924	-1.3599	0.003564	0.023681
i1_LQ_YHS_c13824/f1p0/1278	0	9.864465252	-Inf	0.0035668	0.023695
i2_LQ_YHS_c32854/f1p1/2107	174.2618765	1.433854048	6.9252	0.0035708	0.023719
i4_HQ_YHS_c2474/f2p0/4756	0	14.79134557	-Inf	0.0035784	0.023766
i1_LQ_YHS_c35948/f1p0/1923	23.82216734	83.55271106	-1.8104	0.0035808	0.023779
i2_LQ_YHS_c57804/f2p2/2040	96.89675016	26.97995404	1.8446	0.0035818	0.023779
i2_LQ_YHS_c8780/f1p2/2947	96.07717361	261.6102407	-1.4452	0.0035819	0.023779
i2_LQ_YHS_c50325/f1p1/2966	54.69164209	161.674865	-1.5637	0.0035844	0.023793
i1_LQ_YHS_c25326/f1p0/1864	1440.8474	251.4692341	2.5185	0.0035858	0.023799
i4_LQ_YHS_c13225/f1p0/4168	8.513962619	45.44473901	-2.4162	0.0035864	0.0238
i5_LQ_YHS_c5190/f1p1/5896	0.817023896	16.92070068	-4.3723	0.0035884	0.023809
i1_HQ_YHS_c6065/f3p0/1721	15.56923318	70.392732	-2.1767	0.0035895	0.023814
i1_LQ_YHS_c9705/f1p2/1507	206.3837444	65.00727813	1.6667	0.0035912	0.023822
i1_LQ_YHS_c34774/f1p3/1742	42.49335054	133.1654412	-1.6479	0.0035931	0.023829
i3_LQ_YHS_c9254/f1p0/3571	165.1590425	54.58696504	1.5972	0.0035937	0.023829
i4_LQ_YHS_c13767/f1p8/4136	7.353215061	44.1560671	-2.5862	0.0035934	0.023829
i3_LQ_YHS_c8515/f1p0/3489	6.951627488	133.4487712	-4.2628	0.0035972	0.023849
i3_LQ_YHS_c5423/f1p32/3294	1012.6642	119.5301809	3.0827	0.0035994	0.02386
i1_LQ_YHS_c31691/f1p0/1700	1180.346432	429.8149942	1.4574	0.0036052	0.023893
i1_LQ_YHS_c35309/f1p2/1704	162.7580655	54.615915	1.5753	0.0036055	0.023893
i2_LQ_YHS_c26834/f2p0/2189	161.8595862	45.11863118	1.8429	0.0036087	0.023912
i1_LQ_YHS_c5086/f1p0/1476	567.1317439	3445.173723	-2.6028	0.0036134	0.023939
i2_LQ_YHS_c59018/f1p1/2073	47.83619476	8.802848552	2.4421	0.0036149	0.023946
i2_LQ_YHS_c24590/f1p0/2134	89.80745489	242.3853403	-1.4324	0.0036157	0.023948

i2_LQ_YHS_c8355/f1p0/2975	83.30940166	22.33956765	1.8989	0.0036179	0.023959
i4_HQ_YHS_c2188/f3p10/4622	352.5462945	954.0707192	-1.4363	0.0036228	0.023989
i4_LQ_YHS_c4655/f1p2/4370	0	39.91189232	-Inf	0.0036337	0.024057
i2_HQ_YHS_c45991/f37p3/2232	1931.059399	652.7973944	1.5647	0.0036364	0.024072
i3_LQ_YHS_c7275/f1p5/3595	30.71002857	102.1760269	-1.7343	0.0036416	0.024103
i2_LQ_YHS_c50604/f1p0/2696	157.8552783	52.19994621	1.5965	0.0036453	0.024124
i4_LQ_YHS_c14115/f1p0/4074	214.680957	673.5680091	-1.6496	0.0036466	0.024129
i1_HQ_YHS_c17301/f2p0/1447	211.195738	52.20666069	2.0163	0.003651	0.024155
i4_LQ_YHS_c10186/f1p2/4618	0	13.20346332	-Inf	0.0036525	0.024162
i1_LQ_YHS_c10681/f1p2/1683	101.3394672	2.907755461	5.1231	0.0036564	0.024184
i1_HQ_YHS_c17280/f3p3/1982	1537.22469	534.5734231	1.5239	0.0036583	0.024194
i2_HQ_YHS_c5992/f3p0/2576	126.9835811	39.30197425	1.692	0.0036599	0.024201
i3_LQ_YHS_c4675/f1p0/3490	194.2931266	69.03281143	1.4929	0.0036626	0.024216
i1_LQ_YHS_c3797/f1p0/1516	6551.491292	308.9141893	4.4065	0.0036662	0.024233
i6_LQ_YHS_c938/f1p1/6993	0	12.06554204	-Inf	0.0036662	0.024233
i0_LQ_YHS_c1976/f1p5/745	2637.01622	795.3166293	1.7293	0.0036709	0.02426
i2_LQ_YHS_c58445/f1p0/2050	389.6497315	79.75798356	2.2885	0.0036763	0.024293
i3_LQ_YHS_c7396/f1p3/3746	102.6860193	274.5400068	-1.4188	0.0036836	0.024338
i2_LQ_YHS_c27203/f1p2/2198	272.0928802	1089.668382	-2.0017	0.0036863	0.024352
i4_LQ_YHS_c6943/f1p1/4990	115.1860466	310.7837084	-1.4319	0.0036871	0.024354
i3_LQ_YHS_c19519/f1p2/3406	181.0023224	63.30971886	1.5155	0.0036913	0.024379
i2_LQ_YHS_c62075/f194p22/2946	3294.482918	232.3614411	3.8256	0.0036934	0.024389
i1_LQ_YHS_c3506/f1p0/1709	54.60148398	191.5478056	-1.8107	0.0036942	0.024391
i2_LQ_YHS_c51178/f1p2/2818	7.332233693	42.44172107	-2.5332	0.0036968	0.024405
i2_LQ_YHS_c32911/f1p4/2284	366.1055745	123.7138869	1.5653	0.0037008	0.024428
i1_LQ_YHS_c25516/f1p0/1934	53.09194094	156.795932	-1.5623	0.0037112	0.024493
i1_LQ_YHS_c5627/f1p0/1899	136.4800108	44.43150317	1.619	0.0037119	0.024495
i3_LQ_YHS_c6667/f1p0/3878	168.1282099	441.5771787	-1.3931	0.0037167	0.024523
i6_LQ_YHS_c313/f1p0/6712	0.904307434	16.49230219	-4.1888	0.0037189	0.024534
i2_LQ_YHS_c14325/f1p1/2049	163.7866754	418.3783282	-1.353	0.0037207	0.024543
i3_LQ_YHS_c11494/f1p0/3590	394.2696402	157.6657987	1.3223	0.003728	0.024587
i4_LQ_YHS_c1994/f4p3/4327	652.9967998	1942.989663	-1.5731	0.0037294	0.024593
i3_HQ_YHS_c2639/f3p0/3375	622.6698116	241.4981755	1.3665	0.0037325	0.024611
i1_LQ_YHS_c17863/f1p0/1546	20.87092008	0	Inf	0.0037333	0.024612
i7_LQ_YHS_c58/f1p0/7452	1518.527613	6571.198172	-2.1135	0.0037352	0.024621
i3_LQ_YHS_c4181/f1p4/3520	285.6306008	13.61349669	4.391	0.0037358	0.024622
i1_LQ_YHS_c4298/f1p2/1552	1271.585191	43.61866347	4.8655	0.003737	0.024627
i2_LQ_YHS_c4710/f1p0/2426	10.77536309	0	Inf	0.0037378	0.024629
i2_LQ_YHS_c50072/f1p5/2897	1005.918776	162.7763302	2.6276	0.0037392	0.024635
i0_HQ_YHS_c437/f2p0/712	1263.992428	7381.267789	-2.5459	0.0037408	0.024641
i1_HQ_YHS_c6407/f5p9/1891	903.1864413	342.6807496	1.3982	0.003742	0.024646
i1_HQ_YHS_c22718/f2p0/1778	344.8460982	132.859357	1.3761	0.0037438	0.024655
i1_HQ_YHS_c18903/f2p1/1430	1839.816189	620.4650463	1.5681	0.0037546	0.024722
i2_LQ_YHS_c6898/f1p0/2401	42.61292828	6.978392039	2.6103	0.0037617	0.024765
i0_LQ_YHS_c1303/f1p0/615	389.2951067	1102.659072	-1.5021	0.0037764	0.024859
i1_LQ_YHS_c36216/f1p2/1879	54.96906359	11.18647004	2.2969	0.0037846	0.02491
i3_LQ_YHS_c12129/f1p1/3172	326.6655095	28.13059175	3.5376	0.0037874	0.024925
i2_HQ_YHS_c43172/f2p3/2031	171.9513172	522.3168903	-1.6029	0.0037908	0.024943
i3_HQ_YHS_c1600/f4p3/3552	349.5928212	913.6903475	-1.386	0.0037912	0.024943
i3_LQ_YHS_c13061/f1p2/3356	33.0507578	108.4333653	-1.7141	0.0037994	0.024993
i3_LQ_YHS_c4641/f1p2/3239	57.88675287	171.2392876	-1.5647	0.0038012	0.025002
i1_LQ_YHS_c23002/f1p0/1803	3840.968756	1080.760645	1.8294	0.0038073	0.025038
i0_LQ_YHS_c1073/f1p0/915	977.9804118	334.8337111	1.5464	0.0038103	0.025055
i3_LQ_YHS_c11586/f1p4/3611	122.444982	38.33889762	1.6753	0.0038121	0.025063
i1_LQ_YHS_c36162/f1p0/1789	370.2834024	44.63908793	3.0523	0.0038144	0.025074
i2_LQ_YHS_c53499/f1p0/2875	6.994862367	41.72126018	-2.5764	0.0038147	0.025074
i2_LQ_YHS_c12306/f1p0/2965	21.6219721	137.6992281	-2.671	0.0038159	0.025078
i2_LQ_YHS_c14282/f1p0/2046	302.4354261	114.1286946	1.406	0.0038219	0.025114
i1_LQ_YHS_c13577/f1p2/1304	1025.01649	3509.999105	-1.7758	0.0038227	0.025116
i2_LQ_YHS_c37693/f1p5/2512	31.57409539	107.4773564	-1.7672	0.0038235	0.025118
i3_LQ_YHS_c4845/f1p0/3362	2.360446833	29.96673844	-3.6662	0.0038272	0.025138
i2_LQ_YHS_c61946/f10p3/2199	691.3247507	264.3094259	1.3871	0.0038333	0.025172
i2_LQ_YHS_c9368/f1p0/2289	18.05306581	71.90213854	-1.9938	0.0038331	0.025172
i2_LQ_YHS_c27204/f1p4/2196	372.8346598	45.16199568	3.0454	0.0038352	0.025181
i2_LQ_YHS_c8312/f1p2/2647	162.2998443	421.3137701	-1.3762	0.0038358	0.025181
i2_LQ_YHS_c35251/f1p1/2154	179.8691108	460.695444	-1.3569	0.0038394	0.025202
i6_LQ_YHS_c980/f1p0/6768	254.4925726	98.18931588	1.374	0.0038432	0.025223
i1_LQ_YHS_c12536/f1p1/1478	41.10176606	129.2918045	-1.6534	0.003845	0.025228
i2_LQ_YHS_c38581/f1p1/2313	107.0573456	30.8747604	1.7939	0.003845	0.025228
i3_HQ_YHS_c15679/f2p0/3083	126.4104543	334.2550339	-1.4028	0.0038477	0.025243
i2_LQ_YHS_c33865/f1p4/2381	345.8450121	80.84918255	2.0968	0.0038514	0.02526
i2_LQ_YHS_c8506/f1p1/2185	7.983112022	56.31019668	-2.8184	0.0038514	0.02526
i2_LQ_YHS_c51764/f1p2/2071	8.149257589	306.6697424	-5.2339	0.0038533	0.025269
i2_HQ_YHS_c2527/f3p1/2597	294.9753631	103.684327	1.5084	0.003856	0.025283
i2_HQ_YHS_c5602/f4p1/2552	81.37742612	222.3677843	-1.4502	0.0038574	0.025289
i2_LQ_YHS_c62351/f1p17/2285	2256.393919	746.8474368	1.5951	0.0038609	0.025309
i1_LQ_YHS_c2528/f2p1/1953	210.0287495	75.10815833	1.4835	0.0038642	0.025327
i1_LQ_YHS_c17997/f1p0/1634	622.5135369	99.88012112	2.6398	0.0038659	0.025334
i2_HQ_YHS_c32676/f2p0/2529	456.3054367	177.1038919	1.3654	0.0038722	0.025372
i1_LQ_YHS_c28308/f1p0/1299	521.889364	201.1033437	1.3758	0.0038732	0.025375
i2_LQ_YHS_c49411/f1p0/2260	173.0209037	29.26898604	2.5635	0.0038769	0.025396
i2_LQ_YHS_c21994/f1p1/2058	86.70829691	3.209336919	4.7558	0.0038797	0.025411
i2_LQ_YHS_c20897/f1p1/2754	204.2832191	7.35840966	4.795	0.0038862	0.02545
i1_LQ_YHS_c8138/f1p0/1581	284.4645804	105.6581162	1.4288	0.0039007	0.025542
i2_HQ_YHS_c9499/f6p0/2772	75.67924859	205.959567	-1.4444	0.0039044	0.025561
i2_LQ_YHS_c54806/f1p1/2919	84.04502762	230.8395889	-1.4577	0.0039048	0.025561
i3_LQ_YHS_c9812/f1p0/3554	43.44216499	319.0966327	-2.8768	0.0039081	0.02558
i2_LQ_YHS_c57799/f1p6/2503	225.7342502	566.5701432	-1.3276	0.0039113	0.025597
i2_LQ_YHS_c51326/f1p0/2909	128.9382853	40.71746319	1.663	0.003912	0.025598

i4_LQ_YHS_c13219/f1p3/4130	3.699899815	32.56513217	-3.1378	0.0039194	0.025643
i1_LQ_YHS_c35249/f1p8/1679	64.7946955	14.06476298	2.2038	0.0039274	0.025692
i2_LQ_YHS_c41327/f1p18/2488	734.9775862	2083.872803	-1.5035	0.0039342	0.025733
i0_LQ_YHS_c1687/f1p0/917	32.45982931	106.929842	-1.7199	0.003935	0.025735
i0_LQ_YHS_c2779/f1p36/993	14.4219957	64.89645511	-2.1699	0.0039356	0.025735
i2_HQ_YHS_c8011/f2p2/2309	31.80111589	131.7149596	-2.0503	0.0039362	0.025736
i2_LQ_YHS_c54448/f1p0/2893	134.6887631	358.7706416	-1.4134	0.0039377	0.025742
i3_LQ_YHS_c7133/f1p0/3216	160.895828	422.3260538	-1.3922	0.0039402	0.025755
i1_LQ_YHS_c3689/f1p0/1834	63.1766602	181.5456198	-1.5229	0.0039419	0.025762
i2_LQ_YHS_c18066/f1p0/2741	57.51119337	276.4129183	-2.2649	0.0039432	0.025768
i1_LQ_YHS_c17992/f1p1/1597	50.17884836	279.7206756	-2.4788	0.0039499	0.025808
i2_LQ_YHS_c37501/f1p0/2352	77.24364452	213.7007988	-1.4681	0.0039508	0.02581
i3_LQ_YHS_c19912/f1p0/3655	51.6103264	157.6481857	-1.611	0.003952	0.025815
i2_LQ_YHS_c35542/f1p33/2404	14022.16313	514.395843	4.7687	0.0039573	0.025846
i2_LQ_YHS_c9827/f1p5/2972	690.6612754	97.23291418	2.8285	0.0039655	0.025896
i2_LQ_YHS_c51277/f1p0/3004	290.5944353	17.6440859	4.0418	0.0039666	0.0259
i2_LQ_YHS_c56390/f1p0/2684	41.59397849	132.3191895	-1.6696	0.0039762	0.025958
i1_LQ_YHS_c36686/f1p5/1763	13.68844729	0.353872233	5.2736	0.0039912	0.026053
i1_LQ_YHS_c28626/f1p0/1082	1678.17383	9018.357366	-2.426	0.0039931	0.026062
i3_LQ_YHS_c13991/f1p0/3060	0	9.839385654	-Inf	0.0039955	0.026074
i1_LQ_YHS_c32158/f1p0/1814	165.8862709	421.1922485	-1.3443	0.0039971	0.026081
i1_HQ_YHS_c2295/f3p0/1830	194.769384	625.5391518	-1.6833	0.0040003	0.026098
i4_LQ_YHS_c3422/f1p0/4765	3.086845874	108.0679968	-5.1297	0.0040052	0.026127
i2_LQ_YHS_c23933/f1p3/2156	58.53049858	12.42612875	2.2358	0.0040065	0.026131
i3_LQ_YHS_c13680/f1p0/3089	0	9.819361971	-Inf	0.0040087	0.026142
i2_LQ_YHS_c49164/f1p4/2464	976.9664885	3272.16266	-1.7439	0.0040105	0.026151
i2_LQ_YHS_c33017/f1p0/2505	108.8460155	25.90550115	2.071	0.0040166	0.026187
i6_HQ_YHS_c186/f2p0/6159	0	28030.92801	-Inf	0.0040188	0.026198
i2_LQ_YHS_c50871/f1p1/2392	0	57.84112616	-Inf	0.0040196	0.026199
i4_LQ_YHS_c2547/f2p0/4492	0	9.800996855	-Inf	0.0040208	0.026204
i1_LQ_YHS_c26481/f1p0/1620	874.6805152	2606.820641	-1.5755	0.0040231	0.026215
i2_LQ_YHS_c24275/f1p0/2563	333.2557876	872.7682853	-1.389	0.0040278	0.026242
i1_LQ_YHS_c23842/f1p0/1745	55.70388414	162.5790166	-1.5453	0.0040296	0.026243
i3_LQ_YHS_c6601/f1p0/3246	123.1683784	38.42124369	1.6807	0.0040294	0.026243
i6_LQ_YHS_c1053/f1p7/6151	24.13358842	90.17000382	-1.9016	0.0040296	0.026243
i1_LQ_YHS_c26419/f1p2/1856	149.4256995	49.91316555	1.5819	0.004032	0.026245
i2_HQ_YHS_c2777/f5p2/2747	102.5681972	269.6654161	-1.3946	0.0040311	0.026245
i2_LQ_YHS_c23517/f1p342/2361	46.99645062	152.9412126	-1.7024	0.0040316	0.026245
i2_LQ_YHS_c28174/f1p0/2044	45.08082624	139.0960789	-1.6255	0.0040315	0.026245
i2_LQ_YHS_c10703/f1p2/2168	549.9842924	1510.643111	-1.4577	0.0040354	0.026264
i1_LQ_YHS_c10784/f1p3/1531	1.331498208	19.56253912	-3.877	0.0040409	0.026296
i1_LQ_YHS_c33470/f1p3/1895	148.5493242	0	Inf	0.0040443	0.026314
i5_LQ_YHS_c1263/f1p0/5937	127.6651177	330.5728704	-1.3726	0.0040454	0.026318
i2_LQ_YHS_c29080/f1p0/2018	69.6390779	193.252393	-1.4725	0.0040466	0.026322
i6_LQ_YHS_c315/f1p0/6100	16.21503969	67.62403698	-2.0602	0.0040489	0.026334
i2_LQ_YHS_c24608/f1p4/2387	77.97641264	226.22333	-1.5366	0.004052	0.02635
i2_LQ_YHS_c7929/f1p1/2149	45.01662674	0.713866171	5.9787	0.0040547	0.026364
i3_LQ_YHS_c20873/f1p4/3031	0	9.742584375	-Inf	0.0040597	0.026393
i1_LQ_YHS_c8720/f3p0/1615	182.9028343	466.1765147	-1.3498	0.0040625	0.026408
i5_LQ_YHS_c3423/f1p1/5860	47.51518297	153.1470586	-1.6885	0.004064	0.026414
i1_LQ_YHS_c31715/f1p0/1741	15.91963948	67.60235473	-2.0863	0.0040661	0.026421
i2_LQ_YHS_c50747/f1p0/2342	858.0113664	57.39910477	3.9019	0.0040662	0.026421
i2_LQ_YHS_c28902/f1p1/2044	27.97403898	98.5427151	-1.8167	0.0040679	0.026429
i3_LQ_YHS_c18940/f1p0/3754	86.88920794	1.079981815	6.3301	0.004069	0.026432
i2_HQ_YHS_c60733/f23p6/2290	457.0687877	1220.469695	-1.417	0.0040699	0.026435
i2_LQ_YHS_c36518/f1p0/2608	0	9.722560692	-Inf	0.004073	0.026451
i2_LQ_YHS_c10246/f1p0/2163	8.25798923	46.69383656	-2.4994	0.0040748	0.02646
i3_LQ_YHS_c12887/f1p1/3255	438.2126305	169.8595026	1.3673	0.0040778	0.026475
i2_HQ_YHS_c30089/f26p3/2767	351.042758	138.7621502	1.339	0.004082	0.026488
i2_HQ_YHS_c62018/f124p6/2245	1173.527839	4284.335979	-1.8682	0.0040817	0.026488
i3_LQ_YHS_c8550/f1p3/3350	10.63227644	0	Inf	0.004081	0.026488
i6_LQ_YHS_c421/f1p0/6984	4.266835923	32.8082616	-2.9428	0.0040812	0.026488
i1_HQ_YHS_c5229/f2p0/1876	45.68939479	6.077045015	2.9104	0.0040831	0.026492
i6_LQ_YHS_c533/f1p0/6084	0	9.705854143	-Inf	0.0040842	0.026496
i5_LQ_YHS_c1902/f1p0/5340	0	9.698073871	-Inf	0.0040895	0.026526
i1_LQ_YHS_c13664/f1p1/1297	105.425945	31.62867394	1.7369	0.0040987	0.026582
i0_LQ_YHS_c1497/f1p3/769	36.20931633	5.613535316	2.6894	0.0040994	0.026583
i4_LQ_YHS_c14045/f1p0/4038	540.0928156	1565.265295	-1.5351	0.0041	0.026583
i2_HQ_YHS_c3714/f4p0/2844	127.7657582	329.7566718	-1.3679	0.0041022	0.026594
i1_LQ_YHS_c26596/f1p3/1811	0	9.671928483	-Inf	0.0041071	0.026622
i2_LQ_YHS_c12993/f1p0/2662	0	9.665806778	-Inf	0.0041112	0.026646
i1_LQ_YHS_c5980/f1p0/1839	329.3641031	831.8587617	-1.3367	0.0041167	0.026675
i2_LQ_YHS_c9781/f1p0/2483	0	36.66175483	-Inf	0.0041168	0.026675
i1_HQ_YHS_c1152/f4p0/1907	16976.0061	0	Inf	0.0041181	0.02668
i3_LQ_YHS_c11777/f1p0/3727	0.359624837	12.43390902	-5.1116	0.0041194	0.026684
i3_LQ_YHS_c10799/f1p0/3434	207.9438907	49.73453075	2.0639	0.0041234	0.026707
i2_HQ_YHS_c18689/f4p1/2293	473.0666801	36.57004901	3.6933	0.0041277	0.026731
i2_LQ_YHS_c43358/f1p1/2041	79.12191125	214.7083069	-1.4402	0.00413	0.026742
i3_LQ_YHS_c17929/f1p1/3536	1426.111104	69.46278826	4.3597	0.0041316	0.026749
i0_LQ_YHS_c2070/f1p1/686	2228.483823	730.6769743	1.6088	0.0041367	0.026779
i2_LQ_YHS_c4638/f1p5/2650	676.3897419	266.1158705	1.3458	0.0041382	0.026784
i3_LQ_YHS_c16774/f1p0/3172	0	36.68011994	-Inf	0.0041395	0.026789
i0_LQ_YHS_c3547/f1p29/259	0	9.619637708	-Inf	0.0041424	0.026805
i4_LQ_YHS_c9952/f1p1/4858	160.91702	12.50290635	3.686	0.0041477	0.026836
i4_LQ_YHS_c10168/f1p588/4194	0.801919753	16.36710418	-4.3512	0.004152	0.026859
i3_LQ_YHS_c8287/f1p2/3432	402.4514648	139.8373156	1.5251	0.0041578	0.026894
i2_LQ_YHS_c4220/f1p3/2974	38.84497067	268.6253976	-2.7898	0.0041592	0.026896
i5_LQ_YHS_c2688/f1p3/5548	307.5343817	116.6008234	1.3992	0.0041593	0.026896
i4_LQ_YHS_c11890/f1p0/4904	4.373837061	209.2105274	-5.5799	0.0041627	0.026914

i3_LQ_YHS_c16962/f1p0/3835	0	9.589029181	-Inf	0.0041633	0.026915
i1_LQ_YHS_c20591/f1p0/1821	86.87292258	23.76058551	1.8703	0.0041655	0.026923
i3_LQ_YHS_c16936/f1p0/3125	384.4593888	1007.772411	-1.3903	0.0041657	0.026923
i2_LQ_YHS_c27184/f1p1/2869	23.68749383	2.276788592	3.3791	0.0041792	0.027006
i2_LQ_YHS_c58709/f1p0/2271	611.2310785	41.81699567	3.8696	0.0041796	0.027006
i3_LQ_YHS_c18540/f1p2/3090	65.5842864	186.9440296	-1.5112	0.0041949	0.027101
i2_LQ_YHS_c51747/f1p3/2252	14.51007627	65.87848964	-2.1828	0.0041974	0.027114
i3_HQ_YHS_c21536/f4p1/3043	472.0505773	1305.385356	-1.4675	0.0041987	0.027118
i5_LQ_YHS_c4509/f1p9/5187	424.7360685	156.3954166	1.4414	0.0042118	0.027199
i2_LQ_YHS_c41282/f1p1/2147	129.7016171	9.127770825	3.8288	0.0042125	0.0272
i7_LQ_YHS_c118/f1p0/7613	19.80714133	77.37444117	-1.9658	0.004219	0.027238
i1_LQ_YHS_c10503/f1p0/1720	107.8962759	32.40261115	1.7355	0.0042263	0.027282
i3_LQ_YHS_c19797/f1p0/3423	114.8812508	5.401567642	4.4106	0.0042273	0.027284
i1_LQ_YHS_c12444/f1p0/1966	28.15610247	93.45556511	-1.7308	0.0042319	0.027311
i4_LQ_YHS_c5108/f1p0/4938	43.12945502	135.4075113	-1.6506	0.0042383	0.027348
i1_LQ_YHS_c10219/f1p0/1892	248.8769501	629.1459364	-1.338	0.0042396	0.027353
i2_LQ_YHS_c35284/f1p1/2193	189.8754922	482.3568099	-1.345	0.0042456	0.027384
i2_LQ_YHS_c51346/f1p7/2201	228.1190318	85.69434912	1.4125	0.0042453	0.027384
i2_LQ_YHS_c64655/f1p0/2002	122.6299117	38.54201923	1.6698	0.0042573	0.027456
i1_LQ_YHS_c21411/f1p5/1885	246.5102132	67.71305799	1.8641	0.0042583	0.027459
i2_LQ_YHS_c56541/f1p1/2308	3.237887298	27.81735965	-3.1029	0.0042645	0.027496
i2_LQ_YHS_c25829/f1p5/2718	18.13989099	73.72260493	-2.0229	0.0042677	0.027509
i2_LQ_YHS_c53057/f1p2/2466	14.31199192	171.5469561	-3.5833	0.0042675	0.027509
i4_LQ_YHS_c8568/f1p0/4831	0	16.72038364	-Inf	0.0042739	0.027545
i1_HQ_YHS_c39925/f2p1/1859	668.1936724	264.4556738	1.3372	0.0042749	0.027548
i3_LQ_YHS_c20867/f1p0/3121	26.82887904	3.009019879	3.1564	0.0042756	0.027548
i3_LQ_YHS_c14147/f1p0/3032	0	13.64682957	-Inf	0.0042831	0.027593
i1_LQ_YHS_c28782/f1p0/1309	330.4607206	845.2177514	-1.3548	0.0042855	0.027605
i4_LQ_YHS_c8288/f1p0/4529	82.21187945	11.15807331	2.8813	0.0042891	0.027624
i2_LQ_YHS_c4111/f1p5/2843	3.344888437	27.27422824	-3.0275	0.0042897	0.027625
i2_LQ_YHS_c19274/f1p0/2242	110.4995247	285.0786836	-1.3673	0.0042916	0.027633
i4_LQ_YHS_c3615/f1p0/4462	35.02852088	172.3546992	-2.2988	0.0042924	0.027634
i2_LQ_YHS_c10387/f1p1/2510	64.58161832	14.60670884	2.1445	0.0042973	0.027663
i2_HQ_YHS_c41793/f2p0/2470	76.55683386	2.84934298	4.7478	0.0042981	0.027664
i2_LQ_YHS_c56427/f1p0/2230	135.9607866	352.0655518	-1.3727	0.0043007	0.027677
i3_HQ_YHS_c21575/f152p0/3451	8951.293391	986.498991	3.1817	0.0043101	0.027734
i4_LQ_YHS_c13687/f1p25/4207	10.84880625	54.59087495	-2.3311	0.004317	0.027775
i1_LQ_YHS_c10942/f1p39/1933	16.40057256	0	Inf	0.0043189	0.027783
i1_LQ_YHS_c38733/f1p3/1312	303.2662784	738.5836877	-1.2842	0.004327	0.027821
i2_LQ_YHS_c13030/f1p0/2593	27.68118304	3.259969128	3.086	0.0043265	0.027821
i4_LQ_YHS_c4927/f1p0/4681	373.2945085	29.18158405	3.6772	0.004326	0.027821
i5_LQ_YHS_c1364/f1p0/5168	157.6154371	55.38313774	1.5089	0.0043266	0.027821
i1_HQ_YHS_c2176/f3p3/1985	189.9483334	459.9467446	-1.2759	0.0043383	0.027889
i1_LQ_YHS_c8224/f1p0/2016	600.8093398	1673.493381	-1.4779	0.0043434	0.027919
i2_HQ_YHS_c37273/f2p4/2388	138.5824402	45.62052968	1.603	0.0043457	0.027922
i2_LQ_YHS_c36003/f1p1/2697	23.25361205	86.24620796	-1.891	0.0043452	0.027922
i2_LQ_YHS_c59983/f1p0/2052	0	9.326429299	-Inf	0.0043449	0.027922
i2_LQ_YHS_c48411/f2p1/2998	315.5847629	119.7957062	1.3975	0.0043491	0.02794
i4_LQ_YHS_c6333/f1p0/4643	0.272341299	12.22581171	-5.4884	0.0043523	0.027957
i1_LQ_YHS_c42104/f1p0/1450	13237.10537	1.112248908	13.539	0.0043543	0.027966
i2_LQ_YHS_c26510/f1p0/2751	4.925669026	38.90205271	-2.9815	0.0043573	0.027974
i2_LQ_YHS_c28208/f1p0/2079	67.74054064	177.4384123	-1.3892	0.0043567	0.027974
i2_LQ_YHS_c38049/f1p1/2616	170.6190471	434.6876126	-1.3492	0.0043563	0.027974
i0_LQ_YHS_c2815/f1p5/817	347.0490567	134.4823505	1.3677	0.0043595	0.027985
i3_LQ_YHS_c14288/f1p1/3071	0	14.38684113	-Inf	0.0043621	0.027998
i4_HQ_YHS_c14546/f5p1/4249	410.5470831	159.8827953	1.3605	0.0043704	0.028047
i3_LQ_YHS_c18445/f1p5/3598	27.27531231	94.64340606	-1.7949	0.004377	0.028086
i2_LQ_YHS_c43167/f2p1/2041	247.2633763	42.3187744	2.5467	0.0043881	0.028153
i3_LQ_YHS_c18746/f1p0/3047	216.204052	78.30635717	1.4652	0.0043927	0.028179
i2_LQ_YHS_c8989/f4p1/2488	152.2548415	51.71262268	1.5579	0.0043947	0.028188
i3_LQ_YHS_c8328/f1p1/3167	351.8449745	919.6772545	-1.3862	0.0043998	0.028217
i1_LQ_YHS_c18323/f1p1/1774	346.6894942	100.2943039	1.7894	0.0044005	0.028218
i3_LQ_YHS_c9516/f1p0/3320	1.634047791	20.71929853	-3.6645	0.0044165	0.028317
i1_LQ_YHS_c32943/f1p24/1785	124.0159743	38.25931545	1.6966	0.0044193	0.028331
i5_LQ_YHS_c4188/f1p1/5924	1.263932271	17.60735567	-3.8002	0.0044223	0.028346
i4_LQ_YHS_c3582/f1p2/4627	11.36742729	55.1444319	-2.2783	0.0044246	0.028357
i3_HQ_YHS_c15239/f17p0/3532	705.1689821	2069.725822	-1.5534	0.0044269	0.028368
i0_LQ_YHS_c2100/f1p0/738	1533.627338	546.5949672	1.4884	0.0044312	0.028392
i1_LQ_YHS_c35743/f1p2/1628	29.10364478	139.6339138	-2.2624	0.0044328	0.028399
i2_LQ_YHS_c39330/f1p0/2533	0	9.833263948	-Inf	0.004443	0.028456
i3_LQ_YHS_c14852/f1p2/3033	30.97396511	0	Inf	0.0044423	0.028456
i4_HQ_YHS_c1926/f4p0/4663	2779.347932	355.6439276	2.9662	0.0044449	0.028465
i2_HQ_YHS_c62507/f3p14/2309	402.9010287	1046.321875	-1.3768	0.0044514	0.028502
i2_LQ_YHS_c64497/f1p0/2022	271.64416	680.6490032	-1.3252	0.0044557	0.028523
i2_LQ_YHS_c64686/f1p1/2008	244.2131689	608.1729605	-1.3163	0.0044557	0.028523
i2_HQ_YHS_c20859/f2p1/2711	314.4781359	1105.827767	-1.8141	0.0044569	0.028527
i3_LQ_YHS_c4539/f1p0/3304	145.3702964	370.4350408	-1.3495	0.0044604	0.028545
i1_LQ_YHS_c35965/f1p20/1847	273.2948498	19.65215291	3.7977	0.0044687	0.028591
i2_HQ_YHS_c1587/f12p4/2954	633.0533502	254.2001738	1.3164	0.0044686	0.028591
i2_LQ_YHS_c9174/f1p3/2859	5.693572443	39.77444978	-2.8044	0.0044696	0.028593
i2_HQ_YHS_c63612/f2p17/2406	1697.455809	47.05229282	5.173	0.004476	0.02863
i3_LQ_YHS_c8211/f1p0/3888	23.91037597	0	Inf	0.0044803	0.028654
i2_HQ_YHS_c45462/f5p4/2323	2774.408943	875.4703552	1.6641	0.0044924	0.028727
i2_LQ_YHS_c6151/f1p3/2251	1430.765028	524.8321034	1.4469	0.0044998	0.028771
i1_LQ_YHS_c28454/f1p0/1208	1720.893979	621.4350412	1.4695	0.0045026	0.028785
i3_HQ_YHS_c13052/f2p3/3631	1296.834971	121.949547	3.4106	0.0045181	0.02888
i3_HQ_YHS_c21456/f2p0/3530	47.95528903	177.8117177	-1.8906	0.0045212	0.028896
i4_LQ_YHS_c8782/f1p0/4715	0	2213.807965	-Inf	0.0045275	0.028932
i4_LQ_YHS_c6605/f1p0/4615	30.85945918	102.1872045	-1.7274	0.0045293	0.02894

i1_LQ_YHS_c23046/f1p0/1517	417.4207579	163.8854608	1.3488	0.0045307	0.028945
i1_LQ_YHS_c4178/f1p0/1794	976.1757882	377.4781214	1.3707	0.0045361	0.028976
i1_LQ_YHS_c36007/f1p1/1972	88.93268677	12.93189761	2.7818	0.0045419	0.029009
i1_HQ_YHS_c20884/f2p3/1637	436.5864082	1132.324691	-1.3749	0.0045531	0.029077
i2_HQ_YHS_c7254/f4p5/3016	123.2394879	313.2192674	-1.3457	0.0045544	0.029081
i0_LQ_YHS_c2798/f1p1/787	4611.394008	1219.819198	1.9185	0.004557	0.02909
i1_LQ_YHS_c20746/f1p0/1610	3.264754267	27.77506095	-3.0887	0.0045566	0.02909
i2_LQ_YHS_c34870/f1p0/2375	29.60683975	0	Inf	0.004558	0.029093
i2_LQ_YHS_c28371/f1p5/2075	202.1563257	507.0916101	-1.3268	0.004559	0.029096
i4_LQ_YHS_c5190/f1p1/4426	4.997848422	34.20704398	-2.7749	0.0045607	0.029102
i2_HQ_YHS_c45667/f2p3/2150	266.4429113	686.2690097	-1.3649	0.0045617	0.029105
i1_LQ_YHS_c13477/f1p0/1130	106.5263166	289.0102213	-1.4399	0.0045629	0.029107
i3_LQ_YHS_c14103/f1p0/3098	63.06968418	14.19383135	2.1517	0.0045633	0.029107
i2_LQ_YHS_c55575/f1p4/2646	164.6097046	419.8419596	-1.3508	0.0045648	0.029113
i3_LQ_YHS_c18884/f1p0/3505	0.272341299	11.88643423	-5.4478	0.0045685	0.029129
i3_LQ_YHS_c5226/f1p10/3361	53.42796595	0	Inf	0.0045684	0.029129
i1_LQ_YHS_c23146/f1p1/1962	473.4784495	21.4905114	4.4615	0.0045697	0.029133
i2_LQ_YHS_c55131/f1p2/2181	158.0865568	54.86793004	1.5267	0.0045711	0.029138
i2_LQ_YHS_c44070/f1p1/2022	142.9516889	21.57613505	2.728	0.0045724	0.029142
i3_LQ_YHS_c2022/f3p1/3484	366.8858691	107.4786222	1.7713	0.0045746	0.029152
i1_LQ_YHS_c11627/f1p7/1927	29.16451971	100.5686347	-1.7859	0.0045782	0.02917
i2_HQ_YHS_c25265/f2p6/2252	206.9325319	511.97161	-1.3069	0.0045786	0.02917
i2_HQ_YHS_c36997/f2p3/2933	47.92841368	142.572558	-1.5727	0.0045805	0.029178
i2_LQ_YHS_c24630/f1p7/2429	39.74147652	0.719987877	5.7865	0.0045832	0.029192
i2_LQ_YHS_c10684/f2p2/2770	222.1397072	562.4656634	-1.3403	0.0045904	0.029234
i2_HQ_YHS_c13409/f6p1/2557	71.76485464	900.9561428	-3.6501	0.0045913	0.029236
i1_LQ_YHS_c9603/f1p1/1656	231.1365022	680.788023	-1.5585	0.0045997	0.029285
i1_LQ_YHS_c41444/f1p0/1969	74.36784852	0	Inf	0.0046023	0.029298
i2_LQ_YHS_c20024/f1p5/2949	482.6208037	165.9146173	1.5404	0.0046076	0.029328
i2_HQ_YHS_c60900/f42p2/2153	605.8772937	44.08154085	3.7808	0.0046101	0.02934
i2_LQ_YHS_c18780/f1p0/2096	39.10394671	123.2648384	-1.6564	0.0046162	0.029375
i1_HQ_YHS_c2035/f4p0/1518	938.8177324	361.193877	1.3781	0.0046196	0.029393
i2_HQ_YHS_c40864/f2p0/2513	107.7284166	11.9326033	3.1744	0.0046218	0.029403
i1_LQ_YHS_c20267/f1p0/1734	451.7790957	1158.621389	-1.3587	0.0046279	0.029431
i1_LQ_YHS_c3417/f2p4/1820	1190.818809	4330.487673	-1.8626	0.004628	0.029431
i3_HQ_YHS_c2137/f2p1/3651	414.835495	1090.435533	-1.3943	0.0046268	0.029431
i2_LQ_YHS_c33701/f1p2/2138	17.90028551	1.176783094	3.9271	0.00463	0.029439
i3_LQ_YHS_c5244/f1p0/3517	9.47741448	48.00521695	-2.3406	0.0046323	0.02945
i2_LQ_YHS_c51951/f1p2/2405	269.8990313	102.8514252	1.3919	0.0046332	0.029452
i1_LQ_YHS_c18770/f1p1/1800	8.498391741	91.79766041	-3.4332	0.0046346	0.029457
i4_HQ_YHS_c2272/f2p0/4830	41.18375042	133.7068338	-1.6989	0.0046362	0.029463
i3_LQ_YHS_c18734/f1p0/3129	58.67485738	12.52632738	2.2278	0.0046379	0.02947
i1_HQ_YHS_c8043/f5p0/1893	663.6221434	261.0170292	1.3462	0.0046418	0.029484
i2_LQ_YHS_c18618/f1p0/2857	94.98033712	27.61479127	1.7822	0.0046416	0.029484
i2_LQ_YHS_c42128/f1p0/2748	71.22018879	197.0497658	-1.4682	0.0046418	0.029484
i1_LQ_YHS_c32118/f1p0/1696	8.396004061	43.3943733	-2.3697	0.0046457	0.029497
i2_LQ_YHS_c27349/f1p1/2567	171.1969825	428.3596123	-1.3232	0.0046449	0.029497
i2_LQ_YHS_c45051/f1p0/2015	50.67949067	150.3950305	-1.5693	0.0046456	0.029497
i1_LQ_YHS_c21739/f1p0/1861	494.6612454	1324.63546	-1.4211	0.0046524	0.029536
i2_LQ_YHS_c11818/f1p0/2767	40.29745521	4.096781966	3.2981	0.0046632	0.0296
i2_LQ_YHS_c35224/f1p0/2144	90.98711464	25.45988356	1.8374	0.0046651	0.029608
i3_LQ_YHS_c10280/f2p3/3499	458.515474	110.4797013	2.0532	0.0046846	0.029728
i3_LQ_YHS_c3578/f1p2/3068	190.6945362	69.98261954	1.4462	0.0046908	0.029764
i5_LQ_YHS_c1533/f1p0/5613	51.27457426	149.1519745	-1.5405	0.0046942	0.029781
i0_LQ_YHS_c2055/f1p0/598	1443.507988	505.3729542	1.5142	0.0046989	0.029807
i3_LQ_YHS_c8050/f1p4/3855	179.111071	63.0515019	1.5063	0.0047003	0.029812
i1_LQ_YHS_c28413/f1p0/1091	1853.155454	11576.47166	-2.6431	0.004703	0.029821
i2_LQ_YHS_c5425/f1p14/2718	4698.811674	870.3166543	2.4327	0.0047026	0.029821
i1_LQ_YHS_c17127/f2p0/1944	61.83847936	174.1465689	-1.4937	0.0047048	0.029829
i2_HQ_YHS_c60078/f6p2/2331	701.8083983	2043.785753	-1.5421	0.0047062	0.029834
i2_LQ_YHS_c35842/f1p7/2702	26.67737926	0	Inf	0.0047106	0.029857
i1_LQ_YHS_c9549/f2p0/1797	324.7011523	810.6914349	-1.32	0.0047115	0.02986
i2_LQ_YHS_c44430/f1p0/2026	22.23422063	81.89452616	-1.881	0.0047154	0.02988
i2_LQ_YHS_c36182/f1p1/2462	15.52647341	0	Inf	0.0047182	0.029894
i5_LQ_YHS_c3128/f1p3/5260	62.39892669	430.2297589	-2.7855	0.0047203	0.029903
i3_LQ_YHS_c3826/f1p0/3187	2.390655117	24.24577745	-3.3423	0.004732	0.029974
i2_LQ_YHS_c20068/f1p58/2799	39.17691476	257.95292	-2.719	0.004735	0.029989
i2_LQ_YHS_c19689/f1p15/2507	3243.448362	1009.215109	1.6843	0.0047376	0.029994
i2_LQ_YHS_c38964/f1p0/2351	72.85679761	18.74349862	1.9587	0.0047376	0.029994
i5_LQ_YHS_c893/f1p0/5921	75.36793882	19.78734298	1.9294	0.0047364	0.029994
i2_HQ_YHS_c12204/f2p0/2322	0	9.465489738	-Inf	0.004746	0.030043
i1_LQ_YHS_c5849/f1p0/1868	0	12.91906142	-Inf	0.0047482	0.030053
i3_LQ_YHS_c16769/f1p0/3070	25.76255602	104.9871009	-2.0269	0.0047515	0.030069
i1_LQ_YHS_c9923/f1p0/1762	2.353297465	73.89831539	-4.9728	0.0047552	0.030089
i1_LQ_YHS_c20715/f1p0/1450	45.05475631	137.4113152	-1.6087	0.0047572	0.030095
i1_LQ_YHS_c6841/f1p8/1559	185.7050063	66.06218035	1.4911	0.0047579	0.030095
i2_LQ_YHS_c40100/f1p1/2781	588.9852075	27.23196908	4.4349	0.0047579	0.030095
i1_HQ_YHS_c2125/f5p0/1758	433.0983159	1718.310964	-1.9882	0.00476	0.030104
i3_LQ_YHS_c11105/f1p0/3194	41.6188014	6.797032892	2.6143	0.0047614	0.030108
i1_LQ_YHS_c3858/f1p0/1423	99.55387772	254.2752114	-1.3528	0.0047648	0.030122
i4_LQ_YHS_c11452/f1p2/4787	5.144743123	106.6334747	-4.3734	0.0047648	0.030122
i4_LQ_YHS_c13610/f1p0/4081	19.09077462	69.96931033	-1.8738	0.0047755	0.030186
i3_LQ_YHS_c18969/f1p0/3798	71.14099646	18.77908285	1.9216	0.0047765	0.030188
i2_LQ_YHS_c54582/f1p0/2550	96.07848762	12.16293609	2.9817	0.0047792	0.030202
i2_LQ_YHS_c25764/f1p2/2214	488.9367527	25.96896955	4.2348	0.0047815	0.030209
i3_LQ_YHS_c12946/f1p1/3201	0	12.90681801	-Inf	0.0047817	0.030209
i3_HQ_YHS_c2316/f2p2/3335	425.0441338	165.4575095	1.3612	0.0047835	0.030216
i2_LQ_YHS_c41881/f1p13/2504	4903.53819	296.7153553	4.0467	0.0047852	0.03022
i2_LQ_YHS_c50872/f1p0/2772	204.4047619	517.209349	-1.3393	0.0047852	0.03022

i1_LQ_YHS_c5283/f1p0/1619	5.201351641	37.90216563	-2.8653	0.0047861	0.030221
i3_LQ_YHS_c4033/f1p0/3398	0	9.400955552	-Inf	0.0047949	0.030273
i2_LQ_YHS_c25285/f1p0/2247	187.5678339	34.49472347	2.443	0.0047995	0.030298
i2_LQ_YHS_c27124/f1p0/2805	387.9991998	65.8028976	2.5598	0.0048114	0.030369
i2_LQ_YHS_c9973/f1p1/2815	68.93431245	587.3322431	-3.0909	0.0048128	0.030374
i4_LQ_YHS_c7863/f1p0/4673	69.24624213	17.22733805	2.007	0.0048162	0.030391
i2_LQ_YHS_c37844/f1p2/2837	3.177470728	28.04484832	-3.1418	0.0048213	0.03042
i3_LQ_YHS_c16960/f1p0/3827	10.86011072	50.10013384	-2.2058	0.004823	0.030426
i2_HQ_YHS_c62585/f2p1/2402	2355.423337	790.4760923	1.5752	0.0048261	0.030442
i1_LQ_YHS_c6955/f1p1/1457	690.6678395	220.4499657	1.6475	0.0048315	0.030472
i1_LQ_YHS_c14671/f1p0/1264	179.7333351	65.36604697	1.4592	0.0048352	0.030487
i4_LQ_YHS_c10072/f1p0/4298	203.5134894	76.00737378	1.4209	0.004835	0.030487
i3_LQ_YHS_c9410/f1p0/3573	0	9.336421366	-Inf	0.0048439	0.030538
i3_LQ_YHS_c7800/f1p15/3245	29.70887201	101.3858968	-1.7709	0.0048483	0.030562
i1_LQ_YHS_c8267/f1p0/1895	42.78379025	134.5080229	-1.6526	0.004851	0.030575
i2_LQ_YHS_c49736/f1p1/2375	26.96560498	94.26954969	-1.8057	0.0048523	0.030579
i2_HQ_YHS_c9163/f5p0/2669	51.56423369	7.402920163	2.8002	0.0048564	0.030597
i2_LQ_YHS_c13536/f1p3/2453	386.7770759	993.1457189	-1.3605	0.0048562	0.030597
i1_LQ_YHS_c17716/f1p3/1839	171.2746921	430.7929607	-1.3307	0.0048595	0.030609
i4_LQ_YHS_c8360/f1p0/4890	13.43167687	60.49418178	-2.1712	0.0048595	0.030609
i0_LQ_YHS_c1790/f1p1/953	11.43833454	71030.85315	-12.6	0.0048632	0.030618
i3_LQ_YHS_c19248/f1p0/3657	31.99620598	106.5164113	-1.7351	0.0048635	0.030618
i4_LQ_YHS_c10770/f1p0/5003	72.63403515	18.74740853	1.954	0.0048624	0.030618
i5_LQ_YHS_c4895/f1p2/5129	47.05789523	0	Inf	0.0048616	0.030618
i2_LQ_YHS_c39457/f1p0/2341	198.7924928	72.52923505	1.4546	0.0048647	0.030621
i3_LQ_YHS_c8557/f1p0/3446	253.5477408	73.92631932	1.7781	0.0048655	0.030623
i1_LQ_YHS_c12195/f1p8/1746	8.768655491	46.82179959	-2.4168	0.0048718	0.030655
i1_LQ_YHS_c8342/f1p1/1896	0	9.299691134	-Inf	0.0048719	0.030655
i0_LQ_YHS_c2988/f1p0/963	404.208355	1.079981815	8.5479	0.0048751	0.030669
i2_LQ_YHS_c22219/f1p5/2198	146.6867491	38.09904577	1.9449	0.0048754	0.030669
i3_LQ_YHS_c9826/f1p5/3201	87.89757129	233.1588366	-1.4074	0.0048762	0.03067
i1_LQ_YHS_c33566/f1p4/1869	257.1059003	614.0942397	-1.2561	0.0048839	0.030714
i1_LQ_YHS_c24960/f1p0/1443	55.35856641	10.74578859	2.365	0.004886	0.030724
i2_LQ_YHS_c18347/f1p1/2795	97.93320357	28.53616198	1.779	0.0048867	0.030724
i4_LQ_YHS_c15700/f1p3/4008	44.20340252	3.589354539	3.6224	0.0048885	0.030731
i3_LQ_YHS_c5477/f1p1/3301	39.3537042	122.4179138	-1.6372	0.0048916	0.030747
i1_LQ_YHS_c22744/f1p2/1926	886.3349249	344.4250718	1.3637	0.0048933	0.03075
i2_HQ_YHS_c23760/f3p0/2226	152.55393	377.9770549	-1.309	0.0048934	0.03075
i2_HQ_YHS_c32432/f4p8/2618	524.893334	1460.628311	-1.4765	0.0048941	0.03075
i2_LQ_YHS_c33352/f1p1/2168	43.50292023	150.6533266	-1.792	0.0048973	0.030767
i1_LQ_YHS_c10304/f1p0/1863	425.7669881	1102.703688	-1.3729	0.0049018	0.030787
i1_LQ_YHS_c34482/f1p1/1386	0	9.261302336	-Inf	0.0049013	0.030787
i4_LQ_YHS_c11779/f1p3/4497	18.76643826	1.079981815	4.1191	0.0049177	0.030883
i1_LQ_YHS_c17594/f1p1/1469	62.05121787	170.0805163	-1.4547	0.0049199	0.030893
i3_HQ_YHS_c1088/f10p0/3638	791.30111	268.9231137	1.557	0.0049232	0.030909
i1_LQ_YHS_c33224/f1p1/1588	422.6236701	1088.401562	-1.3648	0.0049266	0.030914
i2_LQ_YHS_c53689/f1p5/2290	83.11142862	229.4553013	-1.4651	0.0049261	0.030914
i3_LQ_YHS_c14154/f1p2/3031	0.257237156	92.43767332	-8.4892	0.0049262	0.030914
i4_LQ_YHS_c6152/f1p1/4690	28.65131754	97.80882525	-1.7714	0.0049252	0.030914
i1_LQ_YHS_c6446/f1p1/1496	5.864798203	35.81613547	-2.6105	0.0049336	0.030952
i2_HQ_YHS_c61035/f26p8/2630	322.1767799	842.2325536	-1.3864	0.0049339	0.030952
i1_LQ_YHS_c5735/f1p4/1771	732.4983826	286.148241	1.3561	0.0049369	0.030967
i1_LQ_YHS_c43879/f1p0/1033	1205.745305	427.8827946	1.4946	0.0049378	0.030969
i1_LQ_YHS_c20736/f1p0/1815	144.2691768	49.41300583	1.5458	0.0049399	0.030978
i2_LQ_YHS_c5436/f1p4/2829	1800.910594	15153.15141	-3.0728	0.0049417	0.030979
i3_LQ_YHS_c17727/f1p7/3771	40.85352846	150.2837752	-1.8792	0.0049421	0.030979
i3_LQ_YHS_c6912/f1p7/3451	1006.229871	393.8132315	1.3534	0.0049409	0.030979
i3_LQ_YHS_c6996/f1p0/3494	37.75908323	183.3163654	-2.2794	0.0049452	0.030995
i2_LQ_YHS_c15242/f1p0/2040	0	9.202889855	-Inf	0.0049461	0.030997
i1_LQ_YHS_c23153/f1p5/1801	9.101413359	74.50400984	-3.0332	0.0049492	0.031012
i3_LQ_YHS_c19849/f1p2/3840	0	9.195109584	-Inf	0.0049521	0.031026
i4_LQ_YHS_c11815/f1p3/4524	92.23353492	10.27840853	3.1657	0.004955	0.03104
i0_LQ_YHS_c1116/f1p0/766	2251.995456	675.6129079	1.7369	0.0049597	0.031066
i1_LQ_YHS_c17780/f1p2/1643	75.73522044	309.3535726	-2.0302	0.0049631	0.031083
i2_LQ_YHS_c8036/f1p3/2697	203.4253227	73.98591735	1.4592	0.0049688	0.031115
i2_LQ_YHS_c18325/f1p3/2768	121.6969326	12.90741079	3.237	0.0049727	0.031135
i2_LQ_YHS_c26257/f1p0/3027	122.2527414	40.01596019	1.6112	0.0049751	0.031146
i2_HQ_YHS_c1331/f7p1/2331	709.0920131	287.7565793	1.3011	0.0049776	0.031158
i1_LQ_YHS_c11342/f1p0/1836	256.4715958	625.5416548	-1.2863	0.004983	0.031187
i2_LQ_YHS_c22138/f1p3/3015	54.91357402	332.1196324	-2.5965	0.0049873	0.031211
i1_HQ_YHS_c1633/f6p0/1926	12979.67232	0	Inf	0.0049892	0.031218
i3_LQ_YHS_c5982/f1p1/3229	0	11.76064345	-Inf	0.004991	0.031223
i5_LQ_YHS_c2339/f1p0/5469	63.86808431	253.0179743	-1.9861	0.0049913	0.031223
i1_LQ_YHS_c19045/f1p0/1677	8.618419474	44.25851708	-2.3605	0.0049943	0.03123
i1_LQ_YHS_c4566/f1p5/1449	171.5208521	566.272364	-1.7231	0.0049944	0.03123
i2_HQ_YHS_c48414/f2p0/2149	183.3963607	65.90692704	1.4765	0.0049943	0.03123
i2_LQ_YHS_c11426/f1p4/2548	58.89854493	13.37198628	2.139	0.0049959	0.031236
i2_LQ_YHS_c2922/f1p0/2402	284.4541927	112.9811345	1.3321	0.0049987	0.031249
i5_LQ_YHS_c4478/f1p4/5905	0.544682597	45.08079449	-6.371	0.0049994	0.03125
i1_HQ_YHS_c40670/f2p4/1337	1559.618643	6684.874531	-2.0997	0.0050011	0.031252
i1_LQ_YHS_c19880/f3p0/1753	269.0158166	104.0543932	1.3704	0.0050023	0.031252
i1_LQ_YHS_c26142/f1p0/1845	46.34983871	9.195109584	2.3336	0.0050024	0.031252
i2_HQ_YHS_c11253/f4p0/2892	54.45330876	158.7108681	-1.5433	0.0050016	0.031252
i2_HQ_YHS_c57571/f3p0/2215	242.8753925	587.7334475	-1.2749	0.0050042	0.03126
i0_LQ_YHS_c2214/f1p24/841	404.6983403	1005.552444	-1.3131	0.005005	0.031261
i2_LQ_YHS_c55937/f1p1/2658	238.2686781	91.4847409	1.381	0.0050103	0.031289
i2_LQ_YHS_c26304/f1p1/2373	8.428748255	166.4157279	-4.3033	0.0050224	0.031361
i5_LQ_YHS_c1455/f1p0/5347	24.97367961	85.22199336	-1.7708	0.0050233	0.031362
i1_LQ_YHS_c31762/f1p0/1976	0	9.101625437	-Inf	0.0050245	0.031366

i0_LQ_YHS_c2321/f1p0/969	127.0565408	319.7988037	-1.3317	0.0050294	0.031392
i6_LQ_YHS_c1088/f1p0/6038	0	12.10618218	-Inf	0.0050369	0.031436
i1_LQ_YHS_c41584/f1p1/1906	842.4951707	2562.613688	-1.6049	0.005042	0.031459
i4_LQ_YHS_c7116/f1p0/4360	515.1128326	174.336895	1.563	0.0050416	0.031459
i5_LQ_YHS_c3217/f1p0/5117	329.1645658	45.85315336	2.8437	0.0050428	0.03146
i2_LQ_YHS_c18087/f1p6/2592	111.0341499	310.8977337	-1.4854	0.005055	0.031532
i1_HQ_YHS_c2656/f3p0/1581	926.7992904	358.9271596	1.3686	0.0050581	0.031547
i6_LQ_YHS_c690/f1p0/6198	9.972171192	0	Inf	0.0050669	0.031598
i3_HQ_YHS_c7046/f2p16/3248	212.9746976	80.07453279	1.4113	0.0050693	0.031609
i2_LQ_YHS_c44732/f1p1/2029	34.67512031	108.2524396	-1.6424	0.0050718	0.03162
i3_LQ_YHS_c14260/f1p1/3054	33.24262626	2.238399793	3.8925	0.0050748	0.031635
i3_LQ_YHS_c4325/f1p0/3654	30.49142121	3.892594563	2.9696	0.0050758	0.031637
i2_HQ_YHS_c45330/f5p2/2936	119.4670616	305.5682026	-1.3549	0.0050798	0.031658
i2_HQ_YHS_c9604/f3p2/2361	770.4528527	304.1483308	1.3409	0.0050824	0.031666
i2_LQ_YHS_c44280/f1p164/2029	75.54911071	0	Inf	0.0050831	0.031666
i2_LQ_YHS_c48889/f1p0/2879	177.1694269	19.94480809	3.151	0.0050824	0.031666
i1_LQ_YHS_c11715/f1p9/1886	588.6832228	40.83274934	3.8497	0.0050843	0.031669
i1_LQ_YHS_c18264/f1p0/1740	115.1013241	35.91080517	1.6804	0.0050868	0.031677
i2_LQ_YHS_c63517/f1p6/2611	410.2358342	1065.174352	-1.3766	0.0050864	0.031677
i4_LQ_YHS_c6924/f1p3/4347	69.15827288	192.9975339	-1.4806	0.005088	0.031681
i1_LQ_YHS_c11113/f1p8/1603	24.80119008	2.560004933	3.2762	0.0050911	0.031696
i2_LQ_YHS_c55209/f1p1/3008	10.57392904	50.93366909	-2.2681	0.0051039	0.031771
i2_LQ_YHS_c49115/f1p0/2645	144.6094729	43.16226218	1.7443	0.005106	0.03178
i2_LQ_YHS_c35240/f1p2/2823	219.4435418	534.6965637	-1.2849	0.005107	0.031783
i2_LQ_YHS_c64442/f1p1/2015	696.3100045	1948.355153	-1.4845	0.0051098	0.031796
i1_LQ_YHS_c22404/f1p4/1894	155.2858926	54.76101693	1.5037	0.0051111	0.0318
i2_LQ_YHS_c37542/f1p0/2395	998.1543321	268.0161982	1.8969	0.0051119	0.031801
i5_LQ_YHS_c2161/f1p0/5118	167.6206133	55.55612384	1.5932	0.0051133	0.031805
i1_LQ_YHS_c7795/f1p0/2010	267.7782762	103.5358277	1.3709	0.0051168	0.031819
i4_LQ_YHS_c13669/f1p6/4130	208.7889209	78.19340258	1.4169	0.0051161	0.031819
i1_LQ_YHS_c28015/f1p0/1260	364.0668777	4.79674616	6.246	0.0051212	0.031834
i2_HQ_YHS_c5718/f2p3/2249	281.4680567	727.2132095	-1.3694	0.0051208	0.031834
i2_LQ_YHS_c50213/f1p3/2309	29.70922743	0	Inf	0.0051202	0.031834
i1_HQ_YHS_c16625/f9p0/1621	7097.771516	1548.125608	2.1968	0.0051243	0.031845
i2_LQ_YHS_c12163/f1p0/3040	30.92321707	101.7102659	-1.7177	0.0051241	0.031845
i1_LQ_YHS_c28291/f1p0/1294	21.7017676	80.06236959	-1.8833	0.00513	0.031876
i4_LQ_YHS_c5499/f1p0/4618	72.33827952	13.68640392	2.402	0.0051326	0.031889
i2_LQ_YHS_c5870/f1p1/2340	256.860438	98.63505324	1.3808	0.0051346	0.031897
i2_LQ_YHS_c53865/f1p1/2785	57.54684564	163.069777	-1.5027	0.0051383	0.031916
i2_HQ_YHS_c2502/f2p0/2843	70.44132795	190.6140321	-1.4362	0.0051416	0.031932
i1_LQ_YHS_c31873/f1p0/1766	133.9680964	842.2439772	-2.6523	0.0051495	0.03196
i2_LQ_YHS_c11906/f1p0/3005	297.6528521	116.443199	1.354	0.0051474	0.03196
i2_LQ_YHS_c18605/f1p6/2130	70.7990033	196.7450276	-1.4745	0.0051492	0.03196
i2_LQ_YHS_c50914/f1p6/2654	0	16.18459903	-Inf	0.0051481	0.03196
i3_LQ_YHS_c19688/f1p1/3726	0	11.77454542	-Inf	0.005149	0.03196
i1_LQ_YHS_c19396/f1p2/1365	406.2989857	1692.898639	-2.0589	0.005154	0.031984
i1_LQ_YHS_c17569/f1p0/1487	1742.653543	18761.73724	-3.4284	0.0051578	0.032004
i2_LQ_YHS_c41098/f1p1/2371	168.751788	59.64244072	1.5005	0.0051605	0.032016
i1_LQ_YHS_c14037/f1p4/1079	1798.689828	617.0788518	1.5434	0.0051626	0.032025
i1_LQ_YHS_c3933/f1p0/1982	518.2516844	1395.117324	-1.4287	0.0051632	0.032025
i1_LQ_YHS_c39866/f4p7/1453	289.5004232	60.36230883	2.2618	0.00517	0.032063
i1_LQ_YHS_c28061/f1p0/1306	116.1450956	569.8420729	-2.2946	0.005172	0.032071
i2_LQ_YHS_c29526/f1p1/2037	339.0096067	7.919866645	5.4197	0.0051736	0.032077
i2_LQ_YHS_c18057/f1p4/2565	59.51043805	170.092167	-1.5151	0.0051834	0.03213
i3_LQ_YHS_c8878/f1p45/3840	213.1079457	80.14752024	1.4109	0.0051829	0.03213
i1_LQ_YHS_c20920/f1p0/1936	101.6540848	671.2185016	-2.7231	0.005186	0.032142
i2_LQ_YHS_c7256/f1p1/2414	504.4209199	201.752635	1.322	0.005199	0.032218
i2_HQ_YHS_c1705/f7p0/2393	131.6378591	30.6516151	2.1025	0.0052004	0.032222
i5_LQ_YHS_c2960/f1p0/5082	1.089365194	16.93247107	-3.9582	0.0052038	0.032239
i4_LQ_YHS_c5116/f1p0/4772	1353.486204	347.7904955	1.9604	0.005208	0.032257
i6_LQ_YHS_c991/f2p0/6142	864.5160246	302.8217419	1.5134	0.0052079	0.032257
i1_LQ_YHS_c25795/f1p1/1661	95.90309001	247.0542037	-1.3652	0.0052123	0.03228
i4_LQ_YHS_c13206/f1p0/4052	7.906319168	43.32482274	-2.4541	0.0052166	0.032302
i2_LQ_YHS_c59052/f1p1/2029	147.4497171	12.15349725	3.6008	0.0052276	0.032363
i3_LQ_YHS_c4276/f1p0/3300	81.82020452	22.77625896	1.8449	0.0052278	0.032363
i4_LQ_YHS_c14140/f1p0/4173	125.0526624	0	Inf	0.0052326	0.032389
i4_LQ_YHS_c11056/f1p12/4282	237.3814243	93.16318296	1.3494	0.0052342	0.032395
i2_HQ_YHS_c24591/f2p5/2139	3.56730385	28.65745008	-3.006	0.0052409	0.032432
i3_LQ_YHS_c17665/f1p2/3862	186.3976942	465.5061674	-1.3204	0.0052418	0.032434
i2_LQ_YHS_c11668/f1p0/2177	30.27996321	2.830977864	3.419	0.0052446	0.032446
i6_LQ_YHS_c867/f1p0/6211	0	16273.88598	-Inf	0.0052471	0.032458
i2_HQ_YHS_c60349/f5p0/2475	237.2072115	594.0056745	-1.3243	0.0052486	0.032463
i2_LQ_YHS_c12210/f1p5/2912	194.291487	466.6945654	-1.2643	0.0052506	0.032471
i2_HQ_YHS_c23258/f2p5/2351	419.7762407	48.90715675	3.1015	0.0052555	0.032497
i4_LQ_YHS_c10667/f1p2/4295	30.55483205	102.8408799	-1.7509	0.0052572	0.032504
i4_LQ_YHS_c5118/f1p0/4759	181.3122164	8.208058685	4.4653	0.0052579	0.032504
i1_LQ_YHS_c24884/f1p3/1487	200.1940689	12.10227227	4.048	0.0052662	0.032549
i2_HQ_YHS_c15683/f6p4/2381	530.4627069	70.30805437	2.9155	0.0052665	0.032549
i1_LQ_YHS_c3131/f1p0/1705	175.840303	62.61362503	1.4897	0.0052698	0.032565
i1_HQ_YHS_c3910/f3p1/1731	573.5596523	232.1923231	1.3046	0.0052724	0.032573
i1_LQ_YHS_c14025/f1p0/1301	456.4746519	126.5536378	1.8508	0.0052717	0.032573
i2_HQ_YHS_c32530/f2p1/2682	73.96459152	198.6611481	-1.4254	0.0052759	0.03259
i1_LQ_YHS_c26267/f1p0/1789	203.6536978	45.14766136	2.1734	0.00528	0.032611
i2_LQ_YHS_c15079/f1p1/2039	1663.808489	589.7056451	1.4964	0.0052815	0.032617
i2_LQ_YHS_c18381/f1p3/2733	75.42881375	20.22024416	1.8993	0.0052831	0.032622
i4_HQ_YHS_c2066/f3p0/4941	24.95475067	90.79536155	-1.8633	0.0052845	0.032627
i2_HQ_YHS_c2072/f14p2/2656	858.9567822	342.1416885	1.328	0.0052863	0.032629
i3_LQ_YHS_c5305/f1p2/3691	41.89125402	130.0922403	-1.6348	0.0052863	0.032629
i5_LQ_YHS_c2368/f1p3/5206	29.84260368	110.214771	-1.8849	0.0052879	0.032635

i5_LQ_YHS_c1121/f1p0/5847	12.86300189	57.09961535	-2.1503	0.0052896	0.032641
i2_LQ_YHS_c18299/f1p3/2931	5.598334131	36.2607675	-2.6953	0.0052928	0.032657
i1_LQ_YHS_c17647/f1p0/1850	220.2371597	82.07442672	1.4241	0.0053038	0.032721
i4_LQ_YHS_c6477/f1p0/4395	44.25343135	2.869366663	3.947	0.0053057	0.032728
i1_HQ_YHS_c7968/f4p0/1579	1625.818739	399.2713196	2.0257	0.0053073	0.03273
i3_HQ_YHS_c3763/f2p0/3681	393.4001173	160.6354442	1.2922	0.005307	0.03273
i3_LQ_YHS_c6519/f1p2/3214	48.34811642	144.7267531	-1.5818	0.005308	0.03273
i2_LQ_YHS_c11085/f1p1/3016	426.5131958	1102.558195	-1.3702	0.0053168	0.032776
i5_LQ_YHS_c4701/f1p0/5122	362.8024969	73.12122035	2.3108	0.0053163	0.032776
i4_LQ_YHS_c13989/f1p11/4090	25.54175979	316.717182	-3.6323	0.0053179	0.032778
i2_HQ_YHS_c9517/f2p0/2390	56.97657659	159.1893061	-1.4823	0.0053343	0.032873
i3_LQ_YHS_c17721/f1p0/3564	1880.097487	661.0562061	1.508	0.0053346	0.032873
i3_HQ_YHS_c2384/f2p0/3576	118.4475793	307.9597637	-1.3785	0.0053365	0.032881
i2_LQ_YHS_c18500/f1p5/2227	24.39833036	116.9509055	-2.261	0.0053388	0.03289
i2_LQ_YHS_c52358/f1p1/2296	62.11798677	14.86871595	2.0627	0.0053429	0.032912
i3_LQ_YHS_c5614/f1p0/3205	35.94159687	107.7506202	-1.584	0.0053439	0.032914
i1_LQ_YHS_c22417/f1p1/1862	54.09497281	11.99934928	2.1725	0.0053474	0.032931
i2_LQ_YHS_c7081/f1p4/2500	107.7177727	268.2243757	-1.3162	0.0053522	0.032956
i2_LQ_YHS_c40812/f1p1/2508	50.95104331	145.5636857	-1.5145	0.0053532	0.032958
i3_LQ_YHS_c14389/f1p4/3007	77.75525262	21.17222339	1.8768	0.0053582	0.032984
i1_LQ_YHS_c20951/f1p0/1756	182.7959779	450.0910485	-1.3	0.0053636	0.033014
i1_LQ_YHS_c36237/f1p0/1833	24.2086424	86.69352366	-1.8404	0.0053685	0.03304
i2_HQ_YHS_c45498/f17p5/2153	186.7724232	458.3868207	-1.2953	0.0053784	0.033088
i2_LQ_YHS_c39811/f1p1/2092	52.27505348	11.24934566	2.2163	0.0053785	0.033088
i3_LQ_YHS_c8599/f1p0/3500	745.5637293	2134.498526	-1.5175	0.0053777	0.033088
i3_LQ_YHS_c11708/f1p5/3107	35.75826961	156.5030758	-2.1298	0.0053838	0.033117
i1_LQ_YHS_c25427/f1p6/1539	361.7750273	142.6359066	1.3428	0.0053861	0.033123
i1_LQ_YHS_c7685/f1p1/1447	147.3396799	50.24089239	1.5522	0.0053858	0.033123
i2_HQ_YHS_c17743/f5p0/2797	233.2588179	586.0868736	-1.3292	0.0053925	0.033158
i1_LQ_YHS_c32144/f1p0/1972	228.4585393	40.43764419	2.4982	0.0053968	0.033178
i3_LQ_YHS_c14370/f1p0/3015	38.79552827	6.400308722	2.5997	0.0053972	0.033178
i3_LQ_YHS_c3799/f1p0/3592	1457.919301	535.5740601	1.4448	0.0054009	0.033197
i1_LQ_YHS_c22520/f1p0/1637	345.6085672	63.89546269	2.4354	0.0054021	0.0332
i2_LQ_YHS_c9967/f2p5/2372	10.26630764	46.92025944	-2.1923	0.0054047	0.033212
i1_LQ_YHS_c20818/f1p0/1559	57.05578566	211.894269	-1.8929	0.0054084	0.03323
i1_LQ_YHS_c41164/f1p3/1909	69.59287387	0.359993938	7.5948	0.0054144	0.03326
i1_LQ_YHS_c5162/f1p0/1764	331.1127525	98.75128543	1.7455	0.0054147	0.03326
i1_LQ_YHS_c23169/f1p1/1452	475.6312093	1253.724316	-1.3983	0.0054161	0.033261
i2_HQ_YHS_c4735/f3p1/2403	817.2592852	146.5315788	2.4796	0.0054159	0.033261
i4_LQ_YHS_c3948/f1p0/4615	444.2009749	178.4364815	1.3158	0.0054184	0.03327
i2_HQ_YHS_c61548/f2p0/2048	782.8507724	28.04488787	4.8029	0.0054259	0.033308
i3_LQ_YHS_c4525/f1p2/3509	366.2112341	147.19071	1.315	0.0054254	0.033308
i3_LQ_YHS_c2844/f1p0/3798	95.48063239	29.35907284	1.7014	0.0054273	0.033313
i3_LQ_YHS_c10747/f1p0/3114	22.21703894	150.6116602	-2.7611	0.0054302	0.033326
i2_LQ_YHS_c4186/f1p5/2579	46.88135827	582.6650569	-3.6356	0.0054367	0.033357
i2_LQ_YHS_c8873/f1p7/2682	1522.819437	573.2104725	1.4096	0.005436	0.033357
i1_LQ_YHS_c26262/f1p0/1689	23.95602707	81.99417156	-1.7751	0.0054395	0.03337
i2_LQ_YHS_c59260/f1p2/2045	6.4094808	83.83904567	-3.7093	0.0054463	0.033408
i1_LQ_YHS_c18250/f1p1/1767	96.32737271	556.9182493	-2.5314	0.0054487	0.033418
i1_LQ_YHS_c14328/f1p0/1349	242.771175	596.5654399	-1.2971	0.0054574	0.033463
i3_LQ_YHS_c3948/f1p0/3103	300.6736268	120.5122572	1.319	0.0054567	0.033463
i5_LQ_YHS_c3058/f1p0/5927	1.351215809	17.05482496	-3.6578	0.0054586	0.033466
i1_LQ_YHS_c31550/f2p0/1606	72.02419446	190.1221257	-1.4004	0.0054604	0.033473
i4_LQ_YHS_c6253/f1p3/4891	199.2329375	71.82587351	1.4719	0.0054663	0.033505
i4_LQ_YHS_c11477/f1p21/4530	141.1622579	50.24756733	1.4902	0.0054779	0.033572
i3_LQ_YHS_c11070/f1p3/3757	188.9088187	470.9724346	-1.318	0.0054885	0.033629
i3_LQ_YHS_c11645/f1p0/3210	3.596248367	24.65806217	-2.7775	0.0054887	0.033629
i2_LQ_YHS_c51837/f1p0/2076	0	11.58426	-Inf	0.0054912	0.033641
i2_LQ_YHS_c25528/f1p0/2697	91.22094583	26.74571134	1.7701	0.0055034	0.033711
i1_LQ_YHS_c39749/f1p3/1298	142.4355786	353.6692748	-1.3121	0.0055053	0.033718
i2_LQ_YHS_c37834/f1p4/2877	201.7344964	496.7694778	-1.3001	0.0055142	0.033763
i2_LQ_YHS_c51436/f1p5/2620	30.01382944	99.68379421	-1.7317	0.0055147	0.033763
i3_LQ_YHS_c11372/f1p1/3407	0.904307434	15.20315727	-4.0714	0.0055146	0.033763
i2_LQ_YHS_c25505/f1p0/2396	194.2413673	71.16884147	1.4485	0.0055164	0.033769
i2_LQ_YHS_c37095/f1p2/2394	162.9122292	404.4460716	-1.3119	0.005519	0.033781
i2_LQ_YHS_c51100/f1p0/2790	55.66907914	4.392241718	3.6638	0.0055229	0.0338
i2_LQ_YHS_c51523/f1p207/2028	9208.324476	1.472242847	12.611	0.0055259	0.033814
i2_LQ_YHS_c33076/f1p2/2552	22.30051443	80.77907988	-1.8569	0.0055291	0.03383
i1_HQ_YHS_c1647/f10p2/1949	513.9166912	1372.126998	-1.4168	0.0055322	0.03384
i2_LQ_YHS_c50644/f1p0/2129	36.02933877	112.2134387	-1.639	0.0055314	0.03384
i1_LQ_YHS_c7751/f1p1/1704	251.3102633	7.170336031	5.1313	0.0055398	0.033883
i2_LQ_YHS_c19984/f1p4/2933	74.96853174	205.4004427	-1.4541	0.0055444	0.033906
i2_HQ_YHS_c10211/f2p0/2612	125.6343603	11.01676152	3.5115	0.0055492	0.033931
i2_LQ_YHS_c53371/f1p7/2698	87.75507944	231.7440207	-1.401	0.0055561	0.033969
i2_LQ_YHS_c29437/f1p4/2132	32.62022571	154.4777094	-2.2436	0.005557	0.033971
i2_LQ_YHS_c10956/f1p6/2277	5.628542416	36.45989898	-2.6955	0.005558	0.033972
i1_LQ_YHS_c21299/f1p11/1735	624.8250499	252.8780481	1.305	0.0055739	0.034063
i2_LQ_YHS_c38537/f1p0/2077	165.5528729	54.14960073	1.6123	0.0055742	0.034063
i3_LQ_YHS_c19890/f1p0/3859	637.7532709	265.3188655	1.2653	0.0055788	0.034086
i2_LQ_YHS_c37255/f1p4/2642	16.90535322	110.6251625	-2.7101	0.0055828	0.034107
i2_LQ_YHS_c10485/f1p10/2327	144.7915891	368.6825417	-1.3484	0.0055886	0.034138
i2_LQ_YHS_c10241/f1p7/2337	5.963844568	161.602517	-4.7601	0.0055902	0.034139
i2_LQ_YHS_c24806/f1p0/2344	10.00826508	0	Inf	0.0055904	0.034139
i2_LQ_YHS_c55266/f1p1/2722	397.4727272	982.7827464	-1.306	0.0055909	0.034139
i3_LQ_YHS_c11071/f1p0/3310	595.7864962	248.7859232	1.2599	0.0055965	0.034168
i3_LQ_YHS_c5033/f1p1/3517	138.9452699	338.7990992	-1.2859	0.0055987	0.034178
i3_LQ_YHS_c17511/f1p3/3224	21.20286416	77.9297369	-1.8779	0.0055996	0.034179
i1_LQ_YHS_c17807/f1p0/1852	0	25.20103427	-Inf	0.0056037	0.034199
i0_LQ_YHS_c1020/f1p0/782	697.1901065	276.6319587	1.3336	0.005606	0.034208

i2_HQ_YHS_c17463/f2p2/2252	17.22174318	65.32651216	-1.9234	0.0056065	0.034208
i1_LQ_YHS_c6495/f1p0/1708	123.6344634	4.79674616	4.6879	0.0056074	0.034209
i5_LQ_YHS_c2809/f1p2/5209	54.50149577	157.3515008	-1.5296	0.0056166	0.034261
i3_LQ_YHS_c17733/f1p4/3096	46.00022944	9.45996081	2.2817	0.0056218	0.034288
i1_LQ_YHS_c5419/f1p0/1941	89.31187602	233.5582445	-1.3869	0.0056345	0.034361
i3_LQ_YHS_c10477/f1p12/3326	92.23753683	238.9818081	-1.3735	0.0056414	0.0344
i1_LQ_YHS_c9050/f1p36/1444	0	9.073228707	-Inf	0.0056473	0.03443
i2_HQ_YHS_c4270/f3p0/2216	92.55358812	236.2463322	-1.3519	0.0056479	0.03443
i2_LQ_YHS_c10982/f1p24/2635	89.33514554	3.184850097	4.8099	0.0056513	0.034447
i2_LQ_YHS_c54568/f1p1/2523	71.40095501	196.8911942	-1.4634	0.0056529	0.034452
i2_HQ_YHS_c38367/f2p1/2990	0	11.76230201	-Inf	0.0056553	0.034463
i1_HQ_YHS_c3087/f2p0/1658	739.5227878	2141.050688	-1.5337	0.0056647	0.034515
i1_LQ_YHS_c13048/f1p1/1746	506.0622247	201.5195778	1.3284	0.0056659	0.034518
i2_HQ_YHS_c12695/f4p2/2565	252.5333136	70.67586812	1.8372	0.0056723	0.034553
i1_HQ_YHS_c40265/f6p8/1386	3761.46717	482.5617389	2.9625	0.0056736	0.034557
i3_LQ_YHS_c16929/f1p0/3355	0	9.034839909	-Inf	0.0056801	0.034583
i3_LQ_YHS_c3130/f1p0/3490	5.71916727	82.69452967	-3.8539	0.0056801	0.034583
i4_LQ_YHS_c4850/f1p4/4304	99.67991912	17.92449767	2.4754	0.0056789	0.034583
i5_LQ_YHS_c3836/f1p6/5881	64.04454345	15.98767934	2.0021	0.0056883	0.034629
i2_LQ_YHS_c25088/f1p0/2781	381.4354272	107.2426011	1.8306	0.0056925	0.03465
i1_HQ_YHS_c2505/f2p2/1719	5.446825971	60.84217183	-3.4816	0.0056969	0.034663
i2_LQ_YHS_c8828/f1p0/2150	0	9.016474793	-Inf	0.0056958	0.034663
i3_LQ_YHS_c8408/f1p0/3682	3.417067833	25.72414201	-2.9123	0.0056964	0.034663
i2_LQ_YHS_c25001/f1p5/2984	17.78325205	1.07386011	4.0496	0.0056987	0.03467
i2_LQ_YHS_c39879/f1p2/2616	17.74924409	68.53363729	-1.9491	0.0056997	0.034672
i2_LQ_YHS_c23689/f1p8/2855	0	9.008694521	-Inf	0.0057025	0.034684
i2_LQ_YHS_c34010/f1p2/2239	132.2338833	333.4839419	-1.3345	0.0057048	0.034694
i0_LQ_YHS_c3114/f1p0/397	44.29783543	3.061310654	3.855	0.0057074	0.034696
i1_LQ_YHS_c33365/f1p0/1683	0	10.63002942	-Inf	0.0057067	0.034696
i2_HQ_YHS_c17403/f2p0/2690	89.51746516	230.0400991	-1.3616	0.0057095	0.034696
i2_LQ_YHS_c38249/f1p0/2330	153.3569197	53.60212594	1.5165	0.0057061	0.034696
i2_LQ_YHS_c9853/f1p1/2222	155.6399956	53.54703059	1.5393	0.0057079	0.034696
i4_LQ_YHS_c9328/f1p0/4725	149.4084843	378.6306572	-1.3415	0.0057088	0.034696
i3_LQ_YHS_c14156/f1p1/3056	117.3617828	292.6554617	-1.3182	0.0057171	0.034738
i1_LQ_YHS_c17907/f1p0/1728	153.4404694	368.0695054	-1.2623	0.0057179	0.034739
i0_LQ_YHS_c2709/f1p3/987	1917.929778	689.9174079	1.4751	0.0057198	0.034746
i2_LQ_YHS_c58434/f1p1/2097	617.2915498	258.1000756	1.258	0.0057252	0.034774
i1_LQ_YHS_c27667/f1p0/1172	388.0276572	107.3348986	1.854	0.0057293	0.034795
i2_LQ_YHS_c55876/f1p0/2429	35.33441178	5.993552937	2.5596	0.0057328	0.034808
i3_LQ_YHS_c13438/f1p0/3216	2.662996416	24.82551934	-3.2207	0.0057324	0.034808
i2_LQ_YHS_c8272/f1p2/2443	241.868804	71.21240594	1.764	0.0057369	0.034823
i6_LQ_YHS_c529/f1p0/6249	450.1993852	64.91221563	2.794	0.0057366	0.034823
i2_LQ_YHS_c20431/f1p0/2290	78.5453928	284.8347268	-1.8585	0.0057403	0.03484
i1_LQ_YHS_c9689/f1p0/1684	75.2142418	455.8992785	-2.5996	0.0057457	0.034868
i2_LQ_YHS_c8408/f1p0/2389	0	9.1054958	-Inf	0.0057515	0.03489
i3_LQ_YHS_c13655/f1p0/3135	7.327620235	62.22424767	-3.0861	0.0057514	0.03489
i4_LQ_YHS_c5165/f1p0/4515	0.359624837	30293.23616	-16.362	0.0057512	0.03489
i3_LQ_YHS_c15014/f1p0/3061	543.9905627	225.3394416	1.2715	0.0057527	0.034893
i1_LQ_YHS_c22802/f1p0/1588	95.780518	244.4953447	-1.352	0.0057571	0.034915
i1_LQ_YHS_c18038/f1p32/1618	12.84202052	58.40388847	-2.1852	0.0057649	0.034958
i2_HQ_YHS_c5541/f2p2/2676	270.5124443	107.466259	1.3318	0.0057728	0.034997
i5_HQ_YHS_c742/f2p0/5394	55.92459417	147.8738479	-1.4028	0.0057727	0.034997
i3_LQ_YHS_c13702/f1p1/3304	1365.962293	5048.110639	-1.8858	0.0057766	0.035016
i2_LQ_YHS_c33889/f1p1/2547	2411.57751	816.6787848	1.5621	0.005778	0.03502
i2_LQ_YHS_c25954/f1p1/2195	0	8.919673514	-Inf	0.0057794	0.035024
i3_LQ_YHS_c7048/f1p0/4025	2.050747881	20.94741953	-3.3526	0.0057915	0.035093
i3_HQ_YHS_c1268/f6p3/3561	785.0999457	144.6743821	2.4401	0.0057935	0.035101
i0_LQ_YHS_c2682/f1p0/795	63.40783579	176.4587835	-1.4766	0.0057958	0.035107
i2_LQ_YHS_c53521/f1p4/2760	91.64927231	237.4212161	-1.3733	0.005796	0.035107
i4_LQ_YHS_c9431/f1p0/5034	7.059086988	40.24735877	-2.5113	0.0058001	0.035128
i2_HQ_YHS_c26691/f2p4/2677	162.2808914	395.3993008	-1.2848	0.0058044	0.035149
i2_LQ_YHS_c25084/f1p3/2218	1.316394066	18.14152126	-3.7846	0.0058063	0.035154
i2_LQ_YHS_c28130/f1p1/2014	850.9752283	339.2845257	1.3266	0.0058066	0.035154
i2_LQ_YHS_c38405/f1p6/3070	25.67240627	2.123233398	3.5959	0.0058109	0.035175
i1_LQ_YHS_c8446/f1p0/1571	1246.39331	149.1810024	3.0626	0.0058134	0.035186
i1_HQ_YHS_c18636/f2p0/1750	955.9156567	388.6602481	1.2984	0.0058157	0.035196
i2_LQ_YHS_c35063/f1p1/2935	75.69688864	20.01325218	1.9193	0.0058209	0.035223
i2_LQ_YHS_c14140/f1p0/2046	322.9658514	808.4971446	-1.3239	0.0058218	0.035224
i1_LQ_YHS_c17550/f1p0/1807	433.3048828	80.14364988	2.4347	0.0058253	0.03524
i3_LQ_YHS_c11871/f1p0/3279	830.6430179	335.8454883	1.3064	0.0058263	0.035242
i1_HQ_YHS_c2250/f3p0/1529	306.9839129	11.8713467	4.6926	0.0058348	0.035289
i2_LQ_YHS_c40118/f1p4/2687	20.89985622	73.62146028	-1.8166	0.0058359	0.035292
i5_LQ_YHS_c4510/f1p0/5398	200.9425479	463.5089226	-1.2058	0.0058378	0.035299
i2_LQ_YHS_c37356/f1p4/2366	175.6981164	62.80185797	1.4842	0.005842	0.03532
i2_LQ_YHS_c6445/f1p4/2386	246.9597844	614.4748105	-1.3151	0.0058443	0.035329
i3_LQ_YHS_c12880/f1p0/3647	8.485823509	44.086477	-2.3772	0.005856	0.035392
i3_LQ_YHS_c8594/f1p0/3960	0.359624837	11.45187449	-4.9929	0.0058562	0.035392
i2_LQ_YHS_c6048/f1p3/2385	11.24244742	55.16220424	-2.2947	0.00586	0.035406
i5_LQ_YHS_c899/f1p0/5322	7.454805709	870.3331758	-6.8673	0.0058592	0.035406
i1_LQ_YHS_c36807/f1p0/1780	5.201351641	34.29713078	-2.7211	0.0058671	0.035444
i2_HQ_YHS_c60197/f14p7/2343	315.2513851	124.8522477	1.3363	0.0058717	0.035468
i1_HQ_YHS_c12027/f2p0/1961	132.6719741	45.918794	1.5307	0.0058769	0.03548
i1_HQ_YHS_c17138/f2p0/1993	497.78857	159.2898184	1.6439	0.005878	0.03548
i1_LQ_YHS_c25002/f1p0/1628	793.4752617	58.41387941	3.7638	0.0058748	0.03548
i2_LQ_YHS_c18495/f1p2/2430	62.94882592	261.9725591	-2.0572	0.0058757	0.03548
i2_LQ_YHS_c50014/f1p0/2745	424.920291	1430.083357	-1.7508	0.0058769	0.03548
i3_LQ_YHS_c3623/f1p0/3489	375.5940469	68.17810649	2.4618	0.005878	0.03548
i2_LQ_YHS_c25660/f1p5/3003	134.5599141	320.0366784	-1.25	0.0058822	0.035501
i3_LQ_YHS_c2049/f2p3/3462	1.286185781	18.01577002	-3.8081	0.0058841	0.035505

i3_LQ_YHS_c9077/f1p2/3514	188.8768811	33.31959894	2.503	0.0058845	0.035505
i2_LQ_YHS_c12338/f1p0/2315	91.90652621	27.4990321	1.7408	0.0058863	0.035512
i1_LQ_YHS_c3132/f1p0/1862	246.1269263	94.65738825	1.3786	0.0058887	0.035522
i1_LQ_YHS_c21623/f1p0/1571	12.96459253	0.359993938	5.1705	0.0059037	0.035608
i3_LQ_YHS_c5258/f1p2/3399	216.0512466	82.23631542	1.3935	0.0059087	0.035633
i3_LQ_YHS_c8925/f1p5/3494	1275.307616	97.31917016	3.712	0.0059107	0.035641
i2_LQ_YHS_c54752/f1p0/2298	448.9329791	182.0230722	1.3024	0.0059129	0.03565
i2_LQ_YHS_c20095/f1p4/2740	28.31314081	4.19970495	2.7531	0.0059259	0.035724
i3_HQ_YHS_c16502/f11p0/3264	916.2043143	2859.927769	-1.6422	0.0059277	0.03573
i2_LQ_YHS_c49899/f1p2/2571	96.87462471	29.75638979	1.7029	0.0059292	0.035735
i3_HQ_YHS_c21065/f9p2/3217	157.0269666	1365.154712	-3.12	0.0059341	0.03576
i5_LQ_YHS_c1064/f1p0/5697	41.38922825	119.3871314	-1.5283	0.0059393	0.035787
i4_LQ_YHS_c7438/f1p0/4584	102.6189368	31.17081293	1.719	0.0059488	0.03584
i1_LQ_YHS_c31872/f1p0/1585	41.3129105	6.179967999	2.7409	0.0059514	0.035843
i3_HQ_YHS_c2099/f2p0/3667	74.55645348	12.77613062	2.5449	0.0059515	0.035843
i4_HQ_YHS_c1648/f7p0/4490	818.7395318	334.7195709	1.2905	0.0059506	0.035843
i1_LQ_YHS_c38477/f1p0/1105	117.842352	38.79912973	1.6028	0.0059646	0.035912
i3_HQ_YHS_c2187/f2p0/3171	88.037325	228.7241369	-1.3774	0.0059652	0.035912
i3_LQ_YHS_c12989/f1p0/3513	111.0097494	31.18526814	1.8317	0.0059651	0.035912
i2_LQ_YHS_c50341/f1p8/2411	33.22945485	209.0264807	-2.6532	0.0059739	0.035959
i2_LQ_YHS_c40383/f1p4/3006	81.73843441	24.46832997	1.7401	0.0059758	0.035967
i3_LQ_YHS_c3961/f1p4/3302	147.6966324	51.19899333	1.5284	0.0059942	0.036073
i3_HQ_YHS_c15608/f3p0/3115	276.314533	681.458473	-1.3023	0.005997	0.036085
i2_LQ_YHS_c17532/f2p1/2397	81.358192	22.83806879	1.8328	0.0060101	0.036158
i2_LQ_YHS_c6572/f1p1/2924	376.8702746	152.2911285	1.3072	0.0060106	0.036158
i1_LQ_YHS_c21948/f1p3/1834	163.730542	400.047901	-1.2888	0.0060176	0.036191
i2_LQ_YHS_c19606/f1p3/2985	1.558527079	23.00339438	-3.8836	0.0060168	0.036191
i3_HQ_YHS_c2165/f2p1/3422	49.48312434	248.0895206	-2.3259	0.0060243	0.036227
i2_LQ_YHS_c41395/f1p0/2121	68.17438055	182.1688866	-1.418	0.0060263	0.036234
i2_LQ_YHS_c63847/f1p5/2516	347.922396	135.295862	1.3626	0.0060307	0.036252
i5_LQ_YHS_c3453/f1p2/5719	3.159830676	24.66805424	-2.9647	0.0060307	0.036252
i1_HQ_YHS_c29150/f17p0/2000	1014.971022	3167.345122	-1.6418	0.006033	0.036261
i3_LQ_YHS_c9830/f1p1/3567	78.0646129	11.98935722	2.7029	0.0060377	0.036284
i2_HQ_YHS_c1270/f10p4/2295	508.6181997	57.24207201	3.1514	0.0060391	0.036289
i1_LQ_YHS_c26393/f1p3/1917	81.26569183	209.0497078	-1.3631	0.0060532	0.036369
i1_LQ_YHS_c4013/f1p8/1695	167.3056319	19.64709699	3.0901	0.0060616	0.036415
i3_LQ_YHS_c10115/f1p1/3534	303.6415974	121.9194126	1.3164	0.0060706	0.036464
i3_LQ_YHS_c7409/f1p0/3285	517.3693085	216.098836	1.2595	0.0060718	0.036467
i2_LQ_YHS_c49081/f1p1/2111	23.87635964	102.959603	-2.1084	0.0060791	0.036506
i1_LQ_YHS_c18689/f1p1/1431	13.50177872	319.8163068	-4.566	0.0060837	0.036529
i1_LQ_YHS_c25946/f1p3/1654	739.1246265	1931.450004	-1.3858	0.0060855	0.036531
i2_LQ_YHS_c49459/f1p11/2440	102.1567627	297.1286316	-1.5403	0.0060848	0.036531
i2_LQ_YHS_c8077/f1p1/2372	25.18847892	3.138088251	3.0048	0.0060899	0.036549
i3_LQ_YHS_c4064/f1p0/3147	160.2277524	10.72130177	3.9016	0.00609	0.036549
i2_HQ_YHS_c2437/f5p1/2276	84.49051066	284.3184495	-1.7506	0.0060934	0.036565
i2_LQ_YHS_c33332/f1p8/2616	2709.937743	902.0582482	1.587	0.0060944	0.036566
i2_HQ_YHS_c3077/f7p0/2571	391.7195603	158.6101174	1.3043	0.0060974	0.03658
i4_HQ_YHS_c14555/f3p5/4237	0	9.634132462	-Inf	0.0060986	0.036583
i2_LQ_YHS_c54502/f1p2/2865	688.2488072	282.8505166	1.2829	0.0061007	0.03659
i2_LQ_YHS_c55530/f1p7/2588	129.4362469	43.04721555	1.5882	0.0061064	0.03662
i1_LQ_YHS_c21261/f1p0/1622	56.7604022	2.31517739	4.6157	0.0061085	0.036628
i2_LQ_YHS_c40902/f1p6/2476	467.5262122	191.7583888	1.2858	0.0061109	0.036638
i1_LQ_YHS_c21761/f1p0/1941	39.29108202	118.7729513	-1.5959	0.0061166	0.036663
i1_LQ_YHS_c42567/f1p0/1619	25.4050171	88.93626683	-1.8077	0.0061172	0.036663
i5_LQ_YHS_c2653/f1p0/5720	651.71506	267.8379968	1.2829	0.0061159	0.036663
i2_LQ_YHS_c2577/f3p3/2269	199.76914	555.5601067	-1.4756	0.0061269	0.036716
i1_LQ_YHS_c19036/f1p0/1870	261.3295959	622.5264699	-1.2523	0.0061308	0.036735
i2_LQ_YHS_c50961/f1p2/2340	360.022273	1123.433511	-1.6418	0.006142	0.036796
i4_LQ_YHS_c3768/f1p16/5057	336.0350216	135.5484303	1.3098	0.0061425	0.036796
i3_LQ_YHS_c6906/f1p0/3162	16.77990662	1.079981815	3.9577	0.0061454	0.036808
i2_LQ_YHS_c36169/f1p2/2176	48.71188798	140.3168588	-1.5263	0.0061467	0.036812
i3_LQ_YHS_c4160/f1p7/3688	8.804749376	43.77711527	-2.3138	0.0061606	0.03689
i1_LQ_YHS_c7803/f1p0/1471	3.529946197	26.7879705	-2.9239	0.0061629	0.036899
i2_LQ_YHS_c33944/f1p189/2284	934.2629449	36705.53739	-5.296	0.0061642	0.036903
i4_LQ_YHS_c3248/f1p9/5106	709.1608377	4477.562171	-2.6585	0.0061663	0.03691
i1_LQ_YHS_c8979/f1p0/1894	65.56605992	174.9221658	-1.4157	0.0061678	0.036915
i1_LQ_YHS_c14426/f1p0/1323	108.5870968	638.4149636	-2.5556	0.0061739	0.036947
i0_LQ_YHS_c1553/f1p0/564	188.37343	69.70876181	1.4342	0.0061787	0.036971
i3_LQ_YHS_c10639/f1p4/3330	146.5955499	15.76627283	3.2169	0.0061807	0.036979
i5_LQ_YHS_c2759/f1p9/5374	300.800701	119.8752884	1.3273	0.0061871	0.037013
i1_LQ_YHS_c5765/f2p0/1888	47.29389489	293.5666894	-2.634	0.0061956	0.037059
i1_LQ_YHS_c12714/f1p2/1990	406.8368971	67.42533896	2.5931	0.0062022	0.037089
i2_LQ_YHS_c7531/f1p0/2977	81.97953939	23.0038674	1.8334	0.0062016	0.037089
i2_LQ_YHS_c5270/f1p0/2104	0.544682597	13.45322701	-4.6264	0.0062113	0.037134
i5_LQ_YHS_c3052/f1p3/5268	24.09623077	85.04158024	-1.8194	0.0062105	0.037134
i1_HQ_YHS_c35114/f2p0/1442	1801.95594	650.4543339	1.47	0.0062131	0.03714
i3_LQ_YHS_c12421/f1p1/3132	30.94594568	1.061616699	4.8654	0.0062186	0.037168
i1_LQ_YHS_c17151/f2p0/1631	306.5987291	767.8775952	-1.3245	0.0062213	0.03718
i2_HQ_YHS_c1847/f5p3/2995	520.6438151	201.3924411	1.3703	0.006223	0.037185
i2_LQ_YHS_c6348/f1p0/2364	5.893734346	39.79005099	-2.7552	0.0062236	0.037185
i2_LQ_YHS_c38185/f1p0/2251	210.8063382	497.9643101	-1.2401	0.0062276	0.037204
i3_HQ_YHS_c21177/f3p2/3514	302.0647697	121.7835496	1.3105	0.0062297	0.037212
i4_HQ_YHS_c2082/f3p0/4773	403.9256751	42.22762182	3.2578	0.0062307	0.037213
i0_LQ_YHS_c2387/f1p0/986	24.31564354	83.99114116	-1.7884	0.0062326	0.03722
i1_LQ_YHS_c5596/f1p0/1996	148.0710059	52.81866966	1.4872	0.0062343	0.037222
i3_LQ_YHS_c17514/f1p0/3544	33.11784863	138.9995172	-2.0694	0.0062344	0.037222
i0_LQ_YHS_c2165/f1p1/551	229.6569078	90.29915129	1.3467	0.0062354	0.037223
i3_HQ_YHS_c16599/f2p0/3606	108.4374975	264.2598188	-1.2851	0.0062377	0.037232
i3_LQ_YHS_c15057/f1p1/3094	53.5742826	6.636763217	3.013	0.0062384	0.037232

i1_LQ_YHS_c27635/f1p0/1079	476.3554184	42.3004895	3.4933	0.0062408	0.037241
i2_LQ_YHS_c23483/f1p2/2438	32.80112837	105.5740302	-1.6864	0.0062436	0.037254
i2_LQ_YHS_c37078/f1p1/2494	64.8686054	171.7389347	-1.4046	0.0062546	0.03731
i5_LQ_YHS_c1696/f1p1/5333	3.797674038	31.82235671	-3.0669	0.0062539	0.03731
i2_LQ_YHS_c19962/f1p1/2108	433.360401	141.2074617	1.6178	0.0062574	0.037322
i4_LQ_YHS_c6662/f1p0/4672	1.438499347	16.9680553	-3.5602	0.006262	0.037345
i3_LQ_YHS_c3746/f1p0/3591	121.2590034	39.27310451	1.6265	0.0062634	0.037348
i2_LQ_YHS_c41050/f1p0/2187	9.762790747	0	Inf	0.0062693	0.037379
i0_LQ_YHS_c2635/f1p2/625	36.72251851	111.7234304	-1.6052	0.0062709	0.037384
i8_LQ_YHS_c134/f1p0/8013	4.101495763	36.61234772	-3.1581	0.0062764	0.037412
i2_LQ_YHS_c23206/f1p2/2496	127.421721	312.3323756	-1.2935	0.0062835	0.03745
i1_LQ_YHS_c27661/f1p0/1189	1136.405587	440.7821125	1.3663	0.0062845	0.037451
i1_LQ_YHS_c24233/f1p0/1860	16.0388618	0	Inf	0.0062859	0.037455
i4_LQ_YHS_c10187/f1p3/4524	5.387681544	33.81087304	-2.6498	0.0062908	0.037479
i1_LQ_YHS_c20586/f1p4/1862	2887.16145	936.0742882	1.625	0.0062948	0.037499
i1_LQ_YHS_c12667/f1p0/1941	212.9337676	501.8842725	-1.2369	0.0063021	0.037537
i4_LQ_YHS_c7250/f1p0/4414	362.0399417	150.2596812	1.2687	0.0063036	0.037542
i2_LQ_YHS_c49632/f1p3/2666	20.78696111	140.9202157	-2.7611	0.0063071	0.037558
i3_LQ_YHS_c4396/f1p0/3280	0	12.16068475	-Inf	0.0063086	0.037563
i2_LQ_YHS_c8086/f1p1/2408	2.315134405	23.08735948	-3.3179	0.0063158	0.037601
i2_LQ_YHS_c52446/f1p0/2385	191.5563316	70.74881603	1.437	0.0063207	0.037621
i9_LQ_YHS_c53/f1p0/9015	238.3801155	2716.674942	-3.5105	0.0063205	0.037621
i3_LQ_YHS_c12419/f1p3/3216	4.516923711	30.90809328	-2.7746	0.0063266	0.037651
i2_LQ_YHS_c43524/f1p0/2615	4.373031655	140.5581753	-5.0064	0.0063305	0.03767
i2_LQ_YHS_c22663/f1p3/2359	81.67870356	215.4330765	-1.3992	0.006332	0.037674
i2_LQ_YHS_c55660/f1p6/2635	0	11.04962139	-Inf	0.006343	0.037735
i1_HQ_YHS_c12082/f3p0/1906	318.123592	780.8753729	-1.2955	0.0063489	0.037763
i1_LQ_YHS_c23770/f1p1/1752	118.4182266	18.6722895	2.6649	0.0063501	0.037763
i2_LQ_YHS_c36377/f1p12/2018	22.42598615	289.1202862	-3.6884	0.0063508	0.037763
i3_LQ_YHS_c18266/f1p0/3377	44.34186734	129.0651816	-1.5414	0.0063506	0.037763
i2_HQ_YHS_c46146/f14p1/2542	498.0852892	1300.334615	-1.3844	0.0063589	0.037806
i2_HQ_YHS_c36926/f2p1/2321	190.053385	70.63973179	1.4279	0.0063635	0.037823
i2_LQ_YHS_c52050/f1p2/2261	417.4151117	172.5195457	1.2747	0.0063637	0.037823
i2_LQ_YHS_c9564/f1p2/2456	28.57612712	95.8285622	-1.7456	0.006364	0.037823
i3_LQ_YHS_c5382/f1p1/3979	23.57127415	1.439975754	4.0329	0.0063663	0.03783
i3_LQ_YHS_c9821/f1p1/3317	64.37970916	175.3100044	-1.4452	0.0063668	0.03783
i1_LQ_YHS_c13651/f1p1/1352	219.9629012	53.06744779	2.0514	0.0063677	0.037831
i2_LQ_YHS_c33762/f1p1/2900	53.28682879	149.6209736	-1.4895	0.0063712	0.037847
i2_LQ_YHS_c43651/f1p0/2041	178.3652487	1.799969692	6.6307	0.0063799	0.037893
i1_HQ_YHS_c20583/f2p1/1821	97.94634985	245.4530518	-1.3254	0.0063838	0.037903
i1_LQ_YHS_c13012/f1p0/1862	860.9816731	174.2093654	2.3052	0.0063829	0.037903
i2_LQ_YHS_c54652/f1p4/2324	14.27382886	59.30140467	-2.0547	0.0063833	0.037903
i3_LQ_YHS_c18589/f1p0/3353	211.1981818	514.422023	-1.2844	0.0063958	0.03797
i2_LQ_YHS_c24816/f1p0/2519	44.71071072	131.3587174	-1.5548	0.006398	0.037977
i3_LQ_YHS_c7281/f1p8/3753	63.68064382	172.3266964	-1.4362	0.0064099	0.038044
i1_LQ_YHS_c11165/f1p0/1395	111.4516637	279.771272	-1.3278	0.0064154	0.038067
i2_LQ_YHS_c13094/f1p3/2214	955.4363762	376.5105014	1.3435	0.0064153	0.038067
i3_LQ_YHS_c6611/f1p0/3173	96.24285244	247.138049	-1.3606	0.0064174	0.038069
i4_HQ_YHS_c2508/f2p4/4789	35.7263057	150.4991402	-2.0747	0.0064174	0.038069
i3_LQ_YHS_c12572/f1p0/3908	49.16687082	143.5446806	-1.5457	0.0064205	0.038084
i1_LQ_YHS_c8273/f1p0/1823	13.3162626	0	Inf	0.0064245	0.038102
i1_LQ_YHS_c33522/f1p0/1563	802.2805747	327.7965544	1.2913	0.0064256	0.038104
i1_LQ_YHS_c36061/f1p0/1712	123.576207	41.56260952	1.572	0.0064297	0.038124
i2_LQ_YHS_c5278/f1p4/2151	90.23329097	1.50450994	5.9063	0.0064363	0.038158
i5_HQ_YHS_c5007/f19p1/5735	1255.77638	493.2776778	1.3481	0.0064389	0.038169
i5_LQ_YHS_c888/f1p0/5095	50.33336754	145.6754548	-1.5332	0.0064409	0.038176
i1_HQ_YHS_c2628/f9p1/1607	931.4486234	2819.943028	-1.5981	0.0064442	0.038191
i5_LQ_YHS_c3315/f1p4/6016	0	58.72410807	-Inf	0.006445	0.038191
i2_LQ_YHS_c56114/f1p0/2815	407.8806686	126.7349969	1.6863	0.0064565	0.038254
i3_LQ_YHS_c3867/f1p3/3689	173.679774	66.0560191	1.3947	0.0064599	0.03827
i3_HQ_YHS_c1748/f3p0/3824	90.54419143	236.0852709	-1.3826	0.0064672	0.038309
i2_LQ_YHS_c11122/f2p1/2543	18.3384337	1.415488932	3.6955	0.0064707	0.038324
i3_HQ_YHS_c2201/f2p6/3151	270.9845562	30.26938569	3.1623	0.0064737	0.038338
i1_HQ_YHS_c15254/f13p0/1792	2466.99973	802.5975933	1.62	0.0064769	0.038347
i2_LQ_YHS_c40915/f1p45/2308	35.87531145	0	Inf	0.0064763	0.038347
i1_LQ_YHS_c26190/f1p14/1526	4801.373757	1322.024838	1.8607	0.0064791	0.038356
i1_LQ_YHS_c17461/f1p0/1771	51.18940177	0	Inf	0.0064839	0.038372
i1_LQ_YHS_c19388/f1p5/1566	271.2990792	52.90390053	2.3584	0.0064835	0.038372
i1_LQ_YHS_c4629/f1p1/1791	35.07496901	275.5191203	-2.9736	0.0064842	0.038372
i2_LQ_YHS_c22865/f1p1/2391	160.0224433	398.6334443	-1.3168	0.0064869	0.038383
i2_LQ_YHS_c35121/f1p0/2407	6.424584943	36.47711809	-2.5053	0.0064924	0.038411
i3_LQ_YHS_c12479/f1p0/3741	225.9810757	22.43968606	3.3321	0.0064946	0.038419
i3_LQ_YHS_c12217/f1p0/3273	99.01633612	31.00663334	1.6751	0.006496	0.038422
i1_LQ_YHS_c4736/f5p0/1766	703.1639681	71.9896603	3.288	0.0065036	0.03846
i4_LQ_YHS_c13460/f1p0/4116	18.65228775	66.37379342	-1.8313	0.006504	0.03846
i2_LQ_YHS_c22601/f1p9/2299	18.14909278	70.6569509	-1.9609	0.006509	0.038481
i3_LQ_YHS_c3895/f1p0/3141	16.80838441	214.3572332	-3.6728	0.0065089	0.038481
i0_LQ_YHS_c2465/f1p0/668	224.5811079	6.764173022	5.0532	0.00651	0.038482
i2_LQ_YHS_c30433/f4p5/2305	330.3906115	123.5378639	1.4192	0.0065128	0.038494
i1_LQ_YHS_c20943/f1p0/1951	106.2283638	315.6605554	-1.5712	0.0065187	0.038519
i2_LQ_YHS_c55491/f1p22/2284	591.9179318	41.18211889	3.8453	0.0065182	0.038519
i2_LQ_YHS_c38151/f1p6/2207	43.32626723	236.2104354	-2.4468	0.006522	0.038524
i2_LQ_YHS_c8354/f1p1/2111	190.4784899	71.30355853	1.4176	0.0065212	0.038524
i4_LQ_YHS_c11306/f1p0/4705	93.9224356	312.1551215	-1.7327	0.006521	0.038524
i1_LQ_YHS_c22030/f1p5/1841	42.19624494	0	Inf	0.0065266	0.038547
i1_LQ_YHS_c35399/f1p0/1864	1772.759844	643.4112917	1.4622	0.0065321	0.038573
i2_LQ_YHS_c13692/f1p6/2558	70.61647308	194.9712033	-1.4652	0.0065325	0.038573
i3_LQ_YHS_c11685/f1p4/3181	5.371305259	129.7226927	-4.594	0.0065375	0.038597
i2_LQ_YHS_c10249/f1p0/2536	4.937431853	32.62962681	-2.7243	0.0065401	0.038608

i2_LQ_YHS_c34293/f1p1/2173	1108.025977	444.9798776	1.3162	0.0065441	0.038627
i1_LQ_YHS_c28358/f1p0/1226	109.5150831	271.2609995	-1.3086	0.006547	0.038639
i2_LQ_YHS_c38201/f1p0/2445	337.7801264	137.8681104	1.2928	0.0065539	0.038675
i2_LQ_YHS_c7043/f1p6/2530	719.7132532	2018.53974	-1.4878	0.0065626	0.038722
i4_LQ_YHS_c6256/f1p4/4885	61.60082336	167.4252083	-1.4425	0.006565	0.038731
i1_HQ_YHS_c17346/f3p0/1788	160.0620281	56.33902688	1.5064	0.006572	0.03876
i2_LQ_YHS_c3986/f1p84/2887	0	10279.9869	-Inf	0.0065729	0.03876
i2_LQ_YHS_c4115/f1p2/2318	39.03651721	120.6681428	-1.6281	0.0065738	0.03876
i2_LQ_YHS_c51554/f1p0/2618	290.875453	117.1170178	1.3125	0.0065731	0.03876
i4_LQ_YHS_c13840/f1p0/4063	87.87960932	226.6127865	-1.3666	0.0065732	0.03876
i2_LQ_YHS_c54528/f1p3/2864	58.10340238	777.5611461	-3.7423	0.0065896	0.038848
i1_LQ_YHS_c10198/f1p2/1687	237.8183757	21.91610531	3.4398	0.0065906	0.038849
i2_LQ_YHS_c20881/f1p2/2420	433.8063879	175.2917986	1.3073	0.0065928	0.038857
i0_LQ_YHS_c1960/f1p0/689	42.58097275	137.1818482	-1.6878	0.0065983	0.038885
i2_HQ_YHS_c57706/f2p2/2076	105.8666865	269.512929	-1.3481	0.0066043	0.038916
i1_LQ_YHS_c23551/f1p4/1891	1053.385901	416.4497236	1.3388	0.006608	0.038929
i1_LQ_YHS_c27523/f1p0/1169	774.5523762	55.88507578	3.7928	0.0066089	0.038929
i4_LQ_YHS_c11165/f1p0/4365	15.4005517	63.77145023	-2.0499	0.0066089	0.038929
i2_LQ_YHS_c33721/f1p1/2150	75.44759788	21.53608769	1.8087	0.0066287	0.03904
i1_LQ_YHS_c22866/f1p2/1819	40.50603025	7.831911427	2.3707	0.0066317	0.039053
i2_LQ_YHS_c36584/f1p1/2522	109.6073272	267.9235809	-1.2895	0.0066341	0.039058
i2_LQ_YHS_c40004/f1p5/2550	234.5195621	569.4346633	-1.2798	0.0066336	0.039058
i1_LQ_YHS_c9031/f1p0/1581	225.2318153	22.99833847	3.2918	0.0066379	0.03907
i4_LQ_YHS_c7761/f1p0/4935	4.221523495	30.04466204	-2.8313	0.0066378	0.03907
i3_LQ_YHS_c20793/f1p0/3052	1.808614868	19.30546817	-3.4161	0.00664	0.039078
i0_LQ_YHS_c482/f2p0/891	3452.939051	232.208316	3.8943	0.0066435	0.039094
i0_LQ_YHS_c1005/f1p0/933	3005.574254	844.0501314	1.8322	0.0066478	0.039105
i2_HQ_YHS_c3359/f2p1/2130	253.3530229	32.80604981	2.9491	0.0066476	0.039105
i2_LQ_YHS_c22221/f1p0/2201	510.4991172	210.5970697	1.2774	0.0066472	0.039105
i2_LQ_YHS_c49443/f1p4/2505	64.08350354	174.7253263	-1.4471	0.0066534	0.039133
i3_LQ_YHS_c18361/f1p0/3805	131.7212843	324.2246585	-1.2995	0.0066629	0.039184
i1_LQ_YHS_c18601/f1p0/1988	115.3107452	851.5494073	-2.8846	0.0066701	0.039217
i3_LQ_YHS_c6110/f1p9/3453	329.2068422	57.96214011	2.5058	0.0066694	0.039217
i3_LQ_YHS_c7908/f1p0/3255	0	8.700991358	-Inf	0.006696	0.039365
i1_LQ_YHS_c22959/f1p0/1359	461.2437744	76.99094711	2.5828	0.0067011	0.03938
i2_LQ_YHS_c34744/f1p1/2606	300.2245212	122.3000637	1.2956	0.0067006	0.03938
i3_LQ_YHS_c10384/f1p0/3270	258.0365626	25.63674002	3.3313	0.0066996	0.03938
i1_LQ_YHS_c20548/f1p0/1984	0.904307434	15.02227114	-4.0541	0.0067034	0.039389
i1_LQ_YHS_c18941/f1p0/1961	73.10732701	217.6859711	-1.5742	0.0067065	0.039397
i2_LQ_YHS_c43650/f1p3/2029	160.0234928	424.7791159	-1.4084	0.006706	0.039397
i2_LQ_YHS_c49262/f1p0/2082	109.0320892	264.9450547	-1.2809	0.0067104	0.039416
i1_LQ_YHS_c21777/f1p2/1939	268.0372857	104.6916016	1.3563	0.0067212	0.039471
i3_LQ_YHS_c16887/f1p0/3496	149.9591195	34.67608261	2.1126	0.0067214	0.039471
i1_LQ_YHS_c8112/f1p2/1845	81.01939534	12.1234815	2.7405	0.0067242	0.039477
i2_LQ_YHS_c4684/f1p0/2618	143.7511924	15.18585795	3.2428	0.006724	0.039477
i1_LQ_YHS_c13041/f1p2/1827	1646.297852	619.7184412	1.4095	0.006731	0.039512
i2_LQ_YHS_c24523/f1p0/2181	0	8.662602559	-Inf	0.0067339	0.039525
i2_LQ_YHS_c51139/f1p1/2277	29.03192374	95.2400138	-1.7139	0.0067433	0.039575
i0_LQ_YHS_c2922/f1p0/457	1295.36907	4579.854879	-1.8219	0.0067444	0.039577
i3_LQ_YHS_c22561/f1p0/3014	0	8.650359149	-Inf	0.0067461	0.039582
i1_LQ_YHS_c24103/f1p0/1445	3772.991189	1140.396889	1.7262	0.0067496	0.039593
i4_LQ_YHS_c7993/f1p1/4427	120.3110638	362.8284782	-1.5925	0.0067497	0.039593
i4_LQ_YHS_c9703/f1p4/4676	0.359624837	11.15973187	-4.9557	0.0067541	0.039614
i2_LQ_YHS_c51508/f1p0/2808	16.50549615	0	Inf	0.0067553	0.039617
i1_LQ_YHS_c28278/f1p0/1272	1404.959566	445.7196952	1.6563	0.0067592	0.039635
i2_LQ_YHS_c23688/f1p0/2492	7.232381922	40.17670288	-2.4738	0.0067625	0.039649
i2_LQ_YHS_c34650/f1p0/2276	9.222713234	0	Inf	0.0067632	0.039649
i2_HQ_YHS_c6802/f3p4/2755	113.4626629	38.10291613	1.5742	0.0067702	0.039685
i2_LQ_YHS_c15052/f1p0/2060	18.9225515	112.4107919	-2.5706	0.0067749	0.039702
i2_LQ_YHS_c37782/f1p3/2228	271.9232186	633.4144361	-1.22	0.006775	0.039702
i4_LQ_YHS_c11923/f1p0/4778	149.5492995	46.60430299	1.6821	0.0067756	0.039702
i5_LQ_YHS_c1124/f1p0/5556	0.359624837	11.82908754	-5.0397	0.0067793	0.039719
i1_LQ_YHS_c23187/f1p0/1730	243.5800299	94.1193523	1.3718	0.0067822	0.03973
i3_LQ_YHS_c15004/f1p0/3045	0	8.61197035	-Inf	0.0067844	0.03973
i3_LQ_YHS_c19283/f1p0/3452	0	8.61197035	-Inf	0.0067844	0.03973
i4_LQ_YHS_c7551/f1p12/4686	186.148267	445.7123149	-1.2597	0.006783	0.03973
i2_HQ_YHS_c32598/f3p4/2721	20.42559739	99.53691283	-2.2849	0.0067892	0.039753
i2_LQ_YHS_c51300/f1p0/2667	28.32271477	90.01628819	-1.6682	0.0067907	0.039757
i1_LQ_YHS_c32572/f1p1/1998	103.7605532	5.755439875	4.1722	0.0067969	0.039783
i3_LQ_YHS_c19325/f1p0/3059	1288.525255	4762.10994	-1.8859	0.0067961	0.039783
i2_LQ_YHS_c39802/f1p4/2354	163.3229492	447.4870506	-1.4541	0.0067977	0.039784
i2_LQ_YHS_c20520/f1p3/2648	593.1040358	135.8427045	2.1263	0.0068097	0.039849
i2_HQ_YHS_c2218/f5p1/2836	572.6813118	237.1885924	1.2717	0.0068121	0.039858
i1_LQ_YHS_c28594/f1p0/1316	318.3486103	750.0230446	-1.2363	0.0068193	0.039895
i2_LQ_YHS_c26049/f2p4/2423	219.4237739	532.3620752	-1.2787	0.0068205	0.039897
i2_LQ_YHS_c19059/f1p0/2718	0	8.565801281	-Inf	0.0068308	0.039948
i3_LQ_YHS_c11694/f1p4/3939	0	8.565801281	-Inf	0.0068308	0.039948
i1_LQ_YHS_c9691/f1p0/1492	179.4516388	429.4346185	-1.2588	0.0068382	0.039982
i3_LQ_YHS_c16783/f1p0/3168	53.28380102	5.399909076	3.3027	0.0068377	0.039982
i1_LQ_YHS_c41860/f1p8/1834	36.59456112	0	Inf	0.0068448	0.040015
i0_LQ_YHS_c1975/f1p2/349	173.8850041	63.22223666	1.4596	0.0068501	0.04004
i3_LQ_YHS_c14357/f1p9/3054	52.36372885	2.353566188	4.4756	0.0068506	0.04004
i3_LQ_YHS_c11566/f1p2/3137	243.4281293	5.661955729	5.4261	0.0068577	0.040076
i1_LQ_YHS_c11047/f1p2/1803	37.02795104	341.0135544	-3.2031	0.0068609	0.04009
i1_LQ_YHS_c21132/f1p1/1847	217.8602839	27.32426767	2.9951	0.0068626	0.040095
i3_LQ_YHS_c14547/f1p0/3046	0	9.036498475	-Inf	0.0068643	0.0401
i3_LQ_YHS_c14110/f1p1/3039	273.6658927	6.956709791	5.2979	0.0068675	0.040114
i3_LQ_YHS_c8326/f1p1/3326	182.2896606	69.03336466	1.4009	0.0068799	0.040182
i1_LQ_YHS_c18413/f1p17/1846	232.6663133	91.31325405	1.3494	0.0068942	0.04026

i3_LQ_YHS_c19359/f1p7/3254	585.9754072	53.99766455	3.4399	0.0068958	0.04026
i6_LQ_YHS_c833/f1p0/6123	0	8.502925661	-Inf	0.0068949	0.04026
i0_LQ_YHS_c2220/f1p1/441	62.63961045	16.13452005	1.9569	0.0069014	0.040282
i2_LQ_YHS_c22991/f1p0/2343	24.92075108	2.952265965	3.0775	0.0069005	0.040282
i2_LQ_YHS_c32807/f1p1/2794	36.9869134	109.7353068	-1.5689	0.0069021	0.040282
i2_LQ_YHS_c33508/f1p1/2252	230.8229964	90.09820192	1.3572	0.0069059	0.040299
i1_HQ_YHS_c27189/f2p0/1092	462.6792867	189.3851917	1.2887	0.0069167	0.040358
i1_LQ_YHS_c7483/f1p0/1909	88.42681945	227.4504316	-1.363	0.0069188	0.040365
i2_LQ_YHS_c40995/f1p16/2369	8.508882425	41.60609379	-2.2898	0.0069239	0.040389
i2_LQ_YHS_c52710/f1p0/2963	276.588606	110.376819	1.3253	0.0069246	0.040389
i2_LQ_YHS_c28088/f1p1/2139	23.89768805	2.489349042	3.263	0.0069266	0.040396
i2_LQ_YHS_c38304/f1p0/2167	104.3490535	10.32126047	3.3377	0.0069301	0.040412
i3_HQ_YHS_c20263/f2p2/3045	36.62774693	112.3287249	-1.6167	0.0069393	0.04046
i1_LQ_YHS_c28910/f1p1/1253	354.6025031	872.6753973	-1.2992	0.0069412	0.040466
i2_LQ_YHS_c11702/f1p0/2231	54.13647719	145.7156626	-1.4285	0.0069462	0.040491
i2_LQ_YHS_c51456/f1p2/2474	0	18.78141441	-Inf	0.0069509	0.040513
i1_LQ_YHS_c8584/f1p4/1533	170.1241719	21.72747845	2.969	0.0069529	0.04052
i1_LQ_YHS_c27549/f1p0/1250	202.0416678	482.5367858	-1.256	0.0069595	0.040551
i3_LQ_YHS_c19518/f1p0/3312	106.9286438	35.91352953	1.574	0.00696	0.040551
i2_LQ_YHS_c45033/f1p1/2056	166.8310667	407.4954525	-1.2884	0.0069612	0.040554
i2_LQ_YHS_c25411/f1p0/2606	0	8.436732909	-Inf	0.0069634	0.040562
i2_LQ_YHS_c35155/f1p1/2771	155.5235318	57.96257358	1.4239	0.0069645	0.040563
i2_LQ_YHS_c3847/f1p1/2135	64.05825576	168.5025866	-1.3953	0.0069725	0.0406
i5_LQ_YHS_c2988/f1p0/5694	25.61232	87.21161615	-1.7677	0.0069718	0.0406
i2_HQ_YHS_c13552/f2p0/2534	206.2873369	79.20158249	1.3811	0.0069778	0.040606
i2_HQ_YHS_c45926/f3p0/2136	122.9841428	40.42879813	1.605	0.0069778	0.040606
i2_LQ_YHS_c53257/f1p0/2248	0	8.424489498	-Inf	0.0069762	0.040606
i3_HQ_YHS_c21199/f3p0/3128	1089.67464	3531.533727	-1.6964	0.0069765	0.040606
i4_LQ_YHS_c7367/f1p0/4703	185.5848626	72.70719685	1.3519	0.0069766	0.040606
i3_LQ_YHS_c3831/f1p0/3812	76.02806082	5.671947797	3.7446	0.0069927	0.040688
i2_LQ_YHS_c21941/f1p2/3008	86.6668667	220.3357837	-1.3462	0.0069937	0.040689
i2_LQ_YHS_c3270/f1p1/2447	55.74872983	153.4616701	-1.4609	0.0069968	0.040698
i2_LQ_YHS_c63024/f1p15/2960	122.5449283	30.532146	2.0049	0.0069964	0.040698
i0_LQ_YHS_c699/f1p0/963	2373.912108	584.7508735	2.0214	0.0070028	0.040728
i2_LQ_YHS_c21836/f1p3/2491	78.56801848	20.5529467	1.9346	0.0070043	0.040731
i1_LQ_YHS_c32042/f1p0/1648	111.832617	273.5708875	-1.2906	0.0070062	0.040737
i2_LQ_YHS_c3083/f1p0/2112	6.162734328	186.1557954	-4.9168	0.0070164	0.040792
i2_LQ_YHS_c37903/f1p0/2537	4.380986429	30.02124101	-2.7767	0.0070189	0.040796
i2_LQ_YHS_c42845/f3p5/2018	504.5809237	209.6266858	1.2673	0.0070186	0.040796
i1_HQ_YHS_c2965/f6p0/1737	1604.625559	566.9748593	1.5009	0.0070222	0.040811
i2_LQ_YHS_c52546/f1p0/2552	19.36899314	68.63999717	-1.8253	0.0070328	0.040867
i5_LQ_YHS_c3686/f1p0/5831	173.869693	39.01216319	2.156	0.0070382	0.040894
i2_HQ_YHS_c29503/f2p2/2624	49.47897761	142.4323515	-1.5254	0.0070402	0.040901
i2_LQ_YHS_c10635/f1p1/2545	525.0448362	1334.054258	-1.3453	0.0070456	0.040927
i2_LQ_YHS_c4590/f1p1/2690	294.5720115	118.9098802	1.3088	0.0070466	0.040928
i2_HQ_YHS_c60409/f4p3/2079	922.9347398	372.7204371	1.3081	0.0070485	0.040934
i1_LQ_YHS_c13903/f1p0/1306	210.8915442	495.9498818	-1.2337	0.0070518	0.040943
i3_LQ_YHS_c4061/f1p0/3773	108.6547047	262.3551896	-1.2718	0.0070514	0.040943
i0_LQ_YHS_c2919/f1p2/1001	1000.03934	406.1051223	1.3001	0.0070575	0.040967
i2_LQ_YHS_c41920/f1p1/2992	386.9196671	136.267865	1.5056	0.0070576	0.040967
i4_LQ_YHS_c5861/f1p0/4722	156.3465155	372.7305082	-1.2534	0.0070597	0.040975
i1_HQ_YHS_c41002/f12p0/1630	7352.956007	1709.494835	2.1048	0.0070633	0.04099
i2_LQ_YHS_c50908/f1p5/2628	135.4356444	47.67863612	1.5062	0.0070676	0.041006
i3_LQ_YHS_c16978/f1p0/3541	152.6685928	7.253828109	4.3955	0.007067	0.041006
i3_LQ_YHS_c11024/f1p4/3323	319.7306831	68.27277619	2.2275	0.0070719	0.041026
i2_LQ_YHS_c51528/f1p1/2086	125.2715964	42.77896697	1.5501	0.0070812	0.041074
i1_LQ_YHS_c43884/f1p0/1051	0	8.32322508	-Inf	0.0070835	0.041083
i3_LQ_YHS_c18046/f1p0/3572	435.857593	182.1510747	1.2587	0.0070867	0.041097
i2_LQ_YHS_c58490/f1p0/2036	238.4918989	96.34432313	1.3077	0.007088	0.041099
i1_HQ_YHS_c2414/f2p0/1774	112.5863581	276.230812	-1.2948	0.007089	0.0411
i1_LQ_YHS_c32888/f1p0/1725	225.2149806	31.05777811	2.8583	0.0070981	0.041148
i2_HQ_YHS_c26171/f3p0/2470	12.39892019	53.25709977	-2.1028	0.0071002	0.041155
i1_LQ_YHS_c23372/f1p0/1677	33.97064449	2.2061327	3.9447	0.0071046	0.041173
i1_LQ_YHS_c39100/f1p0/1230	1093.897075	221.4490984	2.3044	0.0071062	0.041173
i3_LQ_YHS_c4460/f1p2/3218	4.191315211	29.90773318	-2.835	0.0071068	0.041173
i5_LQ_YHS_c1427/f1p1/5827	2.712922302	31.59075815	-3.5416	0.0071056	0.041173
i2_LQ_YHS_c14943/f1p0/2183	11.62005098	71.82105712	-2.6278	0.007111	0.041193
i1_LQ_YHS_c34135/f1p2/1874	46.75130659	131.5105734	-1.4921	0.0071176	0.041226
i2_LQ_YHS_c34573/f1p1/2292	196.5656089	74.17892714	1.4059	0.0071241	0.041259
i2_LQ_YHS_c20165/f1p0/2438	45.79720971	6.373570557	2.8451	0.0071258	0.041264
i4_LQ_YHS_c2905/f1p0/4773	60.61985114	165.7422635	-1.4511	0.0071381	0.04133
i2_LQ_YHS_c56536/f1p1/2734	215.6632182	511.4134701	-1.2457	0.0071409	0.041341
i4_LQ_YHS_c5501/f1p2/4319	302.3006533	732.2469276	-1.2763	0.0071454	0.041362
i1_LQ_YHS_c23464/f1p3/1759	249.4701298	32.99400367	2.9186	0.0071498	0.041378
i1_LQ_YHS_c4542/f1p0/1517	2643.92765	885.7313853	1.5777	0.0071496	0.041378
i2_HQ_YHS_c13578/f2p1/2678	26.61775135	87.91197314	-1.7237	0.0071516	0.041384
i3_LQ_YHS_c8423/f1p0/3370	249.8824605	28.26072591	3.1444	0.0071619	0.041438
i2_HQ_YHS_c6977/f3p0/2136	2087.47396	217.8103379	3.2606	0.0071715	0.041489
i1_LQ_YHS_c35431/f1p2/1996	64.5467882	171.9611329	-1.4137	0.0071754	0.041506
i3_LQ_YHS_c17994/f1p5/3562	43.06835274	179.8520891	-2.0621	0.0072023	0.041657
i2_LQ_YHS_c19283/f1p0/2096	76.36416	10.08867634	2.9202	0.0072108	0.041691
i3_LQ_YHS_c6815/f1p23/4037	5.088473277	31.47124838	-2.6287	0.0072104	0.041691
i4_LQ_YHS_c8936/f1p0/4805	101.5100981	601.7652929	-2.5676	0.0072105	0.041691
i3_LQ_YHS_c11591/f1p6/3297	95.43057844	242.527719	-1.3456	0.0072152	0.041711
i1_LQ_YHS_c11920/f1p0/1864	9.432102053	101.1814699	-3.4232	0.0072231	0.041752
i1_LQ_YHS_c27879/f1p0/1176	175.3033562	65.31498129	1.4244	0.0072306	0.041791
i2_LQ_YHS_c6014/f1p1/2185	48.67961889	10.91490433	2.157	0.0072359	0.041815
i3_LQ_YHS_c10898/f1p0/3446	2.065852024	19.9247844	-3.2698	0.0072365	0.041815
i1_LQ_YHS_c8860/f1p0/1980	181.2465413	67.87226187	1.4171	0.0072389	0.041823

i2_LQ_YHS_c25682/f1p3/2771	40.91719178	115.7210795	-1.4999	0.0072428	0.041841
i2_HQ_YHS_c60951/f5p1/2346	67.9192042	187.9057614	-1.4681	0.0072443	0.041845
i1_LQ_YHS_c39260/f1p1/1979	95.32386327	0	Inf	0.0072453	0.041846
i1_LQ_YHS_c5862/f1p0/1754	450.7251315	167.6331869	1.4269	0.0072515	0.041876
i1_LQ_YHS_c4145/f1p14/2005	13.04010487	56.04190856	-2.1036	0.0072592	0.041916
i2_LQ_YHS_c37684/f1p0/2983	1.089365194	16.03997011	-3.8801	0.007262	0.041927
i3_LQ_YHS_c4135/f1p0/3453	19.17771111	77.58806741	-2.0164	0.0072643	0.041936
i1_LQ_YHS_c25496/f1p1/1392	128.8116946	45.62100269	1.4975	0.0072706	0.041956
i1_LQ_YHS_c37812/f2p13/1115	558.6661239	1412.457259	-1.3381	0.0072688	0.041956
i2_LQ_YHS_c34695/f1p0/2344	21.77348026	76.25765003	-1.8083	0.0072698	0.041956
i2_LQ_YHS_c23453/f1p6/2955	2.178730389	35.42332121	-4.0231	0.0072734	0.041968
i3_LQ_YHS_c8991/f1p0/3086	0	8.566394057	-Inf	0.0072767	0.041982
i2_LQ_YHS_c27103/f1p0/2544	138.1202996	48.57445421	1.5077	0.00729	0.042053
i1_LQ_YHS_c18265/f1p3/1505	25.26271911	85.21362031	-1.7541	0.0072928	0.042055
i2_LQ_YHS_c40926/f1p0/2974	81.66072483	211.2607767	-1.3713	0.0072923	0.042055
i4_LQ_YHS_c11958/f1p3/4330	8.313333981	40.28902515	-2.2769	0.0072922	0.042055
i2_LQ_YHS_c8970/f1p0/2638	154.1828486	15.45410652	3.3186	0.0073037	0.042112
i3_LQ_YHS_c2265/f2p4/3385	112.535099	3.653888725	4.9448	0.0073064	0.042123
i2_HQ_YHS_c57764/f2p0/2036	116.428234	29.65168847	1.9733	0.007309	0.042133
i2_LQ_YHS_c40652/f1p1/3002	8.442580256	76.33110937	-3.1765	0.0073147	0.042161
i2_LQ_YHS_c29069/f1p1/2082	95.77682126	242.9519719	-1.3429	0.0073187	0.042179
i2_LQ_YHS_c6923/f1p0/2906	138.7777445	34.22766044	2.0195	0.0073225	0.042196
i1_LQ_YHS_c9552/f1p1/1968	147.8094856	48.44874364	1.6092	0.0073279	0.042222
i4_LQ_YHS_c14789/f1p0/5024	1.90638909	19.89698045	-3.3836	0.0073334	0.042248
i1_LQ_YHS_c10908/f1p0/1928	1140.263012	463.6630393	1.2982	0.0073382	0.042268
i4_LQ_YHS_c12671/f1p10/4580	1.84597252	44.89058928	-4.604	0.0073386	0.042268
i3_LQ_YHS_c9193/f1p2/3758	88.73746025	225.4068199	-1.3449	0.0073407	0.042275
i2_LQ_YHS_c19676/f1p0/2647	21.20366957	74.52561187	-1.8134	0.0073564	0.042361
i2_LQ_YHS_c18197/f1p0/2271	53.69131606	4.958081625	3.4368	0.0073626	0.042386
i4_LQ_YHS_c13900/f1p0/4167	12.84202052	53.35330827	-2.0547	0.0073618	0.042386
i2_LQ_YHS_c28761/f1p0/2021	82.5319494	25.94716754	1.6694	0.0073637	0.042388
i2_LQ_YHS_c8725/f1p24/2315	421.636853	1041.291931	-1.3043	0.0073669	0.042401
i2_LQ_YHS_c34985/f1p6/2189	74.11287805	221.29302	-1.5782	0.0073919	0.04254
i2_LQ_YHS_c23019/f1p2/2469	5.198010325	32.7687268	-2.6563	0.0073971	0.042565
i2_LQ_YHS_c64193/f2p2/2129	1176.290157	465.4120559	1.3377	0.0073982	0.042566
i1_LQ_YHS_c18310/f1p0/1820	14.78116218	0.746133264	4.3082	0.0074061	0.042603
i2_LQ_YHS_c38116/f1p0/2509	42.37091496	5.659704386	2.9043	0.0074065	0.042603
i2_LQ_YHS_c55308/f1p2/2714	0	23.47800149	-Inf	0.0074132	0.042632
i2_LQ_YHS_c6819/f1p2/2880	138.4178145	5.839997743	4.5669	0.0074146	0.042632
i3_LQ_YHS_c17676/f1p0/3433	79.15087251	13.76811767	2.5233	0.0074149	0.042632
i5_LQ_YHS_c3799/f1p0/5689	313.4532981	130.4156238	1.2651	0.0074127	0.042632
i2_HQ_YHS_c5766/f2p0/2874	512.9117482	218.0083629	1.2343	0.0074167	0.042637
i1_LQ_YHS_c33190/f1p0/1721	363.3114086	146.6252629	1.3091	0.007419	0.04264
i3_LQ_YHS_c5978/f1p0/3485	0	27.13785261	-Inf	0.0074181	0.04264
i2_HQ_YHS_c17459/f2p4/2038	227.9439345	757.2933036	-1.7322	0.0074211	0.042647
i2_LQ_YHS_c55033/f1p3/2282	243.1545277	570.558993	-1.2305	0.0074243	0.04266
i2_LQ_YHS_c34929/f1p1/2156	59.57213513	12.13513214	2.2954	0.0074529	0.04282
i2_HQ_YHS_c30178/f3p2/2221	295.1142612	702.7410129	-1.2517	0.0074553	0.042828
i1_LQ_YHS_c10818/f1p3/1814	423.3102475	45.16258846	3.2285	0.0074576	0.042836
i4_LQ_YHS_c5435/f1p4/4684	50.3782216	139.5835222	-1.4703	0.0074623	0.042858
i2_HQ_YHS_c2531/f2p0/2256	0.544682597	12.68154114	-4.5412	0.0074737	0.042918
i2_LQ_YHS_c18586/f1p0/2783	66.46701766	175.711151	-1.4025	0.0074812	0.042956
i2_LQ_YHS_c13492/f1p13/2322	78.99553118	202.2175242	-1.3561	0.0074826	0.042959
i2_HQ_YHS_c1346/f5p1/2800	53.91356992	152.0806997	-1.4961	0.0074892	0.04299
i2_LQ_YHS_c20370/f1p0/2867	80.44222468	24.71035294	1.7028	0.0074897	0.04299
i1_HQ_YHS_c2655/f2p2/1640	270.3418002	737.6382791	-1.4481	0.0075167	0.04314
i4_LQ_YHS_c5906/f1p0/4690	2.565222194	22.31839796	-3.1211	0.0075189	0.043147
i2_LQ_YHS_c19240/f1p0/2220	195.0828503	48.84211001	1.9979	0.0075205	0.043152
i4_LQ_YHS_c10779/f1p2/4312	69.37247739	181.9835769	-1.3914	0.0075274	0.043186
i2_HQ_YHS_c15219/f2p0/2362	13.10721245	0	Inf	0.0075313	0.043203
i2_LQ_YHS_c44453/f1p1/2096	320.9733779	131.9251501	1.2827	0.0075333	0.043209
i2_LQ_YHS_c37310/f1p2/2440	90.49348526	28.4910587	1.6673	0.0075346	0.043212
i2_LQ_YHS_c38648/f1p0/2120	14.72581743	197.0364912	-3.742	0.0075367	0.043219
i2_LQ_YHS_c23949/f1p3/2414	17.2465326	65.59764441	-1.9273	0.0075403	0.043234
i1_LQ_YHS_c20264/f1p16/1889	57.808713	14.50812923	1.9944	0.0075488	0.043275
i1_LQ_YHS_c33406/f1p1/1379	138.8910859	329.5667419	-1.2466	0.0075492	0.043275
i2_LQ_YHS_c26391/f1p0/2108	29.66437336	0.707744466	5.3894	0.0075565	0.043312
i2_LQ_YHS_c11028/f1p6/3032	775.570073	2184.035848	-1.4937	0.0075616	0.043336
i1_HQ_YHS_c1557/f4p0/1884	590.2764796	179.1290594	1.7204	0.0075658	0.043341
i2_LQ_YHS_c22639/f1p2/2431	1.798124184	17.4086972	-3.2752	0.0075649	0.043341
i2_LQ_YHS_c59038/f1p1/2025	87.66052685	222.1000494	-1.3412	0.0075662	0.043341
i3_LQ_YHS_c4732/f1p1/3239	89.74531619	26.78129557	1.7446	0.0075652	0.043341
i1_LQ_YHS_c23843/f1p3/1559	678.4381322	239.9160952	1.4997	0.007568	0.043347
i1_LQ_YHS_c23966/f1p4/1888	0	14.08972281	-Inf	0.0075725	0.043368
i1_LQ_YHS_c21783/f2p0/1592	120.7077243	300.6968906	-1.3168	0.0075766	0.043386
i1_HQ_YHS_c4477/f6p0/1693	600.983257	149.8192381	2.0041	0.0075862	0.043425
i2_LQ_YHS_c11248/f1p9/2750	716.4258312	105.4342177	2.7645	0.0075862	0.043425
i2_LQ_YHS_c38228/f1p2/2038	9.44132897	0	Inf	0.0075846	0.043425
i1_LQ_YHS_c12397/f1p2/1988	40.05944379	265.1435517	-2.7266	0.0075879	0.043426
i3_LQ_YHS_c7922/f1p0/3877	316.2258854	17.95396019	4.1386	0.0075881	0.043426
i7_LQ_YHS_c298/f1p0/7084	38.53272744	129.2109565	-1.7456	0.0075912	0.043438
i3_LQ_YHS_c18157/f1p15/3213	199.198652	484.2721015	-1.2816	0.0075965	0.043464
i3_LQ_YHS_c12977/f1p0/3333	263.3687545	73.07892164	1.8496	0.0076002	0.043479
i2_LQ_YHS_c57929/f1p3/2036	37.89790347	6.970611768	2.4428	0.0076036	0.043494
i1_LQ_YHS_c14131/f1p0/1142	1001.439638	326.9861996	1.6148	0.0076073	0.043505
i2_LQ_YHS_c33644/f1p1/2164	203.5814682	484.1954437	-1.25	0.0076064	0.043505
i1_LQ_YHS_c22464/f1p4/1943	2774.010992	931.0074839	1.5751	0.007613	0.043532
i5_LQ_YHS_c3668/f1p0/5623	567.091544	237.2153712	1.2574	0.0076141	0.043533
i2_LQ_YHS_c23134/f1p2/2357	59.76167829	158.8799443	-1.4106	0.0076193	0.043557

i4_HQ_YHS_c12984/f2p7/4204	401.6293093	1010.880251	-1.3317	0.007621	0.043557
i4_LQ_YHS_c7974/f1p0/4435	93.03911791	29.01811701	1.6809	0.0076202	0.043557
i3_HQ_YHS_c11703/f3p1/3379	54.23631221	147.7136573	-1.4455	0.0076381	0.04365
i1_LQ_YHS_c8875/f1p0/1793	25.74110792	153.7391587	-2.5783	0.0076456	0.043684
i2_LQ_YHS_c18624/f1p2/2120	188.3842845	422.9230988	-1.1667	0.0076459	0.043684
i1_HQ_YHS_c17081/f2p0/1450	353.2076611	145.8891217	1.2756	0.0076496	0.0437
i5_LQ_YHS_c2408/f1p1/5196	11.74804187	54.57626043	-2.2159	0.0076692	0.043807
i1_LQ_YHS_c13580/f1p2/1177	1227.156307	381.4057074	1.6859	0.0076712	0.043813
i4_LQ_YHS_c12326/f1p0/4708	84.37029744	218.1736094	-1.3707	0.007673	0.043818
i5_LQ_YHS_c4434/f1p0/5186	578.8977705	247.0331153	1.2286	0.0076751	0.043825
i1_LQ_YHS_c20728/f1p2/1968	162.7216748	59.51664993	1.451	0.0076778	0.043836
i2_HQ_YHS_c2432/f7p4/2892	433.6011064	1099.191703	-1.342	0.0076801	0.043838
i3_LQ_YHS_c11538/f1p0/3240	371.6864229	126.7764238	1.5518	0.0076799	0.043838
i2_LQ_YHS_c8619/f1p2/2604	34.31387629	6.418081061	2.4186	0.0076837	0.043854
i1_LQ_YHS_c8035/f1p0/1757	28.91317653	89.3459865	-1.6277	0.0076912	0.043888
i2_HQ_YHS_c2907/f5p10/2724	721.2499179	112.0915974	2.6858	0.0076921	0.043888
i2_LQ_YHS_c34106/f1p2/2374	39.93148641	111.5269837	-1.4818	0.0076924	0.043888
i3_LQ_YHS_c11164/f1p15/3713	315.1631023	132.407298	1.2511	0.0077044	0.043951
i2_LQ_YHS_c57871/f2p3/2219	182.7723984	424.437129	-1.2155	0.0077091	0.043972
i1_LQ_YHS_c35967/f1p0/1954	9.365808258	0	Inf	0.0077172	0.044008
i2_HQ_YHS_c3627/f2p0/2147	37.39518361	110.649017	-1.5651	0.0077167	0.044008
i3_LQ_YHS_c4681/f1p0/3116	197.5470264	79.87089861	1.3065	0.0077196	0.044017
i3_LQ_YHS_c4197/f1p2/3564	14.90037613	61.44466176	-2.0439	0.007725	0.044042
i1_HQ_YHS_c37777/f3p2/1120	2793.640586	813.9078373	1.7792	0.007729	0.044058
i2_HQ_YHS_c13444/f3p1/2435	118.603428	297.5277209	-1.3269	0.0077304	0.044058
i3_LQ_YHS_c20708/f1p2/3062	39.26307934	7.698379916	2.3505	0.0077299	0.044058
i3_LQ_YHS_c12220/f1p0/3733	78.21231301	203.1601437	-1.3771	0.0077326	0.044065
i2_LQ_YHS_c36172/f1p0/2877	114.4711136	27.59030445	2.0528	0.0077352	0.044074
i1_LQ_YHS_c32834/f1p0/1912	100.8287674	32.91837208	1.6149	0.007742	0.044108
i5_LQ_YHS_c4749/f1p1/5043	236.0602432	51.45313995	2.1978	0.0077496	0.044146
i1_HQ_YHS_c29537/f26p0/1993	389.330619	162.5378639	1.2602	0.007761	0.044206
i2_LQ_YHS_c11915/f1p2/2193	40.06532939	118.8469244	-1.5687	0.0077641	0.044218
i1_LQ_YHS_c10823/f1p0/1957	150.9842684	27.20357235	2.4725	0.0077657	0.044222
i1_LQ_YHS_c19151/f1p0/1861	351.2635673	145.9986789	1.2666	0.0077728	0.044247
i1_LQ_YHS_c31864/f1p0/1633	94.70640176	15.55643673	2.606	0.0077727	0.044247
i2_LQ_YHS_c52679/f1p5/2771	73.23390931	246.114069	-1.7487	0.0077714	0.044247
i2_LQ_YHS_c54469/f1p0/2509	143.3100165	51.66199047	1.472	0.0077856	0.04431
i4_LQ_YHS_c5207/f1p0/4274	8.355305092	39.75264777	-2.2503	0.007786	0.04431
i5_LQ_YHS_c2934/f1p0/5616	0.771711468	32.44111972	-5.3936	0.0077866	0.04431
i2_LQ_YHS_c49025/f1p12/2310	703.0570039	1914.750448	-1.4454	0.0077881	0.044313
i1_LQ_YHS_c34111/f1p0/1877	583.5366847	251.6219163	1.2136	0.0077939	0.044341
i1_LQ_YHS_c25954/f1p1/1588	379.8275546	30.84304654	3.6223	0.0078035	0.044391
i1_LQ_YHS_c35205/f1p3/1386	307.531549	124.3564302	1.3063	0.0078083	0.044407
i5_LQ_YHS_c3529/f1p0/5499	2.712922302	42.6959479	-3.9762	0.0078079	0.044407
i0_LQ_YHS_c3236/f1p0/881	305.442814	848.2405622	-1.4736	0.0078117	0.044421
i1_LQ_YHS_c10531/f1p0/1470	287.219858	87.56726671	1.7137	0.0078217	0.04447
i1_LQ_YHS_c5382/f1p1/1536	1.044052767	38.22550955	-5.1943	0.0078221	0.04447
i2_LQ_YHS_c8827/f1p2/2985	453.6264674	1145.13506	-1.3359	0.0078442	0.044588
i4_LQ_YHS_c10371/f1p0/4268	73.35697319	20.21965138	1.8592	0.0078448	0.044588
i4_HQ_YHS_c1797/f5p2/4752	322.0988992	18.4897448	4.1227	0.0078656	0.044702
i0_LQ_YHS_c547/f1p0/868	326.6194035	132.2718349	1.3041	0.0078675	0.044707
i1_LQ_YHS_c20426/f1p0/1455	1287.297021	213.5389101	2.5918	0.0078701	0.044717
i2_LQ_YHS_c35736/f1p24/2664	1261.162725	92.15804714	3.7745	0.0078822	0.04478
i0_LQ_YHS_c836/f1p0/954	3219.535495	1042.798287	1.6264	0.0078893	0.044815
i1_LQ_YHS_c34247/f1p0/1921	134.9301577	44.20891111	1.6098	0.0078935	0.044834
i2_LQ_YHS_c49904/f1p15/2349	48.07361138	0.392261031	6.9373	0.0079156	0.044954
i2_LQ_YHS_c28920/f1p1/2065	6.090554933	50.56865878	-3.0536	0.0079169	0.044956
i2_LQ_YHS_c4630/f1p0/2468	0	8.35324083	-Inf	0.0079223	0.044976
i3_LQ_YHS_c10581/f1p1/3264	0	8.35324083	-Inf	0.0079223	0.044976
i3_LQ_YHS_c10775/f1p0/3929	5.905497172	34.08078019	-2.5288	0.0079257	0.04499
i2_LQ_YHS_c25396/f1p1/2968	83.27158564	210.8006237	-1.34	0.0079279	0.044998
i3_LQ_YHS_c22054/f1p13/3263	1407.331935	269.2653364	2.3859	0.0079387	0.045054
i2_HQ_YHS_c15813/f2p1/2099	102.4590072	33.17769438	1.6268	0.0079423	0.045068
i1_LQ_YHS_c10326/f1p0/1538	2166.967538	784.8044281	1.4653	0.0079485	0.045098
i2_LQ_YHS_c35207/f1p1/2716	30.86695559	93.20169152	-1.5943	0.0079593	0.045144
i2_LQ_YHS_c54296/f1p3/2646	41.35603407	0	Inf	0.0079583	0.045144
i4_LQ_YHS_c13538/f1p3/4052	240.1850912	97.93623506	1.2942	0.0079585	0.045144
i1_LQ_YHS_c3830/f1p7/1497	12.59701292	97.62979654	-2.9542	0.0079688	0.04519
i3_LQ_YHS_c11232/f1p0/3801	7.080873763	38.30441873	-2.4355	0.0079692	0.04519
i1_LQ_YHS_c23967/f1p11/1664	425.3057595	1067.087235	-1.3271	0.007972	0.0452
i1_LQ_YHS_c17017/f2p0/1694	437.5466062	185.0748638	1.2413	0.007974	0.045206
i5_LQ_YHS_c3620/f1p0/5710	255.1375904	105.2768724	1.2771	0.0079789	0.045229
i2_LQ_YHS_c55579/f1p0/2179	0	8.302608621	-Inf	0.0079817	0.045239
i1_LQ_YHS_c13560/f1p1/1382	289.7399939	690.0087616	-1.2519	0.0079922	0.045288
i1_LQ_YHS_c26017/f1p0/2011	1045.799626	429.0739169	1.2853	0.0079919	0.045288
i2_LQ_YHS_c26951/f1p0/2350	15.69307734	1.079981815	3.861	0.0079954	0.045301
i2_LQ_YHS_c52913/f1p0/2363	0	8.288706644	-Inf	0.0079981	0.045311
i1_HQ_YHS_c27200/f2p7/1187	407.4588333	986.1365157	-1.2751	0.0080002	0.045317
i1_LQ_YHS_c22540/f1p3/1612	7.662922387	37.76519722	-2.3011	0.0080026	0.045326
i2_HQ_YHS_c32638/f2p4/2121	823.6489095	82.46210373	3.3202	0.008006	0.045339
i1_LQ_YHS_c25979/f1p0/1509	176.4045919	65.57805419	1.4276	0.0080118	0.045367
i4_LQ_YHS_c6853/f1p0/4776	0	8.276463233	-Inf	0.0080127	0.045367
i2_LQ_YHS_c54717/f1p1/2061	41.3691636	121.3712253	-1.5528	0.0080147	0.045373
i2_LQ_YHS_c8008/f1p2/2602	16.00865352	68.41752487	-2.0955	0.0080174	0.045383
i2_LQ_YHS_c29013/f1p0/2047	267.6632545	97.94121075	1.4504	0.0080215	0.045401
i3_LQ_YHS_c14929/f1p0/3071	347.9794712	122.9330808	1.5011	0.0080263	0.045422
i2_LQ_YHS_c40694/f1p3/2435	0	8.262561257	-Inf	0.0080293	0.045434
i4_LQ_YHS_c8880/f1p0/4549	293.1720558	106.7361988	1.4577	0.0080317	0.045437
i7_LQ_YHS_c163/f1p0/7854	4.018825683	30.39233235	-2.9189	0.0080314	0.045437

i0_LQ_YHS_c975/f1p5/715	2466.454727	70.09359582	5.137	0.0080493	0.045531
i3_LQ_YHS_c6556/f1p0/3541	96.46171593	32.18673357	1.5835	0.0080522	0.045542
i5_LQ_YHS_c5569/f1p0/5003	1.34660235	17.09380654	-3.6661	0.008054	0.045547
i2_LQ_YHS_c38978/f1p44/2747	0	8.238074435	-Inf	0.0080587	0.045568
i1_LQ_YHS_c17579/f1p3/1882	824.8120241	150.1823503	2.4574	0.0080609	0.045576
i1_LQ_YHS_c18529/f1p4/1866	12.11227179	52.40310736	-2.1132	0.0080646	0.045591
i2_LQ_YHS_c54423/f1p3/2365	326.4654134	127.4037598	1.3575	0.0080698	0.04561
i3_LQ_YHS_c5766/f1p0/3526	65.71295462	17.71359579	1.8913	0.0080695	0.04561
i2_LQ_YHS_c3819/f1p2/2879	32.56601666	110.205058	-1.7588	0.0080794	0.045659
i2_LQ_YHS_c49588/f1p1/2772	75.46428772	198.534925	-1.3955	0.0080889	0.045707
i1_LQ_YHS_c28076/f1p0/1173	149.8087375	480.9766322	-1.6828	0.0080928	0.045723
i2_LQ_YHS_c37413/f1p13/2534	3532.881091	271.5747442	3.7014	0.0080935	0.045723
i1_HQ_YHS_c2333/f2p4/1791	206.6637018	67.43138045	1.6158	0.0080955	0.045728
i2_LQ_YHS_c11241/f1p0/2168	0	8.205807342	-Inf	0.0080977	0.045736
i1_LQ_YHS_c8986/f1p0/1917	123.1925981	41.60218388	1.5662	0.0081002	0.045744
i1_LQ_YHS_c14606/f1p3/1321	2822.849438	948.3080022	1.5737	0.008103	0.045752
i5_LQ_YHS_c4292/f1p0/5654	0	102.2615786	-Inf	0.0081035	0.045752
i2_LQ_YHS_c18830/f1p3/2500	188.5858993	446.7339644	-1.2442	0.0081137	0.045805
i1_LQ_YHS_c19894/f1p0/1671	1344.395398	193.0621878	2.7998	0.0081154	0.045809
i3_LQ_YHS_c4210/f1p4/3230	1376.965865	536.488952	1.3599	0.0081179	0.045818
i2_LQ_YHS_c28804/f1p1/2010	420.6978028	1019.816232	-1.2775	0.0081198	0.045823
i1_HQ_YHS_c37666/f4p1/1171	736.9827559	1921.510044	-1.3825	0.0081247	0.045845
i2_LQ_YHS_c8027/f1p0/2424	0	8.167418544	-Inf	0.0081446	0.045952
i2_LQ_YHS_c26085/f1p2/2894	385.8314507	163.401294	1.2396	0.008147	0.045955
i2_LQ_YHS_c8127/f1p2/2245	252.8589137	103.3605507	1.2906	0.008147	0.045955
i2_LQ_YHS_c10727/f1p2/2962	560.4510026	156.1957258	1.8432	0.0081545	0.045987
i3_LQ_YHS_c14066/f1p0/3025	591.8612369	251.3122013	1.2358	0.0081546	0.045987
i3_LQ_YHS_c14935/f1p0/3055	134.692394	321.864057	-1.2568	0.0081577	0.045999
i2_LQ_YHS_c53717/f1p6/2354	0	8.155175133	-Inf	0.0081597	0.046005
i2_LQ_YHS_c55189/f1p0/2116	0	8.153516567	-Inf	0.0081618	0.046011
i2_LQ_YHS_c24316/f1p0/2740	0	8.149053428	-Inf	0.0081673	0.046037
i2_LQ_YHS_c3536/f1p2/2533	207.6462274	12.82285292	4.0173	0.0081683	0.046038
i2_LQ_YHS_c25516/f1p0/2833	24.87589701	83.27064072	-1.7431	0.0081737	0.04606
i2_LQ_YHS_c5639/f1p4/2265	187.4226828	71.42251507	1.3918	0.0081741	0.04606
i2_LQ_YHS_c34077/f1p1/2241	195.6458503	74.28090409	1.3972	0.0081757	0.046063
i1_LQ_YHS_c25109/f1p0/1723	0	8.141273156	-Inf	0.008177	0.046065
i1_LQ_YHS_c5597/f1p4/1717	514.4843071	214.8690891	1.2597	0.0081804	0.046073
i2_HQ_YHS_c24536/f3p3/2397	224.3582319	543.1849205	-1.2756	0.0081796	0.046073
i1_HQ_YHS_c17297/f3p1/1773	308.9477362	738.8663866	-1.258	0.0081848	0.046088
i2_LQ_YHS_c49365/f1p0/2257	153.8036916	56.79250496	1.4373	0.0081842	0.046088
i2_LQ_YHS_c52678/f1p2/2435	1280.854635	95.24929333	3.7493	0.0081877	0.046098
i2_LQ_YHS_c24311/f1p1/2463	12.73501938	59.53734661	-2.225	0.0081895	0.046103
i8_LQ_YHS_c6/f2p0/8877	9.080431991	190.3984253	-4.3901	0.0081906	0.046104
i5_LQ_YHS_c1693/f1p1/5488	62.66115312	190.0938081	-1.6011	0.0081953	0.046125
i2_LQ_YHS_c8517/f1p2/1837	594.7581914	1529.283555	-1.3625	0.008205	0.046174
i2_LQ_YHS_c6657/f1p2/2130	254.0173192	104.1840148	1.2858	0.0082095	0.046189
i6_LQ_YHS_c530/f1p0/6221	0	10.72635769	-Inf	0.008209	0.046189
i1_LQ_YHS_c12598/f1p0/1723	11.3334277	135.7627273	-3.5824	0.0082131	0.046204
i2_LQ_YHS_c7695/f1p0/2160	285.6223098	15.40619867	4.2125	0.0082175	0.046223
i1_LQ_YHS_c28021/f1p0/1325	0	13.7158269	-Inf	0.0082216	0.046241
i2_LQ_YHS_c10476/f1p0/2344	292.9377662	87.32054108	1.7462	0.0082265	0.046263
i2_LQ_YHS_c56470/f1p0/2602	172.8053744	21.64457915	2.9971	0.008229	0.046271
i4_LQ_YHS_c8703/f1p0/4221	6.382613832	34.33398078	-2.4274	0.0082336	0.046292
i2_HQ_YHS_c2712/f5p3/2707	182.4541954	428.1736763	-1.2307	0.0082351	0.046295
i2_LQ_YHS_c52225/f1p0/2913	103.9074886	405.8738808	-1.9657	0.0082378	0.046305
i4_HQ_YHS_c2251/f2p0/4906	74.53014417	315.3981434	-2.0813	0.0082446	0.046338
i4_LQ_YHS_c5192/f1p10/4463	93.66172907	231.7726904	-1.3072	0.0082487	0.04635
i5_HQ_YHS_c764/f2p0/5669	183.6996199	13.74422363	3.7405	0.0082479	0.04635
i2_HQ_YHS_c13690/f2p0/2393	0	8.082860676	-Inf	0.0082501	0.046353
i3_LQ_YHS_c2467/f1p4/3193	139.3746328	341.0638789	-1.2911	0.0082513	0.046354
i1_LQ_YHS_c25951/f1p0/1965	126.085729	44.73920634	1.4948	0.0082673	0.046438
i3_LQ_YHS_c20801/f1p0/3057	1570.255355	5707.695117	-1.8619	0.0082685	0.04644
i0_LQ_YHS_c1050/f1p0/507	13.66411623	0.719987877	4.2463	0.0082706	0.046446
i1_LQ_YHS_c14512/f1p6/1218	185.6447513	41.37971158	2.1655	0.0082719	0.046448
i1_HQ_YHS_c1089/f15p0/1991	2605.277121	911.0907193	1.5158	0.0082745	0.046457
i5_LQ_YHS_c842/f1p6/5189	0	8.06003242	-Inf	0.0082791	0.046478
i5_LQ_YHS_c3306/f1p19/5423	86.60372036	10.76415371	3.0082	0.0082814	0.046485
i2_LQ_YHS_c49692/f1p1/2033	32.51147732	113.7362742	-1.8067	0.0082875	0.046514
i2_LQ_YHS_c48943/f1p0/2281	31.59128546	3.215458624	3.2964	0.0082887	0.046515
i2_LQ_YHS_c34029/f1p0/2745	311.5192162	127.9780925	1.2834	0.0083013	0.046581
i2_LQ_YHS_c9199/f1p2/2546	60.98837259	16.07670034	1.9236	0.0083029	0.046584
i3_LQ_YHS_c10470/f1p1/3807	249.5468808	82.61392126	1.5949	0.0083055	0.046589
i4_LQ_YHS_c9044/f1p2/4593	137.4549035	50.46285213	1.4457	0.0083058	0.046589
i2_LQ_YHS_c19294/f1p1/2168	26.74572548	88.60925253	-1.7281	0.0083102	0.046609
i3_LQ_YHS_c5082/f1p4/3779	90.55230776	27.47012169	1.7209	0.0083251	0.046687
i3_LQ_YHS_c9964/f1p0/3259	60.51713316	16.01216616	1.9182	0.008327	0.046692
i2_LQ_YHS_c40334/f1p1/2332	3.037725395	24.07666172	-2.9866	0.008329	0.046698
i3_LQ_YHS_c4806/f1p0/3586	52.92292079	179.7933245	-1.7644	0.0083408	0.046759
i3_HQ_YHS_c5633/f3p2/3577	505.4878377	158.21284	1.6758	0.0083481	0.046794
i4_LQ_YHS_c3374/f1p30/4660	102.8643777	260.5450674	-1.3408	0.0083494	0.046796
i1_LQ_YHS_c8462/f1p0/1669	2396.327659	846.533796	1.5012	0.0083534	0.046813
i2_LQ_YHS_c51476/f1p0/2457	0	7.999961374	-Inf	0.0083565	0.046825
i1_LQ_YHS_c24155/f1p0/1682	71.9790101	20.62415582	1.8032	0.0083615	0.046837
i2_LQ_YHS_c39077/f1p1/2240	141.7104266	52.84987097	1.423	0.0083613	0.046837
i2_LQ_YHS_c52140/f1p0/2437	104.2328254	16.2569937	2.6807	0.0083602	0.046837
i1_LQ_YHS_c43995/f1p3/1033	234.2001946	549.727487	-1.231	0.0083662	0.046849
i2_LQ_YHS_c55665/f1p0/2504	91.80827688	29.4709221	1.6393	0.0083667	0.046849
i3_LQ_YHS_c3842/f1p1/3840	188.2721039	72.38346013	1.3791	0.0083649	0.046849
i1_LQ_YHS_c3655/f1p0/1590	27.99376495	4.076758283	2.7796	0.0083678	0.046851

i5_HQ_YHS_c5084/f4p1/5247	106.4987975	264.4448901	-1.3121	0.0083825	0.046927
i3_LQ_YHS_c13498/f1p1/3613	58.633353	14.78581664	1.9875	0.0083855	0.046939
i1_HQ_YHS_c39884/f3p0/1923	331.7660471	105.7069294	1.6501	0.0083925	0.046972
i0_HQ_YHS_c51/f11p0/773	35855.97512	1177.218254	4.9288	0.0083965	0.046975
i2_LQ_YHS_c10898/f1p0/2945	4.796414377	32.65071628	-2.7671	0.0083965	0.046975
i2_LQ_YHS_c53445/f1p2/2241	76.66242642	22.32060975	1.7801	0.0083969	0.046975
i4_LQ_YHS_c8439/f1p0/5007	70.51952101	20.23023622	1.8015	0.0083969	0.046975
i3_LQ_YHS_c19047/f1p1/3229	334.6853748	800.0067843	-1.2572	0.0084104	0.047045
i1_LQ_YHS_c24479/f1p0/1866	285.9975294	116.6813922	1.2934	0.0084135	0.047052
i2_LQ_YHS_c60470/f4p3/2119	121.1080482	294.2791282	-1.2809	0.0084135	0.047052
i1_LQ_YHS_c37834/f2p4/1358	438.5469489	184.507012	1.2491	0.0084185	0.047069
i2_HQ_YHS_c19557/f2p2/2339	909.4088179	159.786467	2.5088	0.0084182	0.047069
i3_HQ_YHS_c1607/f4p0/3626	51.28934811	141.291507	-1.4619	0.008421	0.047077
i2_LQ_YHS_c14600/f1p2/2002	72.26744643	202.9016943	-1.4894	0.0084296	0.04712
i2_LQ_YHS_c5406/f1p0/2812	120.8510671	8.846805826	3.7719	0.0084305	0.04712
i1_LQ_YHS_c18031/f1p0/1504	94.31788145	451.0364605	-2.2576	0.0084374	0.047148
i2_LQ_YHS_c41166/f1p1/2342	75.14760096	21.93612899	1.7764	0.0084375	0.047148
i7_LQ_YHS_c112/f1p0/7725	30.54548544	4.84291523	2.657	0.0084416	0.047165
i3_LQ_YHS_c12711/f1p10/3842	118.2807982	3.395159205	5.1226	0.0084591	0.047257
i1_LQ_YHS_c18532/f2p0/1932	226.2893984	531.693353	-1.2324	0.0084649	0.047285
i1_HQ_YHS_c11484/f2p0/1747	96.78708505	30.98546366	1.6432	0.0084667	0.047289
i3_LQ_YHS_c3219/f1p0/3135	432.1930737	179.7239679	1.2659	0.0084719	0.047312
i2_LQ_YHS_c3610/f1p0/2240	18.66831699	86.84953018	-2.2179	0.0084745	0.047322
i3_LQ_YHS_c12559/f1p1/3088	138.2728609	41.66225492	1.7307	0.0084758	0.047323
i2_LQ_YHS_c55180/f1p0/2076	75.47666209	23.42168104	1.6882	0.0084801	0.047342
i3_LQ_YHS_c18917/f1p0/3449	0	11.01004704	-Inf	0.0084891	0.047387
i2_LQ_YHS_c22023/f1p2/2843	239.5533775	96.28974034	1.3149	0.0084962	0.047421
i3_LQ_YHS_c13065/f1p0/3263	115.4845859	41.5703898	1.4741	0.0084992	0.047432
i2_HQ_YHS_c17420/f2p0/2666	58.60600255	190.0939229	-1.6976	0.0085042	0.047454
i1_LQ_YHS_c22571/f1p0/1839	48.69202557	378.3186393	-2.9578	0.0085169	0.04752
i2_LQ_YHS_c53161/f1p2/2302	27.81251524	4.070636578	2.7724	0.0085192	0.047527
i1_LQ_YHS_c6857/f1p0/1571	263.6248272	106.3251403	1.31	0.0085251	0.047555
i2_LQ_YHS_c33758/f1p9/2852	9.072477217	43.68418436	-2.2675	0.0085262	0.047556
i2_LQ_YHS_c20765/f1p3/2608	40.33306561	116.657418	-1.5322	0.008528	0.04756
i2_LQ_YHS_c5796/f1p2/2503	19.54656286	69.70718346	-1.8344	0.0085325	0.04758
i2_LQ_YHS_c4765/f1p0/2674	24.00976101	2.95838767	3.0207	0.0085353	0.04759
i3_LQ_YHS_c14432/f1p0/3087	655.2803377	47.98021757	3.7716	0.0085393	0.047606
i0_LQ_YHS_c667/f1p0/678	1356.852788	531.1715099	1.353	0.0085459	0.047627
i2_LQ_YHS_c20890/f1p1/2250	946.9471935	393.3821878	1.2674	0.0085459	0.047627
i4_LQ_YHS_c10743/f1p5/4716	180.2756505	425.9641474	-1.2405	0.0085441	0.047627
i3_LQ_YHS_c22560/f1p2/3000	9.017937873	44.26463878	-2.2953	0.0085476	0.047631
i1_LQ_YHS_c11726/f1p0/1421	27.95594894	1.459999436	4.2591	0.0085517	0.047637
i2_LQ_YHS_c49710/f1p38/2629	800.5540008	7.559872706	6.7265	0.0085509	0.047637
i2_LQ_YHS_c52091/f1p12/2523	7898.776678	857.0700442	3.2041	0.0085497	0.047637
i1_LQ_YHS_c23678/f1p0/1765	144.4612642	340.275647	-1.236	0.008554	0.047645
i4_LQ_YHS_c10817/f1p0/4525	55.90595365	233.3640909	-2.0615	0.0085641	0.047695
i1_LQ_YHS_c23837/f1p2/1613	0.544682597	12.32320577	-4.4998	0.0085699	0.047722
i1_LQ_YHS_c24483/f1p3/1726	134.6055856	49.10743425	1.4547	0.008579	0.047765
i3_LQ_YHS_c16670/f1p0/4059	35.35792906	107.9759773	-1.6106	0.0085798	0.047765
i8_LQ_YHS_c109/f1p0/8219	9.027970196	176.9729627	-4.293	0.0085806	0.047765
i1_LQ_YHS_c33490/f1p0/1590	160.0473823	58.6331148	1.4487	0.0085869	0.047795
i1_LQ_YHS_c24913/f1p2/1416	1302.804458	5.789365534	7.814	0.0085882	0.047797
i3_LQ_YHS_c1866/f2p0/3605	132.4096735	48.56880552	1.4469	0.0085896	0.047799
i2_LQ_YHS_c5761/f1p3/2283	124.0741602	291.157034	-1.2306	0.0085957	0.047827
i2_LQ_YHS_c21177/f1p2/2299	90.41426781	29.20445186	1.6304	0.0086061	0.047874
i2_LQ_YHS_c35919/f1p1/2438	101.7136959	33.36517523	1.6081	0.0086059	0.047874
i3_LQ_YHS_c4632/f1p1/3147	37.93064766	111.8289184	-1.5599	0.0086075	0.047876
i1_HQ_YHS_c7891/f2p3/1988	639.0839805	274.3952174	1.2198	0.0086108	0.047887
i2_LQ_YHS_c3959/f1p4/2864	189.4112585	73.28429459	1.3699	0.0086118	0.047887
i3_HQ_YHS_c1894/f2p0/3196	228.1281102	525.0582868	-1.2026	0.0086124	0.047887
i2_LQ_YHS_c35022/f1p3/2108	79.20251215	23.23870287	1.769	0.0086151	0.047896
i3_LQ_YHS_c14181/f1p6/3105	14.54790903	0	Inf	0.0086179	0.047907
i2_LQ_YHS_c18773/f1p0/2652	30.49601792	5.113888161	2.5761	0.0086197	0.047911
i1_LQ_YHS_c5827/f1p2/1468	362.3634355	152.3813351	1.2498	0.0086276	0.047949
i1_LQ_YHS_c23557/f1p40/1606	3.639483246	26.31944443	-2.8543	0.0086361	0.047976
i2_LQ_YHS_c18052/f1p2/2718	10.62177738	0	Inf	0.008636	0.047976
i2_LQ_YHS_c19674/f1p3/2483	53.93851252	13.30579353	2.0193	0.0086381	0.047976
i3_LQ_YHS_c12593/f1p0/3126	63.00892894	5.678069502	3.4721	0.0086356	0.047976
i4_LQ_YHS_c2773/f1p0/4357	6.250017867	34.74393393	-2.4748	0.0086382	0.047976
i5_LQ_YHS_c1347/f1p1/5484	198.1961742	80.67935427	1.2967	0.0086383	0.047976
i1_LQ_YHS_c3923/f1p1/1393	142.4647254	334.0498213	-1.2295	0.0086474	0.048021
i3_LQ_YHS_c14870/f1p7/3031	164.1775785	63.39640831	1.3728	0.0086496	0.048028
i4_LQ_YHS_c4004/f1p0/4616	411.5745872	148.3311963	1.4723	0.0086601	0.04808
i1_LQ_YHS_c21473/f1p0/1659	707.948635	295.6725767	1.2596	0.0086678	0.048117
i0_LQ_YHS_c2246/f1p3/918	5053.459392	1421.72894	1.8296	0.0086807	0.048183
i4_LQ_YHS_c11963/f1p48/4626	82.31228051	203.9492486	-1.309	0.0086909	0.048235
i2_LQ_YHS_c54557/f1p0/2958	14.97082502	58.4505701	-1.9651	0.0086925	0.048238
i3_LQ_YHS_c10269/f1p1/3482	46.41011884	130.8078453	-1.4949	0.008703	0.04829
i2_HQ_YHS_c2620/f10p2/2548	595.103734	251.6058421	1.242	0.0087193	0.048376
i4_LQ_YHS_c6366/f1p3/4939	342.1793512	805.813526	-1.2357	0.008725	0.048402
i1_LQ_YHS_c10343/f1p6/2000	615.5202865	240.5177182	1.3557	0.0087285	0.048411
i4_LQ_YHS_c11919/f1p0/4681	84.91719403	23.98989205	1.8236	0.0087288	0.048411
i3_LQ_YHS_c19881/f1p1/3582	33.92532368	126.7368543	-1.9014	0.0087311	0.048413
i5_HQ_YHS_c654/f3p0/5653	81.74430326	302.0040018	-1.8854	0.0087302	0.048413
i1_LQ_YHS_c28912/f1p12/1130	209.3125359	42.34053687	2.3055	0.008737	0.04844
i1_LQ_YHS_c35585/f1p0/1701	399.6616682	137.9821308	1.5343	0.0087391	0.048446
i2_LQ_YHS_c52000/f1p4/2303	21.08571939	69.73937034	-1.7257	0.0087418	0.048456
i2_HQ_YHS_c11691/f4p0/2269	148.6802195	8.264812599	4.1691	0.0087437	0.048461
i2_HQ_YHS_c61067/f16p3/2215	517.1584824	1276.320478	-1.3033	0.0087468	0.048473

i1_LQ_YHS_c3654/f1p0/1864	2.435967545	67.92312757	-4.8013	0.0087545	0.04851
i2_LQ_YHS_c28831/f1p0/2081	97.4076558	26.72675345	1.8658	0.008757	0.048518
i3_LQ_YHS_c14074/f1p0/3056	173.3659618	53.54316023	1.695	0.0087639	0.048551
i3_LQ_YHS_c12619/f1p0/3493	159.1710189	380.9784154	-1.2591	0.0087712	0.04858
i4_LQ_YHS_c4070/f1p0/4994	41.55930155	8.982549133	2.21	0.0087713	0.04858
i1_LQ_YHS_c21190/f1p4/1587	127.9543104	45.01630097	1.5071	0.0087836	0.048643
i3_LQ_YHS_c17559/f1p0/3426	442.5477062	33.05308915	3.743	0.0087878	0.048661
i1_LQ_YHS_c36091/f1p8/1511	90.23831727	222.0694804	-1.2992	0.0087959	0.048694
i2_HQ_YHS_c6993/f2p4/2414	377.8321779	41.25277478	3.1952	0.0087949	0.048694
i3_LQ_YHS_c19083/f1p1/3063	91.85643877	234.1810322	-1.3502	0.0088019	0.048722
i3_LQ_YHS_c18450/f1p56/3507	28.65085918	88.67094259	-1.6299	0.008804	0.048728
i5_LQ_YHS_c4371/f1p0/5824	140.7542857	7.132540009	4.3026	0.0088067	0.048737
i3_LQ_YHS_c18871/f1p0/3700	17.93603235	1.061616699	4.0785	0.00881	0.04875
i2_LQ_YHS_c5447/f1p28/2404	2040.068012	753.06374	1.4378	0.0088158	0.048777
i1_HQ_YHS_c40306/f10p6/1861	2131.548659	732.4348886	1.5411	0.0088173	0.048779
i5_LQ_YHS_c2065/f1p0/5239	99.30119541	410.7535597	-2.0484	0.0088204	0.048791
i2_LQ_YHS_c5478/f1p0/2430	6.859730492	41.3745359	-2.5925	0.0088225	0.048797
i3_LQ_YHS_c18436/f1p1/3439	40.89827121	114.6061062	-1.4866	0.0088251	0.048806
i2_LQ_YHS_c54808/f1p2/2473	8.781690459	0	Inf	0.0088268	0.04881
i1_HQ_YHS_c4545/f3p0/1905	96.77417814	237.4305363	-1.2948	0.0088427	0.048881
i1_LQ_YHS_c24046/f1p0/1822	263.3572274	90.03978944	1.5484	0.0088423	0.048881
i2_HQ_YHS_c4335/f3p2/2218	206.6832495	473.5097239	-1.196	0.0088438	0.048881
i2_LQ_YHS_c10812/f1p1/2565	1.536273569	16.75995799	-3.4475	0.0088436	0.048881
i0_HQ_YHS_c221/f4p0/933	9899.713902	1140.468176	3.1178	0.0088518	0.04892
i2_LQ_YHS_c55885/f1p4/2823	6.777060412	93.47250514	-3.7858	0.0088725	0.049029
i0_LQ_YHS_c860/f1p0/749	135.1663883	48.67958899	1.4733	0.0088756	0.049039
i1_HQ_YHS_c31598/f2p0/1581	1084.173438	434.313124	1.3198	0.0088764	0.049039
i2_LQ_YHS_c10788/f1p1/2689	23.54856227	0	Inf	0.0088781	0.049043
i4_LQ_YHS_c6957/f1p0/4755	85.64626177	383.4648725	-2.1626	0.008885	0.049075
i2_LQ_YHS_c11354/f1p1/2666	1.089365194	15.6855051	-3.8479	0.0088911	0.049103
i2_LQ_YHS_c20559/f1p4/2781	178.3675058	466.0623817	-1.3857	0.0088936	0.049112
i3_LQ_YHS_c8205/f1p1/3829	440.3877432	57.82793673	2.9289	0.0089098	0.049195
i1_HQ_YHS_c37877/f2p9/1136	733.7766136	306.9937232	1.2571	0.0089141	0.049213
i2_LQ_YHS_c22357/f1p0/2452	18.54988332	70.2368057	-1.9208	0.0089204	0.049242
i1_LQ_YHS_c44019/f1p0/1047	124.7278557	310.2038852	-1.3144	0.0089254	0.049264
i0_LQ_YHS_c990/f1p0/868	590.6929582	30.05575945	4.2967	0.0089278	0.049272
i2_HQ_YHS_c32604/f2p0/2336	0	10.61166431	-Inf	0.0089307	0.049282
i1_LQ_YHS_c13534/f1p0/1302	9.095536134	42.20194944	-2.2141	0.0089345	0.049287
i1_LQ_YHS_c7123/f1p3/1708	30.30841587	125.3432823	-2.0481	0.0089325	0.049287
i2_HQ_YHS_c45350/f4p1/2249	147.6090089	470.0792607	-1.6711	0.0089335	0.049287
i3_LQ_YHS_c5721/f1p0/3203	43.16141056	120.1322791	-1.4768	0.0089376	0.049298
i2_LQ_YHS_c41918/f1p1/2750	0	8.274804667	-Inf	0.0089415	0.049314
i3_LQ_YHS_c14750/f1p0/3020	0	8.207465908	-Inf	0.0089468	0.049338
i1_LQ_YHS_c34285/f1p9/1833	484.390158	101.1327358	2.2599	0.0089481	0.049339
i3_LQ_YHS_c19296/f1p0/3740	1.07887451	13.7448164	-3.6713	0.0089578	0.049387
i1_LQ_YHS_c7911/f2p0/1875	273.6745464	114.7800779	1.2536	0.0089595	0.049391
i1_LQ_YHS_c28138/f1p0/1175	422.8611825	179.3200167	1.2376	0.0089607	0.049392
i2_LQ_YHS_c24300/f1p0/2241	260.829169	24.68420756	3.4014	0.0089637	0.049402
i1_HQ_YHS_c11847/f2p4/1809	105.5232608	253.4021007	-1.2639	0.0089673	0.049417
i3_LQ_YHS_c8230/f1p0/3848	80.40139765	204.5356248	-1.3471	0.0089685	0.049418
i2_LQ_YHS_c6691/f1p0/2869	193.8132012	77.83613299	1.3162	0.0089848	0.049502
i1_LQ_YHS_c5266/f1p0/1960	483.0928126	62.0177287	2.9615	0.0090002	0.049581
i1_LQ_YHS_c6456/f1p1/1998	45.94016829	124.8304457	-1.4421	0.0090014	0.049582
i0_LQ_YHS_c1941/f1p0/340	94.88672637	27.34606968	1.7949	0.0090046	0.049583
i1_LQ_YHS_c13204/f1p2/2004	275.5126898	16.07835891	4.0989	0.0090043	0.049583
i3_LQ_YHS_c18206/f1p0/3075	31.51113454	98.62616764	-1.6461	0.0090025	0.049583
i2_LQ_YHS_c22983/f1p1/2822	1458.946918	135.9179026	3.4241	0.0090073	0.049592
i1_LQ_YHS_c20440/f1p16/2028	36.54209933	0	Inf	0.0090114	0.049609
i2_HQ_YHS_c17822/f2p4/2288	101.4259034	245.6637943	-1.2763	0.0090194	0.049647
i2_LQ_YHS_c63403/f1p3/2362	26.48053355	91.94726502	-1.7959	0.0090282	0.04969
i2_LQ_YHS_c6485/f2p9/2307	70.3559281	3.892594563	4.1759	0.0090311	0.0497
i2_LQ_YHS_c23281/f1p10/2824	1057.64376	142.0133128	2.8968	0.0090323	0.049702
i0_LQ_YHS_c1892/f1p1/972	67.14973184	211.2399207	-1.6534	0.0090353	0.049706
i4_LQ_YHS_c13626/f1p0/4208	316.8671443	102.1176551	1.6336	0.0090355	0.049706
i4_LQ_YHS_c7927/f1p0/4682	3.037725395	23.82737104	-2.9716	0.0090362	0.049706
i2_LQ_YHS_c21384/f1p2/2692	38.1845435	110.6065986	-1.5344	0.0090442	0.049744
i1_HQ_YHS_c16752/f3p0/1993	28.35417845	122.8495887	-2.1153	0.0090556	0.049801
i2_LQ_YHS_c8639/f1p8/2089	274.2423537	635.5272835	-1.2125	0.0090619	0.04983
i1_LQ_YHS_c29016/f1p0/1245	76.39260429	194.7918955	-1.3504	0.0090675	0.049855
i1_LQ_YHS_c10725/f1p75/1466	1305.895894	42.56806509	4.9391	0.0090752	0.049892
i2_LQ_YHS_c49714/f1p0/2420	230.6989907	93.94573275	1.2961	0.0090862	0.049946
i2_HQ_YHS_c15645/f7p0/2468	686.0897094	113.6071256	2.5943	0.0090889	0.049956
i2_LQ_YHS_c52346/f1p13/2493	1115.406685	462.0305618	1.2715	0.0090903	0.049958
i2_LQ_YHS_c18819/f1p3/2457	122.4134764	300.1906092	-1.2941	0.0090954	0.04998
