# Supplementary material for: Integration of full-length transcriptomics and targeted metabolomics to identify benzylisoquinoline alkaloid biosynthetic genes in Corydalis yanhusuo
Source: Hortic Res. 2021 Jan 10;8:16. doi: 10.1038/s41438-020-00450-6 (PMC7797006; doi:10.1038/s41438-020-00450-6)
Supplement: Supplementary file 15 — BIA synthetic pathway-related unigenes and putative enzymes [file 41438_2020_450_MOESM15_ESM.pdf]

Table S3. BIA synthetic pathway-related unigenes and putative enzymes

Unigene ID	Putative	Subject ID	Species	Identit	Align	Mismat	Gap	Gene	Gene	Sub	Subj	Evalue	Score
c35142/f1p1/2101	3-OHase	OVA00542.1	*Macleaya cordata*	74.6	398	86	6	112	1299	27	411	1.50E-181	624
c53720/f1p1/2154	3-OHase	OVA00542.1	*Macleaya cordata*	75.4	398	83	6	110	1297	27	411	4.90E-183	629
c12257/f1p0/3283	3-OHase	OVA00542.1	*Macleaya cordata*	72.7	311	70	6	3024	2098	27	324	3.90E-131	457.2
c45126/f1p1/2080	3-OHase	OVA00542.1	*Macleaya cordata*	70.3	256	61	6	110	871	27	269	7.50E-104	365.9
c20949/f1p2/1391	4’-OMT	sp|Q7XB10.1|	*Papaver somniferu*	75.6	348	80	1	79	1107	10	357	7.10E-159	548.1
c37524/f6p1/1275	4’-OMT	sp|Q7XB10.1|	*Papaver somniferu*	75.4	346	80	1	80	1102	10	355	2.10E-157	543.1
c32618/f1p0/1832	4-HPPDC	OVA02291.1	*Macleaya cordata*	86	444	62	0	296	1627	76	519	1.10E-226	773.9
c5010/f1p1/1862	4-HPPDC	OVA02291.1	*Macleaya cordata*	85.3	491	72	0	13	1485	29	519	8.40E-248	844
c8977/f1p2/4550	4-HPPDC	OVA02291.1	*Macleaya cordata*	85	374	56	0	352	1473	146	519	4.50E-186	640.2
c28392/f1p0/1305	6-OMT	AAQ01669.1	*Papaver somniferu*	71.7	350	92	4	114	1157	1	345	1.90E-150	520
c27717/f1p0/1347	6-OMT	AAQ01669.1	*Papaver somniferu*	71.1	350	94	4	78	1121	1	345	2.20E-149	516.5
c29075/f1p2/1221	6-OMT	AAQ01669.1	*Papaver somniferu*	69.4	350	100	4	76	1119	1	345	2.00E-144	500
c14617/f1p0/1365	6-OMT	AAQ01669.1	*Papaver somniferu*	66.2	222	69	3	170	829	32	249	1.20E-83	298.1
c3747/f1p5/1439	6-OMT	AAQ01669.1	*Papaver somniferu*	62.9	350	131	6	88	1131	4	345	2.60E-116	406.8
c23730/f2p0/1758	BBE	AAC61839.1	*Papaver somniferu*	62.3	494	185	1	21	1502	31	523	2.60E-190	652.9
c17006/f3p0/1786	BBE	AAC61839.1	*Papaver somniferu*	62.3	494	185	1	158	1639	31	523	3.40E-190	652.5
c17058/f2p0/1721	CFS	ADB89213.1	*Papaver somniferu*	72.7	472	126	2	123	1532	22	492	9.60E-206	704.1
c10904/f4p29/1899	CFS	ADB89213.1	*Papaver somniferu*	69.8	470	139	2	128	1531	24	492	1.30E-198	680.6
c6431/f1p0/1853	CFS	ADB89213.1	*Papaver somniferu*	67.1	434	140	2	241	1536	60	492	4.60E-169	582.4
c11806/f1p0/4369	CNMT	sp|Q7XB08.1|	*Papaver somniferu*	67.3	254	83	0	3295	4056	98	351	1.9E-104	369
c28135/f1p0/1348	CNMT	sp|Q7XB08.1|	*Papaver somniferu*	65.3	346	118	1	72	1109	8	351	2.6E-137	476.5
c24066/f1p0/2926	CNMT	sp|Q7XB08.1|	*Papaver somniferu*	64.8	347	120	1	1526	2566	7	351	1.1E-137	478.8
c1985/f4p11/1737	CNMT	sp|Q7XB08.1|	*Papaver somniferu*	60.3	343	146	0	151	1179	8	350	9.7E-121	421.8
c16778/f6p0/1763	CNMT	sp|Q7XB08.1|	*Papaver somniferu*	60.3	343	146	0	174	1202	8	350	9.6E-121	421.8
c4736/f5p0/1766	DBOX	AGL44334.1	*Papaver somniferu*	65.8	500	166	4	136	1620	30	529	3.3E-201	689.1
c18483/f1p1/1812	DBOX	AGL44334.1	*Papaver somniferu*	61.6	503	187	4	141	1631	27	529	8.9E-186	637.9
c41475/f3p0/1804	MSH	AGC92398.1	*Papaver somniferu*	64.4	500	160	6	174	1667	42	525	6.50E-189	648.3
c18372/f1p0/2001	MSH	AGC92398.1	*Papaver somniferu*	62.9	522	196	6	88	1647	20	525	1.90E-184	633.6
c39834/f10p10/197	MSH	AGC92398.1	*Papaver somniferu*	62.4	534	177	8	54	1649	14	525	3.40E-191	656
c51026/f1p24/2331	NCS	sp|Q4QTJ2.1|	*Papaver somniferu*	68.4	206	59	1	1427	2026	1	206	8.40E-80	286.2
c21575/f152p0/345	NCS	sp|Q4QTJ2.1|	*Papaver somniferu*	68	206	59	1	2469	3065	1	206	2.80E-79	285
c5528/f2p13/3744	NCS	sp|Q4QTJ2.1|	*Papaver somniferu*	68	206	59	1	2767	3363	1	206	3.00E-79	285
c11157/f1p0/3506	NCS	sp|Q4QTJ2.1|	*Papaver somniferu*	68	206	59	1	2527	3123	1	206	2.80E-79	285
c13667/f1p0/3404	NCS	sp|Q4QTJ2.1|	*Papaver somniferu*	68	206	59	1	2430	3026	1	206	3.60E-79	284.6
c1427/f9p1/4754	NCS	sp|Q4QTJ2.1|	*Papaver somniferu*	68	206	59	1	3775	4371	1	206	3.80E-79	285
c25433/f1p23/3357	NCS	sp|Q4QTJ2.1|	*Papaver somniferu*	67.5	206	60	1	2384	2980	1	206	7.90E-79	283.5
c13052/f2p3/3631	NCS	sp|Q4QTJ2.1|	*Papaver somniferu*	67.5	206	60	1	2657	3253	1	206	8.50E-79	283.5
c1890/f4p0/4699	NCS	sp|Q4QTJ2.1|	*Papaver somniferu*	67.5	206	60	1	3708	4304	1	206	1.10E-78	283.5
c1926/f4p0/4663	NCS	sp|Q4QTJ2.1|	*Papaver somniferu*	67.5	206	60	1	3677	4273	1	206	1.10E-78	283.5
c49550/f1p24/2168	NCS	sp|Q4QTJ2.1|	*Papaver somniferu*	64.5	217	59	2	1156	1785	1	206	4.80E-77	276.9
c11949/f1p5/3118	NCS	sp|Q4QTJ2.1|	*Papaver somniferu*	61.7	206	73	1	2357	2956	1	206	4.50E-66	241.1
c17808/f2p4/2277	NMCH	sp|I3V6B1.1|	*Papaver somniferu*	70.5	254	72	2	138	896	36	287	3.70E-104	367.1
c39871/f17p0/1638	NMCH	sp|I3V6B1.1|	*Papaver somniferu*	66.7	433	139	3	50	1345	6	434	2.50E-171	589.7
c20382/f1p0/1777	NMCH	sp|I3V6B1.1|	*Papaver somniferu*	66	373	125	1	135	1253	36	406	8.00E-146	505
c1899/f1p7/5251	P6H	AGC92397.1	*Papaver somniferu*	69	248	73	1	3340	2597	293	536	1E-101	360.1
c26207/f1p2/1728	P6H	AGC92397.1	*Papaver somniferu*	67.4	282	88	1	752	1597	259	536	4.1E-116	406.4
c3246/f6p2/1907	P6H	AGC92397.1	*Papaver somniferu*	66.9	362	105	3	633	1715	191	538	7E-141	488.8
c21069/f1p0/1820	P6H	AGC92397.1	*Papaver somniferu*	66.8	512	152	3	142	1674	38	532	2.7E-206	706.1
c5577/f2p0/1891	P6H	AGC92397.1	*Papaver somniferu*	65.9	536	171	6	245	1831	8	538	1.4E-205	703.7
c15063/f7p11/1822	P6H	AGC92397.1	*Papaver somniferu*	65.5	536	173	6	88	1674	8	538	1.5E-204	700.3
c29531/f19p0/1873	P6H	AGC92397.1	*Papaver somniferu*	65.1	502	164	3	194	1696	47	538	2.3E-197	676.4
c6211/f1p2/1934	P6H	AGC92397.1	*Papaver somniferu*	65.1	502	164	3	191	1693	47	538	2.4E-197	676.4
c40297/f16p1/1786	P6H	AGC92397.1	*Papaver somniferu*	64.9	502	165	3	190	1692	47	538	1.1E-196	674.1
c20953/f1p0/1821	P6H	AGC92397.1	*Papaver somniferu*	64.3	384	126	3	443	1591	165	538	2.2E-147	510.4
c7006/f1p0/1871	P6H	AGC92397.1	*Papaver somniferu*	60	248	99	2	191	931	47	288	1.1E-80	288.9
c2471/f1p3/996	SanR	D5JWB3.1	*Eschscholzia cali*	75.1	202	67	1	62	853	6	273	1.00E-112	403
c43660/f1p1/1142	SanR	D5JWB3.1	*Eschscholzia cali*	74.5	204	70	1	76	882	1	273	1.00E-112	401
c17984/f1p1/2525	SanR	D5JWB3.1	*Eschscholzia cali*	69.5	189	83	1	1448	2254	3	273	1.00E-106	382
c39645/f1p3/1119	SanR	D5JWB3.1	*Eschscholzia cali*	69	187	84	2	115	912	5	273	1.00E-103	372
c589/f1p0/961	SanR	D5JWB3.1	*Eschscholzia cali*	71.4	192	77	1	62	814	6	273	1.00E-102	368
c39042/f1p1/2249	SanR	D5JWB3.1	*Eschscholzia cali*	64.2	170	95	0	48	791	9	273	1.00E-92	338
c25582/f3p0/1524	SOMT1	sp|Q39522|SM	*Coptis japonica*	63.2	218	127	3	84	1118	37	378	1.0E-121	433
c27036/f4p6/1229	SOMT1	sp|Q39522|SM	*Coptis japonica*	60.9	215	138	3	98	1153	29	378	1.0E-120	429
c8446/f1p0/1571	SPS	ADB89214.1	*Papaver somniferu*	80.6	284	55	0	190	1041	33	316	3.10E-134	466.5
c39970/f36p0/1817	SPS	ADB89214.1	*Papaver somniferu*	77.5	463	100	2	191	1573	33	493	1.90E-212	726.5
c19894/f1p0/1671	SPS	ADB89214.1	*Papaver somniferu*	76	434	100	2	251	1546	62	493	1.80E-193	663.3
c5951/f1p54/2944	SPS	ADB89214.1	*Papaver somniferu*	74.8	484	115	3	1383	2819	12	493	1.50E-211	724.2
c1817/f8p1/1699	SPS	ADB89214.1	*Papaver somniferu*	73.6	470	118	4	181	1584	28	493	1.10E-201	690.6
c6450/f4p0/1686	SPS	ADB89214.1	*Papaver somniferu*	73	486	123	3	105	1544	10	493	2.00E-211	723
c23592/f1p0/1721	SPS	ADB89214.1	*Papaver somniferu*	72.9	469	122	3	148	1548	28	493	2.70E-200	686
c1016/f17p0/1838	STOX	OVA00265.1	*Macleaya cordata*	65.5	349	184	3	60	1643	8	537	0	718
c29443/f6p1/1720	STOX	OVA00265.1	*Macleaya cordata*	65.5	349	184	3	107	1690	8	537	0	717
c3575/f1p0/1947	STOX	AGL44336.1	*Papaver somniferu*	62	327	200	4	179	1753	5	527	0	651
c15163/f1p3/2206	STOX	OVA00265.1	*Macleaya cordata*	61.9	309	190	5	240	1718	38	531	0	639
c26267/f1p0/1789	STOX	AGL44336.1	*Papaver somniferu*	61	322	206	1	25	1599	1	527	0	649
c51889/f1p3/2124	STOX	OVA00265.1	*Macleaya cordata*	60.2	317	210	7	55	1617	12	531	0	645
c42734/f2p0/1369	TNMT	sp|Q108P1.1|	*Papaver somniferu*	71	345	98	1	130	1164	16	358	1.30E-152	527.3
c40365/f2p0/1356	TNMT	sp|Q108P1.1|	*Papaver somniferu*	70.7	345	99	1	100	1134	16	358	1.60E-152	526.9
c8264/f2p0/1582	TNMT	sp|Q108P1.1|	*Papaver somniferu*	69.3	345	104	1	97	1131	16	358	1.10E-144	501.1
c32536/f1p1/1707	TNMT	sp|Q108P1.1|	*Papaver somniferu*	63.4	186	67	1	857	1414	174	358	1.50E-70	255
c36847/f1p0/1504	TNMT	sp|Q108P1.1|	*Papaver somniferu*	61.3	367	126	3	96	1196	8	358	9.10E-128	444.9
c28752/f1p0/1293	TNMT	sp|Q108P1.1|	*Papaver somniferu*	60.8	367	128	3	13	1113	8	358	6.70E-127	441.8
c11715/f1p9/1886	TyDC1	P54768.1	*Papaver somniferu*	79.4	510	90	3	104	1588	1	510	2.70E-246	839
c32638/f2p4/2121	TyDC1	P54768.1	*Papaver somniferu*	79.2	510	91	3	338	1822	1	510	6.20E-247	841.3
c27377/f5p7/2155	TyDC2	P54769.1	*Papaver somniferu*	81.1	529	88	4	232	1791	1	526	2.90E-260	885.6
c33007/f1p6/2484	TyDC2	P54769.1	*Papaver somniferu*	81.1	529	88	4	223	1782	1	526	7.50E-260	884.4
c2693/f4p2/1575	TyrAT	ADC33123.1	*Papaver somniferu*	68.9	411	128	0	70	1302	6	416	2.6E-173	596.3
c18980/f1p1/1356	COR	Q9SQ68	*Papaver somniferu*	63.9	202	114	3	277	1224	6	318	1.00E-113	407
c8373/f1p0/4184	COR	Q9SQ68	*Papaver somniferu*	63.3	200	116	3	3132	4079	6	318	1.00E-112	404
c38807/f1p0/1120	COR	Q9SQ68	*Papaver somniferu*	62.7	200	119	3	20	976	3	318	1.00E-109	394
