# Supplementary material for: Integration of full-length transcriptomics and targeted metabolomics to identify benzylisoquinoline alkaloid biosynthetic genes in Corydalis yanhusuo
Source: Hortic Res. 2021 Jan 10;8:16. doi: 10.1038/s41438-020-00450-6 (PMC7797006; doi:10.1038/s41438-020-00450-6)
Supplement: Supplementary file 16 — Sequence information of all OMT candidates and reference sequences [file 41438_2020_450_MOESM16_ESM.pdf]

Table S4. Sequence information of all OMT candidates and reference sequences.

Unigene ID / Enzyme name	Accession number (UniProt)	Length	Predicted CDS sequence
4'OMT (Coptis japonica)	>sp|Q9LEL5|4OMT_COPJA 3'-hydrox	350	MAFHGKDDVLDIKAQAHVWKIIYGFADSLVLRCAVELGIVDIIDNNNQPMALADLASKLPVSDVNCDNLY
4'OMT (Eschscholzia californica)	>tr|J7M6V4|J7M6V4_ESCCA 3'-hydr	353	MGLEFNEEVDIKAQAHLWNIIYGFADSLVLRSAVELGIADIIKNNNGSITVSELASKLPISNVNSDNLYR
4'OMT2 (Papaver somniferum)	>sp|Q7XB10|4OMT2_PAPSO 3'-hydro	357	MGSLDAKPAAATQEVSIKDQAQLWNIIYGFADSLVLRCAVEIGIADIIKNNDGAITLAQLAAKLPITNVS
4'OMT1 (Papaver somniferum)	>sp|Q7XB11|4OMT1_PAPSO 3'-hydro	354	MGSSIDAETHEVDIKDQAQLWNIIYGYADSLVLRCTVEIGIADIIKNNNGSITLSELVSKLPLSNVNSDN
6OMT (Coptis japonica)	>sp|Q9LEL6|6OMT_COPJA (RS)-norc	347	MEVKKDNLSSQAKLWNFIYGFAESLVLKCAVQLDLANIIHNSGTSMTLSELSSRLPSQPVNEDALYRVMR
6OMT (Papaver somniferum)	>sp|Q6WUC1|6OMT_PAPSO (RS)-norc	346	METVSKIDQQNQAKIWKQIYGFAESLVLKCAVQLEIAETLHNNVKPMSLSELASKLPVAQPVNEDRLFRI
6OMT (Thalictrum flavum subsp. glaucum)	>sp|Q5C9L7|6OMT_THLFG (RS)-norc	350	MEMINKENLSSQAKLWNFIYGFADSLVLKSAVQLDLANIIHNHGSPMTLSELSLHLPSQPVNQDALYRVL
CoOMT (Coptis japonica)	>sp|Q8H9A8|COOMT_COPJA Columbam	351	MDTPNTFQNDDEIKAQAQVWKHMFGFAETIMLRSTVSLGIPDIIHNNGPVTLSQLVTHLPLKSTSIDRFH
N7OMT (Papaver somniferum)	>sp|C7SDN9|N7OMT_PAPSO Norretic	357	MEVVSQIDQENQAIIWKQIYGFSESLLLKCAVQCEIAETIHNHGTPMSILELAAKLPIDQPVNIDRLYRV
R7OMT (Papaver somniferum)	>sp|Q6WUC2|7OMT_PAPSO (R,S)-ret	355	MDTAEERLKGQAEIWEHMFAFVDSMALKCAVELGIPDIINSHGRPVTISEIVDSLKTNTPSSSPNIDYLT
SOMT (Argemone mexicana)	>tr|A0A1P7ZIM6|A0A1P7ZIM6_ARGME	277	MDVNNGIVETSIYMSRVGKLGSYSAISMALRAAIELNLFEIISGFGPEAQVSATDLVSKMSNIINNPNAP
SOMT (Coptis japonica)	>sp|Q39522|SMT_COPJA (S)-scoule	381	MCTSLSELKCPVFSTKRKLLLEFALRTSVDMAAQEGVNYLSGLGLSRLICLPMALRAAIELNVFEIISQA
SOMT (Thalictrum flavum subsp. glaucum)	>tr|Q5C9L2|Q5C9L2_THLFG (S)-sco	355	MALQEGVNYLSGLGLSRLICLPMALRAAIELNVFEIIFQAGPEAQLSPAEIVAKIPTKNPNAAIALDRIL
SOMT1 (Papaver somniferum)	>sp|I3V6A7|SOMT1_PAPSO Scouleri	390	MATNGEIFNTYGHNRQTATVTKITASNESSNGVCYLSETANLGKLICIPMALRAAMELNVFQLISKFGTD
c2788/f1p0/674		149	MSCVAKAYPHVKCKSFDQPHVVAEAPVIPGVEFIGGDVFEFIPPADAISLKSFLHGSQDEECIKLLKRCK
c25582/f3p0/1524		349	MDAPVNYLSGFELLSRLTCFPMALRAAIDLNVFPIISGFGLDAKLSASQLVAQMPTTNPNAATALERILR
c27036/f4p6/1229		352	MDYKAQPVNYLSGFELLSRLACFPMALRAAIDLNVFQIISRFGPDAKLNSSQLVAEMPTTNPNAASALER
c37316/f10p2/1314		379	LPRNPKQPGKKILSHSKFLDFQEMEITINGGNNEQEMKWQGQVWSHICGFVDSTVLKTTLELSIFDTVHT
c37524/f6p1/1275		369	KHNSNSKAKGRRRRRSRRVKMGVNDIAEAQDVDIKAQAHLWNIIYGFADSLVLRCAVELGIADIINSNNG
c37677/f4p0/1270		356	MEITINEGNKEQEMKWQGQIWSHICGIVDSIVLKTTLELSIFDTVHTHTNSRISFSQLSKSPSLASIKPQ
c40307/f20p0/1422		360	FTVAEKMEGMSDLSLENEAVTIWKFIYGFCDTLVLKCCVNLEIADTIHKHGQPMTLSELASQLSVDLQKT
c40323/f7p0/1529		392	MAGSQDSQPHGIILNKDSYQSFEAEVQVYNYALSFVSSMSLKCAVQLGIPDIIHNHGSCKPITLSNLVDA
c40977/f3p3/1223		365	STQSTWGFTVAEKMEGMSDLSLENEAVTIWKFIYGFCDTLVLKCCVNLEIADTIHKHGQPMTLSELASQL
c14617/f1p0/1365		239	MLYLVQITLFSKETTSESGEIKYGLLPPAKYVVRGWQNSMVAALLLINDKNFIASLHYLKDGLGGECDAF
c17262/f2p4/1457		356	MEITINEGNKEQEMKWQGQIWSHICGIVDSIVLKTTLELNIFDTVHTHTNSIITFSHLSKSPSLASIKPQ
c20949/f1p2/1391		369	KHNSNSKAKGRGRRRSRRVKMGVNDIAEAQDVDIKAQAHLWNIIYGFADSLVLRCAVELGIADIINSNNG
c21040/f1p0/1573		356	MEITINEGNKEQEMKWQGQIWSHICGIVDSIVLQTTLELNIFDTVHTHTNSIITFSQLSKSPSLASIKPQ
c27717/f1p0/1347		373	SDPNRAELSSFELVTKFRESKRRNKMEVIKMSDQTDQAKLWKFIYGFADSLVLKCAVELEIADTIHKHGE
c28013/f1p0/1263		261	MAGLLTAMDMDFIAPWHLLEDGLDGRTDAFEVAFGKKVFDYLSENPKSSQLFNDYMAIHSSLLALELVKC
c28392/f1p0/1305		348	MEVIKKSDQTDQAKLWKFIYGFADSLVLKCAVELEIADTIHKHGEPMTLSELASQLPKQPIDADRLYRIM
c29075/f1p2/1221		330	MEVINKSDQTDQAKLWKFIYGFADSLVLKCAVELEIADTIHKHGDPMTLSELASQLPKQPIDADRLYRIM
c35877/f1p0/1959		406	TPTATNISLCLHLLSSIHSWFLEQRERERERVEKQRSIMANETSETRNNARLRILELANMISVPMALNAI
c3747/f1p5/1439		351	MEMTKSDQAVVAKIWNLAYGFADSLVVKCGVDLEIADTIHKHGEPMTLSEIASQLPKQPVDIDRLYRIMR
c38477/f1p0/1105		336	MEGMSDLSLENEAVTIWKFIYGFCDTLVLKCCVNLEIADTIHKHGQPMTLSELASQLSVDLQKTIDTDRL
c5439/f1p0/1823		372	RERVEKQRSIMANETSETRNNARLRILELANMISVPMALNAIVRLNVPDAIWQGGSNTPISATQILTRLQ
c8331/f1p2/1555		283	KHNSNSKAKGRRRRRSRRVKMGVNDIAEAQDVDIKAQAHLWNIIYGFADSLVLRCAVELGIADIINSNNG
c9140/f3p4/1432		392	IIHHPNSLPHFLLLLKKLDQLISSLTQMGSTENEIKTSATTPEEEEEACLYAMQLASASVLPMILKSAIE
c10669/f2p5/2283		356	MEITINEGNKEQEMKWQGQIWSHICGIVDSIVLQTTLELNIFDTVHTHTNSIITFSQLSKSPSLASIKPQ

RILRYLVKMEILRVEKSDDGQKKYALEPIATLLSRNAKRSMVPMILGMTQKDFMTPWHSMKDGLSDNGTAFEKAMGMTIWEYLEGHPDQSQLFNEGMAGETRLLTSSLISGSRDMFQGIDSLVDVGGGNGTTVKAISDAFPHIKCTLFDLF
VLRYLVHMGILKETKSTINGGEIKKLYSLEPVGSLLVKDAERNMVPIVLGMTQQDFMIPWHYIKEGLGEGSTAFEKGMGMTLWEYLEGHPEQGHLFNVGMEGETRLLTKTLIESCKDTFEGLSSLVDVGGGNGTTIKAISEAFPHIKCSLY
SDYLYRMVRYLVHLNIIEQETCNGGVEKVYSLKPVGTLLLRDAERSMVPMILGMTQKDFMVSWHFMKEGLGNGSTTAFEKGMGMDIWKYLEGNPDQSQLFNEGMAGETRLLTKTLIEDCRDTFQGLDSLVDIGGGNGTTIKAIYEAFPHIK
LYRLLRYLVHLNILGQQTCAAGVDRVYSLKPVGTLLLKDSERSMAPVILGLSQKDFLFVWNFVKEGLGTGSTTAFEKAMGMDMWKYLEVNPNQSQLFDEGQAGETRLLTKTLLVDCRDTFQGMDSLVDVGGGNGTTIKAIHEAFPHIKCTL
YLVHMKLFTKASIDGELRYGLAPPAKYLVKGWDKCMVGSILAITDKDFMAPWHYLKDGLSGESGTAFEKALGTNIWGYMAEHPEKNQLFNEAMANDSRLIMSALVKECGNIFNGITTLVDVGGGTGTAVRNIANAFPHIKCTVYDLPHVIA
MRYLVHMELFKIDATTQKYSLAPPAKYLLRGWEKSMVDSILCINDKDFLAPWHHLGDGLTGNCDAFEKALGKSIWVYMSVNPEKNQLFNAAMACDTRLVTSALANECKSIFSDGISTLVDVGGGTGTAVKAISKAFPDIKCTIYDLPHVIA
RYLVHMKLFTKSSIDGELRYGLAPPAKFLVKGWDKCMLGAILTITDKDFMAPWHYLKEGILNDGSTSTAFEKALGTNIWDYMAEHPEKNQLFNEGMANDTRLIMSALVKECSSMFDGITTIVDVGGGTGTAVRNIAKAFPHIKCTVYDLPH
HFMRYLVHMQLFTISTDQITKEDKYELTPASKLLVHGHQKSLAPYVMLQTHPEEFSVWSHVINVLDGKKPYWESNDTSMYEKTEGDPEINEILNDAMTSHSTFMLPALVSGLMKENVLDGVASIVDVGGNSGVVAKGIVDAFPHVKCSVMD
MRYLVHQKLFNKEVISTLNGGTVQVTEKYWLAPPAKYLIRGSQQSMVPSVLGIIDEDMFAPWHILKDSLTGECNIFETALGKSISVYMSENPEMNQISNGAMAFDSGLVTSHLVNECKSVFGDEIKTLVDVGGGTGTALRAISKAFPNIKC
RIMRLLVHKRLFTSELHQESNQLLYNLTRSSKWLLKDSKFNLSPLVLWETNPILLKPWQYLGKCAQEKSSPFERAHGCEIWDLALADPKFNNFLNGAMQCSTTTIINEMLLEYKDGFSGIAGSLVDVGGGTGSIIAEIVKAHPHIQGINFD
IFLDRILRLLGVSSILSMSTRAISPLSSSNGKIMNKVERVYGLTEESRSLVPREEDGVSLLPTLMFTTSKLVVESFFNLKGVVLQKNYVPFDMTHGVSIFEYACNEPKLNKGFNEAMGFFSVIVFEEVFRVYNGFVGMKELLDVGGGTGTS
GPDAQLSPSDIVAKIPTKNPSAAISLDRILRMLGASSILSVSTTKSGRVYGLNEESRCLVASEDKVSVVPMLLFTSDKAVVESFYNIKDVVLEEGVIPFDRTHGMDFFQYAGKEERVNKSFNQAMGAGSTIAFDEVFKVYKGFDNLKELVD
RMLGASSILSVTTMKDGRVYGLTEESRCLVADKNGVSVVPMLLFTSDKAVVESFYNIKDVVLEEGVIPFDRTHGMDFFAYAGKEQSVNKSFNQAMGAGSTIAFDEVFKVYKGFHDLKELVNVGGGIGTSLSNIIFKYPHIKGINFELPHVI
AKVSASEIASKMPNAKNNPEAAMYLDRILRLLGASSILSVSTTKKSINRGGDDVVVHEKLYGLTNSSCCLVPRQEDGVSLVEELLFTSDKVVVDSFFKLKCVVEEKDSVPFEVAHGAKIFEYAATEPRMNQVFNDGMAVFSIVVFEAVFRF
EVIPADKGKVIIIEIVLDQDEDDDELTTARVGLDIDTMLSSEGKERTKDEWRVLVQKAGYSRVEIIPIFAIQSVIVAYP
ILAANSLLSPSNELNGEISYGLTEDSRYLVPVQKDGVSLVPMVLLTINKYVMESFFQLKDAVLDEGCVPFDRTFGVSIFEFAGKEPKVGNIFNEAMRSSSSFVLDEVFKVYEGFDEMKELVDVGGGIGGTMSKIVSKFSHIHGINFDLPHV
ILRILAANSLLSSSNECNGEISYGLTKDSRYLIPDQKDGVSLVPMVLLSINKYVMESFFQLKDAVLDEGCVPFDRTFGVSIFEFAGKEPKVGKMFNEAMRSSSIYVLDEVLKVYEGFDEMNELVDVGGGIGGTMSKIVSNYPNIHGINFDL
HTNSLITFSQLSKSPSLASIKPQNLYRLLRYLVHLNLLTIKVVEGEETFSLTILSKLLLENKDKSLRDWSLGIDDPTSINIWHELSKFVTDPADAPTPFVQIHGKTLWELAGEIPEVNTLINNAMACDTRLVMPAVVQGCQELLNGISSLV
TVTISDIASKLPVDNVNEDNLYRVLRYLVYMGLLKESQDKCYSLEPVATLLLKDAQRSMVPIILGMTQKDFMVPWFFMKEGLGSGSTTAFEKGMGMTLWEYLEGHPDQSQLFNEGMAGETRLLTSSLINGCRDTFQGLTSLVDVGGGNGTT
NLNRMLRYLVHLNLLAIKVIEGEETFSLTNLSKLLLENQEKSLRDWSLGVNHQDLIEPWHELSKFVTDPADTPIPFVQIHGKTFWEWSGENPELNALINNTMASDSRLVMPAVVQGCQELFNGISSLVDIGGGTGAAMSCVAKAYPHVKCK
IDTDRLYRLMRYLVHLKFFTTEEGSDHLGEIKYGLLPLAKFLIRGWPKSMAGLLTAMDKDFIAPWHHLEDGLDGRTDAFEVAFGKKVFDYLSENPKSSQLFNDYMAIHSSLLALELVKCKNVFEDRIKTLVDAGGGTGTTAKAIANAFPHI
LSLPSTKTEYVHRLMRFLVHIGCFAHVNQEGFVLTPLSRLLLTDANNSLSSFLLGMLDPAMVTPWHFVSTWLRGTSSANNITPSGGADDGLMQTMPFEFAHGMNVWDFMKKNSDFNKTINEAMACDTRLVMNSVLNDPKVGEMVFNKNFTT
SVDLQKTIDTDRLYRLMRYLVHLKFFTTEEGSDHLGEIKYGLLPLAKFLIRGWPKSMAGLLTAMDKDFIAPWHHLEDGLDGRTDAFEVAFGKKVFDYLSENPKSSQLFNDYMAIHSSLLALELVKCKNVFEDRIKTLVDAGGGTGTTAKAI
EKANGKKIWDFMSENPEKNKLFNEAMACDSRLVTWALVQDCKDVFKGIKTLVDVGGGTGTAVKAISDAFPDIKCAVYDLPHVIADSPVAPNIDRIEGDMFKSIPNADAIFMKVNNLAISTCPFLSKLILIFLMIFHLIESSASSMIGTTTN
NLYRMLRYLVHLNLLTIQVVEGEETFSLTNLSKLLLETQEKSLRDWSLGIDNKISIDTWHELSNVVTDPADAPIPLVKIHGKTFWELAGENPELNTLINNTMACDSRMVMPAVVQGCPELFNGISSLVDIGGGTGTAMTCVAKAYPHVKCK
TVTISDIASKLPVDNVNEDNLYRVLRYLVYMGLLKESQDKCYSLEPVATLLLKDAQRSMVPIILGMTQKDFMVPWFFMKEGLGSGSTTAFEKGMGMTLWEYLEGHPDQSQLFNEGMAGETRLLTSSLINGCRDTFQGLTSLVDVGGGNGTT
NLYRMLRYLVHLNLLTIKVVEGEETFSLTNLSKLLLENQEKSLRDWSLGIDNKTSIDTWHELSNVVTDPADAPIPFVQIHGKTFWELAGEDPELNTLINNTMACDSRLVMPAVVQGCQELFNGISSLVDIGGGTGTAMACVAKAYPHVKCK
PMTLSELASQLPKQPIDADRLYRIMRYLVQIKLFSKETTSQSGEIKYGLLPPAKYVVRGWQNSMVAALLLINDKNFIASWHYLKDGLGGDCDAFEKANGKKIWDFMSENPKKNKLFNEAMACDSRLVTWALVQDCKDVFKGIKTLVDVGGG
KNVFEDRIKTLVDAGGGTGTTAKAIANAFPHIKCMVYELPHVNADAPVDPNIQRIDGDIFKSVPKADAILMQGVLHDWNDDECIQILKNCRESIPQDGGKVIIIEVVVNANSNHPYTKIMVLADLDMLIHGVRERTDEEWKKLLEAAGFTR
RYLVQIKLFSKETTSQSGEIKYGLLPPAKYVVRGWQNSMVAALLLINDKNFIASWHYLKDGLGGECDAFEKANGKKIWDFMSENPEKNKLFNEAMACDSRLVTWALVQDCKDVFKGIKTLVDVGGGTGTAVKAISDAFPDIKCAVYDLPHV
RYLFQIKLFSKETTSESGEIKYGLLPPAKYVVRGWLNSMVAALLLINDKNFIASWHYLKDGLGGECDAFEKANGKKICDFMSENPEKNKLFNEAMACDSRLVTWALVQDCKDVFKGIKTLVDVGGGTGTAVKAISDAFPDIKCAVYDLPHV
VRLNVPDAIWQGGSNTPISATQILTRLQSTGGDPENLQRILRMLTTYQVFTEHIILIDDEQTQHQQQQQNHRRLYSLTDIGKTLVTDQDGLSYAPYVIQHHQDALVKAWPLLHEAVLDSSVEPFVKANGEPAYGFYGDNPEMNELMLKAMS
YLVQMKLFTKEISSQSGEIKYGILPPAKFLIRAWDKSMVATILAFINKDFSAARLHLKDGLIGDCDPFEKAVGKNIWDYLSDNPEKNKEFNEAMASDSRLIAWTLVNDCKHVFEGIKTLVDVGGGTGTMVKAISNAFPKIKCIVYDLPHVI
YRLMRYLVHLKFFTTEEGSDHLGEIKYGLLPLAKFLIRGWPKSMAGLLTAMDKDFIAPWHHLEDGLDGRTDAFEVAFGKKVFDYLSENPKSSQLFNDYMAIHSSLLALELVKCKNVFEDRIKTLVDAGGGTGTTAKAIANAFPHIKCMVYE
STGGDPENLQRILRMLTTYQVFTEHIILIDDDQQQQNRRLYSLTDIGKTLVTDQDGLSYAPYVIQHHQDALVKAWPLLHEAVLDSSVEPFVKANGEPAYGFYGDNPEMNELMLKAMSGVSVPFMKAILEGYDDGFRGIQRLVDVGGSAGDC
TVTISDIASKLPVDNVNEENLYRVLRYLVYMGLLKESQDKCYSLEPVATLLLKDAQRSMVPIILGMTQKDFMVPWFFMKEGLGSGSTTAFEKGMGMTLWEYLEGHPDQSQLFNEGMAGETRLLTSSLINGCRDTFQGLTSLVDVGGGNGTT
LDLLEIMAKSGVGAYISPSELASKLPTTNPAAPVMLDRMLRLLASYKVLTCKLNTLDDGRVERLYGLAPVCKYLIKNEDGVSMAPLVLMNQDKVLMESWYYLKDTVLDGGIPFNKAYGMTAFEYHGTDPRFNKVFNRGMSDHTTITMKKIL
NLYRMLRYLVHLNLLTIKVVEGEETFSLTNLSKLLLENQEKSLRDWSLGIDNKTSIDTWHELSNVVTDPADAPIPFVQIHGKTFWELAGENPELNTLINNTMACDSRLVMPAVVQGCQELFNGISSLVDIGGGTGTAMACVAKAYPHVKCK

PHVIANSYDLPNIERIGGDMFKSVPSAQAIILKLILHDWNDEDSIKILKQCRNAVPKDGGKVIIVDVALDEESDHELSSTRLILDIDMLVNTGGKERTKEVWEKIVKSAGFSGCKIRHIAAIQSVIEVFP
DLPHVVADSHDLPNIEKIPGDIFKFIPNAQAILLKLILHDWSDEDSVKILKKCREAVPQDTGRVIIVDVALEEESEHPLTKTRLVLDVDMLVNTGGRERSEDDWAKLLKLAGFRTHKIRHIAAVQSVIEAFP
CTLYDLPHVVANSHDLPNIEKVPGDMFKSVPSAQAILLKLILHDWTDEECVNILKKCKEAIPKETGKVIIVDVALEEESNHELTKTRLILDIDMLVNTGGRERTADDWENLLKRAGFRSHKIRPIRAIQSVIEAFP
YDLPHVIANSDDHPNILKVPGDMFMSVPSAQVLLLKCVLHDWTDEHCVNILKKCKEAIPKETGKVIIVDVALEEESEHELTKARLILDIDMLVNTGGRERTAEDWENLLKRAGFRSHKIRPIRAIQSVIEAFP
DSPGYSEVHCVAGDMFKFIPKADAIMMKCILHDWDDKECIEILKRCKEAVPVKGGKVIIVDIVLNVQSEHPYTKMRLTLDLDMMLNTGGKERTEEEWKKLIHDAGYKGHKITQITAVQSVIEAYPY
DSPEIPNITKISGDMFKSIPSADAIFMKCILHDWNDDECIQILKRCKEALPKGGKVIIVDVVIDMDSTHPYAKIRLTLDLDMMLNTGGKERTKEEWKTLFDAAGFASHKVTQISAVQSVIEAYPY
VIADSPGYTEINSIQGDMFKYIPNADAIMMKCILHDWDDKECIEILKRCKDAVPRDGGKVIIIDIILDVKSEHPYTKMRLTLDLDMMLNTGGKERTEEEWKKLIHDAGYKGYKITHISAVQSVIEAYPY
LNHVIERVIKNPKLDYVAGDMFTSIPNADAILLKSTLHNYEDDDCIKILNIAKEALPSTGGKVILVEIVVDTENLPLFTSARLSMGMDMMLMSGKERTKKEWEDLLRKANFTSHQVIPIMAIESIIVAYS
TLFDLPHVIADSPEIPTITKVSGDMFKSIPSADAIFMKNILHDWNDDECIQILKRCKDVVSAGGKLIMVEMVLDEDSFHPYSKLRLTSDIDMMVNNGGKERTEKEWEKLFDAAGFASCKFTQMSVGFAAQSIIEVY
LPHVVATAAEFPGVKHVGGDMFVDIPEADAVIMKWILHDWSDEDCTIILKNCYRAIRKKKNGKVIIVDCVLRPDGNDLFDKMGLIFDVLMMAHTTAGKERTEAEWKILLNNAGFPRYNVIRTPAFPCIIEAFPE
ISKIVSKYPSIHGINFDLSHVISVAPQYPGIENIAGDMFEEVPKAQNILLNWVLHD
VGGGIGTSLSNIVAKHPHIRGINFELPHVIGDAPDYPGVEHVPGDMFEGVPNAQNILLKWVLHDWDDDRSIKILKNCWKALPENGTVIVIEFVLPQVLGNNAESFNALTPDLLMMALNPGGKERTTIEFDGLAKAAGFAETKFFPISQGL
ADAPNYPGVEHIAGNMFEGVPNAQNILLKWVLHDWDDERSIKILQNCWKALPEGGTVIVVEFVLPQILGNNAESFNALTPDLLMMTLNPGGKERTTTEFDGLAKAAGFAETKFFPISQGLHVMEFHKATAGVAS
YDGFLDMKELLDVGGGIGTSVSKIVAKYPLIRGVNFDLPHVISVAPQYPGVEHVAGDMFEEVPKGQNMLLKWVLHDWGDERCVKLLKNCWNSLPVGGKVLIIEFVLPNELGNNAESFNALIPDLLLMALNPGGKERTISEYDDLGKAAGF

IVDAPNYPGVKHISGDMFEEIPKAENIFLKWVLHDWDDESCKKLLRKCWNALDEGGKVIVIELVLPQVLGNNAESHSALAGDLMMMALSPGGKERTIIQFHNLAQAAGFNKVKSFPVNQGLHVIEFQK
PHVIADAPSYPGVKHISGDMFEEIPKAENIFLKWVLHDWDDESCKKLLRKCWNALDEGGKVIVIELVLPEVLGNNAESHSALAADLIMMALSPGGKERTIIQFHNLAQAAGFNIVKSFPVNQGLHVIEFQK
DIGGGTGAAMTYVAKAYPHVKCKSFDLPHVVPEAPAFPGVELIGGDMFEFIPPADAISLKFMLHNWLDEACIKLLKRCKEVIPADKGKVIIIDIVLDQDEDDDELTKARVSLDIDMMLSSGGKERTKEEWRVLVEKSGYSRVEIIPIFAI
IKGIYDAFPHIKCSVYDLPHVIANAHPHPNIERIPGDMFKSVPSAQAILLKLILHDWTDEESVDILKRCREAVPKEGGRVIIVDVALEEGSEHELTKTRLILDIDMLVNTGGRERTVDDWDRMLKLAGFSSHKIRHIAAIQSVIEAFP
SFDQPHVVAEAPVIPGVEFIGGDVFEFIPPADAISLKSFLHGSQDEECIKLLKRCKEVIPADKGKVIIIEIVLDQDEDDDELTTARVGLDIDTMLSSEGKERTKDEWRVLVQKAGYSRVEIIPIFAIQSVIVAYP
KCMVYELPHVNADAPVDPNIQRIDGDIFKSVPKADAILMQGVLHDWNDDECIQILKNCRESIPQDGGKVIIIEVVVNANSNHPYTKIMLLADLDMLIHGGRERTDEEWKKLLEAAGFTRYKLTEISAMHSIIEAYPYLS
LIDVGGGIGAAAISIAEAYPHLKCSVLDLPHVVAAATDVPRNNNNAVDVIEYIGGDMFEFIPHTDAIFLKYILHDWSDESCLKLLGRCKEAILPSKEKGGKLIIVEMVLEDQKKEHERTQAQYLLDVLLMVDTTGKERNEKQWESLFLKS
ANAFPHIKCMVYELPHVNADAPVDPNIQRIDGDIFKSVPKADAILMQGVLHDWNDDECIQILKNCRESIPQDGGKVIIIEVVVNANSNHPYTKIMLLADLDMLIHGGRERTDEEWKKLLEAAGFTRYKLTEISAMHSIIEAYPY
AFKYLSNVKRRYHETEVK
CLDLPHVVAEAPAFPGVELIGGDMFEFIPPADAISMKSILHSRQDETCIQLLKRCKEVIPADKGKVIIIDIVLDQDEDDDELTKARVSLDIDMMLNAGGKERTKDEWRVVVEKAGYSRVEIIPIFAIPSVIVAYP
IKGIYDAFPHIKCSVYDLPHVIANAHPHPNIERIPGDMFKSVPSAQAILLKLILHDWTDEESVDILKRCREAVPKEGGRVIIVDVALEEGSEHELTKTRLILDIDMLVNTGGRERTVDDWDRMLKLAGFSSHKIRHIAAIQSVIEAFP
SLDLPHVVAEAPAFPGVELFGGDMFEFIPPADAISLKSILHSWQDEACIKLLKRCKEVIPADKGKVIIIDIVLDQDEDDDELTKARVSLDIDMMMNAGGKERTKDEWRVLVEKAGYSRVEIIPIFAIPSVIVAYP
TGTAVKAISDAFPDIKCAVYDLPHVIADSPVAPNIDRIEGDMFKSIPNADAIFMKCILHDWNDDECIQILKQCKKALPRDGGKVIIVDVVLNVDSKHPYTKMRLTLDLDMMLNTGGKERTEEEWKELFEAAGFSGYKIIQTSALQSVIEA
YKLTEISAMHSIIEAYPISYFFVFVFLSSTPCCLVRLRNY
IADSPVAPNIDRIEGDMFKSIPNADAIFMKCILHDWNDDECIQILKQCKKALPQDGGKVIIVDVVLNVDSKHPYTKMRLTLDLDMMLNTGGKERTEEEWKELFEAAGFSGYKIIQTSALQSVIEAYP
IADSPVAPNIDRIEGYMFKSIPNADAIFMKCILHDWNDDECIQILKQCKNALPRDGGKVIIVDVVLNVDSKHPYTKMRLTFDLDMMLNTGGKEMTEEEWKELFEAAGFS
GVSVPFMKAILDGYDDGFRGIQRLVDVGGSAGDCLKMIMMKFPDISQGINFDLPEVVAKAPNISGVKHIGGDMFKSIPEGDAIFMKWVLTTWTDDECQVIMKNCFNALPQGGKLIACEPVLPHESDTSHRTRALLEGDIFVMTIYRAKGK
ADSSIHHNPNIVRVEGDMFKFIPNAEAILMKWILHDWADYECIQILKLCKEAIPQDGGKVIIVDIVLNVYSEHPYTKARLATDLDMLVGGTGGKERTEEEWKKLLEAAGFTAYKITEISAVQSVIEAFPC
LPHVNADAPVDPNIQRIDGDIFKSVPKADAILMQGVLHDWNDDECIQILKNCRESIPQDGGKVIIIEVVVNANSNHPYTKIMLLADLDMLIHGGRERTDEEWKKLLEAAGFTRYK
LKMIMNKFPDISQGINFDLPEVVAKAPNISGVKHIGGDMFKSIPEGDAIFMKWVLTTWTDDECQVIMKNCFNALPQGGKLIACEPVLPHESDTSHRTRALLEGDIFVMTIYRAKGKHRTEDEFRQLGLSSGFPHFRAIYIDHFYTLLEFQ
IKGIYDAFPHIKCSVYDLPHVIANAHPHPNIERIPGDMFKSVPSAQAILLKVRLLYPYIFKG
ETYKGFEGLNSIVDVGGGIGATVSMIVSKYPSIKGINFDLPHVIEDAPTYPGVEHVGGDMFASVPKGDAIFMKWILHDWSDEHNLKTLKNCYEALPANGKVIIAESILPVFPETNAAASGVFHVDVIMLAHNPGGKERTEKEFEALAKEA
SLDLPHVVAEAPAFPGVELFGGDMFEFIPPADAISLKSILHSWQDEACIKLLKRCKEVIPADKGKVIIIDIVLDQDEDDDELTKARVSLDIDMMMNAGGKERTKDEWRVLVEKAGYSRVEIIPIFAIPSVIVAYP

HVMEFHKINC

IKTIPIPISNGLHVIEFHK

QSVIVAYP

GFNHYKITSLVGLRSLIEVYP

YP

HRTEDEFRQLGLSSGFPHFRAIYIDHFYTLLEFQK

K

GFSGFKVVCCAYNSWIMEFCK
